# Predictive framework for codend size selection of brown shrimp (*Crangon crangon*) in the North Sea beam-trawl fishery

**DOI:** 10.1371/journal.pone.0200464

**Published:** 2018-07-16

**Authors:** Juan Santos, Bent Herrmann, Daniel Stepputtis, Claudia Günther, Bente Limmer, Bernd Mieske, Sebastian Schultz, Thomas Neudecker, Axel Temming, Marc Hufnagl, Eckhard Bethke, Gerd Kraus

**Affiliations:** 1 Thünen Institute of Baltic Sea Fisheries, Rostock, Germany; 2 Fishing Gear Technology, SINTEF Fisheries and Aquaculture, Hirtshals, Denmark; 3 Norwegian College of Fishery and Aquatic Science, University of Tromsø, Tromsø, Norway; 4 Institute for Hydrobiology and Fisheries Sciences, University of Hamburg, Hamburg, Germany; 5 Thünen Institute of Sea Fisheries, Hamburg, Germany; Texas A&M University, UNITED STATES

## Abstract

The brown shrimp (*Crangon crangon*) fishery is of great socio-economic importance to coastal communities on the North Sea. The fishery is exploited by beam trawlers often using codends with very small mesh sizes, leading to concerns about catch rates of undersized shrimp. However, little information is available on codend size selection, making it difficult to provide scientifically based advice on alternative codend designs. Therefore, this study establishes a predictive framework for codend size selection of brown shrimp, based on a large selectivity dataset from 33 different codend designs tested during four experimental fishing cruises, during which more than 350,000 brown shrimp were length measured. Predictions by the framework confirm concerns about the exploitation pattern in the fishery, because the retention probability of undersized shrimp reaches 95% with the currently applied designs. The framework predictions allow the exploration of obtainable exploitation patterns depending on codend design. For example, increasing codend mesh size to 25–29 mm would reduce the retention rate of undersized shrimp to a maximum of 50%, depending on codend mesh type.

## Introduction

The brown shrimp (*Crangon crangon*) fishery is socio-economically one of the most important fisheries in the North Sea [1,2]. It supports an international fleet of approximately 560 vessels [3], employing more than 1,000 fishermen, and producing yearly revenues of up to ~€100 million [4]. Landings have been consistently larger than 30,000 tonnes since 2003, with Dutch and German beam-trawl fleets in the length category 10–30 m making up approximately 90% of the total landings [5].

Despite its relevance, the brown shrimp fishery is one of the least regulated fisheries in European waters [6]. The European fishery management applied to this fishery does not include quotas or fishing-effort restrictions, while the minimum landing size is based on market preferences, since only shrimps with total lengths >50 mm are commercially exploited [7]. However, in recent years, brown shrimp producer organisations have initiated a certification process of the fishery by the Marine Stewardship Council. A key finding for successful certification was the need to investigate gear technology on codend selectivity, which should improve the exploitation patterns of brown shrimp [1,4].

Codends made of diamond mesh (mesh in standard net orientation, T0) with small mesh sizes of ca. 20–22 mm are currently used to avoid the loss of commercial sizes of brown shrimp, at the expense of a large bycatch of small individuals [2,8,9,10]. One obvious strategy for reducing unwanted catches of small shrimp would be to increase the mesh size, but it has not been determined what that mesh size should be. Revill and Holst [9] studied the selectivity of diamond-mesh codends with mesh sizes of 16, 22, 24, and 26 mm, but only relative changes in selectivity were estimated. Therefore, the effect of mesh size on codend selectivity of brown shrimp is largely unknown and prevents the identification of optimal codend mesh size. In addition to mesh size, it has been demonstrated that altering codend mesh geometry using square mesh or T90 mesh (mesh orientation turned by 90°) can provide better selectivity for crustaceans than standard diamond-mesh codends [11–14]. Therefore, square-mesh codends or T90 codends may be alternative codend designs for the brown shrimp fishery. However, this leads again to the question: What mesh size should be used for such codends?

It is the objective of this study to fill the knowledge gap about codend selectivity in the brown shrimp fishery, by developing a framework for predicting codend size selectivity for different mesh sizes and mesh types. This framework will allow the prediction of size-selective retention probabilities for brown shrimp in codends varying in mesh size and mesh type, including diamond-mesh, square-mesh, and T90 codends. The predictive framework is intended to improve decision-making about fishery exploitation patterns.

## Material and methods

### Ethic statement

Experimental fishing was conducted on board a German Fishing Research Vessel owned and operated by the Federal Office for Agriculture and Food (BLE), the legal entity which regulates and controls the fishing activity in German waters. The use of the Research Vessel for conducting the fishing trials implicitly granted the authors with the fishing permission from the German authorities. No other authorization or ethics board approval was required to conduct the study. Information on animal welfare and steps to ameliorate suffering and methods of sacrifice is not applicable, since the animals were not exposed to any additional stress other than that involved in commercial fishing practices. This study did not involve endangered or protected species.

### Experimental codends

**Fig 1:**
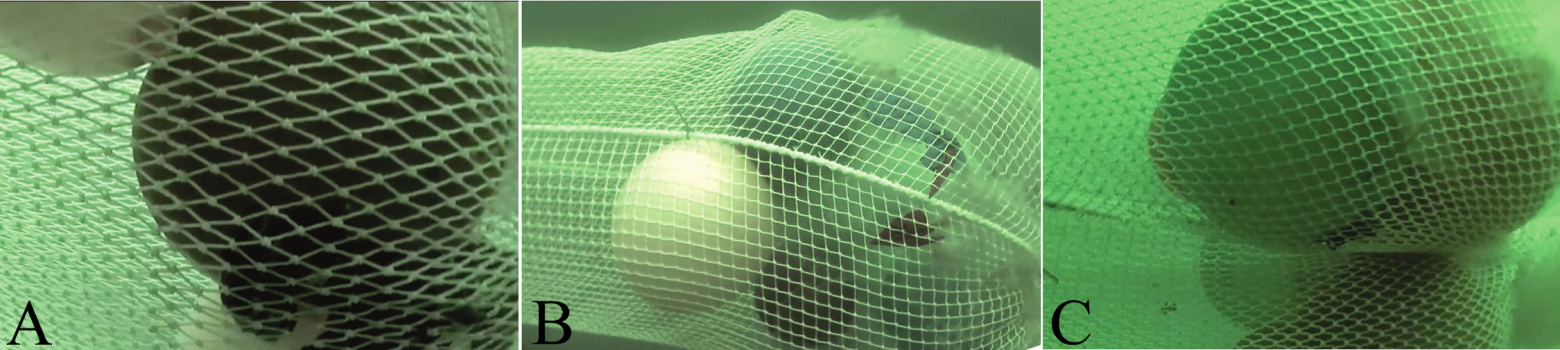
Netting configurations applied in the experimental codends. (A) traditional diamond mesh (T0), (B) square mesh (T45), (C) T90 mesh. A, B, and C Pictures were taken underwater using plastic balls to simulate catch volume.

A total of 33 different codends were used for experimental fishing. Among them, 13 were made of standard diamond mesh, with mesh sizes ranging from ~19 mm to ~36 mm; 8 square-mesh codends, with mesh sizes ranging from ~17 mm to ~29 mm; and 12 T90 codends, with mesh sizes ranging from ~19 mm to ~36 mm. The square-mesh codends were constructed using standard diamond netting turned 45° (T45), and the netting used for T90 codends was turned 90° (Fig 1). Codend mesh sizes were measured using OMEGA gauge according to Fonteyne *et al*. [15].

The number of meshes in circumference applied in the codends decreased with mesh size to attempt neutralising the potential effect of the number of meshes on codend size selectivity [16]. All codends were made of 210 Deniers–mass in grams per 9,000 m of the fibre–PA twine netting.

### Sea trials and experimental design

Experimental fishing was conducted during four cruises on board the German research vessel *RV/Solea* (42 m, 950 kW) in commercial fishing grounds off the Wadden Sea coast, in the south-eastern part of the North Sea. Three of the cruises were conducted during the main fishing season (April, September, and November 2013), and one was conducted during low season in January 2013. The collection of size-selection data was based on the paired gear method [17]. Two identical beam trawls, similar to those used in the commercial fishery were mounted on each side of the vessel. The beam width was 7 m with a U-shaped groundrope of 9.2 m. The vertical opening of the net was 0.5 m. The trawl bodies were made of standard diamond netting with 30 mm mesh size in the front, decreasing to 20 mm in the rear. A sieve net of 60 mm mesh size [18] was mounted in both trawls, as it is mandatory in the commercial fishery to reduce fish bycatch. During each of the experimental hauls, one of the two nets mounted a control codend of 11 mm nominal mesh size, and the other mounted one of the 33 test codends. The mesh size of the control codend was not measured, since its nominal value met the lower limit of measurable sizes by the Omega gauge (10 mm ± 1 mm precision). Control and test codends were exchanged between trawls after several hauls to level out potential differences in catch efficiency between the two trawls. It was assumed that the control codend was not selective for the relevant sizes of brown shrimp; therefore, catches from this codend were considered representative of the population available for the experimental test codends. The differences in catches observed in the control and test codend were used to estimate the size-selection properties of the test codends at haul level.

Catch sampling was carried out for each codend separately. Brown shrimp catches were sorted from the bulk of the catch and weighted. A random subsample of brown shrimp was collected from the total catch and frozen for later length measurement in the laboratory. Once in the laboratory, the subsamples were thawed and placed on a plate to be photographed. The total length of each individual was obtained by digital image analysis. Total lengths where rounded to the half millimetre below, to provide count data of the number of shrimp in each half millimetre-width length groups for the subsequent size selectivity analysis.

### Assessing the selectivity of individual hauls

The logistic function [17] was used to describe the size selection of each experimental haul:

$$r\left(\text{l,L50,SR}\right)=\frac{\text{exp}\left(\frac{\text{ln}\left(9\right)\times \left(l-L\text{50}\right)}{\text{SR}}\right)}{1+\text{exp}\left(\frac{\text{ln}\left(9\right)\times \left(l-L\text{50}\right)}{\text{SR}}\right)}$$ (1)

Eq (1) quantifies the probability that a brown shrimp with length *l* will be retained in the test codend. It is defined by two selectivity parameters: *L50* represents the shrimp length with 50% retention probability, and *SR* (selection range) represents the range of lengths between 75% and 25% retention probabilities. The two selectivity parameters were obtained at haul level by modelling the length-dependent catch share of brown shrimp between the test and control codends. Assuming that the selectivity of the test codend can be described by the logistic function (Eq (1)), the proportion of a given length class *l* in the test codend to the total catch can be modelled by:

$$\varphi \left(l,L50,SR,SP\right)=\frac{SPxr\left(l,L50,SR\right)}{\left(1-SP\right)+SPxr\left(l,L50,SR\right)}$$ (2)

where the split parameter (*SP*) is the length-independent probability for brown shrimp to enter the test codend. A value of *SP* ~0.5 indicates an equal probability of entering the control or the test codend; *SP* >0.5 indicates a greater probability of entering the test codend. Although the *SP* parameter is not of primary interest in this study, its estimation is required to estimate the selection parameters of the test codend correctly [19]. The value for parameters *L50*, *SR*, and *SP* were obtained by minimising the following likelihood function:

$$\begin{array}{c} \sum_{l}\{{nt}_{l}\times ln\left(\frac{qt\times \varphi \left(l,L50,SR,SP\right)}{qt\times \varphi \left(l,L50,SR,SP\right)+qc\times \left(1-\varphi \left(l,L50,SR,SP\right)\right)}\right)+\\ {nc}_{l}\times ln\left(\frac{qc\times \left(1-\varphi \left(l,L50,SR,SP\right)\right)}{qt\times \varphi \left(l,L50,SR,SP\right)+qc\times \left(1-\varphi \left(l,L50,SR,SP\right)\right)}\right)\} \end{array}$$ (3)

In Eq (3), *nt*_*l*_ is the number of shrimps with length *l* measured in the test codend; *nc*_*l*_ is the number of shrimps with length *l* measured in control codend. Values qt and qc are the length-independent subsampling factors, calculated as the ratios of shrimp length measured to the total catch of shrimps for the test and control codends, respectively.

The ability of Eq (2) to describe the experimental data sufficiently well was evaluated based on the model *p*-value, model deviance vs. degrees of freedom (DOF), and inspection of how the model curve reflects the length-based trend in the data [17]. In case of a poor fit statistics (*p*-value being <0.05; deviance being >>DOF), the predicted curve from the analysed haul was inspected to determine whether the poor result was caused by structural problems when describing the experimental data, or by overdispersion in the data [17]. In case of no clear pattern in deviation, it was assumed that poor fit statistics would be the result of overdispersion in data, and the specific haul would be kept for further analysis.

### Meta-analysis of codend selectivity

The values of *L50*, *SR*, and *SP*, estimated for each experimental haul (Eqs (1–3)), were used to model the variation of brown shrimp codend selectivity over the range of experimental codend designs tested during the four research cruises. The meta-analysis was conducted using the Fryer method [20], which quantifies the influence of a set of fixed factors on the experimental codend selectivity, including codend mesh size and other factors measured at haul level. Further, the Fryer method accounts for uncertainty in the estimation of selectivity for individual hauls owing to finite sample sizes, known as within-haul variation, and between-haul variation caused by variations in the fishing process due to uncontrolled changes in fishing conditions. We therefore applied the Fryer method to the experimental selectivity parameters, including information on their covariance (within-haul variation) and a set of fixed factors, as follows:

$$L50_{mean}=\alpha_{0}+\alpha_{1}\times m+\alpha_{2}\times m^{2}+\alpha_{3}\times w+\alpha_{4}\times m\times w+\alpha_{5}\times s+\alpha_{6}\times p$$

$$SR_{mean}=\beta_{0}+\beta_{1}\times m+\beta_{2}\times m^{2}+\beta_{3}\times w+\beta_{4}\times m\times w+\beta_{5}\times s+\beta_{6}\times p$$

$$SP_{mean}=\gamma_{0}+\gamma_{1}\times m+\gamma_{2}\times m^{2}+\gamma_{3}\times w+\gamma_{4}\times m\times w+\gamma_{5}\times s+\gamma_{6}\times p$$ (4)

In Eq (4), the coefficients *α*_*j*_, *β*_*j*_, and *γ*_*j*_ (*j* = 0,…,6) quantify the effect of each of the fixed factors on *L50*_*mean*_, *SR*_*mean*_, and *SP*_*mean*_, respectively. The first terms considered are the intercept terms *α*_*0*_, *β*_*0*_, and *γ*_*0*_. The effect of mesh size (*m*) is accounted for by the terms *α*_*1*_ *× m*, *β*_*1*_ *× m*, *γ*_*1*_ *× m*. The second-order terms *α*_*2*_ *× m*^*2*^, *β*_*2*_ *× m*^*2*^, and *γ*_*2*_ *× m*^*2*^ were considered because exploratory scatterplots indicated a potential non-linear relationship between the values of the experimental size selectivity parameters and mesh size. Some studies have demonstrated that codend catch weight in some cases can affect codend size selection [10,21]. Consequently, the potential effect of catch weight (*w*) in the test codend is accounted for by the terms *α*_*3*_ *× w*, *β*_*3*_ *× w*, and *γ*_*3*_ *× w*, while *α*_*4*_ *× m×w*, *β*_*4*_ *× m×w*, and *γ*_*4*_ *× m×w* quantify the potential interaction effect between catch weight and codend mesh size. Additionally, the sea state can potentially influence codend selectivity [22]. Therefore, sea state (*s*) was measured using the vessel’s bridge facilities (scale range 0–9). An average value of *s* was calculated for every experimental haul and accounted for by the terms *α*_*5*_ *× s*, *β*_*5*_ *× s*, and *γ*_*5*_ *× s*. Beam trawls can have different fishing power, even in the twin configuration used in this study. Therefore, the mounting position *p* of the test codend was recorded for each haul (port = 0 or starboard = 1), and included in Eq (4) as *α*_*5*_ *× p*, *β*_*5*_ *× p*, and *γ*_*5*_ *× p*.

For the analysis based on Eq (4), it is required that the size selection of the experimental codends included in the model have comparable between-haul variability. However, this assumption might not be fulfilled for codends with different mesh orientation [16]. For this reason, Eq (4) was applied separately for the three different mesh configurations considered in the study (diamond-mesh, square-mesh, and T90 codends).

Based on the full model (Eq (4)) with 21 fixed factors, many submodels can be formulated leaving out one or more terms at a time for *L50*_*mean*_ and/or *SR*_*mean*_ and/or *SP*_*mean*_. Considering all combinations, this led to 2,097,152 different competing models that were all candidates to model the size selection in diamond-mesh, square-mesh, and T90 codends separately. Analogue to the procedure in Wienbeck et al. [16], the candidate models were automatically ranked by decreasing value of AICc [23], and the model with the lowest AICc was selected to predict the size selection for diamond-mesh, square-mesh, and T90 codends, respectively.

Before the selected models could be used, it was necessary to validate their predictive capabilities, by inspecting their ability to describe the main trends in codend selectivity observed experimentally. This was done by plotting the *L50* and *SR* values obtained from the experimental hauls against the predicted values, which involve considering the mean predictions (*L50*_*mean*_, *SR*_*mean*_), the uncertainty in mean parameters (*varL50*_*mean*_, *varSR*_*mean*_), and the estimated between-haul variation (*D*_*L50*_, *D*_*SR*_). Therefore, the following lower and upper 95% limits of the CI for *L50*_*mean*_ and *SR*_*mean*_ were used in the comparisons:

$$limL50_{mean}=l50_{mean}\pm 1.96\times \sqrt{varL50+D_{L50}}$$

$$limSR_{mean}=SR_{mean}\pm 1.96\times \sqrt{varSR+D_{SR}}$$ (5)

### Predictive framework

Once the predictive capabilities of the selected models for diamond-mesh, square-mesh, and T90 codends were validated, they were applied to calculate the size of brown shrimp *L*_*r*_ associated to given retention probabilities *r* from a wide range of codend designs, differing in mesh size separately for the three types of codends investigated:

$$L_{r}=L50_{mean}+\frac{SR_{mean}}{ln\left(9\right)}\times ln\left(\frac{0.01\times r}{1.0-0.01\times r}\right)$$ (6)

The retention probabilities assessed in Eq (6) ranged from *r* = 0.05 to *r* = 0.95, with intermediate probabilities in steps of 0.05. Retention probabilities were plotted in percentage terms (for ease of reading) against codend mesh size and their associated *L*_*r*_, providing isolines of codend retention probability. Sizes of shrimp with less than 5% retention probability (*r* <0.05) and sizes of shrimp with more than 95% retention probability (*r* >0.95) were considered to be fully released or fully retained by the codend, respectively.

**Fig 2:**
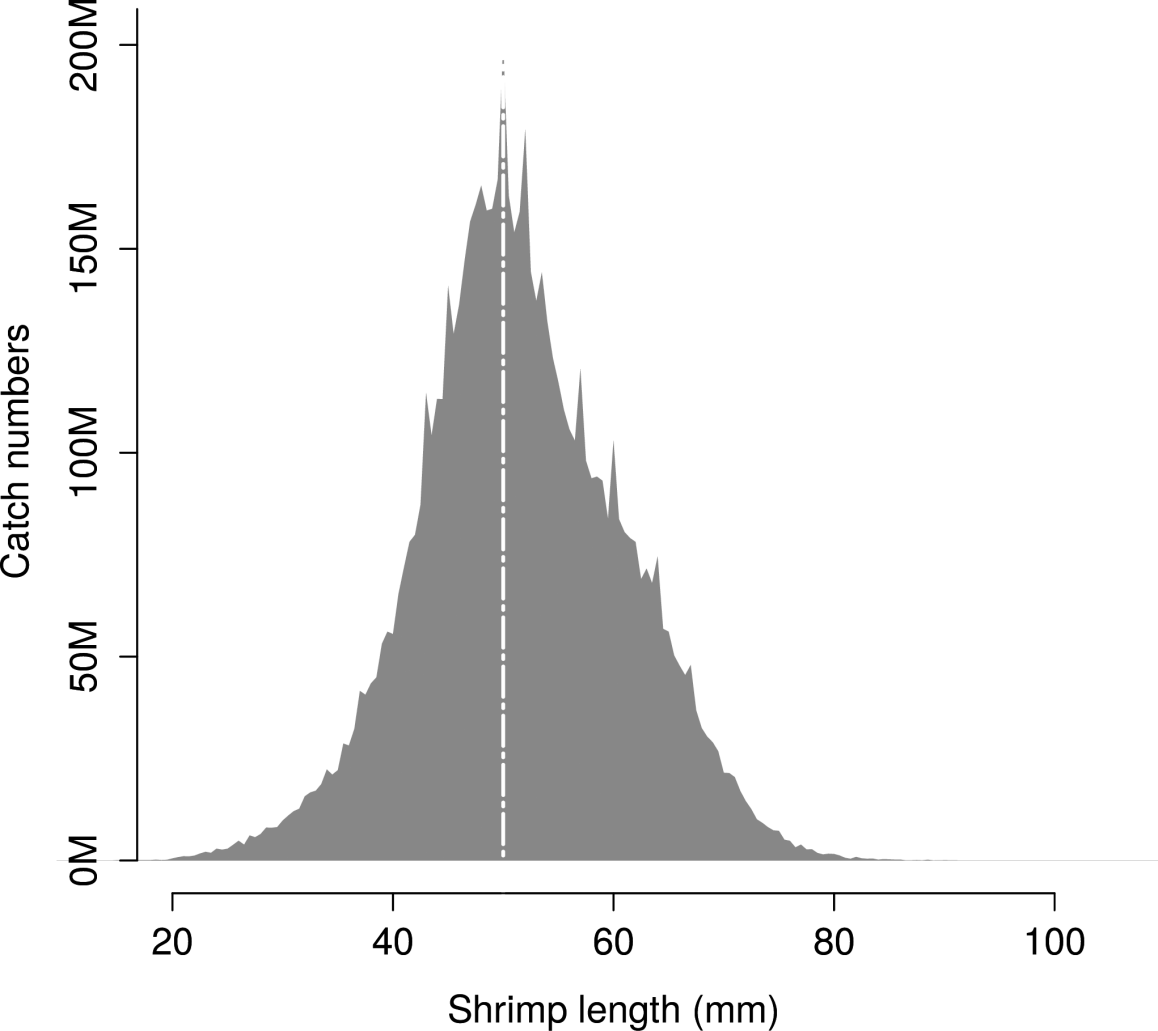
Size structure of the brown shrimp population fished during the sea trials. Numbers (in thousands, M) obtained after pooling the catches from the control codend over the experimental hauls.

Although the predictive framework of retention probability provides information independent of the size structure of the available brown shrimp population, it is of interest to give an example of codend performance for a given population structure. This assessment was conducted by applying the predictive capabilities of the selected models on the size structure of brown shrimp population (*nPop*_*l*_) used during the sea trials. The structure of *nPop*_*l*_ was therefore obtained by pooling the brown shrimp catches from the control codend over the experimental hauls (Fig 2). The predicted size-selection curve for a given codend design was applied to *nPop*_*l*_, to produce simulated catches (*nCatch*_*l*_) of brown shrimp. Based on *nPop*_*l*_ and *nCatch*_*l*_, the following codend usability indicators were calculated for diamond-mesh, square-mesh, and T90 codends with 21 mm (midpoint mesh size considering the commercial range), 23 mm, 25 mm, 27 mm, and 29 mm mesh sizes:

$$nR=\frac{100\times \sum_{l<mls}nCatch_{l}}{\sum_{l}nCatch_{l}}$$

$$nP=\frac{100\times \sum_{l}nCatch_{l}}{\sum_{l}nPop_{l}}$$

$$nPa=\frac{100\times \sum_{l\geq mls}nCatch_{l}}{\sum_{l\geq mls}nPop_{l}}$$

$$nPb=\frac{100\times \sum_{l<mls}nCatch_{l}}{\sum_{l<mls}nPop_{l}}$$ (7)

where *mls* = 50 mm is the minimum landing size established by the fleet for market reasons [7]; *nR* is the percentage of catches of undersized shrimp relative to the total catch, therefore, quantifying the expected proportion of brown shrimp bycatch associated with a given codend. The indicator *nP* represents the percentage of brown shrimp entering the codend, which is finally caught, providing information on the length-independent retention efficiency of the codend. Finally, *nPa* and *nPb* indicate codend retention efficiencies for length ranges of brown shrimp greater than and less than *mls*, respectively. Therefore, a codend with a good compromise between retention of commercial shrimp and release of undersized shrimp would have *nR* and *nPb* values close to 0%, and an *nPa* value close to 100%. All analysis described above were conducted with the software SELNET [24].

## Results

**Fig 3:**
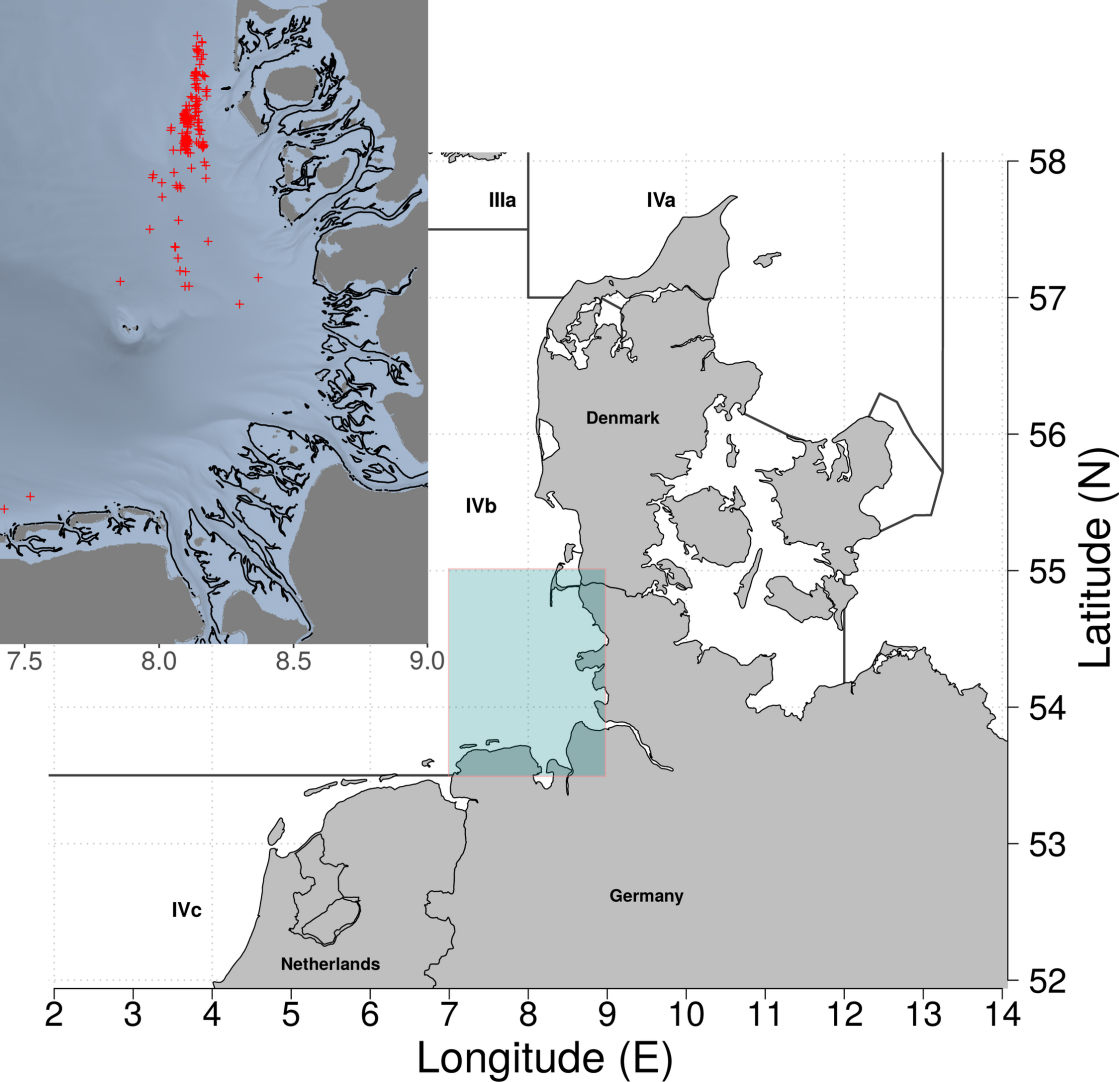
Location of the experimental hauls conducted during the four different cruises.

A total of 208 hauls were conducted during the four research cruises carried out in January (27 hauls), April (85 hauls), September (63 hauls), and November (33 hauls) 2013. Most of the hauls were conducted in a rectangle defined between 54.25N-55.00N and 008.00E-008.40E, in the German waters of the Wadden Sea (Fig 3 and S1 Table). In all, 89 hauls were conducted using diamond-mesh codends, 51 using square-mesh codends, and 68 using T90 codends (Table 1). Beam trawls were towed at a speed of ~3 knots with a towing duration of 60 min, on fishing grounds between 11 and 24 m deep, confirming that fishing conditions were similar among hauls, codends and cruises (S1 Table). In 75% of the experimental hauls, the catch weight in the test codend did not exceed 40 kg. On average, smaller catches in the test codends were observed during the first two cruises (14.98 kg, (standard deviation, s.d. = 5.24) and 8.80 kg (s.d. = 4.73)) compared with the catches in September and November (50.43 kg (s.d. = 46.50) and 82.41 kg (s.d. = 39.53)). Shrimp catches were systematically subsampled, and approximately 1 kg of shrimp was used for length measurement per codend. In total, 160,612 brown shrimp were measured from hauls using diamond-mesh test codends, 85,304 individual measurements were obtained from hauls using square-mesh codends, and 109,451 individual measurements from hauls using T90 codends. The subsampled factors ranged between 0.03 and 0.47 in test codends and between 0.02 and 0.26 in the control codend (Table 1).

**Table 1:** List of codends tested in experimental trials, and brown shrimp catch information related to each codend.

Test codend						Control codend		
Codend type	Codend mesh size (mm)	Number of hauls	Number measured	sub-sample factor (qt)	Lengths (mm)	Number measured	sub-sample factor (qc)	Lengths (mm)
Diamond mesh	19.05 (0.07)	6	6857	0.06	50.6 (21.0–81.0)	7414	0.06	49.1 (12.5–83.0)
(T0)	20.19 (0.34)	9	8780	0.18	50.0 (10.5–86.5)	10037	0.13	47.9 (13.5–93.0)
	21.45 (0.07)	9	7158	0.26	51.1 (24.5–90.0)	8498	0.21	49.0 (19.5–81.5)
	22.95 (0.19)	6	5376	0.09	53.2 (24.5–84.0)	5765	0.07	50.3 (15.0–84.0)
	24.65 (0.07)	6	4441	0.28	53.7 (25.5–82.5)	5054	0.20	50.1 (20.5–85.0)
	25.10 (0.29)	5	4482	0.20	51.9 (18.0–82.0)	4707	0.15	49.8 (19.0–83.5)
	27.15 (0.60)	6	5665	0.07	55.2 (25.5–89.5)	8692	0.03	48.0 (20.0–88.5)
	27.83 (0.24)	8	5537	0.16	54.9 (17.5–81.0)	7453	0.09	49.2 (19.5–81.0)
	29.35 (0.26)	5	3351	0.12	54.3 (18.0–86.5)	4331	0.05	50.5 (15.0–80.5)
	31.58 (0.28)	6	4643	0.23	54.7 (24.0–88.0)	6549	0.05	48.0 (20.0–82.0)
	32.25 (0.13)	6	3602	0.23	58.2 (25.0–81.0)	4816	0.19	51.3 (22.5–85.5)
	32.28 (0.13)	12	7899	0.34	56.5 (23.0–87.0)	10828	0.18	50.4 (15.0–87.5)
	36.38 (0.45)	5	3639	0.41	57.4 (33.5–95.5)	5038	0.13	51.2 (20.0–87.5)
Square mesh	17.25 (0.07)	2	1900	0.47	48.7 (18.5–75.5)	2312	0.26	47.3 (21.5–83.0)
(T45)	18.75 (0.21)	5	4483	0.09	52.4 (24.5–81.0)	4545	0.08	51.6 (11.0–80.5)
	20.98 (0.88)	12	8577	0.06	55.2 (23.0–84.5)	9161	0.06	52.5 (18.5–89.5)
	23.40 (0.12)	6	4943	0.07	54.6 (26.0–85.0)	6704	0.04	49.1 (19.5–87.5)
	24.95 (0.21)	8	6387	0.08	54.6 (20.5–87.5)	8172	0.04	49.7 (17.5–85.5)
	25.20 (0.18)	6	3232	0.11	57.0 (35.0–81.5)	3615	0.07	53 (20.5–80.0)
	27.78 (0.15)	6	4040	0.12	56.8 (27.5–86.0)	6546	0.05	49.1 (15.0–99.5)
	29.28 (0.21)	6	4392	0.29	55.9 (24.5–87.0)	6295	0.13	49.4 (17.5–85.0)
T90	18.88 (0.33)	6	5133	0.10	52.3 (20.5–84.5)	6729	0.06	49.1 (16.5–89.0)
	20.18 (0.56)	3	3581	0.11	49.0 (25.5–82.0)	3568	0.09	47.4 (14.5–76.5)
	21.15 (0.49)	6	3678	0.15	55.6 (27.5–82.0)	4248	0.14	52.6 (20.5–77.5)
	22.50 (0.48)	6	5690	0.18	51.4 (9.5–79.0)	6670	0.13	48.3 (10.0–80.5)
	24.35 (0.66)	7	4221	0.27	57.2 (33.5–81.5)	4672	0.22	53.9 (21.5–83.0)
	24.63 (0.59)	5	3800	0.09	57.8 (24.5–86.0)	4308	0.03	52 (20.0–92.5)
	27.55 (0.13)	6	3923	0.06	56.5 (26.0–88.5)	5793	0.02	51.5 (20.0–90.0)
	27.83 (0.29)	6	3363	0.36	59.0 (28.5–79.5)	3766	0.19	53.8 (26.0–84.0)
	29.03 (0.63)	6	4174	0.04	55.8 (25.0–85.5)	5741	0.03	50.6 (21–87.5)
	31.28 (0.55)	6	3254	0.29	59.0 (30.5–85.0)	3984	0.18	51.7 (14.5–80.5)
	31.40 (0.34)	5	3686	0.05	55.5 (21.0–88.0)	5102	0.03	50.4 (17.0–88.5)
	36.50 (0.20)	6	4498	0.03	56.1 (24.5–88.5)	5869	0.02	51.0 (20.5–84.0)

Average mesh size of the test codends with standard deviation (sd, in brackets) was taken as the inner distance from knot to knot in stretched meshes (sd in brackets), obtained using OMEGA gauge. Number of brown shrimps measured in test and control codend obtained after pooling catches from all hauls conducted with a given codend. Description of length structure of brown shrimp caught in the test and control codends includes the mean length and the length range (in brackets) found in the measured sub-samples. Sub-sampled factors presented are averaged over hauls conducted with each of the tested codends.

### Size selectivity of individual hauls

A visual inspection of the experimental data demonstrated clear size-selection trends (S1 Fig), except in three hauls (two hauls using diamond-mesh codends and one haul using a T90 codend). For these three hauls, it was not possible to estimate the covariance matrix of the selectivity parameters. Therefore, these hauls were excluded from further analysis. The selectivity parameters (*L50* and *SR*), the split parameter (*SP*), and the covariance of the remaining 205 hauls were successfully estimated using Eqs (1–3). For all 205 valid hauls, the selectivity parameters, fit statistics, the characteristics of the codends used (codend type and mesh size), and the additional fixed factors considered for the subsequent meta-analysis (Eq (4)) are summarised in S2 Table. The inspection of the fit statistics for each estimation on haul level resulted in 39 fits (~19% of the total estimated models) with *p*-values <0.05. However, inspection of the residuals associated with these hauls did not show any systematic trend. Therefore, it was decided to use the selectivity data from all 205 hauls for subsequent analysis.

### Meta-analysis of codend selectivity

**Fig 4:**
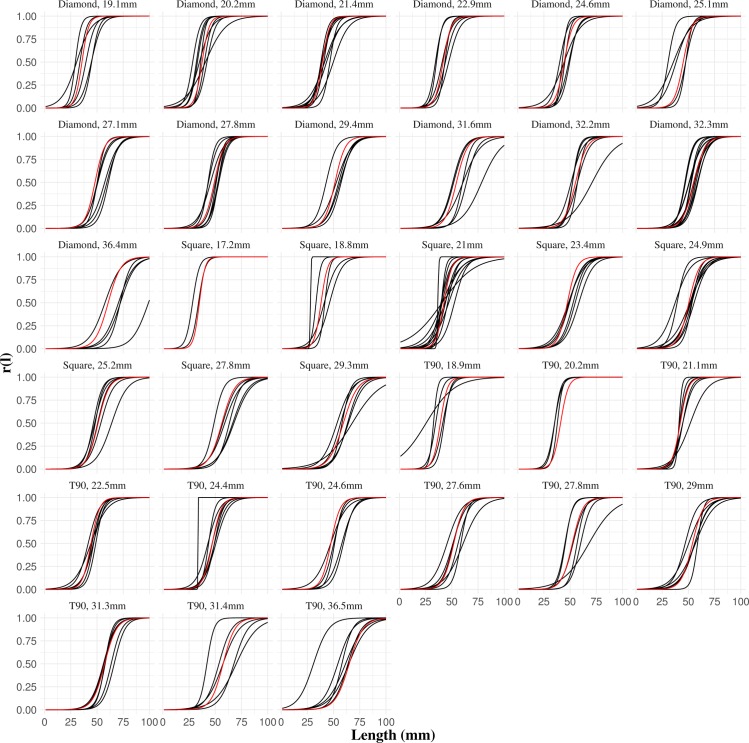
Predicted size selection curves for each of the experimental codends. For visual comparison, the mean curves predicted by the selected models (red lines) for each codend are plotted together with the size selection curves obtained experimentally (black lines).

The Fryer method was successfully applied in the selectivity meta-analysis for diamond-mesh, square-mesh, and T90 codends. The results from the full model (Eq (4)) and the associated 2,097,151 reduced models for each codend type were ranked by increasing the AICc value. The models with lowest AICc values for each codend type were selected. The three resulting models allowed predictions of the mean size selectivity for each of the codends used in experimental fishing (Fig 4). In general, the position of the predicted size-selection curves in relation to the distribution of experimental curves indicates good predictive capabilities of the selected models. Further details from each of the selected models are described in the subsections below.

#### Model for diamond-mesh codends

The selected model for predicting diamond-mesh codend selectivity has the following fixed factor structure:

$$L50_{mean}=\alpha_{1}\times m+\alpha_{2}\times m^{2}$$

$$SR_{mean}=\beta_{1}\times m$$

$$SP_{mean}=\gamma_{0}$$ (8)

**Fig 5:**
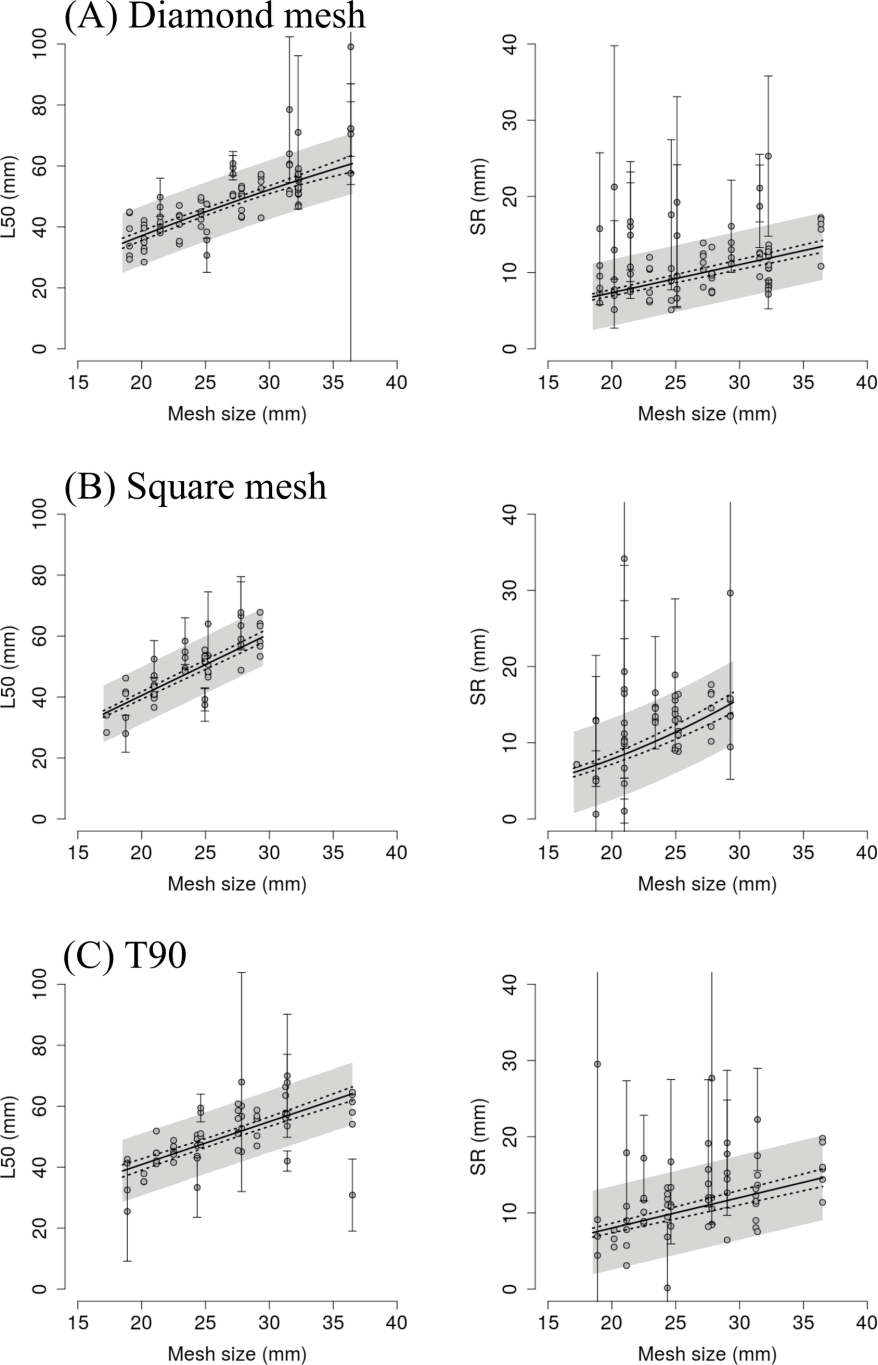
***L50* and *SR* mean values (solid line) estimated by the predictive models for the three different codend mesh types ((A) Diamond mesh, (B) Square mesh, and (C) T90).** Predictions are plotted against the L50 and SR values obtained from individual hauls (circle marks). Dotted lines represent the CIs accounting for the uncertainty of the estimation, while the grey band represents the CIs accounting for the total variation in the data, including the between-haul variation. CIs associated to experimental values (vertical lines) only plotted for the experimental points falling outside the grey band. *SR* predictions for the square-mesh codends and *L50* predictions for T90 codends were estimated using a fixed total catch weight of 35 kg, a value near the mean total catches observed in the test codends.

Only four of the terms used in the full model structure (Eq (4)) were kept in the selected model. The linear (*m*) and quadratic mesh size (*m*^*2*^) were the two factors used to describe the experimental *L50* values. The effect of the linear term is positive and stronger than the negative value of the quadratic term (Table 2), resulting in a nearly linear trend with a positive slope over the range of experimental mesh sizes (Fig 5). *SR*_*mean*_ increases linearly with mesh size, and no other term was found influential (Table 2 and Fig 5). The structure of the *SP* equation only included the intercept term, which was found to be not significantly different from 0.5 (*γ*_*0*_ = 0.49 (0.48–0.52)), indicating equal probability that brown shrimp entered either the control or the test codend.

**Table 2:** Estimated parameters of the selected predictive models for different mesh types (diamond, square and T90).

Mesh configuration	Parameter	Fixed factor	Coefficient	Value	SE	95% CI	p-value
Diamond-mesh	L50 (mm)	m	α_1_	2.05	0.12	1.821 to 2.287	<0.001
		m²	α_2_	-0.01	>0.01	-0.018 to -0.002	0.011
	SR (mm)	m	β_1_	0.37	0.01	0.350 to 0.396	<0.001
	SP	intercept	γ_0_	0.49	0.01	0.476 to 0.518	<0.001
Square-mesh	L50 (mm)	m	α_1_	2.02	0.03	1.960 to 2.082	<0.001
	SR (mm)	m²	β_2_	0.02	>0.01	0.014 to 0.017	<0.001
		w	β_3_	0.04	>0.01	0.023 to 0.053	<0.001
	SP	intercept	γ_0_	0.51	0.01	0.484 to 0.540	<0.001
T90	L50 (mm)	m	α_1_	1.93	0.03	1.866 to 1.988	<0.001
		w	α_2_	0.31	0.06	0.193 to 0.438	<0.001
		mxw	α_3_	-0.01	>0.01	-0.017 to -0.009	<0.001
	SR (mm)	m	β_1_	0.40	0.02	0.366 to 0.427	<0.001
	SP	intercept	γ_0_	0.51	0.01	0.483 to 0.530	<0.001
**Between haul variation**		Diamond-mesh	Square-mesh	T90			
	D_11_	26.14	22.11	26.44			
	D_22_	6.38	7.93	7.43			
	D_33_	0.01	0.01	0.01			

Top: fixed factors included in the model matrix (see also Eqs (8–10)). Bottom: Diagonal of the D-Matrix presenting the estimated between-haul variation of the selective parameters. SE = standard errors, CI = confidence interval.

The *L50*_*mean*_ and *SR*_*mean*_ predictions from Eq (8) describe well the experimental data (Fig 5), because most of the experimental values fall within the CI of *L50*_*mean*_ and *SR*_*mean*_, whereas the outer points overlap their own CIs with the CI from the predictions.

#### Model for square-mesh codends

The selected model for square-mesh codends has the following structure:

$$L50_{mean}=\alpha_{1}\times m$$

$$SR_{mean}=\beta_{2}\times m^{2}+\beta_{3}\times w$$

$$SP_{mean}=\gamma_{0}$$ (9)

Mesh size (*m*) was the only fixed factor included in Eq (9) to describe the distribution of the experimental *L50* values. The estimated linear coefficient was similar to the one estimated for diamond-mesh codends (Table 2); however, the lack of a quadratic term results in a linear trend of *L50*_*mean*_ over the range of mesh sizes (Fig 5). The fixed-factors structure related to *SR* is more complex, combining the effect of the second-order polynomial mesh size (*m*^*2*^) and catch weight (*w*). The value of both terms is positive (Table 2). Therefore, *SR*_*mean*_ also increases with increasing catch weight. As for diamond-mesh codends, the predicted *SP*_*mean*_ was not significantly different from 0.5 (Table 2).

Predictions from Eq (9) using a fixed catch weight of 35 kg (a value near the average catch weight obtained during the sea trials in the test codend) describe well the distribution of the experimental *L50* and *SR* obtained by square-mesh codends (Fig 5), with all experimental values or their respective CIs falling within the CI of the predictions.

### Model for T90 codends

The selected model for T90 codends has the following structure:

$$L50_{mean}=\alpha_{1}\times m+\alpha_{3}\times w+\alpha_{4}\times m\times w$$

$$SR_{mean}=\beta_{1}\times m$$

$$SP_{mean}=\gamma_{0}$$ (10)

Eq (10) includes the fixed-factors mesh size (*m*), catch weight (*w*), and the interaction term (*m × w*) to describe the variation of experimental *L50* values obtained with T90 codends. The main-factors mesh size and catch weight affect the *L50*_*mean*_ positively, whereas the negative value of the interaction term (Table 2) lead to an opposite effect of catch weight depending on mesh size. In particular, increasing catch weight increases *L50*_*mean*_ for mesh sizes less than 25 mm, whereas the opposite effect is predicted for mesh sizes greater than 25 mm. As with the diamond-mesh model (Eq (8)), the T90 model incorporated only the linear mesh size term (*m*) for *SR*, and the associated coefficients are very similar (Table 2). As in Eqs (8) and (9), only the intercept was used to describe the experimental *SP* values, and the predicted *SP*_*mean*_ was not significantly different from 0.5 (Table 2).

Predictions from Eq (10) using 35 kg as fixed catch weight describes well the distribution of the experimental *L50* and *SR* obtained by T90 codends (Fig 5), with most experimental values or their respective CIs falling within the CI of the predictions.

### Predictive framework

**Fig 6:**
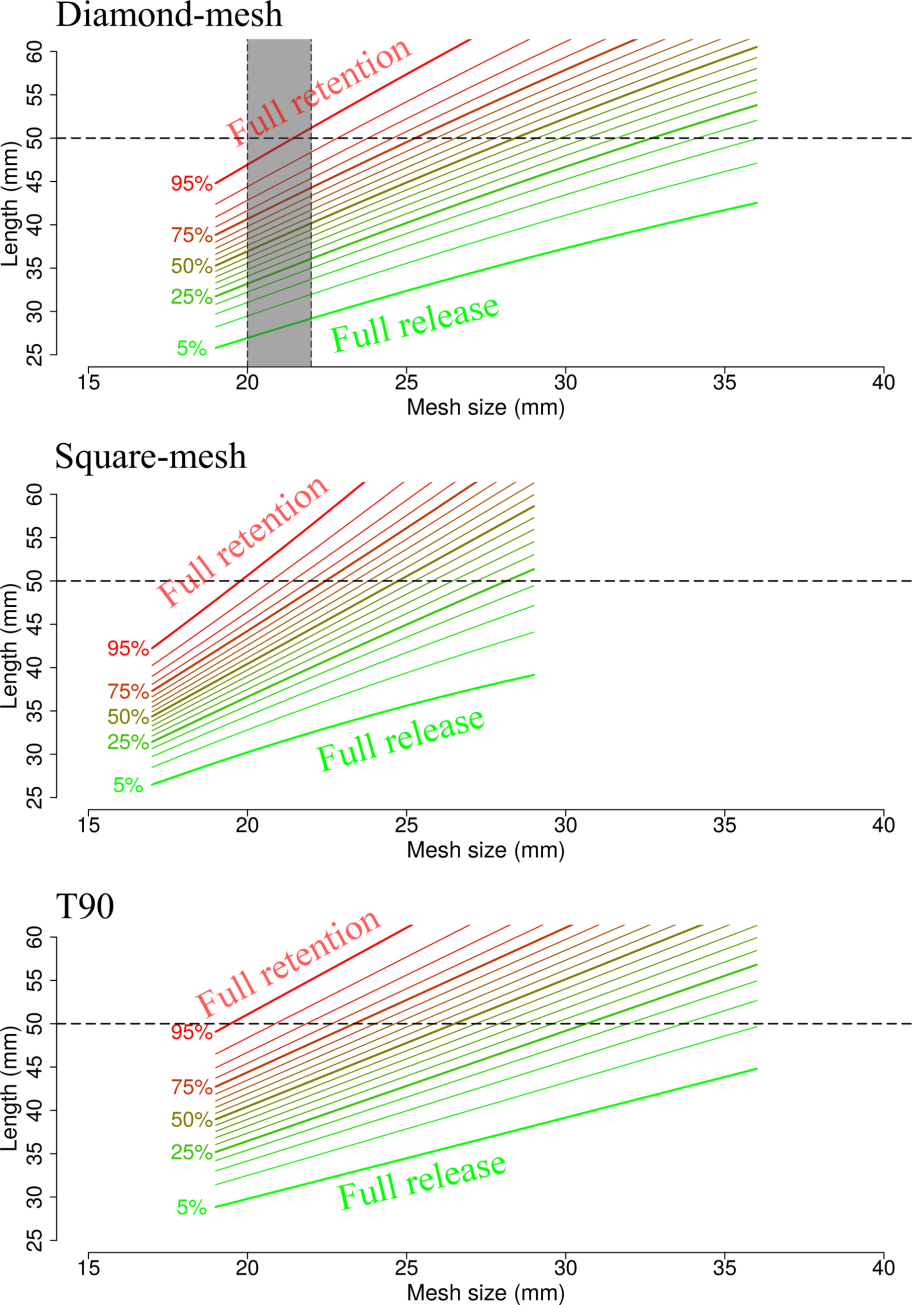
Isolines of predicted retention probabilities (5%–95% in steps of 5%). Grey bars represent the range of codend mesh sizes currently used in the fishery. Horizontal dashed lines represent minimum commercial size at 50 mm shrimp length (See S3 Table).

The good predictive capabilities of the three models shown in Figs 4 and 5 allowed the establishment of the predictive framework. Isolines in Fig 6 describing codend retention probabilities for the three different mesh types, covering the range of mesh sizes applied experimentally, were estimated using the established framework (see S3 Table to assess the numerical values used in Fig 6). Predictions for square-mesh and T90 codend were obtained assuming a fixed catch weight of 35 kg. Isolines of retention probability show that lengths equal to or greater than *mls* = 50 mm are fully retained (greater than 95% retention probability) by diamond-mesh codends between 20 and 21 mm. The retention probability in this range of mesh size remains great for smaller lengths. For example, the retention probability for 45 mm brown shrimp is still greater than 80%. The framework reveals that it would require codends with diamond-mesh size of 29 mm to reduce the retention probability for the species *mls* to ca. 50%, whereas the probability of retaining 45 mm brown shrimp individuals would drop to 25%. On the other hand, the framework predicts retention probabilities between 80% and 95% for square-mesh and T90 codends using commercial mesh sizes between 20 and 22 mm. Applying a square-mesh codend with a mesh size of ~25 mm, or a T90 mesh size of ~27 mm, would result in a retention efficiency similar to diamond-mesh codends with 29 mm mesh size.

The percentage of undersized shrimp relative to the total catch (*nR* indicator in Eq (7)) for the diamond-mesh codend with commercial mesh size of 21 mm is ~42% (Table 3). For the same codend, it is expected to catch 85 of every 100 shrimps entering the codend (*nP* ~85%). As reflected by the isolines for this codend design, the indicator *nPa* shows nearly full retention efficiency for individuals equal to or greater than species *mls* (*nPa* ~99%), at the expense of retaining a large number of undersized shrimp (*nPb* ~72%). By changing the mesh type to square mesh or T90, a considerable reduction in the retention efficiency of the undersized shrimp (*nPb* ~55% and *nPb* ~57% for square-mesh and T90 codends, respectively) is expected, without considerable effects on the retention efficiency for marketable sizes (*nPa* ~96%). Using a T90 codend with 23 mm mesh size, or increasing the diamond-mesh size to 25 mm, would reduce the retention efficiency for undersized shrimps to less than 50% (*nPb* ~43%), while maintaining the retention efficiency for marketable shrimps at greater than 90% (*nPa* ~91%). Implementing any of these codend designs would reduce bycatch to *nR* = ~32%. Likewise, applying either a square-mesh codend with 25 mm mesh size, a T90 codend with 27 mm mesh size, or increasing the diamond-mesh size to 29 mm would reduce the retention efficiency for undersized shrimps to values smaller than 25% (nPb ~23 and *nPb* = 21.5, respectively), while the retention efficiency for the commercial shrimp would be reduced to nearly 75% (nPa ~74%).

**Table 3:** Values for the codend usability indicators nR, nP, nPa nPb.

Mesh size (mm)	Mesh configuration	nR (%)	nP (%)	nPa (%)	nPb (%)
21	Diamond	42.31	85.48	98.79	72.21
	Square	36.47	75.48	96.07	54.96
	T90	37.41	76.93	96.47	57.46
23	Diamond	37.62	77.08	96.32	57.9
	Square	29.28	61.8	87.55	36.13
	T90	32.18	67.45	91.64	43.34
25	Diamond	32.21	67.21	91.28	43.22
	Square	23.57	48.28	73.92	22.72
	T90	27.03	57.38	83.89	30.97
27	Diamond	26.95	56.93	83.32	30.63
	Square	20.18	36.76	58.77	14.81
	T90	22.63	47.60	73.78	21.50
29	Diamond	22.55	47.19	73.22	21.25
	Square	18.74	27.79	45.25	10.40
	T90	19.26	38.71	62.61	14.88

Predictions for the square-mesh codends and T90 codends considering a fixed catch weight of 35 kg.

## Discussion

This study attempts to fill gaps in knowledge of codend size selection in the brown shrimp beam-trawl fishery in the North Sea. Based on a comprehensive dataset derived from experimental fishing, we deliver a framework to predict the codend size selectivity for a wide range of codend designs. These predictions can be used to identify suitable codend specifications under a given harvesting strategy.

Polet [10] studied the selectivity of a standard codend with commercial mesh size of 21.7 mm, estimating *L50* = 39.4 mm (37.0–41.4 mm) and *SR* = 11.6 mm (10.2–13.0). As a benchmarking exercise, we used our tool to predict the selectivity parameters for a codend with the same mesh size and mesh configuration, giving an *L50*_*mean*_ = 39.8 mm (38.2–41.3 mm), nearly the same *L50* as Polet [10], and a slightly lower *SR*_*mean*_ = 8.0 mm (7.5–8.5). These similar results, obtained from the different studies–and different experimental designs–support the usability of our predictive framework.

In addition to the ability to predict the size-selection parameters for a wide set of codend designs, the predictive framework is further applied to estimate codend usability indicators. It is important to mention here that the given codend usability indicators depend on the actual population structure and so need to be recalculated if they are applied to other brown shrimp populations.

The predictions highlight the very poor selectivity delivered by the codends currently used in the commercial fishery. For a 21 mm diamond-mesh size, the framework predicts almost full retention for the *mls* of brown shrimp (Fig 6 and Table 3). Accordingly, the *nPa* value (retention of marketable sizes) for this codend was estimated to be ~99%. Simultaneously, the *nR* (rate of bycatch of undersized shrimp) indicator was ~40%, which indicates an average bycatch rate of ca. 40% in the fishery for this codend and population structure. Using the predictive capabilities of the framework provide different alternatives to mitigate the bycatch problem. For example, it would be possible to reduce the bycatch rate by half (*nR* ~20%), while maintaining the catchability of commercial sizes greater than 70%, by implementing either codends with ~29 mm diamond-mesh size, square-mesh codends with ~25 mm mesh size, or T90 codends with ~27 mm mesh size. This example illustrates how the framework can provide predictions and thereby recommendations on codend design, suitable for specific management strategies.

This study quantifies for the first time the selectivity properties of square-mesh and T90 codends in the North Sea brown shrimp fishery. As with diamond-mesh codends, the present analysis shows *L50*_*mean*_ and *SR*_*mean*_ values from square-mesh and T90 codends increasing with increasing mesh sizes. The predictions using a fixed catch weight of 35 kg show *L50*_*mean*_ values of T90 codends to be greater than those of diamond-mesh codends of the same mesh size. For example, the *L50*_*mean*_ expected for the mesh size of 21.7 mm used by Polet [10] is *L50* = 43.3 mm (41.6–44.9 mm) for T90 mesh configuration, a value significantly greater than the *L50*_*mean*_ value expected from the diamond-mesh codend. Square-mesh codends with small mesh sizes provide *L50*_*mean*_ values similar to T90 codends; however, differences arise for mesh sizes greater than 25 mm. For example, the expected *L50*_*mean*_ for square-mesh codends at 26 mm mesh size is estimated to be 52.7 mm (51.0–54.2 mm), whereas for T90 it is estimated to be *L50*_*mean*_ = 49.3 mm (47.9–50.8 mm). The *SR*_*mean*_ for diamond-mesh and T90 codends is similar, presenting a linear trend over mesh size, while the *SR*_*mean*_ for square-mesh codends present a quadratic functional form, making the estimated values significantly greater than the diamond-mesh and T90 estimations for mesh sizes larger than 25 mm. Based on these results, it is demonstrated that mesh type influences significantly the size selectivity of brown shrimp. Therefore, mesh type should be considered together with mesh size in the search for specific harvesting strategies.

In addition to mesh size, catch weight (*w*), sea state (*s*), and the side on which the test codend was mounted (*p*) were considered to be fixed factors in the development of the predictive framework. The selected model for diamond-mesh codends only incorporated mesh size (*m*) and the square of mesh size (*m*^*2*^) as influential terms; therefore, the effect of the remaining factors was not sufficiently strong to be selected by AICc. This result contrasts with the results obtained by Polet [10], who estimated negative and positive effects of catch weight and sea state on *L50* values, respectively. Contrary to the method applied in this study, Polet [10] did not account for the between-haul variation in the analysis. Because the methodology applied in this study meets the standard approach to multivariate regression modelling in size-selection studies, we consider the results of our approach to be reliable.

Only mesh size was necessary to explain the *L50* variation from square-mesh codends, whereas the structure for *SR*_*mean*_ includes mesh size and catch weight as influential effects. We speculate that the positive effect of catch weight on *SR*_*mean*_ might be related to decreasing possibilities for brown shrimp to contact the codend meshes, or an increasing longitudinal deformation of the meshes with increasing catch weight, providing different escapement opportunities for a given length of shrimp. The positive effect of catch weight on *L50*_*mean*_ for T90 codends with meshes smaller than 25 mm agrees with the effect found for roundfish species in diamond-mesh codends [25]; however, the presence of the negative interaction term in Eq (10) leads to an opposite effect of catch weigh for T90 codends with mesh sizes larger than 25 mm, in accordance with results previously obtained for T90 codends and very large catches [26]. The ambivalent effect of catch weight on *L50*_*mean*_ for T90 codends should be interpreted with caution owing to the low catch weights obtained in this study, less than catch weights usually found in the commercial fishery. To what extent this could influence the applicability of our results is unknown. Further investigations aiming to obtain larger catches, and involving theoretical studies of brown shrimp selectivity, would be required to better understand the effect of catch size on the selectivity of square-mesh and T90 codends.

In addition to *L50* and *SR* (Eq (4)), the parameter *SP* was modelled to detect any factor compromising the entrance of brown shrimp in the test codends. The three selected models (Eqs 7–9) only accounted for the intercept term to describe experimental *SP* values. Therefore, there is no indication that any of the fixed factors (included mesh size) affect the probability that brown shrimp will enter the test codend.

The analysis applied in this study allows the quantification of non-controlled variation between the experimental hauls [20]. Because the modelling was conducted separately for diamond-mesh, square-mesh, and T90 codends, their between-haul variation can be compared. Although the between-haul variation associated with *L50* and *SR* was great, no remarkable differences were found between the three different mesh orientations (Table 2). This result indicates that applying either square-mesh or T90 codends in the commercial fishery would not result in a greater variation in the size-selection patterns of the fleet, compared with the currently applied diamond-mesh codends.

Often, size-selection studies focus on investigating individual codend designs to meet specific needs in a given fishery. As the needs change with time, similar studies are repeated over decades without a clear and unified strategy [27]. Our approach goes beyond the standard strategy. Based on a comprehensive dataset, collected during a single year, the predictive framework presented in this study can provide advice regarding the expected selectivity of a wide span of codend designs. This is the basis to support current and future scientifically based management decisions to be applied in the North Sea brown shrimp fishery.

## Supporting information

S1 Table Operational information of the test hauls. Geographical coordinates (decimal degrees) refer to the start and end of each haul. Operational information is completed with towing direction (°), distance towed (in nautical miles, nm), and the average fishing depth in meters (m) (n.a. = not available). Hauls ordered by codend type, mesh size, and cruise.
(DOCX)

S2 Table Results from the selectivity analysis for individual hauls. Catch weight, sea state and trawl side are the fixed factors measured at haul level, and included together with mesh size in the meta-analysis. Standard deviation of the estimated *L50*, *SR*, and *SP* are shown in brackets. CO11 to CO33 are the vectorised form of the covariance matrix from the estimated *L50*, *SR*, and *SP*. Hauls ordered by codend type, mesh size, and cruise.
(DOCX)

S3 Table Predicted lengths of brown shrimps associated to given retention probabilities (from *r* = 5% to *r* = 95%) for different codend types and mesh sizes. Values *L50*_*mean*_ and *SR*_*mean*_ estimated by the predictive framework. The numerical information presented here was used to plot the isolines for codend retention in Fig 5.
(DOCX)

S1 Fig By-haul catch sharing between control and test codends. Grey polygon and black line represent the length distributions of brown shrimp in control and test codends, respectively. Mark circles represent the experimental catch proportion in the test codend relative to the total catch, obtained upon raised data. Blue line is the φ(l) curve estimated according to Eqs 2 and 3. Dotted red line represents species minimum landing size at 50 mm length. Hauls ordered by codend type, mesh size, and cruise.
(PDF)

## References

[1] Neudecker T, Damm U. The by-catch situation in German brown shrimp (*Crangon crangon L*.) fisheries with particular reference to plaice (*Pleuronectes platessa L*.). Journal of Applied Ichthyology. 2010;26(s1):67–74.

[2] Catchpole T, Revill A, Innes J, Pascoe S. Evaluating the efficacy of technical measures: a case study of selection device legislation in the UK *Crangon crangon* (brown shrimp) fishery. ICES Journal of Marine Science.2008;65(2):267–275.

[3] Anom. Report of the Working Group on Crangon Fisheries and Life History (WGCRAN). Imuiden, the Netherlands: ICES; 2015. CM 2015/SS-GEPD:07.

[4] Aviat D, Diamantis C, Neudecker T, Berkenhagen J, Müller M. The North Sea brown shrimp fisheries. Directorate-General for Internal Policies, European Parliament; 2011. Available from: http://www.europarl.europa.eu/studies

[5] Anom. Report of the Working Group on Crangon Fisheries and Life History (WGCRAN). Oostende, Belgium: ICES; 2016. CM 2016/SSGEPD:07.

[6] Tulp I, Chen C, Haslob H, Schulte K, Siegel V, Steenbergen J, et al Annual brown shrimp (*Crangon crangon*) biomass production in Northwestern Europe contrasted to annual landings. ICES Journal of Marine Science. 2016;73(10):2539–2551.

[7] Hufnagl M, Temming A. Growth in the brown shrimp Crangon crangon. II. Meta-analysis and modelling. Marine Ecology Progress Series. 2011;435:155–172.

[8] Ulleweit J, Stransky C, Panten K. Discards and discarding practices in German fisheries in the North Sea and Northeast Atlantic during 2002–2008. Journal of Applied Ichthyology. 2010;26(s1):54–66.

[9] Revill AS, Holst R. Reducing discards of North Sea brown shrimp (*C*. *crangon*) by trawl modification. Fisheries Research. 2004;68(1):113–122.

[10] Polet H. Codend and whole trawl selectivity of a shrimp beam trawl used in the North Sea. Fisheries research. 2000;48(2):167–183.

[11] Deval M, Özgen G, Özbilgin H. Selectivity of 50 mm T0 and T90 codends for commercial shrimp species in the Turkish deepwater trawl fishery, Eastern Mediterranean. Journal of Applied Ichthyology. 2016;32(6):1041–1057.

[12] Broadhurst MK, Millar RB, Kennelly SJ, Macbeth WG, Young DJ, Gray CA. Selectivity of conventional diamond-and novel square-mesh codends in an Australian estuarine penaeid-trawl fishery. Fisheries Research. 2004;67(2):183–194.

[13] Campos A, Fonseca P, Erzini K. Size selectivity of diamond and square mesh cod ends for rose shrimp (*Parapenaeus longirostris*) and Norway lobster (*Nephrops norvegicus*) off the Portuguese south coast. Fisheries Research. 2002;58(3):281–301.

[14] Thorsteinsson G. The use of square mesh codends in the Icelandic shrimp (*Pandalus borealis*) fishery. Fisheries research. 1992;13(3):255–266.

[15] Fonteyne R, Gabriele B, Leonori I, O’Neill FG. Review of mesh measurement methodologies. Fisheries Research. 2007;85:279–284.

[16] Wienbeck H, Herrmann B, Moderhak W, Stepputtis D. Effect of netting direction and number of meshes around on size selection in the codend for Baltic cod (*Gadus morhua*). Fisheries Research. 2011;109(1):80–88.

[17] Wileman DA, Ferro RST, Fonteyne R, Millar RB (Editors). Manual of methods of measuring the selectivity of towed fishing gears. ICES cooperative research report. 1996;215:38–99.

[18] Revill A, Holst R. The selective properties of some sieve nets. Fisheries Research. 2004;66(2–3):171–183.

[19] Millar R, Walsh S. Analysis of trawl selectivity studies with an application to trouser trawls. Fisheries Research. 1992;13(3):205–220.

[20] Fryer RJ. A model of between haul variation in selectivity. ICES Journal of Marine Science. 1991;48:281–290.

[21] O’Neill FG, Knudsen LH, Wileman DA, McKay SJ. Cod-end drag as a function of catch size and towing speed. Fisheries Research. 2005;72(2–3):163–171.

[22] O’Neill FG, McKay S, Ward J, Strickland A, Kynoch R, Zuur A. An investigation of the relationship between sea state induced vessel motion and cod-end selection. Fisheries Research. 2003;60(1):107–130.

[23] Hurvich CM, Tsai CL. Regression and time series model selection in small samples. Biometrika. 1989; pp. 297–307.

[24] Herrmann B, Wienbeck H, Moderhak W, Stepputtis D, Krag LA. The influence of twine thickness, twine number and netting orientation on codend selectivity. Fisheries Research. 2013;145:22–36.

[25] O’Neill F, Kynoch R. The effect of cover mesh size and cod-end catch size on cod-end selectivity. Fisheries Research. 1996;28(3):291–303.

[26] Anom. Report of the Study Group on Turned 90 Codend Selectivity,focusing on Baltic Cod Selectivity (SGTCOD). IMR, Reykjavik, Iceland: ICES; 2011. CM 2011/SSGESST:08.

[27] Madsen N. Selectivity of fishing gears used in the Baltic Sea cod fishery. Reviews in Fish Biology and Fisheries. 2007;17(4):517–544.

