# Supplementary material for: Predictive framework for codend size selection of brown shrimp (Crangon crangon) in the North Sea beam-trawl fishery
Source: PLoS One. 2018 Jul 16;13(7):e0200464. doi: 10.1371/journal.pone.0200464 (PMC6047787; doi:10.1371/journal.pone.0200464)
Supplement: S1 Table — Geographical coordinates (decimal degrees) refer to the start and end of each haul. Operational information is completed with towing direction (°), distance towed (in nautical miles, nm), and the average fishing depth in meters (m) (n.a. = not available). Hauls ordered by codend type, mesh size, and cruise. (DOCX) [file pone.0200464.s001.docx]

**S1 Table. Operational information of the test hauls.** Geographical coordinates (decimal degrees) refer to the start and end of each haul. Operational information is completed with towing direction (°), distance towed (in nautical miles, nm), and the average fishing depth in meters (m) (n.a. = not available). Hauls ordered by codend type, mesh size, and cruise.

| Cruise | Station | Codend type | Mesh size | Latitude start | Longitude start | Latitude end | Longitude end | Direction | Distance | Depth |
| --- | --- | --- | --- | --- | --- | --- | --- | --- | --- | --- |
| January | 1 | Diamond-mesh | 19.05 | 54.728 | 8.132 | 54.775 | 8.153 | 25 | 2.9 | 11 |
| January | 2 | Diamond-mesh | 19.05 | 54.776 | 8.141 | 54.727 | 8.143 | 165 | 2.9 | 12 |
| January | 3 | Diamond-mesh | 19.05 | 54.735 | 8.131 | 54.781 | 8.138 | 25 | 2.8 | 11 |
| November | 4 | Diamond-mesh | 19.05 | 54.572 | 8.053 | 54.602 | 8.126 | 60 | 3.1 | 14 |
| November | 5 | Diamond-mesh | 19.05 | 54.607 | 8.151 | 54.559 | 8.126 | 180 | 3 | 14 |
| November | 6 | Diamond-mesh | 19.05 | 54.566 | 8.116 | 54.613 | 8.148 | 15 | 3.1 | 14 |
| January | 7 | Diamond-mesh | 20.188 | 54.237 | 8.299 | 54.275 | 8.351 | 39 | 2.9 | 13 |
| January | 8 | Diamond-mesh | 20.188 | 54.295 | 8.368 | 54.338 | 8.332 | 330 | 2.9 | 12 |
| January | 9 | Diamond-mesh | 20.188 | 54.672 | 8.143 | 54.722 | 8.135 | 360 | 3 | 13 |
| April | 10 | Diamond-mesh | 20.188 | 54.685 | 8.14 | 54.638 | 8.144 | 160 | 2.8 | 14 |
| April | 11 | Diamond-mesh | 20.188 | 54.632 | 8.144 | 54.587 | 8.161 | 160 | 2.8 | 14 |
| April | 12 | Diamond-mesh | 20.188 | 54.523 | 8.055 | 54.505 | 7.983 | 240 | 2.8 | 18 |
| April | 13 | Diamond-mesh | 20.188 | 54.519 | 7.978 | 54.48 | 8.029 | 130 | 2.9 | 17 |
| April | 14 | Diamond-mesh | 20.188 | 54.42 | 8.072 | 54.396 | 8.128 | 130 | 2.4 | 19 |
| April | 15 | Diamond-mesh | 21.45 | 54.715 | 8.133 | 54.762 | 8.137 | 10 | 2.8 | 13 |
| April | 16 | Diamond-mesh | 21.45 | 54.77 | 8.158 | 54.724 | 8.173 | 150 | 2.8 | 13 |
| April | 17 | Diamond-mesh | 21.45 | 54.724 | 8.141 | 54.676 | 8.135 | 175 | 2.9 | 13 |
| April | 18 | Diamond-mesh | 21.45 | 54.69 | 8.177 | 54.738 | 8.171 | 5 | 2.9 | 13 |
| April | 19 | Diamond-mesh | 21.45 | 54.717 | 8.141 | 54.764 | 8.14 | 350 | 2.8 | 12 |
| September | 20 | Diamond-mesh | 21.45 | 54.495 | 8.063 | 54.516 | 7.984 | 315 | 3 | n.a. |
| September | 21 | Diamond-mesh | 21.45 | 54.512 | 7.975 | 54.497 | 8.06 | 115 | 3.1 | n.a. |
| September | 22 | Diamond-mesh | 21.45 | 54.491 | 8.069 | 54.484 | 8.159 | 95 | 3.2 | n.a. |
| January | 23 | Diamond-mesh | 22.95 | 54.74 | 8.136 | 54.691 | 8.12 | 175 | 3 | 13 |
| January | 24 | Diamond-mesh | 22.95 | 54.689 | 8.118 | 54.722 | 8.177 | 40 | 2.9 | 12 |
| January | 25 | Diamond-mesh | 22.95 | 54.732 | 8.17 | 54.778 | 8.165 | 360 | 2.7 | 11 |
| November | 26 | Diamond-mesh | 22.95 | 54.622 | 8.106 | 54.669 | 8.135 | 40 | 3 | 14 |
| November | 27 | Diamond-mesh | 22.95 | 54.67 | 8.138 | 54.623 | 8.096 | 200 | 3.2 | 14 |
| November | 28 | Diamond-mesh | 22.95 | 54.581 | 8.108 | 54.631 | 8.112 | 350 | 3 | 16 |
| April | 29 | Diamond-mesh | 24.65 | 54.592 | 8.093 | 54.639 | 8.119 | 15 | 2.9 | 14 |
| April | 30 | Diamond-mesh | 24.65 | 54.637 | 8.113 | 54.589 | 8.1 | 200 | 2.9 | 14 |
| April | 31 | Diamond-mesh | 24.65 | 54.587 | 8.103 | 54.637 | 8.109 | 360 | 3.1 | 15 |
| April | 32 | Diamond-mesh | 24.65 | 54.66 | 8.104 | 54.612 | 8.097 | 190 | 2.9 | 16 |
| April | 33 | Diamond-mesh | 24.65 | 54.587 | 8.102 | 54.637 | 8.113 | 15 | 3.1 | 15 |
| April | 34 | Diamond-mesh | 24.65 | 54.612 | 8.106 | 54.657 | 8.139 | 35 | 3 | 15 |
| January | 35 | Diamond-mesh | 25.1 | 54.821 | 8.142 | 54.774 | 8.158 | 163 | 2.9 | 11 |
| January | 36 | Diamond-mesh | 25.1 | 54.771 | 8.158 | 54.732 | 8.195 | 165 | 2.6 | 13 |
| January | 37 | Diamond-mesh | 25.1 | 54.788 | 8.143 | 54.739 | 8.137 | 175 | 2.9 | 13 |
| April | 38 | Diamond-mesh | 25.1 | 54.667 | 8.134 | 54.719 | 8.137 | 20 | 3.1 | 14 |
| April | 39 | Diamond-mesh | 25.1 | 54.652 | 8.097 | 54.604 | 8.102 | 170 | 2.9 | 15 |
| September | 40 | Diamond-mesh | 27.15 | 54.588 | 8.09 | 54.639 | 8.099 | 30 | 3.1 | n.a. |
| September | 41 | Diamond-mesh | 27.15 | 54.642 | 8.094 | 54.589 | 8.1 | 170 | 3.2 | n.a. |
| September | 42 | Diamond-mesh | 27.15 | 54.579 | 8.095 | 54.63 | 8.112 | 15 | 3.1 | n.a. |
| September | 43 | Diamond-mesh | 27.15 | 54.628 | 8.116 | 54.578 | 8.102 | 200 | 3 | n.a. |
| September | 44 | Diamond-mesh | 27.15 | 54.637 | 8.095 | 54.586 | 8.101 | 180 | 3.1 | n.a. |
| September | 45 | Diamond-mesh | 27.15 | 54.587 | 8.099 | 54.639 | 8.1 | 350 | 3.2 | n.a. |
| April | 46 | Diamond-mesh | 27.825 | 54.685 | 8.137 | 54.735 | 8.134 | 355 | 3 | 13 |
| April | 47 | Diamond-mesh | 27.825 | 54.668 | 8.135 | 54.624 | 8.111 | 180 | 2.8 | 15 |
| April | 48 | Diamond-mesh | 27.825 | 54.643 | 8.118 | 54.593 | 8.102 | 180 | 3 | 14 |
| April | 49 | Diamond-mesh | 27.825 | 54.599 | 8.101 | 54.649 | 8.096 | 5 | 3 | 15 |
| April | 50 | Diamond-mesh | 27.825 | 54.648 | 8.094 | 54.599 | 8.1 | 170 | 2.9 | 14 |
| April | 51 | Diamond-mesh | 27.825 | 54.599 | 8.097 | 54.648 | 8.099 | 5 | 2.9 | 14 |
| April | 52 | Diamond-mesh | 27.825 | 54.533 | 8.12 | 54.578 | 8.101 | 345 | 2.8 | 16 |
| April | 53 | Diamond-mesh | 27.825 | 54.596 | 8.098 | 54.645 | 8.094 | 350 | 2.9 | 16 |
| September | 54 | Diamond-mesh | 29.35 | 54.582 | 8.157 | 54.53 | 8.171 | 160 | 3.1 | n.a. |
| September | 55 | Diamond-mesh | 29.35 | 54.546 | 8.168 | 54.493 | 8.178 | 160 | 3.2 | n.a. |
| September | 56 | Diamond-mesh | 29.35 | 54.495 | 8.077 | 54.505 | 7.993 | 290 | 3 | n.a. |
| November | 57 | Diamond-mesh | 29.35 | 54.632 | 8.104 | 54.582 | 8.102 | 180 | 3 | 16 |
| November | 58 | Diamond-mesh | 29.35 | 54.592 | 8.106 | 54.634 | 8.148 | 15 | 2.9 | 14 |
| September | 59 | Diamond-mesh | 31.575 | 54.743 | 8.136 | 54.69 | 8.136 | 170 | 3.2 | n.a. |
| September | 60 | Diamond-mesh | 31.575 | 54.654 | 8.099 | 54.601 | 8.098 | 180 | 3.2 | n.a. |
| September | 61 | Diamond-mesh | 31.575 | 54.587 | 8.096 | 54.638 | 8.098 | 1 | 3.1 | n.a. |
| September | 62 | Diamond-mesh | 31.575 | 54.644 | 8.102 | 54.592 | 8.099 | 185 | 3.1 | n.a. |
| September | 63 | Diamond-mesh | 31.575 | 54.501 | 8.01 | 54.482 | 8.094 | 115 | 3.2 | n.a. |
| September | 64 | Diamond-mesh | 31.575 | 54.799 | 8.139 | 54.748 | 8.136 | 175 | 3 | n.a. |
| April | 65 | Diamond-mesh | 32.25 | 54.644 | 8.101 | 54.594 | 8.1 | 200 | 3 | 14 |
| April | 66 | Diamond-mesh | 32.25 | 54.592 | 8.106 | 54.642 | 8.118 | 350 | 3 | 16 |
| April | 67 | Diamond-mesh | 32.25 | 54.64 | 8.119 | 54.59 | 8.101 | 200 | 3.1 | 15 |
| April | 68 | Diamond-mesh | 32.25 | 54.584 | 8.101 | 54.636 | 8.099 | 350 | 3.1 | 16 |
| April | 69 | Diamond-mesh | 32.25 | 54.591 | 8.101 | 54.64 | 8.116 | 15 | 3 | 16 |
| April | 70 | Diamond-mesh | 32.25 | 54.641 | 8.116 | 54.594 | 8.1 | 190 | 2.9 | 15 |
| April | 71 | Diamond-mesh | 32.275 | 54.59 | 8.104 | 54.637 | 8.098 | 350 | 2.8 | 14 |
| April | 72 | Diamond-mesh | 32.275 | 54.64 | 8.095 | 54.687 | 8.124 | 10 | 3 | 14 |
| April | 73 | Diamond-mesh | 32.275 | 54.686 | 8.123 | 54.638 | 8.097 | 190 | 3 | 16 |
| April | 74 | Diamond-mesh | 32.275 | 54.633 | 8.096 | 54.585 | 8.1 | 170 | 2.9 | 15 |
| April | 75 | Diamond-mesh | 32.275 | 54.591 | 8.094 | 54.64 | 8.115 | 20 | 3 | 16 |
| April | 76 | Diamond-mesh | 32.275 | 54.644 | 8.111 | 54.598 | 8.1 | 180 | 2.8 | 13 |
| April | 77 | Diamond-mesh | 32.275 | 54.792 | 8.139 | 54.743 | 8.137 | 165 | 3 | 12 |
| April | 78 | Diamond-mesh | 32.275 | 54.741 | 8.133 | 54.79 | 8.144 | 20 | 2.9 | 11 |
| April | 79 | Diamond-mesh | 32.275 | 54.791 | 8.148 | 54.741 | 8.143 | 170 | 3 | 13 |
| April | 80 | Diamond-mesh | 32.275 | 54.738 | 8.139 | 54.787 | 8.149 | 10 | 3 | 13 |
| April | 81 | Diamond-mesh | 32.275 | 54.788 | 8.142 | 54.741 | 8.138 | 185 | 2.8 | 14 |
| April | 82 | Diamond-mesh | 32.275 | 54.743 | 8.139 | 54.792 | 8.128 | 360 | 3 | 13 |
| September | 83 | Diamond-mesh | 36.375 | 54.362 | 8.058 | 54.311 | 8.075 | 145 | 3.1 | n.a. |
| September | 84 | Diamond-mesh | 36.375 | 54.31 | 8.078 | 54.361 | 8.067 | 5 | 3.1 | n.a. |
| September | 85 | Diamond-mesh | 36.375 | 54.361 | 8.061 | 54.308 | 8.075 | 160 | 3.2 | n.a. |
| September | 86 | Diamond-mesh | 36.375 | 54.308 | 8.099 | 54.28 | 8.175 | 110 | 3.2 | n.a. |
| September | 87 | Diamond-mesh | 36.375 | 54.49 | 8.081 | 54.483 | 8.169 | 65 | 3.1 | n.a. |
| January | 88 | Square-mesh | 17.25 | 54.806 | 8.16 | 54.758 | 8.171 | 175 | 2.9 | 12 |
| January | 89 | Square-mesh | 17.25 | 54.758 | 8.151 | 54.711 | 8.136 | 185 | 2.9 | 13 |
| January | 90 | Square-mesh | 18.75 | 54.7 | 8.174 | 54.664 | 8.141 | 220 | 2.5 | 14 |
| January | 91 | Square-mesh | 18.75 | 54.704 | 8.178 | 54.754 | 8.168 | 350 | 3 | 12 |
| November | 92 | Square-mesh | 18.75 | 54.615 | 8.157 | 54.647 | 8.094 | 326 | 2.9 | 16 |
| November | 93 | Square-mesh | 18.75 | 54.66 | 8.103 | 54.61 | 8.098 | 175 | 3.1 | 14 |
| November | 94 | Square-mesh | 18.75 | 54.608 | 8.103 | 54.656 | 8.132 | 30 | 3 | 14 |
| April | 95 | Square-mesh | 20.975 | 55.279 | 8.286 | 55.328 | 8.289 | 350 | 2.9 | 14 |
| April | 96 | Square-mesh | 20.975 | 54.647 | 8.096 | 54.597 | 8.1 | 170 | 3 | 13 |
| April | 97 | Square-mesh | 20.975 | 54.598 | 8.1 | 54.647 | 8.097 | 360 | 3 | 15 |
| April | 98 | Square-mesh | 20.975 | 54.646 | 8.098 | 54.597 | 8.101 | 175 | 3 | 13 |
| April | 99 | Square-mesh | 20.975 | 54.596 | 8.104 | 54.643 | 8.096 | 340 | 2.8 | 16 |
| April | 100 | Square-mesh | 20.975 | 54.641 | 8.099 | 54.593 | 8.101 | 185 | 2.9 | 15 |
| September | 101 | Square-mesh | 20.975 | 54.651 | 8.097 | 54.597 | 8.096 | 180 | 3.3 | n.a. |
| September | 102 | Square-mesh | 20.975 | 54.574 | 8.103 | 54.628 | 8.105 | 360 | 3.3 | n.a. |
| September | 103 | Square-mesh | 20.975 | 54.629 | 8.116 | 54.679 | 8.115 | 360 | 3 | n.a. |
| September | 104 | Square-mesh | 20.975 | 54.708 | 8.136 | 54.655 | 8.139 | 175 | 3.2 | n.a. |
| November | 105 | Square-mesh | 20.975 | 54.616 | 8.151 | 54.667 | 8.135 | 360 | 3.1 | 13 |
| November | 106 | Square-mesh | 20.975 | 54.669 | 8.135 | 54.719 | 8.137 | 360 | 3 | 13 |
| September | 107 | Square-mesh | 23.4 | 54.657 | 8.146 | 54.709 | 8.137 | 340 | 3.1 | n.a. |
| September | 108 | Square-mesh | 23.4 | 54.669 | 8.112 | 54.619 | 8.096 | 185 | 3.1 | n.a. |
| September | 109 | Square-mesh | 23.4 | 54.572 | 8.098 | 54.624 | 8.104 | 360 | 3.1 | n.a. |
| September | 110 | Square-mesh | 23.4 | 54.628 | 8.108 | 54.573 | 8.104 | 180 | 3.3 | n.a. |
| November | 111 | Square-mesh | 23.4 | 54.704 | 8.147 | 54.653 | 8.139 | 180 | 3.1 | 15 |
| November | 112 | Square-mesh | 23.4 | 54.638 | 8.146 | 54.588 | 8.16 | 172 | 3.1 | 15 |
| April | 113 | Square-mesh | 24.95 | 54.646 | 8.099 | 54.596 | 8.101 | 185 | 3 | 13 |
| April | 114 | Square-mesh | 24.95 | 54.586 | 8.107 | 54.633 | 8.094 | 335 | 2.8 | 16 |
| September | 115 | Square-mesh | 24.95 | 54.593 | 8.106 | 54.636 | 8.146 | 350 | 3 | n.a. |
| September | 116 | Square-mesh | 24.95 | 54.658 | 8.102 | 54.608 | 8.094 | 190 | 3 | n.a. |
| September | 117 | Square-mesh | 24.95 | 54.609 | 8.086 | 54.558 | 8.109 | 170 | 3.1 | n.a. |
| September | 118 | Square-mesh | 24.95 | 54.566 | 8.108 | 54.609 | 8.058 | 335 | 3.1 | n.a. |
| November | 119 | Square-mesh | 24.95 | 54.59 | 8.163 | 54.641 | 8.144 | 340 | 3.1 | 15 |
| November | 120 | Square-mesh | 24.95 | 54.646 | 8.134 | 54.699 | 8.138 | 360 | 3.2 | 15 |
| April | 121 | Square-mesh | 25.2 | 54.594 | 8.108 | 54.643 | 8.095 | 345 | 3 | 17 |
| April | 122 | Square-mesh | 25.2 | 54.643 | 8.099 | 54.597 | 8.099 | 185 | 2.8 | 16 |
| April | 123 | Square-mesh | 25.2 | 54.647 | 8.095 | 54.599 | 8.097 | 170 | 2.9 | 14 |
| April | 124 | Square-mesh | 25.2 | 54.604 | 8.098 | 54.652 | 8.098 | 7 | 2.9 | 15 |
| April | 125 | Square-mesh | 25.2 | 54.65 | 8.095 | 54.599 | 8.096 | 175 | 3 | 14 |
| April | 126 | Square-mesh | 25.2 | 54.598 | 8.101 | 54.649 | 8.097 | 360 | 3 | 14 |
| September | 127 | Square-mesh | 27.775 | 54.574 | 8.099 | 54.615 | 8.05 | 330 | 3 | n.a. |
| September | 128 | Square-mesh | 27.775 | 54.616 | 8.043 | 54.576 | 8.1 | 150 | 3.1 | n.a. |
| September | 129 | Square-mesh | 27.775 | 54.567 | 8.107 | 54.621 | 8.098 | 345 | 3.3 | n.a. |
| September | 130 | Square-mesh | 27.775 | 54.657 | 8.101 | 54.606 | 8.098 | 193 | 3.1 | n.a. |
| November | 131 | Square-mesh | 27.775 | 54.625 | 8.145 | 54.676 | 8.138 | 12 | 3.1 | 13 |
| November | 132 | Square-mesh | 27.775 | 54.678 | 8.14 | 54.721 | 8.176 | 20 | 2.9 | 13 |
| September | 133 | Square-mesh | 29.275 | 54.576 | 8.099 | 54.618 | 8.045 | 315 | 3.1 | n.a. |
| September | 134 | Square-mesh | 29.275 | 54.621 | 8.045 | 54.579 | 8.093 | 150 | 3 | n.a. |
| September | 135 | Square-mesh | 29.275 | 54.577 | 8.162 | 54.525 | 8.171 | 165 | 3.1 | n.a. |
| September | 136 | Square-mesh | 29.275 | 54.511 | 8.174 | 54.566 | 8.167 | 359 | 3.3 | n.a. |
| November | 137 | Square-mesh | 29.275 | 54.708 | 8.14 | 54.66 | 8.102 | 195 | 3.2 | 15 |
| November | 138 | Square-mesh | 29.275 | 54.639 | 8.097 | 54.588 | 8.101 | 180 | 3.1 | 15 |
| January | 139 | T90 | 18.875 | 54.663 | 8.136 | 54.708 | 8.158 | 25 | 2.8 | 13 |
| January | 140 | T90 | 18.875 | 54.71 | 8.16 | 54.755 | 8.168 | 22 | 2.7 | 11 |
| January | 141 | T90 | 18.875 | 54.808 | 8.159 | 54.759 | 8.169 | 170 | 2.9 | 12 |
| November | 142 | T90 | 18.875 | 54.662 | 8.134 | 54.611 | 8.153 | 170 | 3.2 | 14 |
| November | 143 | T90 | 18.875 | 54.618 | 8.146 | 54.665 | 8.137 | 5 | 2.9 | 13 |
| January | 144 | T90 | 20.175 | 54.734 | 8.166 | 54.782 | 8.155 | 350 | 2.9 | 12 |
| January | 145 | T90 | 20.175 | 54.784 | 8.146 | 54.737 | 8.146 | 180 | 2.8 | 14 |
| January | 146 | T90 | 20.175 | 54.733 | 8.132 | 54.684 | 8.136 | 170 | 3 | 13 |
| April | 147 | T90 | 21.15 | 54.287 | 7.856 | 54.317 | 7.804 | 335 | 2.6 | 24 |
| April | 148 | T90 | 21.15 | 54.4 | 7.965 | 54.415 | 8.049 | 60 | 3.1 | 18 |
| April | 149 | T90 | 21.15 | 54.582 | 8.097 | 54.633 | 8.096 | 360 | 3.1 | 16 |
| April | 150 | T90 | 21.15 | 54.646 | 8.1 | 54.599 | 8.101 | 175 | 2.8 | 15 |
| April | 151 | T90 | 21.15 | 54.602 | 8.1 | 54.654 | 8.097 | 15 | 3.1 | 15 |
| April | 152 | T90 | 21.15 | 54.657 | 8.099 | 54.609 | 8.095 | 170 | 2.9 | 15 |
| January | 153 | T90 | 22.5 | 54.736 | 8.161 | 54.785 | 8.163 | 12 | 2.9 | 11 |
| January | 154 | T90 | 22.5 | 54.79 | 8.143 | 54.837 | 8.131 | -9 | 2.8 | 13 |
| January | 155 | T90 | 22.5 | 54.781 | 8.164 | 54.732 | 8.169 | 170 | 2.9 | 13 |
| January | 156 | T90 | 22.5 | 53.792 | 7.424 | 53.803 | 7.498 | 80 | 2.7 | 19 |
| January | 157 | T90 | 22.5 | 53.819 | 7.521 | 53.803 | 7.595 | 100 | 2.8 | 12 |
| November | 158 | T90 | 22.5 | 54.585 | 8.163 | 54.633 | 8.149 | 336 | 2.9 | 13 |
| April | 159 | T90 | 24.35 | 54.587 | 8.102 | 54.635 | 8.096 | 355 | 2.9 | 15 |
| April | 160 | T90 | 24.35 | 54.641 | 8.092 | 54.687 | 8.122 | 23 | 3 | 13 |
| April | 161 | T90 | 24.35 | 54.632 | 8.146 | 54.681 | 8.137 | 350 | 2.9 | 14 |
| April | 162 | T90 | 24.35 | 54.638 | 8.1 | 54.589 | 8.1 | 200 | 3 | 15 |
| April | 163 | T90 | 24.35 | 54.59 | 8.1 | 54.638 | 8.094 | 345 | 2.9 | 17 |
| April | 164 | T90 | 24.35 | 54.636 | 8.094 | 54.589 | 8.099 | 180 | 2.8 | 16 |
| April | 165 | T90 | 24.35 | 54.594 | 8.099 | 54.641 | 8.089 | 350 | 2.9 | 17 |
| September | 166 | T90 | 24.625 | 54.276 | 8.096 | 54.326 | 8.071 | 360 | 3.1 | n.a. |
| September | 167 | T90 | 24.625 | 54.337 | 8.07 | 54.287 | 8.088 | 170 | 3.1 | n.a. |
| September | 168 | T90 | 24.625 | 54.276 | 8.111 | 54.233 | 8.154 | 170 | 3 | n.a. |
| November | 169 | T90 | 24.625 | 54.633 | 8.148 | 54.583 | 8.161 | 180 | 3 | 13 |
| November | 170 | T90 | 24.625 | 54.583 | 8.167 | 54.613 | 8.099 | 300 | 3 | 14 |
| September | 171 | T90 | 27.55 | 54.596 | 8.096 | 54.647 | 8.096 | 360 | 3.1 | n.a. |
| September | 172 | T90 | 27.55 | 54.652 | 8.094 | 54.6 | 8.104 | 170 | 3.1 | n.a. |
| September | 173 | T90 | 27.55 | 54.572 | 8.08 | 54.619 | 8.1 | 5 | 2.9 | n.a. |
| September | 174 | T90 | 27.55 | 54.643 | 8.101 | 54.592 | 8.102 | 175 | 3 | n.a. |
| November | 175 | T90 | 27.55 | 54.624 | 8.107 | 54.673 | 8.14 | 358 | 3.2 | 14 |
| November | 176 | T90 | 27.55 | 54.681 | 8.148 | 54.631 | 8.149 | 185 | 3 | 12 |
| April | 177 | T90 | 27.825 | 54.584 | 8.095 | 54.632 | 8.096 | 15 | 2.9 | 14 |
| April | 178 | T90 | 27.825 | 54.631 | 8.091 | 54.581 | 8.1 | 160 | 3 | 14 |
| April | 179 | T90 | 27.825 | 54.58 | 8.099 | 54.617 | 8.153 | 15 | 2.9 | 12 |
| April | 180 | T90 | 27.825 | 54.62 | 8.156 | 54.65 | 8.097 | 340 | 2.7 | 15 |
| April | 181 | T90 | 27.825 | 54.647 | 8.096 | 54.597 | 8.099 | 180 | 3 | 15 |
| April | 182 | T90 | 27.825 | 54.638 | 8.097 | 54.686 | 8.122 | 20 | 3 | 15 |
| September | 183 | T90 | 29.025 | 54.579 | 8.165 | 54.63 | 8.15 | 345 | 3.1 | n.a. |
| September | 184 | T90 | 29.025 | 54.59 | 8.139 | 54.624 | 8.102 | 305 | 2.4 | n.a. |
| September | 185 | T90 | 29.025 | 54.645 | 8.099 | 54.595 | 8.099 | 185 | 3 | n.a. |
| September | 186 | T90 | 29.025 | 54.586 | 8.147 | 54.543 | 8.169 | 150 | 2.7 | n.a. |
| November | 187 | T90 | 29.025 | 54.633 | 8.143 | 54.58 | 8.162 | 162 | 3.2 | 12 |
| November | 188 | T90 | 29.025 | 54.581 | 8.166 | 54.537 | 8.17 | 265 | 2.6 | 12 |
| April | 189 | T90 | 31.275 | 54.651 | 8.091 | 54.601 | 8.092 | 170 | 3 | 14 |
| April | 190 | T90 | 31.275 | 54.639 | 8.1 | 54.589 | 8.1 | 175 | 3 | 13 |
| April | 191 | T90 | 31.275 | 54.59 | 8.1 | 54.639 | 8.101 | 360 | 2.9 | 14 |
| April | 192 | T90 | 31.275 | 54.633 | 8.109 | 54.585 | 8.1 | 190 | 2.9 | 15 |
| April | 193 | T90 | 31.275 | 54.587 | 8.103 | 54.636 | 8.095 | 345 | 3 | 16 |
| April | 194 | T90 | 31.275 | 54.738 | 8.135 | 54.788 | 8.14 | 360 | 3 | 14 |
| September | 195 | T90 | 31.4 | 54.585 | 8.106 | 54.639 | 8.096 | 350 | 3.2 | n.a. |
| September | 196 | T90 | 31.4 | 54.648 | 8.092 | 54.596 | 8.1 | 175 | 3.1 | n.a. |
| September | 197 | T90 | 31.4 | 54.602 | 8.102 | 54.653 | 8.096 | 20 | 3.1 | n.a. |
| September | 198 | T90 | 31.4 | 54.669 | 8.101 | 54.613 | 8.101 | 170 | 3.4 | n.a. |
| November | 199 | T90 | 31.4 | 54.374 | 8.182 | 54.429 | 8.191 | 345 | 3.3 | 18 |
| September | 200 | T90 | 36.5 | 54.59 | 8.163 | 54.644 | 8.142 | 335 | 3.3 | n.a. |
| September | 201 | T90 | 36.5 | 54.648 | 8.135 | 54.607 | 8.099 | 220 | 2.8 | n.a. |
| September | 202 | T90 | 36.5 | 54.586 | 8.101 | 54.64 | 8.097 | 358 | 3.2 | n.a. |
| September | 203 | T90 | 36.5 | 54.641 | 8.095 | 54.589 | 8.1 | 175 | 3.2 | n.a. |
| September | 204 | T90 | 36.5 | 54.588 | 8.101 | 54.639 | 8.105 | 11 | 3.1 | n.a. |
| November | 205 | T90 | 36.5 | 54.539 | 8.174 | 54.59 | 8.161 | 330 | 3.1 | 16 |
