# Supplementary material for: Predictive framework for codend size selection of brown shrimp (Crangon crangon) in the North Sea beam-trawl fishery
Source: PLoS One. 2018 Jul 16;13(7):e0200464. doi: 10.1371/journal.pone.0200464 (PMC6047787; doi:10.1371/journal.pone.0200464)
Supplement: S2 Table — Catch weight, sea state and trawl side are the fixed factors measured at haul level, and included together with mesh size in the meta-analysis. Standard deviation of the estimated L50, SR, and SP are shown in brackets. CO11 to CO33 are the vectorised form of the covariance matrix from the estimated L50, SR, and SP. Hauls ordered by codend type, mesh size, and cruise. (DOCX) [file pone.0200464.s002.docx]

**S2 Table. Results from the selectivity analysis for individual hauls.** Catch weight, sea state and trawl side are the fixed factors measured at haul level, and included together with mesh size in the meta-analysis. Standard deviation of the estimated *L50*, *SR*, and *SP* are shown in brackets. CO11 to CO33 are the vectorised form of the covariance matrix from the estimated *L50*, *SR,* and *SP*. Hauls ordered by codend type, mesh size, and cruise.

| **Cruise** | **Haul** | **Codend type** | **Mesh size (m)** | **Cath weight (w)** | **Sea State (s)** | **Trawl side (p)** | **L50** | **SR** | **SP** | **CO11** | **CO12** | **CO13** | **CO22** | **CO23** | **CO33** | **P-Value** | **Deviance** | **d.o.f** |
| --- | --- | --- | --- | --- | --- | --- | --- | --- | --- | --- | --- | --- | --- | --- | --- | --- | --- | --- |
| January | 1 | Diamond-mesh | 19.05 | 16.88 | 5 | 1 | 33.69 (0.88) | 7.36 (1.32) | 0.44 (0.01) | 0.78 | 0.43 | 0.01 | 1.73 | 0.01 | 0.00 | 0.344 | 118.44 | 113 |
| January | 2 | Diamond-mesh | 19.05 | 21.45 | 4 | 1 | 29.34 (1.36) | 6.01 (1.7) | 0.57 (0.01) | 1.86 | 0.09 | 0.00 | 2.89 | 0.00 | 0.00 | 0.021 | 136.64 | 105 |
| January | 3 | Diamond-mesh | 19.05 | 20.26 | 4 | 1 | 30.55 (2.43) | 15.76 (5.05) | 0.49 (0.02) | 5.89 | 0.16 | 0.02 | 25.52 | 0.07 | 0.00 | 0.158 | 131.26 | 116 |
| November | 4 | Diamond-mesh | 19.05 | 92.9 | 6 | 0 | 39.43 (2.02) | 9.52 (3.09) | 0.56 (0.02) | 4.07 | -0.51 | 0.01 | 9.54 | 0.02 | 0.00 | 0.740 | 75.3 | 84 |
| November | 5 | Diamond-mesh | 19.05 | 101 | 6 | 0 | 44.89 (1.03) | 7.96 (1.68) | 0.45 (0.02) | 1.05 | 1.07 | 0.02 | 2.81 | 0.02 | 0.00 | 0.958 | 71.71 | 94 |
| November | 6 | Diamond-mesh | 19.05 | 74.62 | 5 | 0 | 44.62 (1.97) | 10.92 (3.08) | 0.56 (0.03) | 3.87 | 4.18 | 0.05 | 9.51 | 0.07 | 0.00 | 0.243 | 104.23 | 95 |
| January | 7 | Diamond-mesh | 20.19 | 12.07 | 4 | 1 | 28.43 (3.59) | 7.74 (6.96) | 0.45 (0.01) | 12.88 | -7.07 | 0.01 | 48.37 | 0.05 | 0.00 | 0.026 | 125.86 | 97 |
| January | 8 | Diamond-mesh | 20.19 | 18.2 | 5 | 1 | 34.9 (1.37) | 12.98 (1.95) | 0.5 (0.02) | 1.87 | 1.25 | 0.02 | 3.79 | 0.02 | 0.00 | 0.116 | 131.21 | 113 |
| January | 9 | Diamond-mesh | 20.19 | 19.48 | 4 | 1 | 31.97 (1.02) | 6.98 (1.28) | 0.5 (0.01) | 1.04 | 0.48 | 0.00 | 1.65 | 0.00 | 0.00 | 0.213 | 125.72 | 114 |
| April | 10 | Diamond-mesh | 20.19 | 9.93 | 3 | 0 | 40.63 (4.89) | 21.24 (9.34) | 0.55 (0.05) | 23.95 | 35.45 | 0.25 | 87.33 | 0.44 | 0.00 | 0.302 | 105.79 | 99 |
| April | 11 | Diamond-mesh | 20.19 | 12.01 | 3 | 0 | 39.62 (0.93) | 7.65 (1.38) | 0.49 (0.02) | 0.86 | 0.57 | 0.01 | 1.92 | 0.01 | 0.00 | 0.089 | 120.59 | 101 |
| April | 12 | Diamond-mesh | 20.19 | 9.07 | 2 | 0 | 36.1 (0.85) | 5.15 (1.33) | 0.48 (0.01) | 0.73 | 0.45 | 0.01 | 1.78 | 0.01 | 0.00 | 0.116 | 103.01 | 87 |
| April | 13 | Diamond-mesh | 20.19 | 5.06 | 2 | 1 | 42.06 (1.53) | 9.08 (2.65) | 0.54 (0.03) | 2.35 | 2.55 | 0.03 | 7.01 | 0.05 | 0.00 | 0.145 | 100.98 | 87 |
| April | 14 | Diamond-mesh | 20.19 | 9.52 | 3 | 1 | 32.94 (3.09) | 7.25 (6.68) | 0.5 (0.02) | 9.52 | -6.92 | 0.01 | 44.61 | 0.07 | 0.00 | 0.166 | 99.71 | 87 |
| April | 15 | Diamond-mesh | 21.45 | 5.32 | 3 | 1 | 38.61 (1.32) | 9.81 (1.92) | 0.39 (0.02) | 1.73 | 1.07 | 0.02 | 3.69 | 0.02 | 0.00 | 0.029 | 129.57 | 101 |
| April | 16 | Diamond-mesh | 21.45 | 6.37 | 3 | 1 | 38.01 (1.16) | 7.66 (1.72) | 0.43 (0.02) | 1.35 | 1.02 | 0.02 | 2.94 | 0.02 | 0.00 | 0.032 | 129.94 | 102 |
| April | 17 | Diamond-mesh | 21.45 | 18.03 | 3 | 0 | 40.65 (0.93) | 7.75 (1.35) | 0.49 (0.02) | 0.86 | 0.67 | 0.01 | 1.82 | 0.01 | 0.00 | 0.397 | 106.1 | 103 |
| April | 18 | Diamond-mesh | 21.45 | 1.48 | 3 | 0 | 49.75 (3.14) | 16.06 (2.89) | 0.51 (0.05) | 9.85 | 7.79 | 0.14 | 8.35 | 0.10 | 0.00 | 0.051 | 119.79 | 96 |
| April | 19 | Diamond-mesh | 21.45 | 1.68 | 2 | 0 | 43.32 (3.3) | 14.91 (4.18) | 0.54 (0.05) | 10.89 | 11.12 | 0.15 | 17.49 | 0.16 | 0.00 | 0.015 | 130.62 | 98 |
| September | 20 | Diamond-mesh | 21.45 | 25.81 | 3 | 0 | 46.51 (0.9) | 7.48 (1.08) | 0.56 (0.02) | 0.82 | 0.65 | 0.01 | 1.16 | 0.01 | 0.00 | 0.365 | 102.23 | 98 |
| September | 21 | Diamond-mesh | 21.45 | 20.56 | 3 | 1 | 39.1 (2.08) | 10.75 (3.86) | 0.49 (0.03) | 4.31 | 3.32 | 0.04 | 14.92 | 0.07 | 0.00 | 0.001 | 153.81 | 101 |
| September | 22 | Diamond-mesh | 21.45 | 53.39 | 3 | 1 | 41.48 (2.69) | 16.69 (4) | 0.59 (0.03) | 7.26 | 7.09 | 0.08 | 15.96 | 0.09 | 0.00 | 0.049 | 126.7 | 102 |
| January | 23 | Diamond-mesh | 22.95 | 16.22 | 4 | 1 | 34.38 (0.99) | 7.38 (1.69) | 0.49 (0.01) | 0.98 | 1.07 | 0.01 | 2.86 | 0.01 | 0.00 | 0.428 | 115.07 | 113 |
| January | 24 | Diamond-mesh | 22.95 | 25.32 | 4 | 1 | 35.34 (0.77) | 6.15 (1.05) | 0.47 (0.01) | 0.59 | 0.39 | 0.00 | 1.09 | 0.00 | 0.00 | 0.057 | 141.1 | 116 |
| January | 25 | Diamond-mesh | 22.95 | 11.48 | 4 | 1 | 40.78 (1.72) | 10.4 (2.36) | 0.47 (0.03) | 2.94 | 3.23 | 0.04 | 5.59 | 0.05 | 0.00 | 0.005 | 153.71 | 111 |
| November | 26 | Diamond-mesh | 22.95 | 52.71 | 5 | 0 | 43.83 (1.86) | 10.54 (4.07) | 0.52 (0.02) | 3.45 | 1.82 | 0.03 | 16.56 | 0.07 | 0.00 | 0.654 | 80.27 | 86 |
| November | 27 | Diamond-mesh | 22.95 | 88.15 | 5 | 0 | 43.35 (1.25) | 6.36 (2.01) | 0.47 (0.02) | 1.56 | 0.41 | 0.01 | 4.04 | 0.01 | 0.00 | 0.135 | 101.64 | 87 |
| November | 28 | Diamond-mesh | 22.95 | 70.9 | 2 | 0 | 47.03 (2.18) | 12 (3.85) | 0.47 (0.03) | 4.77 | 4.47 | 0.06 | 14.79 | 0.08 | 0.00 | 0.006 | 134.69 | 96 |
| April | 29 | Diamond-mesh | 24.65 | 5.22 | 4 | 1 | 44.98 (0.86) | 6.34 (1.15) | 0.45 (0.02) | 0.74 | 0.59 | 0.01 | 1.32 | 0.01 | 0.00 | 0.343 | 102.06 | 97 |
| April | 30 | Diamond-mesh | 24.65 | 4.79 | 3 | 1 | 42.5 (0.74) | 5.11 (0.96) | 0.48 (0.02) | 0.54 | 0.23 | 0.01 | 0.92 | 0.00 | 0.00 | 0.374 | 90.63 | 87 |
| April | 31 | Diamond-mesh | 24.65 | 5.45 | 3 | 1 | 48.74 (1.49) | 10.53 (1.7) | 0.45 (0.03) | 2.22 | 1.81 | 0.04 | 2.91 | 0.03 | 0.00 | 0.836 | 83.4 | 97 |
| April | 32 | Diamond-mesh | 24.65 | 5.19 | 3 | 0 | 40.1 (1.13) | 8.92 (1.95) | 0.42 (0.02) | 1.28 | 0.81 | 0.02 | 3.80 | 0.02 | 0.00 | 0.066 | 111.01 | 90 |
| April | 33 | Diamond-mesh | 24.65 | 8.87 | 3 | 0 | 49.63 (1.27) | 8.96 (1.42) | 0.5 (0.03) | 1.62 | 1.33 | 0.03 | 2.02 | 0.02 | 0.00 | 0.114 | 110.79 | 94 |
| April | 34 | Diamond-mesh | 24.65 | 7.52 | 3 | 0 | 44.28 (2.82) | 17.6 (4.97) | 0.55 (0.03) | 7.97 | 8.07 | 0.08 | 24.67 | 0.12 | 0.00 | 0.168 | 109.23 | 96 |
| January | 35 | Diamond-mesh | 25.1 | 12.46 | 3 | 1 | 38.35 (2.27) | 14.85 (4.69) | 0.49 (0.03) | 5.16 | 3.11 | 0.05 | 21.99 | 0.09 | 0.00 | 0.133 | 107.18 | 92 |
| January | 36 | Diamond-mesh | 25.1 | 16.59 | 3 | 1 | 30.67 (2.83) | 7.84 (3.33) | 0.45 (0.02) | 8.04 | -0.95 | 0.02 | 11.12 | 0.02 | 0.00 | 0.000 | 157.02 | 101 |
| January | 37 | Diamond-mesh | 25.1 | 15.64 | 3 | 1 | 35.72 (3.45) | 19.23 (6.93) | 0.43 (0.04) | 11.87 | 13.43 | 0.11 | 48.06 | 0.21 | 0.00 | 0.173 | 62.57 | 53 |
| April | 38 | Diamond-mesh | 25.1 | 4.94 | 3 | 0 | 47.48 (1.25) | 9.57 (1.08) | 0.63 (0.03) | 1.57 | 1.10 | 0.03 | 1.17 | 0.02 | 0.00 | 0.687 | 101.33 | 109 |
| April | 39 | Diamond-mesh | 25.1 | 8.98 | 3 | 1 | 47.5 (0.9) | 6.64 (1.18) | 0.42 (0.02) | 0.82 | 0.61 | 0.01 | 1.39 | 0.01 | 0.00 | 0.123 | 105.78 | 90 |
| September | 40 | Diamond-mesh | 27.15 | 30.31 | 2 | 1 | 50.2 (1.45) | 11.31 (1.32) | 0.3 (0.02) | 2.11 | 1.56 | 0.03 | 1.74 | 0.02 | 0.00 | 0.899 | 92.43 | 111 |
| September | 41 | Diamond-mesh | 27.15 | 26.16 | 2 | 1 | 50.76 (1.82) | 12.16 (1.62) | 0.36 (0.03) | 3.31 | 2.49 | 0.05 | 2.61 | 0.04 | 0.00 | 0.390 | 109.44 | 106 |
| September | 42 | Diamond-mesh | 27.15 | 26.92 | 2 | 1 | 50.11 (1.02) | 8.08 (1.01) | 0.47 (0.03) | 1.04 | 0.85 | 0.02 | 1.01 | 0.02 | 0.00 | 0.803 | 95.35 | 108 |
| September | 43 | Diamond-mesh | 27.15 | 19.84 | 2 | 0 | 59.41 (2.03) | 12.49 (1.15) | 0.51 (0.04) | 4.10 | 1.99 | 0.08 | 1.33 | 0.03 | 0.00 | 0.051 | 133.04 | 108 |
| September | 44 | Diamond-mesh | 27.15 | 21.88 | 2 | 0 | 60.8 (2) | 10.39 (0.68) | 0.78 (0.04) | 3.99 | 1.16 | 0.08 | 0.47 | 0.02 | 0.00 | 0.923 | 87.77 | 108 |
| September | 45 | Diamond-mesh | 27.15 | 28.12 | 2 | 0 | 57.32 (2.59) | 13.89 (1.57) | 0.59 (0.05) | 6.71 | 3.57 | 0.12 | 2.46 | 0.06 | 0.00 | 0.248 | 123.88 | 114 |
| April | 46 | Diamond-mesh | 27.83 | 8.94 | 2 | 1 | 53.36 (1.76) | 9.66 (1.05) | 0.52 (0.05) | 3.08 | 1.54 | 0.08 | 1.10 | 0.03 | 0.00 | 0.006 | 143.85 | 104 |
| April | 47 | Diamond-mesh | 27.83 | 9.98 | 2 | 1 | 52.76 (1.14) | 7.33 (0.81) | 0.55 (0.03) | 1.30 | 0.78 | 0.03 | 0.65 | 0.02 | 0.00 | 0.559 | 96.27 | 99 |
| April | 48 | Diamond-mesh | 27.83 | 7.72 | 2 | 1 | 52.57 (1.38) | 9.28 (1.15) | 0.47 (0.03) | 1.90 | 1.31 | 0.04 | 1.32 | 0.02 | 0.00 | 0.900 | 79.63 | 97 |
| April | 49 | Diamond-mesh | 27.83 | 10.66 | 2 | 0 | 45.5 (2.55) | 13.34 (3.2) | 0.53 (0.04) | 6.51 | 6.68 | 0.09 | 10.23 | 0.10 | 0.00 | 0.021 | 123.85 | 94 |
| April | 50 | Diamond-mesh | 27.83 | 14.62 | 2 | 0 | 43.1 (0.96) | 7.42 (1.25) | 0.46 (0.02) | 0.93 | 0.80 | 0.01 | 1.56 | 0.01 | 0.00 | 0.516 | 98.77 | 100 |
| April | 51 | Diamond-mesh | 27.83 | 15.6 | 3 | 0 | 43.43 (1.23) | 9.92 (1.8) | 0.46 (0.02) | 1.50 | 1.16 | 0.02 | 3.22 | 0.02 | 0.00 | 0.112 | 113.11 | 96 |
| April | 52 | Diamond-mesh | 27.83 | 7.38 | 4 | 1 | 50.88 (1.08) | 7.59 (1.06) | 0.84 (0.01) | 1.16 | 0.85 | 0.01 | 1.13 | 0.01 | 0.00 | 0.288 | 97 | 90 |
| April | 53 | Diamond-mesh | 27.83 | 10.8 | 1 | 1 | 50.14 (1.66) | 9.94 (2.06) | 0.5 (0.03) | 2.77 | 2.55 | 0.04 | 4.24 | 0.04 | 0.00 | 0.139 | 100.33 | 86 |
| September | 54 | Diamond-mesh | 29.35 | 80.36 | 4 | 0 | 57.24 (2.41) | 11.96 (1.26) | 0.69 (0.05) | 5.80 | 2.68 | 0.11 | 1.60 | 0.04 | 0.00 | 0.425 | 101.01 | 99 |
| September | 55 | Diamond-mesh | 29.35 | 64.34 | 4 | 0 | 42.97 (2.29) | 11.13 (2.99) | 0.48 (0.04) | 5.26 | 5.56 | 0.08 | 8.96 | 0.09 | 0.00 | 0.253 | 94.31 | 86 |
| September | 56 | Diamond-mesh | 29.35 | 9.73 | 4 | 1 | 56.23 (3.17) | 13.93 (2.33) | 0.51 (0.06) | 10.04 | 6.36 | 0.17 | 5.41 | 0.10 | 0.00 | 0.002 | 143.69 | 97 |
| November | 57 | Diamond-mesh | 29.35 | 41.18 | 2 | 1 | 54.88 (2.39) | 13.01 (2.47) | 0.49 (0.04) | 5.70 | 4.93 | 0.09 | 6.09 | 0.07 | 0.00 | 0.583 | 93.44 | 97 |
| November | 58 | Diamond-mesh | 29.35 | 73.53 | 3 | 0 | 52.62 (3.07) | 16.08 (3.05) | 0.58 (0.05) | 9.40 | 7.53 | 0.13 | 9.30 | 0.10 | 0.00 | 0.176 | 109.84 | 97 |
| September | 59 | Diamond-mesh | 31.58 | 32.47 | 3 | 0 | 78.48 (12.12) | 21.09 (2.25) | 0.85 (0.12) | 146.85 | 22.64 | 1.42 | 5.08 | 0.19 | 0.01 | 0.030 | 139.43 | 110 |
| September | 60 | Diamond-mesh | 31.58 | 24.01 | 3 | 0 | 60.29 (1.77) | 9.46 (0.69) | 0.51 (0.06) | 3.14 | 1.01 | 0.09 | 0.47 | 0.02 | 0.00 | 0.449 | 114.26 | 113 |
| September | 61 | Diamond-mesh | 31.58 | 17.75 | 4 | 0 | 60.63 (6.22) | 18.7 (2.75) | 0.47 (0.1) | 38.73 | 15.40 | 0.62 | 7.55 | 0.22 | 0.01 | 0.023 | 140.37 | 109 |
| September | 62 | Diamond-mesh | 31.58 | 12.51 | 4 | 1 | 51.76 (1.73) | 12.63 (1.3) | 0.38 (0.03) | 3.00 | 1.88 | 0.05 | 1.70 | 0.03 | 0.00 | 0.606 | 103.46 | 108 |
| September | 63 | Diamond-mesh | 31.58 | 16.64 | 3 | 1 | 63.98 (3.42) | 11.88 (1.09) | 0.57 (0.09) | 11.73 | 3.18 | 0.31 | 1.18 | 0.07 | 0.01 | 0.038 | 138.73 | 111 |
| September | 64 | Diamond-mesh | 31.58 | 19.45 | 3 | 1 | 50.8 (2.66) | 12.45 (2.42) | 0.18 (0.03) | 7.08 | 5.48 | 0.07 | 5.85 | 0.05 | 0.00 | 0.989 | 66.95 | 96 |
| April | 65 | Diamond-mesh | 32.25 | 6.66 | 4 | 0 | 52.32 (1.06) | 8.07 (0.84) | 0.5 (0.03) | 1.13 | 0.71 | 0.02 | 0.70 | 0.01 | 0.00 | 0.385 | 104.53 | 101 |
| April | 66 | Diamond-mesh | 32.25 | 7.59 | 4 | 0 | 52.3 (1.07) | 7.14 (0.95) | 0.42 (0.03) | 1.15 | 0.78 | 0.02 | 0.90 | 0.01 | 0.00 | 0.034 | 121.7 | 95 |
| April | 67 | Diamond-mesh | 32.25 | 6.69 | 4 | 0 | 57.07 (1.38) | 7.71 (0.9) | 0.46 (0.04) | 1.92 | 1.08 | 0.04 | 0.81 | 0.02 | 0.00 | 0.691 | 87.67 | 95 |
| April | 68 | Diamond-mesh | 32.25 | 5.94 | 4 | 1 | 57.02 (2.45) | 12.75 (1.57) | 0.54 (0.05) | 6.01 | 3.35 | 0.11 | 2.46 | 0.05 | 0.00 | 0.687 | 89.72 | 97 |
| April | 69 | Diamond-mesh | 32.25 | 4.17 | 4 | 1 | 50.95 (2) | 12.55 (2.24) | 0.38 (0.03) | 3.99 | 3.52 | 0.06 | 5.01 | 0.05 | 0.00 | 0.826 | 79.25 | 92 |
| April | 70 | Diamond-mesh | 32.25 | 5.27 | 4 | 1 | 71 (12.69) | 25.29 (5.3) | 0.64 (0.15) | 160.93 | 62.25 | 1.82 | 28.10 | 0.66 | 0.02 | 0.123 | 110.11 | 94 |
| April | 71 | Diamond-mesh | 32.28 | 5.79 | 2 | 1 | 56.17 (1.88) | 10.54 (1.4) | 0.55 (0.04) | 3.55 | 2.25 | 0.07 | 1.95 | 0.04 | 0.00 | 0.204 | 105.11 | 94 |
| April | 72 | Diamond-mesh | 32.28 | 8.04 | 2 | 1 | 57.29 (1.76) | 10.6 (1.03) | 0.53 (0.04) | 3.10 | 1.53 | 0.07 | 1.07 | 0.03 | 0.00 | 0.966 | 80.1 | 105 |
| April | 73 | Diamond-mesh | 32.28 | 9.21 | 2 | 0 | 59.15 (2.12) | 11.01 (1.1) | 0.57 (0.05) | 4.49 | 2.00 | 0.10 | 1.21 | 0.04 | 0.00 | 0.760 | 88.77 | 99 |
| April | 74 | Diamond-mesh | 32.28 | 7.82 | 2 | 0 | 52.84 (1.28) | 8.94 (1.25) | 0.41 (0.03) | 1.64 | 1.20 | 0.03 | 1.57 | 0.02 | 0.00 | 0.566 | 95.05 | 98 |
| April | 75 | Diamond-mesh | 32.28 | 5.91 | 2 | 0 | 46.67 (1.19) | 8.4 (1.8) | 0.41 (0.02) | 1.42 | 1.27 | 0.02 | 3.25 | 0.02 | 0.00 | 0.612 | 92.45 | 97 |
| April | 76 | Diamond-mesh | 32.28 | 5.45 | 2 | 1 | 52.19 (1.22) | 8.14 (1.13) | 0.44 (0.03) | 1.49 | 1.09 | 0.03 | 1.29 | 0.02 | 0.00 | 0.329 | 103.64 | 98 |
| April | 77 | Diamond-mesh | 32.28 | 5.47 | 2 | 0 | 50.94 (2.3) | 13.11 (2.12) | 0.46 (0.04) | 5.27 | 4.10 | 0.09 | 4.49 | 0.06 | 0.00 | 0.550 | 95.58 | 98 |
| April | 78 | Diamond-mesh | 32.28 | 4.86 | 3 | 0 | 58.65 (1.94) | 12.09 (1.19) | 0.54 (0.04) | 3.78 | 1.88 | 0.08 | 1.41 | 0.03 | 0.00 | 0.102 | 124.82 | 106 |
| April | 79 | Diamond-mesh | 32.28 | 5.41 | 3 | 0 | 55.6 (2.54) | 13.65 (1.65) | 0.58 (0.05) | 6.43 | 3.58 | 0.11 | 2.72 | 0.06 | 0.00 | 0.586 | 98.29 | 102 |
| April | 80 | Diamond-mesh | 32.28 | 4.22 | 3 | 1 | 55.02 (2.38) | 12.21 (1.73) | 0.49 (0.05) | 5.68 | 3.57 | 0.11 | 2.98 | 0.06 | 0.00 | 0.680 | 95.81 | 103 |
| April | 81 | Diamond-mesh | 32.28 | 3.16 | 3 | 1 | 47.24 (1.44) | 8.74 (1.45) | 0.49 (0.03) | 2.08 | 1.62 | 0.04 | 2.11 | 0.03 | 0.00 | 0.006 | 135.36 | 97 |
| April | 82 | Diamond-mesh | 32.28 | 3.12 | 3 | 1 | 50.66 (2.73) | 13.1 (2.46) | 0.48 (0.05) | 7.43 | 5.62 | 0.12 | 6.05 | 0.09 | 0.00 | 0.006 | 138.9 | 100 |
| September | 83 | Diamond-mesh | 36.38 | 2.39 | 3 | 1 | 72.1 (4.55) | 10.83 (1.08) | 0.54 (0.15) | 20.68 | 4.14 | 0.66 | 1.16 | 0.11 | 0.02 | 0.948 | 83.49 | 106 |
| September | 84 | Diamond-mesh | 36.38 | 2.84 | 2 | 1 | 99.1 (69.15) | 17.19 (1.45) | 0.95 (0.4) | 4781.77 | 73.94 | 27.70 | 2.10 | 0.41 | 0.16 | 0.996 | 76.48 | 112 |
| September | 85 | Diamond-mesh | 36.38 | 3.02 | 2 | 1 | 72.3 (7.41) | 15.68 (2.04) | 0.62 (0.16) | 54.95 | 13.61 | 1.14 | 4.14 | 0.26 | 0.02 | 0.788 | 96.98 | 109 |
| September | 86 | Diamond-mesh | 36.38 | 10.98 | 2 | 0 | 57.59 (4.23) | 16.98 (2.83) | 0.53 (0.07) | 17.93 | 10.64 | 0.27 | 8.00 | 0.15 | 0.00 | 0.058 | 130.95 | 107 |
| September | 87 | Diamond-mesh | 36.38 | 22.86 | 3 | 0 | 70.45 (8.39) | 16.37 (1.97) | 0.76 (0.14) | 70.44 | 15.07 | 1.13 | 3.89 | 0.23 | 0.02 | 0.850 | 93.82 | 109 |
| January | 88 | Square-mesh | 17.25 | 8.29 | 1 | 1 | 34 (1.02) | 7.14 (1.42) | 0.47 (0.01) | 1.03 | 0.38 | 0.01 | 2.01 | 0.01 | 0.00 | 0.407 | 112.87 | 110 |
| January | 89 | Square-mesh | 17.25 | 17.24 | 1 | 1 | 28.34 (1.96) | 7.14 (2.27) | 0.52 (0.01) | 3.86 | -1.27 | 0.01 | 5.16 | 0.00 | 0.00 | 0.000 | 161.29 | 103 |
| January | 90 | Square-mesh | 18.75 | 15.88 | 1 | 1 | 41.71 (0.67) | 5.27 (0.88) | 0.46 (0.01) | 0.45 | 0.33 | 0.01 | 0.77 | 0.01 | 0.00 | 0.304 | 112.93 | 106 |
| January | 91 | Square-mesh | 18.75 | 19.05 | 1 | 1 | 33.26 (1.09) | 4.96 (1.18) | 0.53 (0.01) | 1.19 | 0.21 | 0.01 | 1.40 | 0.00 | 0.00 | 0.006 | 147.26 | 107 |
| November | 92 | Square-mesh | 18.75 | 100.34 | 4 | 0 | 41.11 (2.8) | 12.86 (4.34) | 0.65 (0.02) | 7.87 | 0.46 | 0.04 | 18.80 | 0.06 | 0.00 | 0.007 | 129.04 | 92 |
| November | 93 | Square-mesh | 18.75 | 178.72 | 4 | 0 | 27.98 (3.07) | 0.62 (4.19) | 0.45 (0.01) | 9.42 | -2.24 | 0.00 | 17.58 | 0.00 | 0.00 | 0.039 | 121.84 | 96 |
| November | 94 | Square-mesh | 18.75 | 68.07 | 4 | 0 | 46.21 (1.81) | 12.98 (2.88) | 0.55 (0.03) | 3.28 | 2.56 | 0.04 | 8.29 | 0.05 | 0.00 | 0.060 | 121.72 | 99 |
| April | 95 | Square-mesh | 20.98 | 3.72 | 3 | 0 | 52.46 (3.05) | 12.59 (1.85) | 0.72 (0.05) | 9.32 | 4.98 | 0.15 | 3.42 | 0.07 | 0.00 | 0.873 | 81.35 | 97 |
| April | 96 | Square-mesh | 20.98 | 19.99 | 2 | 0 | 40.69 (21.81) | 34.16 (66.48) | 0.66 (0.18) | 475.74 | 1297.08 | 3.82 | 4419.10 | 11.69 | 0.03 | 0.036 | 109.8 | 85 |
| April | 97 | Square-mesh | 20.98 | 19.49 | 2 | 0 | 46.08 (1.62) | 9.82 (2.82) | 0.78 (0.02) | 2.62 | 2.74 | 0.02 | 7.97 | 0.03 | 0.00 | 0.288 | 91.82 | 85 |
| April | 98 | Square-mesh | 20.98 | 20.39 | 2 | 1 | 36.61 (0.79) | 1.03 (0.8) | 0.48 (0.02) | 0.62 | 0.30 | 0.00 | 0.63 | 0.00 | 0.00 | 0.954 | 66.08 | 87 |
| April | 99 | Square-mesh | 20.98 | 18.14 | 2 | 1 | 40.19 (1.85) | 6.66 (2.51) | 0.47 (0.02) | 3.44 | -0.72 | 0.01 | 6.32 | 0.01 | 0.00 | 0.169 | 94.16 | 82 |
| April | 100 | Square-mesh | 20.98 | 15.77 | 2 | 1 | 40.06 (0.92) | 4.64 (1.24) | 0.55 (0.02) | 0.85 | 0.25 | 0.01 | 1.53 | 0.00 | 0.00 | 0.135 | 108.15 | 93 |
| September | 101 | Square-mesh | 20.98 | 187.34 | 3 | 0 | 47.02 (3.05) | 16.46 (3.65) | 0.57 (0.04) | 9.28 | 8.32 | 0.11 | 13.33 | 0.10 | 0.00 | 0.002 | 146.93 | 102 |
| September | 102 | Square-mesh | 20.98 | 157.79 | 2 | 0 | 40.91 (3.79) | 19.32 (7.08) | 0.55 (0.04) | 14.36 | 19.26 | 0.15 | 50.13 | 0.25 | 0.00 | 0.035 | 132.69 | 105 |
| September | 103 | Square-mesh | 20.98 | 146.92 | 2 | 1 | 40.83 (1.3) | 10.14 (2.06) | 0.48 (0.02) | 1.68 | 1.55 | 0.02 | 4.24 | 0.02 | 0.00 | 0.198 | 112.85 | 101 |
| September | 104 | Square-mesh | 20.98 | 105.1 | 2 | 1 | 39.59 (1.29) | 10.4 (1.89) | 0.53 (0.02) | 1.66 | 0.90 | 0.02 | 3.57 | 0.02 | 0.00 | 0.232 | 119.5 | 109 |
| November | 105 | Square-mesh | 20.98 | 29.1 | 4 | 0 | 43.93 (1.99) | 11.18 (3.45) | 0.46 (0.02) | 3.96 | 1.21 | 0.03 | 11.88 | 0.05 | 0.00 | 0.295 | 97.77 | 91 |
| November | 106 | Square-mesh | 20.98 | 111.56 | 4 | 1 | 43.39 (3.49) | 17.01 (5.87) | 0.57 (0.04) | 12.21 | 11.06 | 0.13 | 34.44 | 0.18 | 0.00 | 0.006 | 130.16 | 92 |
| September | 107 | Square-mesh | 23.4 | 75.82 | 2 | 1 | 49.45 (2.3) | 13.43 (2.28) | 0.48 (0.04) | 5.28 | 4.40 | 0.08 | 5.22 | 0.06 | 0.00 | 0.892 | 92.96 | 111 |
| September | 108 | Square-mesh | 23.4 | 101.04 | 2 | 1 | 48.83 (2.15) | 14.5 (2.31) | 0.55 (0.03) | 4.60 | 3.94 | 0.06 | 5.32 | 0.05 | 0.00 | 0.067 | 129.72 | 107 |
| September | 109 | Square-mesh | 23.4 | 29.75 | 2 | 0 | 52.91 (2.26) | 12.65 (1.68) | 0.51 (0.04) | 5.12 | 3.30 | 0.09 | 2.84 | 0.05 | 0.00 | 0.650 | 96.92 | 103 |
| September | 110 | Square-mesh | 23.4 | 18.72 | 3 | 0 | 58.35 (3.88) | 14.7 (1.81) | 0.66 (0.07) | 15.05 | 6.38 | 0.26 | 3.26 | 0.10 | 0.00 | 0.103 | 130.24 | 111 |
| November | 111 | Square-mesh | 23.4 | 19.89 | 9 | 1 | 54.83 (2.44) | 13.01 (2.43) | 0.5 (0.04) | 5.97 | 4.95 | 0.10 | 5.90 | 0.08 | 0.00 | 0.197 | 109.72 | 98 |
| November | 112 | Square-mesh | 23.4 | 119.36 | 9 | 0 | 48.43 (2.96) | 16.56 (3.72) | 0.59 (0.04) | 8.74 | 8.11 | 0.11 | 13.81 | 0.11 | 0.00 | 0.144 | 111.86 | 97 |
| April | 113 | Square-mesh | 24.95 | 13.02 | 3 | 0 | 39.23 (1.9) | 9.34 (4.07) | 0.52 (0.02) | 3.61 | 0.75 | 0.02 | 16.59 | 0.06 | 0.00 | 0.147 | 98.72 | 85 |
| April | 114 | Square-mesh | 24.95 | 14.07 | 2 | 0 | 55.48 (3.33) | 14.37 (2.55) | 0.62 (0.05) | 11.07 | 7.48 | 0.17 | 6.51 | 0.10 | 0.00 | 0.239 | 97.06 | 88 |
| September | 115 | Square-mesh | 24.95 | 19.28 | 3 | 0 | 53.54 (1.33) | 9.02 (0.91) | 0.51 (0.03) | 1.76 | 1.01 | 0.04 | 0.82 | 0.02 | 0.00 | 0.666 | 99.24 | 106 |
| September | 116 | Square-mesh | 24.95 | 160.97 | 3 | 0 | 37.39 (2.71) | 16.14 (5.27) | 0.47 (0.03) | 7.36 | 3.45 | 0.06 | 27.82 | 0.10 | 0.00 | 0.000 | 159.36 | 101 |
| September | 117 | Square-mesh | 24.95 | 130.9 | 3 | 1 | 51.65 (2.05) | 13.76 (1.76) | 0.58 (0.03) | 4.21 | 2.92 | 0.07 | 3.09 | 0.04 | 0.00 | 0.130 | 122.55 | 106 |
| September | 118 | Square-mesh | 24.95 | 56.99 | 2 | 1 | 52.49 (2.77) | 15.59 (2.13) | 0.48 (0.04) | 7.65 | 4.99 | 0.11 | 4.53 | 0.07 | 0.00 | 0.068 | 131.75 | 109 |
| November | 119 | Square-mesh | 24.95 | 80.79 | 9 | 0 | 53.33 (2.06) | 12.93 (1.86) | 0.47 (0.04) | 4.26 | 3.10 | 0.07 | 3.46 | 0.05 | 0.00 | 0.190 | 105.85 | 94 |
| November | 120 | Square-mesh | 24.95 | 89.27 | 9 | 1 | 51.36 (4.18) | 18.91 (5.03) | 0.6 (0.05) | 17.45 | 17.20 | 0.20 | 25.31 | 0.20 | 0.00 | 0.051 | 124.19 | 100 |
| April | 121 | Square-mesh | 25.2 | 10.85 | 2 | 1 | 64 (5.29) | 16.35 (2.58) | 0.64 (0.09) | 28.03 | 12.24 | 0.44 | 6.66 | 0.18 | 0.01 | 0.495 | 88.51 | 89 |
| April | 122 | Square-mesh | 25.2 | 11.67 | 2 | 1 | 50.59 (2.5) | 11 (3.28) | 0.5 (0.04) | 6.26 | 6.49 | 0.08 | 10.75 | 0.09 | 0.00 | 0.011 | 122.4 | 89 |
| April | 123 | Square-mesh | 25.2 | 13.05 | 3 | 1 | 46.54 (1.65) | 9.53 (2.38) | 0.49 (0.03) | 2.72 | 2.87 | 0.04 | 5.67 | 0.04 | 0.00 | 0.807 | 84.83 | 97 |
| April | 124 | Square-mesh | 25.2 | 9.72 | 3 | 0 | 47.75 (2.09) | 11.51 (2.95) | 0.4 (0.03) | 4.35 | 4.50 | 0.06 | 8.69 | 0.06 | 0.00 | 0.173 | 97.16 | 85 |
| April | 125 | Square-mesh | 25.2 | 17.15 | 3 | 0 | 48.32 (1.32) | 8.86 (1.64) | 0.38 (0.02) | 1.73 | 1.55 | 0.03 | 2.68 | 0.02 | 0.00 | 0.112 | 108.79 | 92 |
| April | 126 | Square-mesh | 25.2 | 10.42 | 3 | 0 | 53.65 (2.67) | 13.14 (2.3) | 0.5 (0.05) | 7.13 | 5.19 | 0.11 | 5.30 | 0.08 | 0.00 | 0.101 | 110.73 | 93 |
| September | 127 | Square-mesh | 27.78 | 37.26 | 2 | 1 | 48.79 (1.45) | 10.18 (1.29) | 0.27 (0.02) | 2.10 | 1.53 | 0.03 | 1.66 | 0.02 | 0.00 | 0.585 | 107.17 | 111 |
| September | 128 | Square-mesh | 27.78 | 52.85 | 2 | 1 | 57.14 (3.04) | 14.55 (1.78) | 0.41 (0.05) | 9.23 | 4.82 | 0.15 | 3.17 | 0.07 | 0.00 | 0.551 | 114.38 | 117 |
| September | 129 | Square-mesh | 27.78 | 8.02 | 2 | 0 | 63.45 (3.53) | 12.11 (1.19) | 0.56 (0.09) | 12.49 | 3.74 | 0.32 | 1.41 | 0.08 | 0.01 | 0.938 | 84.56 | 106 |
| September | 130 | Square-mesh | 27.78 | 24.47 | 2 | 0 | 67.76 (5.95) | 17.63 (1.94) | 0.66 (0.1) | 35.45 | 10.22 | 0.59 | 3.78 | 0.15 | 0.01 | 0.137 | 118.77 | 103 |
| November | 131 | Square-mesh | 27.78 | 21.17 | 5 | 1 | 59.02 (4.44) | 16.36 (3.34) | 0.57 (0.07) | 19.70 | 13.43 | 0.29 | 11.19 | 0.18 | 0.00 | 0.536 | 89.12 | 91 |
| November | 132 | Square-mesh | 27.78 | 11.1 | 5 | 0 | 66.65 (5.63) | 16.61 (2.69) | 0.64 (0.09) | 31.68 | 13.70 | 0.51 | 7.23 | 0.20 | 0.01 | 0.099 | 103.3 | 86 |
| September | 133 | Square-mesh | 29.28 | 22.67 | 2 | 0 | 64.12 (5.54) | 15.56 (1.86) | 0.73 (0.09) | 30.67 | 9.52 | 0.48 | 3.47 | 0.14 | 0.01 | 0.629 | 106.49 | 112 |
| September | 134 | Square-mesh | 29.28 | 5 | 3 | 0 | 58.1 (1.77) | 9.45 (0.87) | 0.15 (0.02) | 3.12 | 1.33 | 0.04 | 0.76 | 0.01 | 0.00 | 0.728 | 111.17 | 121 |
| September | 135 | Square-mesh | 29.28 | 64.48 | 3 | 1 | 63.32 (3.87) | 13.56 (1.39) | 0.57 (0.09) | 14.98 | 4.84 | 0.33 | 1.93 | 0.10 | 0.01 | 0.447 | 110.31 | 109 |
| September | 136 | Square-mesh | 29.28 | 29.66 | 3 | 1 | 53.32 (3.48) | 15.79 (2.84) | 0.57 (0.05) | 12.12 | 8.78 | 0.18 | 8.07 | 0.12 | 0.00 | 0.283 | 94.11 | 87 |
| November | 137 | Square-mesh | 29.28 | 144.05 | 5 | 0 | 67.79 (25.66) | 29.65 (12.3) | 0.71 (0.22) | 658.45 | 302.62 | 5.60 | 151.41 | 2.51 | 0.05 | 0.036 | 115.48 | 90 |
| November | 138 | Square-mesh | 29.28 | 105.95 | 5 | 1 | 56.67 (2.84) | 13.46 (2.68) | 0.51 (0.05) | 8.07 | 6.58 | 0.13 | 7.16 | 0.10 | 0.00 | 0.762 | 85.82 | 96 |
| January | 139 | T90 | 18.88 | 10.92 | 3 | 1 | 32.5 (0.93) | 4.42 (1.13) | 0.49 (0.01) | 0.87 | 0.50 | 0.00 | 1.28 | 0.00 | 0.00 | 0.544 | 106.73 | 109 |
| January | 140 | T90 | 18.88 | 17.92 | 3 | 1 | 34.59 (1.07) | 7.51 (1.35) | 0.52 (0.01) | 1.15 | 0.16 | 0.01 | 1.81 | 0.01 | 0.00 | 0.002 | 161.66 | 113 |
| January | 141 | T90 | 18.88 | 18.32 | 3 | 1 | 25.47 (8.28) | 29.53 (35.99) | 0.53 (0.08) | 68.48 | -12.21 | 0.20 | 1294.95 | 2.59 | 0.01 | 0.004 | 148.41 | 106 |
| November | 142 | T90 | 18.88 | 91.04 | 3 | 0 | 41.35 (1.54) | 9.1 (2.38) | 0.52 (0.02) | 2.36 | 1.06 | 0.02 | 5.65 | 0.03 | 0.00 | 0.504 | 94.21 | 95 |
| November | 143 | T90 | 18.88 | 45.92 | 3 | 0 | 42.62 (1.29) | 6.93 (2.01) | 0.5 (0.02) | 1.67 | 0.62 | 0.01 | 4.03 | 0.02 | 0.00 | 0.208 | 102.75 | 92 |
| January | 144 | T90 | 20.18 | 16.52 | 3 | 1 | 37.84 (0.73) | 5.51 (0.9) | 0.46 (0.01) | 0.53 | 0.27 | 0.00 | 0.82 | 0.00 | 0.00 | 0.884 | 88.04 | 105 |
| January | 145 | T90 | 20.18 | 18.38 | 3 | 1 | 35.26 (0.92) | 6.58 (1.34) | 0.47 (0.01) | 0.84 | 0.42 | 0.01 | 1.78 | 0.01 | 0.00 | 0.032 | 136.9 | 108 |
| January | 146 | T90 | 20.18 | 13.31 | 3 | 1 | 35.17 (0.96) | 7.73 (1.55) | 0.5 (0.01) | 0.92 | 0.67 | 0.01 | 2.39 | 0.01 | 0.00 | 0.592 | 107.89 | 112 |
| April | 147 | T90 | 21.15 | 6.78 | 2 | 1 | 41.68 (1.34) | 5.72 (2.24) | 0.46 (0.02) | 1.79 | -0.19 | 0.01 | 5.03 | 0.01 | 0.00 | 0.137 | 97.15 | 83 |
| April | 148 | T90 | 21.15 | 5.87 | 2 | 1 | 51.85 (3.97) | 17.9 (4.75) | 0.66 (0.05) | 15.77 | 16.11 | 0.18 | 22.52 | 0.18 | 0.00 | 0.307 | 85.84 | 80 |
| April | 149 | T90 | 21.15 | 8.76 | 2 | 1 | 44.69 (1.46) | 8.99 (2.22) | 0.51 (0.03) | 2.13 | 1.69 | 0.03 | 4.92 | 0.03 | 0.00 | 0.035 | 115.75 | 90 |
| April | 150 | T90 | 21.15 | 12.07 | 2 | 0 | 41.1 (1.04) | 3.08 (1.47) | 0.43 (0.02) | 1.08 | 1.11 | 0.01 | 2.17 | 0.01 | 0.00 | 0.011 | 124.75 | 91 |
| April | 151 | T90 | 21.15 | 17.34 | 2 | 0 | 44.63 (1.8) | 10.87 (2.81) | 0.49 (0.03) | 3.23 | 3.78 | 0.04 | 7.89 | 0.06 | 0.00 | 0.531 | 95.27 | 97 |
| April | 152 | T90 | 21.15 | 22.03 | 2 | 0 | 42.09 (0.98) | 7.8 (1.44) | 0.42 (0.02) | 0.97 | 0.59 | 0.01 | 2.07 | 0.01 | 0.00 | 0.523 | 90.56 | 92 |
| January | 153 | T90 | 22.5 | 15.81 | 3 | 1 | 44.2 (2.26) | 17.19 (2.86) | 0.5 (0.03) | 5.13 | 4.18 | 0.06 | 8.16 | 0.06 | 0.00 | 0.263 | 117.92 | 109 |
| January | 154 | T90 | 22.5 | 3.68 | 3 | 1 | 45.19 (1.53) | 10.11 (1.89) | 0.47 (0.03) | 2.35 | 2.34 | 0.04 | 3.56 | 0.04 | 0.00 | 0.838 | 96.36 | 111 |
| January | 155 | T90 | 22.5 | 14.5 | 3 | 1 | 46.5 (1.47) | 11.94 (1.57) | 0.56 (0.03) | 2.16 | 1.71 | 0.03 | 2.47 | 0.03 | 0.00 | 0.070 | 130.45 | 108 |
| January | 156 | T90 | 22.5 | 1.62 | 4 | 0 | 48.79 (1.84) | 8.56 (1.55) | 0.43 (0.05) | 3.38 | 2.40 | 0.08 | 2.41 | 0.05 | 0.00 | 0.001 | 139.89 | 89 |
| January | 157 | T90 | 22.5 | 9.92 | 3 | 0 | 41.54 (1.93) | 11.69 (2.32) | 0.5 (0.03) | 3.71 | 2.69 | 0.05 | 5.39 | 0.04 | 0.00 | 0.001 | 155.77 | 104 |
| November | 158 | T90 | 22.5 | 130.39 | 5 | 0 | 46.8 (1.34) | 8.89 (2.03) | 0.52 (0.03) | 1.81 | 1.85 | 0.03 | 4.12 | 0.04 | 0.00 | 0.987 | 60.42 | 87 |
| April | 159 | T90 | 24.35 | 9.76 | 1 | 1 | 42.98 (2.35) | 12.48 (5.91) | 0.54 (0.03) | 5.55 | 5.41 | 0.05 | 34.94 | 0.13 | 0.00 | 0.612 | 78.73 | 83 |
| April | 160 | T90 | 24.35 | 10.15 | 1 | 1 | 47.04 (1.75) | 11.03 (2.43) | 0.47 (0.03) | 3.07 | 2.92 | 0.05 | 5.89 | 0.05 | 0.00 | 0.756 | 81.33 | 91 |
| April | 161 | T90 | 24.35 | 2.59 | 1 | 0 | 50.57 (2.53) | 13.31 (2.86) | 0.55 (0.04) | 6.38 | 6.02 | 0.09 | 8.18 | 0.09 | 0.00 | 0.341 | 99.05 | 94 |
| April | 162 | T90 | 24.35 | 7.36 | 1 | 0 | 43.64 (1.08) | 6.85 (1.61) | 0.45 (0.02) | 1.16 | 0.56 | 0.01 | 2.60 | 0.01 | 0.00 | 0.674 | 79.6 | 86 |
| April | 163 | T90 | 24.35 | 8.13 | 1 | 0 | 49.25 (1.85) | 11.84 (2.44) | 0.62 (0.03) | 3.41 | 2.86 | 0.04 | 5.95 | 0.04 | 0.00 | 0.153 | 100.47 | 87 |
| April | 164 | T90 | 24.35 | 7.92 | 2 | 0 | 49.14 (1.47) | 9.48 (2.1) | 0.67 (0.02) | 2.15 | 1.31 | 0.03 | 4.41 | 0.02 | 0.00 | 0.021 | 111.2 | 83 |
| April | 165 | T90 | 24.35 | 13.52 | 2 | 1 | 33.33 (4.92) | 0.15 (4.34) | 0.5 (0.01) | 24.22 | -20.42 | 0.00 | 18.83 | 0.00 | 0.00 | 0.047 | 111.32 | 88 |
| September | 166 | T90 | 24.63 | 24.26 | 2 | 0 | 59.42 (2.28) | 11.09 (1.31) | 0.84 (0.03) | 5.21 | 2.63 | 0.06 | 1.72 | 0.03 | 0.00 | 0.617 | 94.22 | 99 |
| September | 167 | T90 | 24.63 | 10.5 | 2 | 1 | 57.93 (2.85) | 13.34 (1.94) | 0.52 (0.06) | 8.12 | 4.85 | 0.15 | 3.77 | 0.08 | 0.00 | 0.491 | 100.66 | 101 |
| September | 168 | T90 | 24.63 | 28.33 | 2 | 1 | 50.16 (1.6) | 10.8 (2.06) | 0.35 (0.03) | 2.55 | 2.42 | 0.04 | 4.25 | 0.03 | 0.00 | 0.114 | 122.69 | 105 |
| November | 169 | T90 | 24.63 | 93.02 | 5 | 0 | 47.28 (3.56) | 16.72 (5.44) | 0.53 (0.05) | 12.64 | 15.29 | 0.16 | 29.59 | 0.22 | 0.00 | 0.157 | 105.62 | 92 |
| November | 170 | T90 | 24.63 | 97.5 | 6 | 1 | 51.02 (1.25) | 8.25 (1.31) | 0.54 (0.03) | 1.57 | 1.31 | 0.03 | 1.71 | 0.03 | 0.00 | 0.969 | 73.59 | 98 |
| September | 171 | T90 | 27.55 | 35.99 | 4 | 0 | 55.98 (1.91) | 12 (1.33) | 0.55 (0.04) | 3.64 | 2.07 | 0.07 | 1.76 | 0.03 | 0.00 | 0.578 | 106.47 | 110 |
| September | 172 | T90 | 27.55 | 33.52 | 4 | 0 | 60.8 (7.91) | 19.13 (4.24) | 0.59 (0.11) | 62.59 | 31.79 | 0.88 | 17.95 | 0.43 | 0.01 | 0.087 | 123.01 | 103 |
| September | 173 | T90 | 27.55 | 102.58 | 4 | 1 | 45.48 (2.47) | 15.7 (3.67) | 0.44 (0.03) | 6.10 | 6.63 | 0.07 | 13.46 | 0.09 | 0.00 | 0.501 | 104.31 | 105 |
| September | 174 | T90 | 27.55 | 97.82 | 4 | 1 | 51.02 (2.93) | 13.82 (3.51) | 0.42 (0.04) | 8.58 | 8.31 | 0.11 | 12.30 | 0.11 | 0.00 | 0.078 | 119.56 | 99 |
| November | 175 | T90 | 27.55 | 55.77 | 6 | 1 | 51.39 (1.81) | 11.69 (2.4) | 0.47 (0.03) | 3.28 | 3.20 | 0.05 | 5.78 | 0.05 | 0.00 | 0.897 | 79.87 | 97 |
| November | 176 | T90 | 27.55 | 24.55 | 5 | 0 | 58.57 (1.43) | 8.2 (1.06) | 0.36 (0.03) | 2.03 | 1.32 | 0.04 | 1.13 | 0.02 | 0.00 | 0.952 | 71.57 | 93 |
| April | 177 | T90 | 27.83 | 8.28 | 1 | 0 | 67.93 (18.09) | 27.69 (9.64) | 0.67 (0.17) | 327.15 | 165.04 | 3.04 | 92.95 | 1.49 | 0.03 | 0.840 | 72.08 | 85 |
| April | 178 | T90 | 27.83 | 6.1 | 1 | 0 | 60.08 (2.06) | 10.56 (1.22) | 0.64 (0.05) | 4.22 | 2.12 | 0.09 | 1.48 | 0.04 | 0.00 | 0.822 | 74.77 | 87 |
| April | 179 | T90 | 27.83 | 4.01 | 1 | 0 | 56.66 (1.34) | 8.52 (1.01) | 0.54 (0.03) | 1.80 | 1.08 | 0.04 | 1.01 | 0.02 | 0.00 | 0.965 | 67.21 | 90 |
| April | 180 | T90 | 27.83 | 2.26 | 1 | 1 | 45.53 (1.28) | 7.61 (1.75) | 0.34 (0.02) | 1.63 | 1.23 | 0.02 | 3.05 | 0.02 | 0.00 | 0.050 | 114.34 | 91 |
| April | 181 | T90 | 27.83 | 5.81 | 1 | 1 | 52.78 (1.96) | 12.01 (1.99) | 0.52 (0.03) | 3.84 | 3.06 | 0.06 | 3.98 | 0.04 | 0.00 | 0.648 | 84.35 | 90 |
| April | 182 | T90 | 27.83 | 14.59 | 1 | 1 | 45.09 (1.11) | 8.42 (1.56) | 0.45 (0.02) | 1.24 | 1.17 | 0.02 | 2.43 | 0.02 | 0.00 | 0.827 | 83.87 | 97 |
| September | 183 | T90 | 29.03 | 9.5 | 3 | 1 | 58.74 (1.16) | 6.44 (0.58) | 0.86 (0.02) | 1.33 | 0.57 | 0.02 | 0.34 | 0.01 | 0.00 | 0.996 | 54.75 | 85 |
| September | 184 | T90 | 29.03 | 66.23 | 4 | 1 | 56.02 (2.94) | 14.42 (1.88) | 0.44 (0.05) | 8.67 | 4.88 | 0.14 | 3.52 | 0.07 | 0.00 | 0.699 | 98.93 | 107 |
| September | 185 | T90 | 29.03 | 165.19 | 4 | 0 | 50.3 (3.52) | 17.74 (3.59) | 0.61 (0.04) | 12.38 | 10.76 | 0.15 | 12.88 | 0.12 | 0.00 | 0.295 | 113.37 | 106 |
| September | 186 | T90 | 29.03 | 100.94 | 4 | 0 | 55.9 (3.2) | 15.25 (2.18) | 0.51 (0.06) | 10.25 | 6.04 | 0.17 | 4.77 | 0.09 | 0.00 | 0.290 | 105.27 | 98 |
| November | 187 | T90 | 29.03 | 103.24 | 5 | 0 | 46.98 (1.96) | 12.62 (2.8) | 0.5 (0.03) | 3.86 | 3.72 | 0.06 | 7.82 | 0.07 | 0.00 | 0.079 | 111.77 | 92 |
| November | 188 | T90 | 29.03 | 109.23 | 4 | 1 | 56.76 (6.23) | 19.2 (4.8) | 0.68 (0.07) | 38.81 | 27.33 | 0.44 | 23.03 | 0.30 | 0.01 | 0.854 | 82.43 | 97 |
| April | 189 | T90 | 31.28 | 5.49 | 3 | 1 | 66.37 (3.2) | 11.54 (1.22) | 0.53 (0.08) | 10.22 | 3.37 | 0.25 | 1.48 | 0.07 | 0.01 | 0.557 | 88.41 | 91 |
| April | 190 | T90 | 31.28 | 5.31 | 3 | 1 | 56.11 (3.58) | 13.16 (3.05) | 0.47 (0.06) | 12.81 | 9.84 | 0.20 | 9.28 | 0.15 | 0.00 | 0.504 | 86.19 | 87 |
| April | 191 | T90 | 31.28 | 5.72 | 3 | 1 | 57.78 (1.53) | 9.01 (1.13) | 0.69 (0.03) | 2.35 | 1.41 | 0.04 | 1.27 | 0.02 | 0.00 | 0.880 | 72.74 | 88 |
| April | 192 | T90 | 31.28 | 4.47 | 3 | 0 | 57.4 (1.33) | 8.06 (1.07) | 0.54 (0.03) | 1.76 | 1.08 | 0.03 | 1.14 | 0.02 | 0.00 | 0.515 | 79.85 | 81 |
| April | 193 | T90 | 31.28 | 3.81 | 3 | 0 | 63.52 (2.75) | 11.2 (1.28) | 0.62 (0.06) | 7.58 | 3.05 | 0.17 | 1.64 | 0.06 | 0.00 | 0.907 | 71.94 | 89 |
| April | 194 | T90 | 31.28 | 4.68 | 3 | 0 | 56.37 (2.74) | 12.27 (1.84) | 0.48 (0.05) | 7.51 | 4.50 | 0.13 | 3.38 | 0.07 | 0.00 | 0.404 | 103.8 | 101 |
| September | 195 | T90 | 31.4 | 18.46 | 2 | 1 | 67.78 (4.69) | 14.95 (1.41) | 0.5 (0.11) | 22.01 | 5.79 | 0.49 | 1.98 | 0.12 | 0.01 | 0.378 | 118.09 | 114 |
| September | 196 | T90 | 31.4 | 26.94 | 3 | 1 | 57.29 (4.85) | 17.5 (3.02) | 0.51 (0.07) | 23.54 | 13.42 | 0.35 | 9.14 | 0.19 | 0.01 | 0.578 | 101.54 | 105 |
| September | 197 | T90 | 31.4 | 28.55 | 3 | 0 | 70 (10.24) | 22.26 (3.41) | 0.62 (0.15) | 104.81 | 31.72 | 1.50 | 11.61 | 0.42 | 0.02 | 0.101 | 123.86 | 105 |
| September | 198 | T90 | 31.4 | 33.4 | 4 | 0 | 53.51 (2.35) | 13.65 (1.9) | 0.51 (0.04) | 5.54 | 3.85 | 0.09 | 3.61 | 0.06 | 0.00 | 0.298 | 114.25 | 107 |
| November | 199 | T90 | 31.4 | 89.33 | 4 | 1 | 42.01 (1.67) | 7.53 (2.22) | 0.42 (0.02) | 2.78 | 0.13 | 0.01 | 4.93 | 0.01 | 0.00 | 0.247 | 97.74 | 89 |
| September | 200 | T90 | 36.5 | 65.72 | 4 | 1 | 63.78 (4.61) | 14.39 (1.83) | 0.48 (0.1) | 21.22 | 7.64 | 0.44 | 3.33 | 0.15 | 0.01 | 0.480 | 111.07 | 111 |
| September | 201 | T90 | 36.5 | 146.8 | 4 | 1 | 61.43 (5.76) | 19.28 (3.22) | 0.44 (0.08) | 33.22 | 16.81 | 0.46 | 10.37 | 0.22 | 0.01 | 0.266 | 112.57 | 104 |
| September | 202 | T90 | 36.5 | 50.8 | 3 | 0 | 54.09 (3.54) | 15.74 (2.94) | 0.49 (0.05) | 12.51 | 9.04 | 0.18 | 8.66 | 0.12 | 0.00 | 0.042 | 123.55 | 98 |
| September | 203 | T90 | 36.5 | 52.9 | 3 | 0 | 57.97 (2.37) | 11.38 (1.33) | 0.44 (0.05) | 5.63 | 2.81 | 0.12 | 1.76 | 0.05 | 0.00 | 0.136 | 120.98 | 105 |
| September | 204 | T90 | 36.5 | 71.36 | 3 | 0 | 64.62 (8.02) | 19.8 (3.39) | 0.68 (0.11) | 64.39 | 25.47 | 0.85 | 11.48 | 0.32 | 0.01 | 0.135 | 120.04 | 104 |
| November | 205 | T90 | 36.5 | 150.29 | 4 | 1 | 30.85 (5.96) | 16.01 (14.31) | 0.48 (0.03) | 35.49 | -53.39 | -0.03 | 204.69 | 0.32 | 0.00 | 0.391 | 100.22 | 97 |
