# Supplementary material for: Predictive framework for codend size selection of brown shrimp (Crangon crangon) in the North Sea beam-trawl fishery
Source: PLoS One. 2018 Jul 16;13(7):e0200464. doi: 10.1371/journal.pone.0200464 (PMC6047787; doi:10.1371/journal.pone.0200464)
Supplement: S3 Table — Values L50mean and SRmean estimated by the predictive framework. The numerical information presented here was used to plot the isolines for codend retention in Fig 5. (DOCX) [file pone.0200464.s003.docx]

**S3 Table. Predicted lengths of brown shrimps associated to given retention probabilities (from *r* = 5% to *r* = 95%) for different codend types and mesh sizes.** Values *L50_mean_* and *SR_mean_* estimated by the predictive framework. The numerical information presented here was used to plot the isolines for codend retention in Fig 5.

| **Codend Type** | **Mesh size** | ***L50_mean_*** | ***Sr_mean_*** | **r5** | **r10** | **r15** | **r20** | **r25** | **r30** | **r35** | **r40** | **r45** | **r50** | **r55** | **r60** | **r65** | **r70** | **r75** | **r80** | **r85** | **r90** | **r95** |
| --- | --- | --- | --- | --- | --- | --- | --- | --- | --- | --- | --- | --- | --- | --- | --- | --- | --- | --- | --- | --- | --- | --- |
| *Diamond-mesh* | 19 | 35.3 | 7.1 | 25.8 | 28.2 | 29.7 | 30.8 | 31.7 | 32.6 | 33.3 | 34.0 | 34.6 | 35.3 | 35.9 | 36.6 | 37.3 | 38.0 | 38.8 | 39.8 | 40.9 | 42.4 | 44.8 |
|  | 20 | 36.9 | 7.5 | 26.9 | 29.5 | 31.1 | 32.2 | 33.2 | 34.1 | 34.8 | 35.6 | 36.3 | 36.9 | 37.6 | 38.3 | 39.0 | 39.8 | 40.7 | 41.6 | 42.8 | 44.4 | 46.9 |
|  | 21 | 38.6 | 7.8 | 28.1 | 30.7 | 32.4 | 33.6 | 34.7 | 35.5 | 36.4 | 37.1 | 37.9 | 38.6 | 39.3 | 40.0 | 40.8 | 41.6 | 42.5 | 43.5 | 44.8 | 46.4 | 49.1 |
|  | 22 | 40.2 | 8.2 | 29.2 | 32.0 | 33.7 | 35.0 | 36.1 | 37.0 | 37.9 | 38.7 | 39.4 | 40.2 | 40.9 | 41.7 | 42.5 | 43.3 | 44.3 | 45.4 | 46.7 | 48.4 | 51.2 |
|  | 23 | 41.8 | 8.6 | 30.3 | 33.2 | 35.0 | 36.4 | 37.5 | 38.5 | 39.3 | 40.2 | 41.0 | 41.8 | 42.5 | 43.3 | 44.2 | 45.1 | 46.1 | 47.2 | 48.5 | 50.3 | 53.3 |
|  | 24 | 43.3 | 9.0 | 31.3 | 34.4 | 36.3 | 37.7 | 38.9 | 39.9 | 40.8 | 41.7 | 42.5 | 43.3 | 44.2 | 45.0 | 45.9 | 46.8 | 47.8 | 49.0 | 50.4 | 52.3 | 55.3 |
|  | 25 | 44.9 | 9.3 | 32.4 | 35.6 | 37.5 | 39.0 | 40.2 | 41.3 | 42.3 | 43.2 | 44.0 | 44.9 | 45.7 | 46.6 | 47.5 | 48.5 | 49.5 | 50.8 | 52.2 | 54.2 | 57.4 |
|  | 26 | 46.4 | 9.7 | 33.4 | 36.7 | 38.7 | 40.3 | 41.6 | 42.7 | 43.7 | 44.6 | 45.5 | 46.4 | 47.3 | 48.2 | 49.1 | 50.1 | 51.3 | 52.5 | 54.1 | 56.1 | 59.4 |
|  | 27 | 47.9 | 10.1 | 34.4 | 37.8 | 40.0 | 41.6 | 42.9 | 44.0 | 45.1 | 46.1 | 47.0 | 47.9 | 48.8 | 49.8 | 50.7 | 51.8 | 52.9 | 54.3 | 55.9 | 58.0 | 61.4 |
|  | 28 | 49.4 | 10.4 | 35.4 | 38.9 | 41.1 | 42.8 | 44.2 | 45.4 | 46.5 | 47.5 | 48.4 | 49.4 | 50.3 | 51.3 | 52.3 | 53.4 | 54.6 | 56.0 | 57.6 | 59.8 | 63.4 |
|  | 29 | 50.9 | 10.8 | 36.4 | 40.0 | 42.3 | 44.0 | 45.4 | 46.7 | 47.8 | 48.9 | 49.9 | 50.9 | 51.8 | 52.9 | 53.9 | 55.0 | 56.3 | 57.7 | 59.4 | 61.7 | 65.4 |
|  | 30 | 52.3 | 11.2 | 37.3 | 41.1 | 43.5 | 45.2 | 46.7 | 48.0 | 49.1 | 50.2 | 51.3 | 52.3 | 53.3 | 54.4 | 55.5 | 56.6 | 57.9 | 59.4 | 61.1 | 63.5 | 67.3 |
|  | 31 | 53.7 | 11.6 | 38.2 | 42.2 | 44.6 | 46.4 | 47.9 | 49.3 | 50.5 | 51.6 | 52.7 | 53.7 | 54.8 | 55.9 | 57.0 | 58.2 | 59.5 | 61.0 | 62.9 | 65.3 | 69.2 |
|  | 32 | 55.1 | 11.9 | 39.1 | 43.2 | 45.7 | 47.6 | 49.2 | 50.5 | 51.8 | 52.9 | 54.0 | 55.1 | 56.2 | 57.3 | 58.5 | 59.7 | 61.1 | 62.7 | 64.5 | 67.1 | 71.1 |
|  | 33 | 56.5 | 12.3 | 40.0 | 44.2 | 46.8 | 48.7 | 50.3 | 51.8 | 53.0 | 54.2 | 55.4 | 56.5 | 57.6 | 58.8 | 60.0 | 61.3 | 62.7 | 64.3 | 66.2 | 68.8 | 73.0 |
|  | 34 | 57.9 | 12.7 | 40.9 | 45.2 | 47.8 | 49.9 | 51.5 | 53.0 | 54.3 | 55.5 | 56.7 | 57.9 | 59.0 | 60.2 | 61.4 | 62.8 | 64.2 | 65.9 | 67.9 | 70.5 | 74.9 |
|  | 35 | 59.2 | 13.1 | 41.7 | 46.1 | 48.9 | 51.0 | 52.7 | 54.2 | 55.5 | 56.8 | 58.0 | 59.2 | 60.4 | 61.6 | 62.9 | 64.2 | 65.7 | 67.4 | 69.5 | 72.3 | 76.7 |
|  | 36 | 60.5 | 13.4 | 42.5 | 47.1 | 49.9 | 52.0 | 53.8 | 55.3 | 56.7 | 58.0 | 59.3 | 60.5 | 61.7 | 63.0 | 64.3 | 65.7 | 67.2 | 69.0 | 71.1 | 74.0 | 78.5 |
| *Square-mesh* | 17 | 34.4 | 5.9 | 26.5 | 28.5 | 29.7 | 30.7 | 31.4 | 32.1 | 32.7 | 33.3 | 33.8 | 34.4 | 34.9 | 35.4 | 36.0 | 36.6 | 37.3 | 38.1 | 39.0 | 40.2 | 42.2 |
|  | 18 | 36.4 | 6.4 | 27.8 | 30.0 | 31.3 | 32.3 | 33.2 | 33.9 | 34.6 | 35.2 | 35.8 | 36.4 | 37.0 | 37.6 | 38.2 | 38.9 | 39.6 | 40.4 | 41.5 | 42.8 | 45.0 |
|  | 19 | 38.4 | 7.0 | 29.0 | 31.4 | 32.9 | 34.0 | 34.9 | 35.7 | 36.4 | 37.1 | 37.8 | 38.4 | 39.0 | 39.7 | 40.4 | 41.1 | 41.9 | 42.8 | 43.9 | 45.4 | 47.8 |
|  | 20 | 40.4 | 7.6 | 30.2 | 32.8 | 34.4 | 35.6 | 36.6 | 37.5 | 38.3 | 39.0 | 39.7 | 40.4 | 41.1 | 41.8 | 42.6 | 43.4 | 44.2 | 45.2 | 46.4 | 48.0 | 50.6 |
|  | 21 | 42.4 | 8.3 | 31.4 | 34.2 | 35.9 | 37.2 | 38.3 | 39.3 | 40.1 | 40.9 | 41.7 | 42.4 | 43.2 | 44.0 | 44.8 | 45.6 | 46.6 | 47.7 | 49.0 | 50.7 | 53.5 |
|  | 22 | 44.5 | 8.9 | 32.5 | 35.5 | 37.4 | 38.8 | 40.0 | 41.0 | 42.0 | 42.8 | 43.7 | 44.5 | 45.3 | 46.1 | 47.0 | 47.9 | 48.9 | 50.1 | 51.5 | 53.4 | 56.4 |
|  | 23 | 46.5 | 9.6 | 33.6 | 36.9 | 38.9 | 40.4 | 41.7 | 42.8 | 43.8 | 44.7 | 45.6 | 46.5 | 47.4 | 48.3 | 49.2 | 50.2 | 51.3 | 52.6 | 54.1 | 56.1 | 59.4 |
|  | 24 | 48.5 | 10.4 | 34.6 | 38.1 | 40.3 | 42.0 | 43.3 | 44.5 | 45.6 | 46.6 | 47.6 | 48.5 | 49.5 | 50.4 | 51.4 | 52.5 | 53.7 | 55.1 | 56.7 | 58.9 | 62.4 |
|  | 25 | 50.5 | 11.1 | 35.6 | 39.4 | 41.7 | 43.5 | 45.0 | 46.2 | 47.4 | 48.5 | 49.5 | 50.5 | 51.6 | 52.6 | 53.7 | 54.8 | 56.1 | 57.6 | 59.3 | 61.7 | 65.5 |
|  | 26 | 52.6 | 11.9 | 36.6 | 40.6 | 43.1 | 45.0 | 46.6 | 48.0 | 49.2 | 50.4 | 51.5 | 52.6 | 53.6 | 54.8 | 55.9 | 57.2 | 58.5 | 60.1 | 62.0 | 64.5 | 68.6 |
|  | 27 | 54.6 | 12.8 | 37.5 | 41.8 | 44.5 | 46.5 | 48.2 | 49.7 | 51.0 | 52.2 | 53.4 | 54.6 | 55.7 | 56.9 | 58.2 | 59.5 | 61.0 | 62.6 | 64.7 | 67.3 | 71.7 |
|  | 28 | 56.6 | 13.6 | 38.3 | 43.0 | 45.8 | 48.0 | 49.8 | 51.3 | 52.8 | 54.1 | 55.4 | 56.6 | 57.8 | 59.1 | 60.4 | 61.9 | 63.4 | 65.2 | 67.4 | 70.2 | 74.9 |
|  | 29 | 58.6 | 14.5 | 39.2 | 44.1 | 47.2 | 49.5 | 51.4 | 53.0 | 54.5 | 55.9 | 57.3 | 58.6 | 59.9 | 61.3 | 62.7 | 64.2 | 65.9 | 67.8 | 70.1 | 73.1 | 78.1 |
| *T90* | 19 | 39.0 | 7.5 | 28.9 | 31.4 | 33.0 | 34.2 | 35.2 | 36.1 | 36.8 | 37.6 | 38.3 | 39.0 | 39.7 | 40.4 | 41.1 | 41.9 | 42.7 | 43.7 | 44.9 | 46.5 | 49.1 |
|  | 20 | 40.4 | 7.9 | 29.8 | 32.5 | 34.2 | 35.4 | 36.5 | 37.4 | 38.2 | 39.0 | 39.7 | 40.4 | 41.2 | 41.9 | 42.7 | 43.5 | 44.4 | 45.4 | 46.7 | 48.4 | 51.1 |
|  | 21 | 41.9 | 8.3 | 30.7 | 33.6 | 35.3 | 36.6 | 37.7 | 38.7 | 39.6 | 40.4 | 41.1 | 41.9 | 42.7 | 43.4 | 44.3 | 45.1 | 46.1 | 47.2 | 48.5 | 50.2 | 53.1 |
|  | 22 | 43.4 | 8.7 | 31.7 | 34.6 | 36.5 | 37.9 | 39.0 | 40.0 | 40.9 | 41.8 | 42.6 | 43.4 | 44.2 | 45.0 | 45.8 | 46.7 | 47.7 | 48.9 | 50.3 | 52.1 | 55.1 |
|  | 23 | 44.8 | 9.1 | 32.6 | 35.7 | 37.6 | 39.1 | 40.3 | 41.3 | 42.3 | 43.2 | 44.0 | 44.8 | 45.7 | 46.5 | 47.4 | 48.4 | 49.4 | 50.6 | 52.1 | 54.0 | 57.1 |
|  | 24 | 46.3 | 9.5 | 33.5 | 36.8 | 38.8 | 40.3 | 41.6 | 42.6 | 43.6 | 44.6 | 45.4 | 46.3 | 47.2 | 48.1 | 49.0 | 50.0 | 51.1 | 52.3 | 53.8 | 55.8 | 59.1 |
|  | 25 | 47.8 | 9.9 | 34.5 | 37.9 | 39.9 | 41.5 | 42.8 | 44.0 | 45.0 | 46.0 | 46.9 | 47.8 | 48.7 | 49.6 | 50.6 | 51.6 | 52.7 | 54.0 | 55.6 | 57.7 | 61.1 |
|  | 26 | 49.3 | 10.3 | 35.4 | 38.9 | 41.1 | 42.7 | 44.1 | 45.3 | 46.3 | 47.4 | 48.3 | 49.3 | 50.2 | 51.2 | 52.2 | 53.2 | 54.4 | 55.8 | 57.4 | 59.6 | 63.1 |
|  | 27 | 50.7 | 10.7 | 36.4 | 40.0 | 42.3 | 44.0 | 45.4 | 46.6 | 47.7 | 48.7 | 49.7 | 50.7 | 51.7 | 52.7 | 53.7 | 54.9 | 56.1 | 57.5 | 59.2 | 61.4 | 65.1 |
|  | 28 | 52.2 | 11.1 | 37.3 | 41.1 | 43.4 | 45.2 | 46.6 | 47.9 | 49.1 | 50.1 | 51.2 | 52.2 | 53.2 | 54.2 | 55.3 | 56.5 | 57.8 | 59.2 | 61.0 | 63.3 | 67.1 |
|  | 29 | 53.7 | 11.5 | 38.2 | 42.2 | 44.6 | 46.4 | 47.9 | 49.2 | 50.4 | 51.5 | 52.6 | 53.7 | 54.7 | 55.8 | 56.9 | 58.1 | 59.4 | 60.9 | 62.8 | 65.2 | 69.1 |
|  | 30 | 55.1 | 11.9 | 39.2 | 43.2 | 45.7 | 47.6 | 49.2 | 50.5 | 51.8 | 52.9 | 54.0 | 55.1 | 56.2 | 57.3 | 58.5 | 59.7 | 61.1 | 62.6 | 64.5 | 67.0 | 71.1 |
|  | 31 | 56.6 | 12.3 | 40.1 | 44.3 | 46.9 | 48.8 | 50.5 | 51.9 | 53.1 | 54.3 | 55.5 | 56.6 | 57.7 | 58.9 | 60.1 | 61.4 | 62.8 | 64.4 | 66.3 | 68.9 | 73.1 |
|  | 32 | 58.1 | 12.7 | 41.0 | 45.4 | 48.0 | 50.1 | 51.7 | 53.2 | 54.5 | 55.7 | 56.9 | 58.1 | 59.2 | 60.4 | 61.7 | 63.0 | 64.4 | 66.1 | 68.1 | 70.8 | 75.1 |
|  | 33 | 59.5 | 13.1 | 42.0 | 46.4 | 49.2 | 51.3 | 53.0 | 54.5 | 55.9 | 57.1 | 58.3 | 59.5 | 60.7 | 62.0 | 63.2 | 64.6 | 66.1 | 67.8 | 69.9 | 72.6 | 77.1 |
|  | 34 | 61.0 | 13.5 | 42.9 | 47.5 | 50.4 | 52.5 | 54.3 | 55.8 | 57.2 | 58.5 | 59.8 | 61.0 | 62.2 | 63.5 | 64.8 | 66.2 | 67.8 | 69.5 | 71.7 | 74.5 | 79.1 |
|  | 35 | 62.5 | 13.9 | 43.9 | 48.6 | 51.5 | 53.7 | 55.5 | 57.1 | 58.6 | 59.9 | 61.2 | 62.5 | 63.8 | 65.0 | 66.4 | 67.8 | 69.4 | 71.3 | 73.5 | 76.4 | 81.1 |
|  | 36 | 64.0 | 14.3 | 44.8 | 49.7 | 52.7 | 54.9 | 56.8 | 58.4 | 59.9 | 61.3 | 62.6 | 64.0 | 65.3 | 66.6 | 68.0 | 69.5 | 71.1 | 73.0 | 75.2 | 78.2 | 83.1 |
