# Supplementary material for: Predictive framework for codend size selection of brown shrimp (Crangon crangon) in the North Sea beam-trawl fishery
Source: PLoS One. 2018 Jul 16;13(7):e0200464. doi: 10.1371/journal.pone.0200464 (PMC6047787; doi:10.1371/journal.pone.0200464)
Supplement: S1 Fig — Grey polygon and black line represent the length distributions of brown shrimp in control and test codends, respectively. Mark circles represent the experimental catch proportion in the test codend relative to the total catch, obtained upon raised data. Blue line is the φ(l) curve estimated according to Eqs 2 and 3. Dotted red line represents species minimum landing size at 50 mm length. Hauls ordered by codend type, mesh size, and cruise. (PDF) [file pone.0200464.s004.pdf]

S1 Figure. By-haul catch sharing between control and test codends. Grey polygon and black line represent the length distributions of brown shrimp in control and test codends, respectively. Mark circles represent the experimental catch proportion in the test codend relative to the total catch, obtained upon raised data. Blue line is the φ(l) curve estimated according to Eq 2 and Eq 3. Dotted red line represents species minimum landing size at 50 mm length.

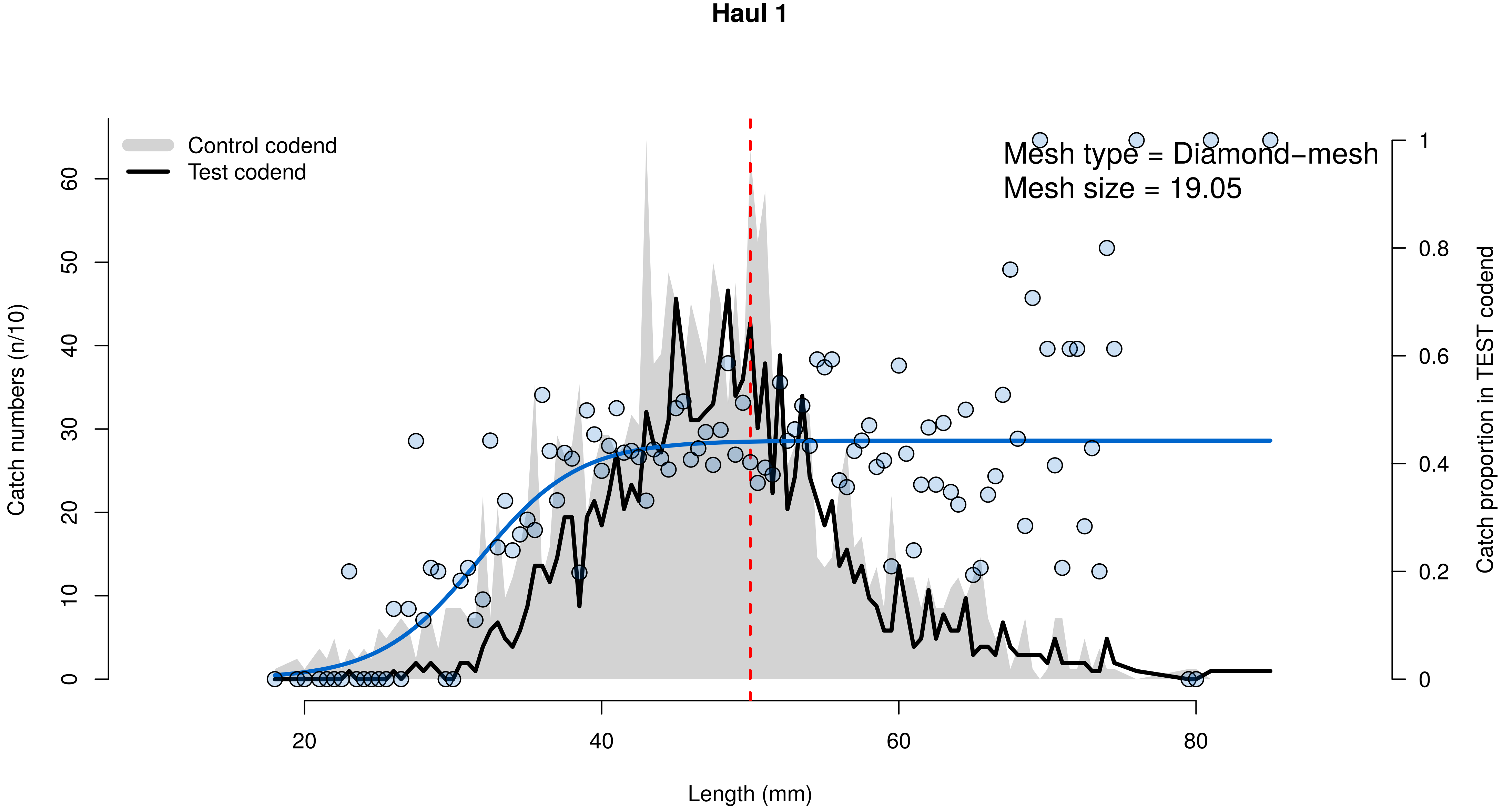

Haul 2

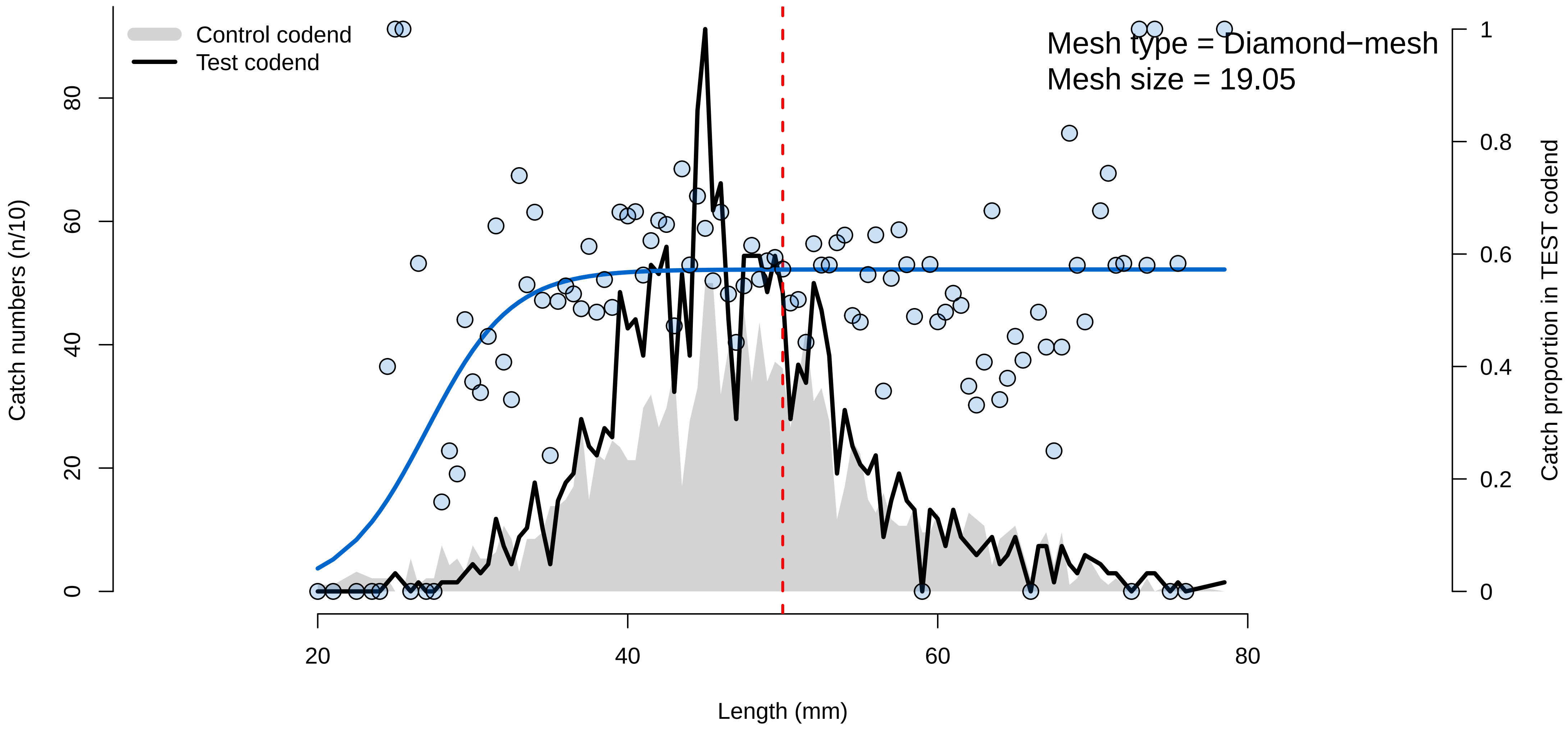

Control codend
Test codend
Mesh type = Diamond−mesh
Mesh size = 19.05
Catch numbers (n/10)
0
20
40
60
80
Catch proportion in TEST codend
0
0.2
0.4
0.6
0.8
1
20
40
60
80
Length (mm)

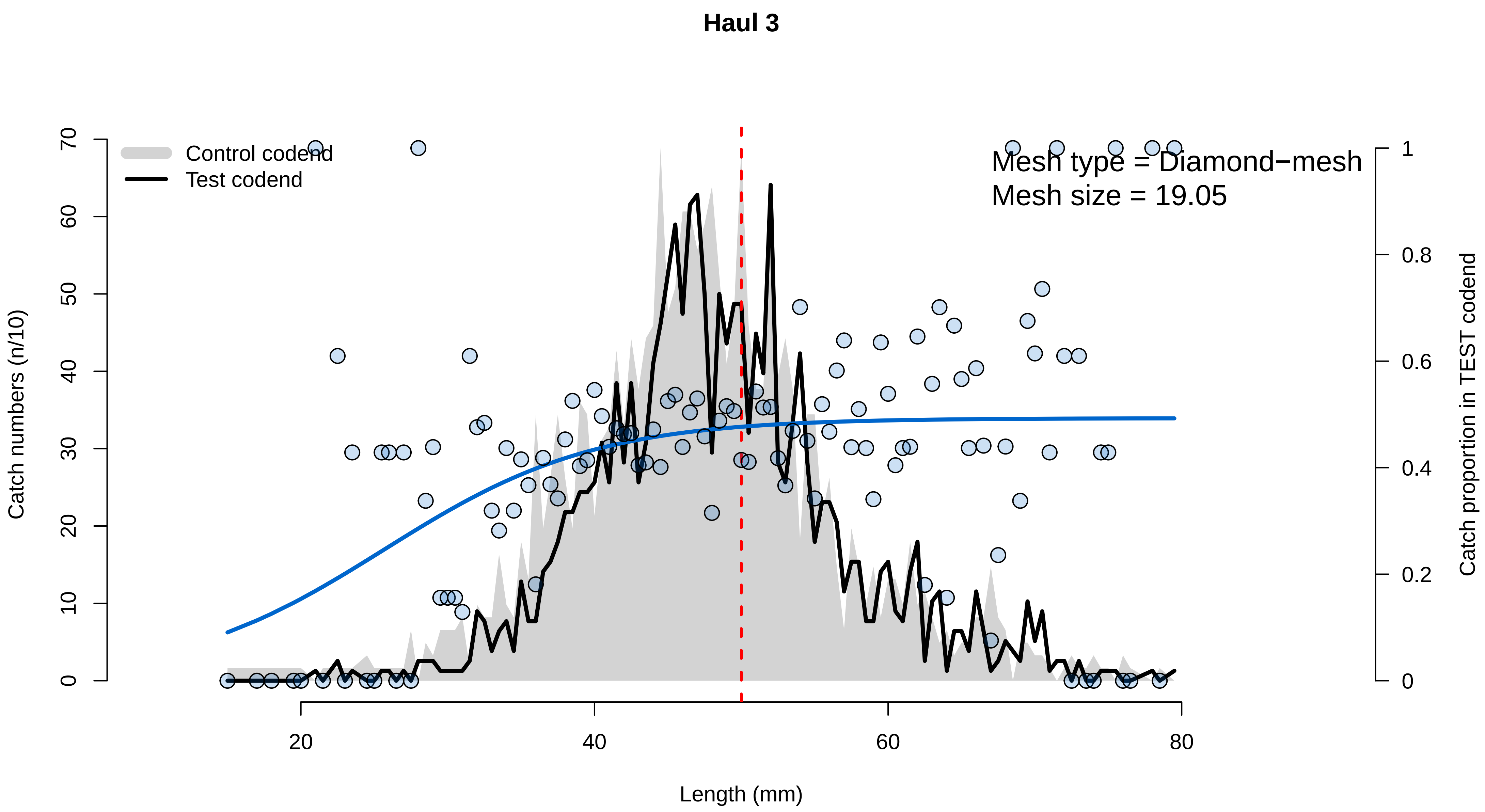

Haul 3
Control codend
Test codend
Mesh type = Diamond−mesh
Mesh size = 19.05
Catch numbers (n/10)
0
10
20
30
40
50
60
70
Catch proportion in TEST codend
0
0.2
0.4
0.6
0.8
1
20
40
60
80
Length (mm)

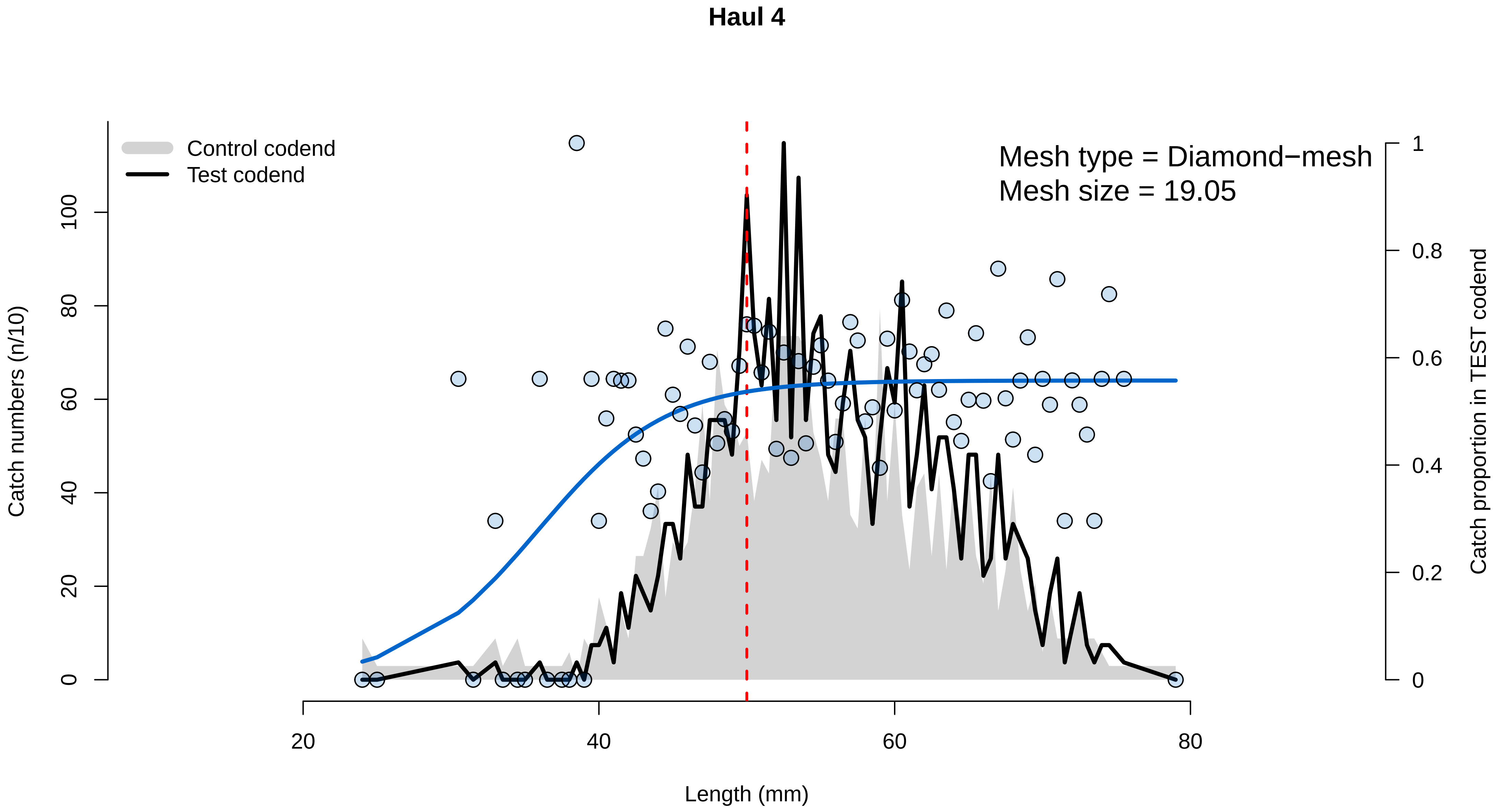
Haul 4
Control codend
Test codend
Mesh type = Diamond−mesh
Mesh size = 19.05
Catch numbers (n/10)
0
20
40
60
80
100
Catch proportion in TEST codend
0
0.2
0.4
0.6
0.8
1
20
40
60
80
Length (mm)

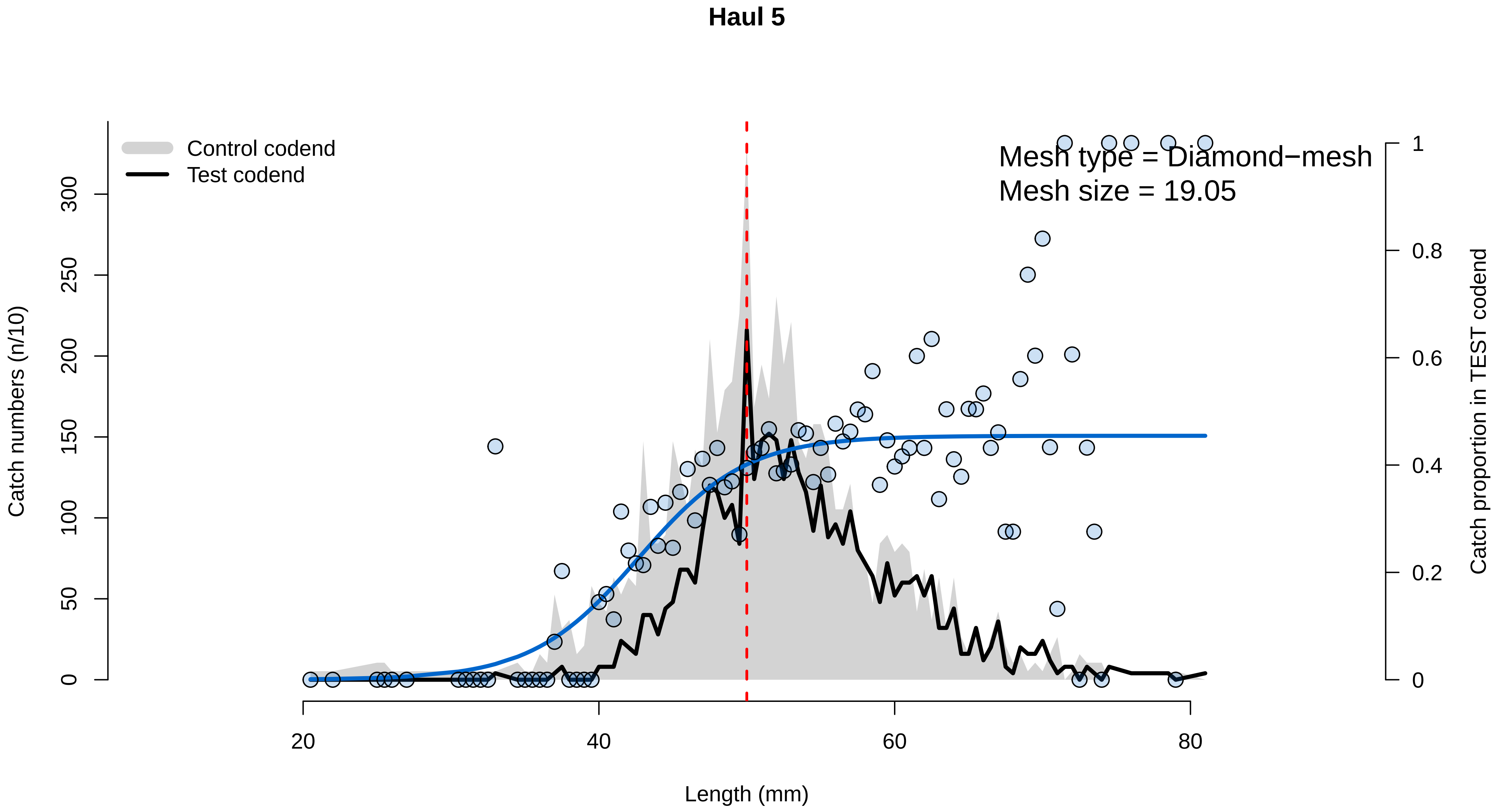
Haul 5
Control codend
Test codend
Mesh type = Diamond−mesh
Mesh size = 19.05
Catch numbers (n/10)
0
50
100
150
200
250
300
20
40
60
80
Length (mm)
Catch proportion in TEST codend
0
0.2
0.4
0.6
0.8
1

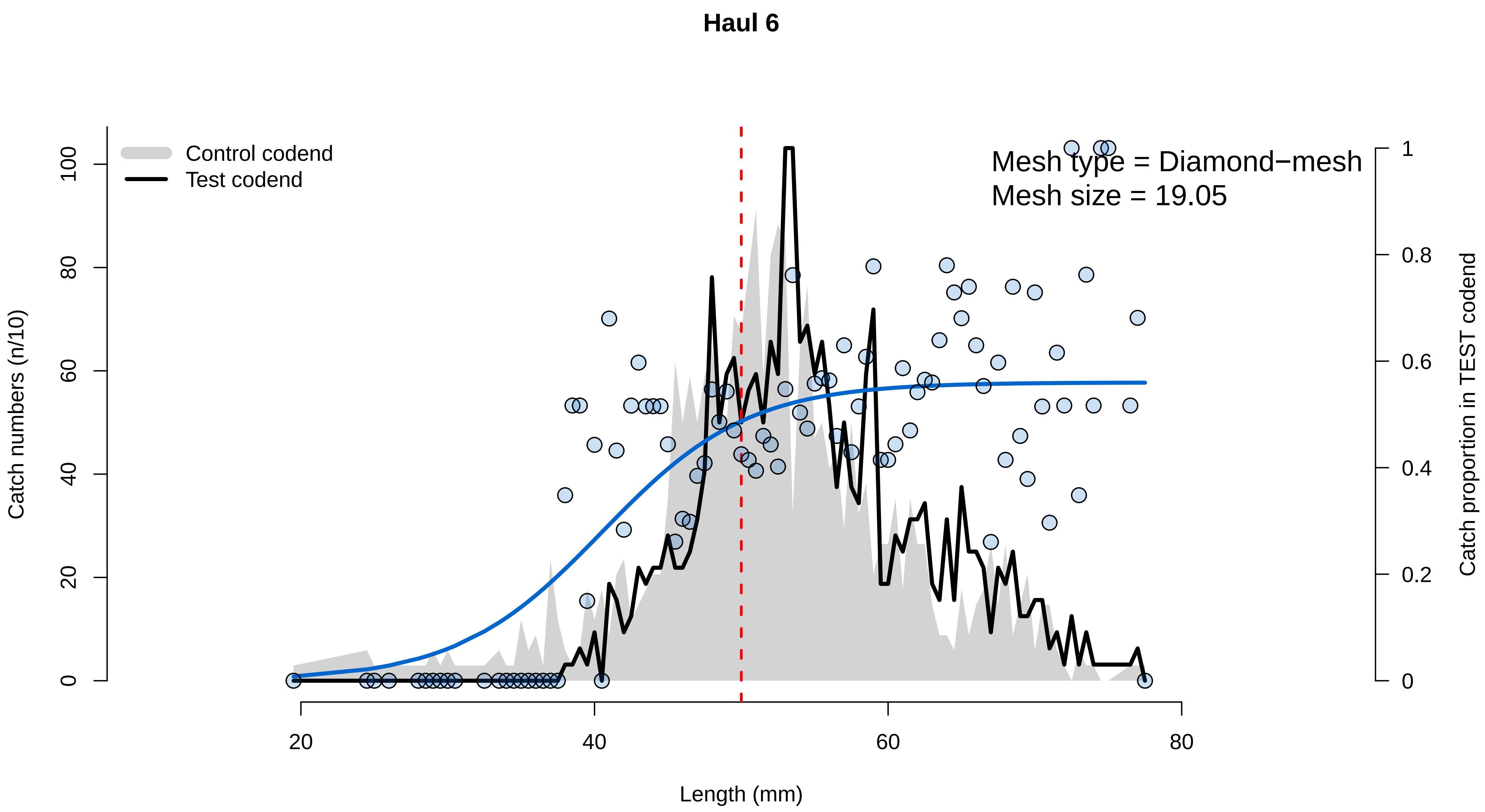
Haul 6
Control codend
Test codend
Mesh type = Diamond−mesh
Mesh size = 19.05
Catch numbers (n/10)
0
20
40
60
80
100
Catch proportion in TEST codend
0
0.2
0.4
0.6
0.8
1
Length (mm)
20
40
60
80

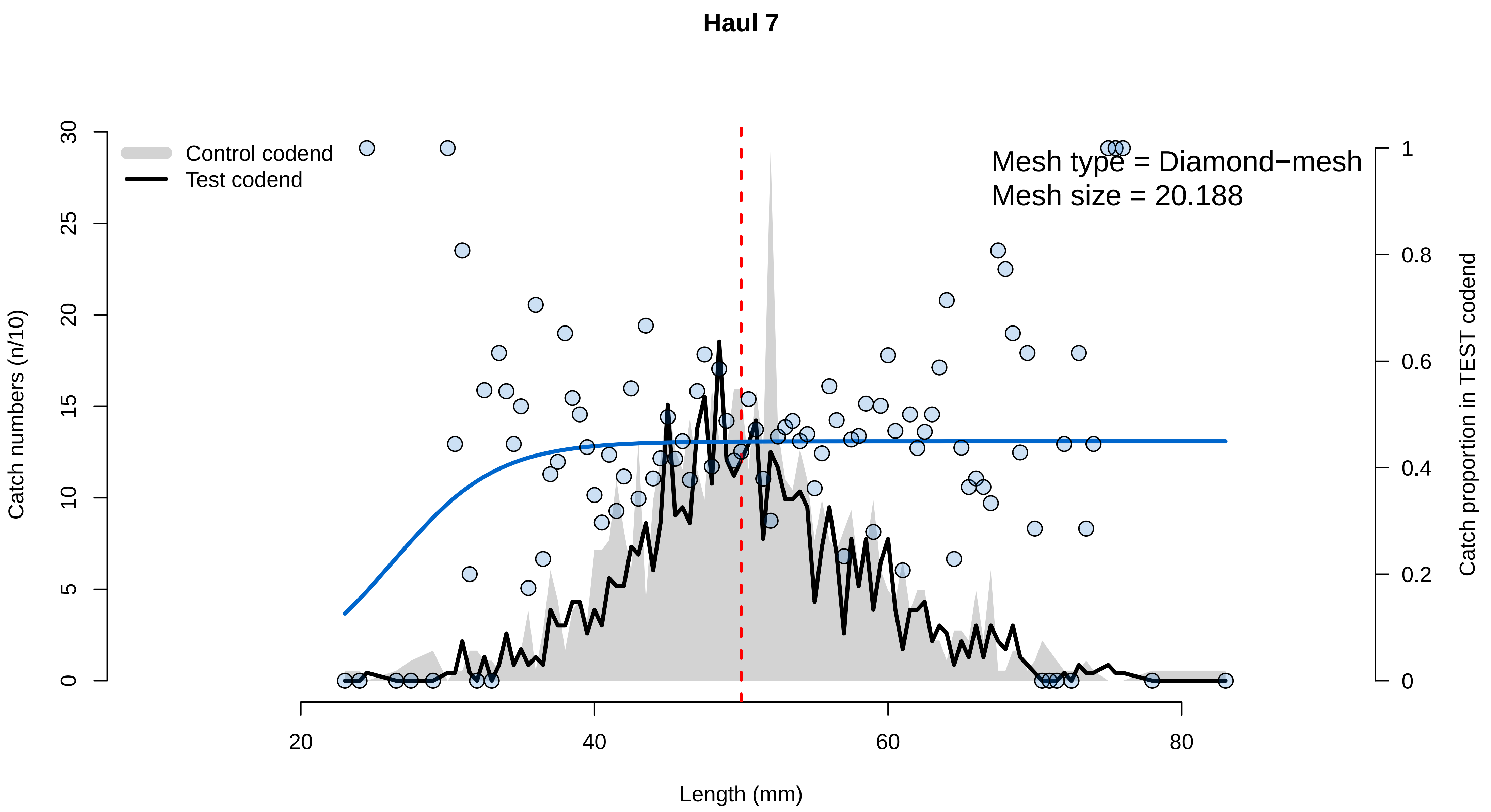
Haul 7
Control codend
Test codend
Mesh type = Diamond−mesh
Mesh size = 20.188
Catch numbers (n/10)
Catch proportion in TEST codend
Length (mm)
0
5
10
15
20
25
30
20
40
60
80
0
0.2
0.4
0.6
0.8
1

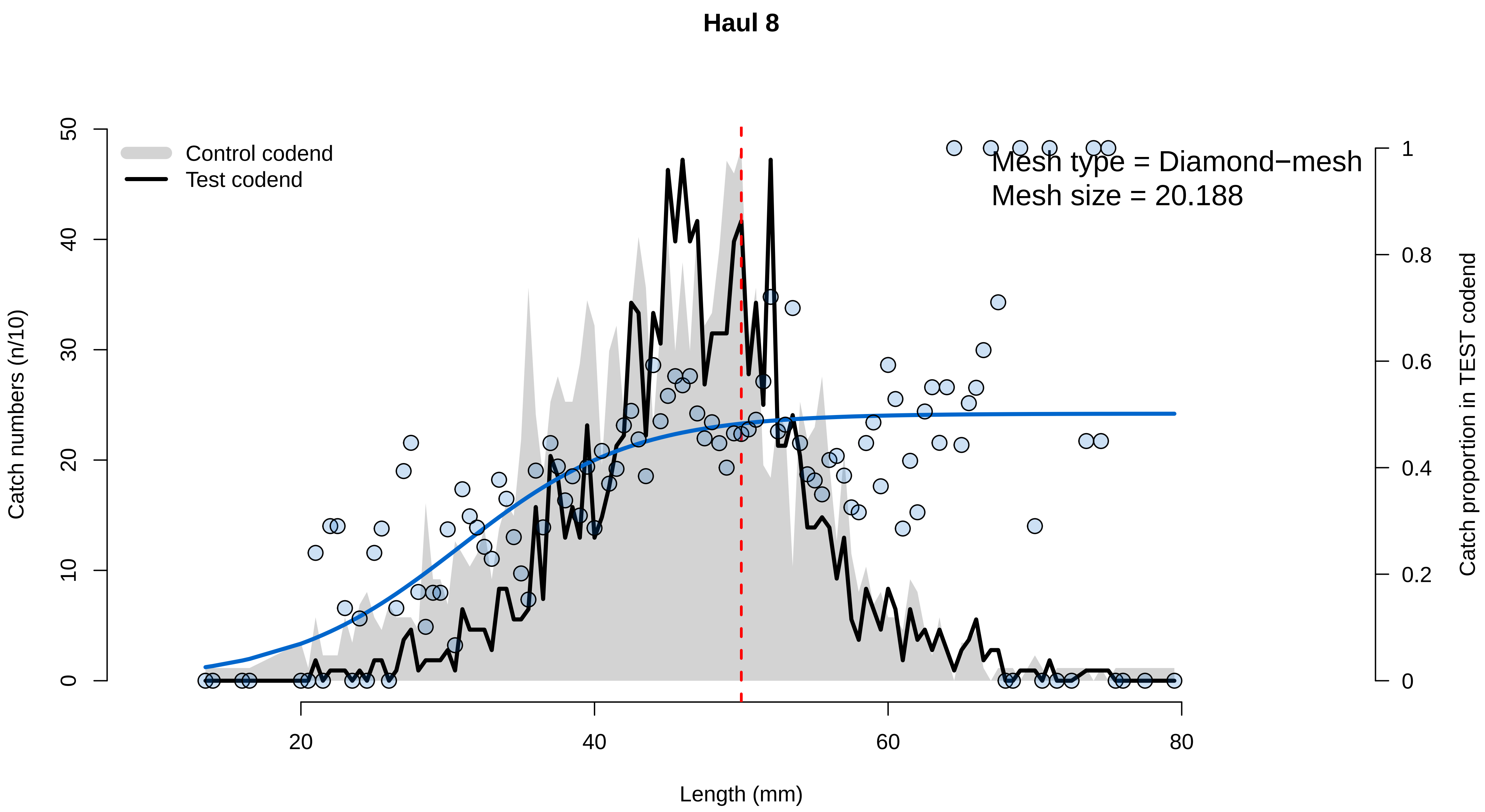

Haul 8
Control codend
Test codend
Mesh type = Diamond−mesh
Mesh size = 20.188
Catch numbers (n/10)
0
10
20
30
40
50
Catch proportion in TEST codend
0
0.2
0.4
0.6
0.8
1
20
40
60
80
Length (mm)

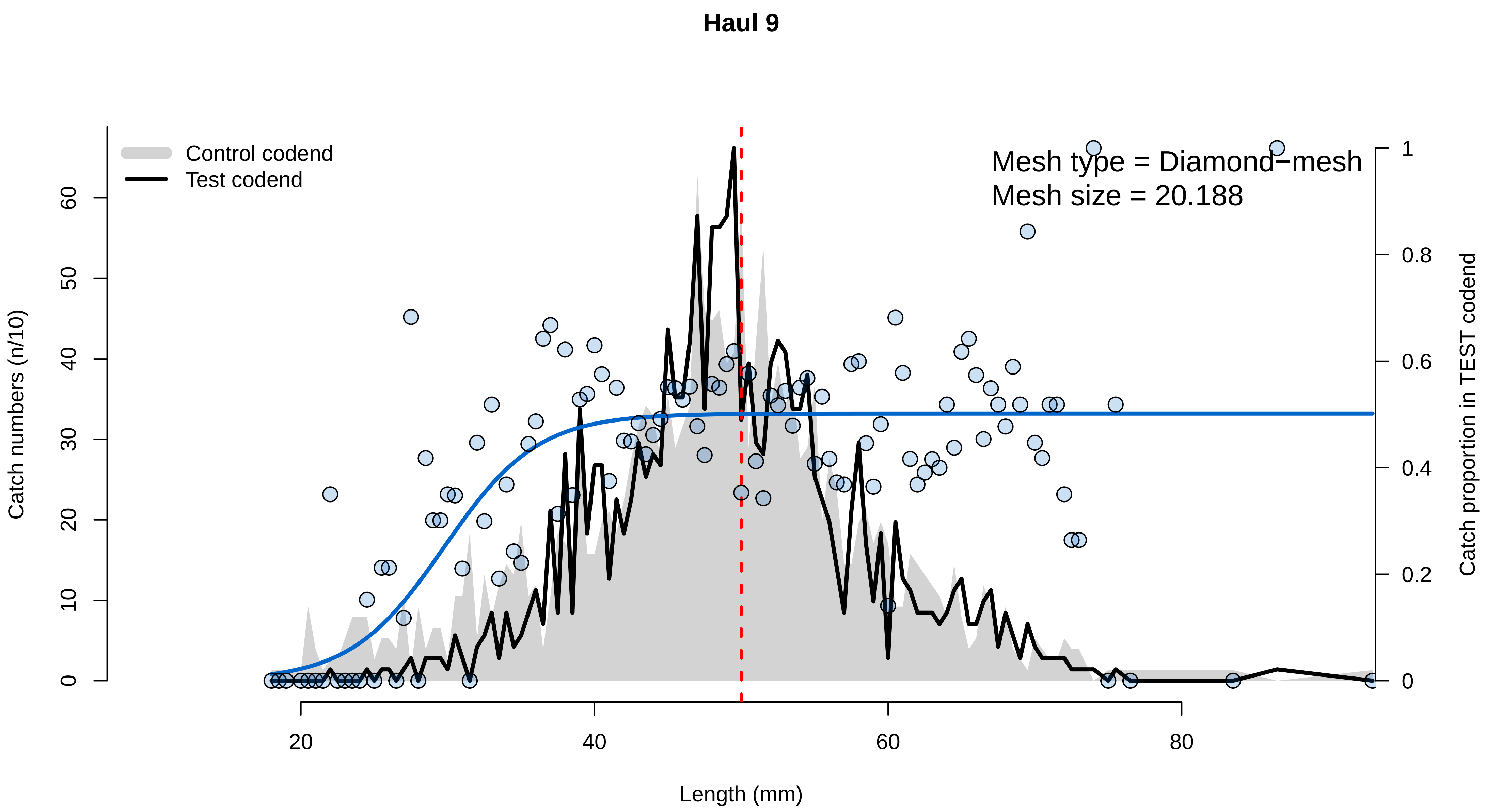

Haul 9
Control codend
Test codend
Mesh type = Diamond−mesh
Mesh size = 20.188
Catch numbers (n/10)
0
10
20
30
40
50
60
20
40
60
80
Length (mm)
Catch proportion in TEST codend
0
0.2
0.4
0.6
0.8
1

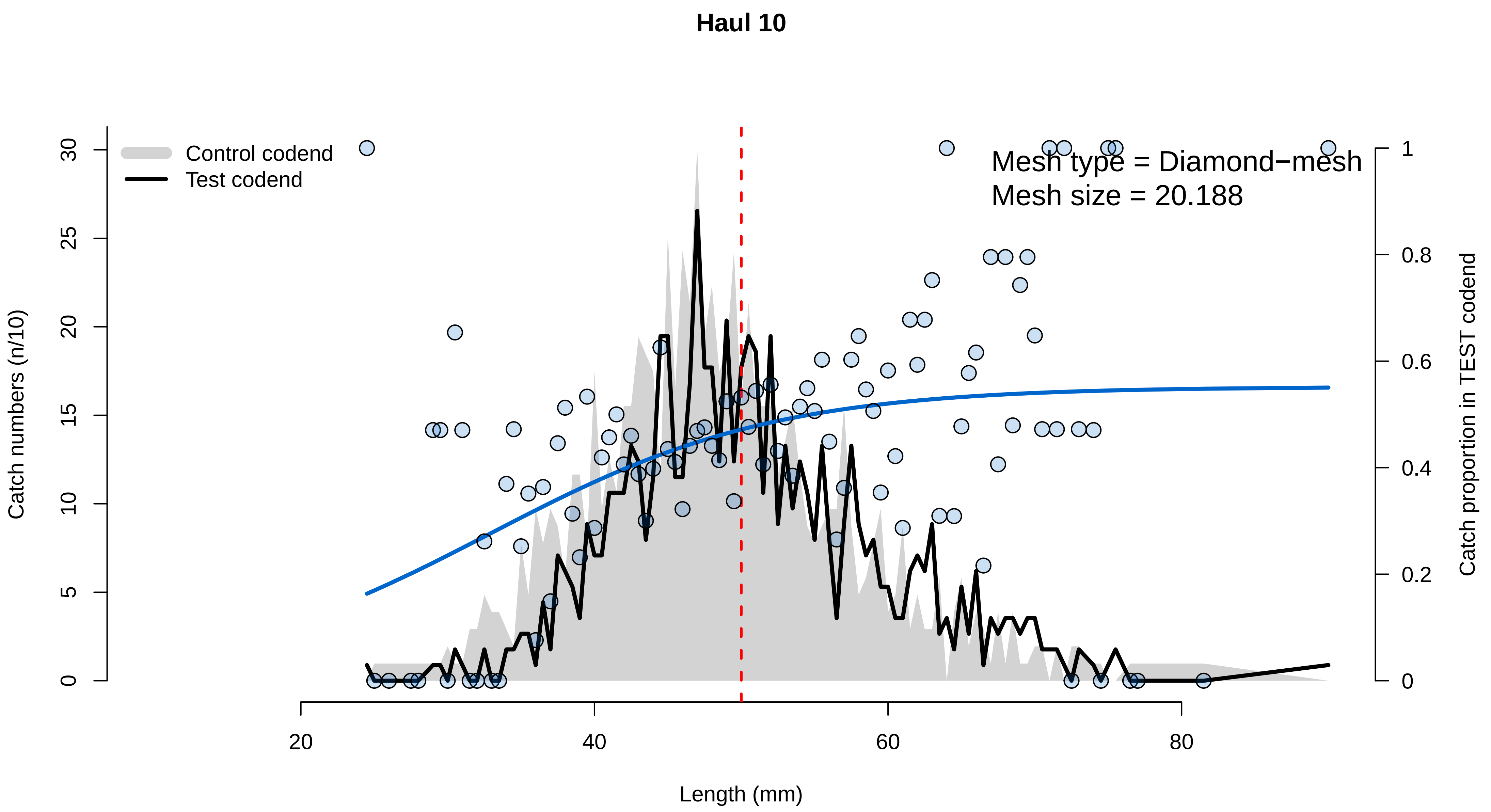

Haul 10
Control codend
Test codend
Mesh type = Diamond−mesh
Mesh size = 20.188
Catch numbers (n/10)
Catch proportion in TEST codend
Length (mm)
0
5
10
15
20
25
30
20
40
60
80
0
0.2
0.4
0.6
0.8
1

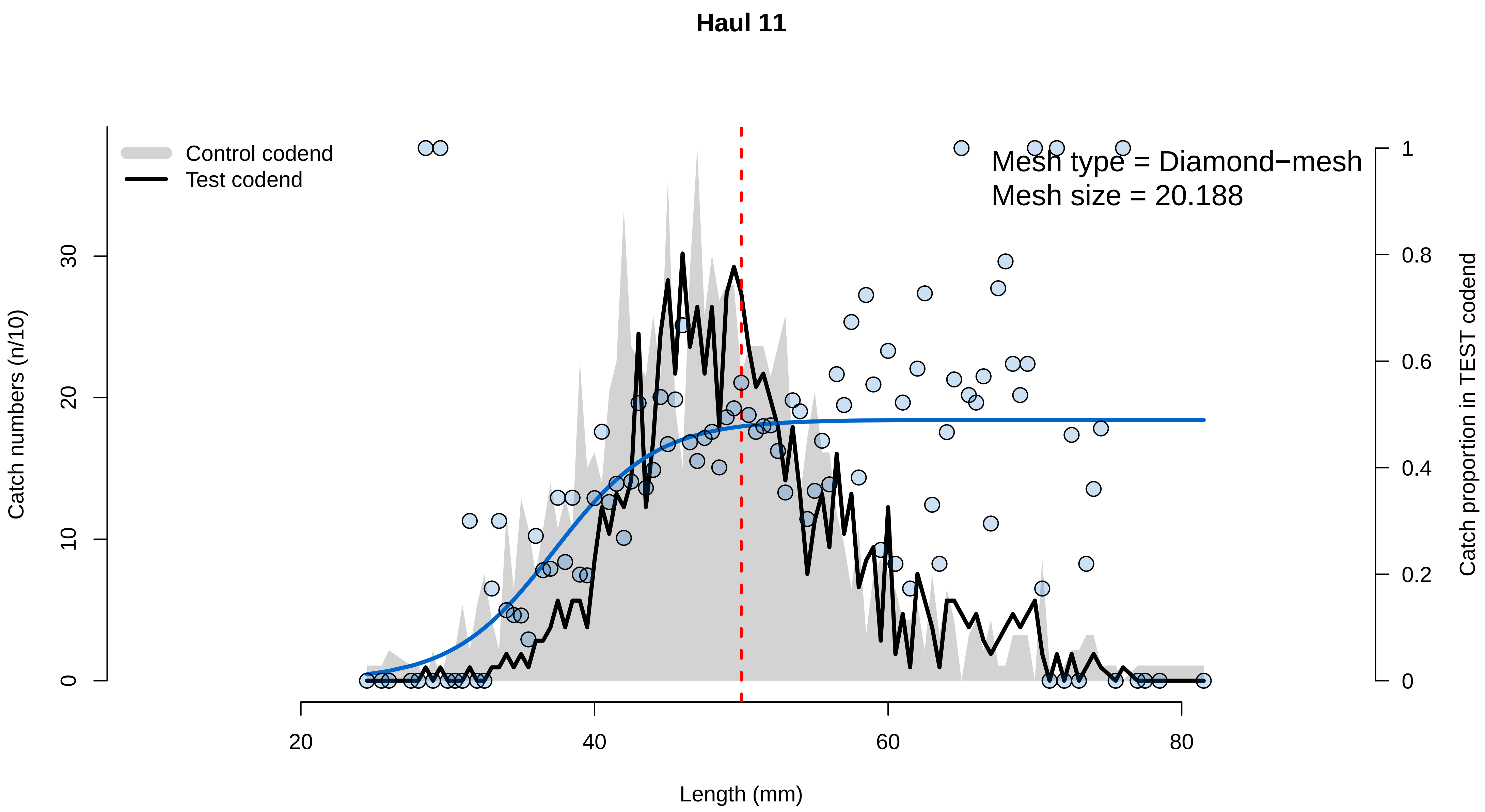

Haul 11
Control codend
Test codend
Mesh type = Diamond−mesh
Mesh size = 20.188
Catch numbers (n/10)
0
10
20
30
Catch proportion in TEST codend
0
0.2
0.4
0.6
0.8
1
20
40
60
80
Length (mm)

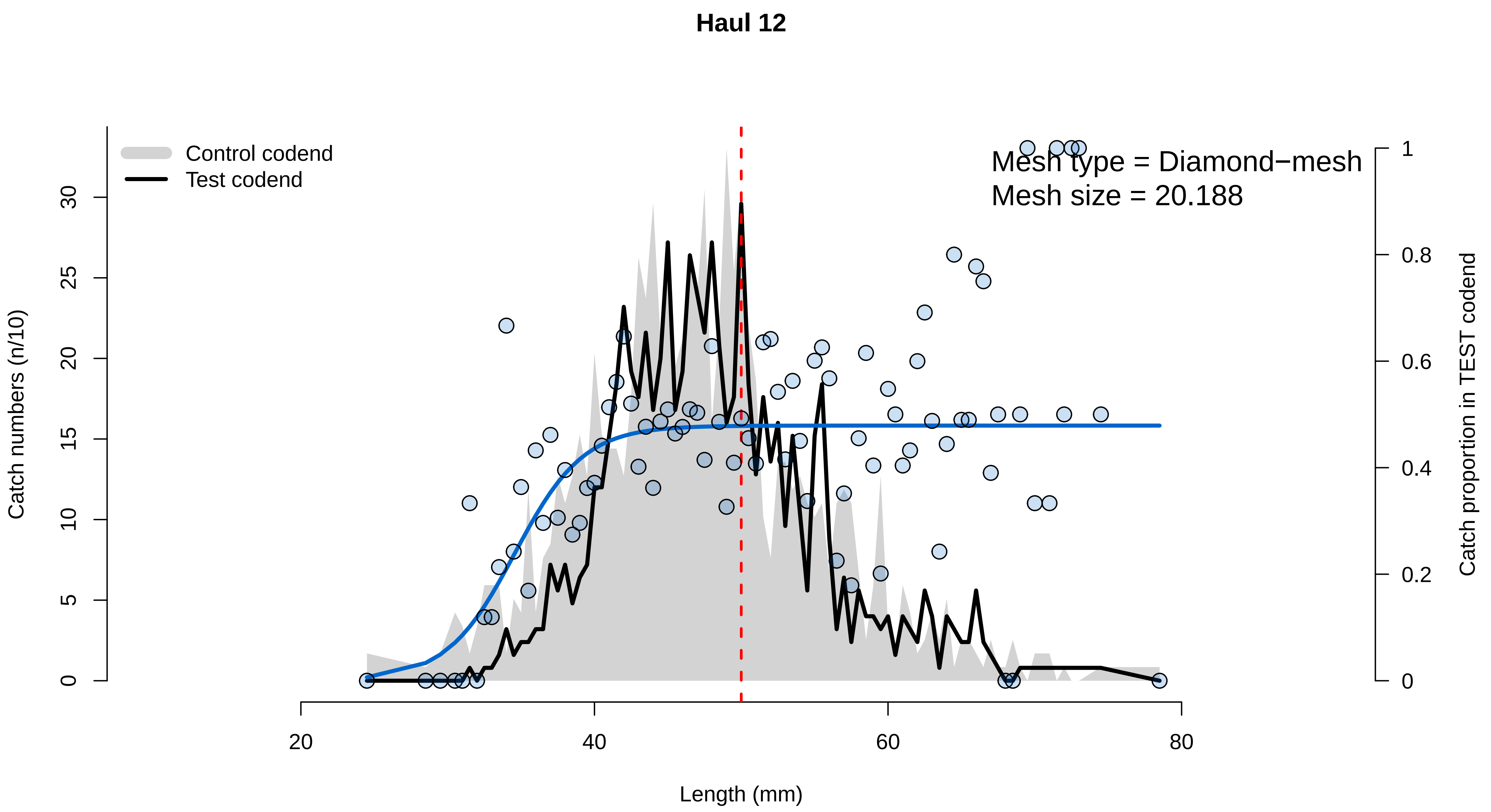

Haul 12
Control codend
Test codend
Mesh type = Diamond−mesh
Mesh size = 20.188
Catch numbers (n/10)
0
5
10
15
20
25
30
Catch proportion in TEST codend
0
0.2
0.4
0.6
0.8
1
Length (mm)
20
40
60
80

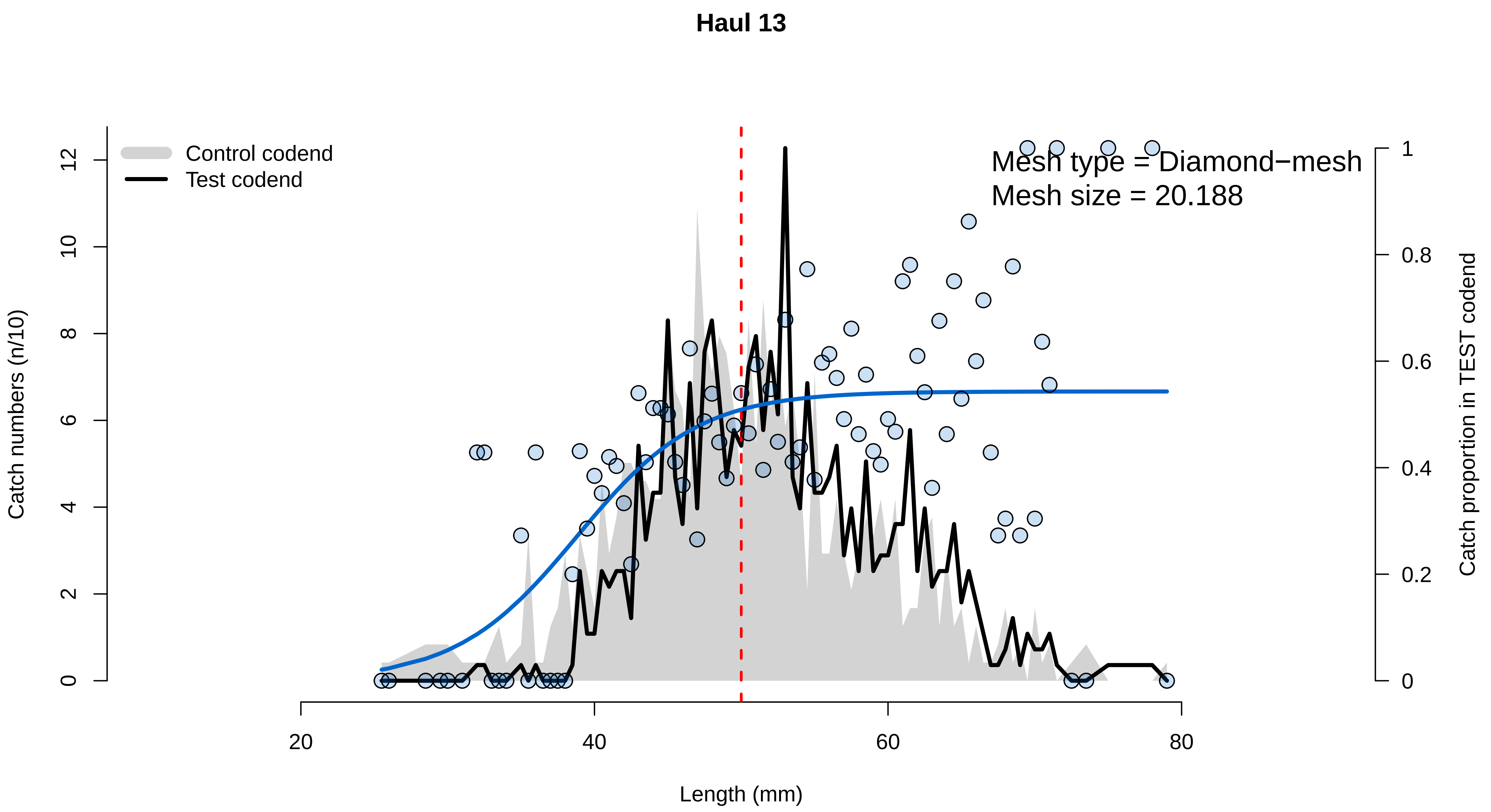

Haul 13
Control codend
Test codend
Mesh type = Diamond−mesh
Mesh size = 20.188
Catch numbers (n/10)
0
2
4
6
8
10
12
Catch proportion in TEST codend
0
0.2
0.4
0.6
0.8
1
20
40
60
80
Length (mm)

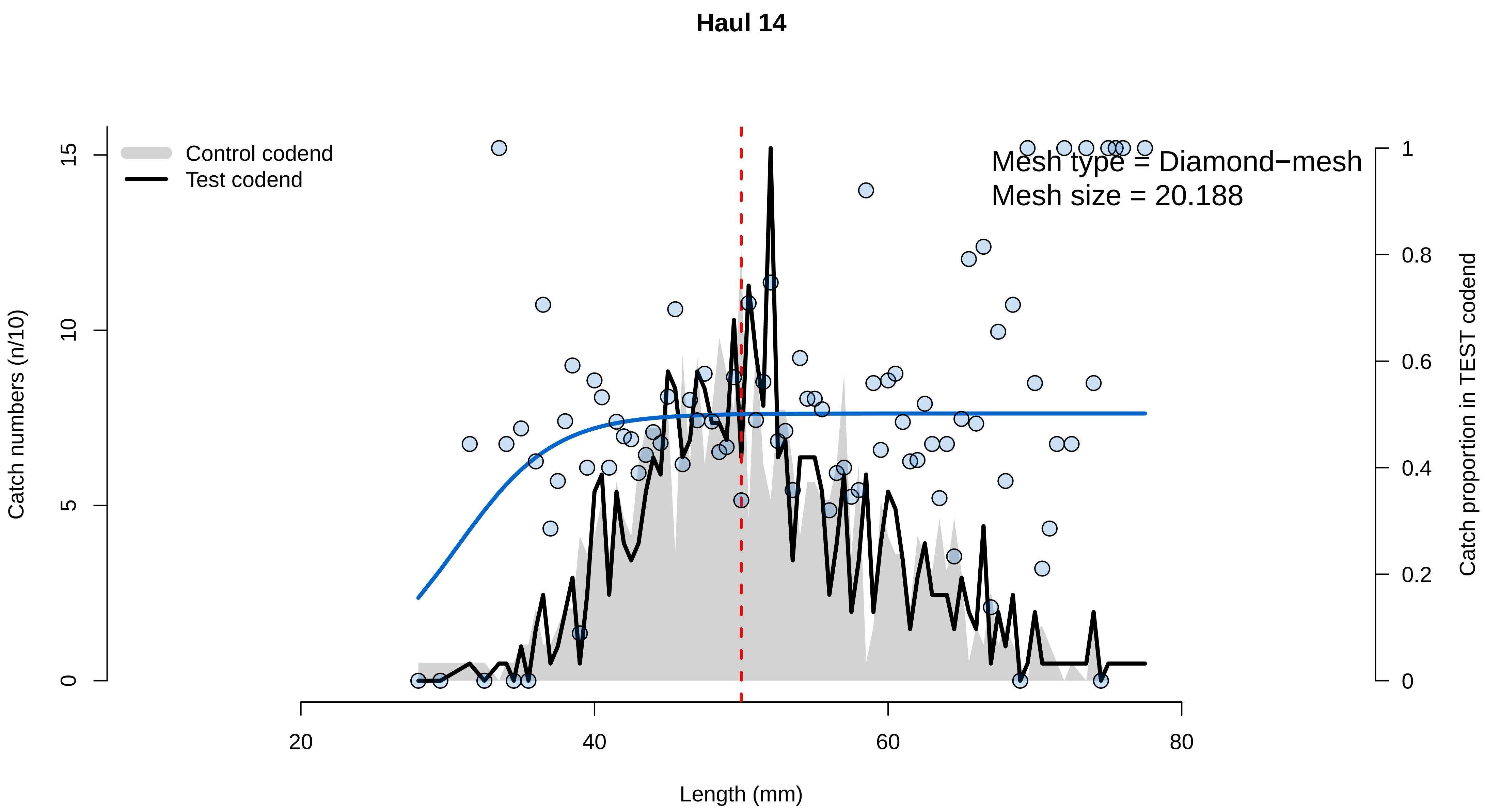

Haul 14
Control codend
Test codend
Mesh type = Diamond−mesh
Mesh size = 20.188
Catch numbers (n/10)
Catch proportion in TEST codend
Length (mm)
0
5
10
15
0
0.2
0.4
0.6
0.8
1
20
40
60
80

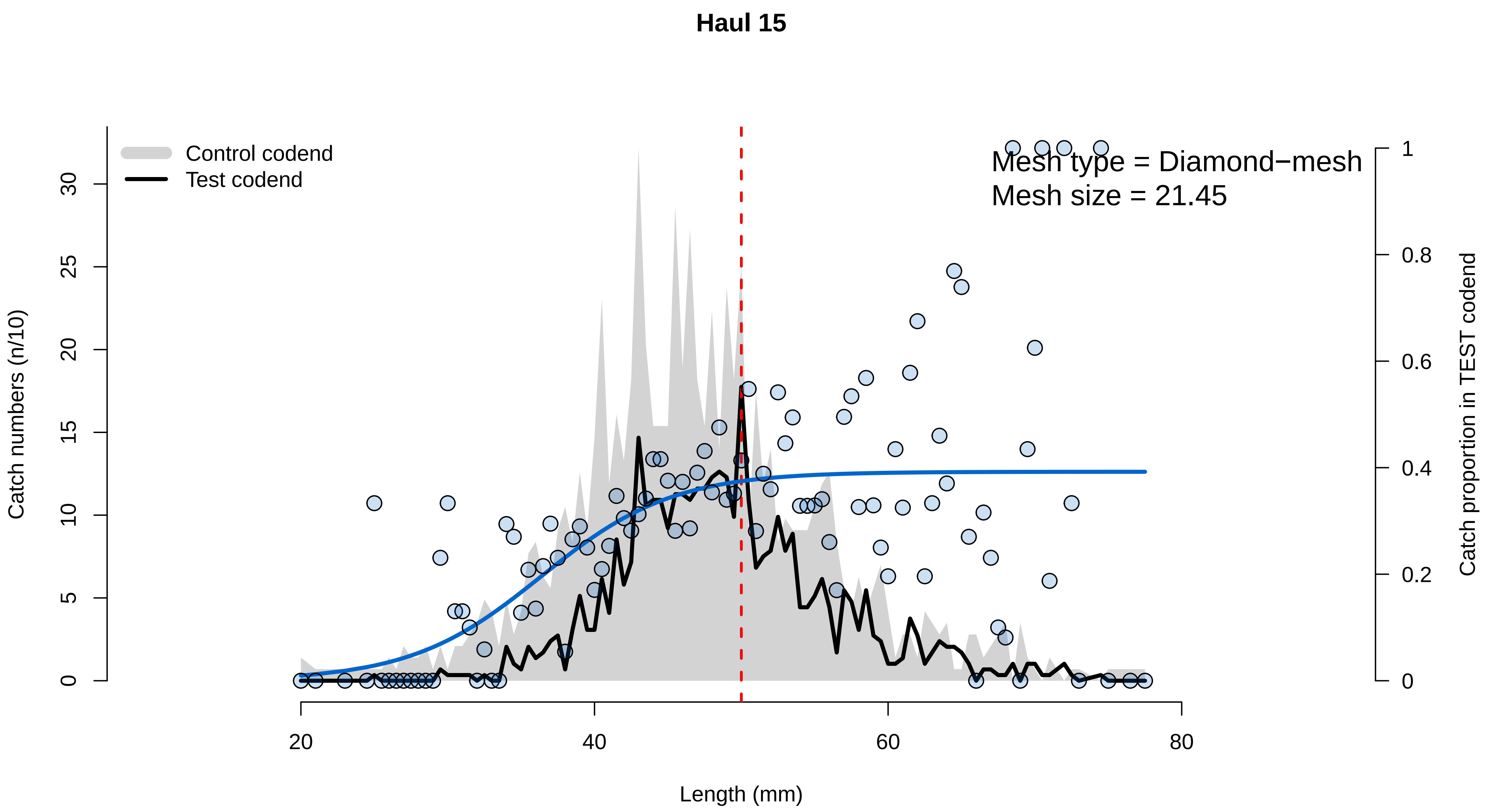

Haul 15
Control codend
Test codend
Mesh type = Diamond−mesh
Mesh size = 21.45
Catch numbers (n/10)
Catch proportion in TEST codend
Length (mm)
0
5
10
15
20
25
30
20
40
60
80
0
0.2
0.4
0.6
0.8
1

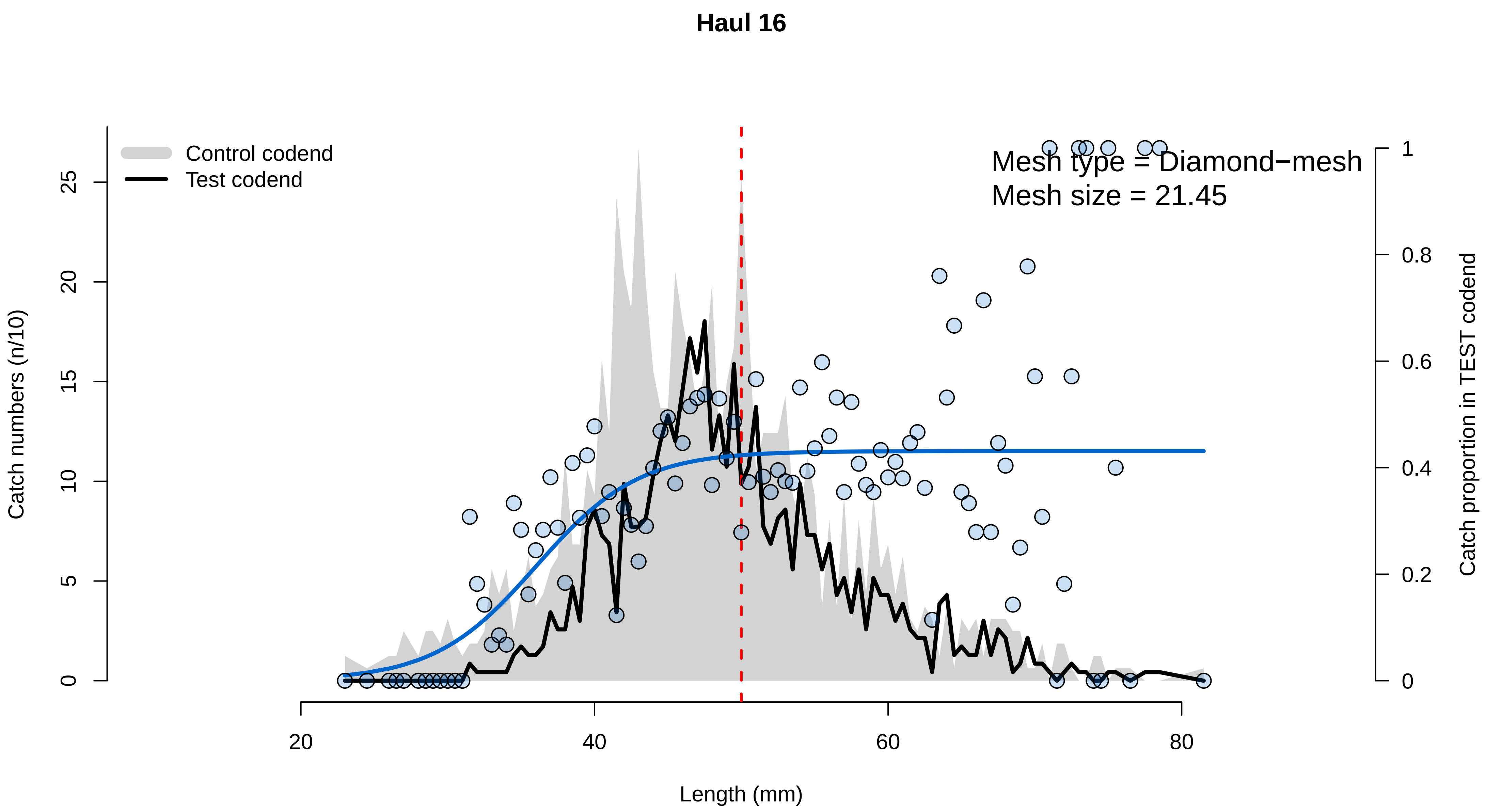
Haul 16
Control codend
Test codend
Mesh type = Diamond−mesh
Mesh size = 21.45
Catch numbers (n/10)
0
5
10
15
20
25
Catch proportion in TEST codend
0
0.2
0.4
0.6
0.8
1
20
40
60
80
Length (mm)

Haul 17

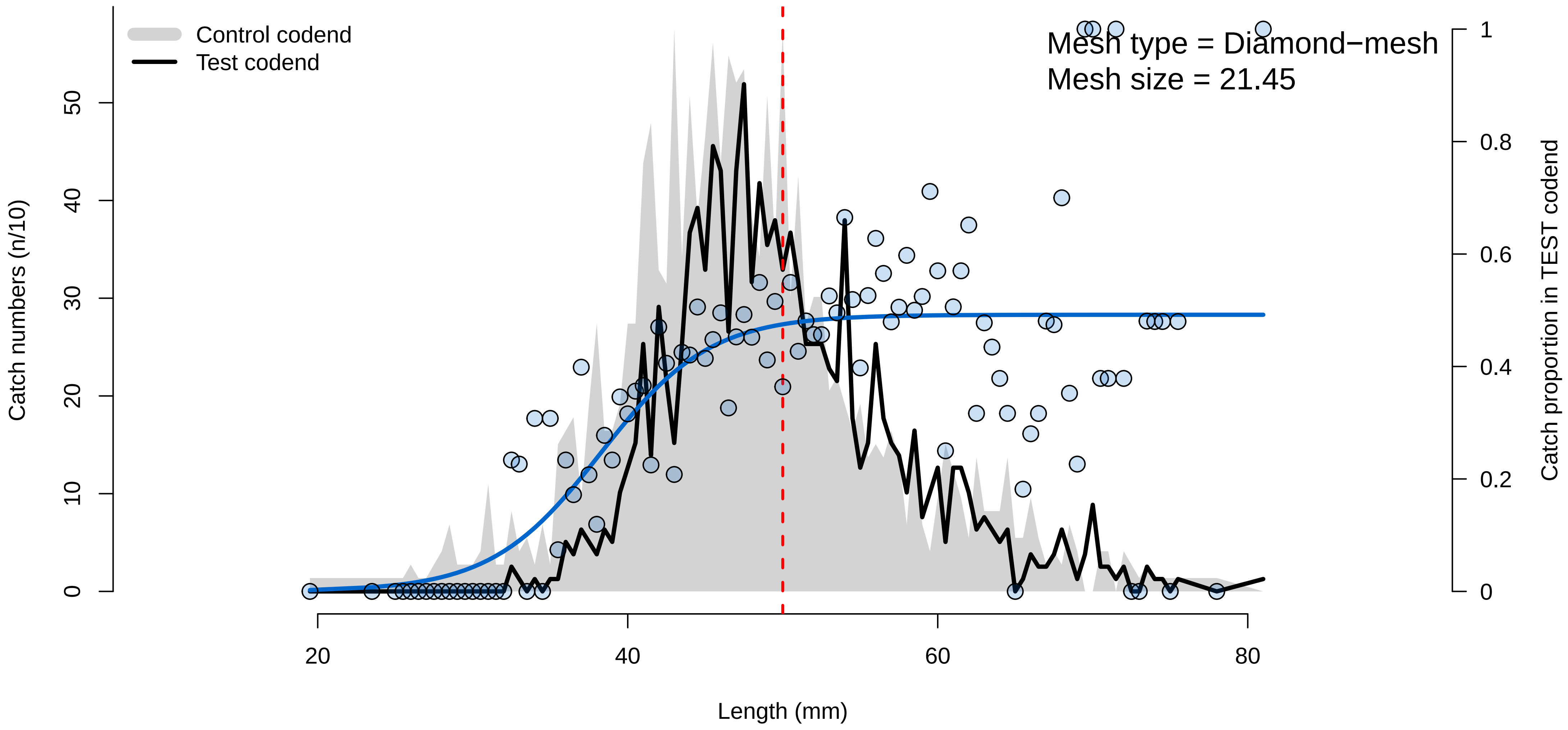

Control codend
Test codend
Mesh type = Diamond−mesh
Mesh size = 21.45
Catch numbers (n/10)
0
10
20
30
40
50
20
40
60
80
Length (mm)
0
0.2
0.4
0.6
0.8
1
Catch proportion in TEST codend

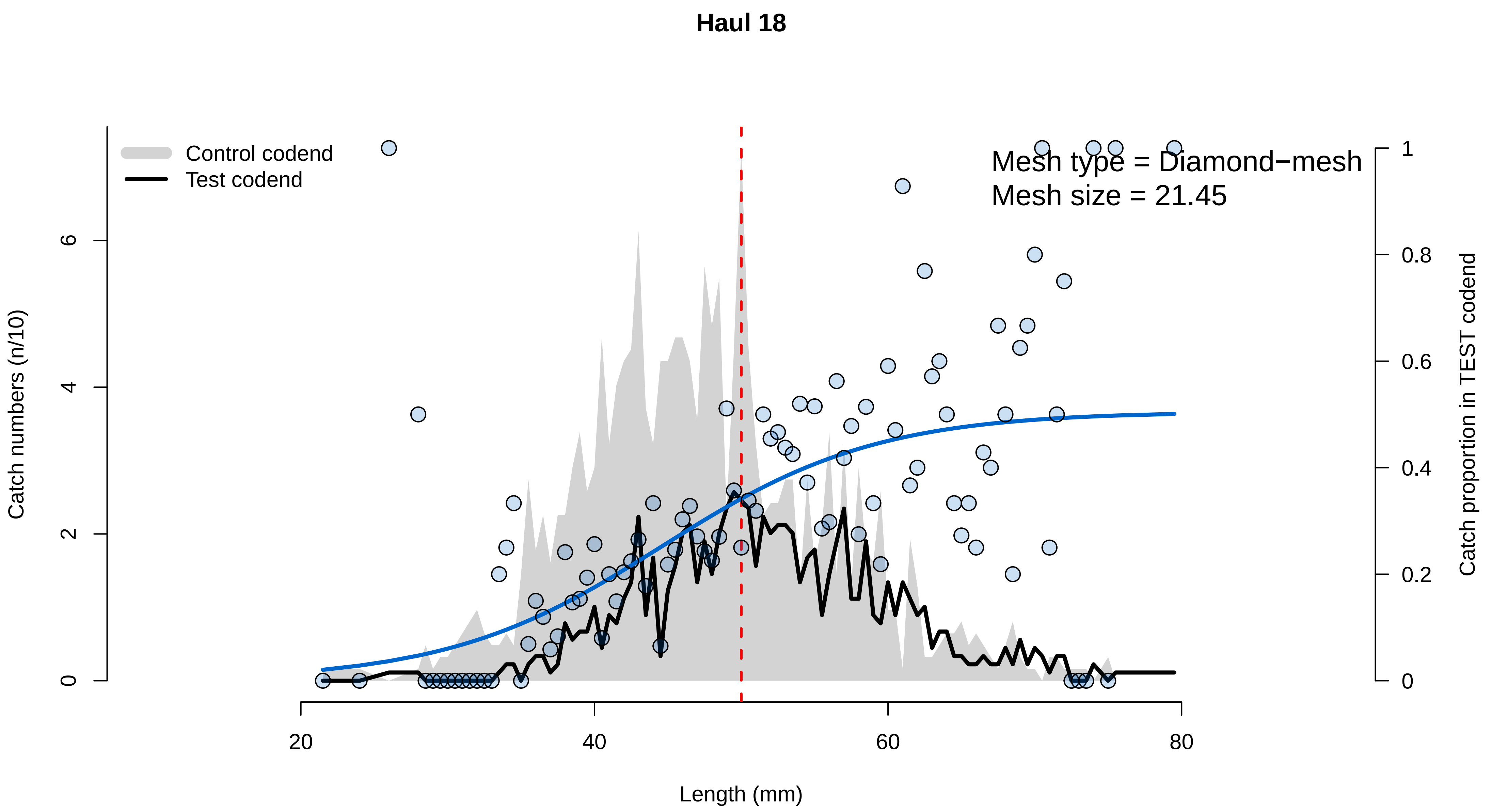

Haul 18
Control codend
Test codend
Mesh type = Diamond−mesh
Mesh size = 21.45
Catch numbers (n/10)
Catch proportion in TEST codend
Length (mm)
0
2
4
6
20
40
60
80
0
0.2
0.4
0.6
0.8
1

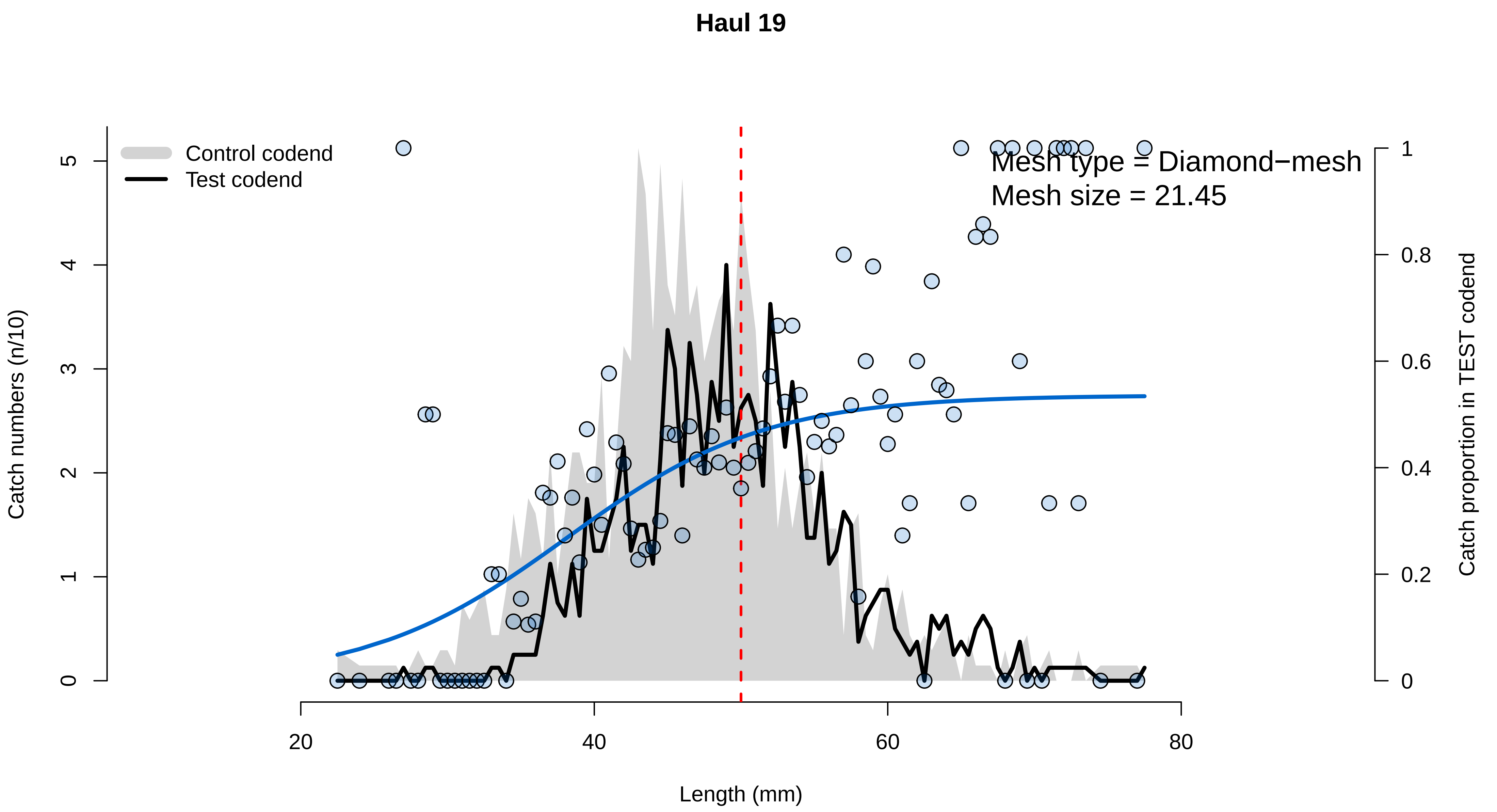
Haul 19
Control codend
Test codend
Mesh type = Diamond−mesh
Mesh size = 21.45
Catch numbers (n/10)
Catch proportion in TEST codend
Length (mm)
0
1
2
3
4
5
0
0.2
0.4
0.6
0.8
1
20
40
60
80

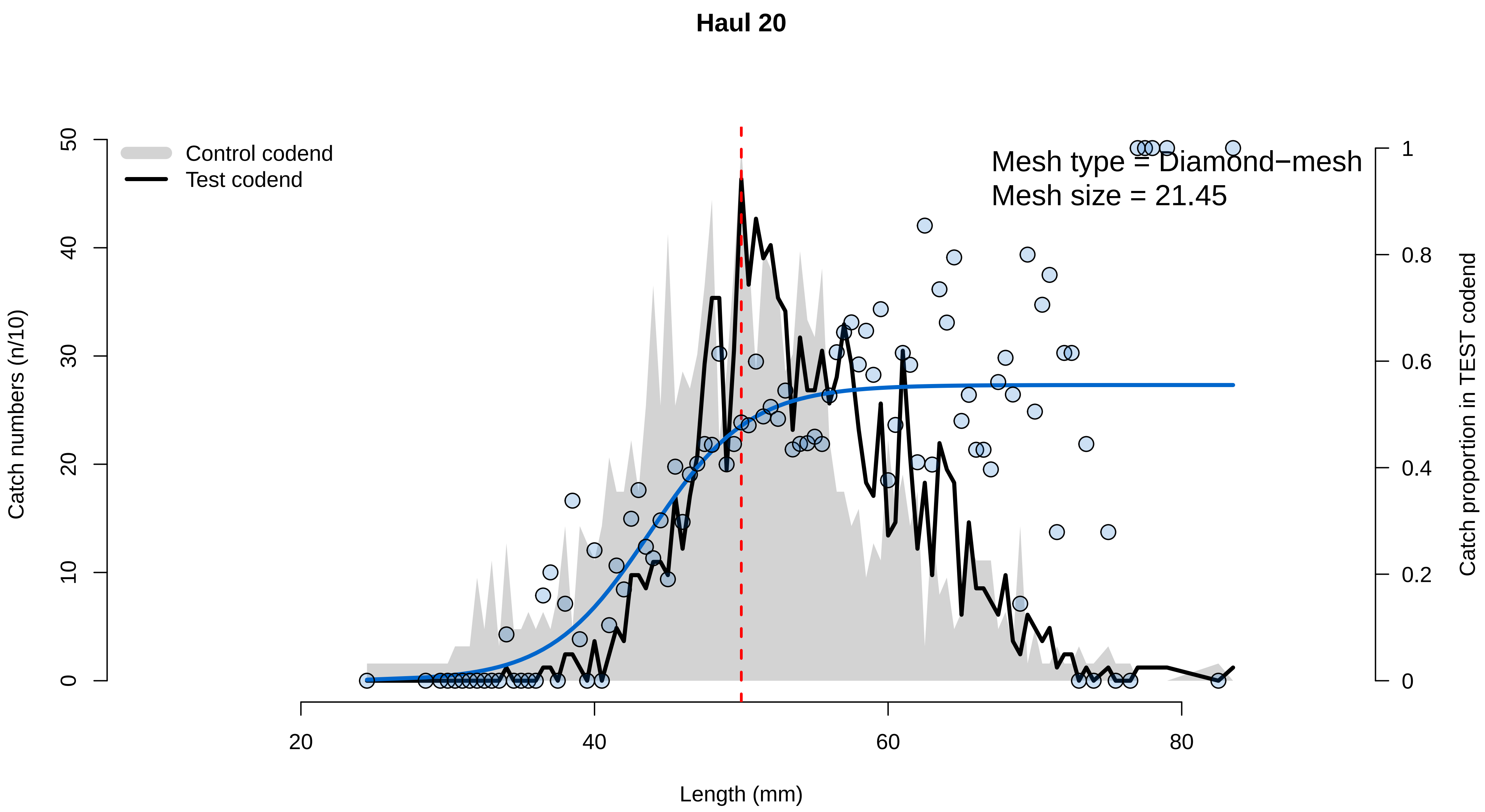

Haul 20
Control codend
Test codend
Mesh type = Diamond−mesh
Mesh size = 21.45
Catch numbers (n/10)
Catch proportion in TEST codend
Length (mm)
0
10
20
30
40
50
0
0.2
0.4
0.6
0.8
1
20
40
60
80

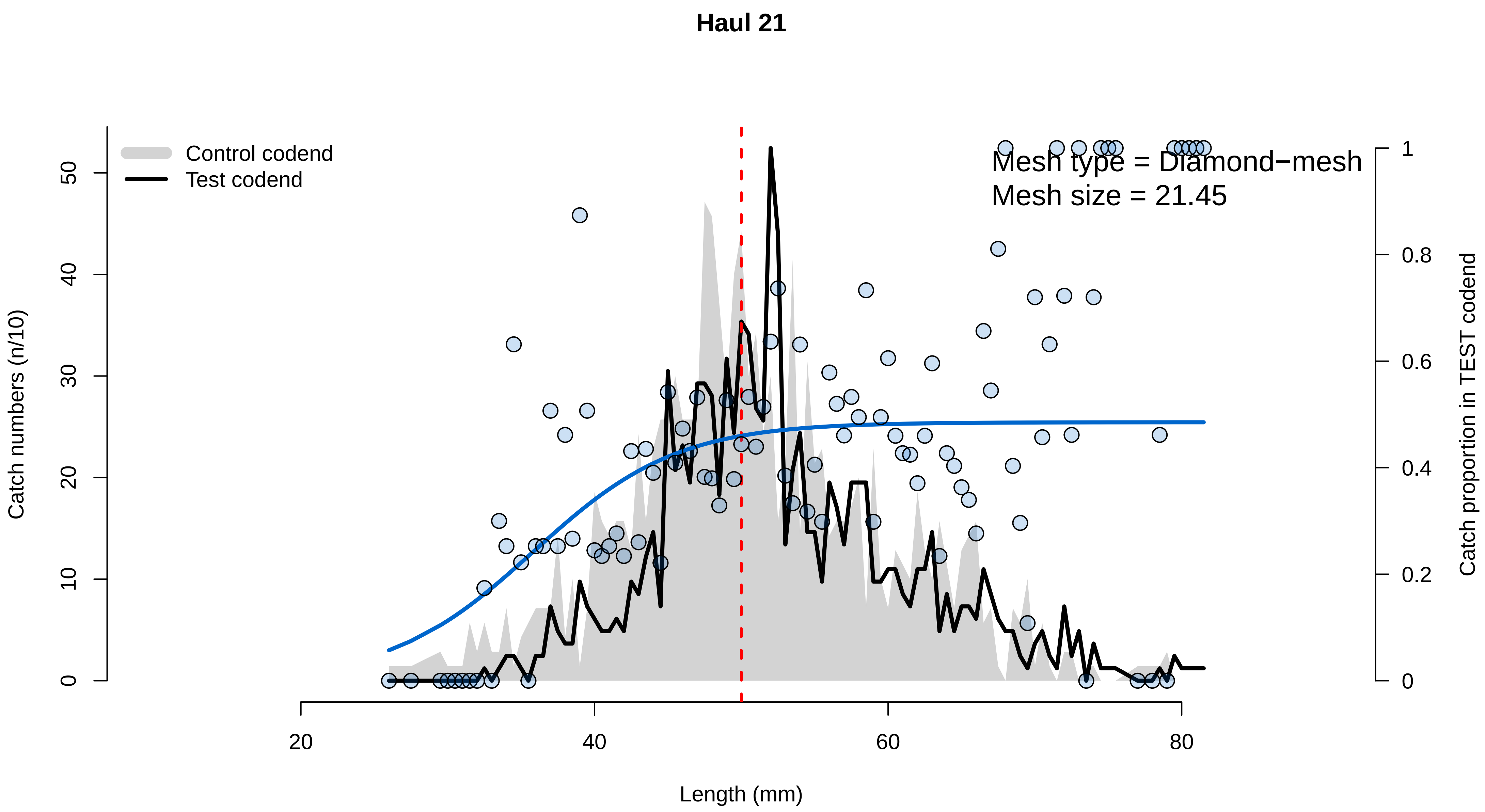

Haul 21
Control codend
Test codend
Mesh type = Diamond−mesh
Mesh size = 21.45
Catch numbers (n/10)
Catch proportion in TEST codend
Length (mm)
0
10
20
30
40
50
0
0.2
0.4
0.6
0.8
1
20
40
60
80

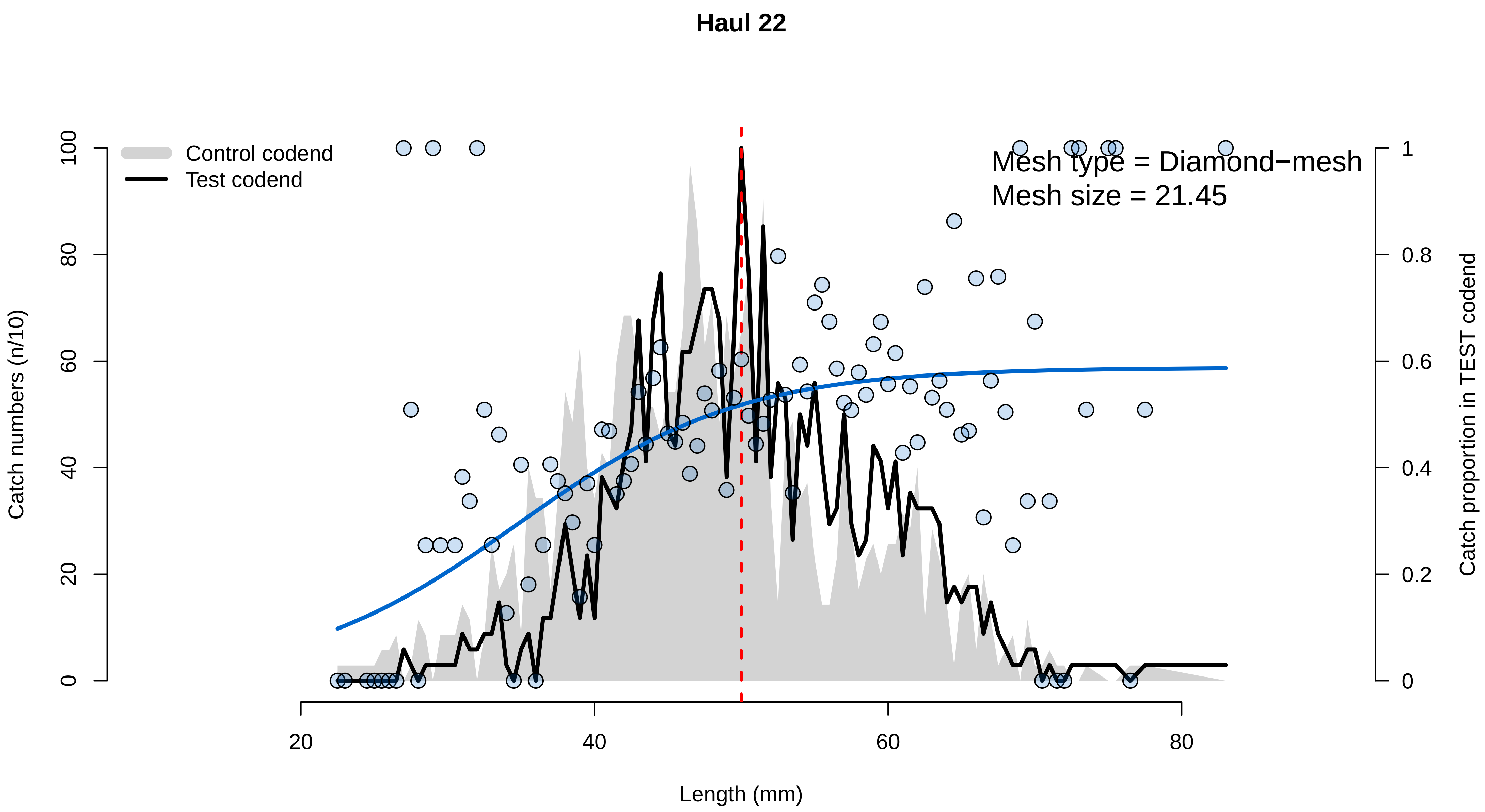

Haul 22
Control codend
Test codend
Mesh type = Diamond−mesh
Mesh size = 21.45
Catch numbers (n/10)
0
20
40
60
80
100
Catch proportion in TEST codend
0
0.2
0.4
0.6
0.8
1
20
40
60
80
Length (mm)

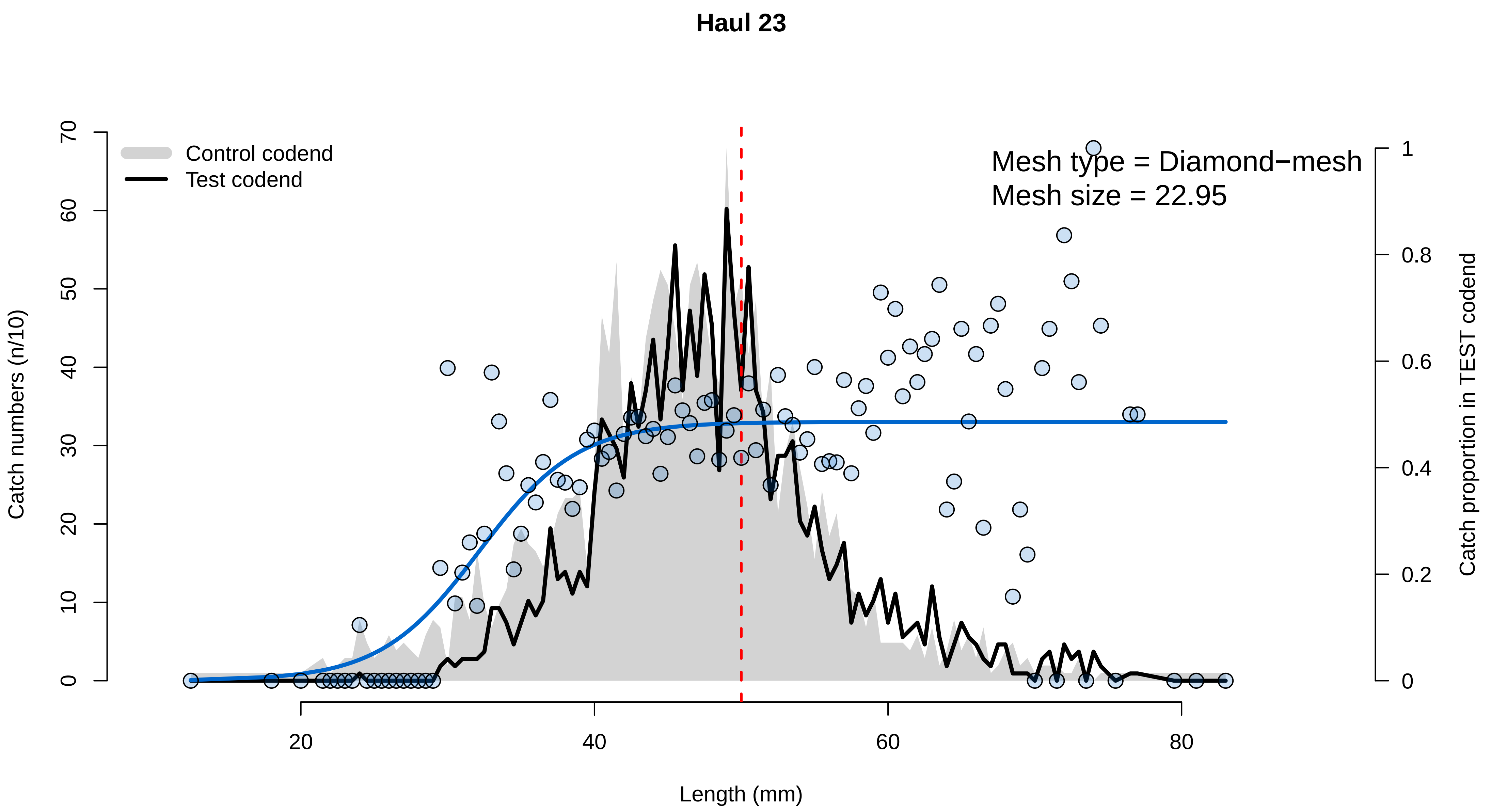

Haul 23
Control codend
Test codend
Mesh type = Diamond−mesh
Mesh size = 22.95
Catch numbers (n/10)
Catch proportion in TEST codend
Length (mm)
0
10
20
30
40
50
60
70
20
40
60
80
0
0.2
0.4
0.6
0.8
1

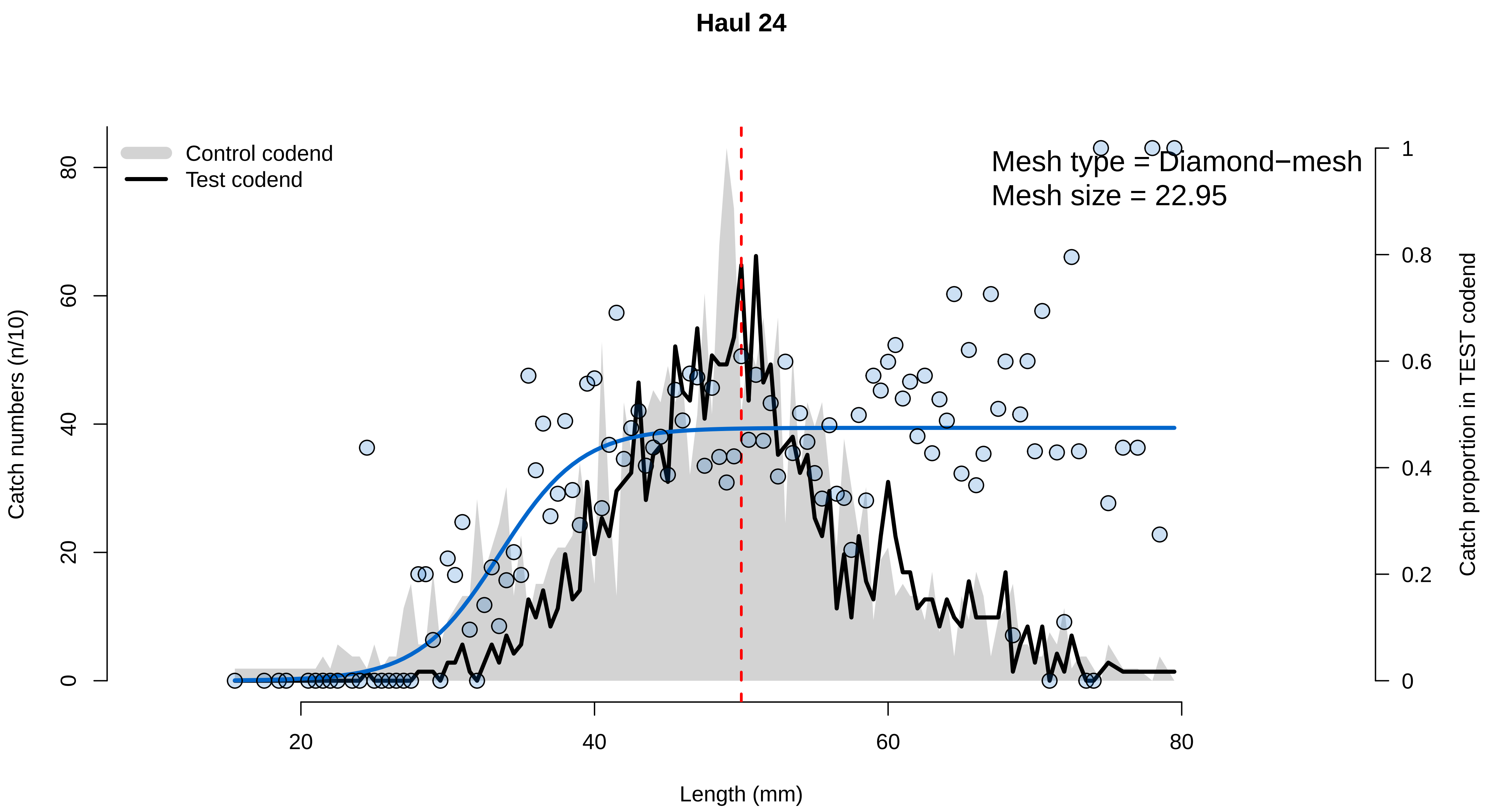

Haul 24
Control codend
Test codend
Mesh type = Diamond−mesh
Mesh size = 22.95
Catch numbers (n/10)
Catch proportion in TEST codend
Length (mm)
0
20
40
60
80
0.2
0.4
0.6
0.8
1

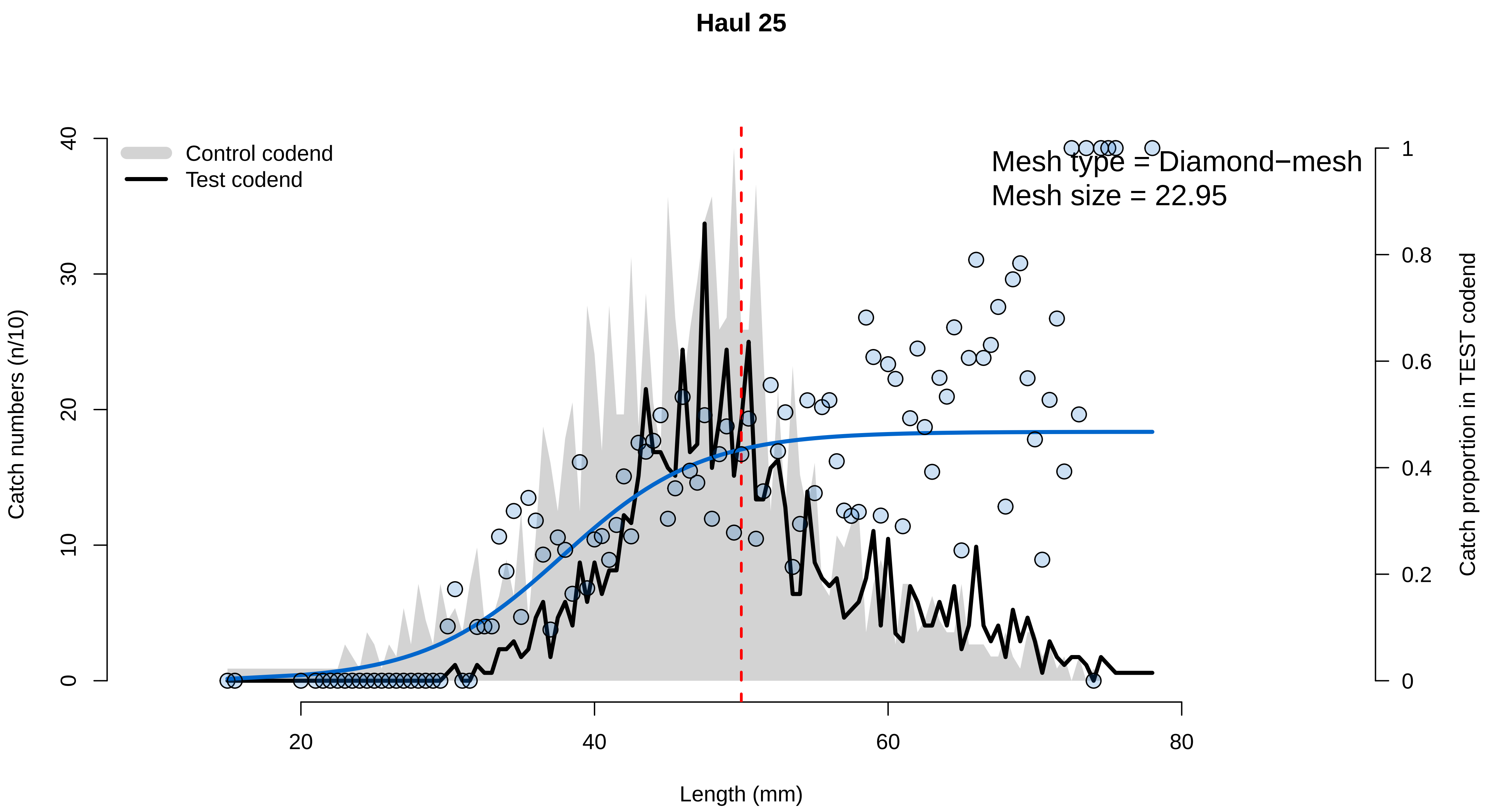

Haul 25
Control codend
Test codend
Mesh type = Diamond−mesh
Mesh size = 22.95
Catch numbers (n/10)
0
10
20
30
40
Catch proportion in TEST codend
0
0.2
0.4
0.6
0.8
1
20
40
60
80
Length (mm)

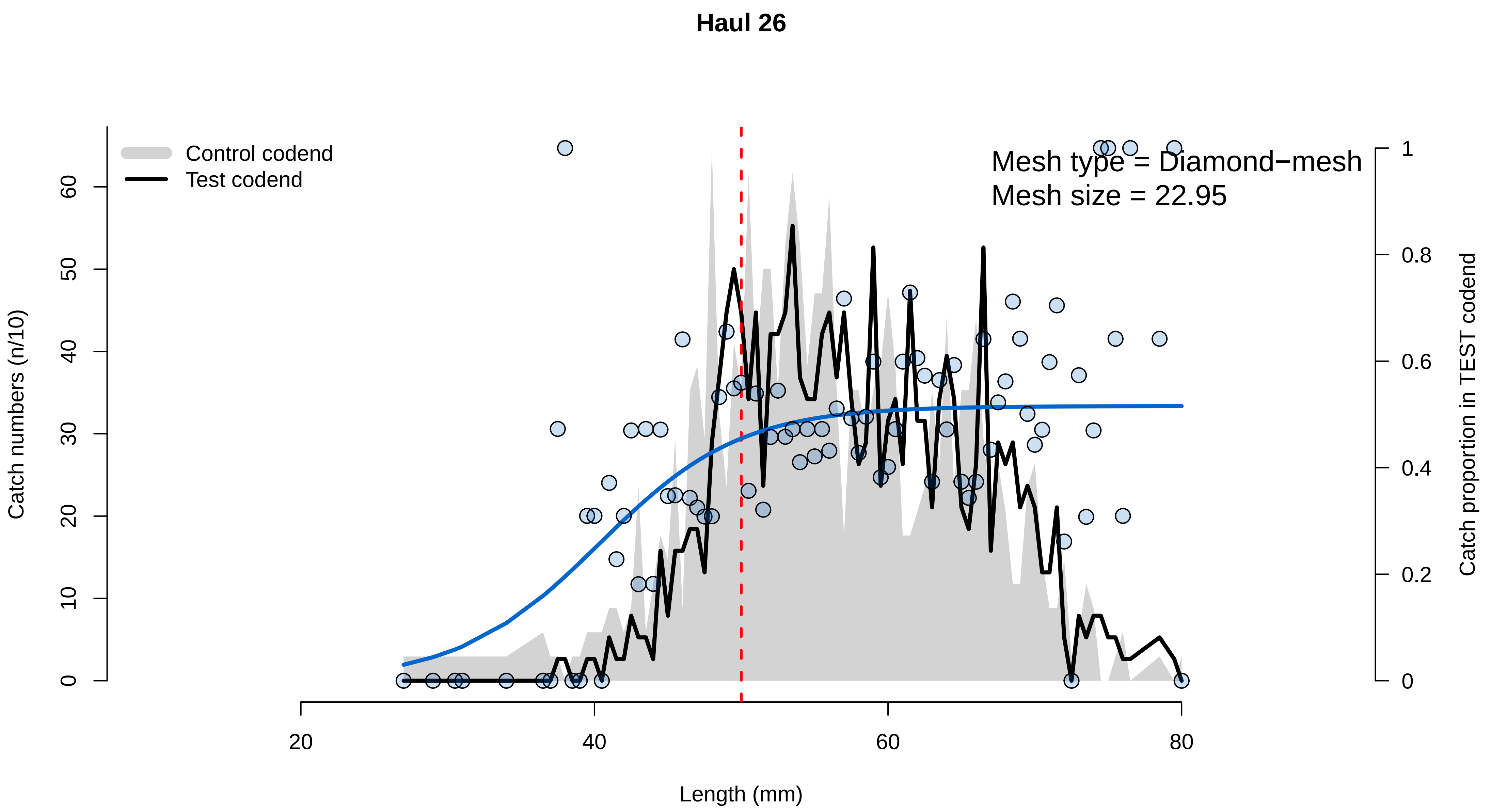

Haul 26
Control codend
Test codend
Mesh type = Diamond−mesh
Mesh size = 22.95
Catch numbers (n/10)
0
10
20
30
40
50
60
Catch proportion in TEST codend
0
0.2
0.4
0.6
0.8
1
20
40
60
80
Length (mm)

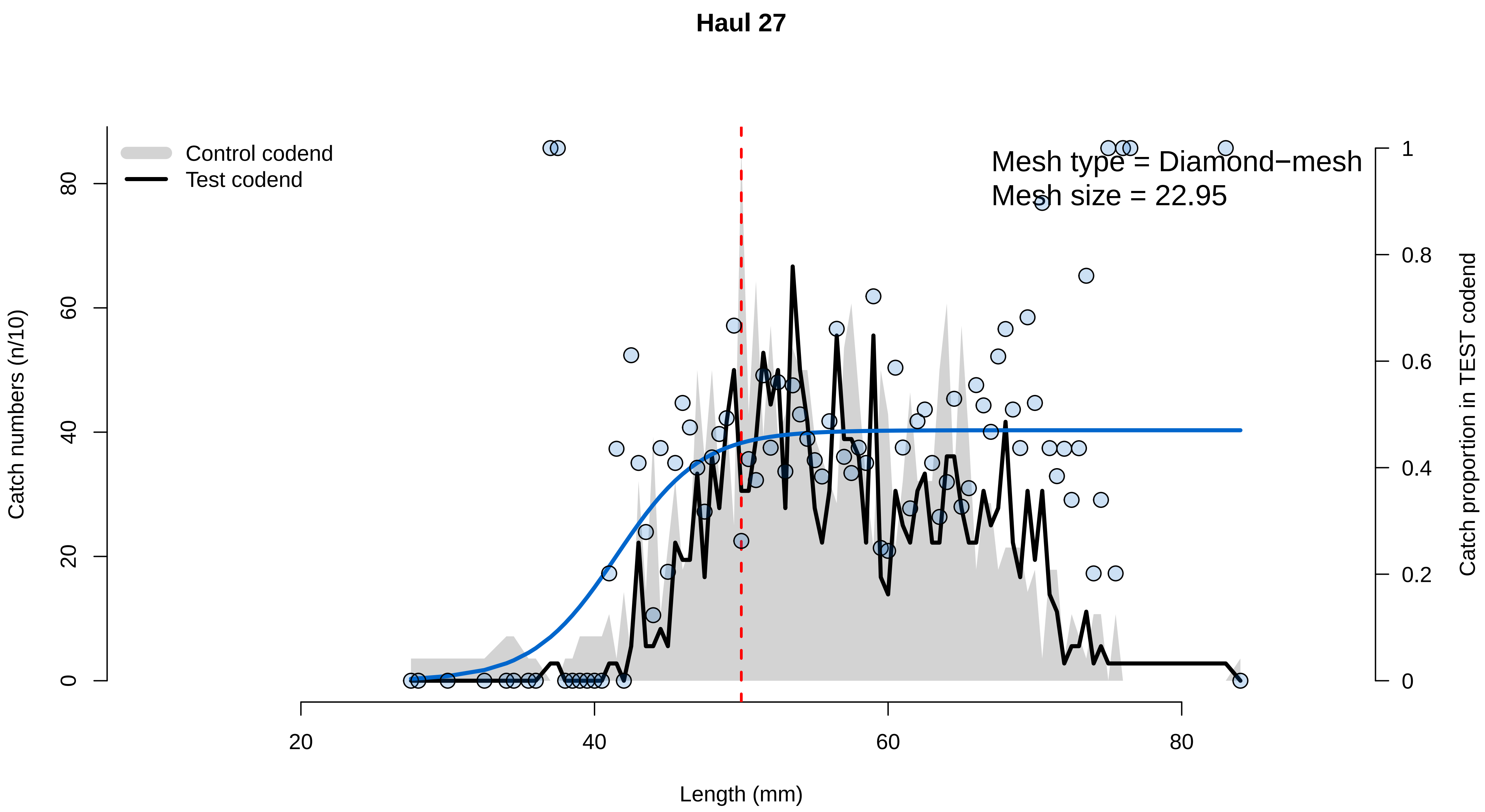
Haul 27
Control codend
Test codend
Mesh type = Diamond−mesh
Mesh size = 22.95
Catch numbers (n/10)
Catch proportion in TEST codend
Length (mm)
0
20
40
60
80
0
0.2
0.4
0.6
0.8
1

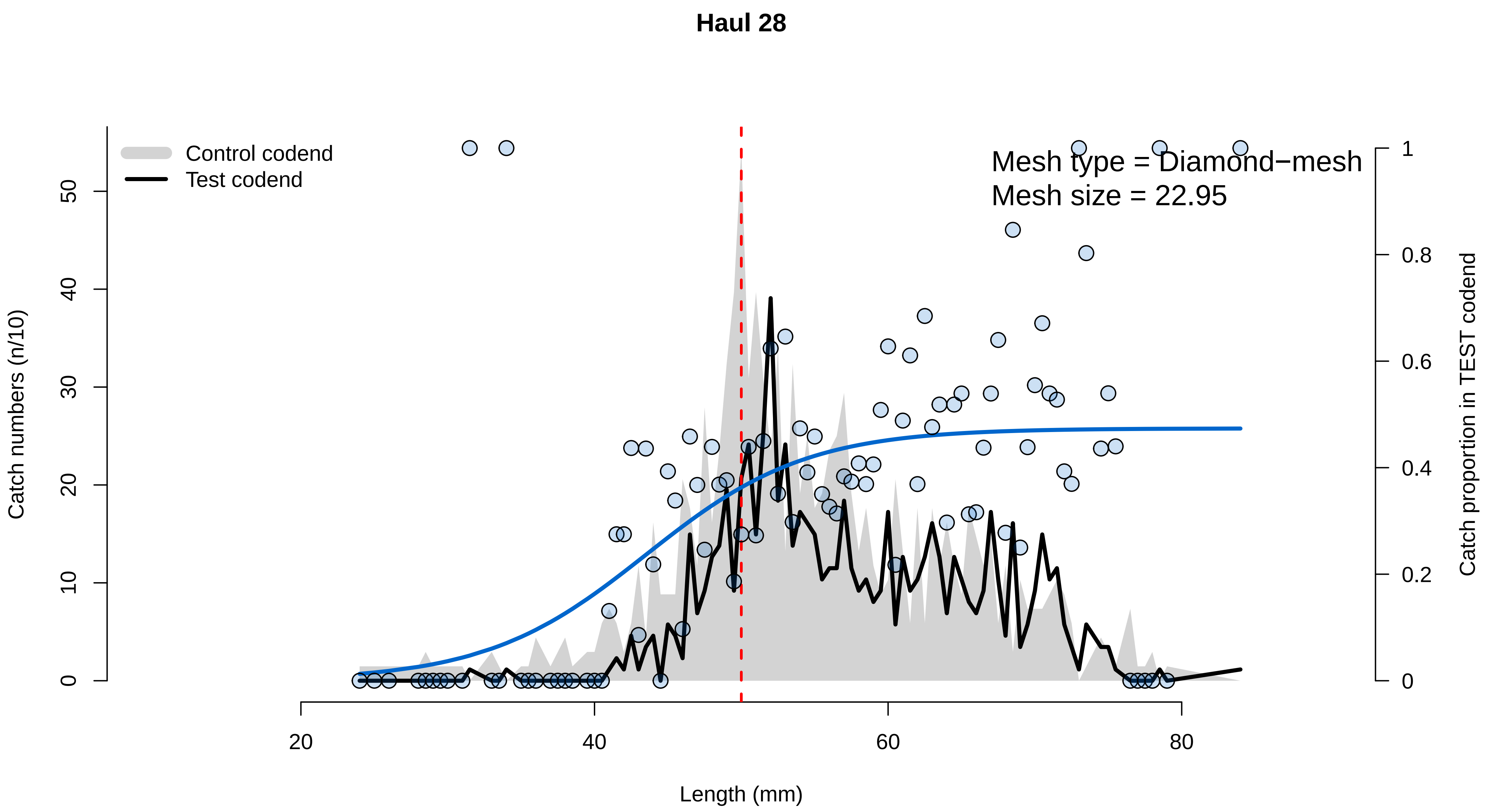

Haul 28
Control codend
Test codend
Mesh type = Diamond−mesh
Mesh size = 22.95
Catch numbers (n/10)
0
10
20
30
40
50
Catch proportion in TEST codend
0
0.2
0.4
0.6
0.8
1
Length (mm)
20
40
60
80

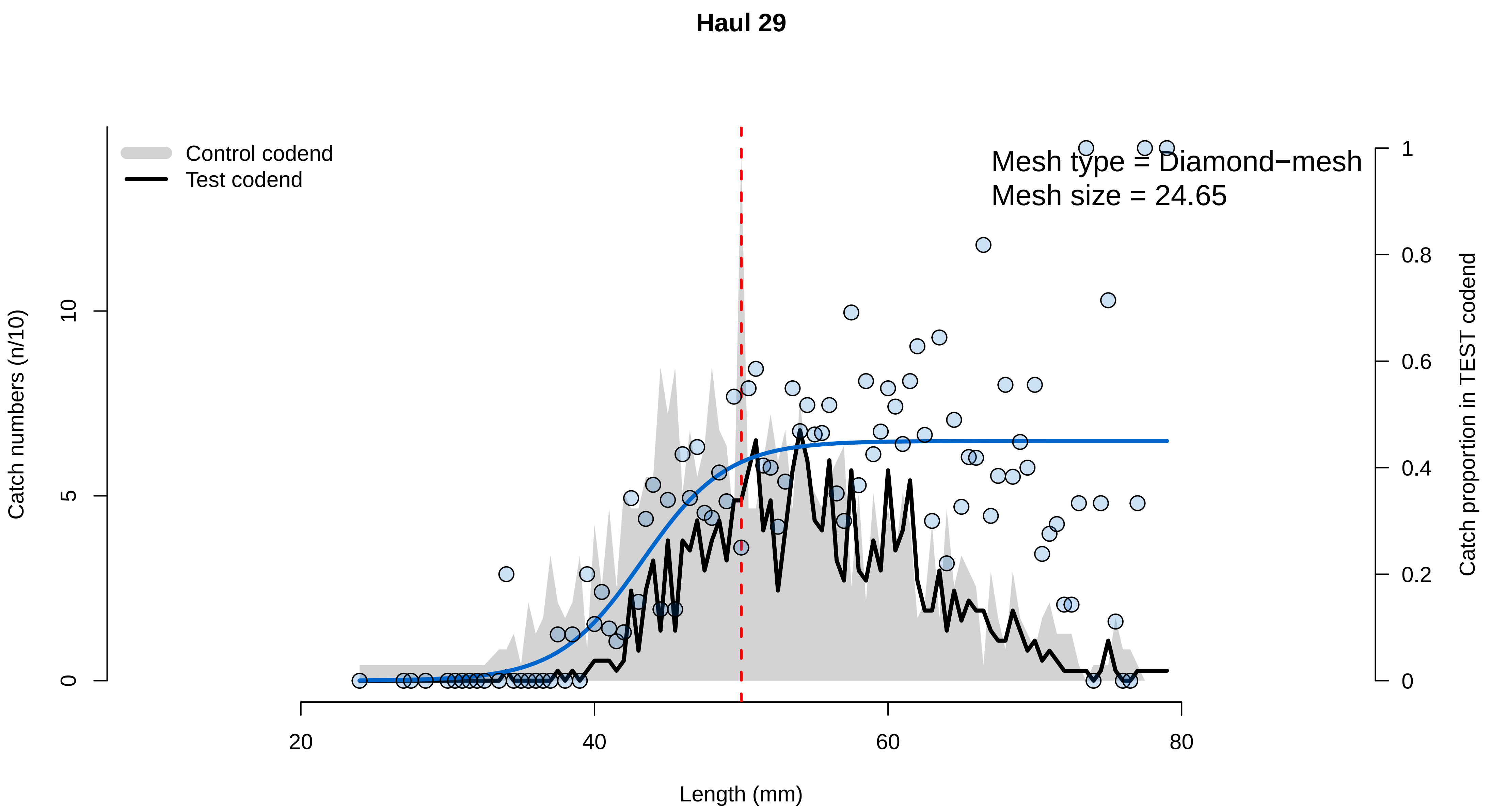

Haul 29
Control codend
Test codend
Mesh type = Diamond−mesh
Mesh size = 24.65
Catch numbers (n/10)
0
5
10
Catch proportion in TEST codend
0
0.2
0.4
0.6
0.8
1
20
40
60
80
Length (mm)

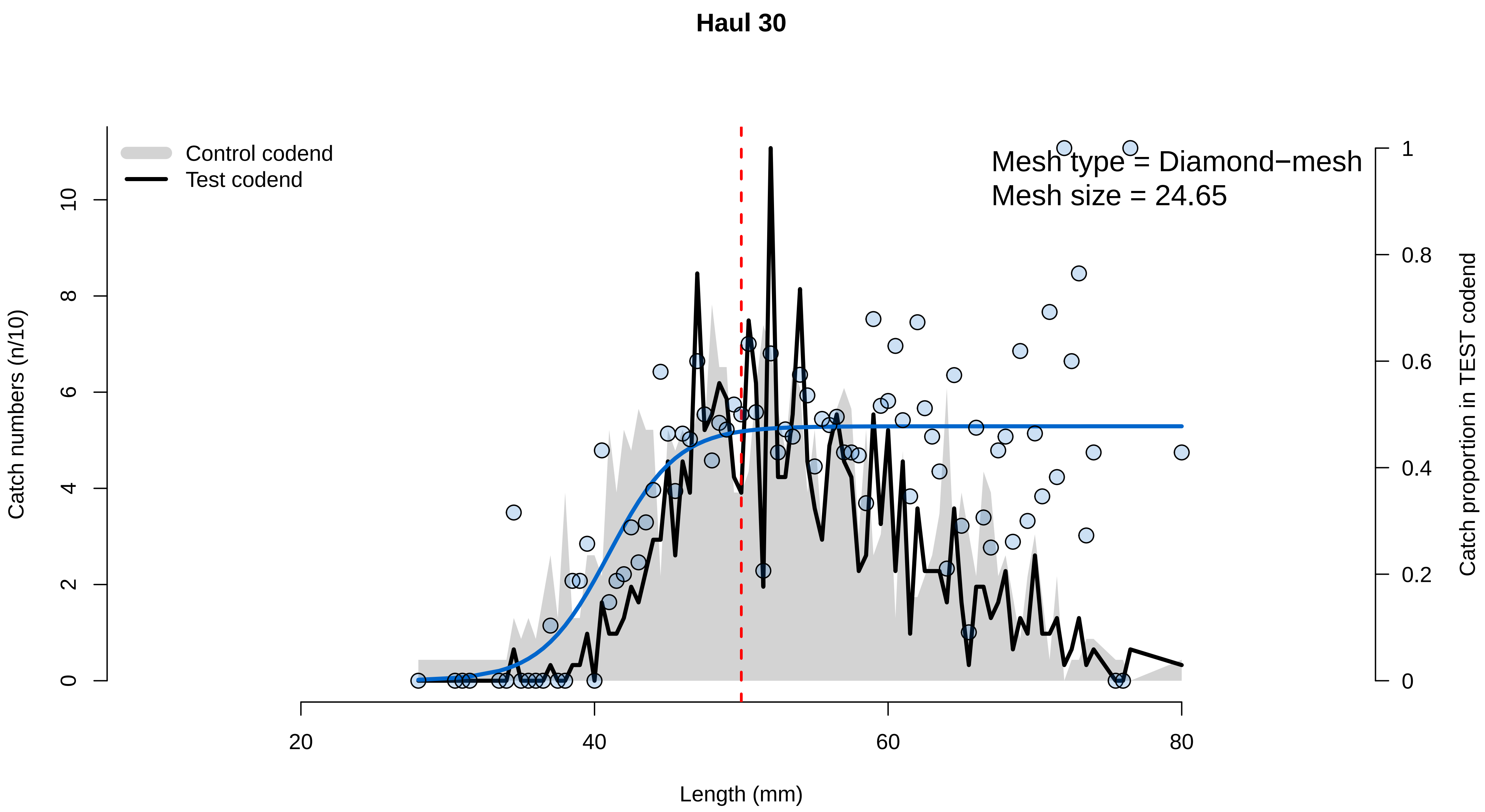

Haul 30
Control codend
Test codend
Mesh type = Diamond−mesh
Mesh size = 24.65
Catch numbers (n/10)
0
2
4
6
8
10
Catch proportion in TEST codend
0
0.2
0.4
0.6
0.8
1
20
40
60
80
Length (mm)

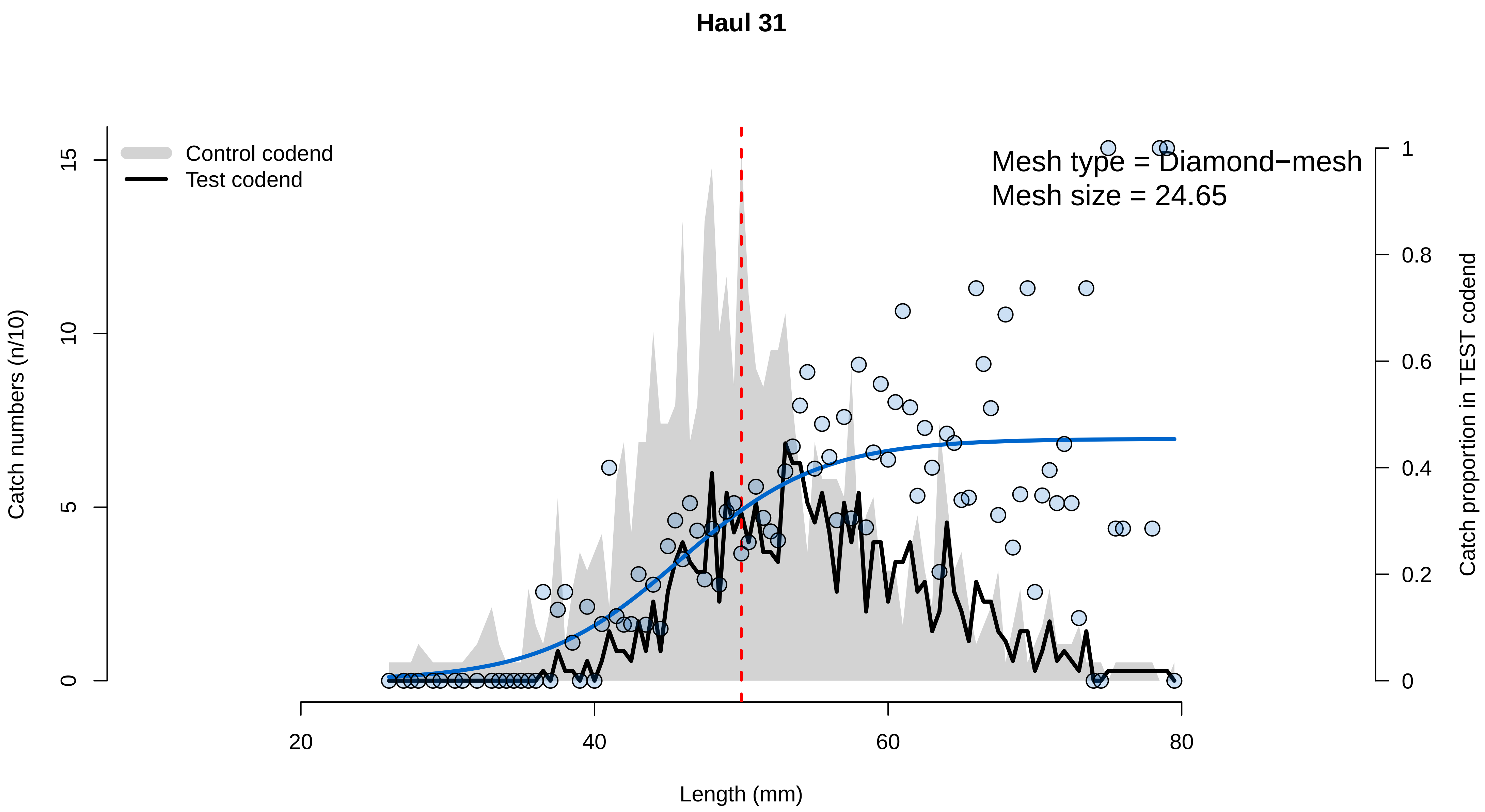
Haul 31
Control codend
Test codend
Mesh type = Diamond−mesh
Mesh size = 24.65
Catch numbers (n/10)
0
5
10
15
Catch proportion in TEST codend
0
0.2
0.4
0.6
0.8
1
20
40
60
80
Length (mm)

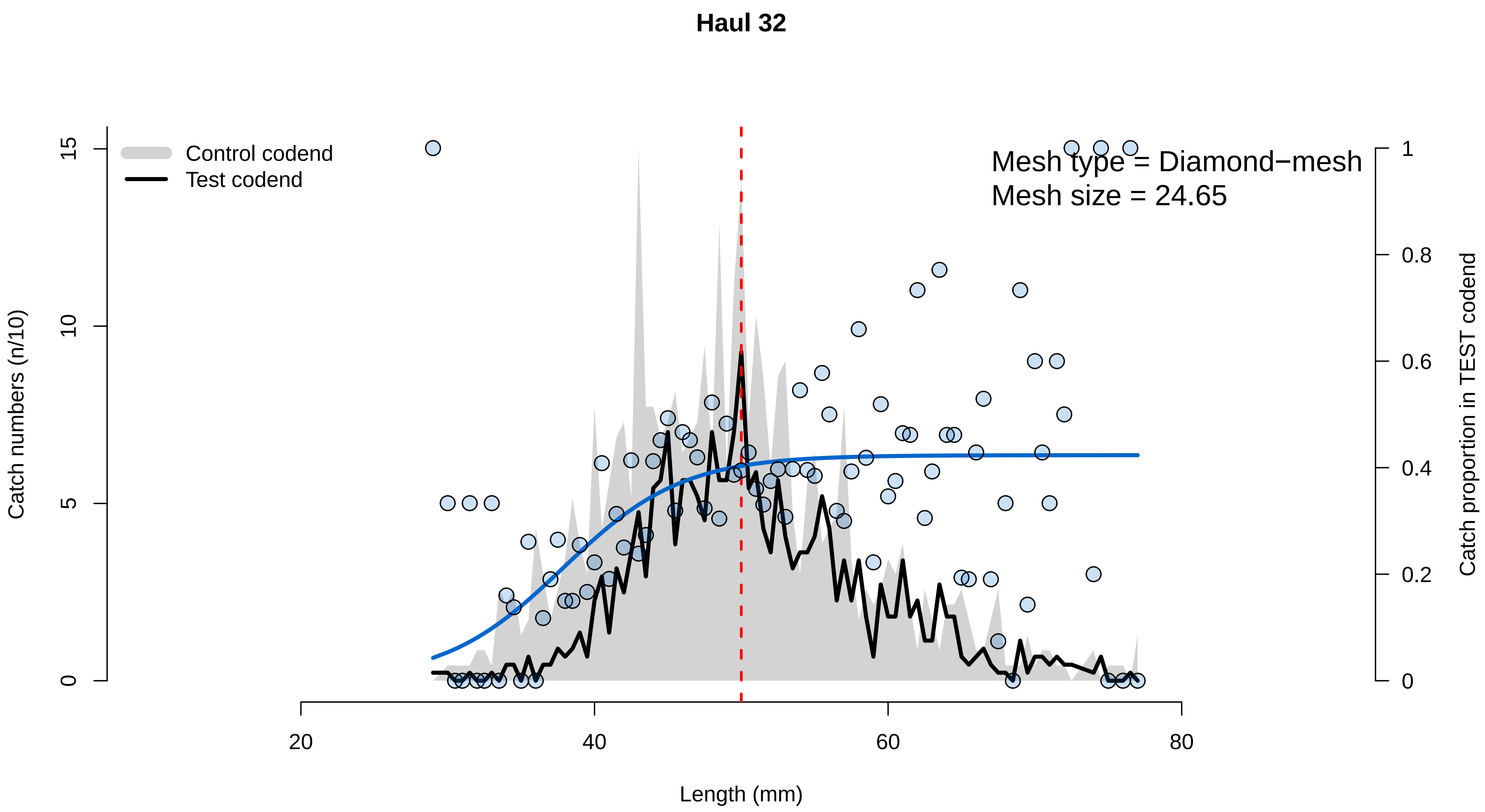

Haul 32
Control codend
Test codend
Mesh type = Diamond−mesh
Mesh size = 24.65
Catch numbers (n/10)
0
5
10
15
Catch proportion in TEST codend
0
0.2
0.4
0.6
0.8
1
20
40
60
80
Length (mm)

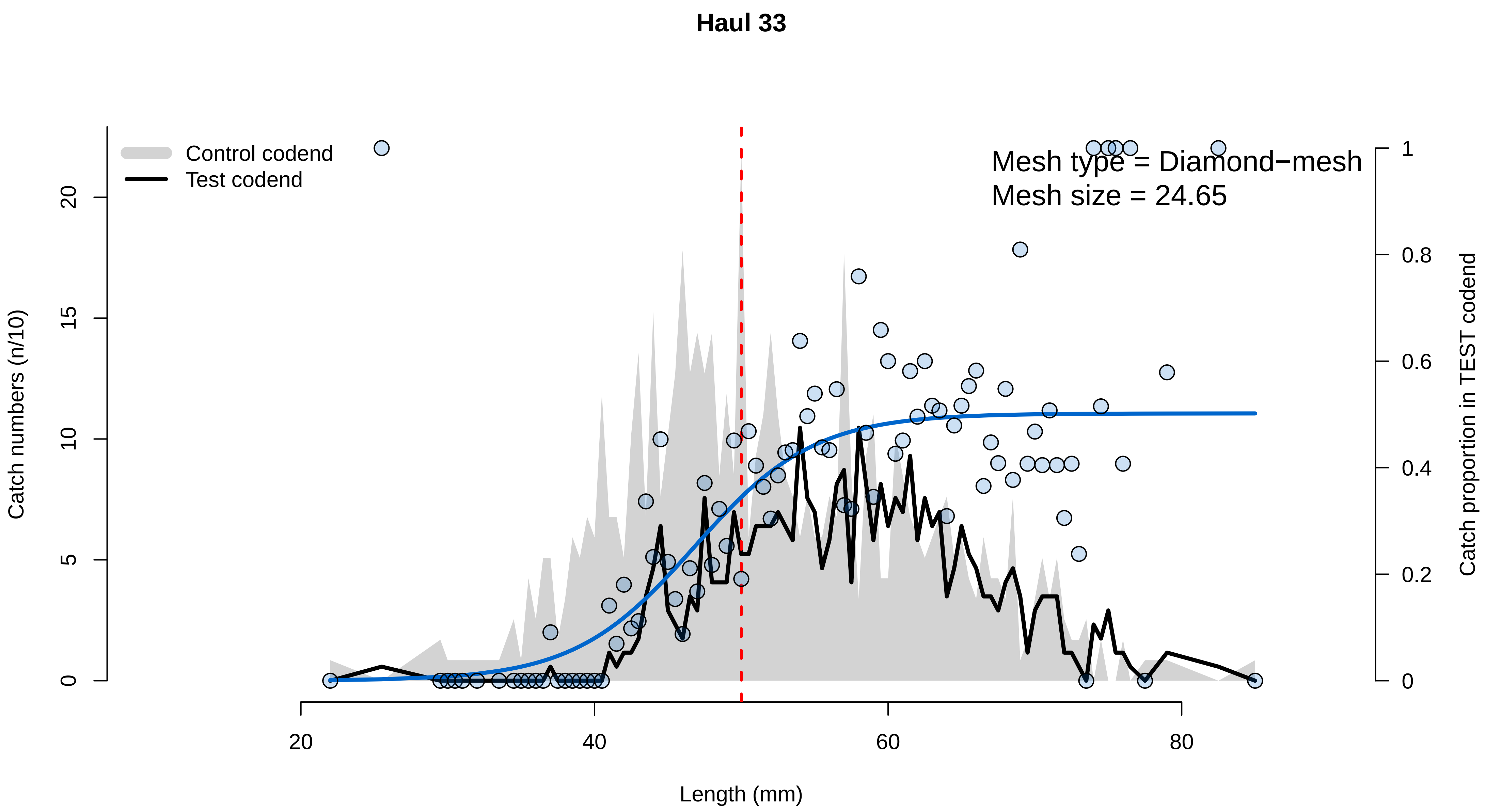
Haul 33
Control codend
Test codend
Mesh type = Diamond−mesh
Mesh size = 24.65
Catch numbers (n/10)
0
5
10
15
20
Catch proportion in TEST codend
0
0.2
0.4
0.6
0.8
1
20
40
60
80
Length (mm)

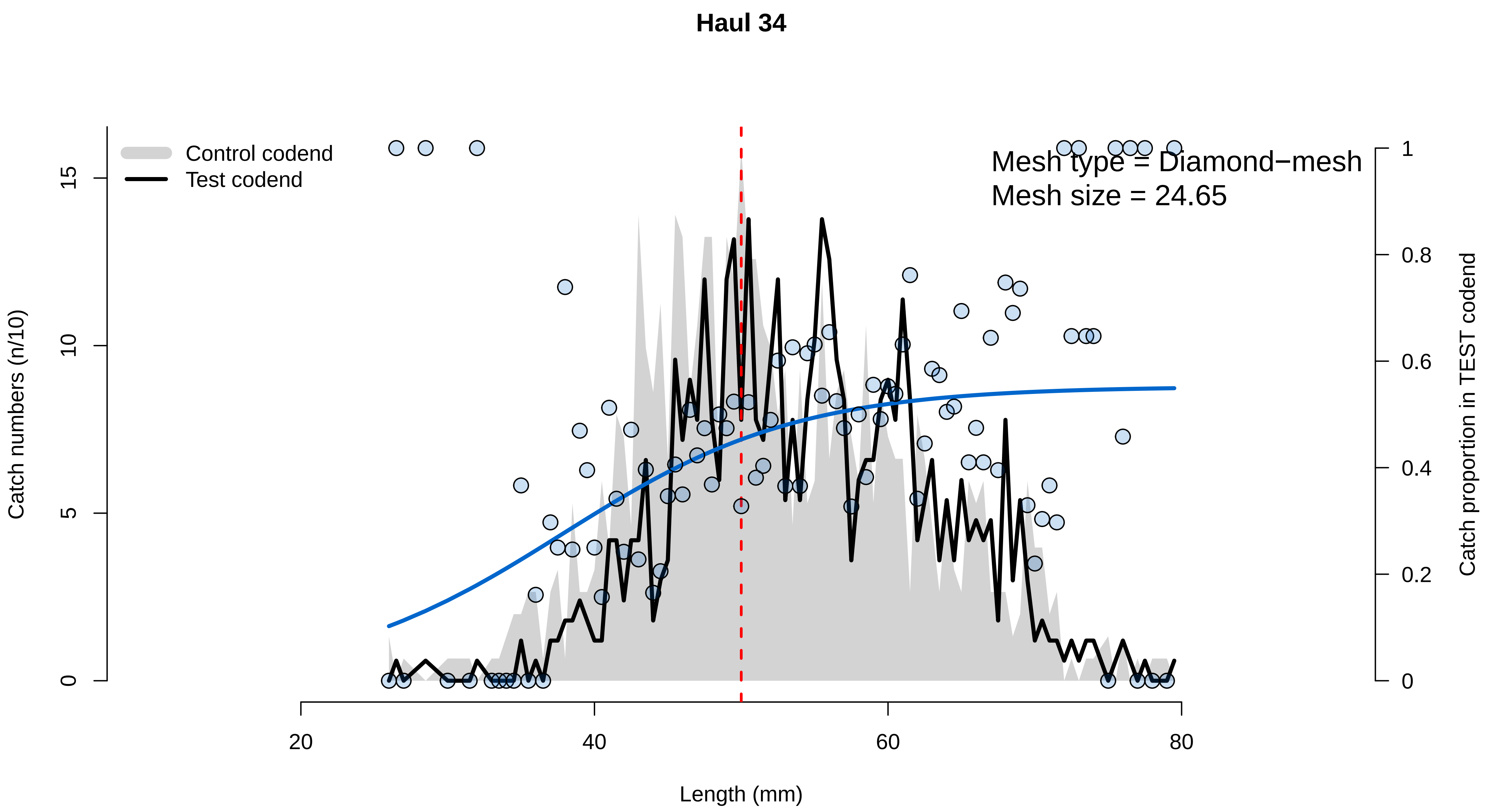

Haul 34
Control codend
Test codend
Mesh type = Diamond−mesh
Mesh size = 24.65
Catch numbers (n/10)
Catch proportion in TEST codend
Length (mm)
0
5
10
15
0
0.2
0.4
0.6
0.8
1
20
40
60
80

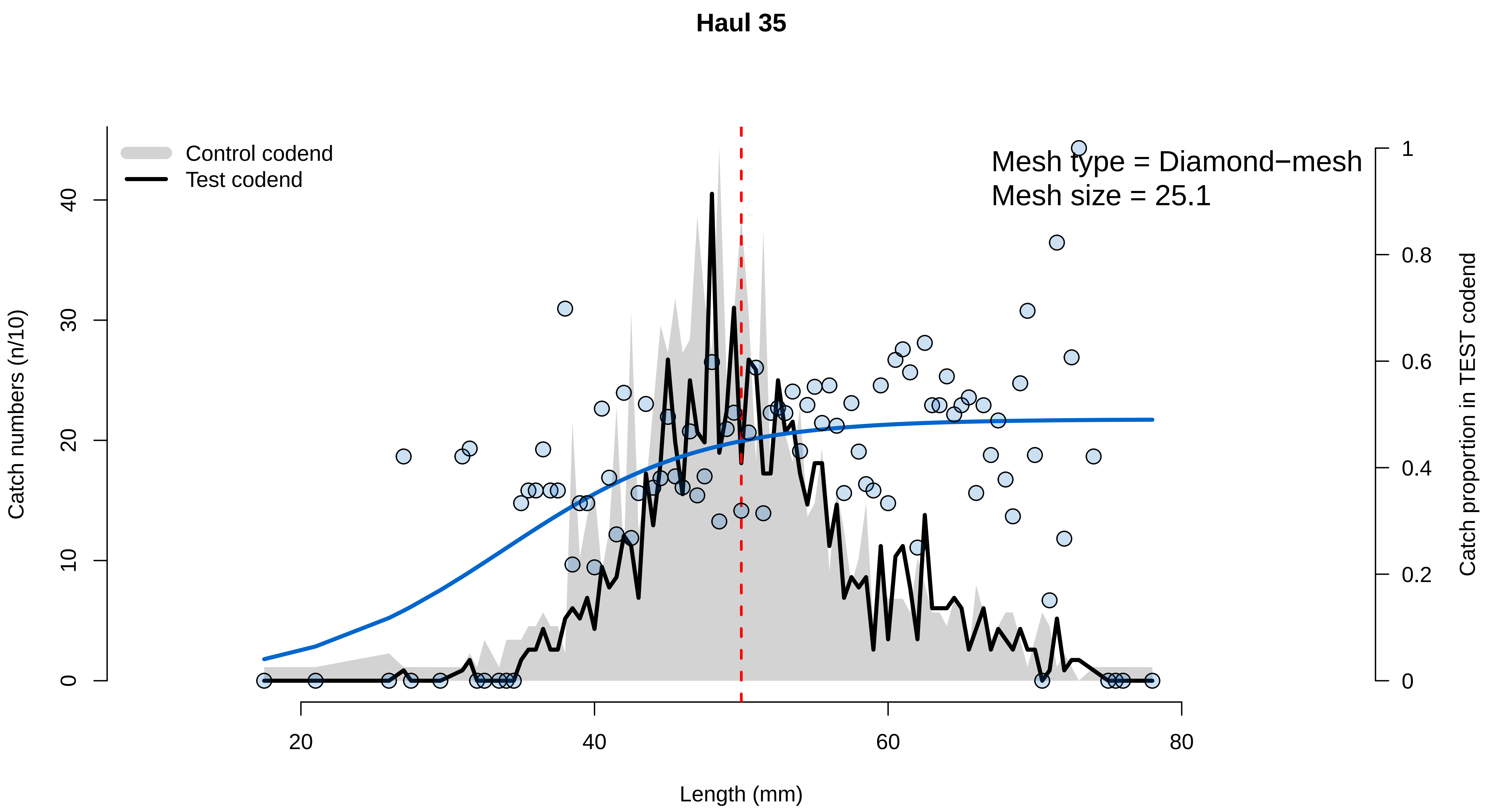
Haul 35
Control codend
Test codend
Mesh type = Diamond−mesh
Mesh size = 25.1
Catch numbers (n/10)
0
10
20
30
40
Catch proportion in TEST codend
0
0.2
0.4
0.6
0.8
1
20
40
60
80
Length (mm)

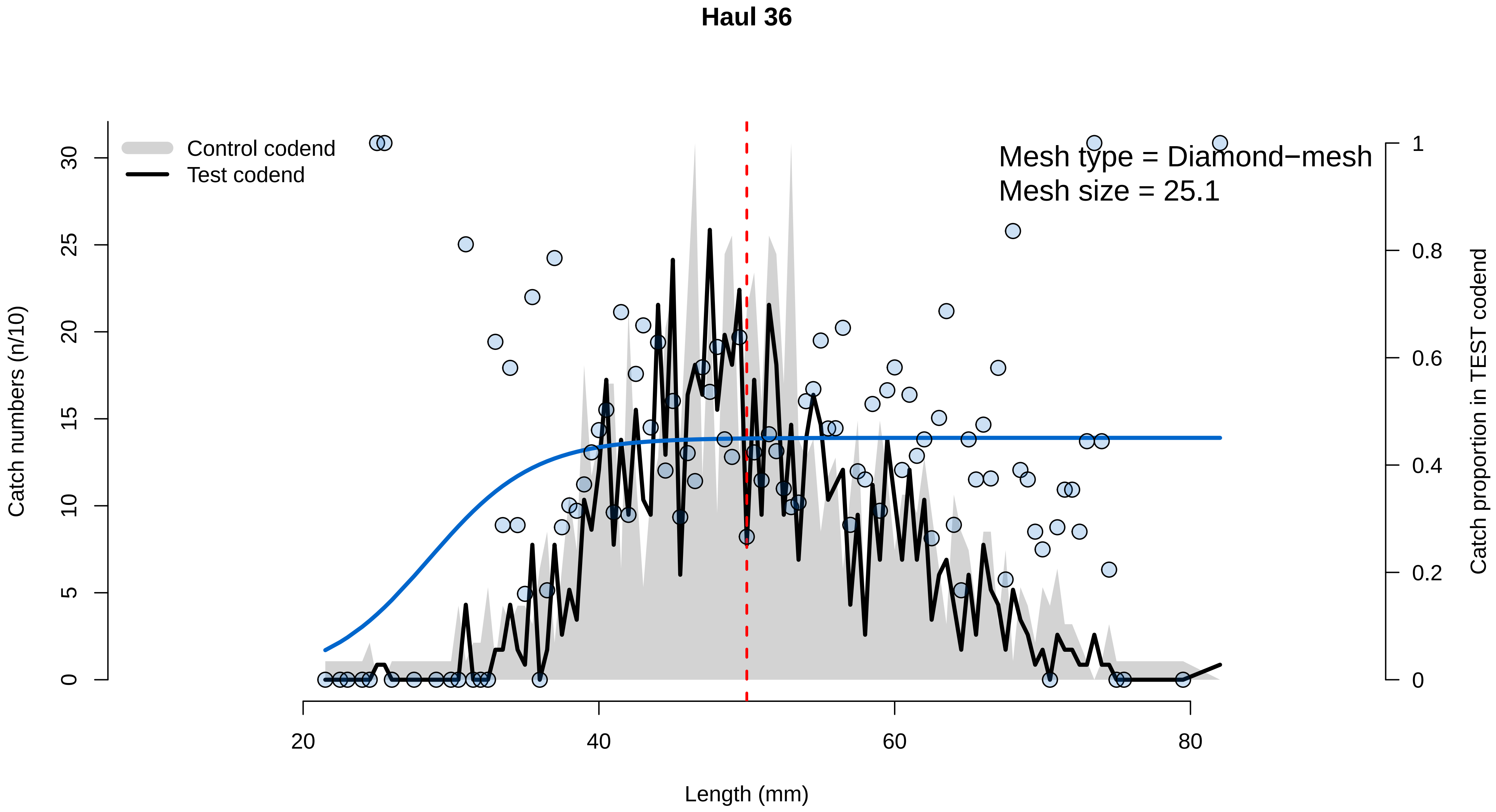

Haul 36
Control codend
Test codend
Mesh type = Diamond−mesh
Mesh size = 25.1
Catch numbers (n/10)
Catch proportion in TEST codend
Length (mm)
0
5
10
15
20
25
30
0
0.2
0.4
0.6
0.8
1
20
40
60
80

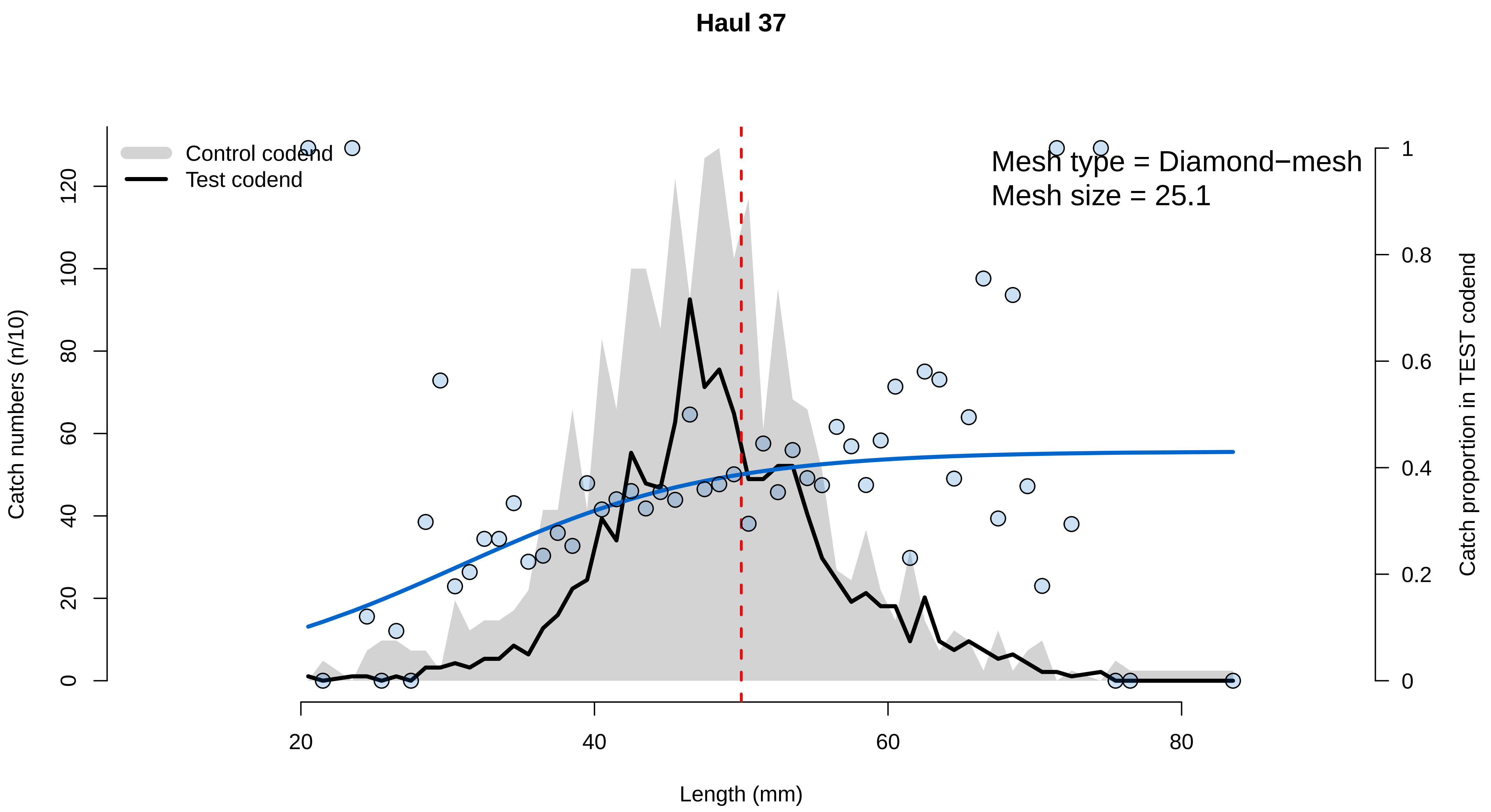

Haul 37
Control codend
Test codend
Mesh type = Diamond−mesh
Mesh size = 25.1
Catch numbers (n/10)
0
20
40
60
80
100
120
Catch proportion in TEST codend
0
0.2
0.4
0.6
0.8
1
20
40
60
80
Length (mm)

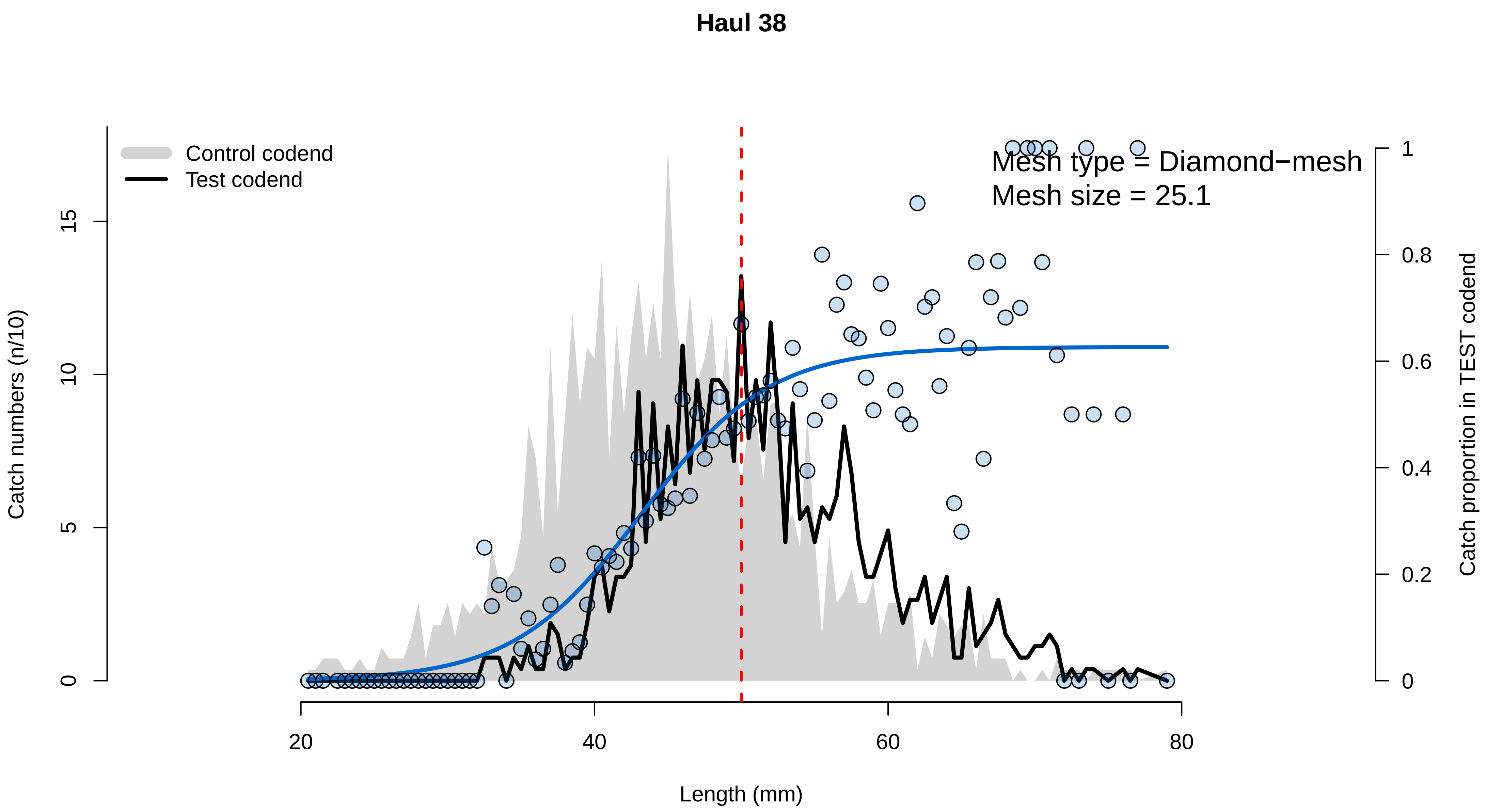
Haul 38
Control codend
Test codend
Mesh type = Diamond−mesh
Mesh size = 25.1
Catch numbers (n/10)
0
5
10
15
20
40
60
80
Length (mm)
Catch proportion in TEST codend
0
0.2
0.4
0.6
0.8
1

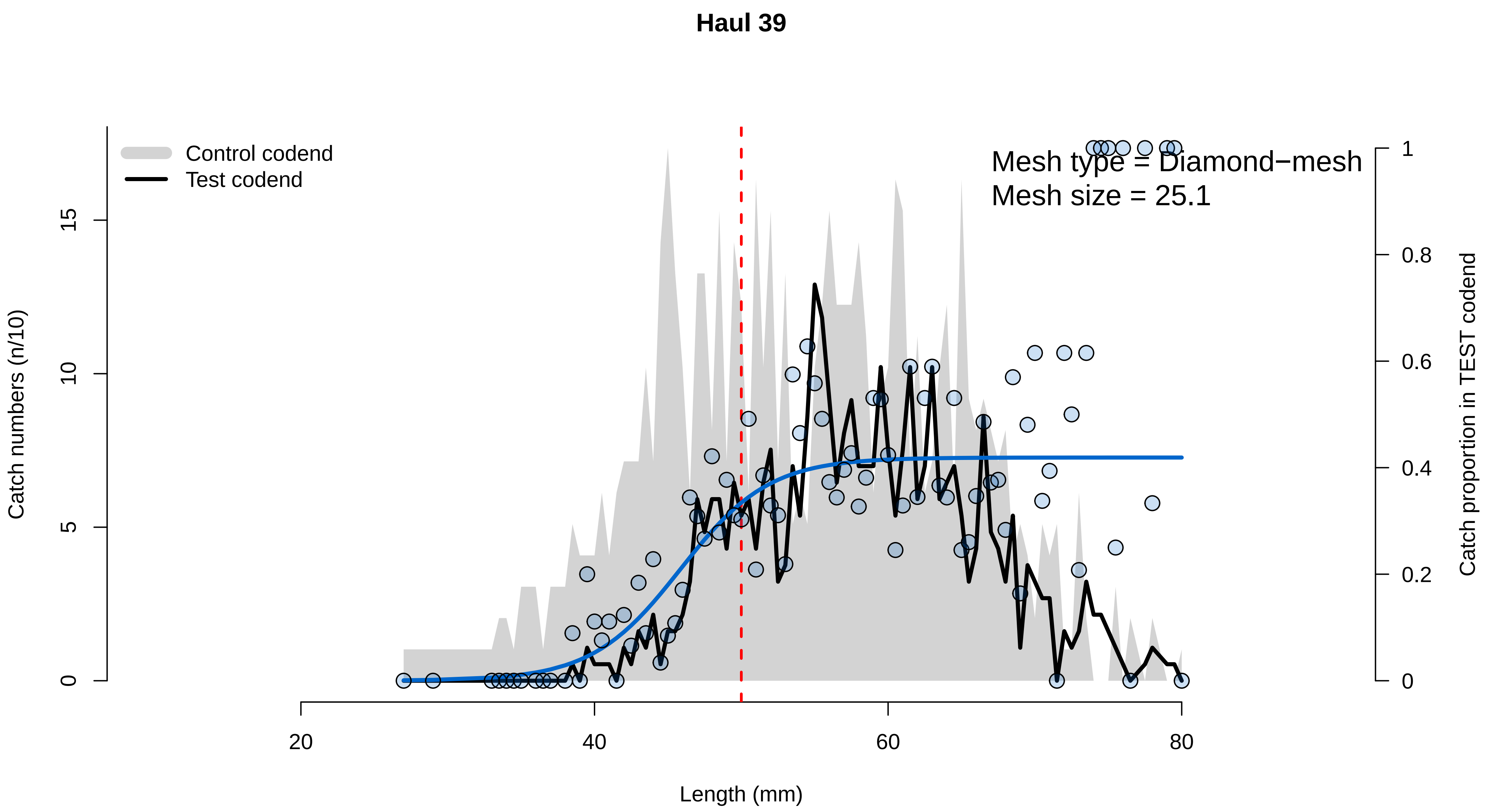

Haul 39
Control codend
Test codend
Mesh type = Diamond−mesh
Mesh size = 25.1
Catch numbers (n/10)
Catch proportion in TEST codend
Length (mm)
0
5
10
15
20
40
60
80
0
0.2
0.4
0.6
0.8
1

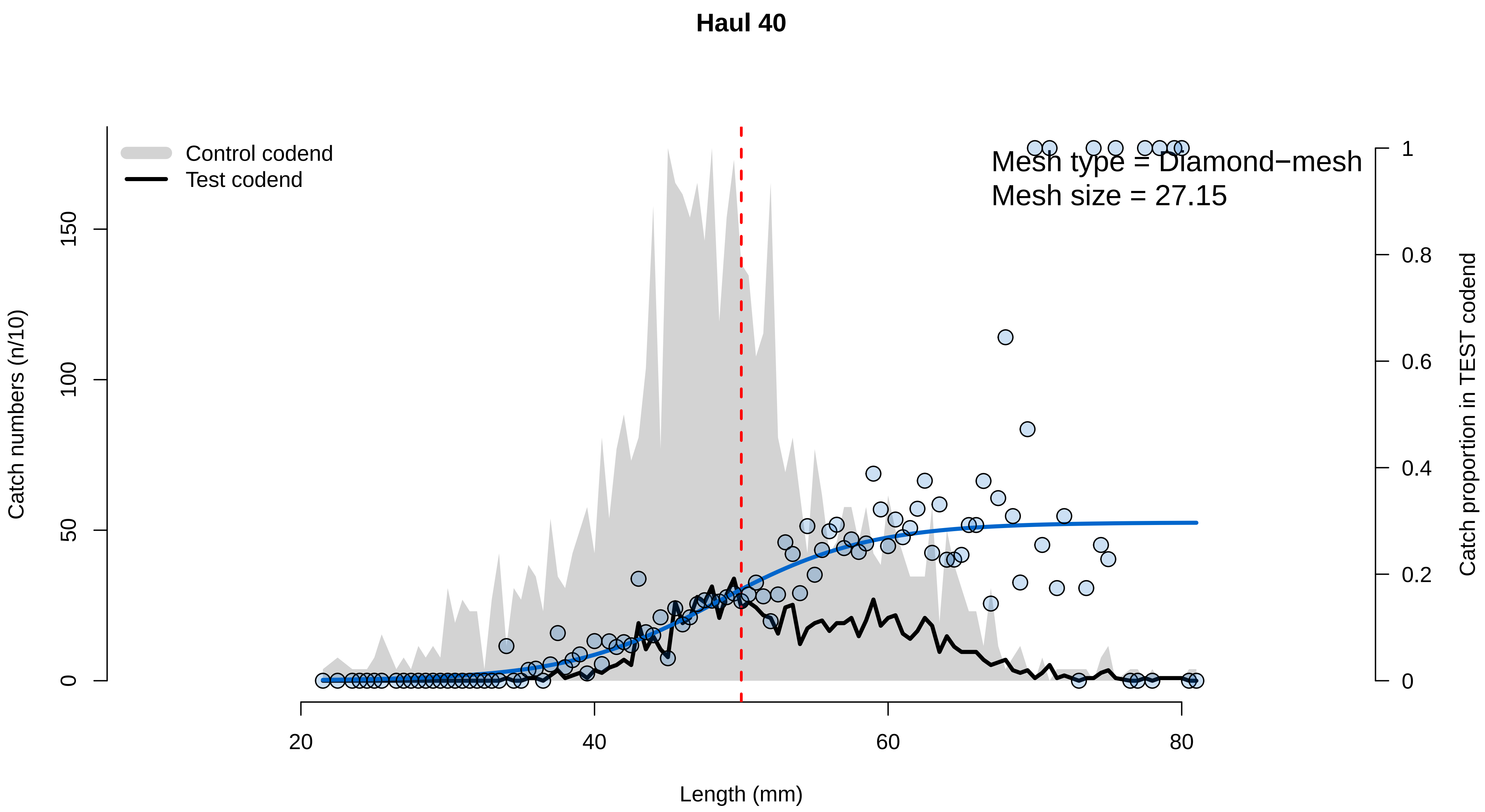
Haul 40
Control codend
Test codend
Mesh type = Diamond−mesh
Mesh size = 27.15
Catch numbers (n/10)
0
50
100
150
Catch proportion in TEST codend
0
0.2
0.4
0.6
0.8
1
20
40
60
80
Length (mm)

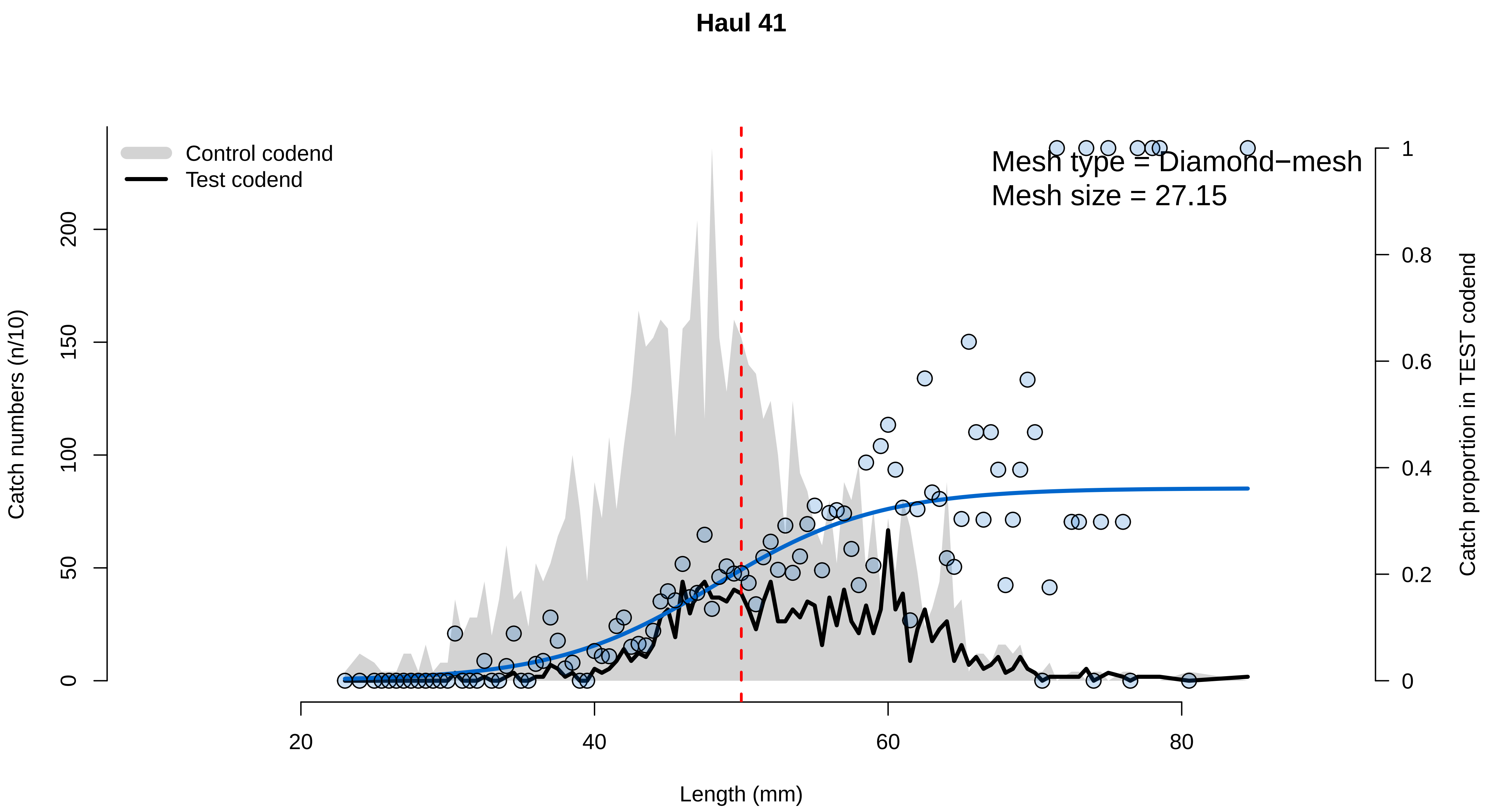
Haul 41
Control codend
Test codend
Mesh type = Diamond−mesh
Mesh size = 27.15
Catch numbers (n/10)
0
50
100
150
200
Catch proportion in TEST codend
0
0.2
0.4
0.6
0.8
1
20
40
60
80
Length (mm)

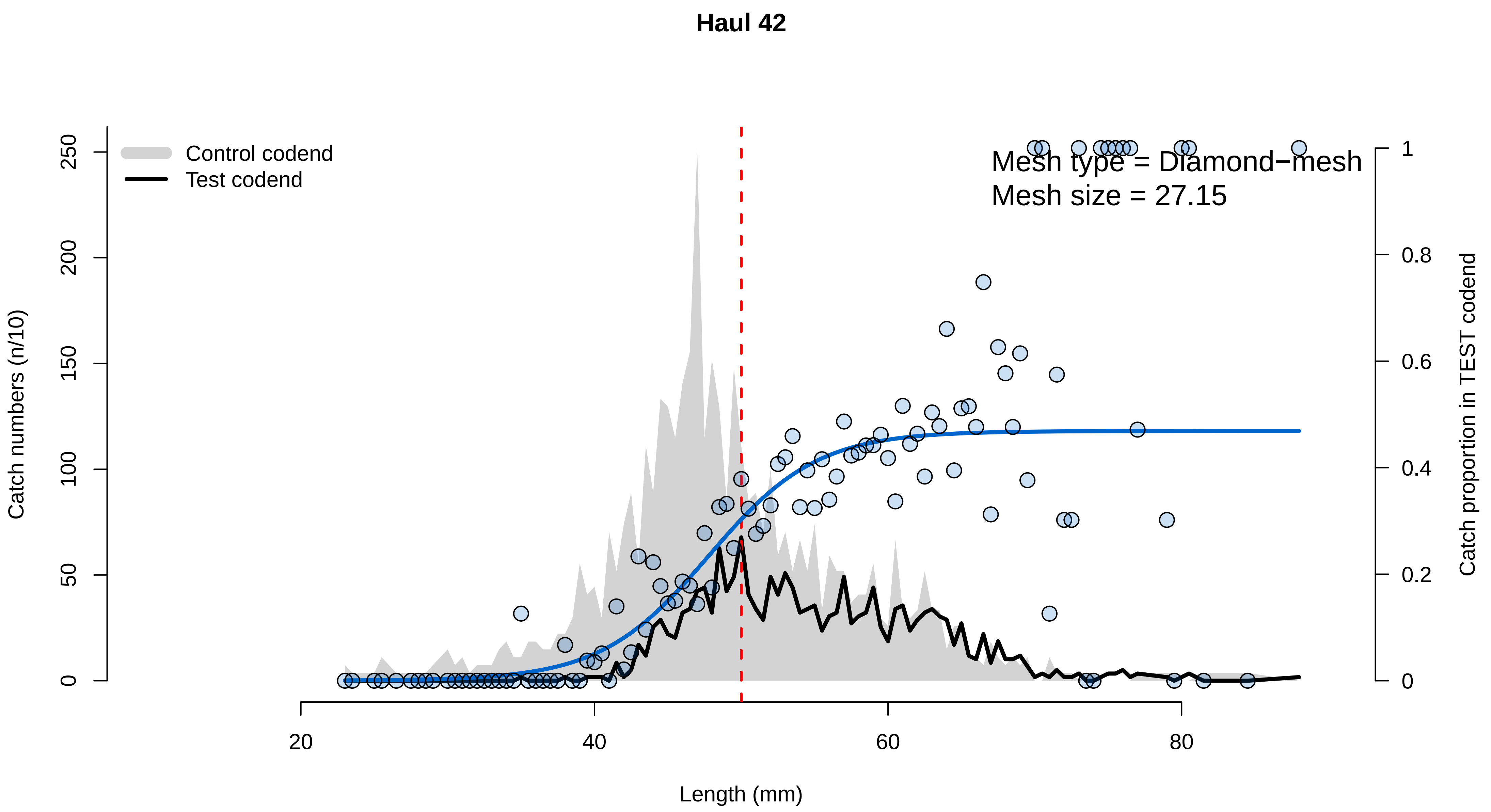

Haul 42
Control codend
Test codend
Mesh type = Diamond−mesh
Mesh size = 27.15
Catch numbers (n/10)
0
50
100
150
200
250
Catch proportion in TEST codend
0
0.2
0.4
0.6
0.8
1
20
40
60
80
Length (mm)

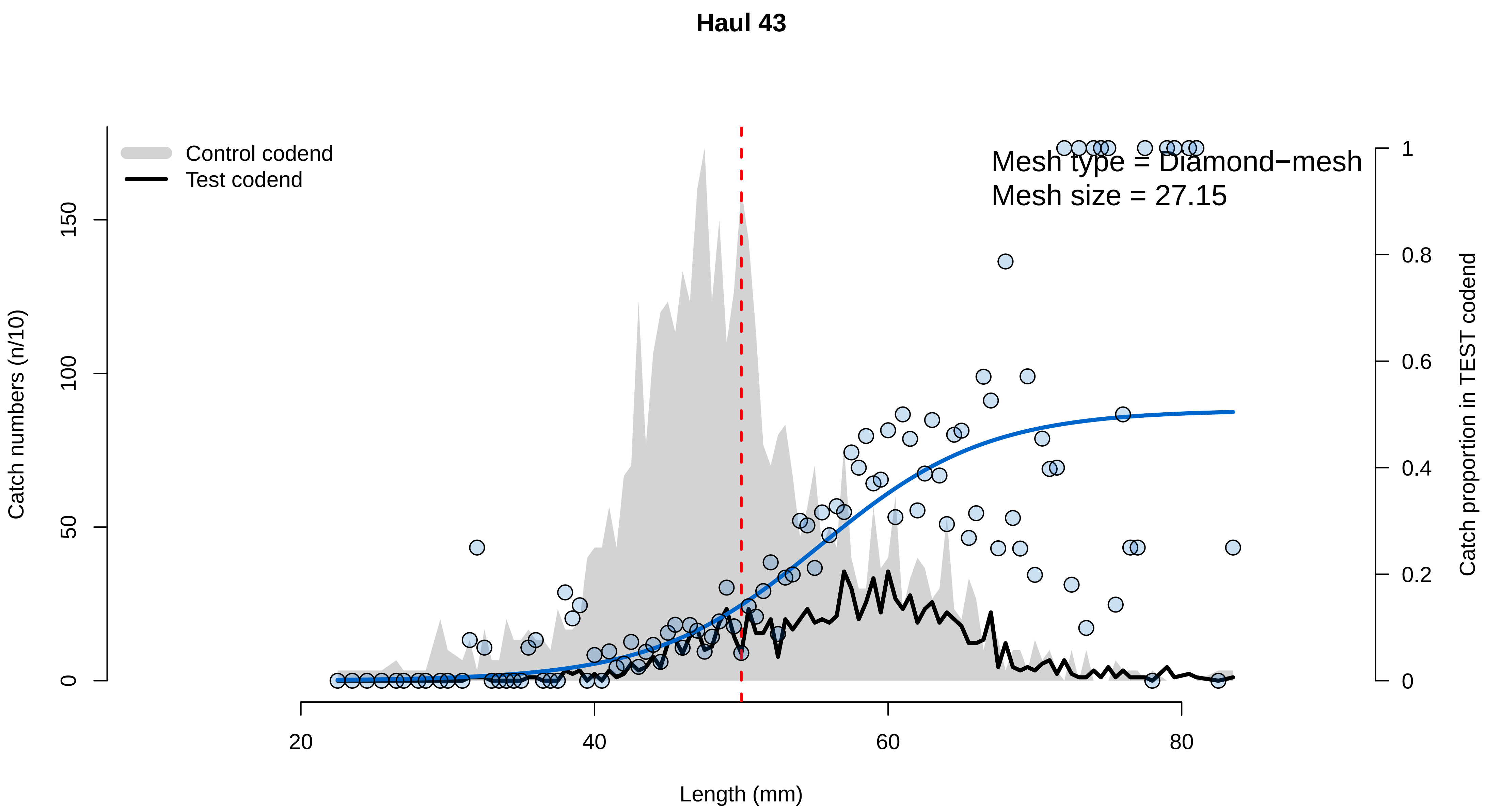
Haul 43
Control codend
Test codend
Mesh type = Diamond−mesh
Mesh size = 27.15
Catch numbers (n/10)
0
50
100
150
Catch proportion in TEST codend
0
0.2
0.4
0.6
0.8
1
20
40
60
80
Length (mm)

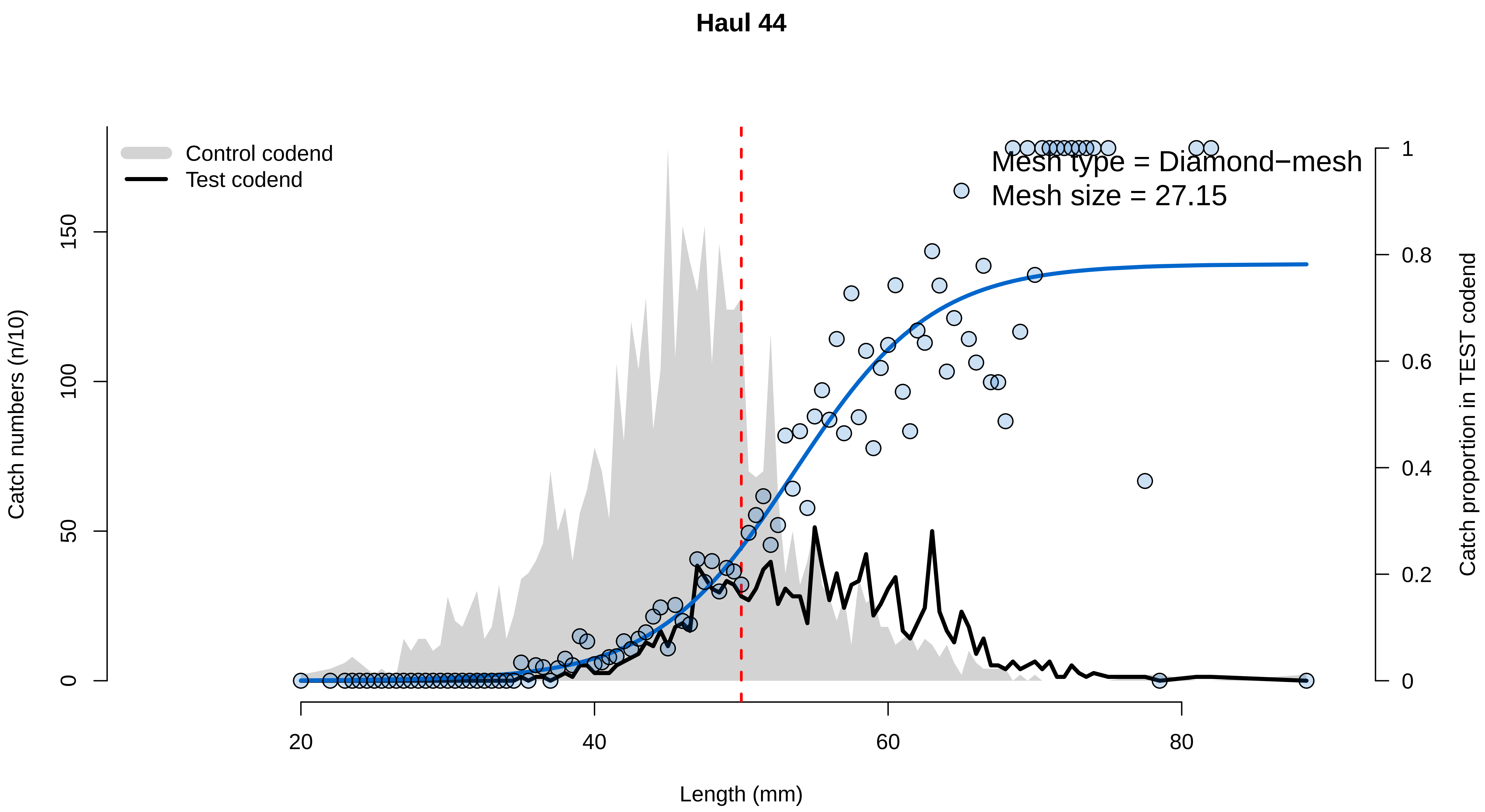
Haul 44
Control codend
Test codend
Mesh type = Diamond−mesh
Mesh size = 27.15
Catch numbers (n/10)
0
50
100
150
Catch proportion in TEST codend
0
0.2
0.4
0.6
0.8
1
20
40
60
80
Length (mm)

Haul 45

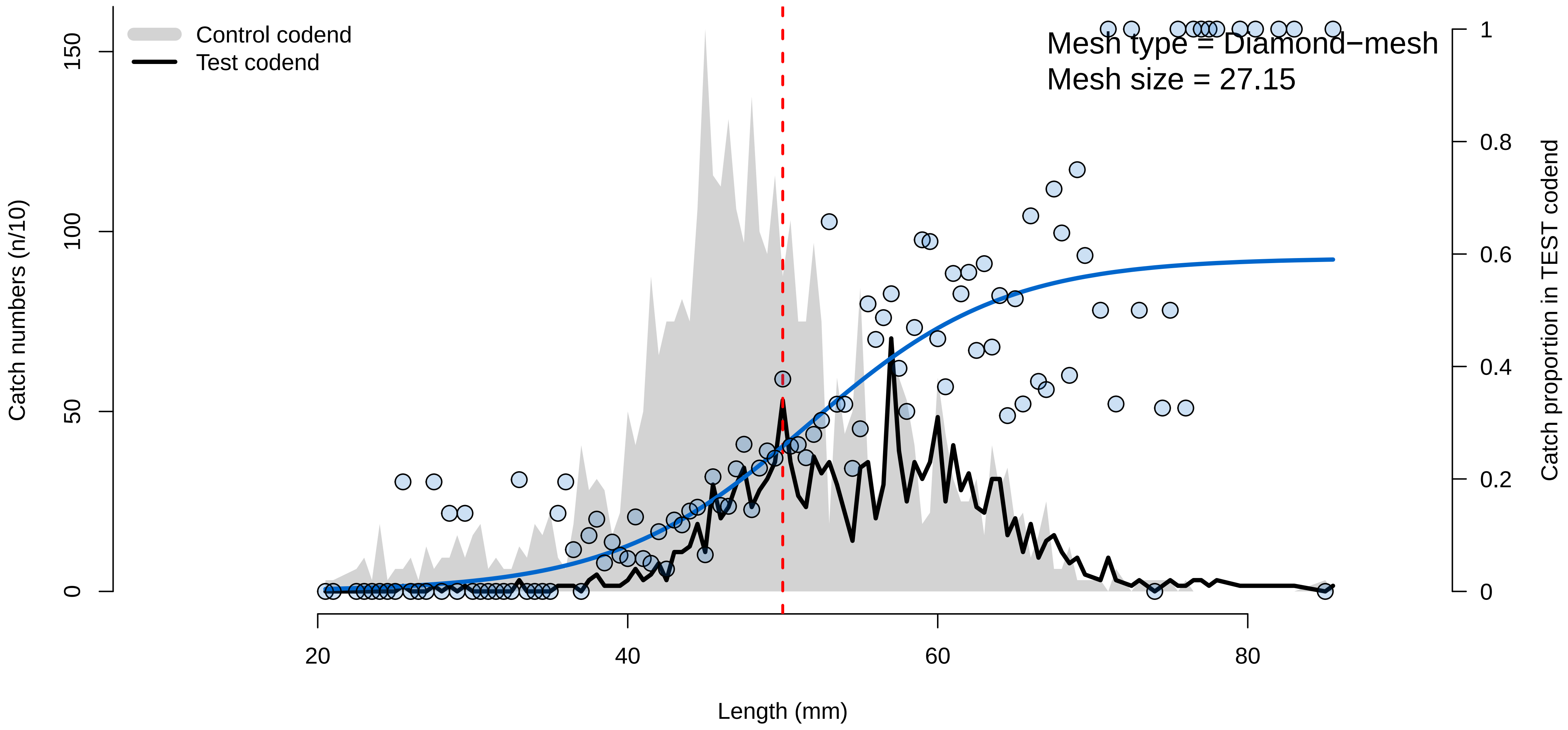
Control codend
Test codend
Mesh type = Diamond−mesh
Mesh size = 27.15
Catch numbers (n/10)
0
50
100
150
Catch proportion in TEST codend
0
0.2
0.4
0.6
0.8
1
20
40
60
80
Length (mm)

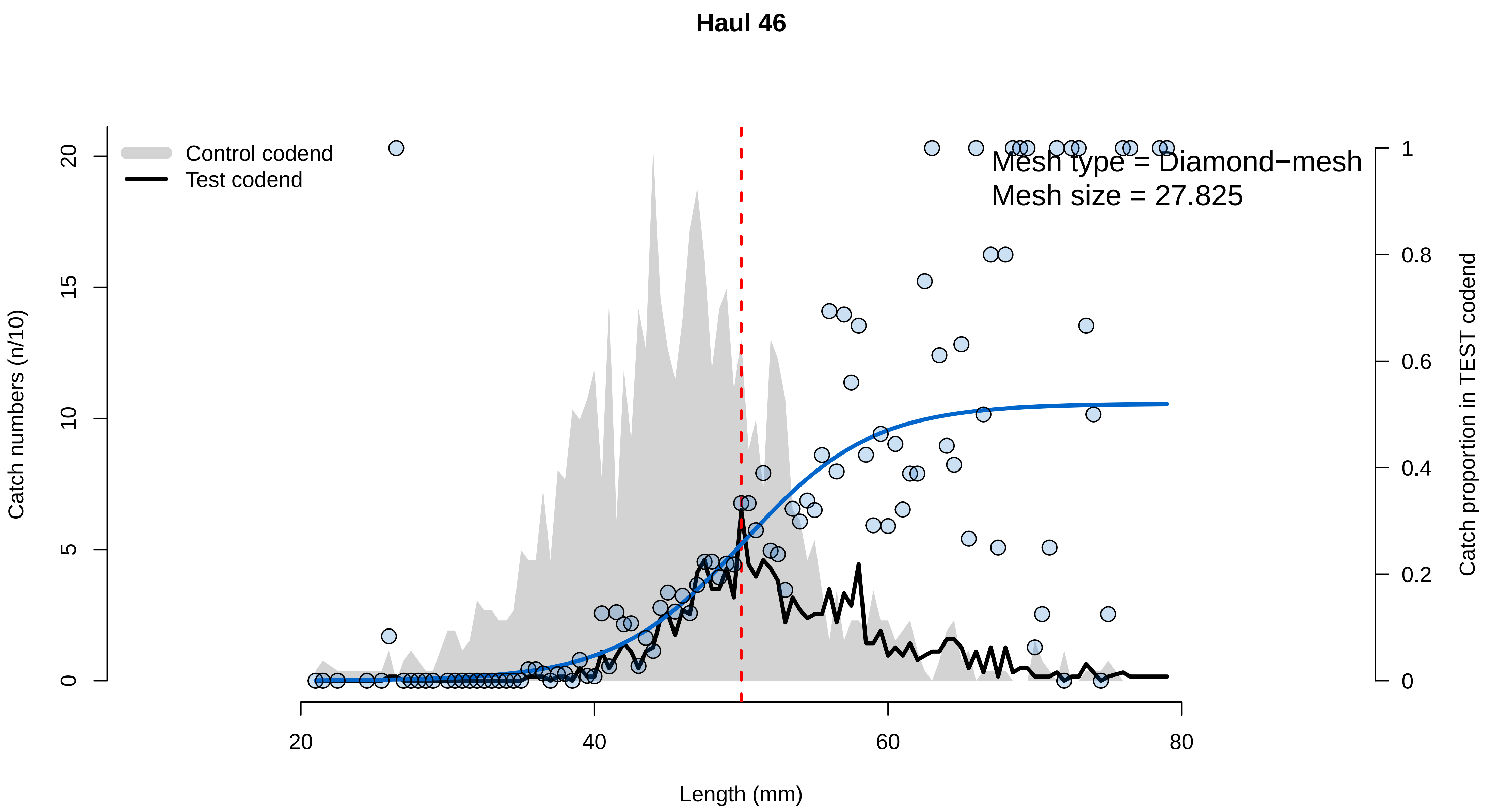
Haul 46
Control codend
Test codend
Mesh type = Diamond−mesh
Mesh size = 27.825
Catch numbers (n/10)
0
5
10
15
20
Catch proportion in TEST codend
0
0.2
0.4
0.6
0.8
1
20
40
60
80
Length (mm)

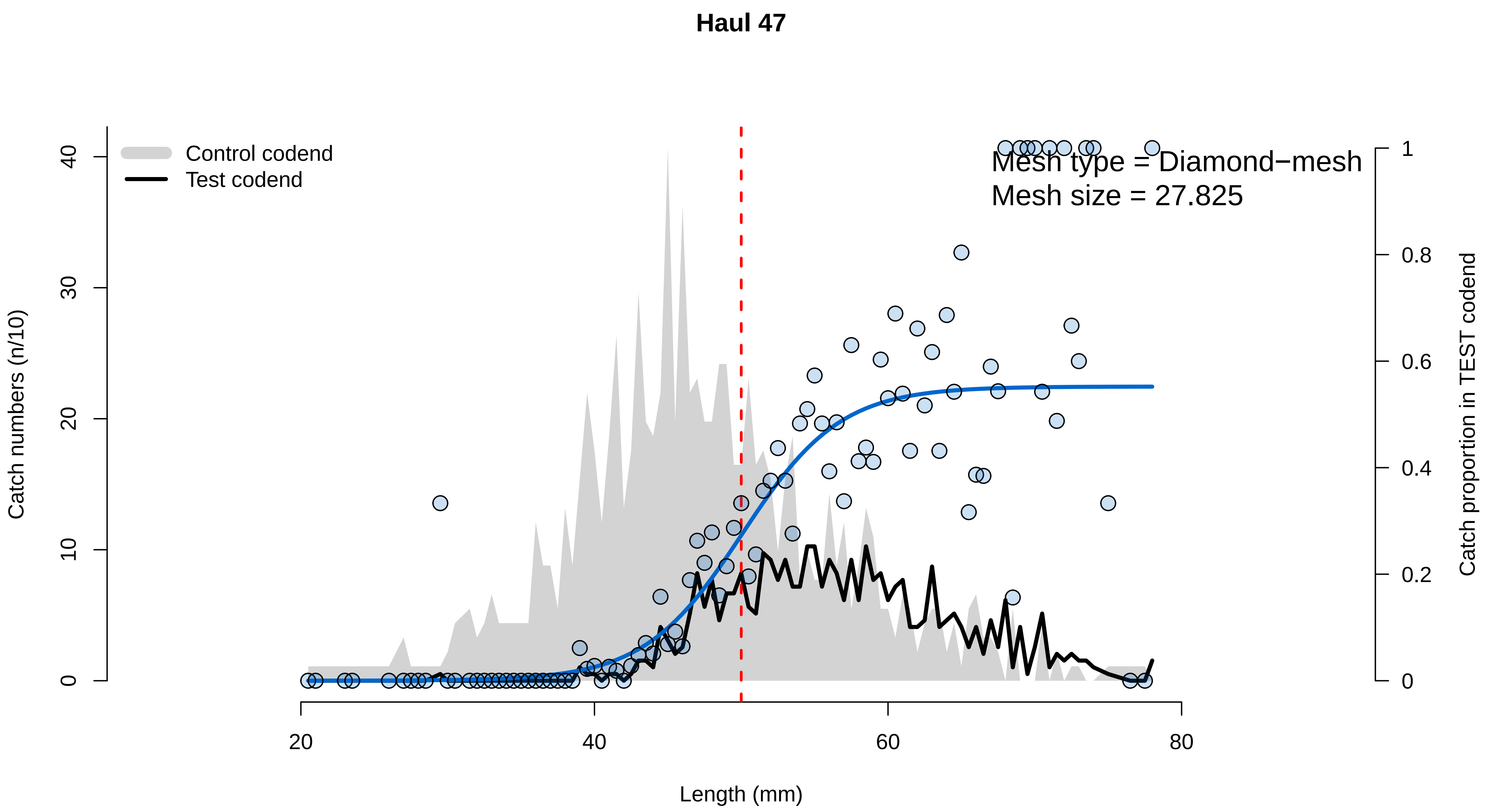

Haul 47
Control codend
Test codend
Mesh type = Diamond−mesh
Mesh size = 27.825
Catch numbers (n/10)
0
10
20
30
40
Catch proportion in TEST codend
0
0.2
0.4
0.6
0.8
1
20
40
60
80
Length (mm)

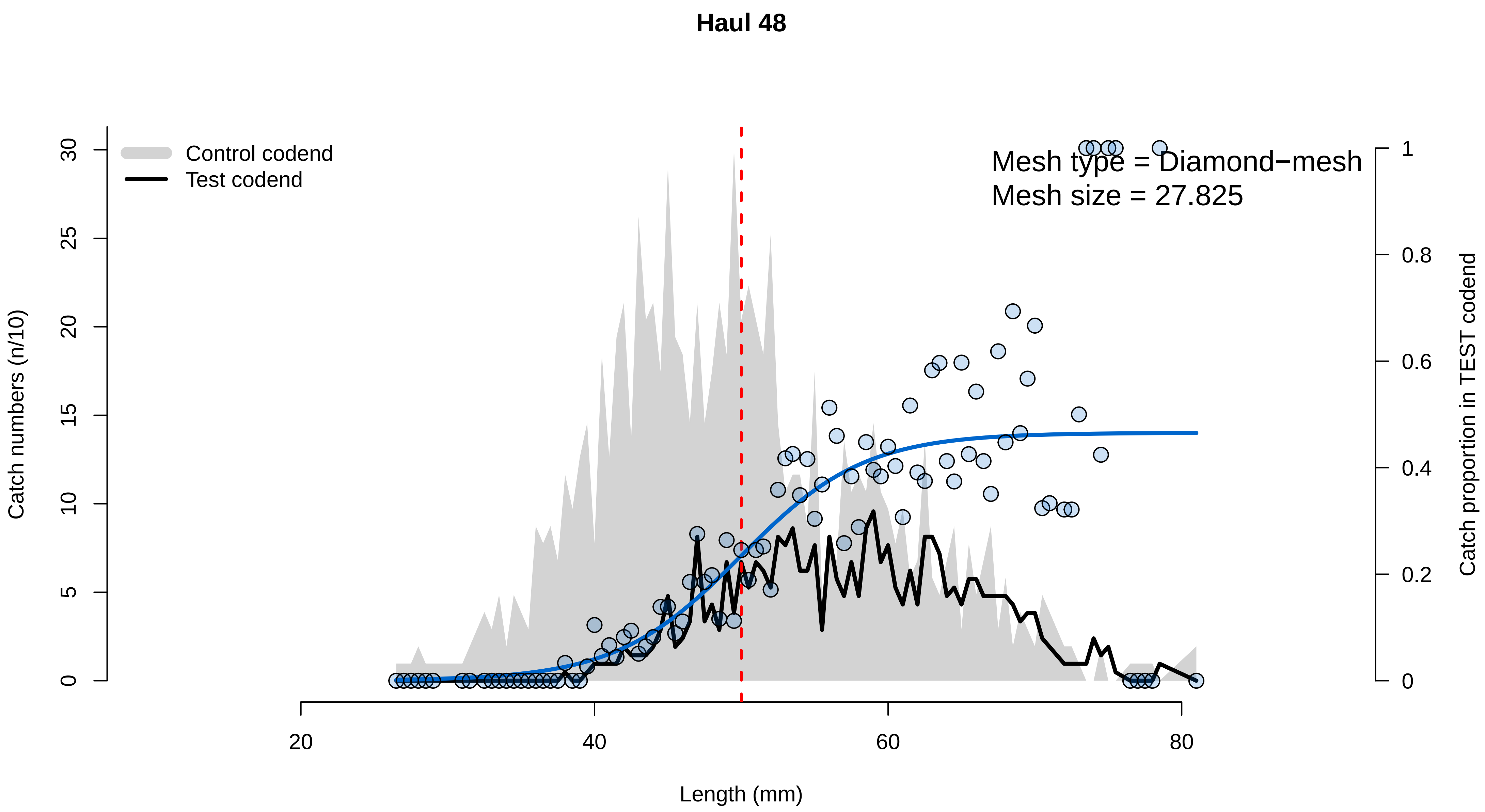

Haul 48
Control codend
Test codend
Mesh type = Diamond−mesh
Mesh size = 27.825
Catch numbers (n/10)
Catch proportion in TEST codend
Length (mm)
0
5
10
15
20
25
30
0
0.2
0.4
0.6
0.8
1
20
40
60
80

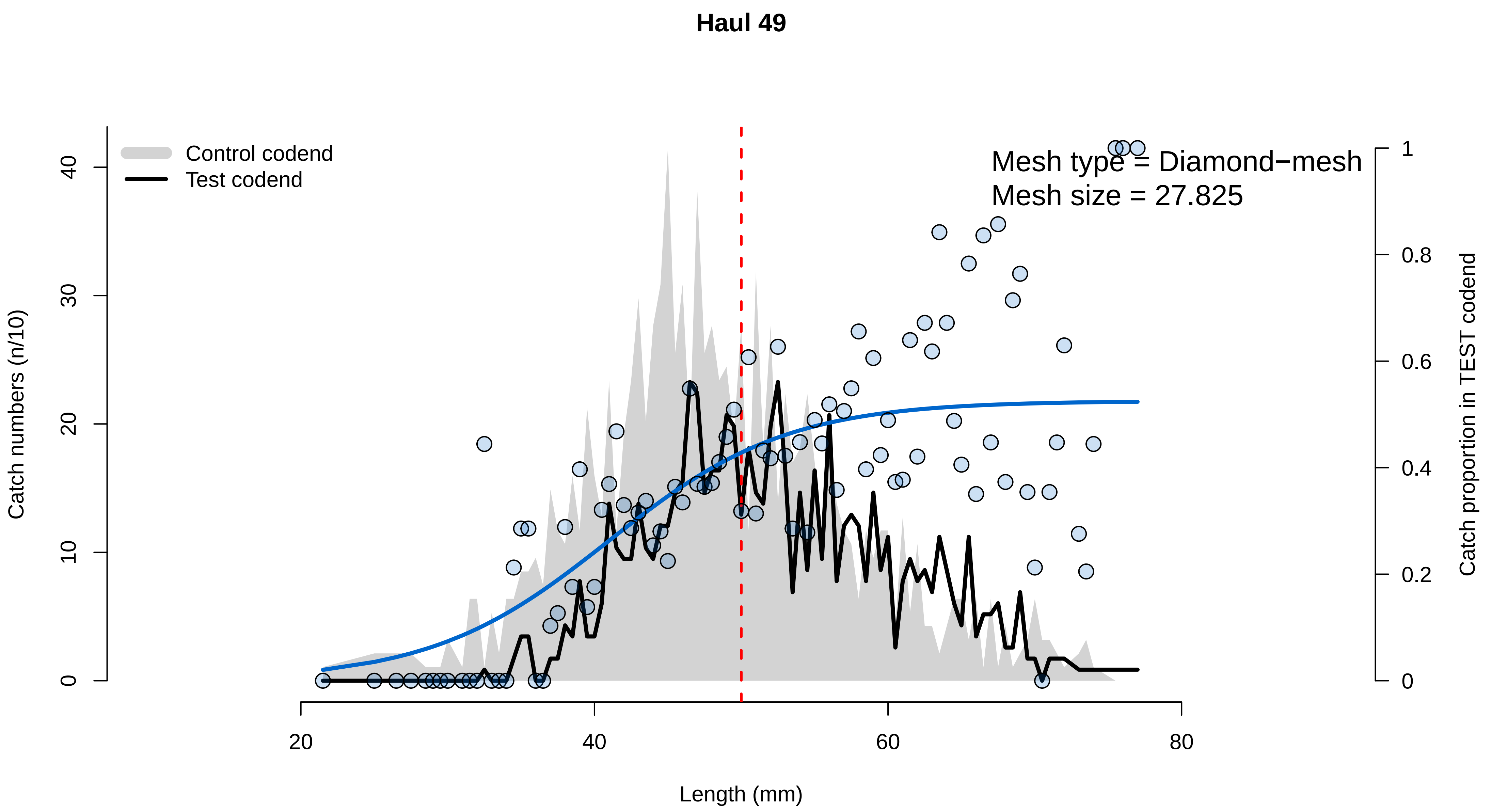
Haul 49
Control codend
Test codend
Mesh type = Diamond−mesh
Mesh size = 27.825
Catch numbers (n/10)
Catch proportion in TEST codend
Length (mm)
0
10
20
30
40
0
0.2
0.4
0.6
0.8
1
20
40
60
80

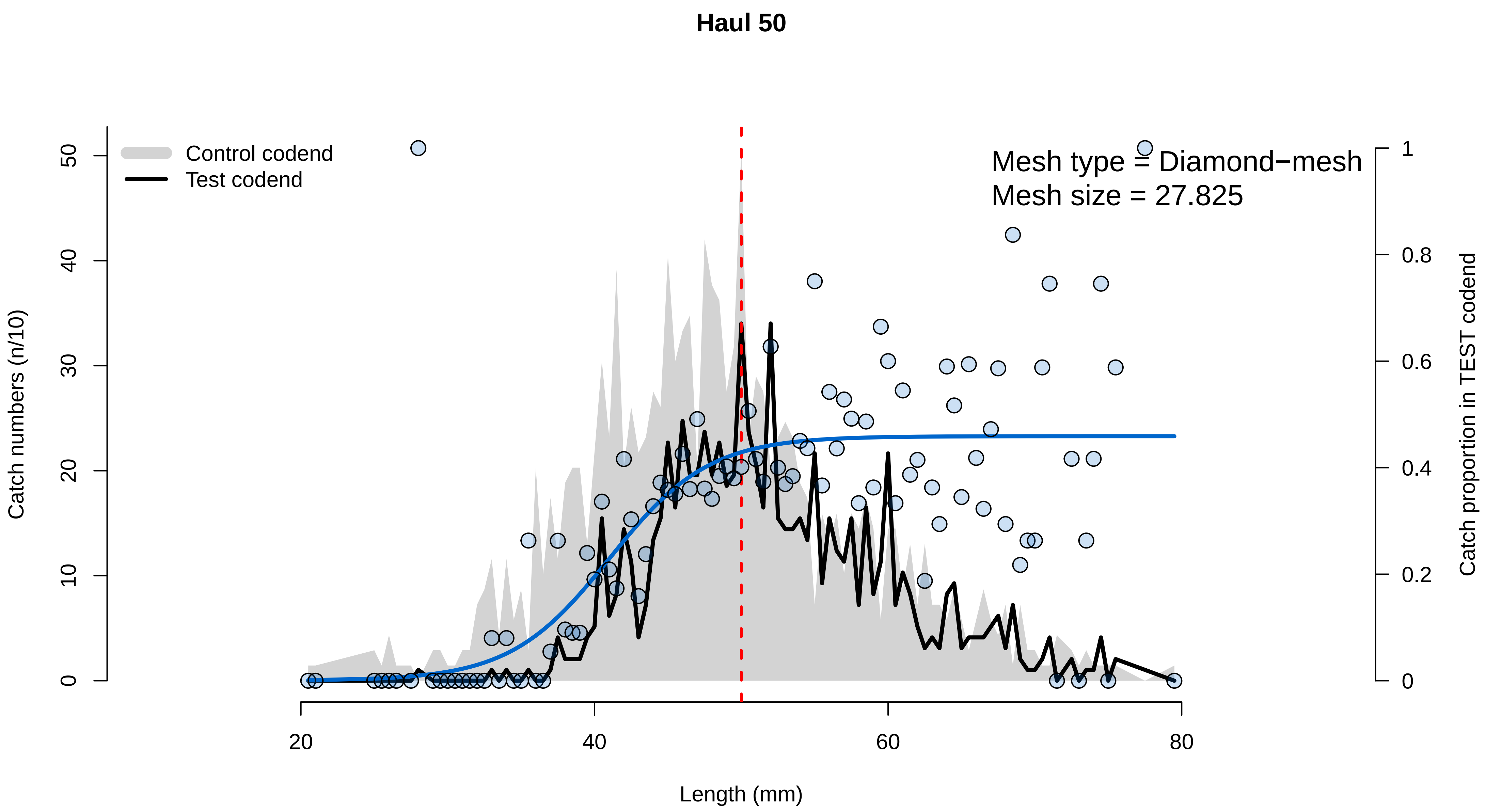

Haul 50
Control codend
Test codend
Mesh type = Diamond−mesh
Mesh size = 27.825
Catch numbers (n/10)
Catch proportion in TEST codend
Length (mm)
0
10
20
30
40
50
0
0.2
0.4
0.6
0.8
1
20
40
60
80

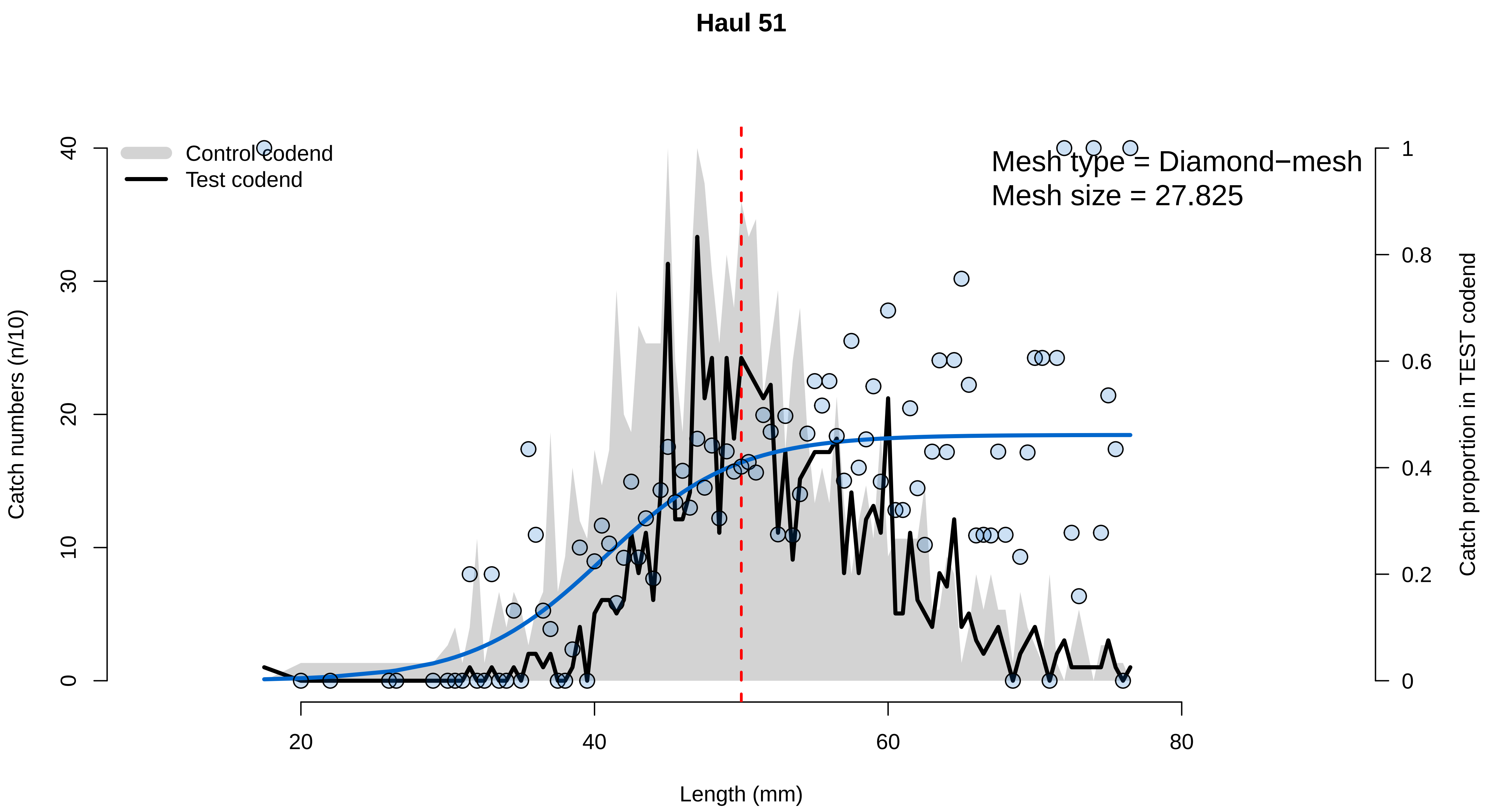

Haul 51
Control codend
Test codend
Mesh type = Diamond−mesh
Mesh size = 27.825
Catch numbers (n/10)
0
10
20
30
40
Catch proportion in TEST codend
0
0.2
0.4
0.6
0.8
1
20
40
60
80
Length (mm)

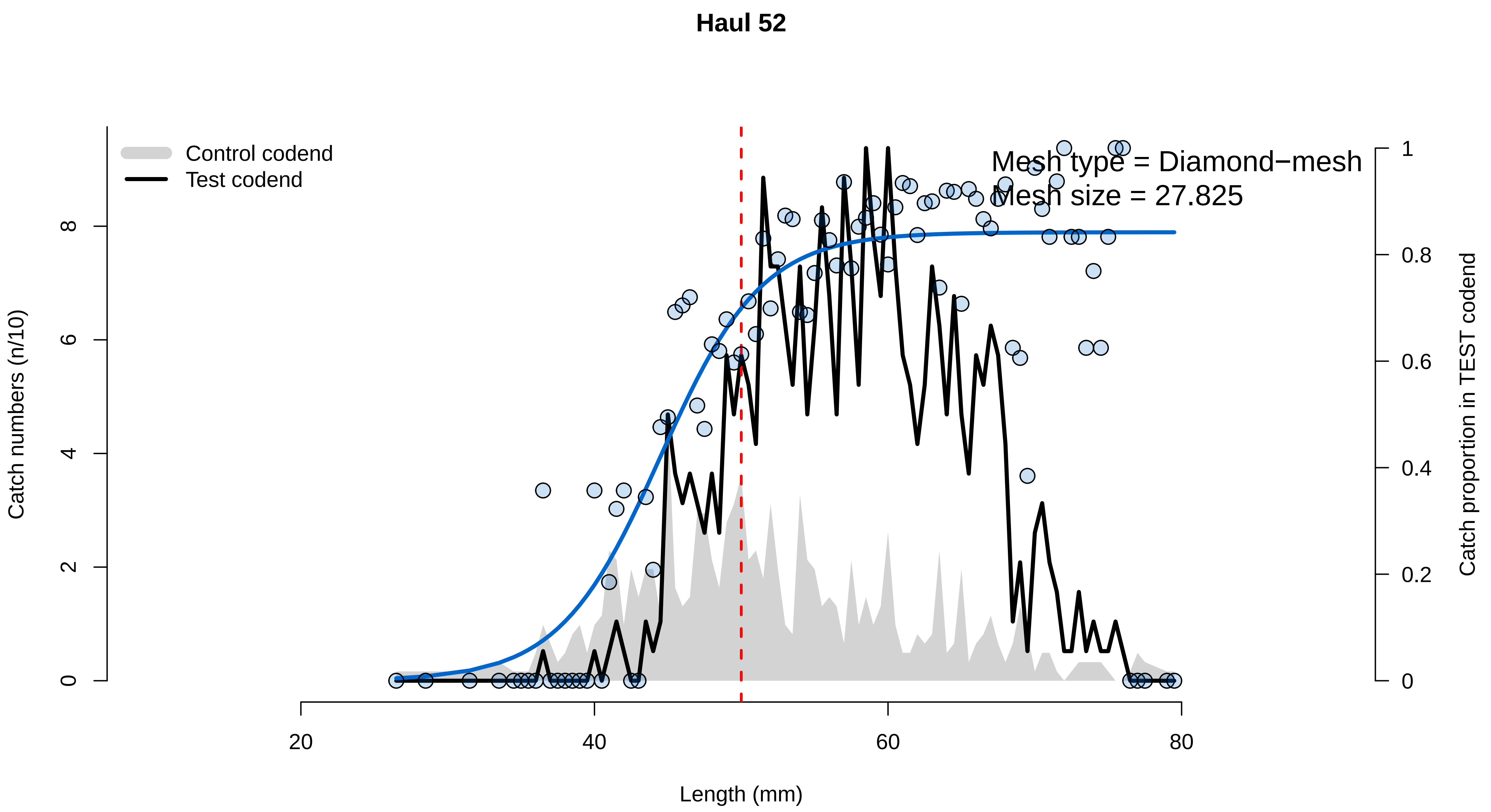

Haul 52
Control codend
Test codend
Mesh type = Diamond−mesh
Mesh size = 27.825
Catch numbers (n/10)
0
2
4
6
8
Catch proportion in TEST codend
0
0.2
0.4
0.6
0.8
1
20
40
60
80
Length (mm)

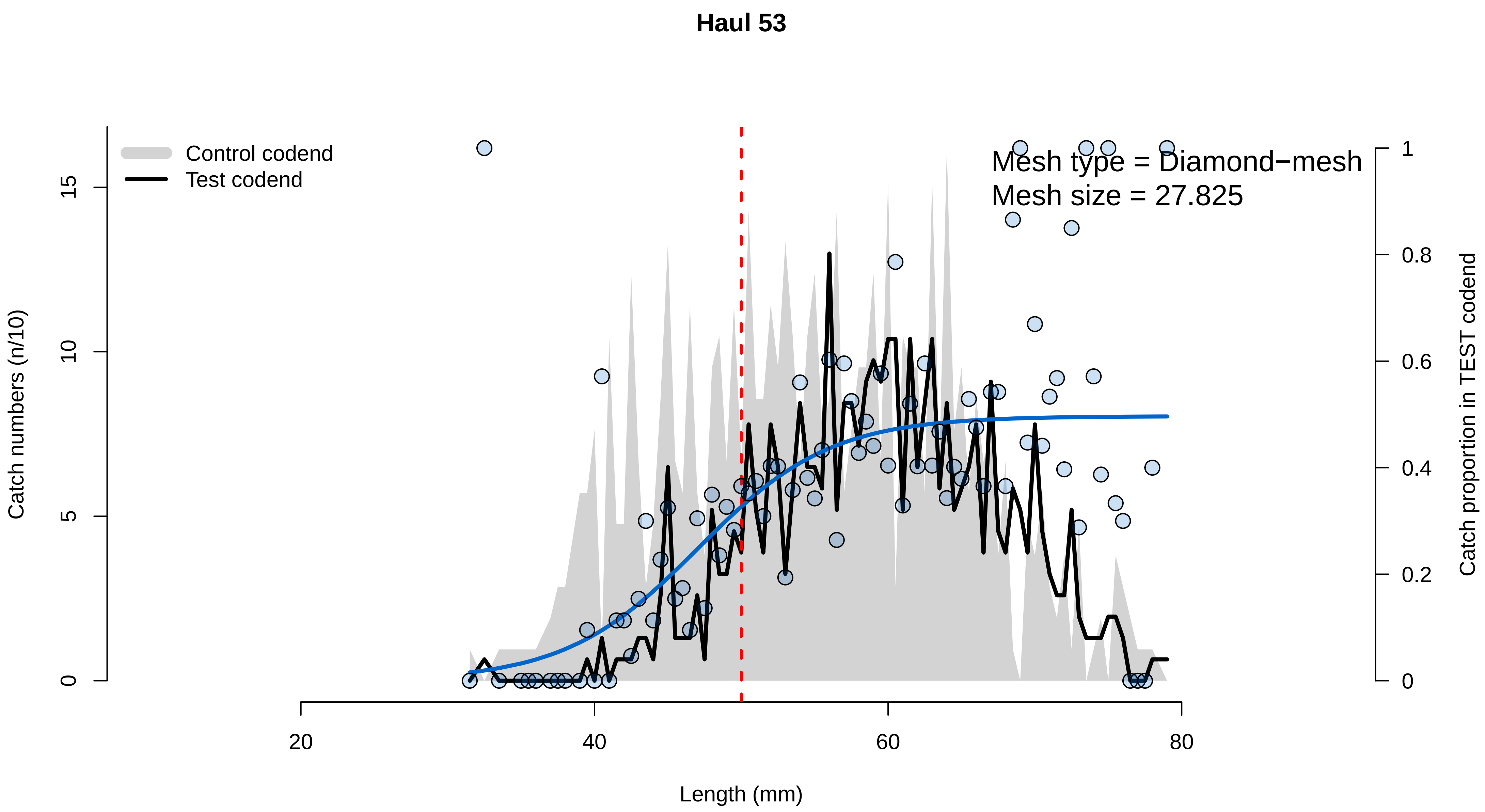
Haul 53
Control codend
Test codend
Mesh type = Diamond−mesh
Mesh size = 27.825
Catch numbers (n/10)
Catch proportion in TEST codend
Length (mm)
0
5
10
15
20
40
60
80
0
0.2
0.4
0.6
0.8
1

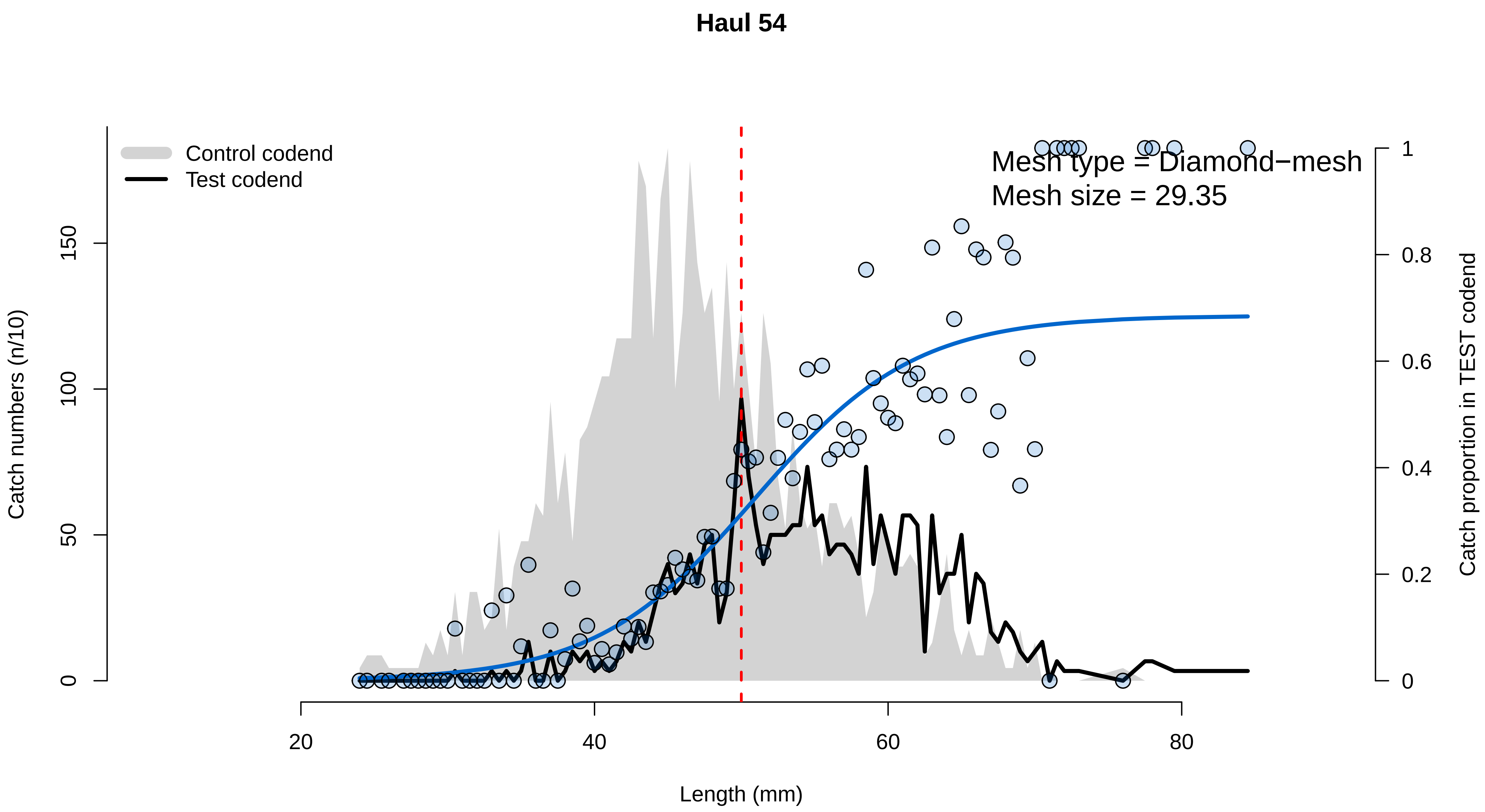

Haul 54
Control codend
Test codend
Mesh type = Diamond−mesh
Mesh size = 29.35
Catch numbers (n/10)
0
50
100
150
Catch proportion in TEST codend
0
0.2
0.4
0.6
0.8
1
20
40
60
80
Length (mm)

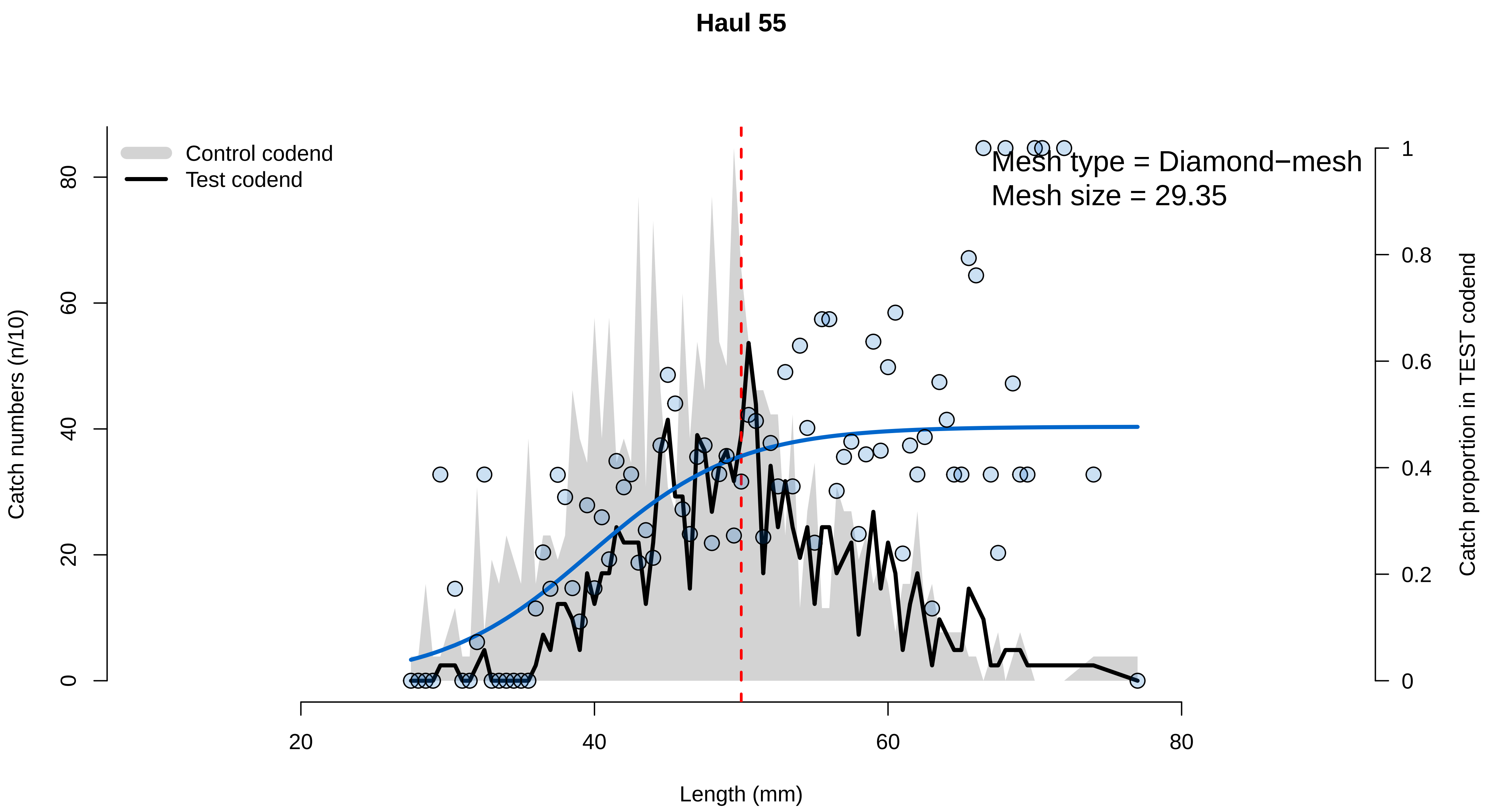

Haul 55
Control codend
Test codend
Mesh type = Diamond−mesh
Mesh size = 29.35
Catch numbers (n/10)
Catch proportion in TEST codend
Length (mm)
0
20
40
60
80
0
0.2
0.4
0.6
0.8
1
20
40
60
80

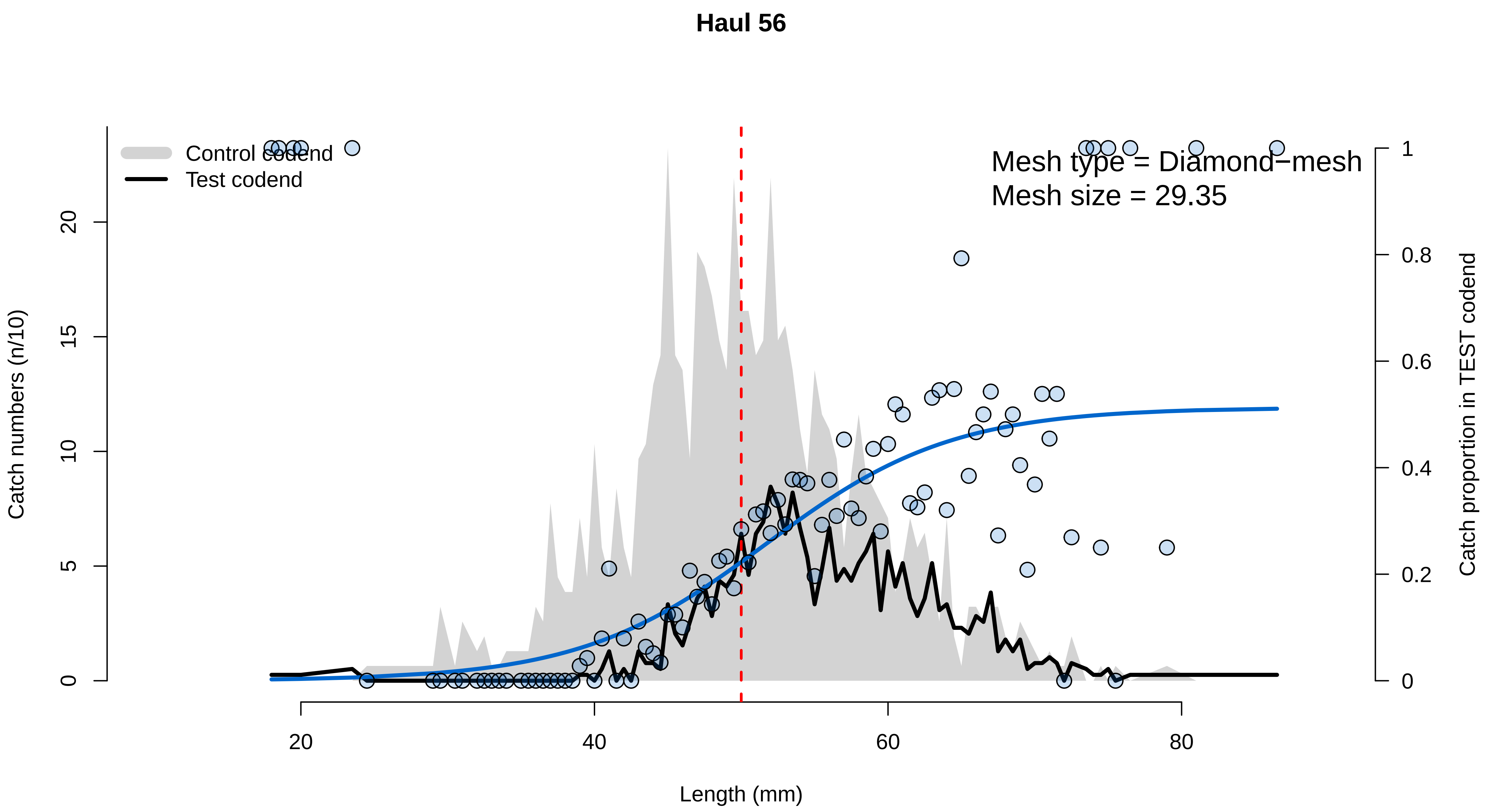

Haul 56
Control codend
Test codend
Mesh type = Diamond−mesh
Mesh size = 29.35
Catch numbers (n/10)
0
5
10
15
20
Catch proportion in TEST codend
0
0.2
0.4
0.6
0.8
1
20
40
60
80
Length (mm)

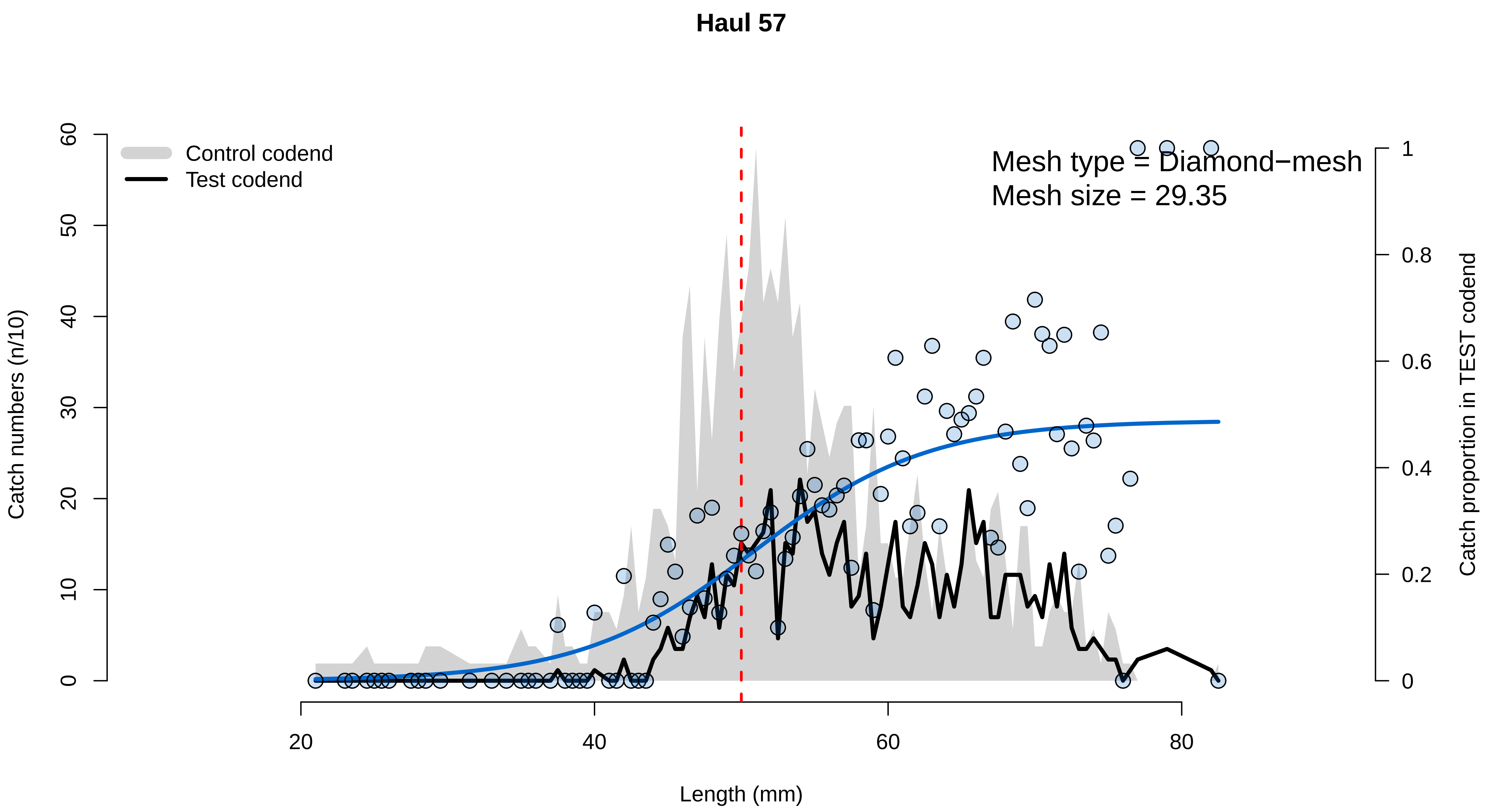

Haul 57
Control codend
Test codend
Mesh type = Diamond−mesh
Mesh size = 29.35
Catch numbers (n/10)
Catch proportion in TEST codend
Length (mm)
0
10
20
30
40
50
60
0
0.2
0.4
0.6
0.8
1
20
40
60
80

Haul 58

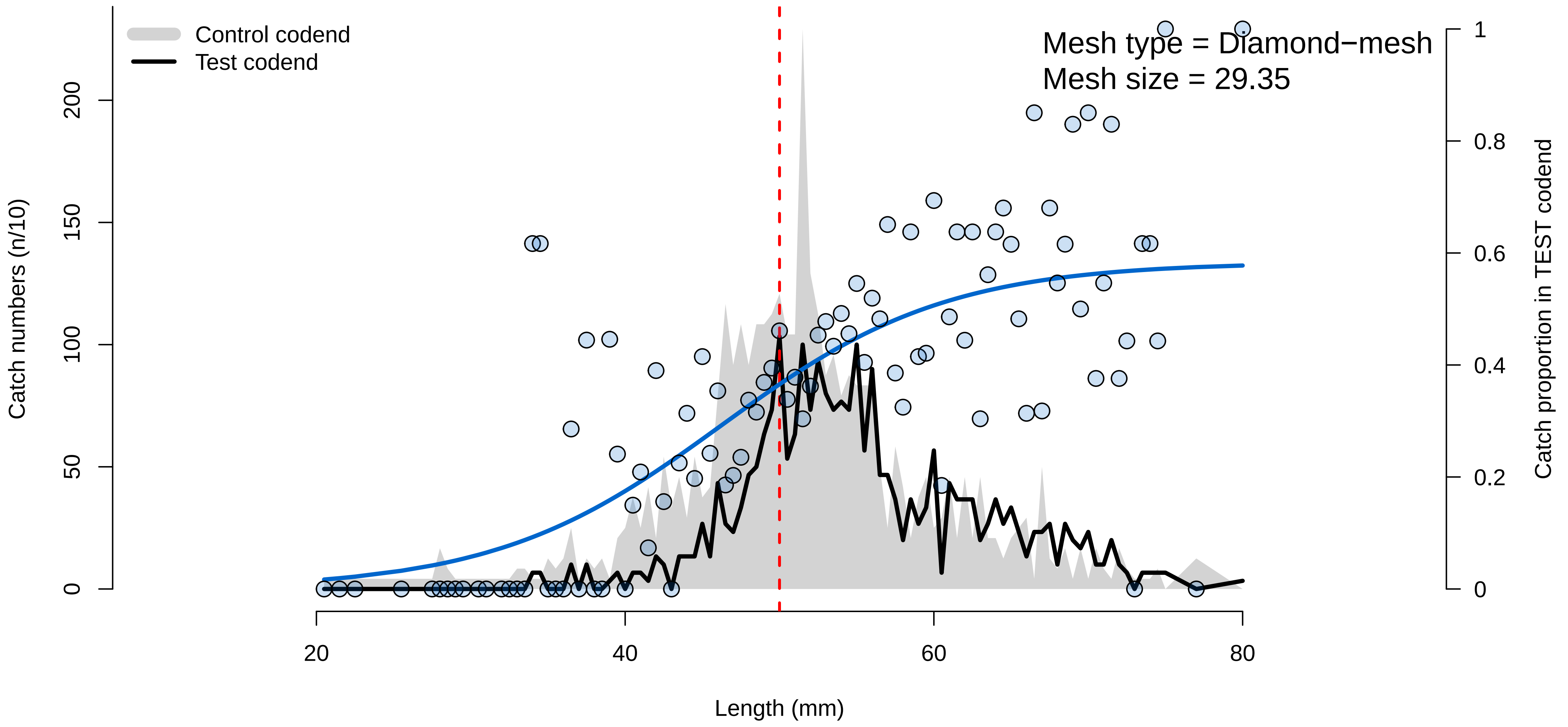

Control codend
Test codend
Mesh type = Diamond−mesh
Mesh size = 29.35
Catch numbers (n/10)
0
50
100
150
200
Catch proportion in TEST codend
0
0.2
0.4
0.6
0.8
1
20
40
60
80
Length (mm)

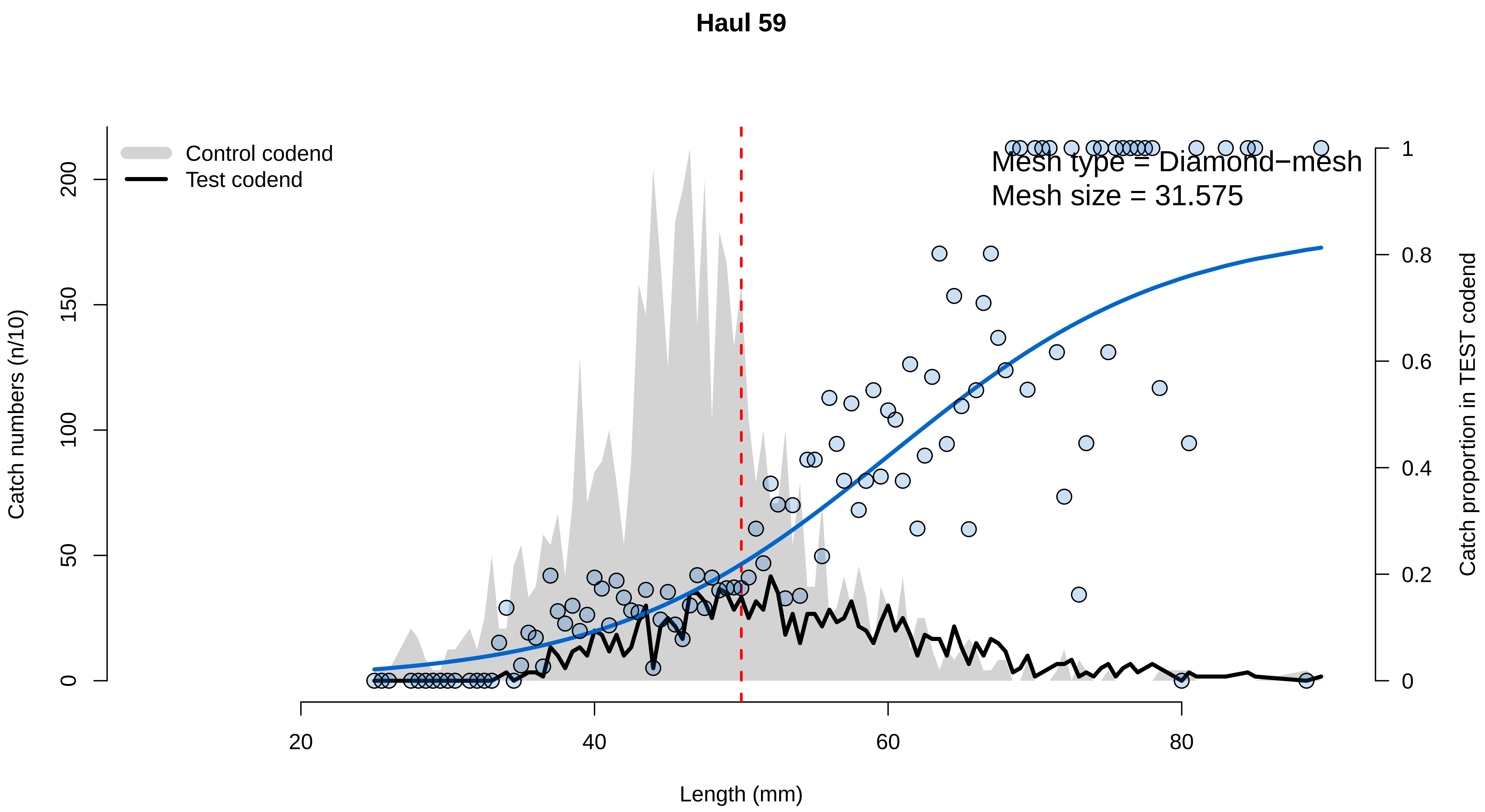

Haul 59
Control codend
Test codend
Mesh type = Diamond−mesh
Mesh size = 31.575
Catch numbers (n/10)
0
50
100
150
200
20
40
60
80
Length (mm)
0
0.2
0.4
0.6
0.8
1
Catch proportion in TEST codend

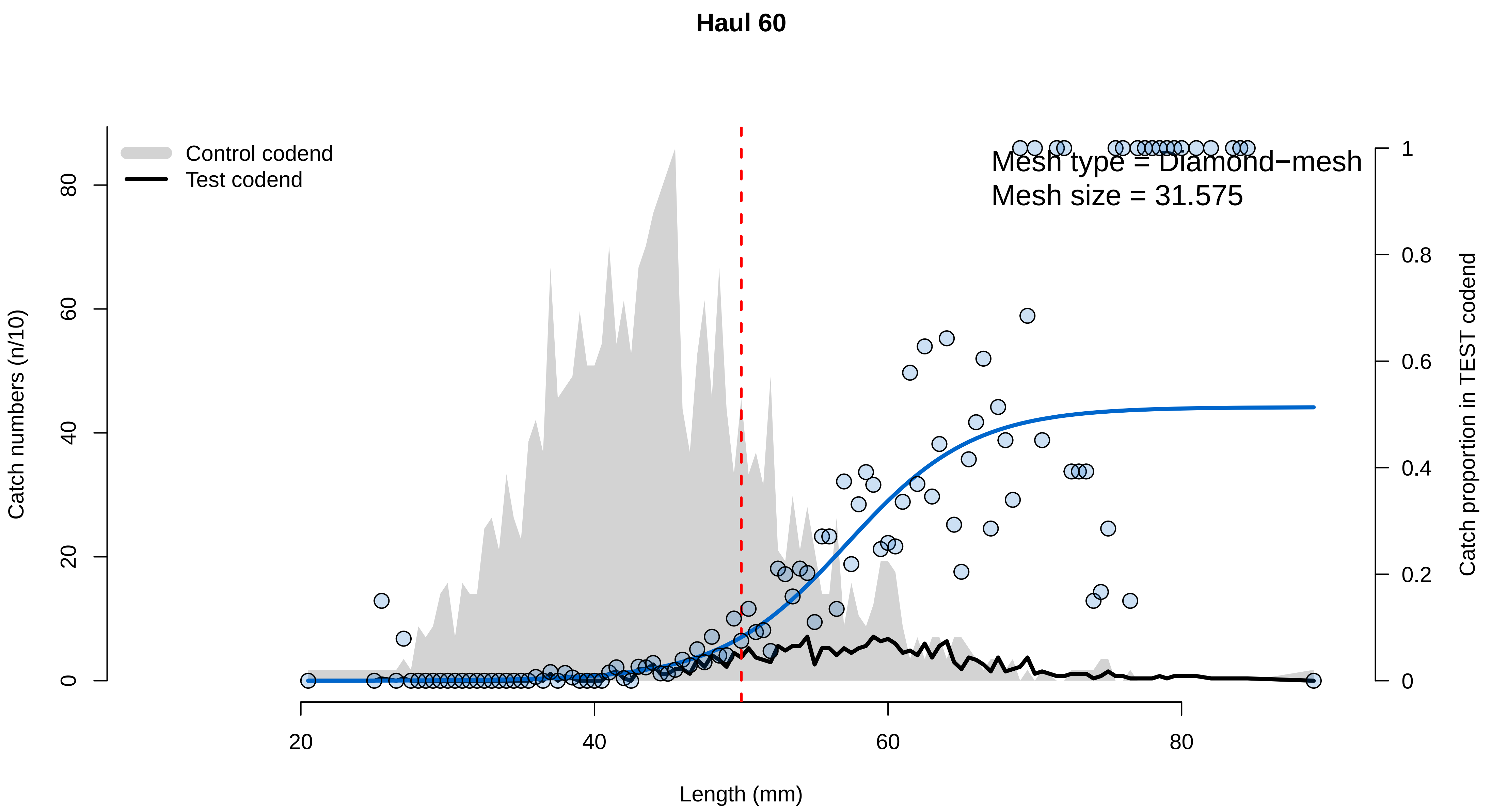
Haul 60
Control codend
Test codend
Mesh type = Diamond−mesh
Mesh size = 31.575
Catch numbers (n/10)
0
20
40
60
80
Catch proportion in TEST codend
0
0.2
0.4
0.6
0.8
1
Length (mm)
20
40
60
80

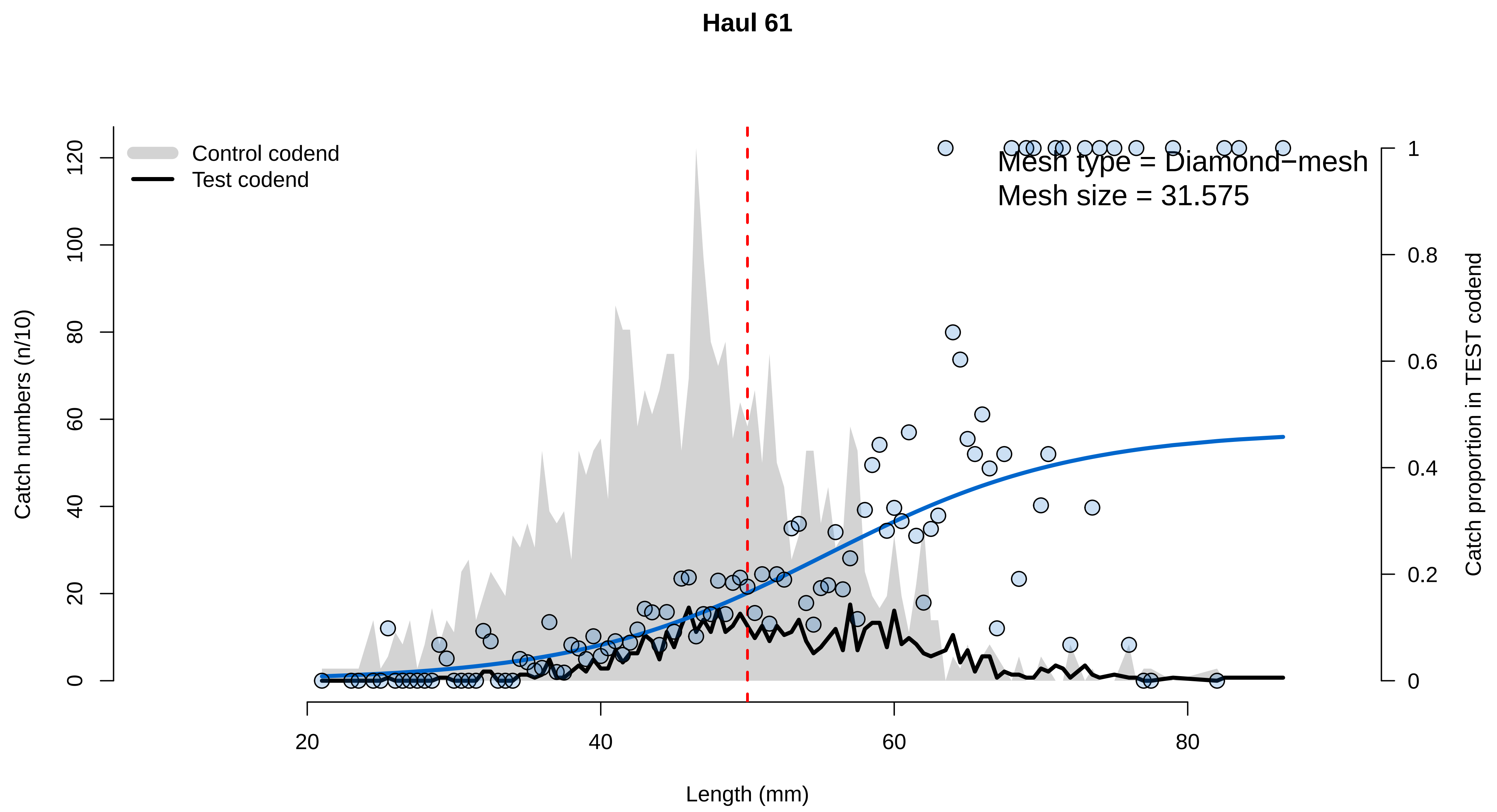

Haul 61
Control codend
Test codend
Mesh type = Diamond−mesh
Mesh size = 31.575
Catch numbers (n/10)
0
20
40
60
80
100
120
Catch proportion in TEST codend
0
0.2
0.4
0.6
0.8
1
20
40
60
80
Length (mm)

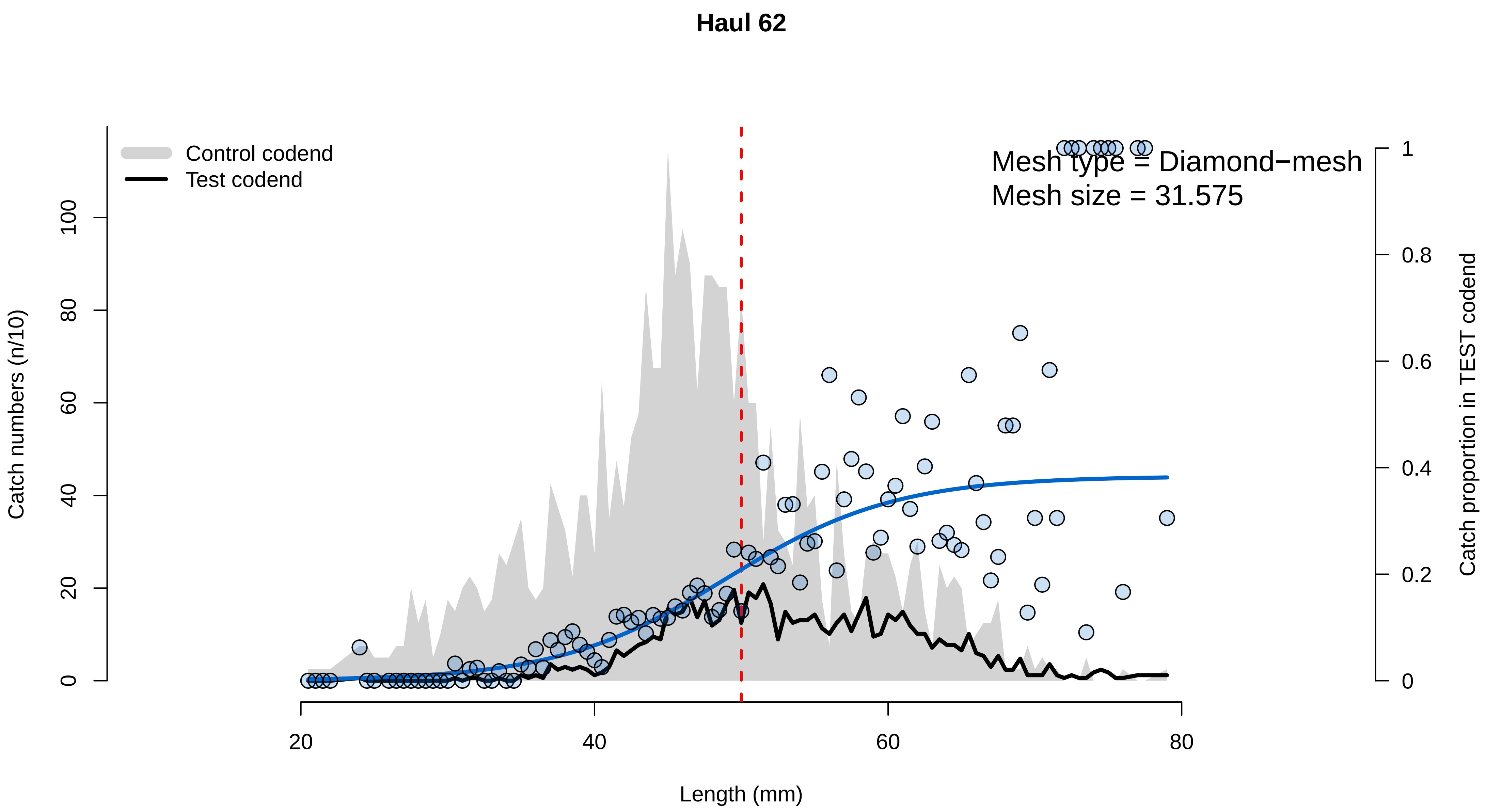

Haul 62
Control codend
Test codend
Mesh type = Diamond−mesh
Mesh size = 31.575
Catch numbers (n/10)
0
20
40
60
80
100
Catch proportion in TEST codend
0
0.2
0.4
0.6
0.8
1
20
40
60
80
Length (mm)

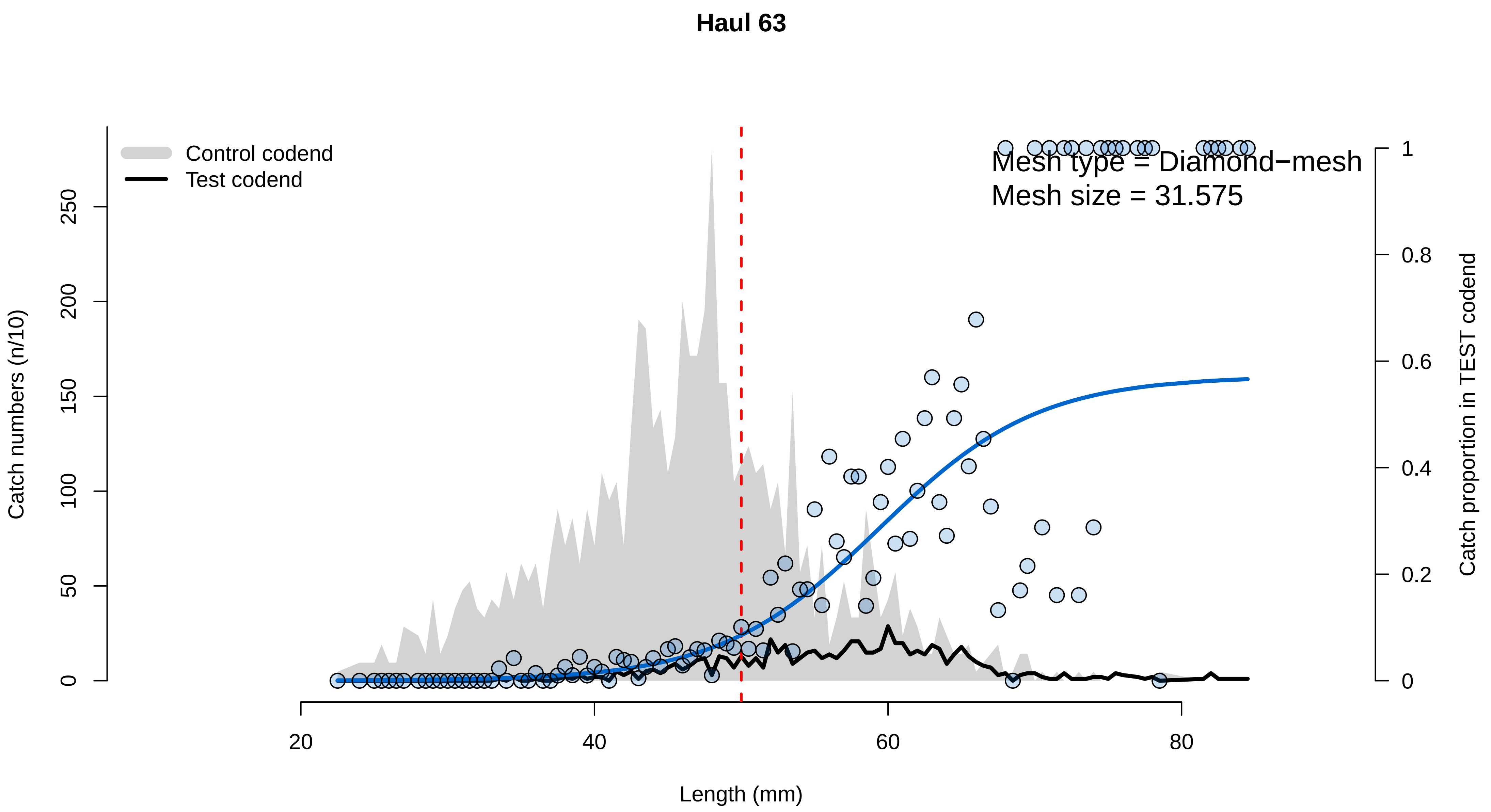

Haul 63
Control codend
Test codend
Mesh type = Diamond−mesh
Mesh size = 31.575
Catch numbers (n/10)
0
50
100
150
200
250
Catch proportion in TEST codend
0
0.2
0.4
0.6
0.8
1
20
40
60
80
Length (mm)

Haul 64

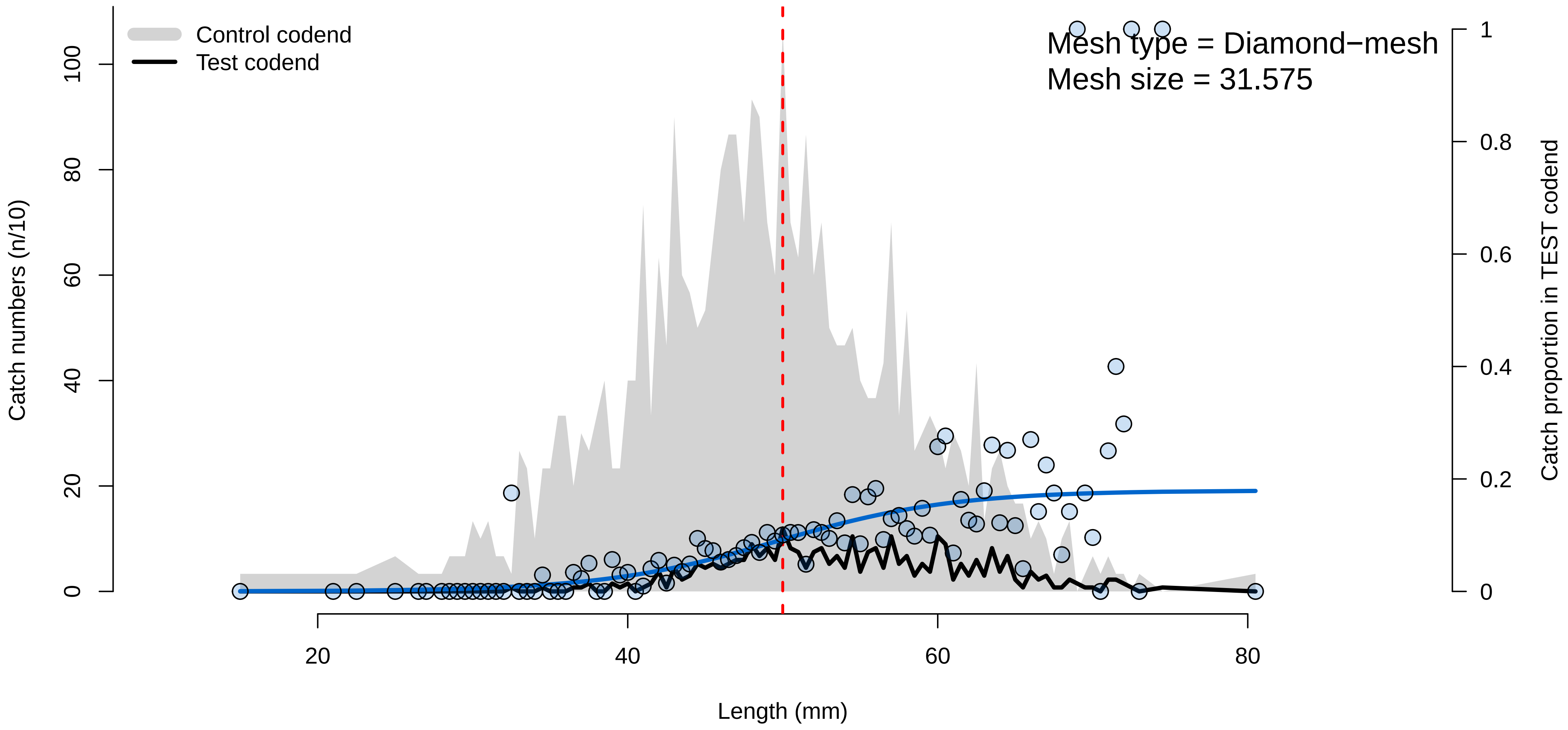

Control codend
Test codend
Mesh type = Diamond−mesh
Mesh size = 31.575
Catch numbers (n/10)
0
20
40
60
80
100
Catch proportion in TEST codend
0
0.2
0.4
0.6
0.8
1
20
40
60
80
Length (mm)

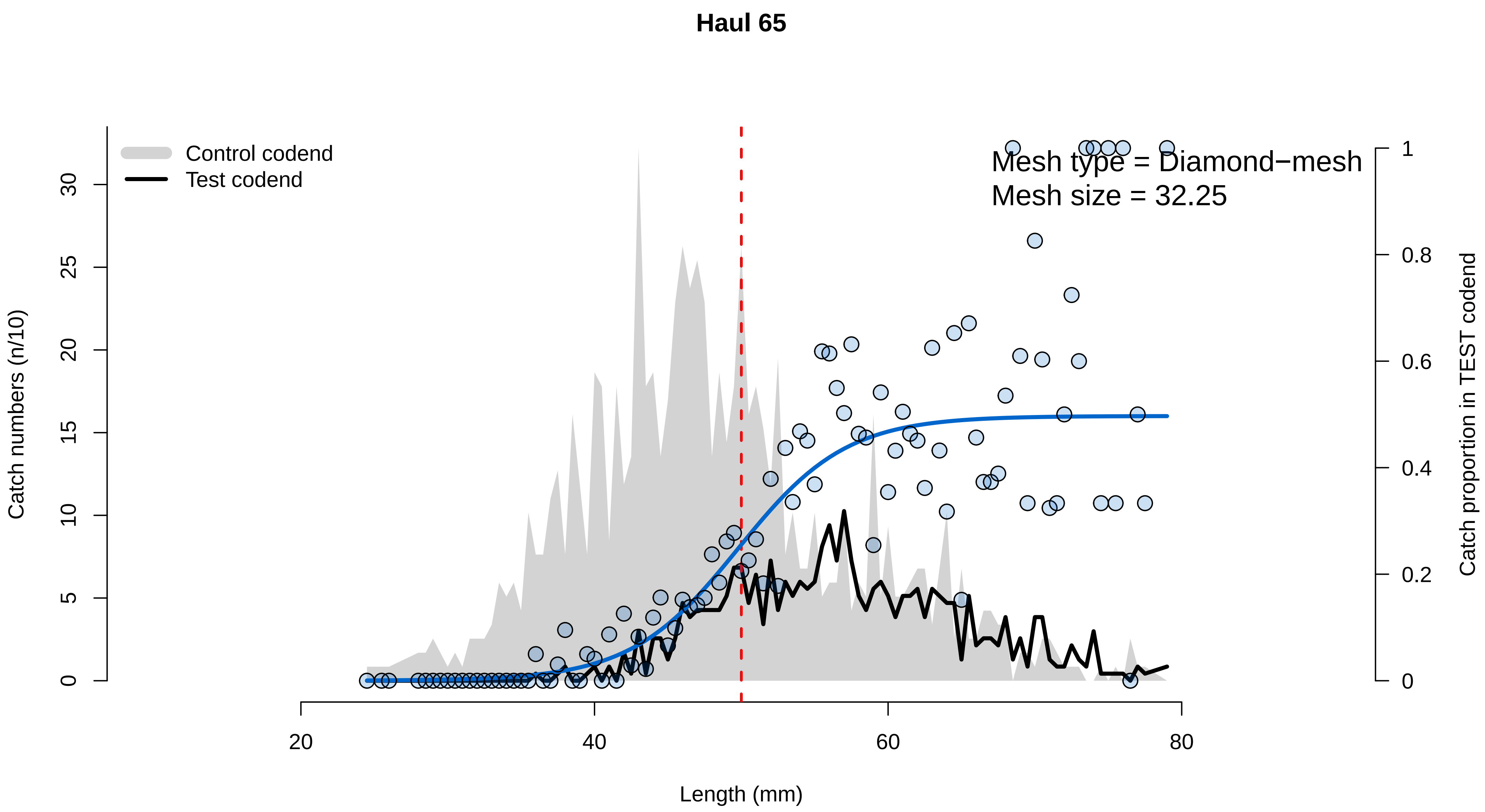

Haul 65
Control codend
Test codend
Mesh type = Diamond−mesh
Mesh size = 32.25
Catch numbers (n/10)
0
5
10
15
20
25
30
Catch proportion in TEST codend
0
0.2
0.4
0.6
0.8
1
20
40
60
80
Length (mm)

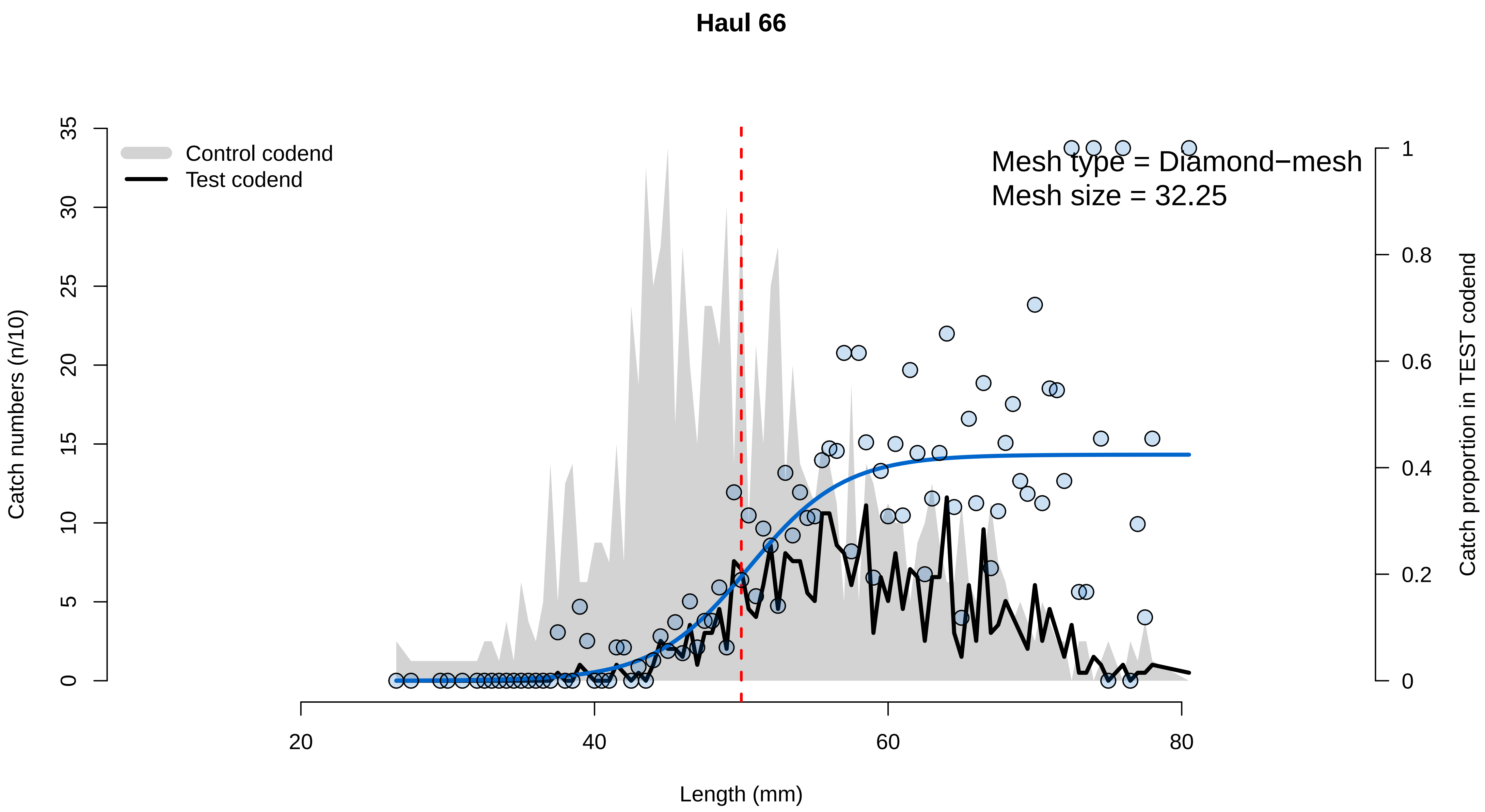

Haul 66
Control codend
Test codend
Mesh type = Diamond−mesh
Mesh size = 32.25
Catch numbers (n/10)
Catch proportion in TEST codend
Length (mm)
0
5
10
15
20
25
30
35
20
40
60
80
0
0.2
0.4
0.6
0.8
1

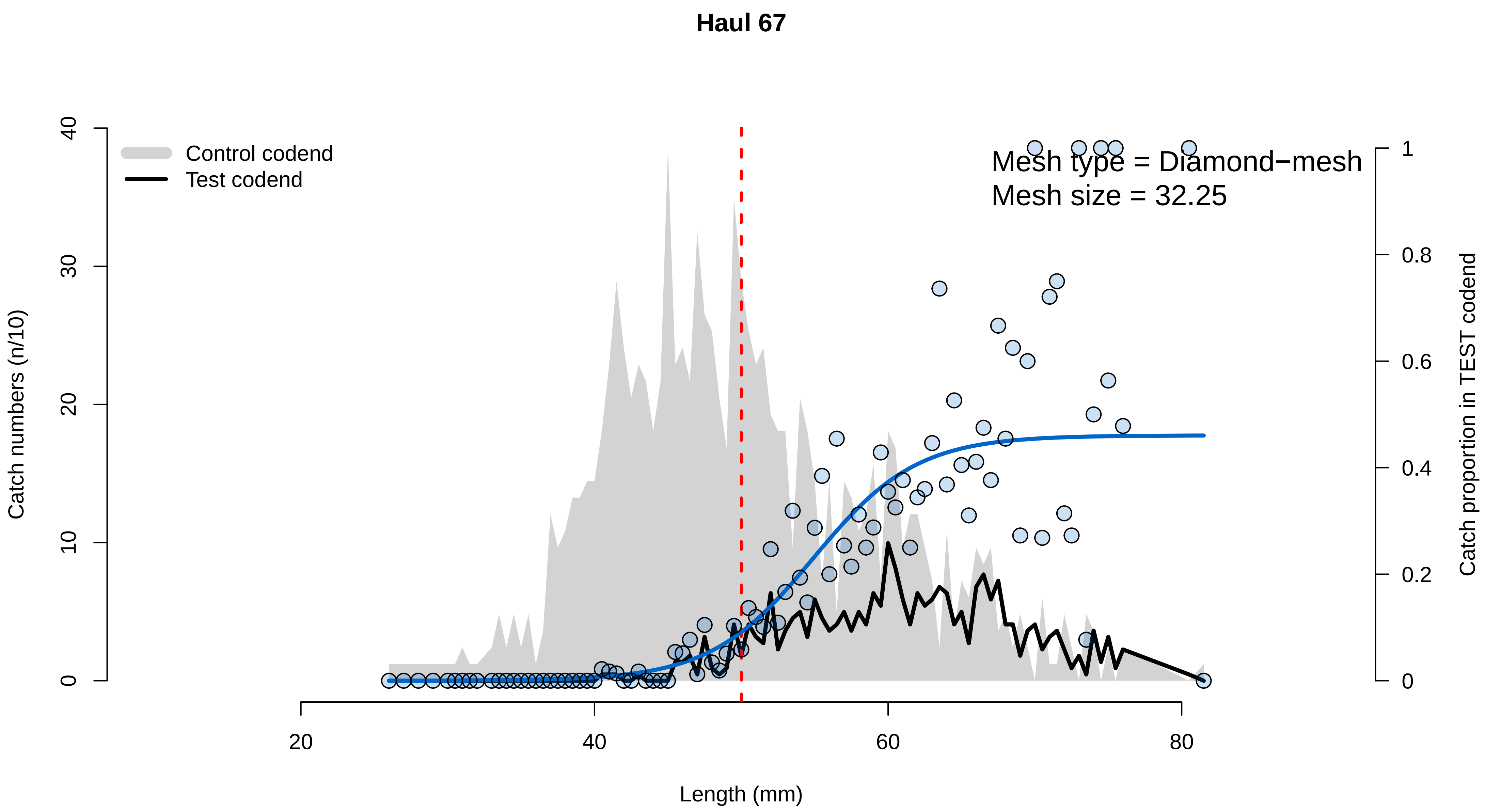
Haul 67
Control codend
Test codend
Mesh type = Diamond−mesh
Mesh size = 32.25
Catch numbers (n/10)
0
10
20
30
40
Catch proportion in TEST codend
0
0.2
0.4
0.6
0.8
1
20
40
60
80
Length (mm)

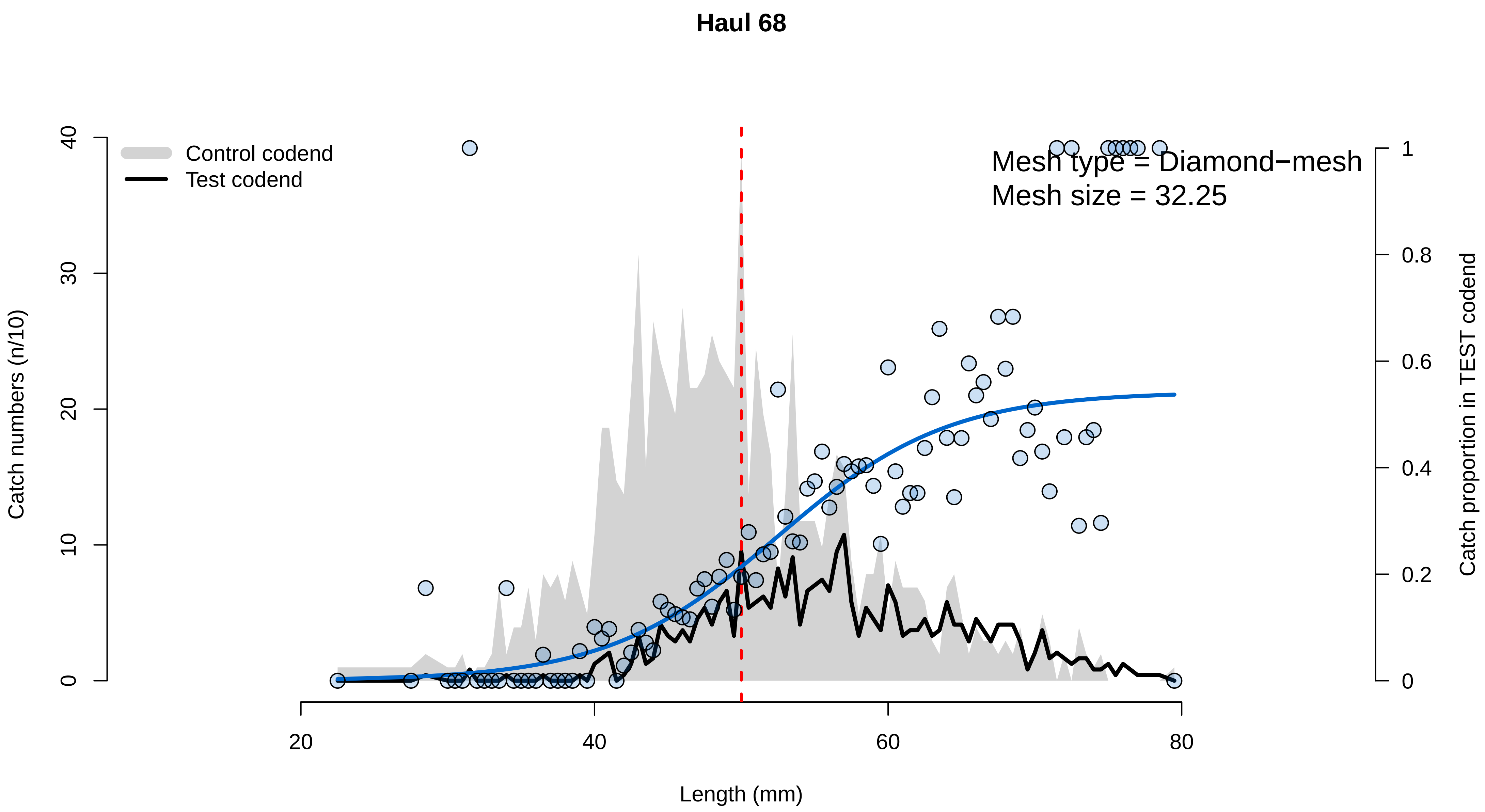

Haul 68
Control codend
Test codend
Mesh type = Diamond−mesh
Mesh size = 32.25
Catch numbers (n/10)
Catch proportion in TEST codend
Length (mm)
0
10
20
30
40
20
40
60
80
0
0.2
0.4
0.6
0.8
1

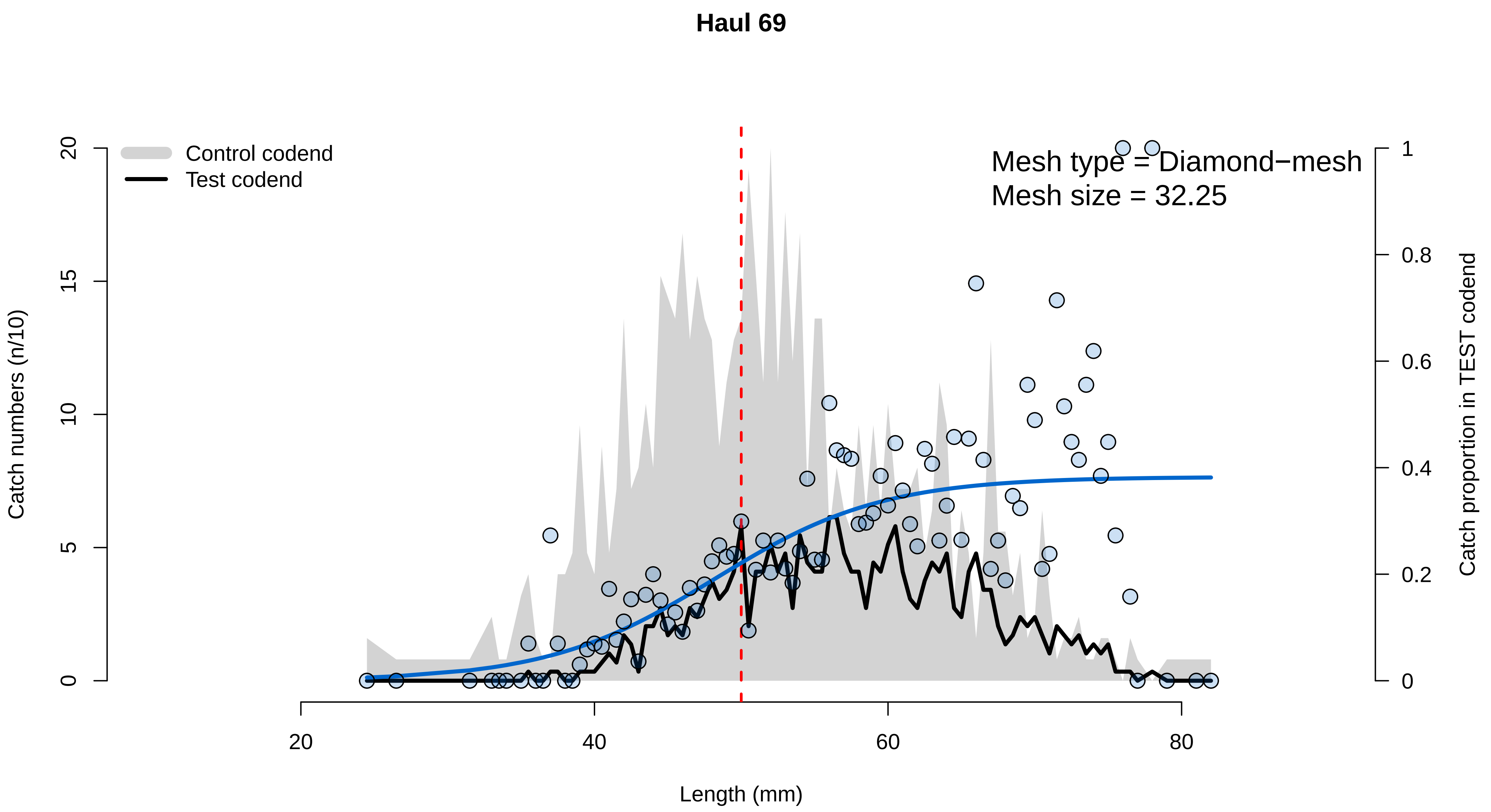

Haul 69
Control codend
Test codend
Mesh type = Diamond−mesh
Mesh size = 32.25
Catch numbers (n/10)
Catch proportion in TEST codend
Length (mm)
0
5
10
15
20
0
0.2
0.4
0.6
0.8
1
20
40
60
80

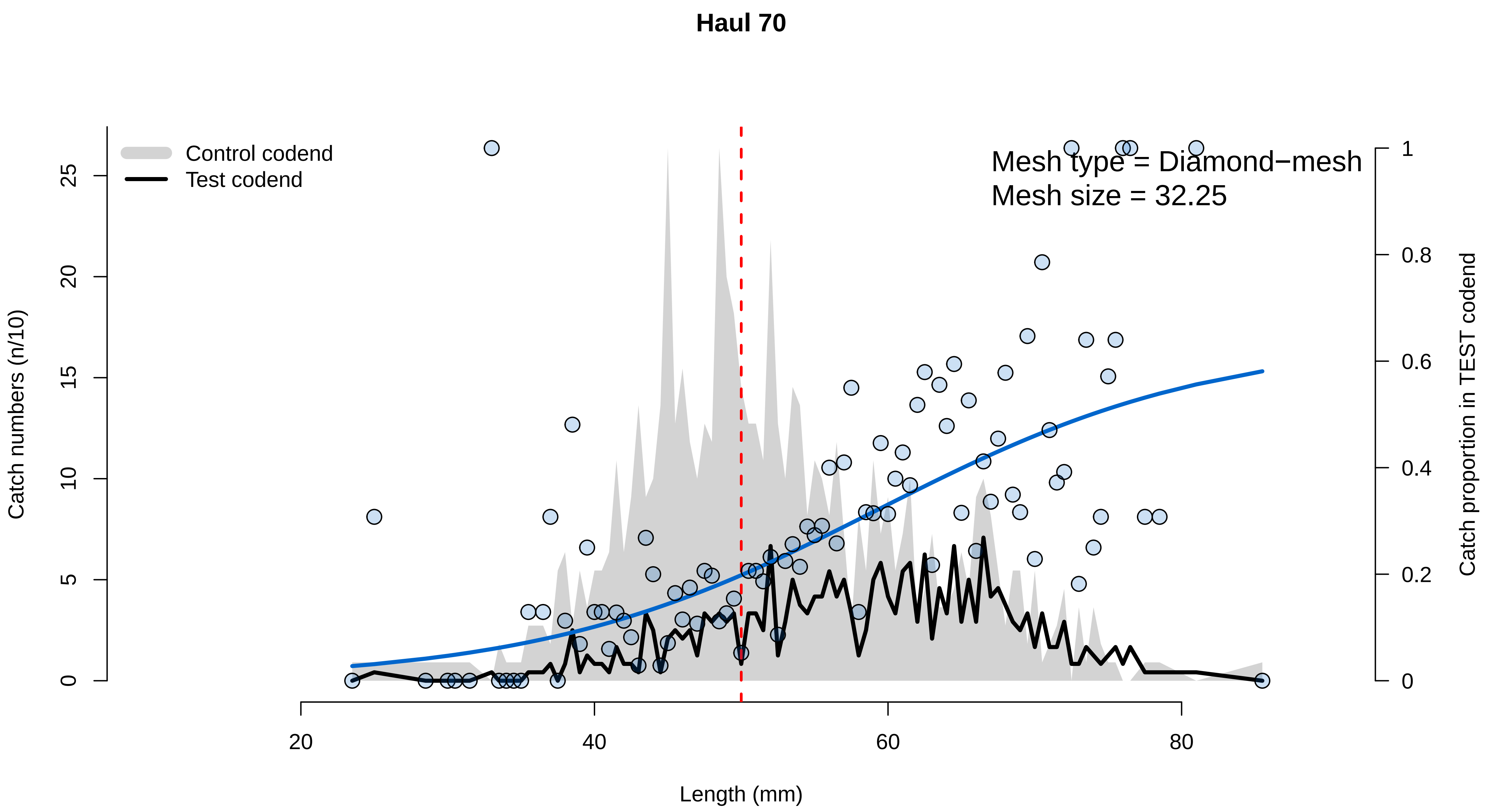

Haul 70
Control codend
Test codend
Mesh type = Diamond−mesh
Mesh size = 32.25
Catch numbers (n/10)
0
5
10
15
20
25
Catch proportion in TEST codend
0
0.2
0.4
0.6
0.8
1
20
40
60
80
Length (mm)

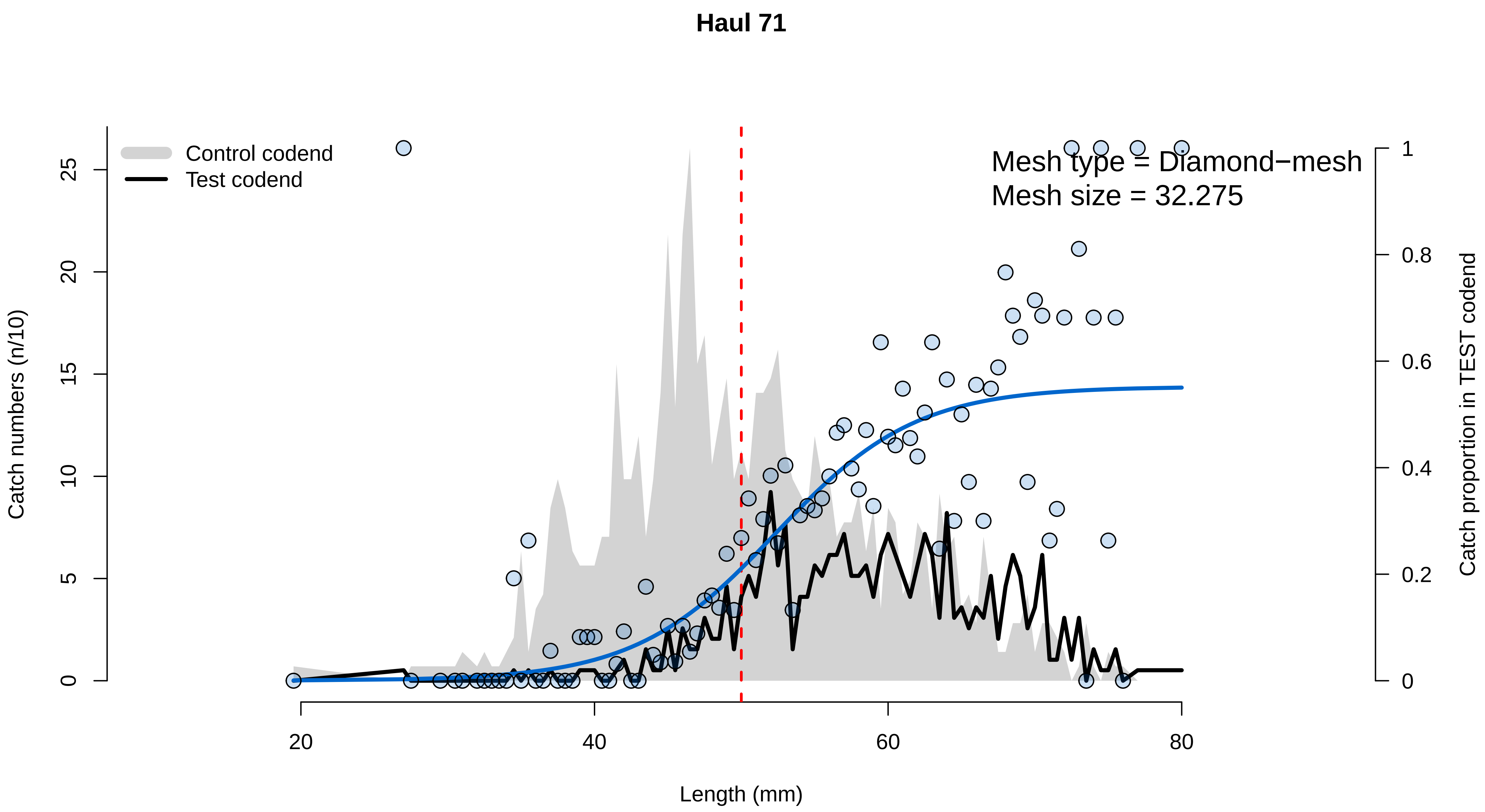

Haul 71
Control codend
Test codend
Mesh type = Diamond−mesh
Mesh size = 32.275
Catch numbers (n/10)
Catch proportion in TEST codend
Length (mm)
0
5
10
15
20
25
0
0.2
0.4
0.6
0.8
1
20
40
60
80

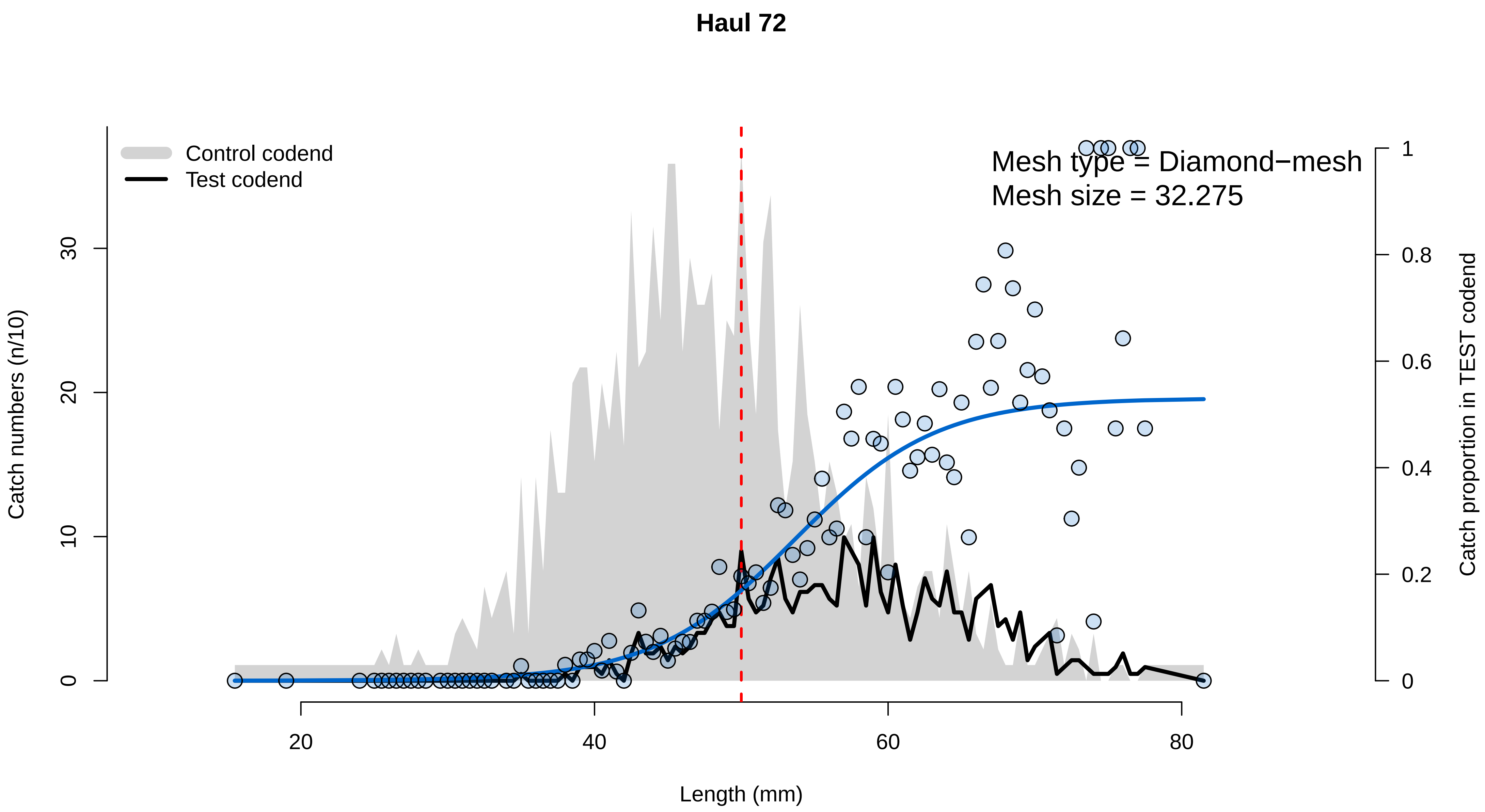
Haul 72
Control codend
Test codend
Mesh type = Diamond−mesh
Mesh size = 32.275
Catch numbers (n/10)
0
10
20
30
Catch proportion in TEST codend
0
0.2
0.4
0.6
0.8
1
20
40
60
80
Length (mm)

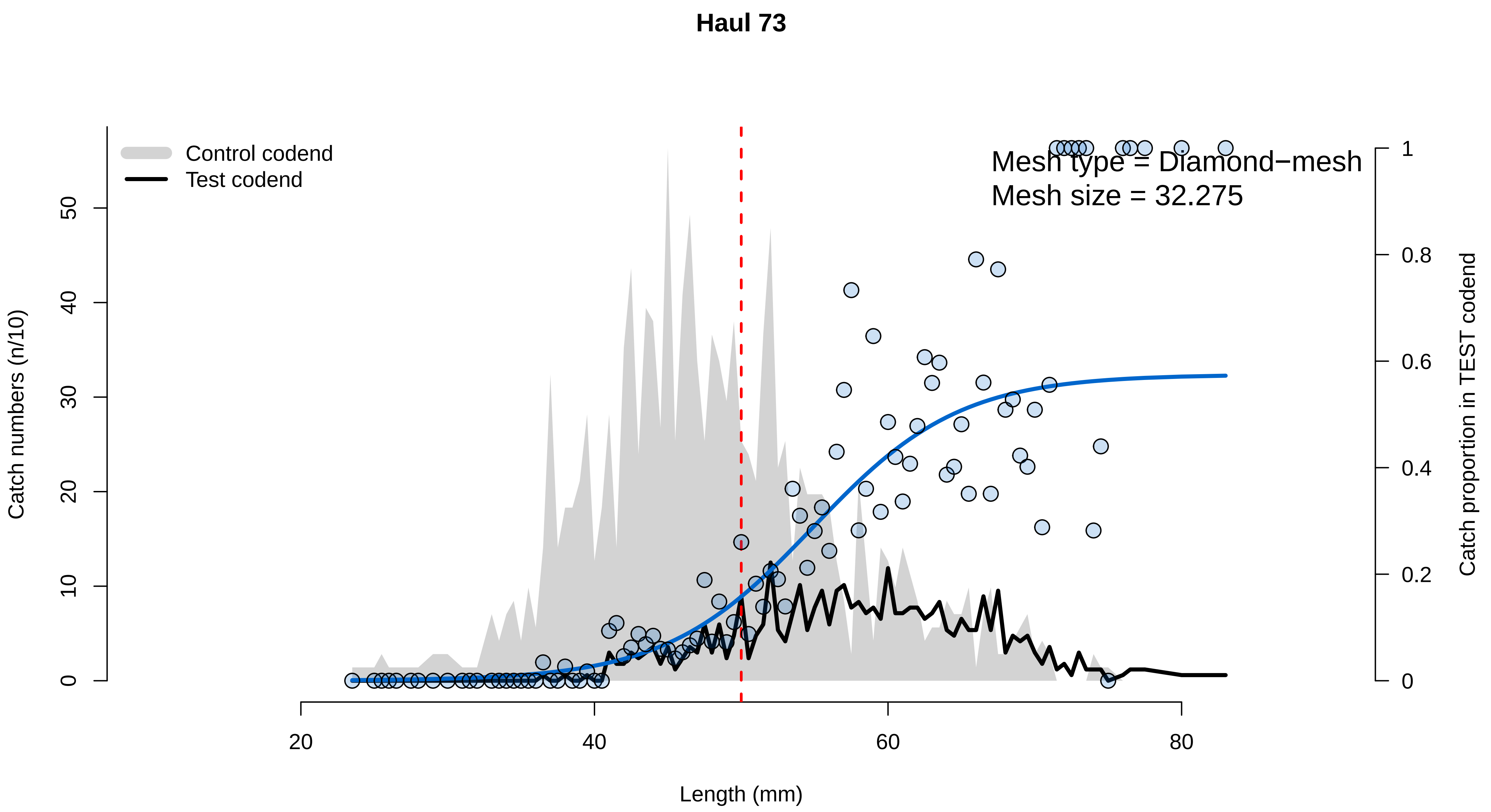
Haul 73
Control codend
Test codend
Mesh type = Diamond−mesh
Mesh size = 32.275
Catch numbers (n/10)
Catch proportion in TEST codend
Length (mm)
0
10
20
30
40
50
0
0.2
0.4
0.6
0.8
1
20
40
60
80

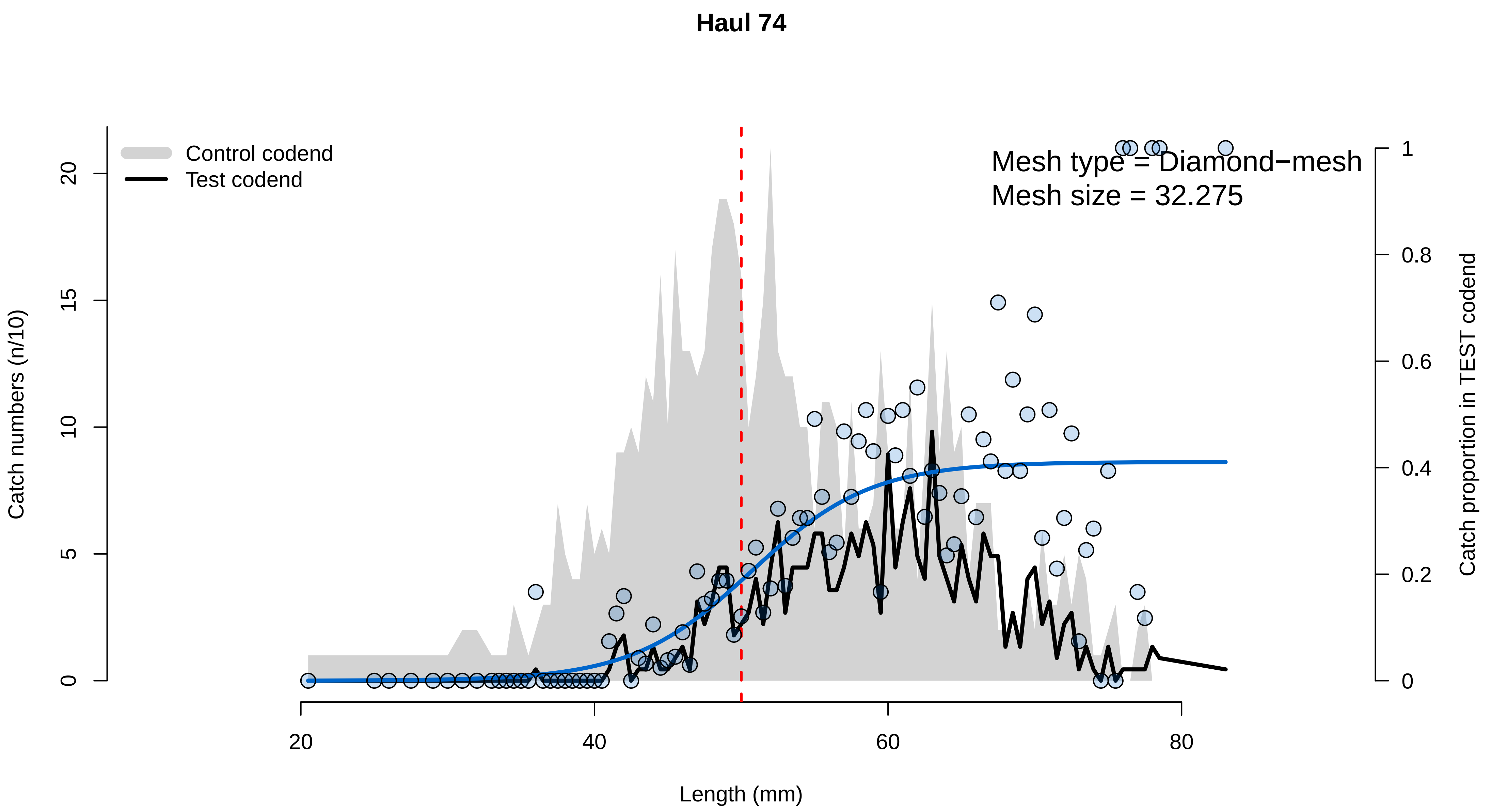
Haul 74
Control codend
Test codend
Mesh type = Diamond−mesh
Mesh size = 32.275
Catch numbers (n/10)
0
5
10
15
20
Catch proportion in TEST codend
0
0.2
0.4
0.6
0.8
1
20
40
60
80
Length (mm)

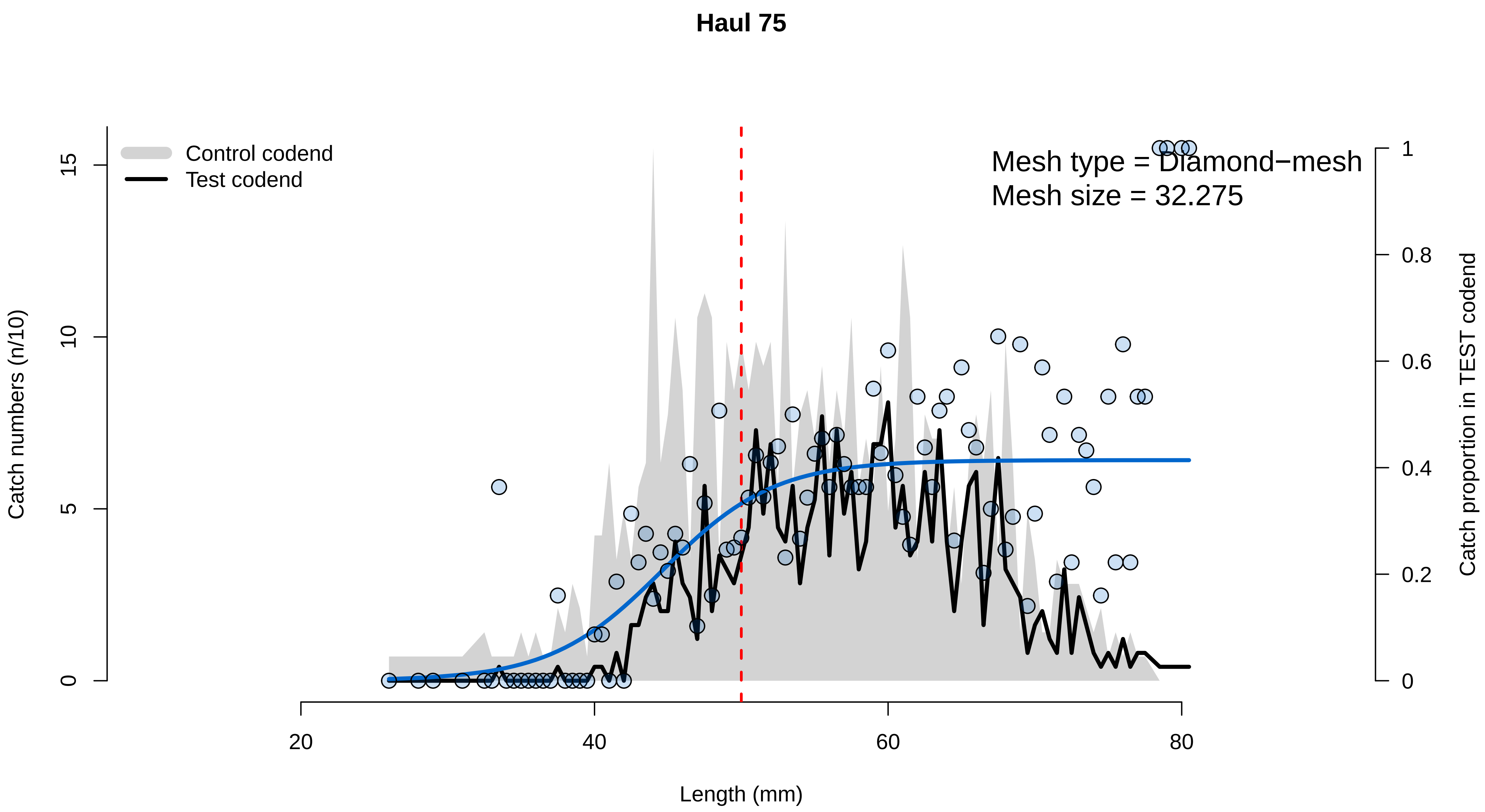

Haul 75
Control codend
Test codend
Mesh type = Diamond−mesh
Mesh size = 32.275
Catch numbers (n/10)
0
5
10
15
Catch proportion in TEST codend
0
0.2
0.4
0.6
0.8
1
20
40
60
80
Length (mm)

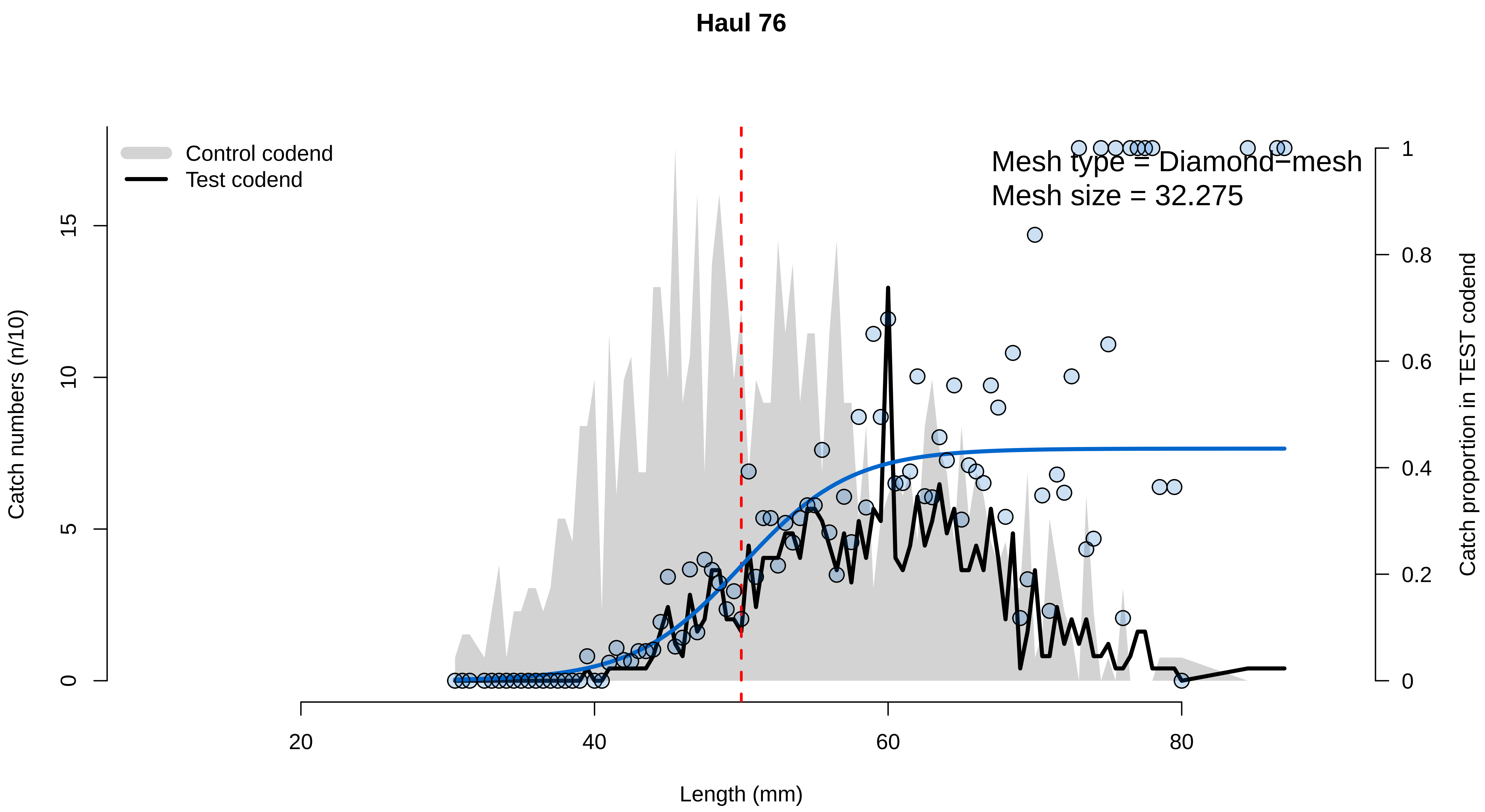
Haul 76
Control codend
Test codend
Mesh type = Diamond−mesh
Mesh size = 32.275
Catch numbers (n/10)
Catch proportion in TEST codend
Length (mm)
0
5
10
15
20
40
60
80
0
0.2
0.4
0.6
0.8
1

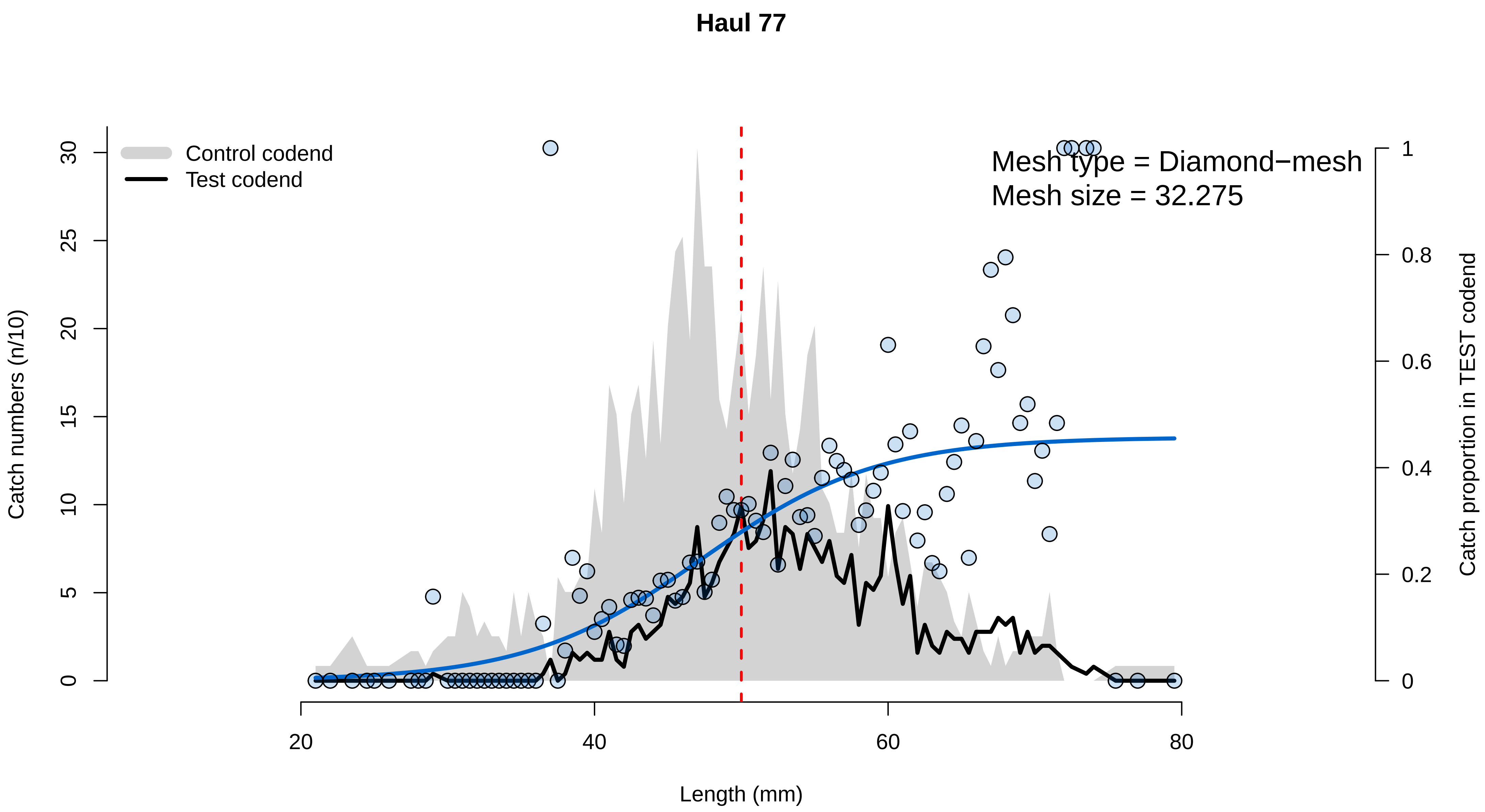
Haul 77
Control codend
Test codend
Mesh type = Diamond−mesh
Mesh size = 32.275
Catch numbers (n/10)
Catch proportion in TEST codend
Length (mm)
0
5
10
15
20
25
30
20
40
60
80
0
0.2
0.4
0.6
0.8
1

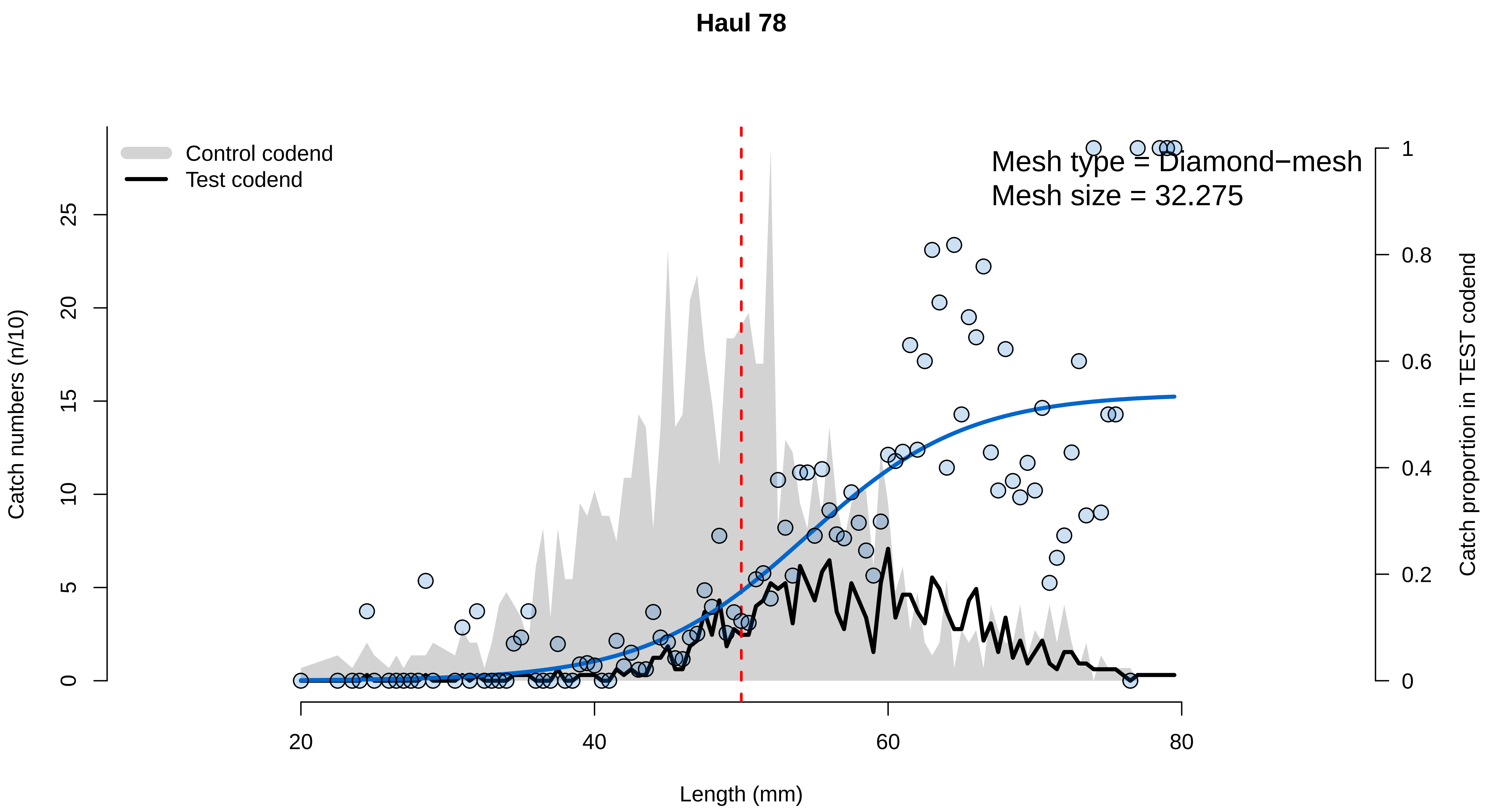

Haul 78
Control codend
Test codend
Mesh type = Diamond−mesh
Mesh size = 32.275
Catch numbers (n/10)
Catch proportion in TEST codend
Length (mm)
0
5
10
15
20
25
0
0.2
0.4
0.6
0.8
1
20
40
60
80

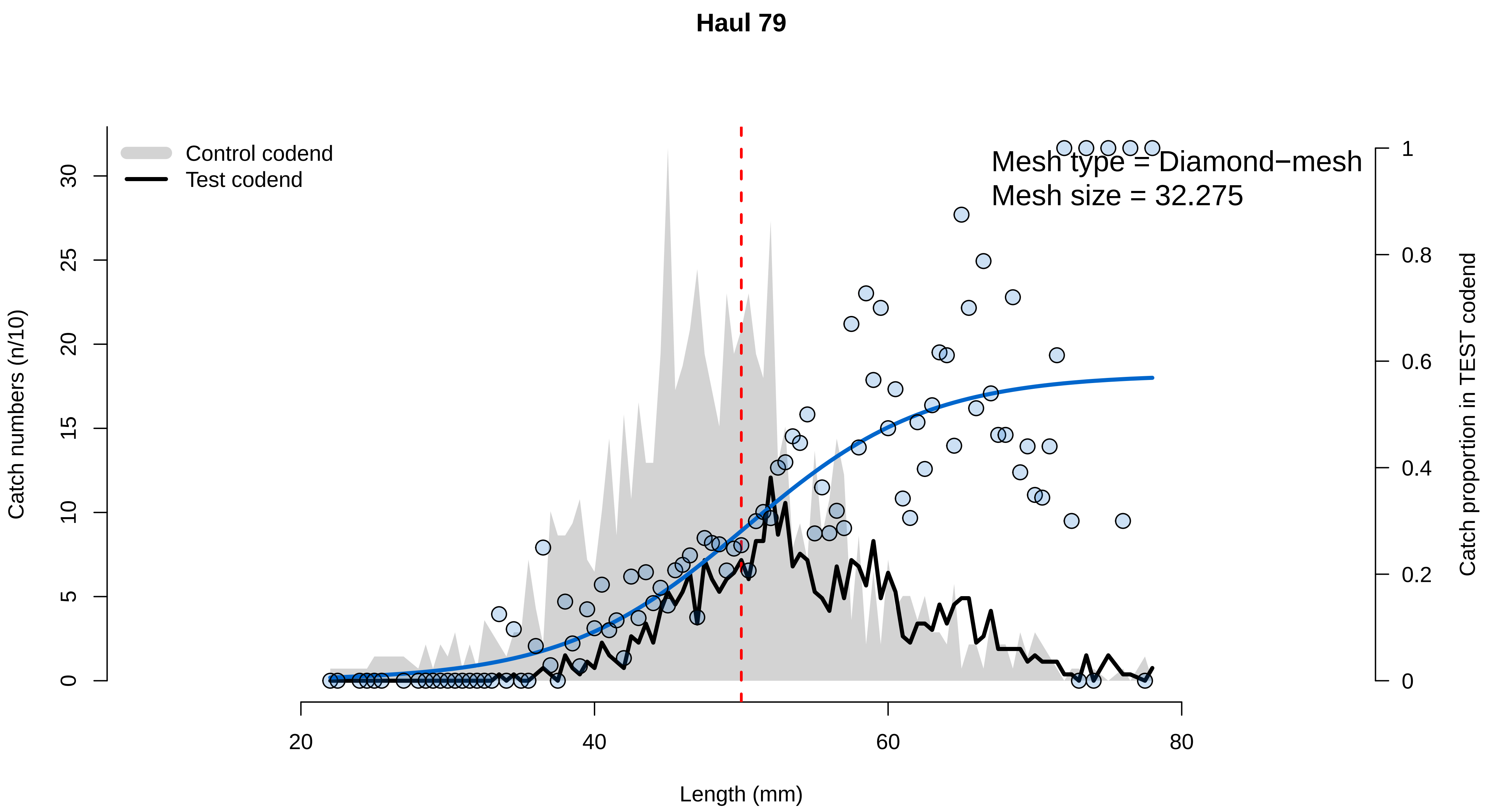

Haul 79
Control codend
Test codend
Mesh type = Diamond−mesh
Mesh size = 32.275
Catch numbers (n/10)
0
5
10
15
20
25
30
Catch proportion in TEST codend
0
0.2
0.4
0.6
0.8
1
20
40
60
80
Length (mm)

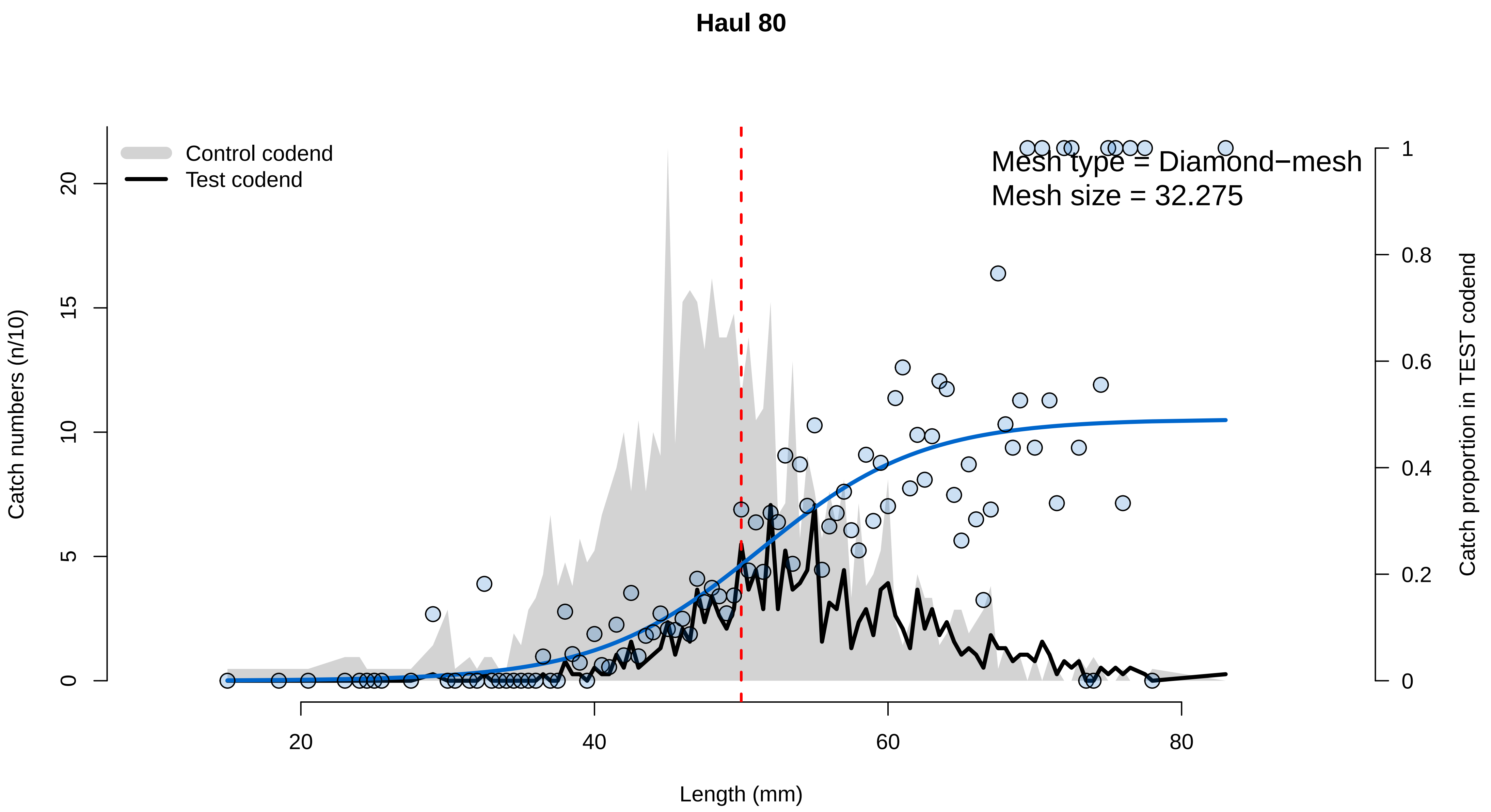
Haul 80
Control codend
Test codend
Mesh type = Diamond−mesh
Mesh size = 32.275
Catch numbers (n/10)
0
5
10
15
20
Catch proportion in TEST codend
0
0.2
0.4
0.6
0.8
1
20
40
60
80
Length (mm)

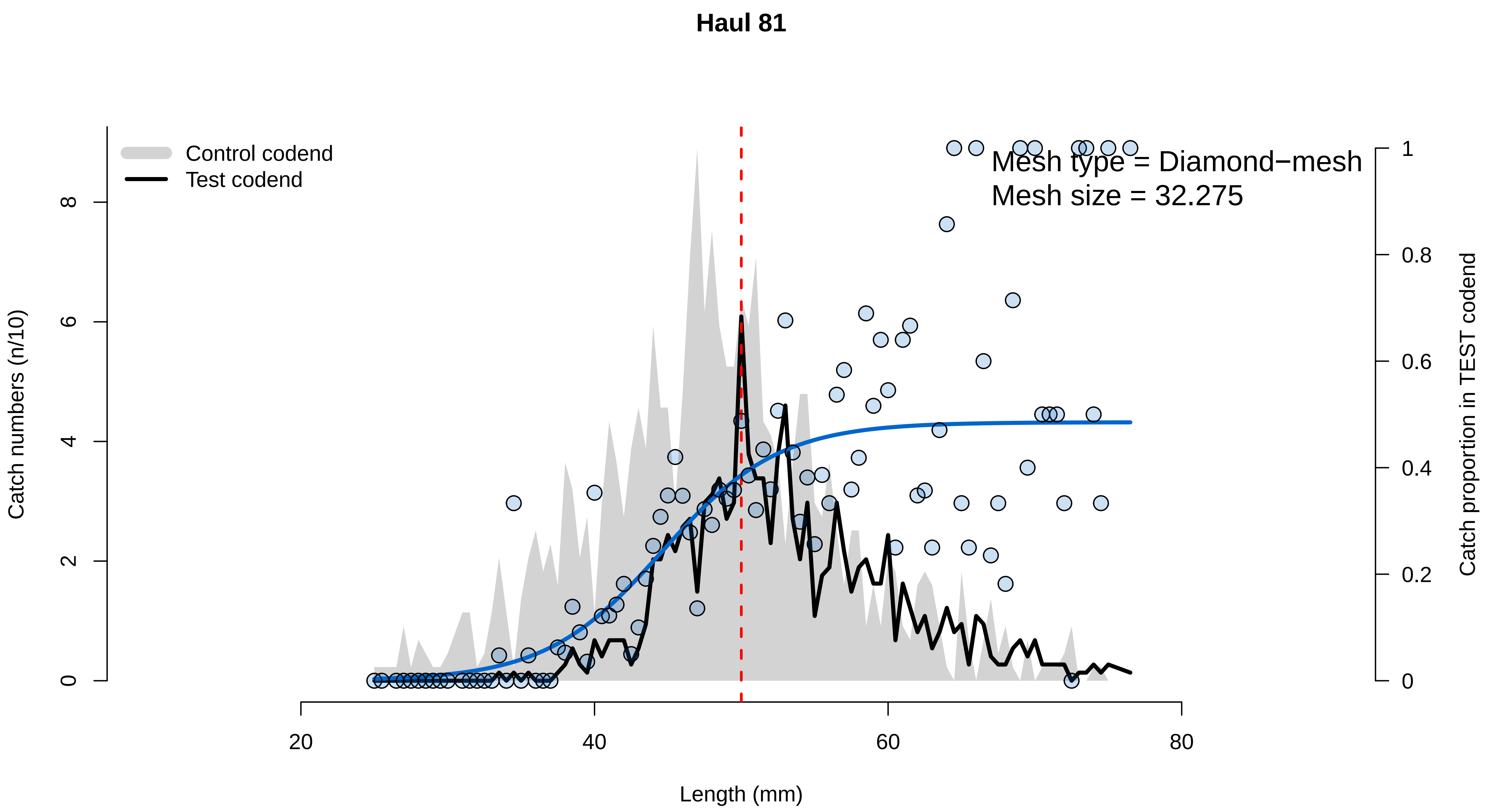

Haul 81
Control codend
Test codend
Mesh type = Diamond−mesh
Mesh size = 32.275
Catch numbers (n/10)
Catch proportion in TEST codend
Length (mm)
0
2
4
6
8
0
0.2
0.4
0.6
0.8
1
20
40
60
80

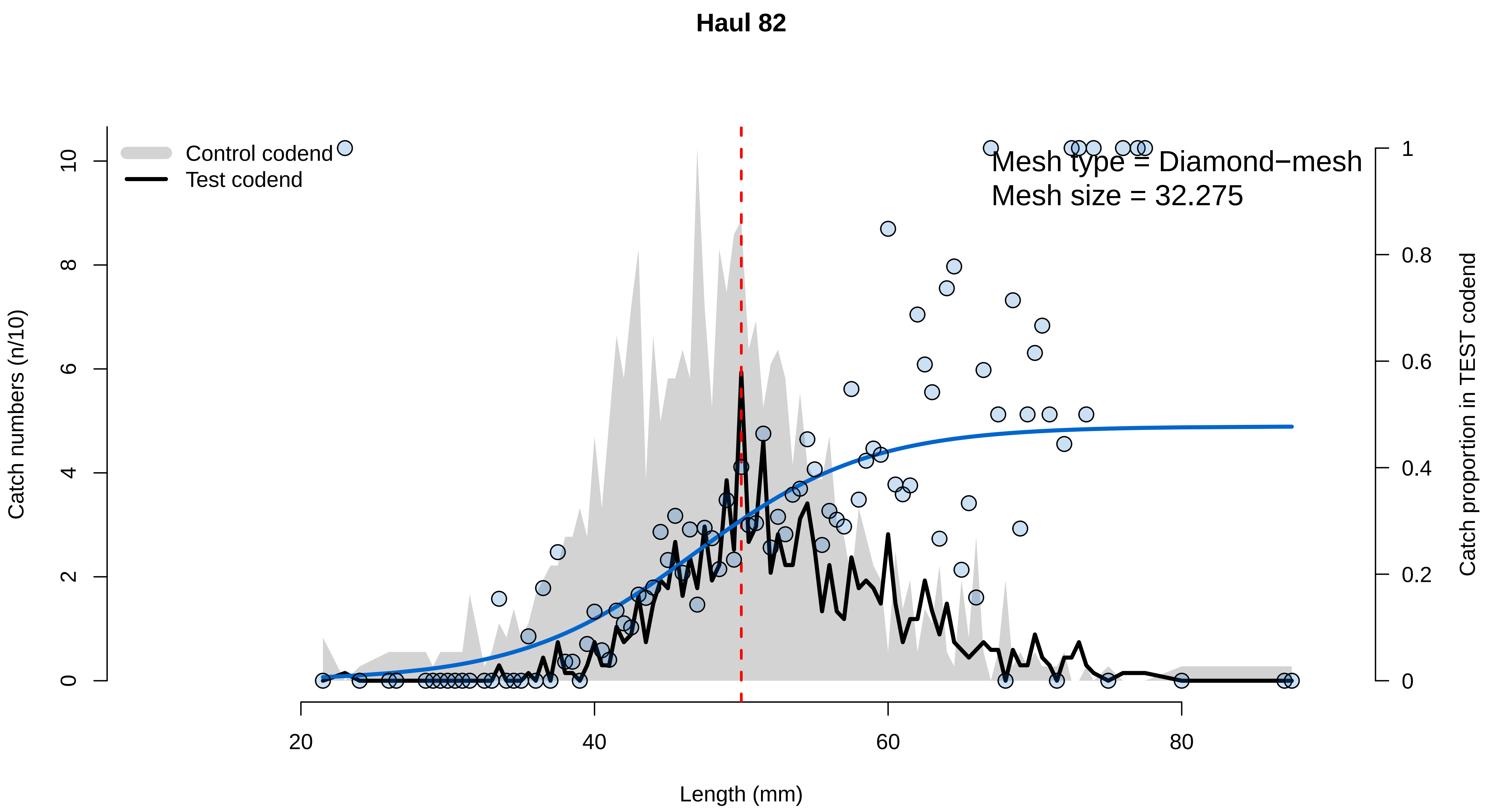
Haul 82
Control codend
Test codend
Mesh type = Diamond−mesh
Mesh size = 32.275
Catch numbers (n/10)
Catch proportion in TEST codend
Length (mm)
0
2
4
6
8
10
20
40
60
80
0
0.2
0.4
0.6
0.8
1

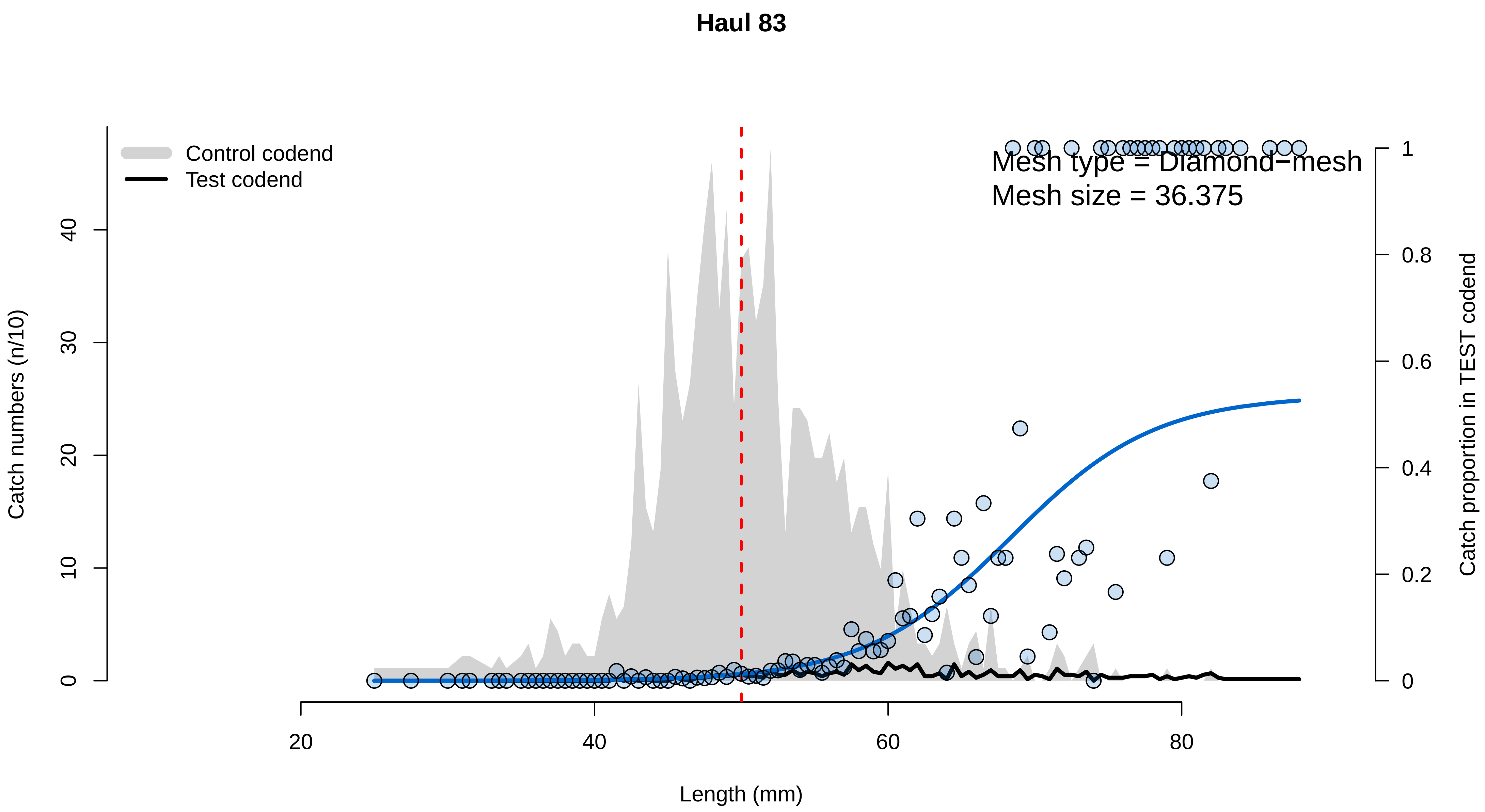

Haul 83
Control codend
Test codend
Mesh type = Diamond−mesh
Mesh size = 36.375
Catch numbers (n/10)
Catch proportion in TEST codend
Length (mm)
0
10
20
30
40
0
0.2
0.4
0.6
0.8
1
20
40
60
80

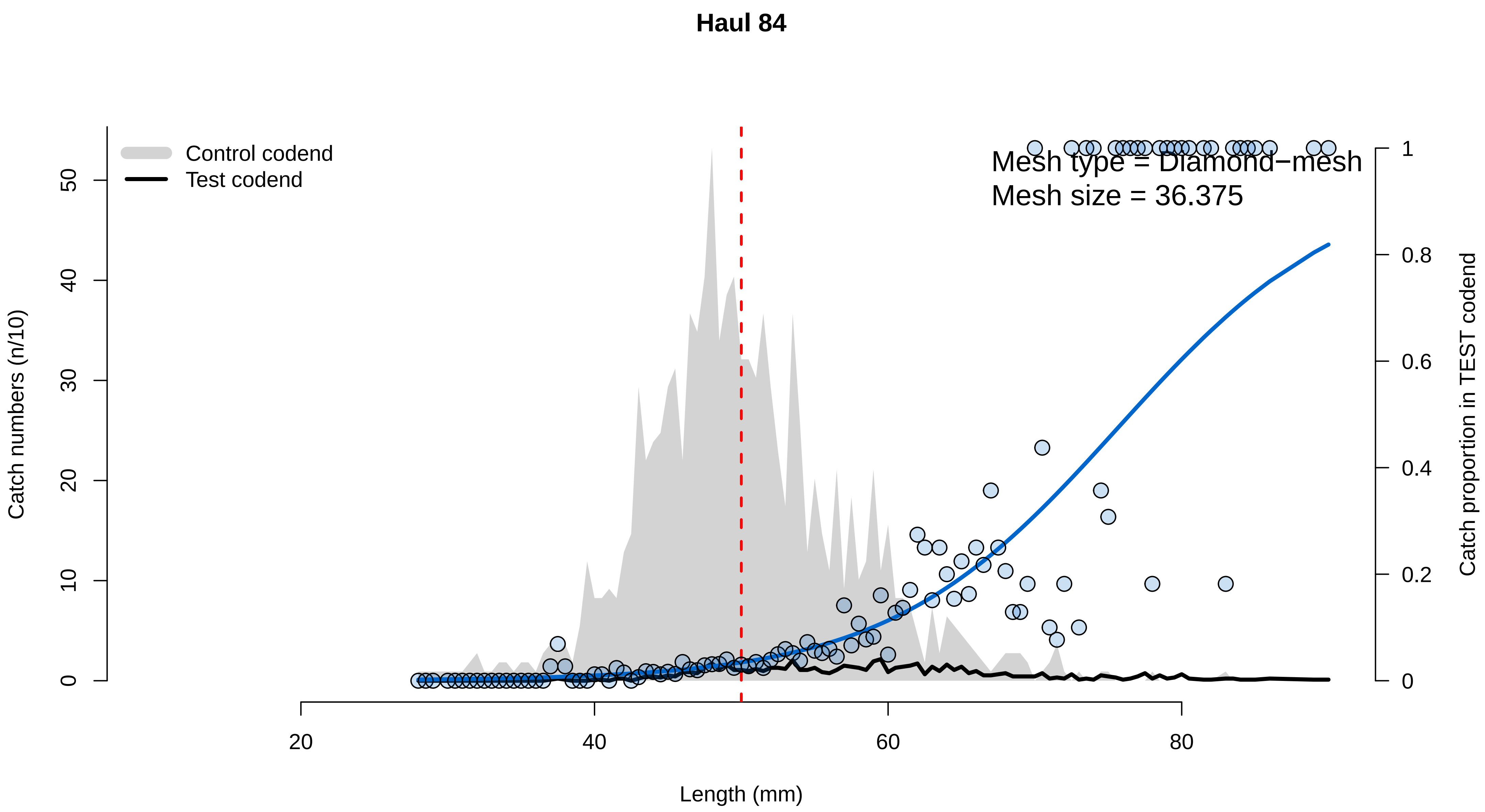
Haul 84
Control codend
Test codend
Mesh type = Diamond−mesh
Mesh size = 36.375
Catch numbers (n/10)
0
10
20
30
40
50
Catch proportion in TEST codend
0
0.2
0.4
0.6
0.8
1
20
40
60
80
Length (mm)

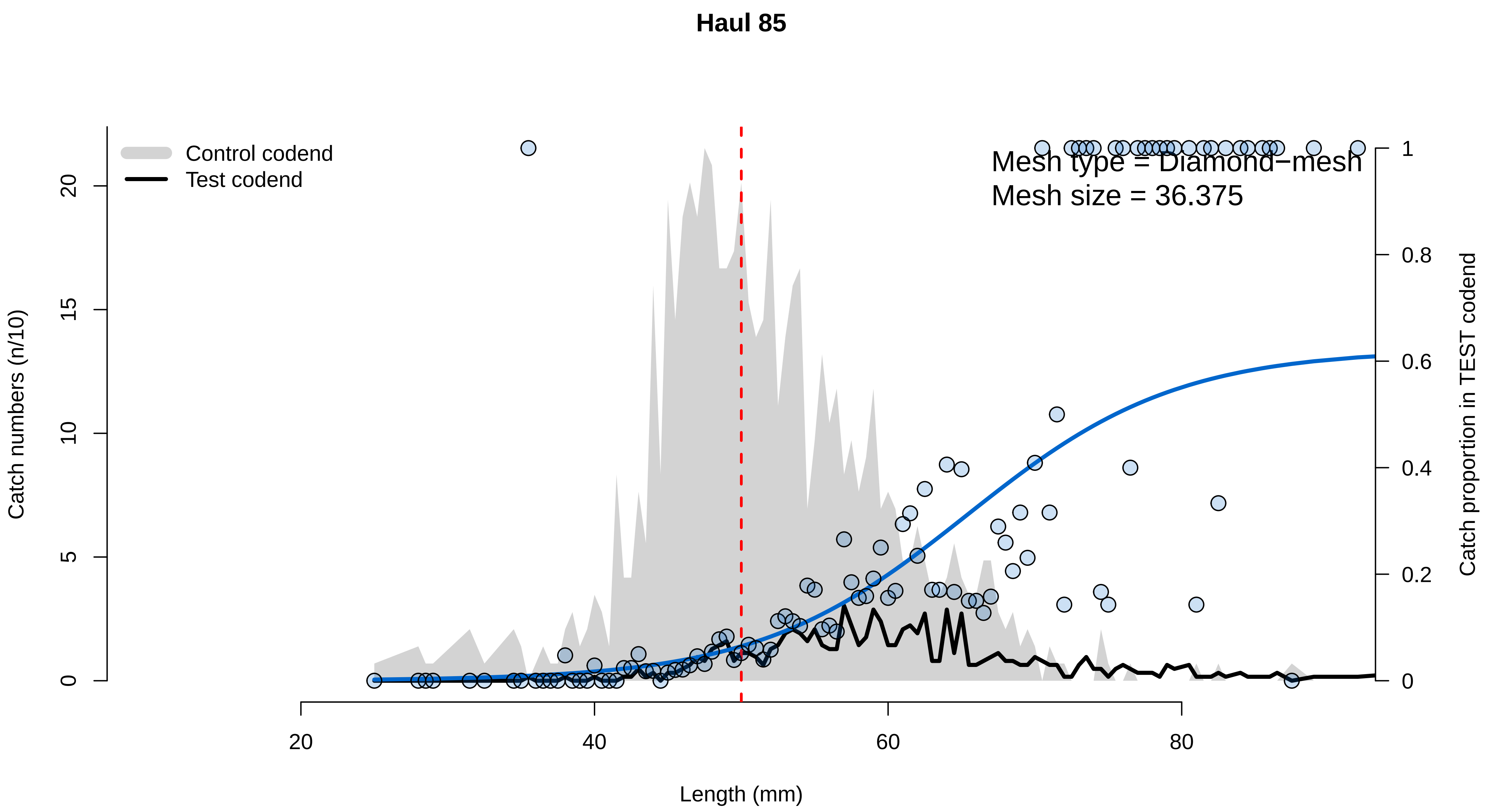

Haul 85
Control codend
Test codend
Mesh type = Diamond−mesh
Mesh size = 36.375
Catch numbers (n/10)
Catch proportion in TEST codend
Length (mm)
0
5
10
15
20
0
0.2
0.4
0.6
0.8
1
20
40
60
80

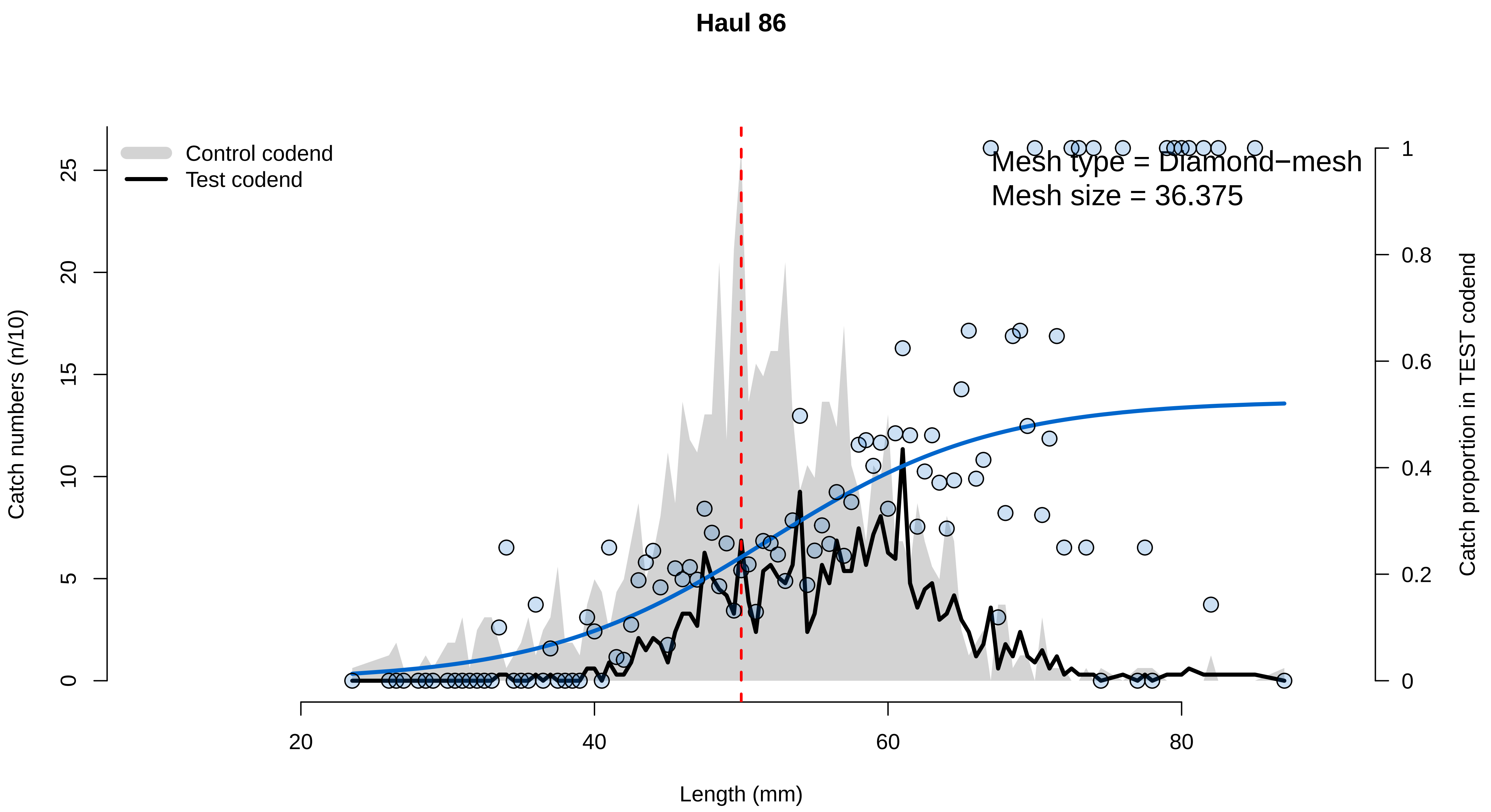

Haul 86
Control codend
Test codend
Mesh type = Diamond−mesh
Mesh size = 36.375
Catch numbers (n/10)
0
5
10
15
20
25
Catch proportion in TEST codend
0
0.2
0.4
0.6
0.8
1
Length (mm)
20
40
60
80

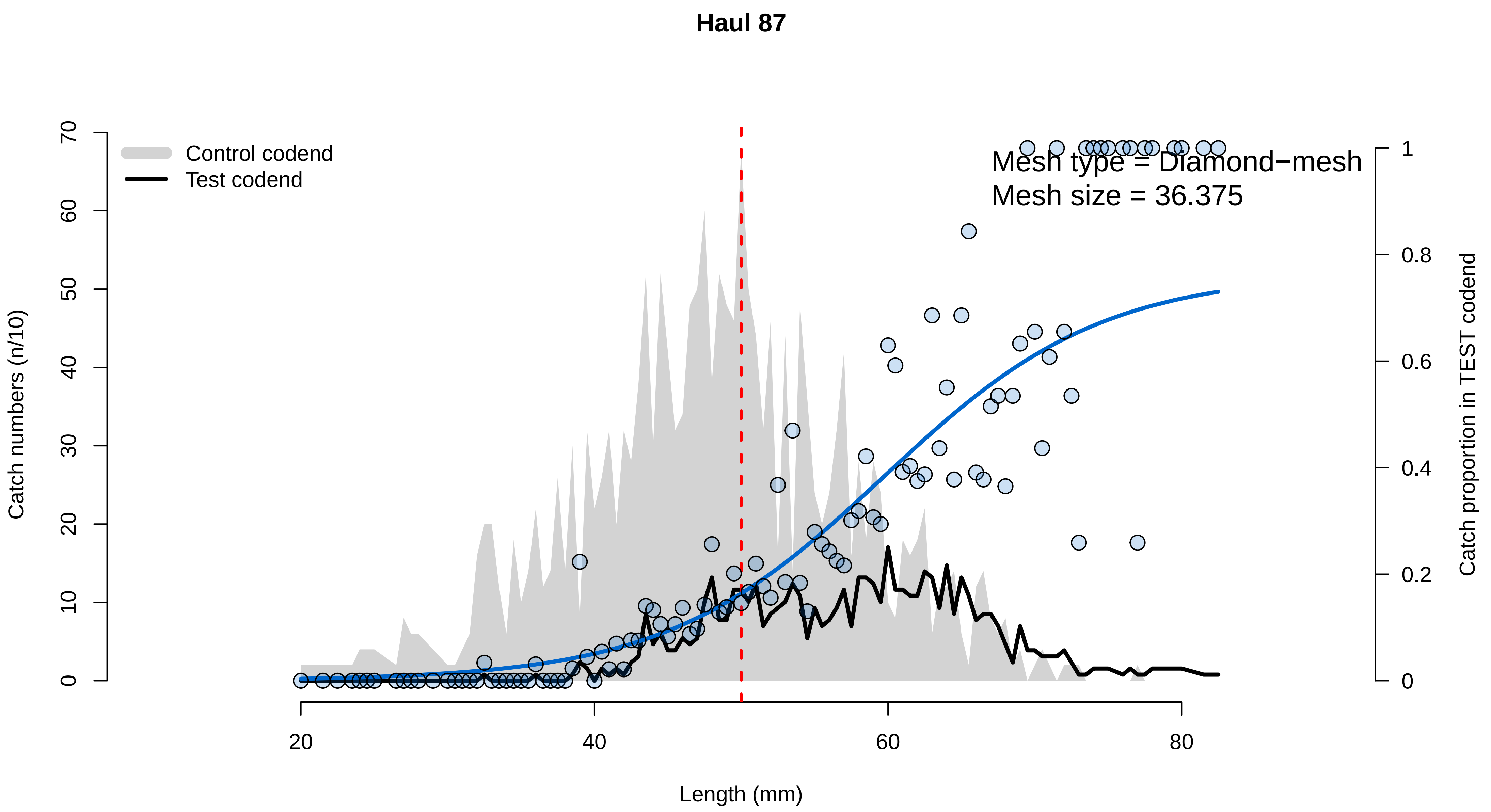

Haul 87
Control codend
Test codend
Mesh type = Diamond−mesh
Mesh size = 36.375
Catch numbers (n/10)
0
10
20
30
40
50
60
70
Catch proportion in TEST codend
0
0.2
0.4
0.6
0.8
1
20
40
60
80
Length (mm)

Haul 88

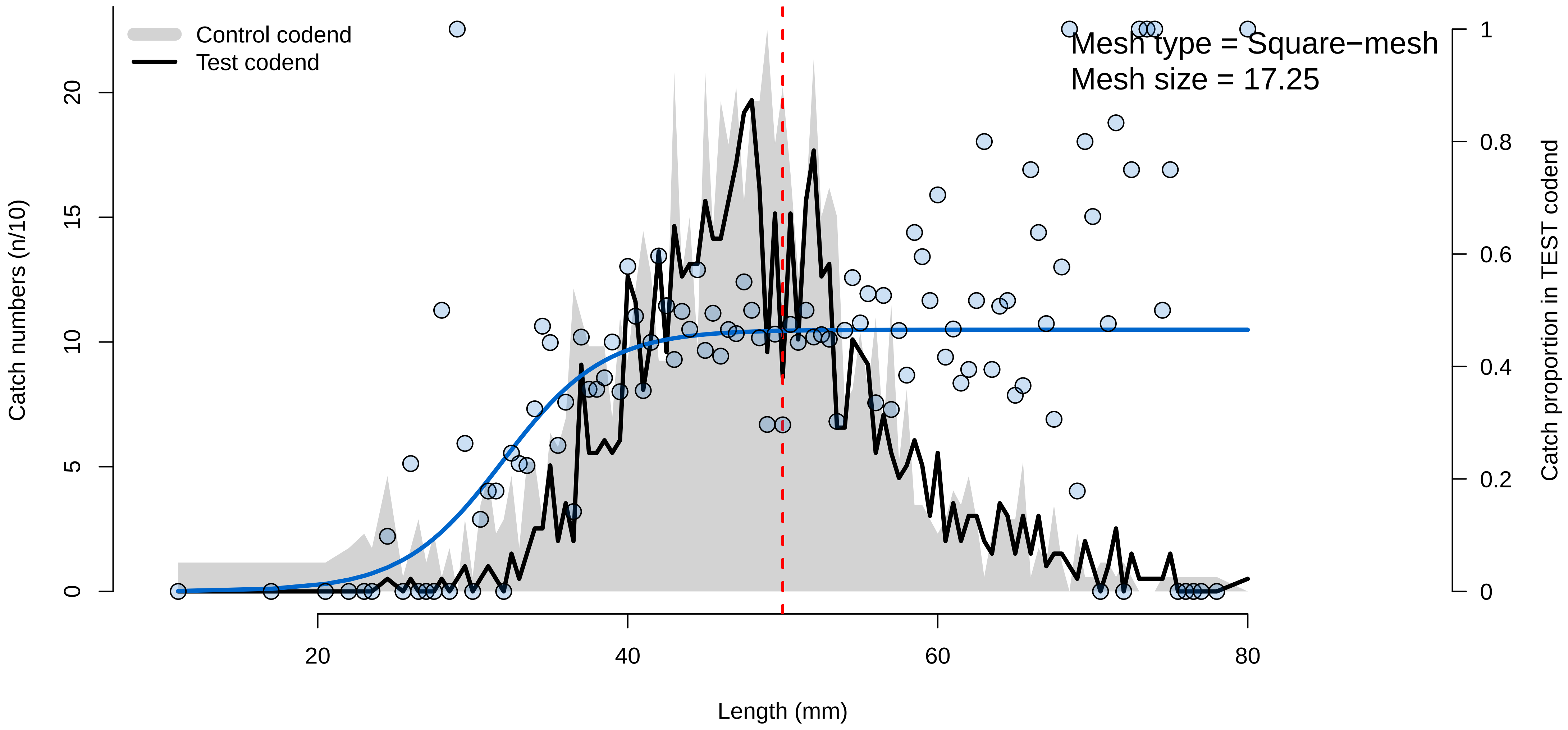

Control codend
Test codend
Mesh type = Square−mesh
Mesh size = 17.25
Catch numbers (n/10)
0
5
10
15
20
Catch proportion in TEST codend
0
0.2
0.4
0.6
0.8
1
20
40
60
80
Length (mm)

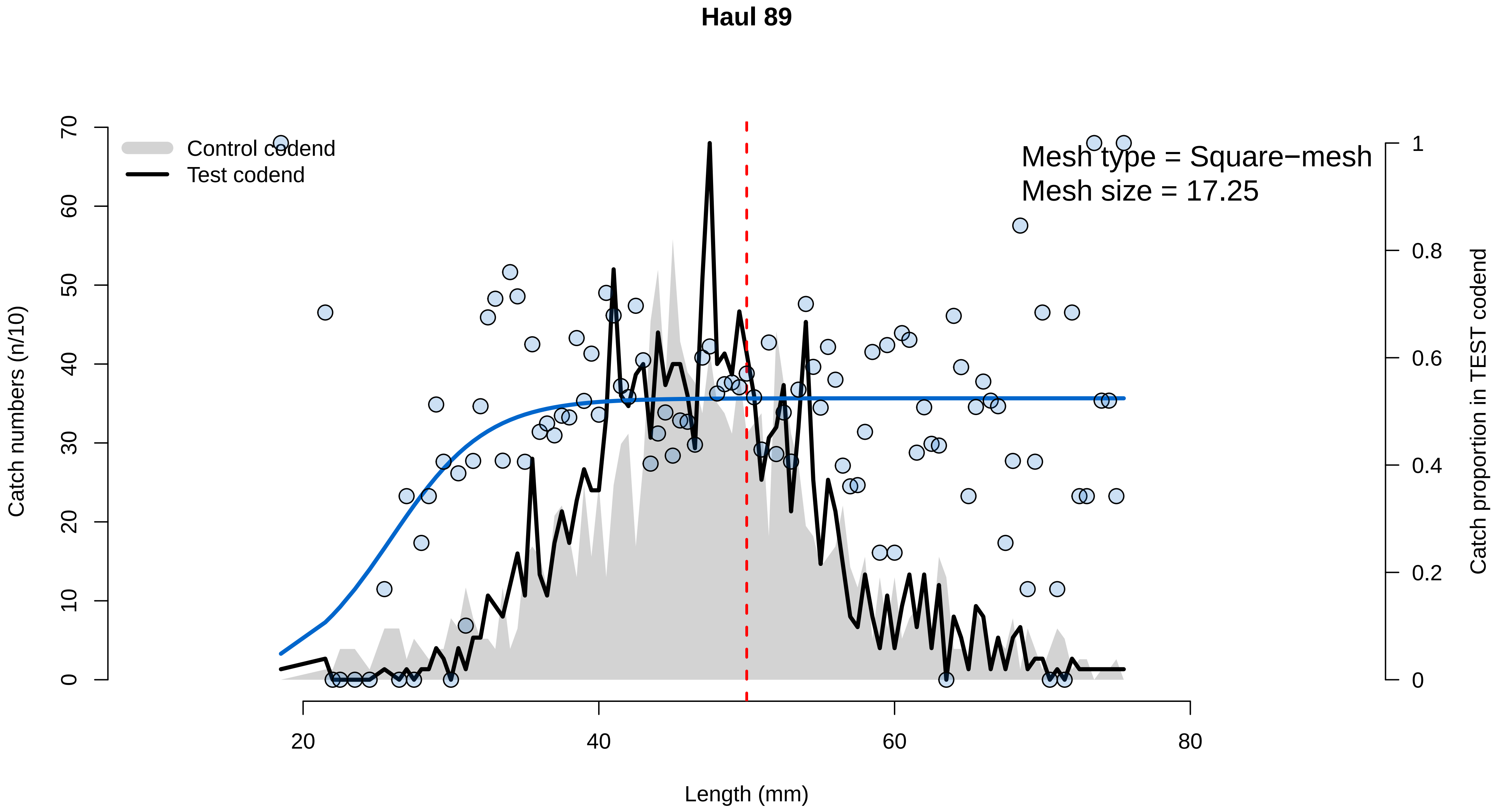
Haul 89
Control codend
Test codend
Mesh type = Square−mesh
Mesh size = 17.25
Catch numbers (n/10)
Catch proportion in TEST codend
Length (mm)
0
10
20
30
40
50
60
70
20
40
60
80
0
0.2
0.4
0.6
0.8
1

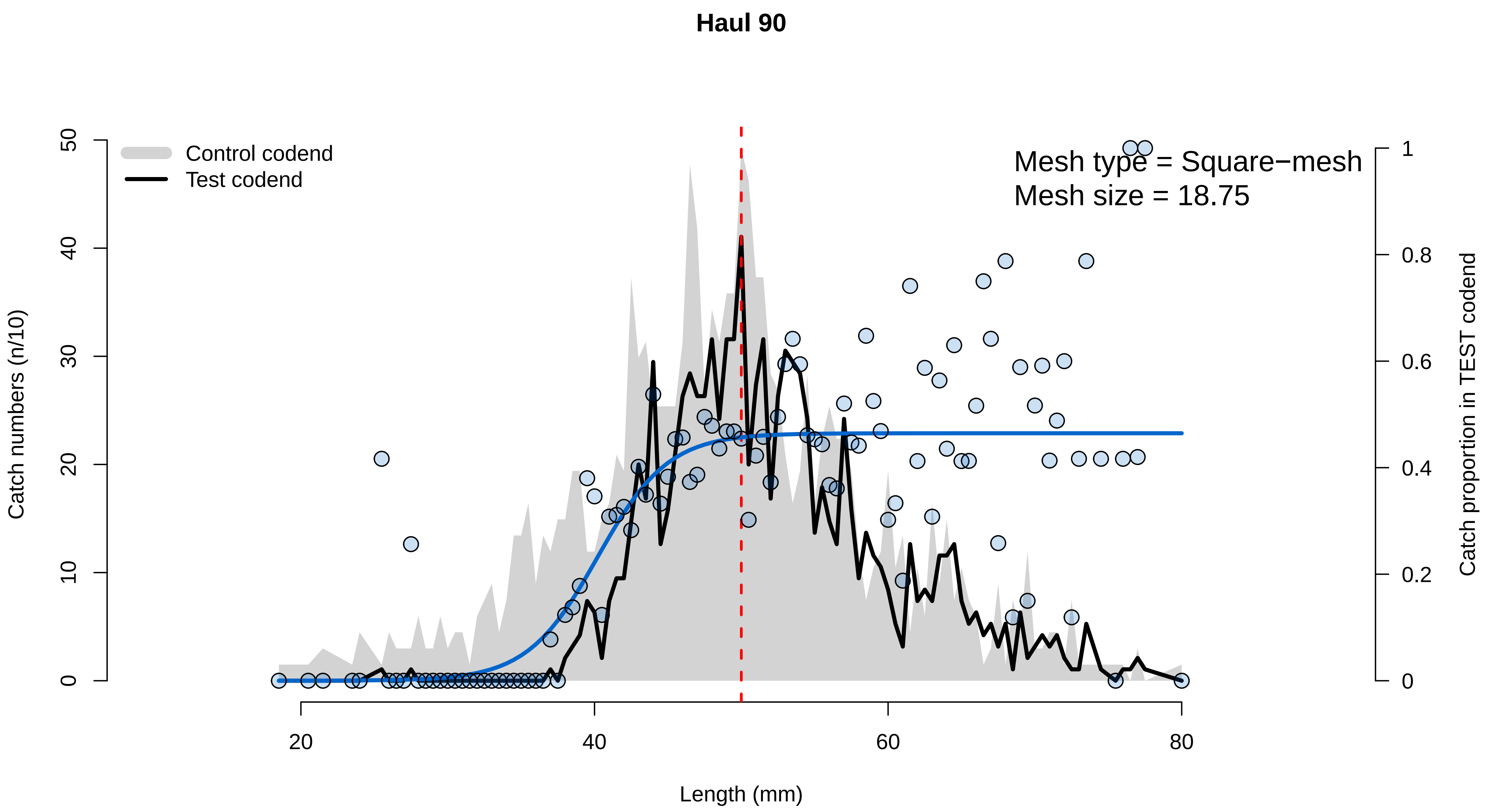
Haul 90
Control codend
Test codend
Mesh type = Square−mesh
Mesh size = 18.75
Catch numbers (n/10)
0
10
20
30
40
50
Catch proportion in TEST codend
0
0.2
0.4
0.6
0.8
1
Length (mm)
20
40
60
80

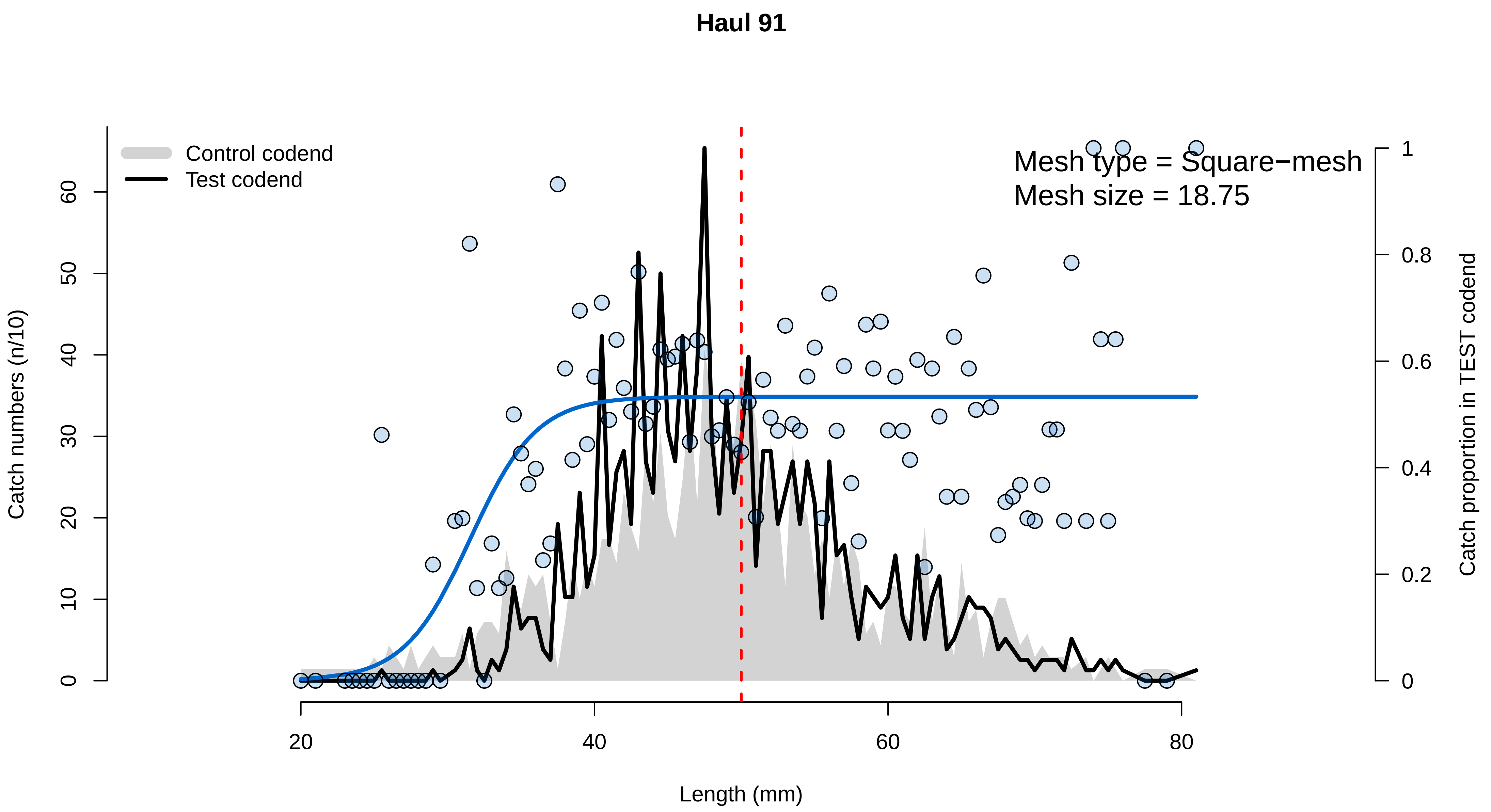
Haul 91
Control codend
Test codend
Mesh type = Square−mesh
Mesh size = 18.75
Catch numbers (n/10)
0
10
20
30
40
50
60
Catch proportion in TEST codend
0
0.2
0.4
0.6
0.8
1
20
40
60
80
Length (mm)

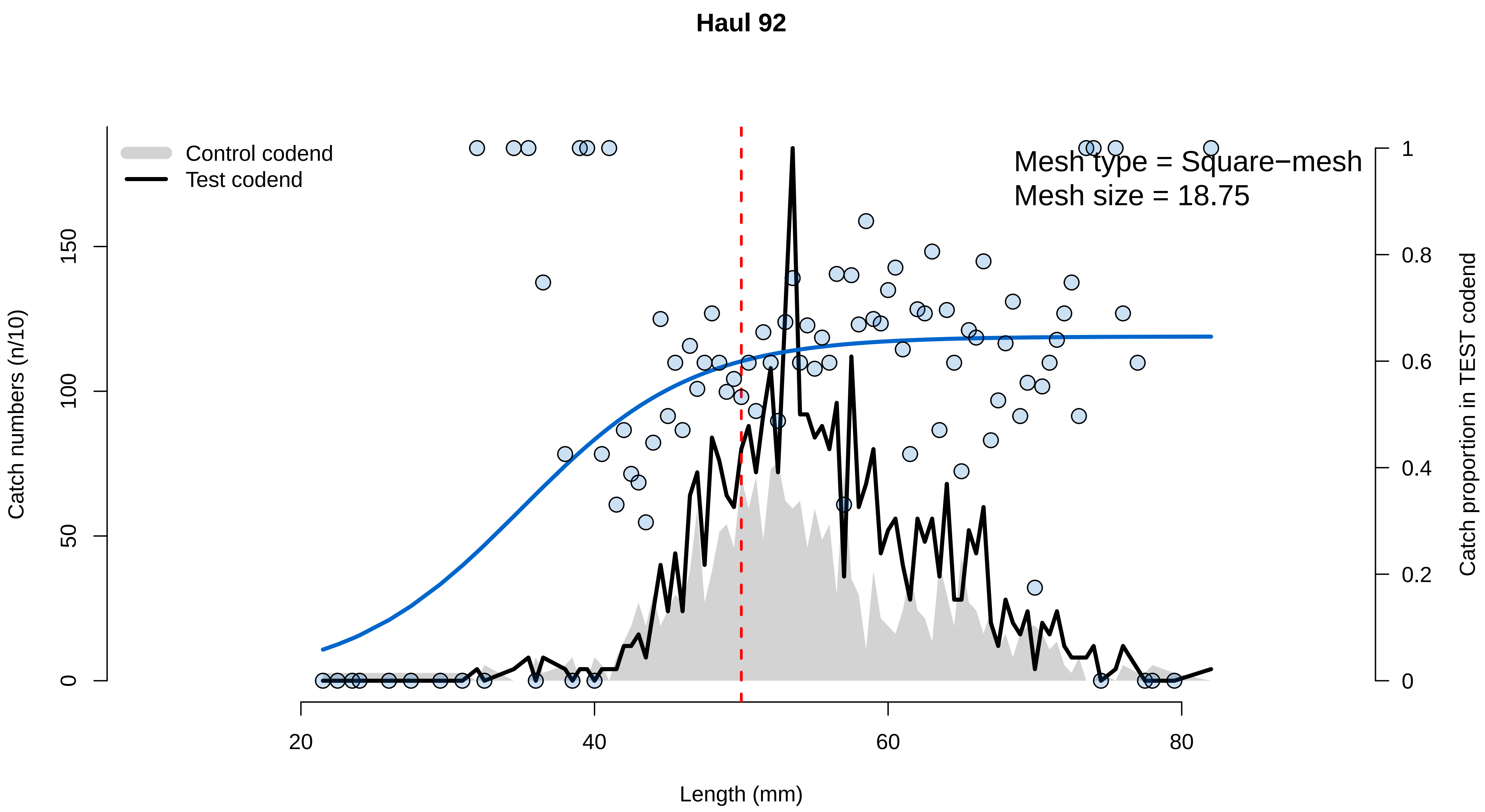
Haul 92
Control codend
Test codend
Mesh type = Square−mesh
Mesh size = 18.75
Catch numbers (n/10)
0
50
100
150
Catch proportion in TEST codend
0
0.2
0.4
0.6
0.8
1
Length (mm)
20
40
60
80

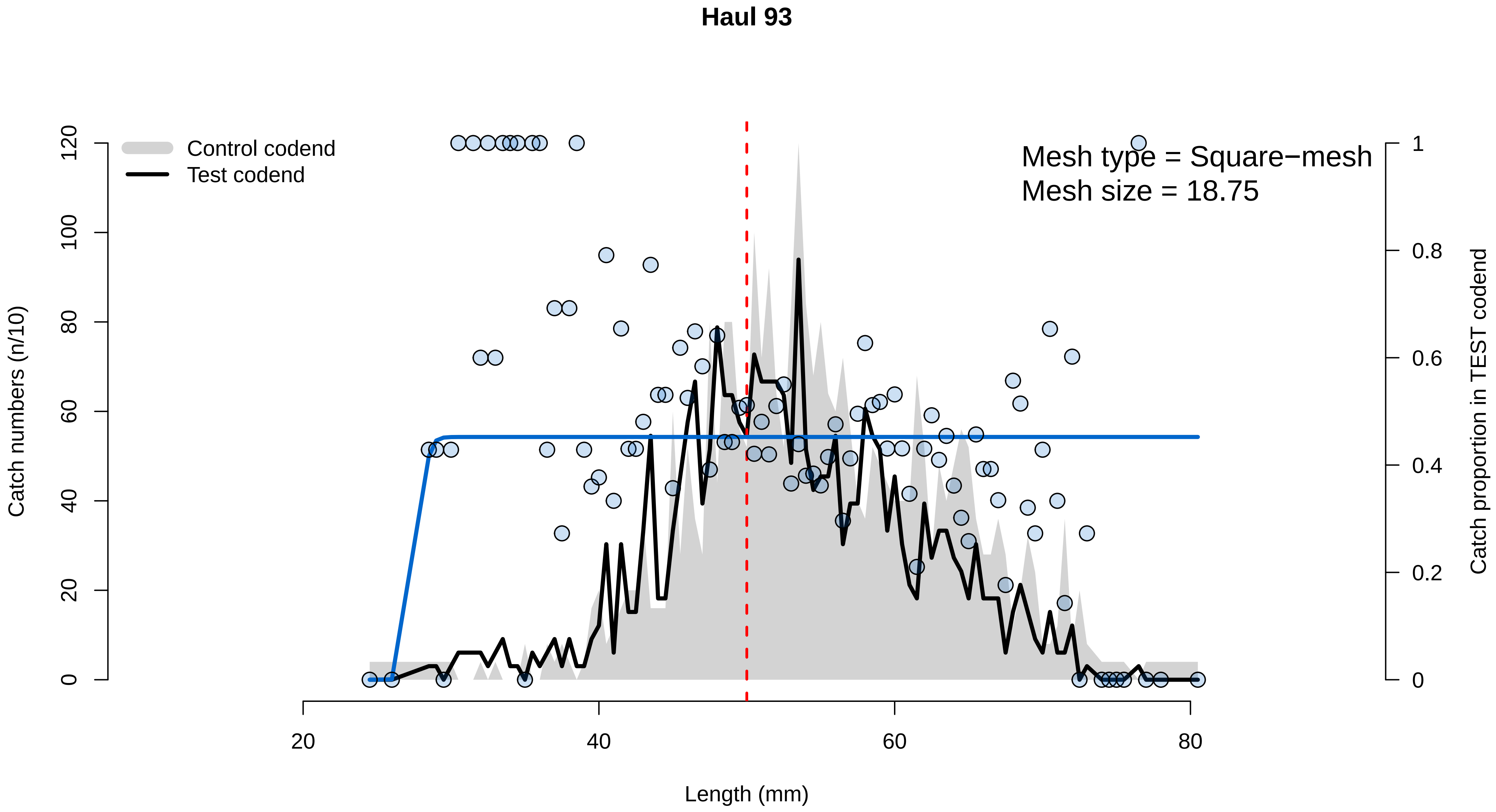

Haul 93
Control codend
Test codend
Mesh type = Square−mesh
Mesh size = 18.75
Catch numbers (n/10)
0
20
40
60
80
100
120
Catch proportion in TEST codend
0
0.2
0.4
0.6
0.8
1
20
40
60
80
Length (mm)

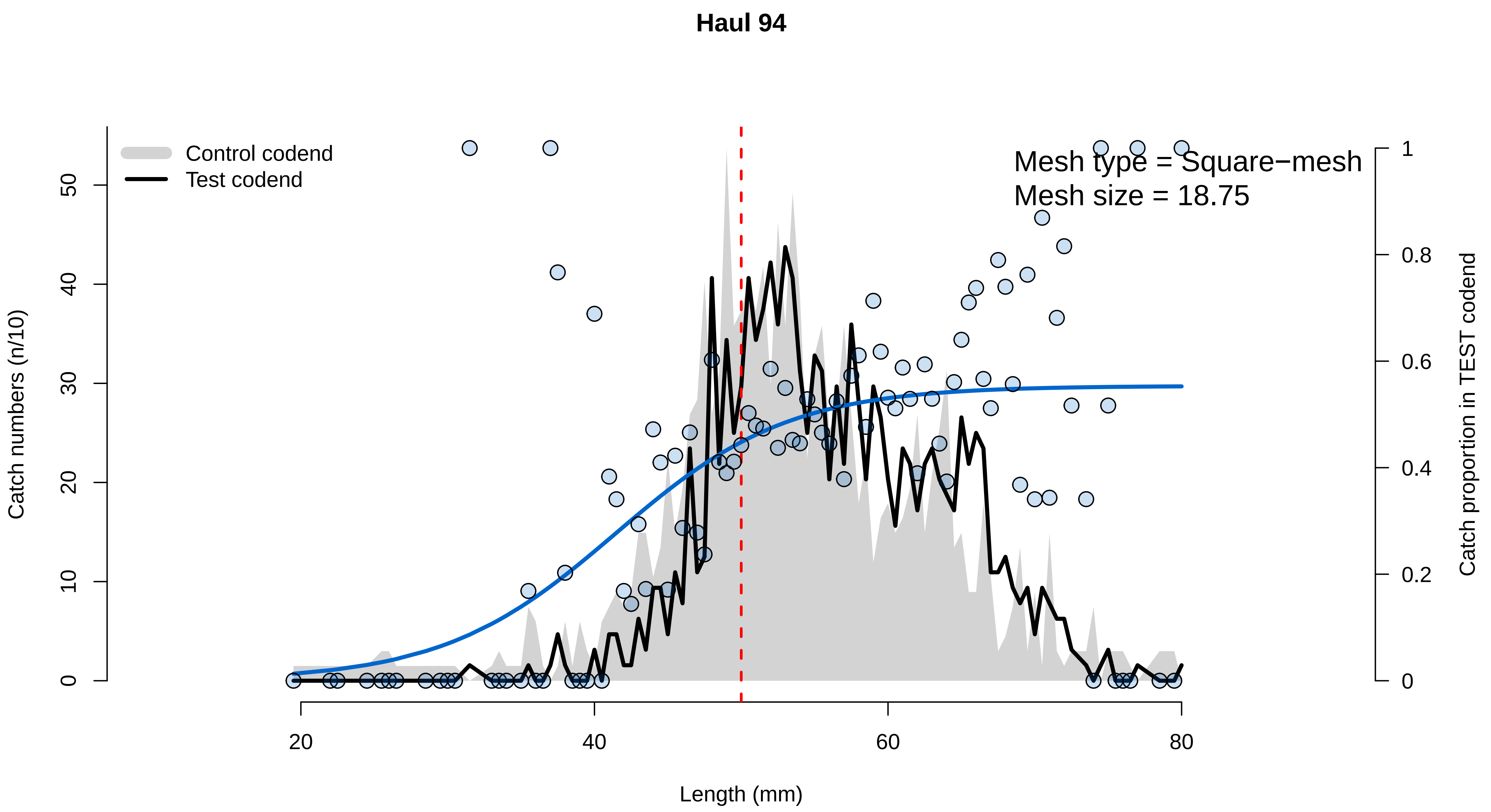
Haul 94
Control codend
Test codend
Mesh type = Square−mesh
Mesh size = 18.75
Catch numbers (n/10)
Catch proportion in TEST codend
Length (mm)
0
10
20
30
40
50
0
0.2
0.4
0.6
0.8
1
20
40
60
80

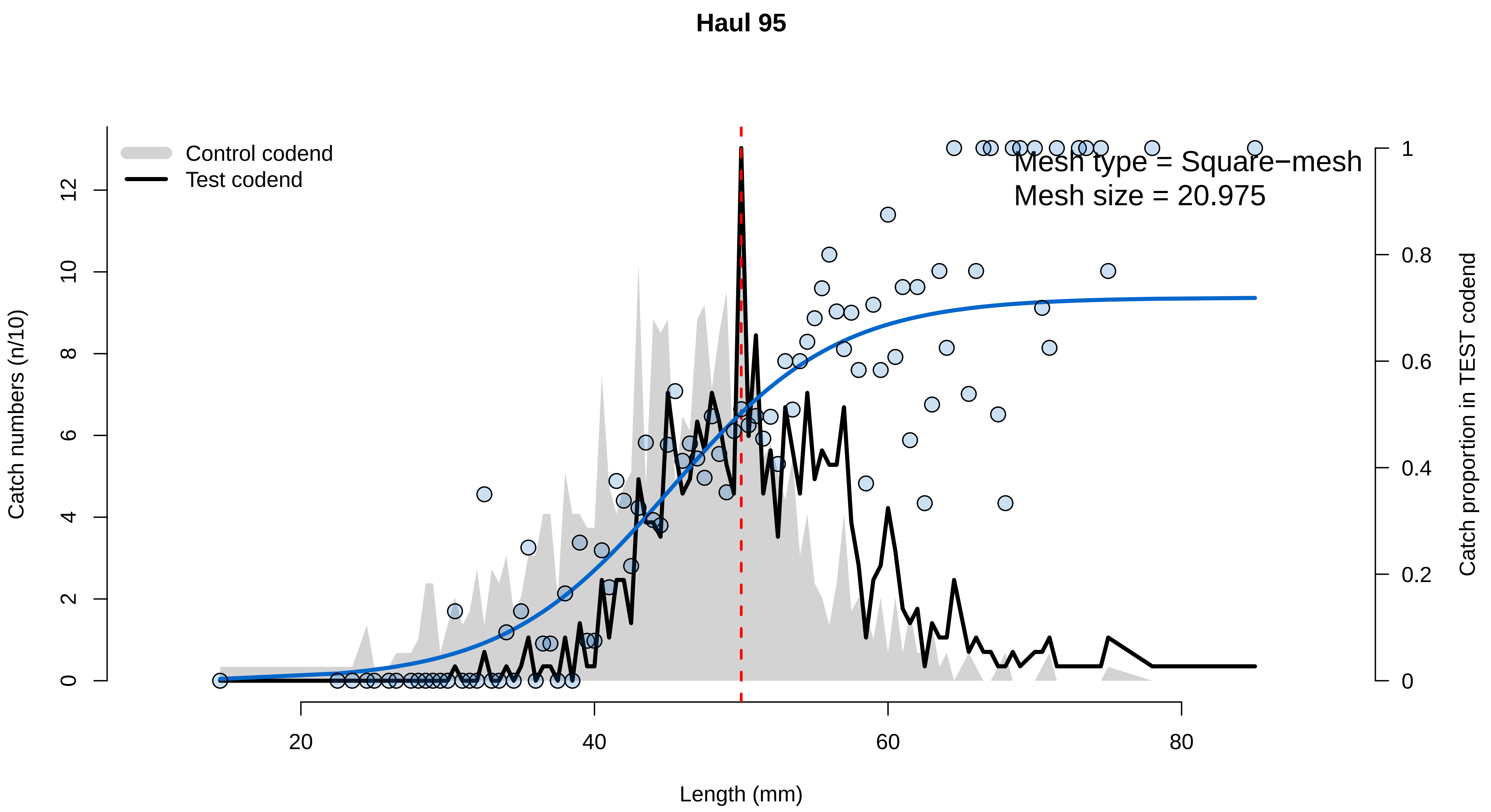

Haul 95
Control codend
Test codend
Mesh type = Square−mesh
Mesh size = 20.975
Catch numbers (n/10)
Catch proportion in TEST codend
Length (mm)
0
2
4
6
8
10
12
20
40
60
80
0
0.2
0.4
0.6
0.8
1

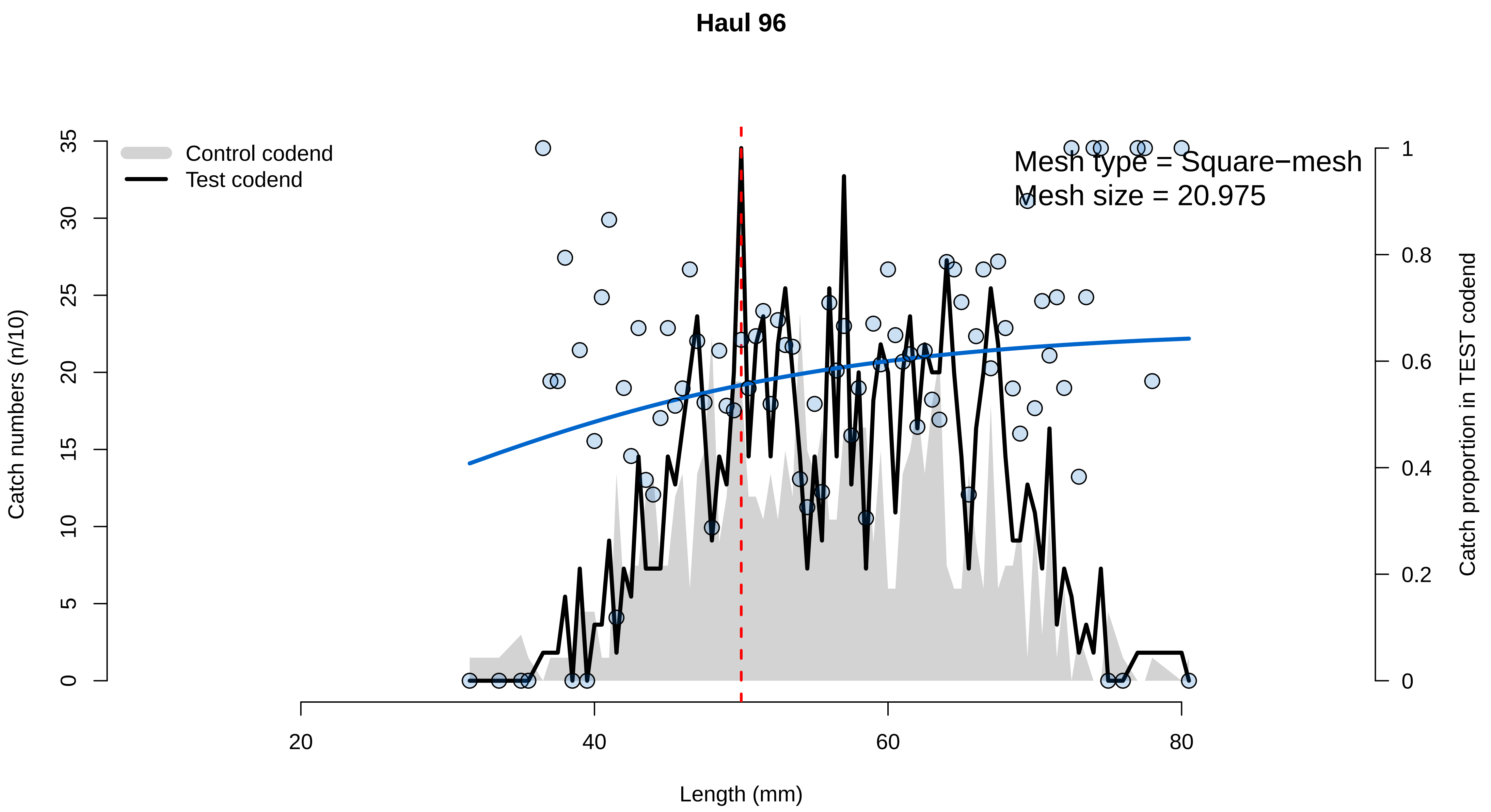
Haul 96
Control codend
Test codend
Mesh type = Square−mesh
Mesh size = 20.975
Catch numbers (n/10)
Catch proportion in TEST codend
Length (mm)
0
5
10
15
20
25
30
35
0
0.2
0.4
0.6
0.8
1
20
40
60
80

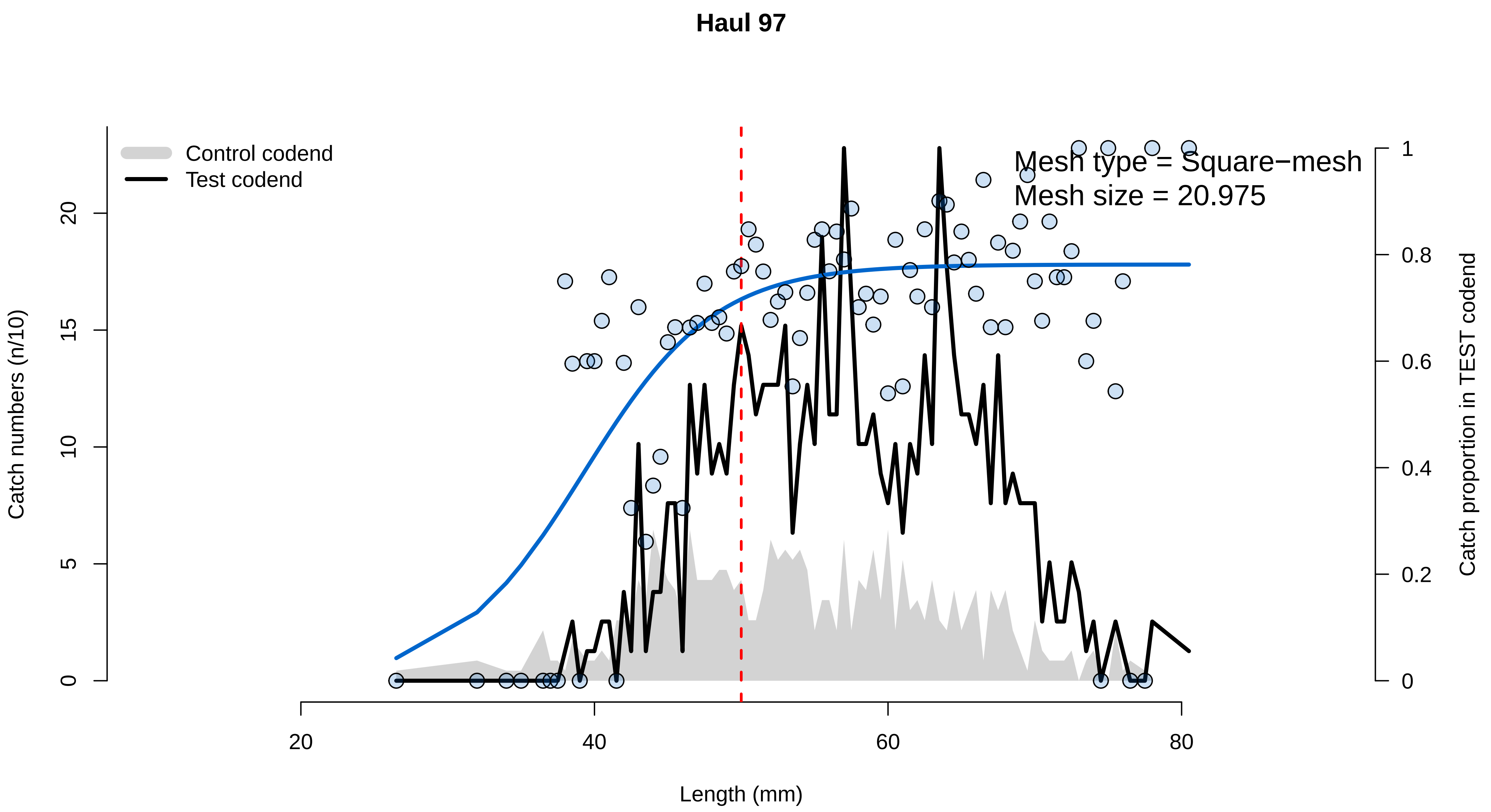

Haul 97
Control codend
Test codend
Mesh type = Square−mesh
Mesh size = 20.975
Catch numbers (n/10)
0
5
10
15
20
Catch proportion in TEST codend
0
0.2
0.4
0.6
0.8
1
20
40
60
80
Length (mm)

Haul 98

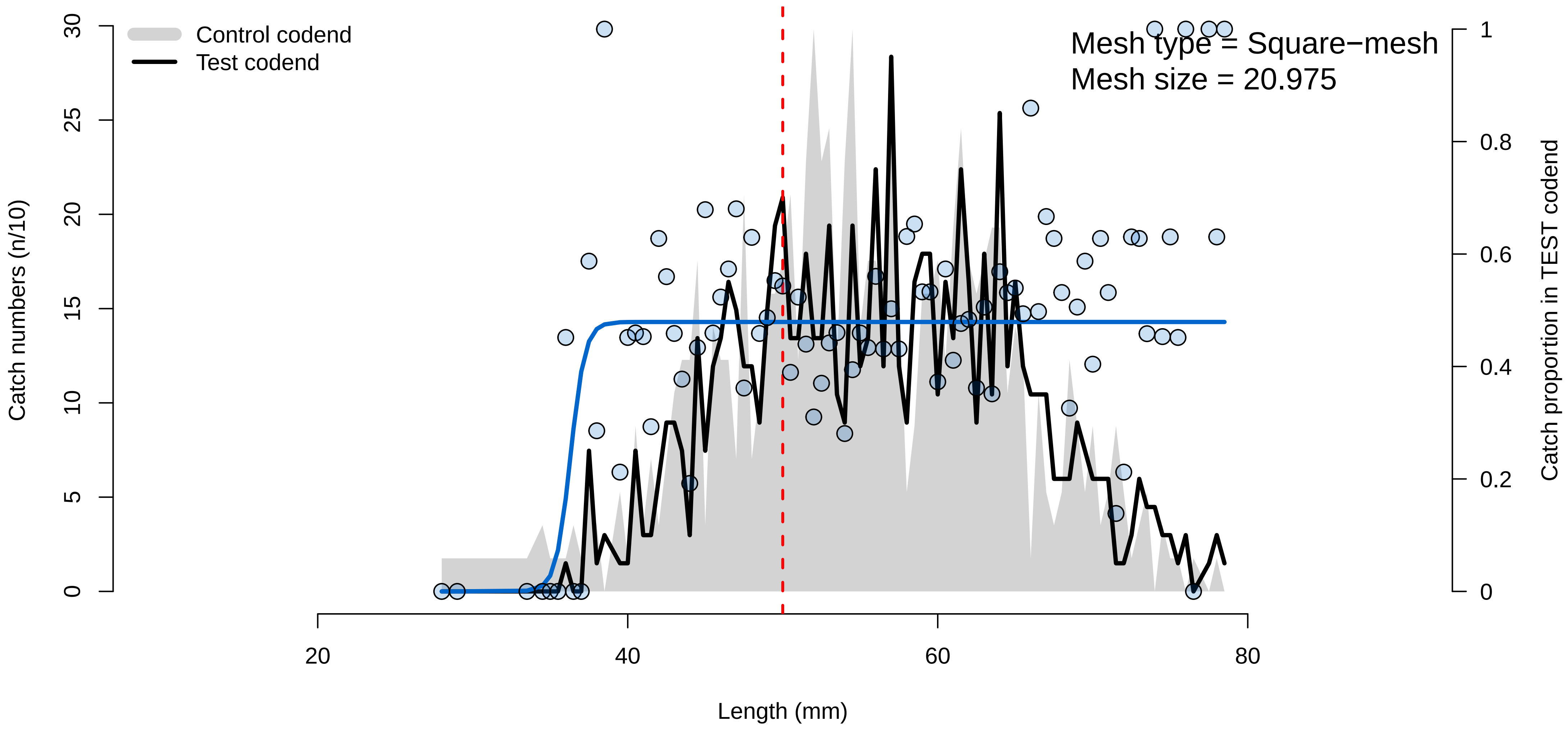

Control codend
Test codend
Mesh type = Square−mesh
Mesh size = 20.975
Catch numbers (n/10)
Catch proportion in TEST codend
Length (mm)
0
5
10
15
20
25
30
0
0.2
0.4
0.6
0.8
1
20
40
60
80

Haul 99

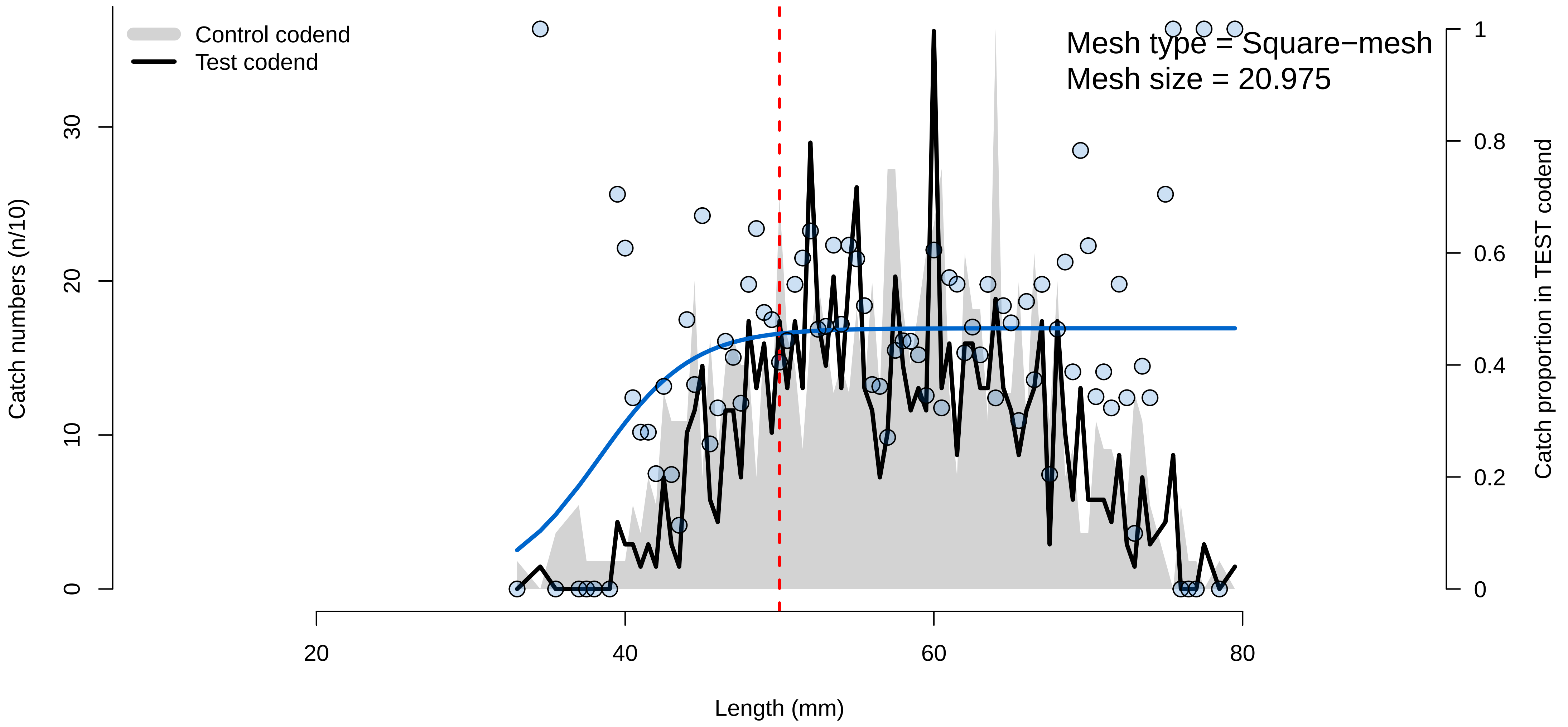

Control codend
Test codend
Mesh type = Square−mesh
Mesh size = 20.975
Catch numbers (n/10)
0
10
20
30
Catch proportion in TEST codend
0
0.2
0.4
0.6
0.8
1
20
40
60
80
Length (mm)

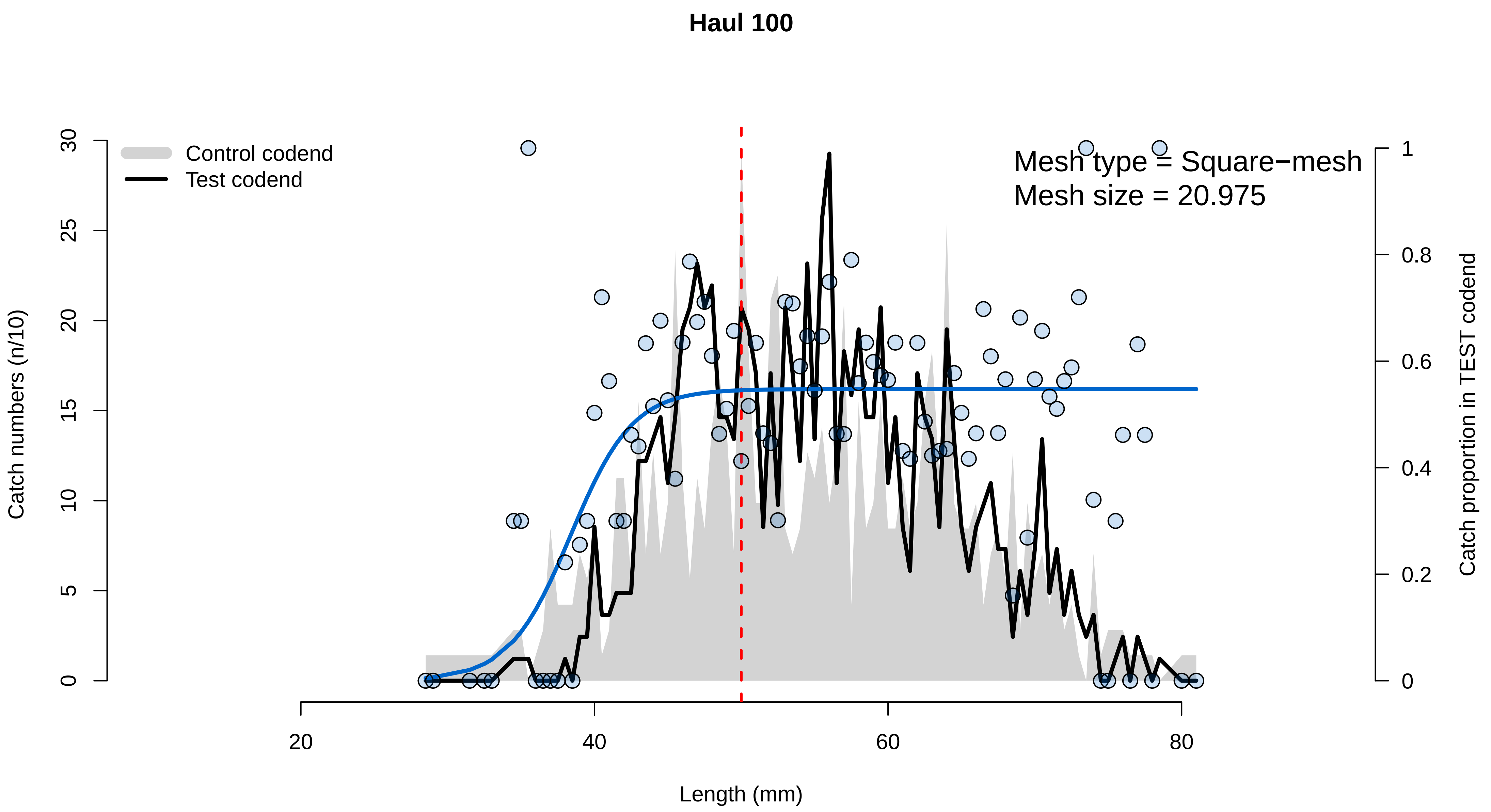

Haul 100
Control codend
Test codend
Mesh type = Square−mesh
Mesh size = 20.975
Catch numbers (n/10)
Catch proportion in TEST codend
Length (mm)
0
5
10
15
20
25
30
0
0.2
0.4
0.6
0.8
1
20
40
60
80

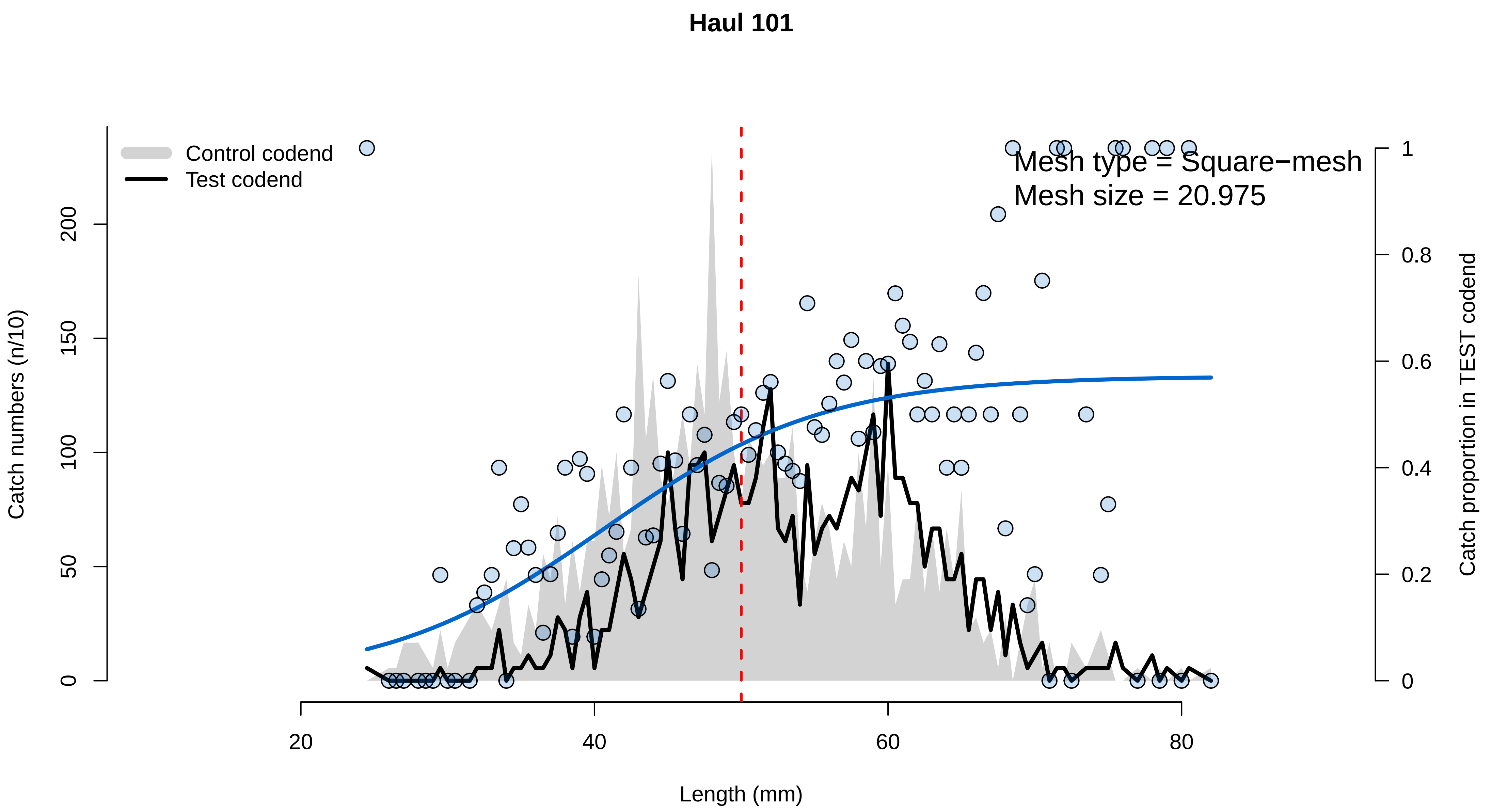
Haul 101
Control codend
Test codend
Mesh type = Square−mesh
Mesh size = 20.975
Catch numbers (n/10)
0
50
100
150
200
Catch proportion in TEST codend
0
0.2
0.4
0.6
0.8
1
20
40
60
80
Length (mm)

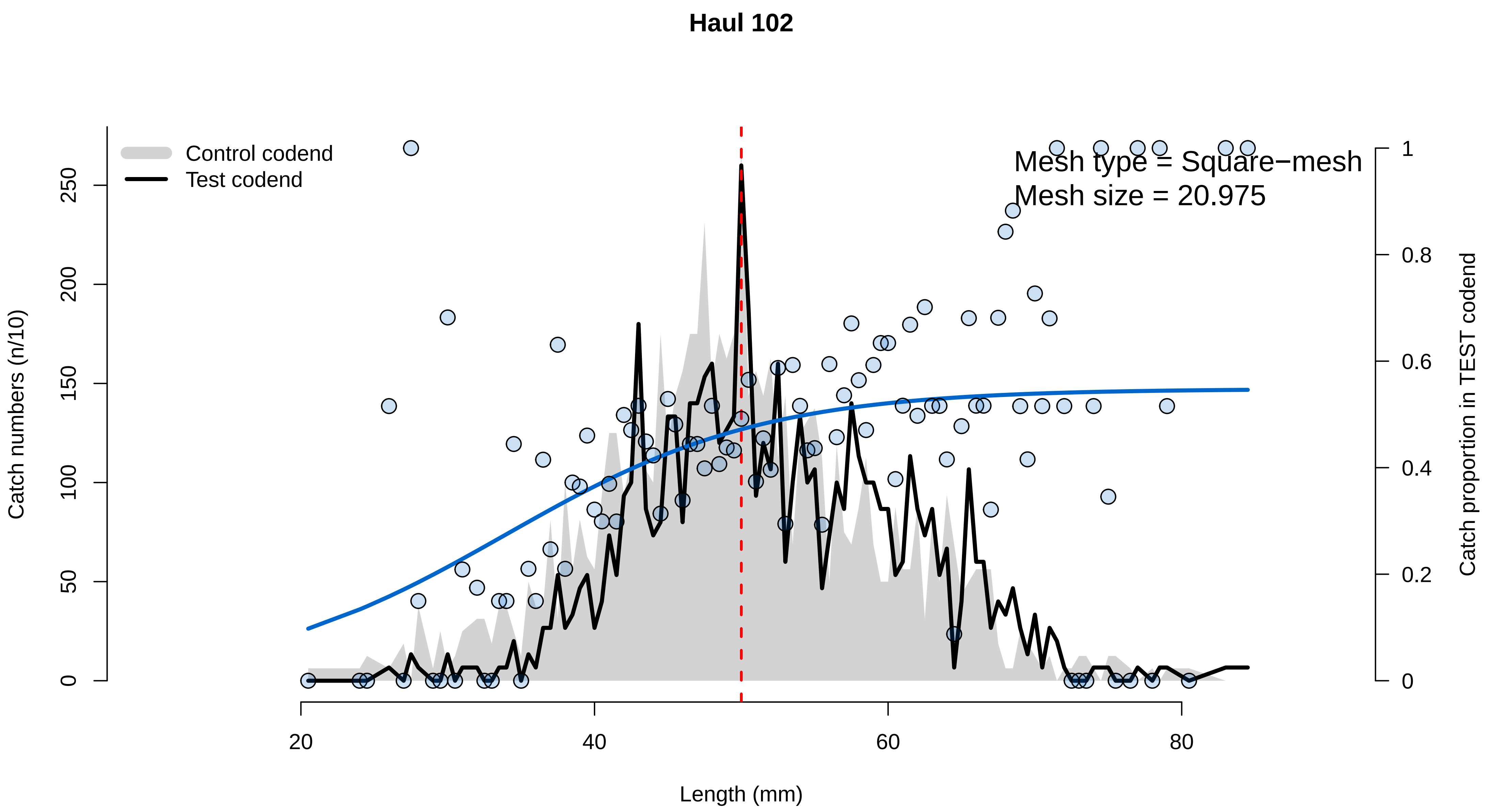
Haul 102
Control codend
Test codend
Mesh type = Square−mesh
Mesh size = 20.975
Catch numbers (n/10)
0
50
100
150
200
250
Catch proportion in TEST codend
0
0.2
0.4
0.6
0.8
1
20
40
60
80
Length (mm)

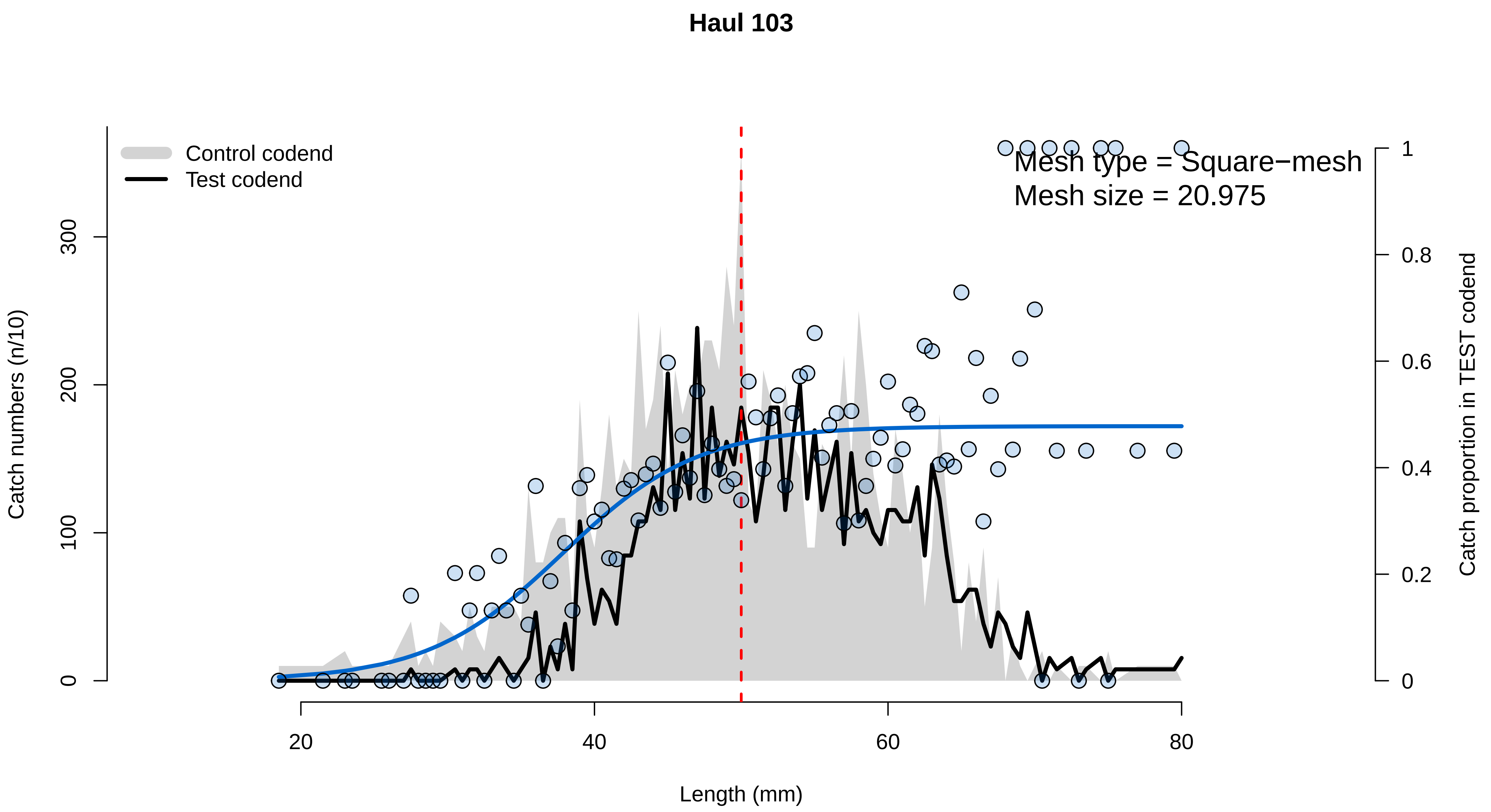

Haul 103
Control codend
Test codend
Mesh type = Square−mesh
Mesh size = 20.975
Catch numbers (n/10)
0
100
200
300
Catch proportion in TEST codend
0
0.2
0.4
0.6
0.8
1
20
40
60
80
Length (mm)

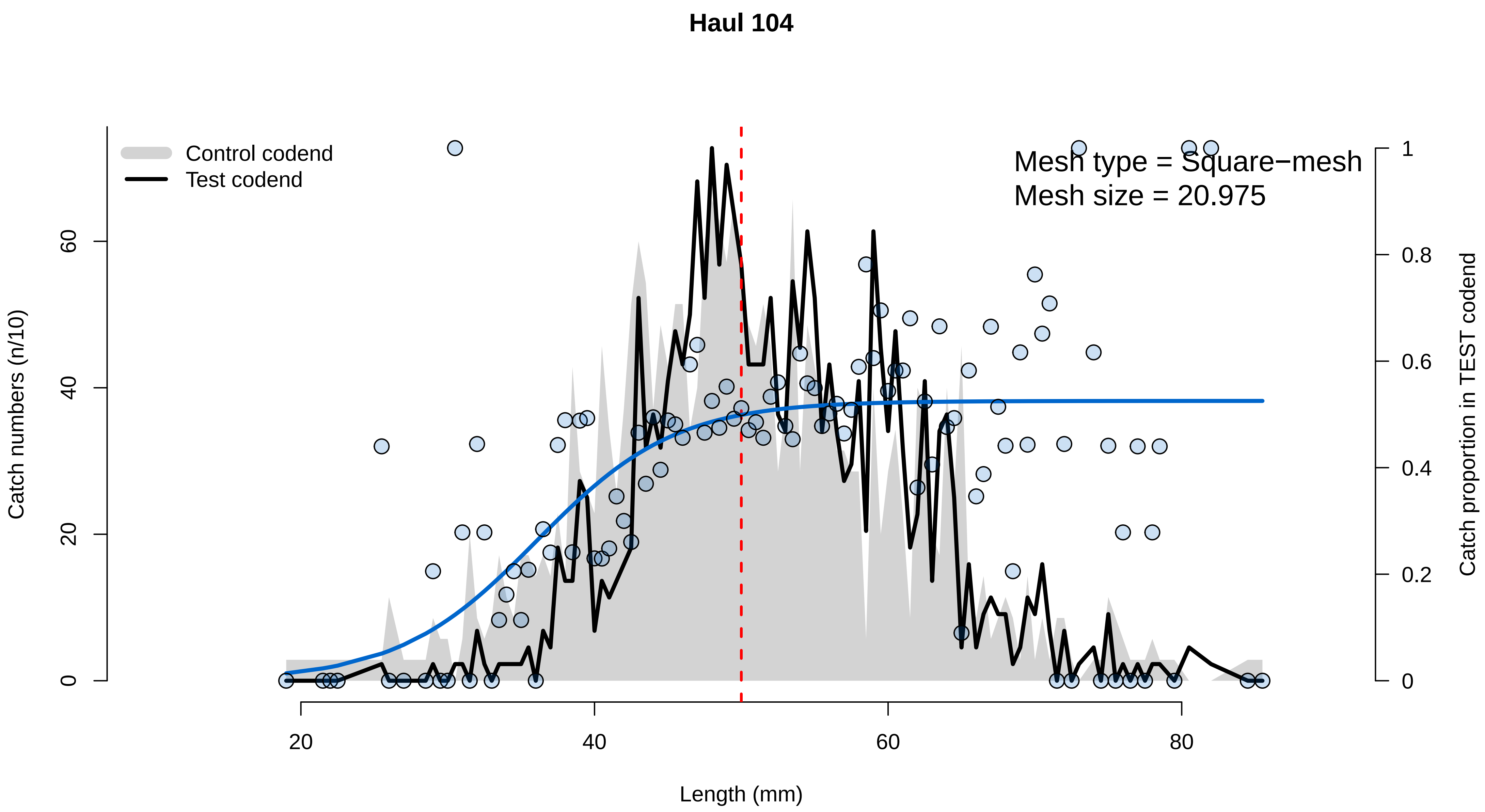
Haul 104
Control codend
Test codend
Mesh type = Square−mesh
Mesh size = 20.975
Catch numbers (n/10)
0
20
40
60
Catch proportion in TEST codend
0
0.2
0.4
0.6
0.8
1
20
40
60
80
Length (mm)

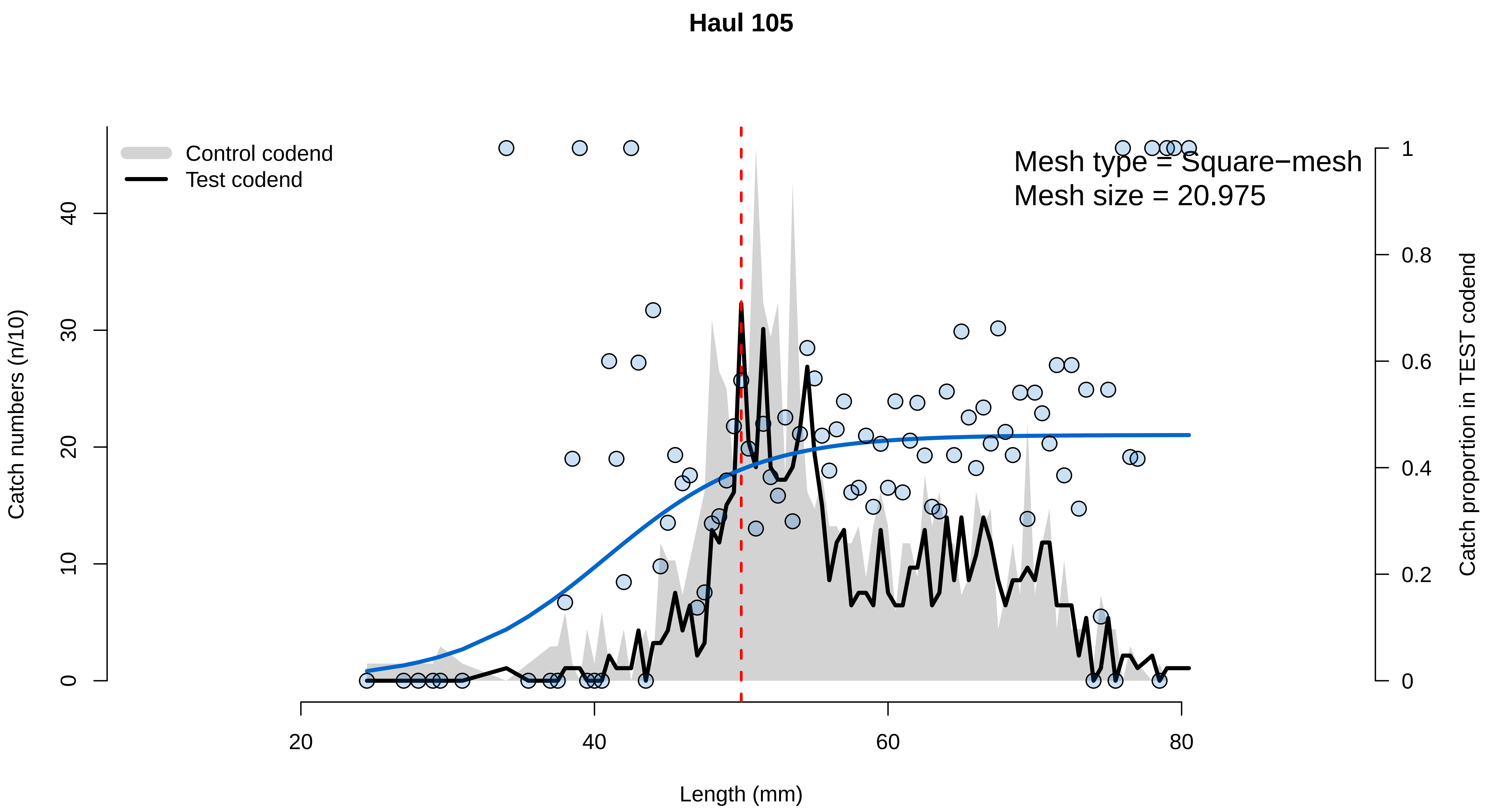

Haul 105
Control codend
Test codend
Mesh type = Square−mesh
Mesh size = 20.975
Catch numbers (n/10)
Catch proportion in TEST codend
Length (mm)
0
10
20
30
40
20
40
60
80
0
0.2
0.4
0.6
0.8
1

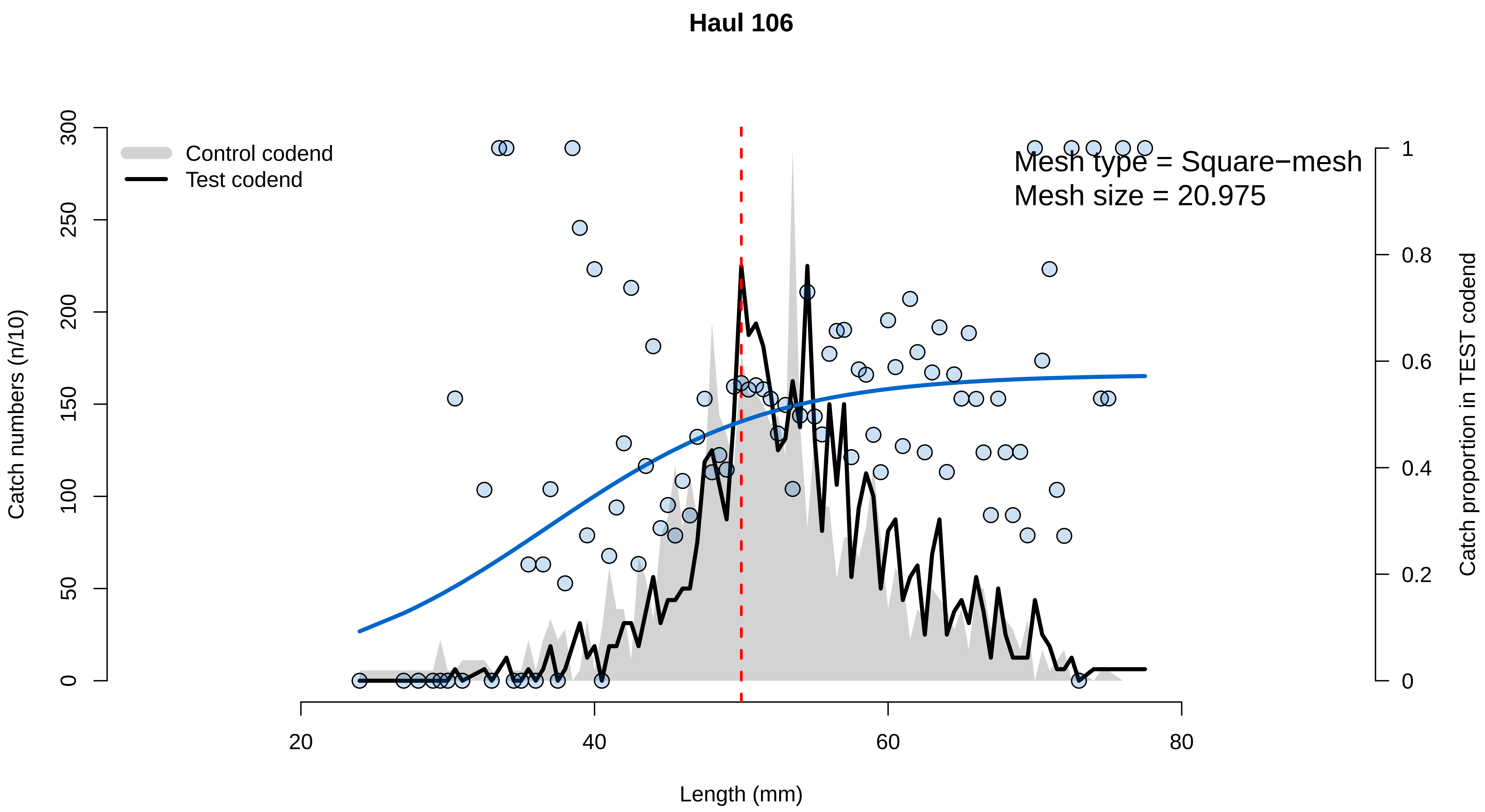

Haul 106
Control codend
Test codend
Mesh type = Square−mesh
Mesh size = 20.975
Catch numbers (n/10)
Catch proportion in TEST codend
Length (mm)
0
50
100
150
200
250
300
20
40
60
80
0
0.2
0.4
0.6
0.8
1

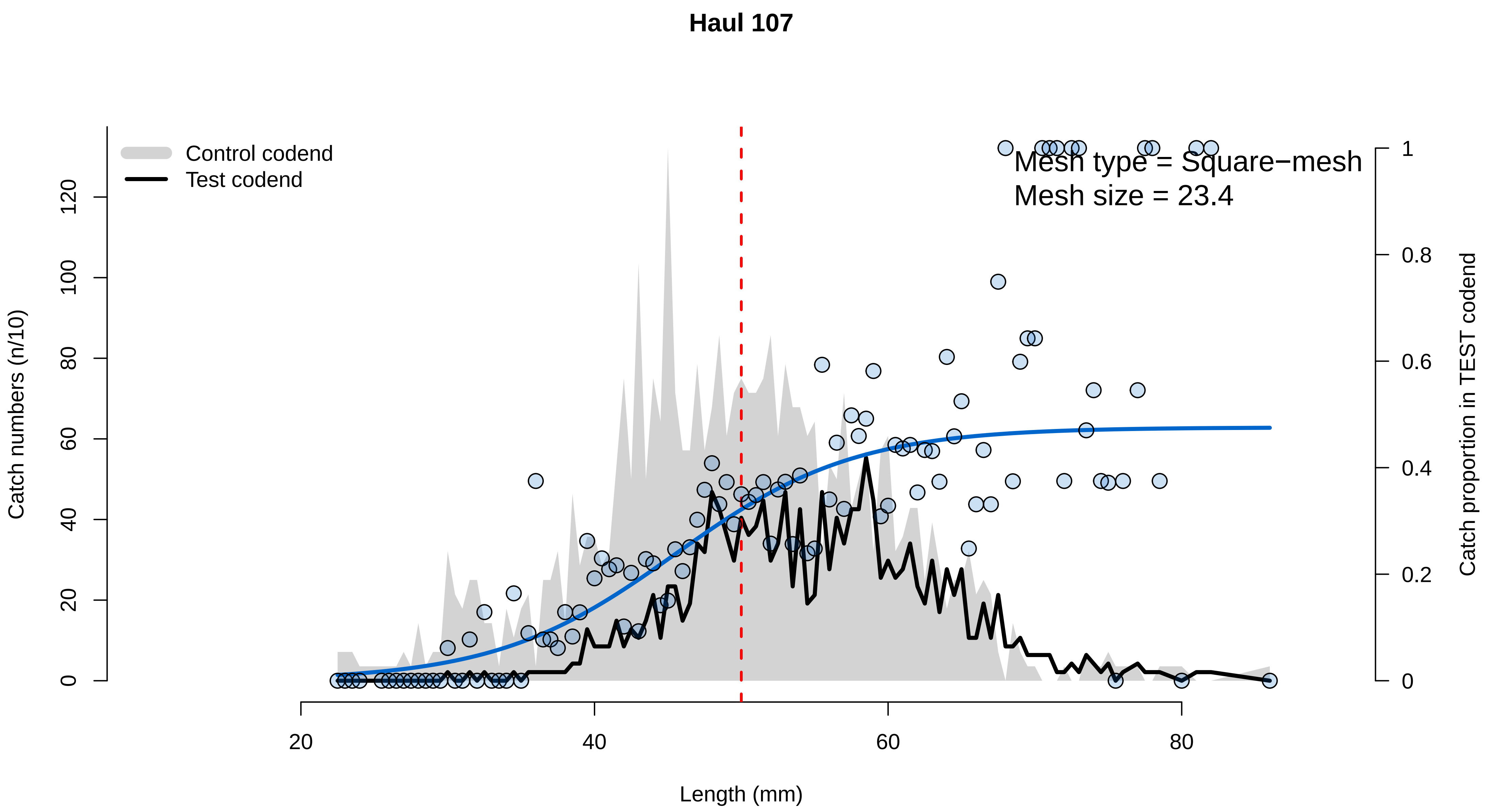

Haul 107
Control codend
Test codend
Mesh type = Square−mesh
Mesh size = 23.4
Catch numbers (n/10)
0
20
40
60
80
100
120
Catch proportion in TEST codend
0
0.2
0.4
0.6
0.8
1
20
40
60
80
Length (mm)

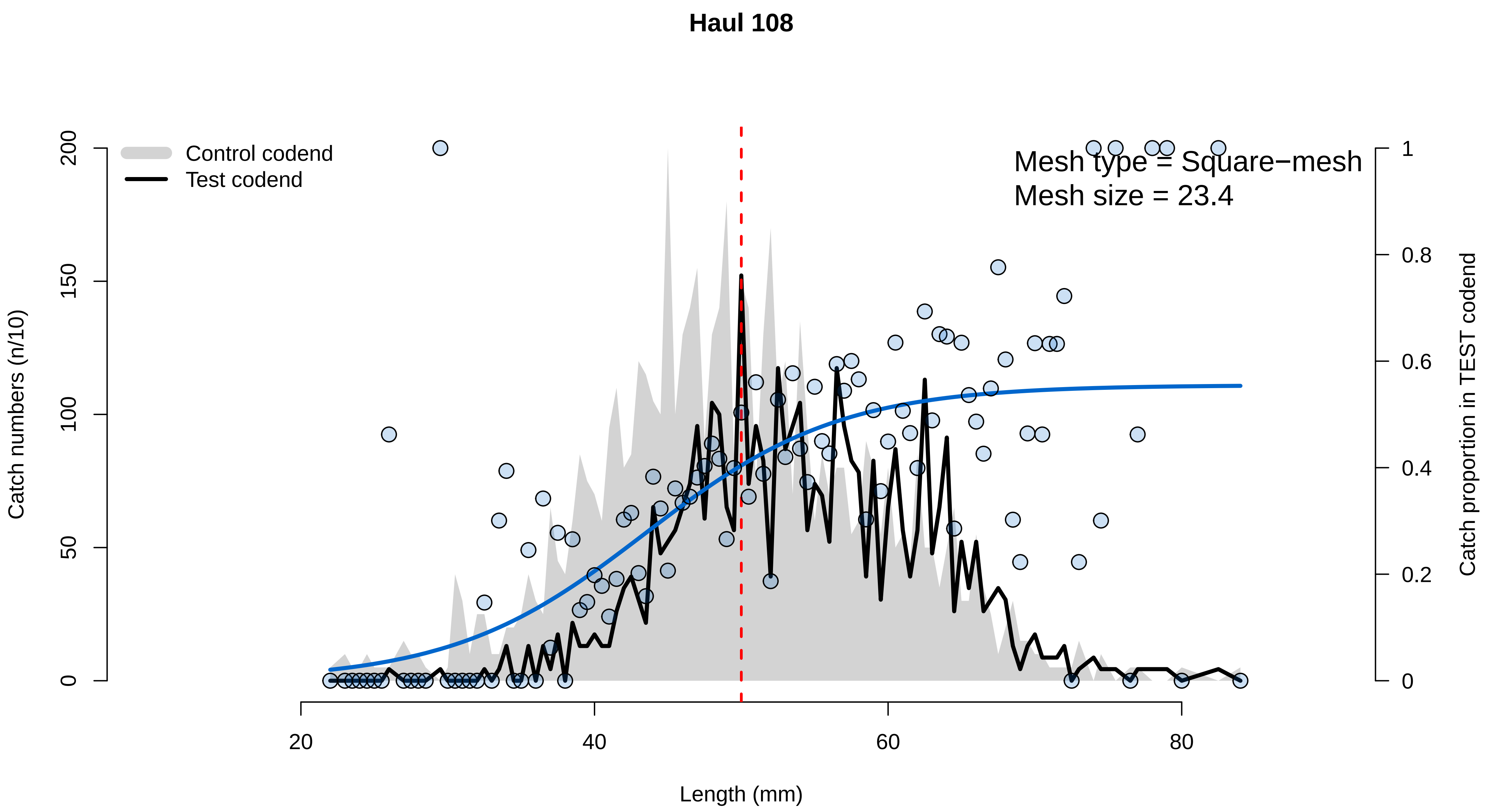
Haul 108
Control codend
Test codend
Mesh type = Square−mesh
Mesh size = 23.4
Catch numbers (n/10)
0
50
100
150
200
Catch proportion in TEST codend
0
0.2
0.4
0.6
0.8
1
20
40
60
80
Length (mm)

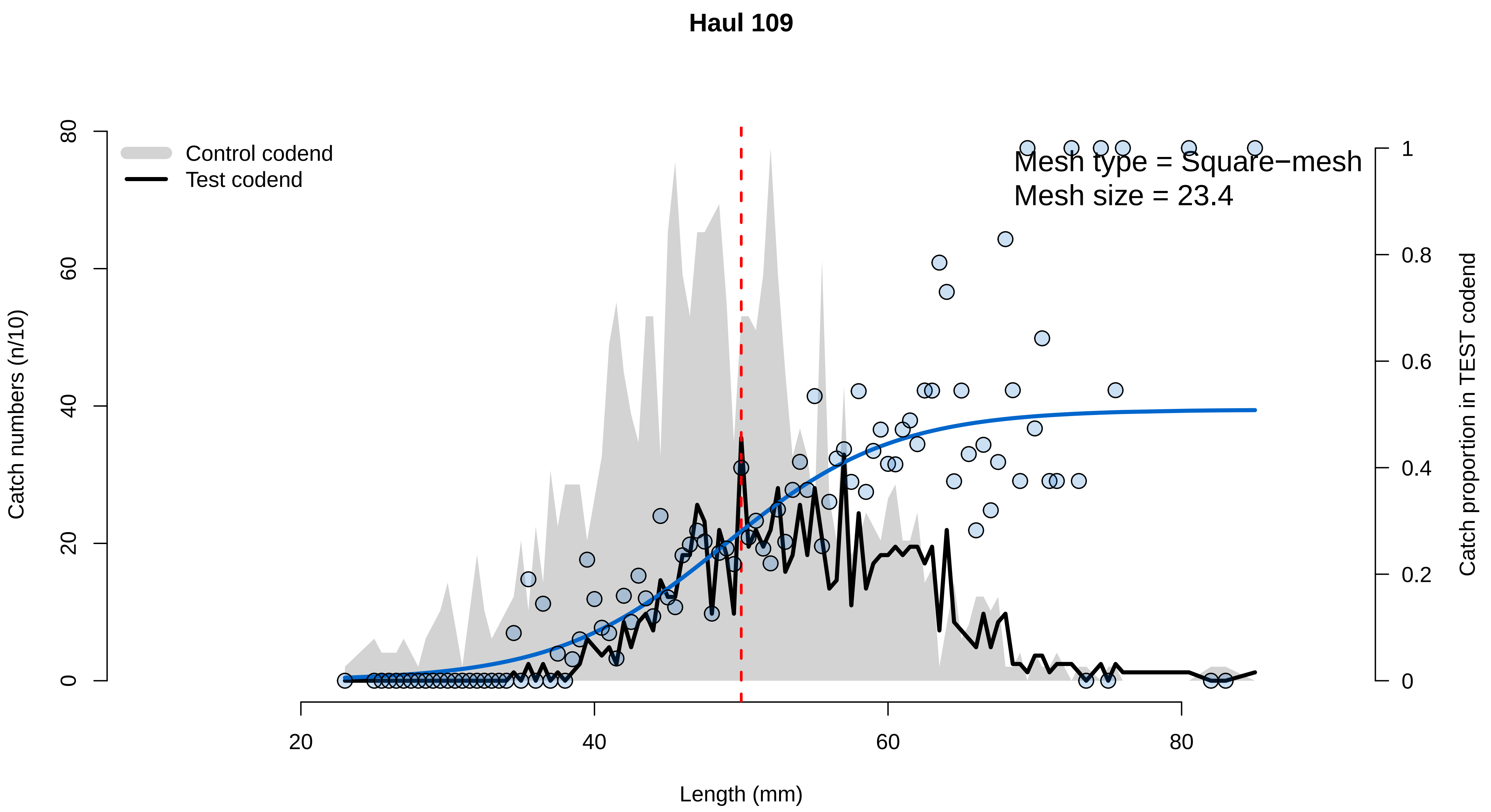
Haul 109
Control codend
Test codend
Mesh type = Square−mesh
Mesh size = 23.4
Catch numbers (n/10)
0
20
40
60
80
Catch proportion in TEST codend
0
0.2
0.4
0.6
0.8
1
20
40
60
80
Length (mm)

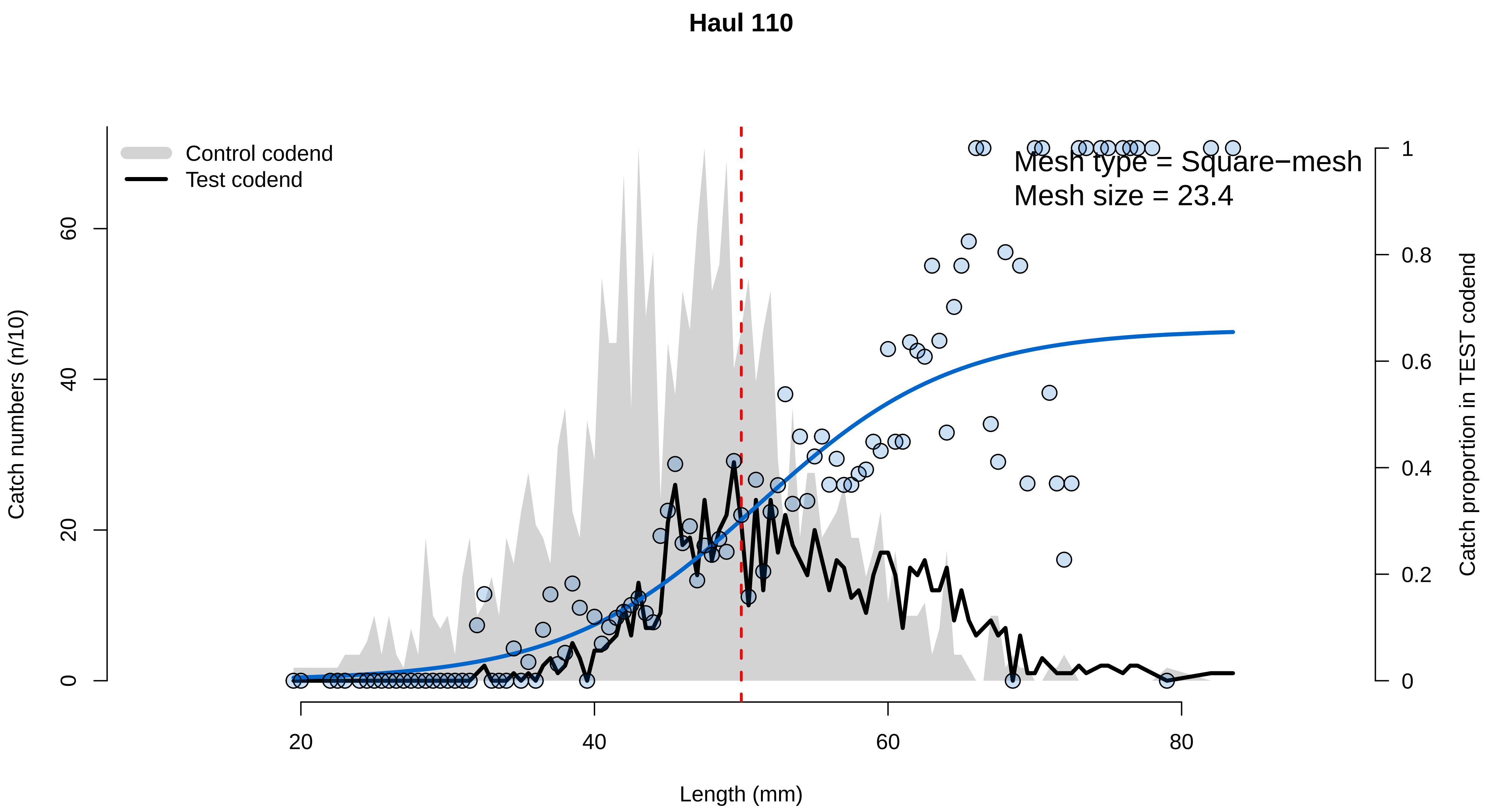
Haul 110
Control codend
Test codend
Mesh type = Square−mesh
Mesh size = 23.4
Catch numbers (n/10)
0
20
40
60
Catch proportion in TEST codend
0
0.2
0.4
0.6
0.8
1
Length (mm)
20
40
60
80

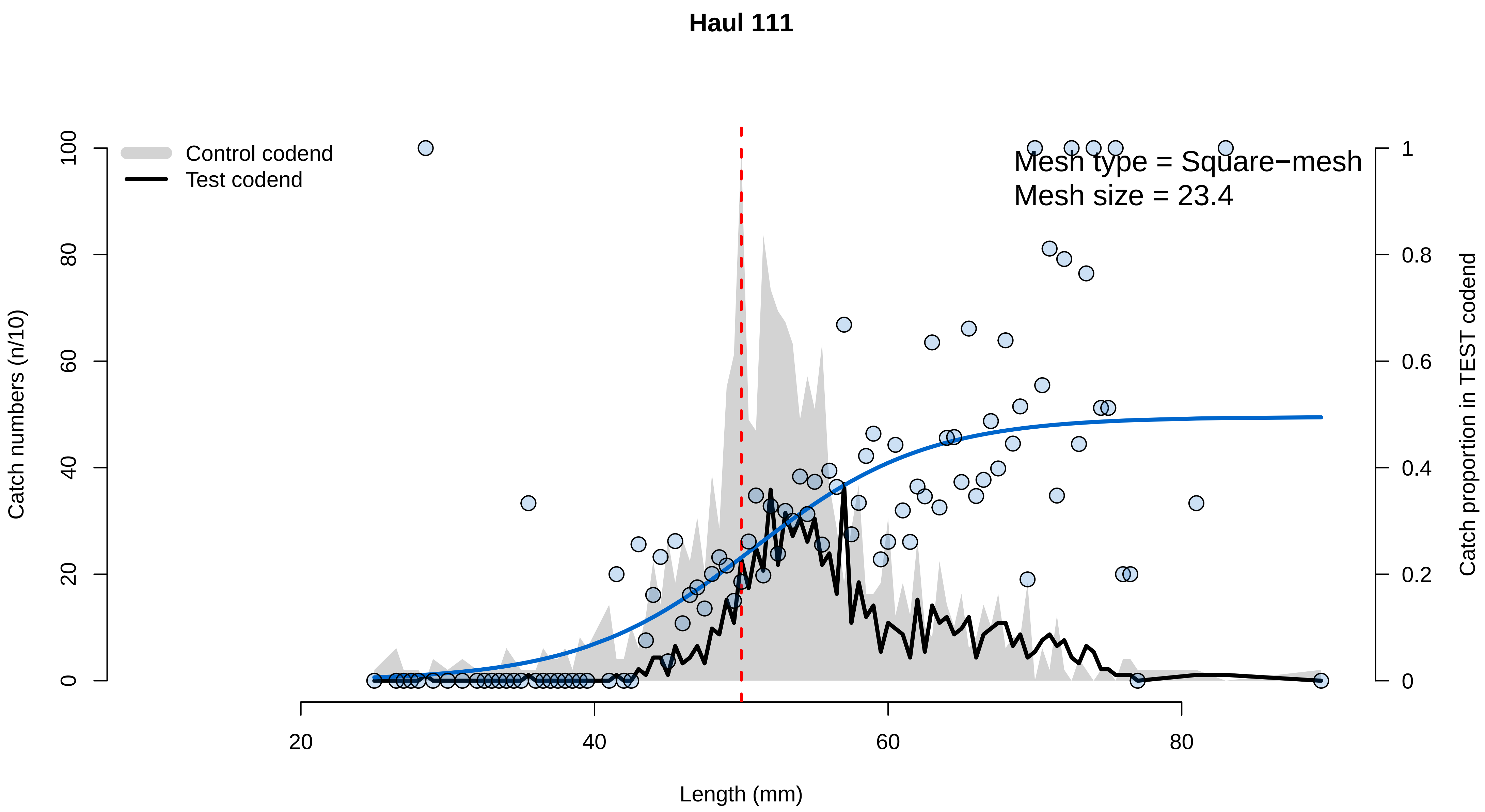

Haul 111
Control codend
Test codend
Mesh type = Square−mesh
Mesh size = 23.4
Catch numbers (n/10)
Catch proportion in TEST codend
Length (mm)
0
20
40
60
80
100
0
0.2
0.4
0.6
0.8
1
20
40
60
80

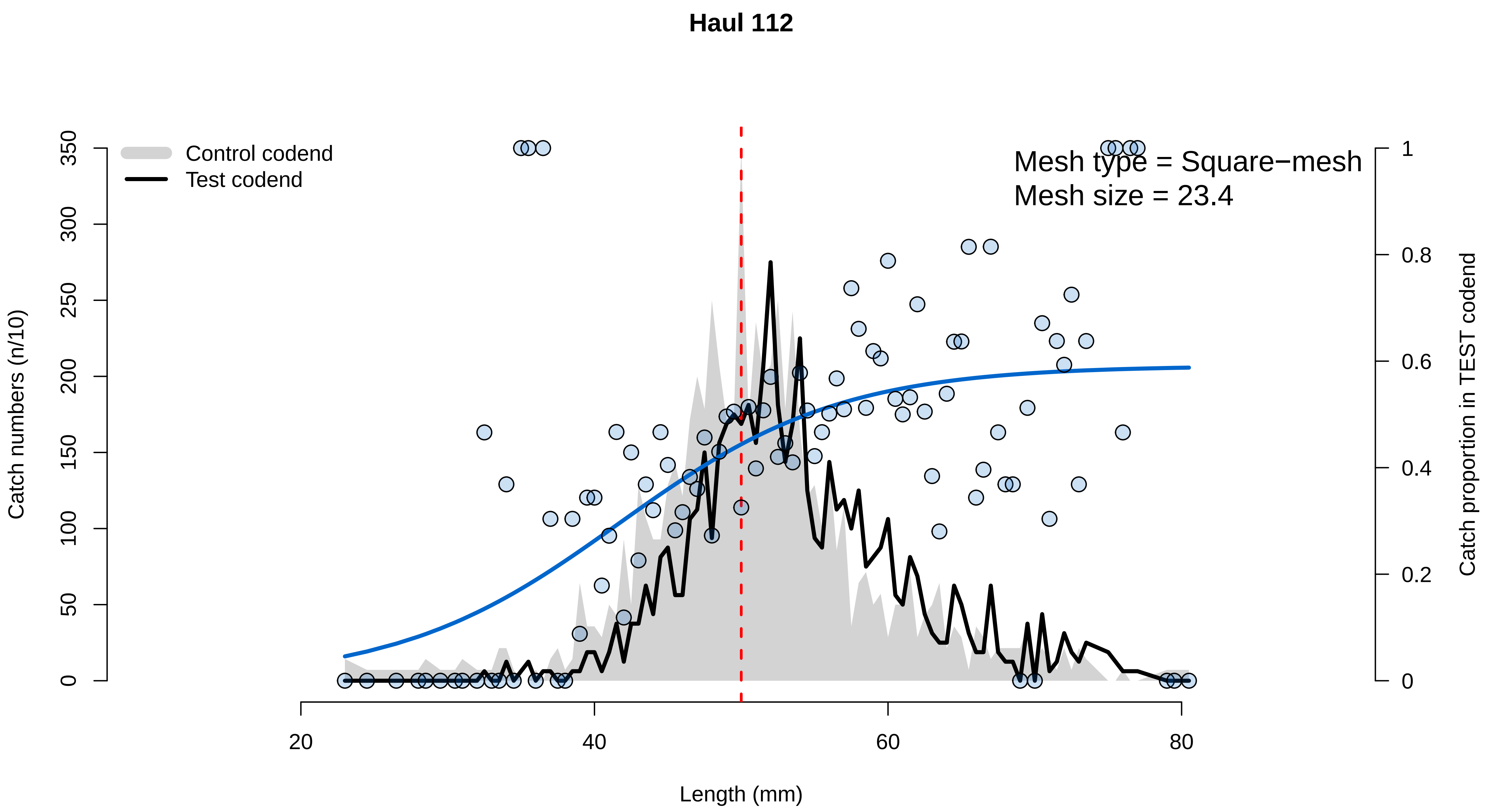

Haul 112
Control codend
Test codend
Mesh type = Square−mesh
Mesh size = 23.4
Catch numbers (n/10)
0
50
100
150
200
250
300
350
Catch proportion in TEST codend
0
0.2
0.4
0.6
0.8
1
20
40
60
80
Length (mm)

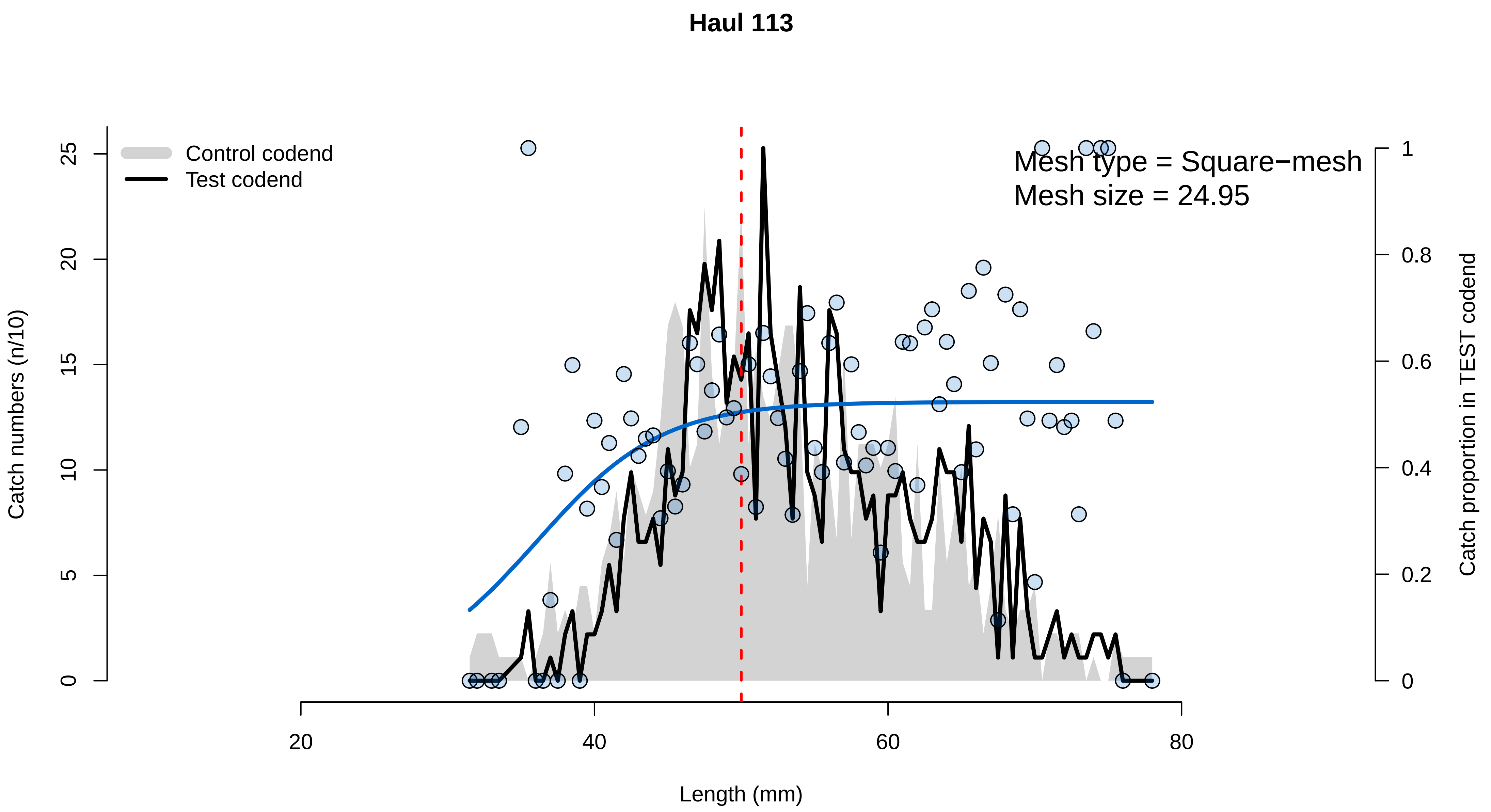
Haul 113
Control codend
Test codend
Mesh type = Square−mesh
Mesh size = 24.95
Catch numbers (n/10)
Catch proportion in TEST codend
Length (mm)
0
5
10
15
20
25
0
0.2
0.4
0.6
0.8
1
20
40
60
80

Haul 114

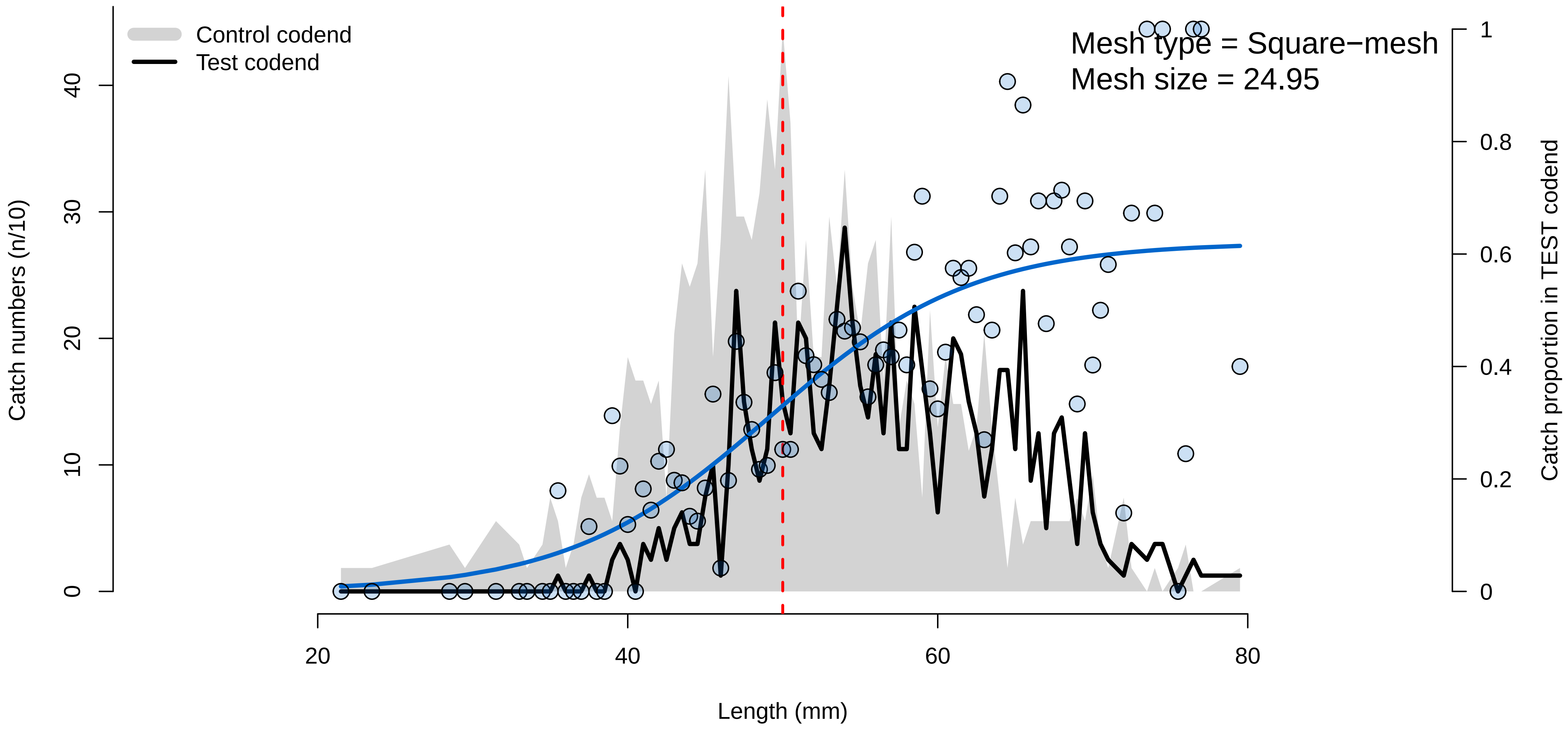

Control codend
Test codend
Mesh type = Square−mesh
Mesh size = 24.95
Catch numbers (n/10)
0
10
20
30
40
Catch proportion in TEST codend
0
0.2
0.4
0.6
0.8
1
20
40
60
80
Length (mm)

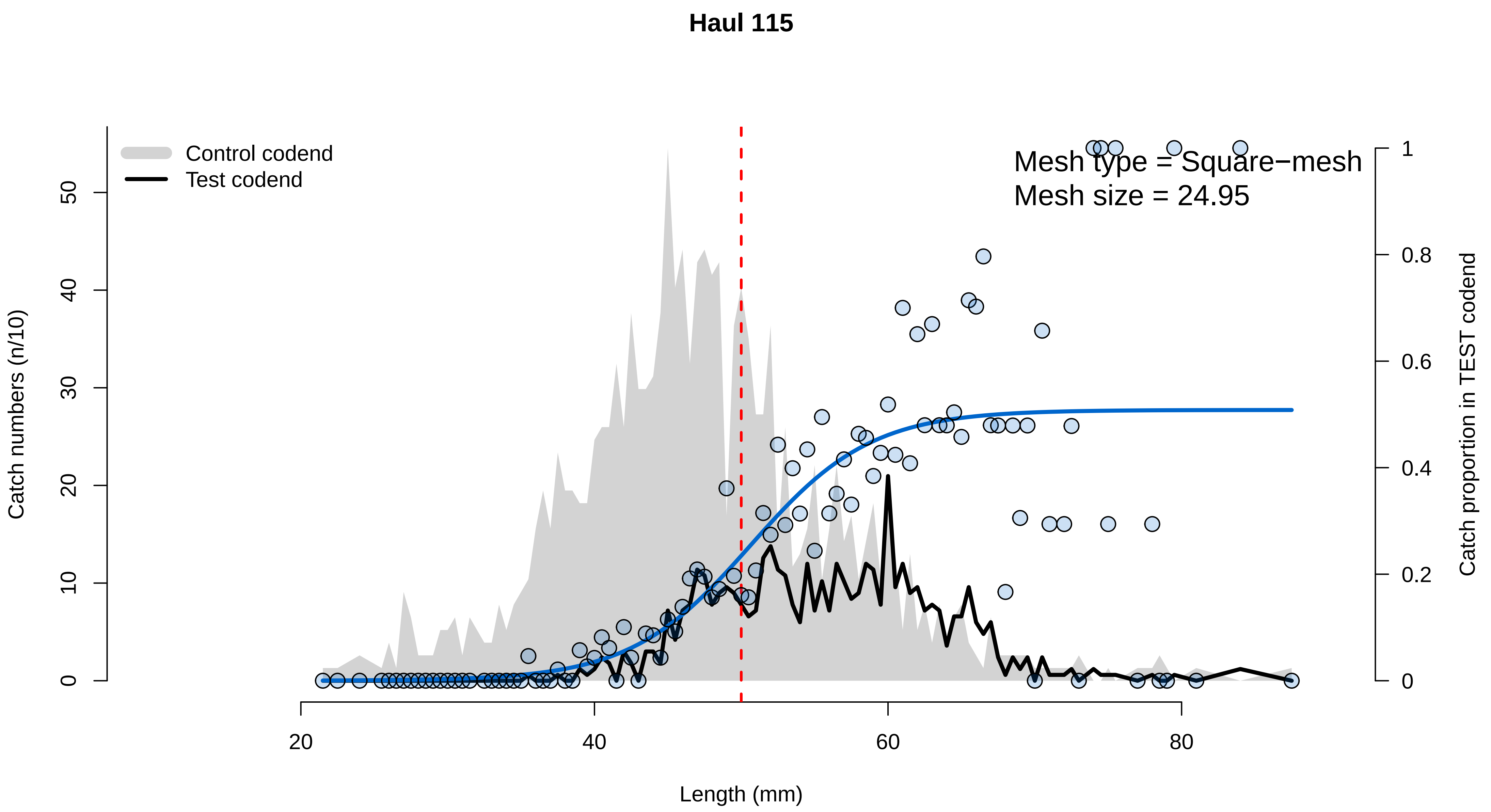

Haul 115
Control codend
Test codend
Mesh type = Square−mesh
Mesh size = 24.95
Catch numbers (n/10)
0
10
20
30
40
50
Catch proportion in TEST codend
0
0.2
0.4
0.6
0.8
1
20
40
60
80
Length (mm)

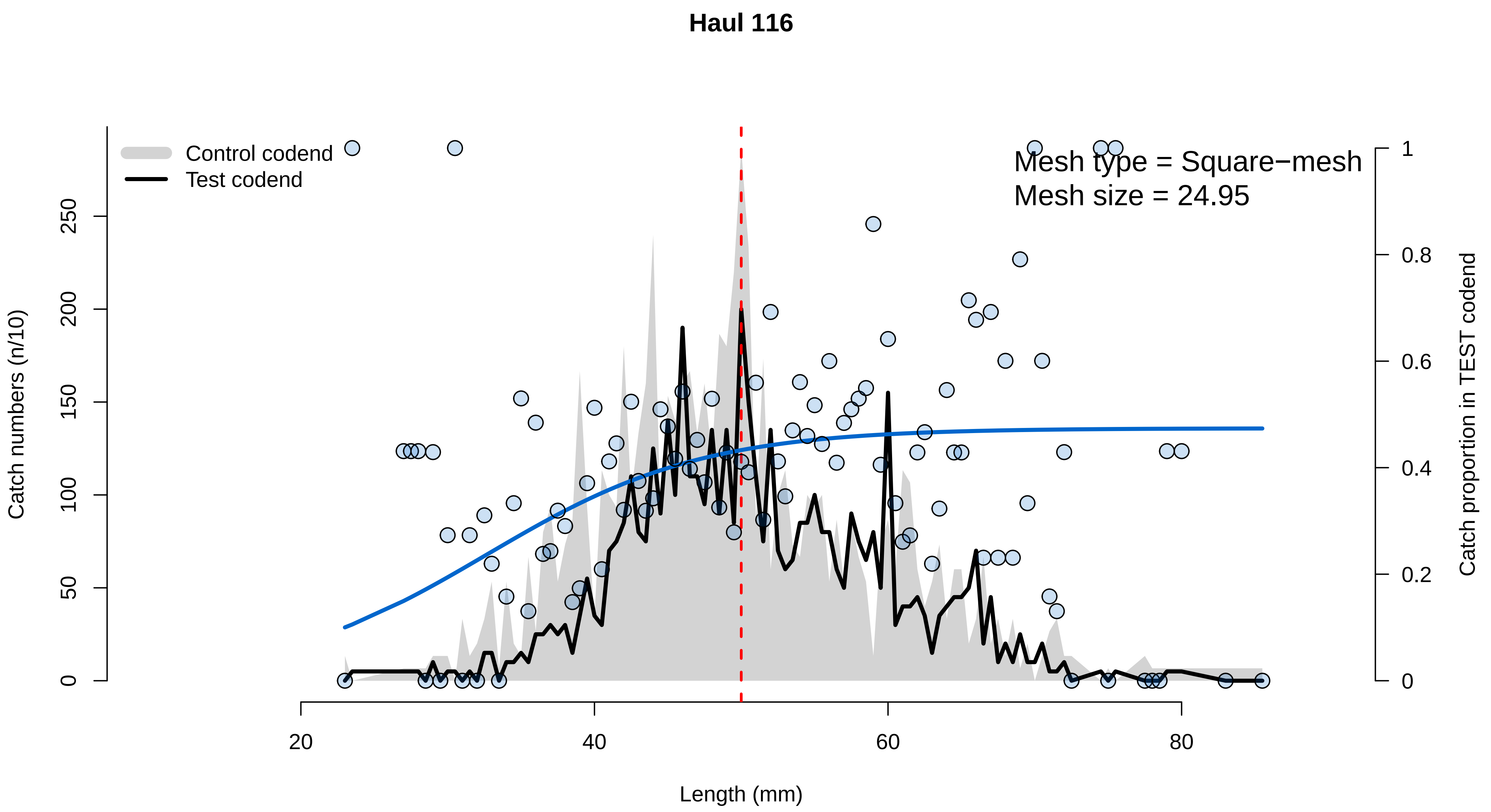
Haul 116
Control codend
Test codend
Mesh type = Square−mesh
Mesh size = 24.95
Catch numbers (n/10)
0
50
100
150
200
250
Catch proportion in TEST codend
0
0.2
0.4
0.6
0.8
1
Length (mm)
20
40
60
80

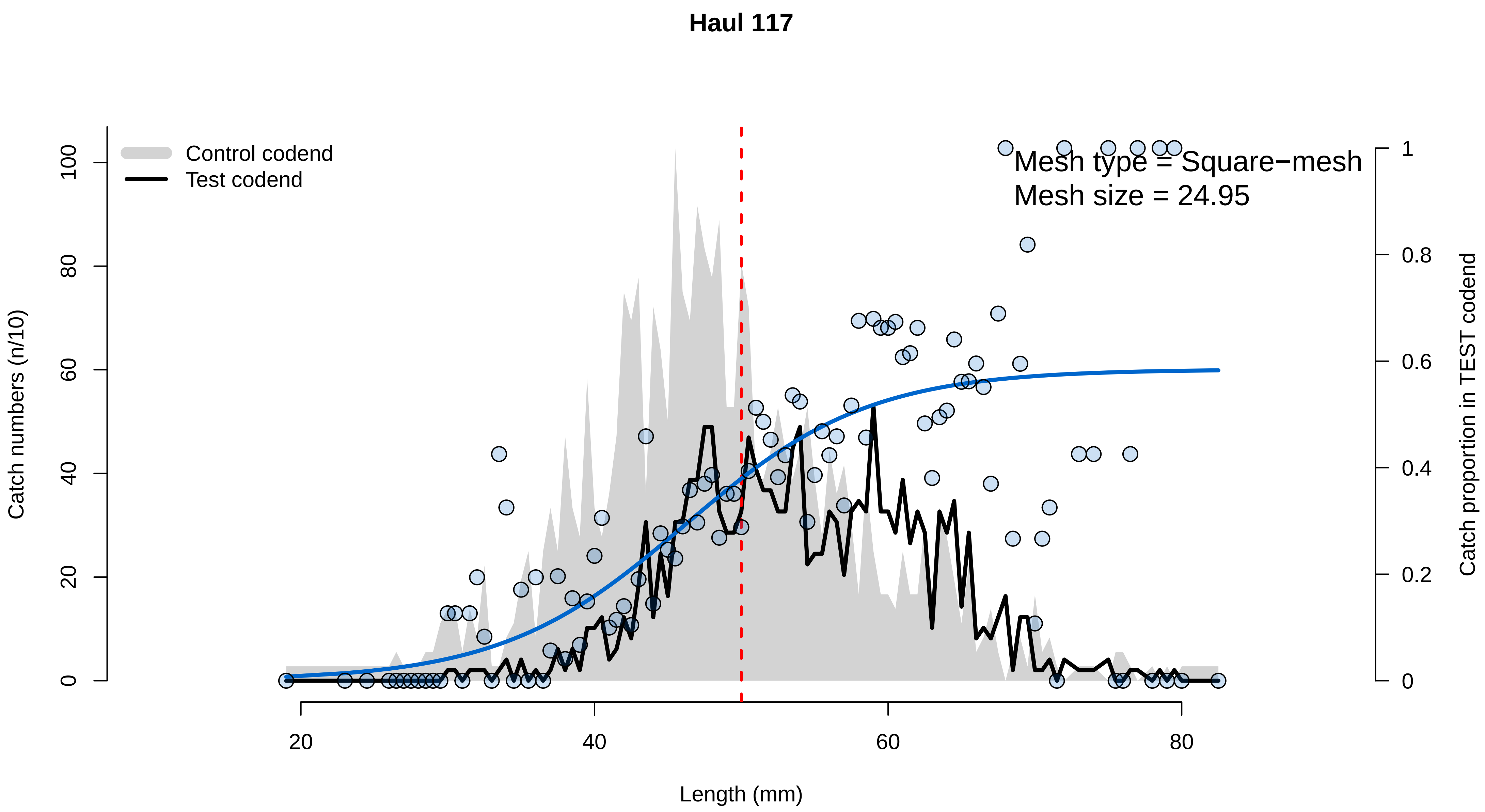
Haul 117
Control codend
Test codend
Mesh type = Square−mesh
Mesh size = 24.95
Catch numbers (n/10)
Catch proportion in TEST codend
Length (mm)
0
20
40
60
80
100
0
0.2
0.4
0.6
0.8
1
20
40
60
80

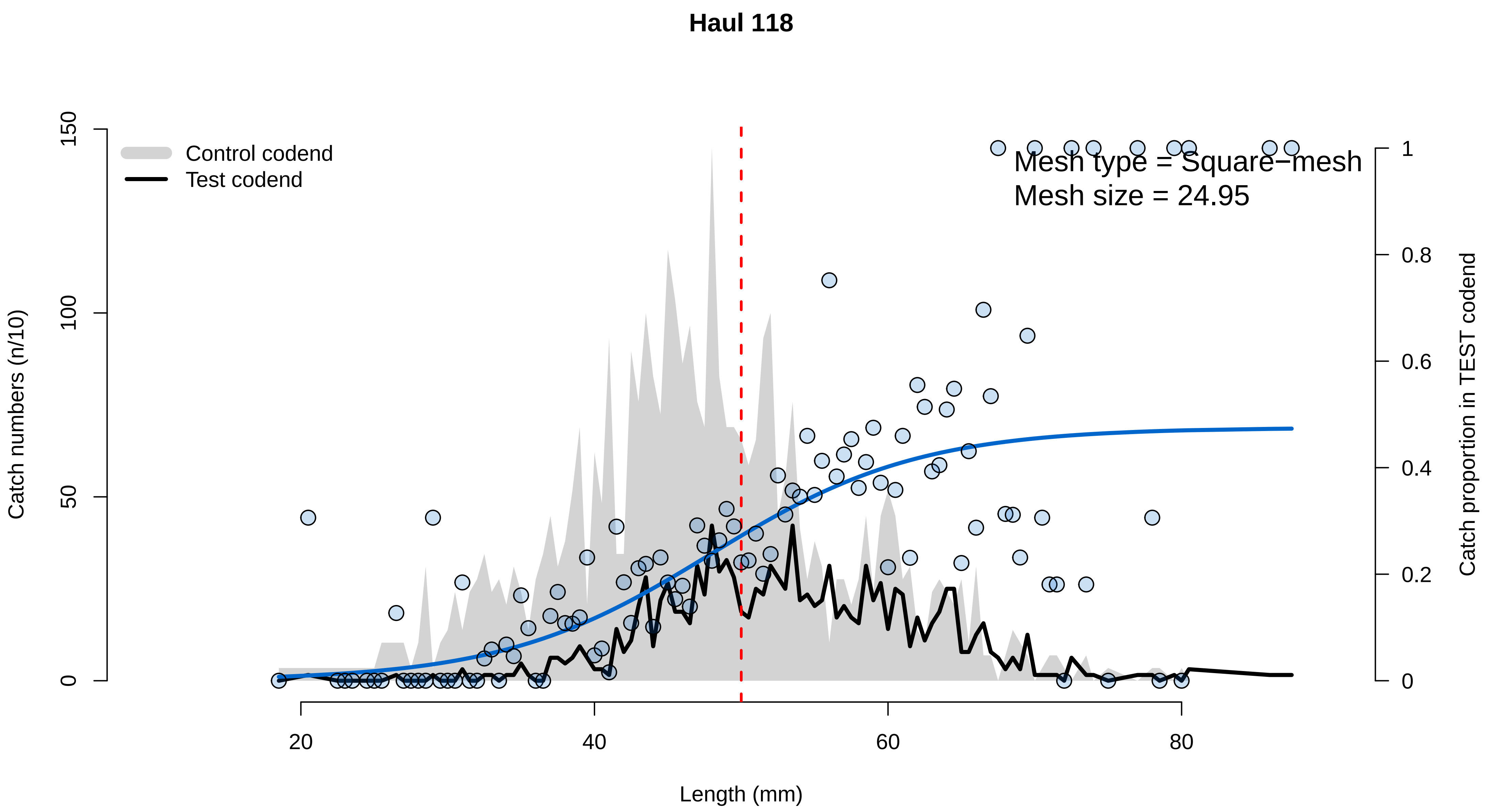
Haul 118
Control codend
Test codend
Mesh type = Square−mesh
Mesh size = 24.95
Catch numbers (n/10)
0
50
100
150
Catch proportion in TEST codend
0
0.2
0.4
0.6
0.8
1
20
40
60
80
Length (mm)

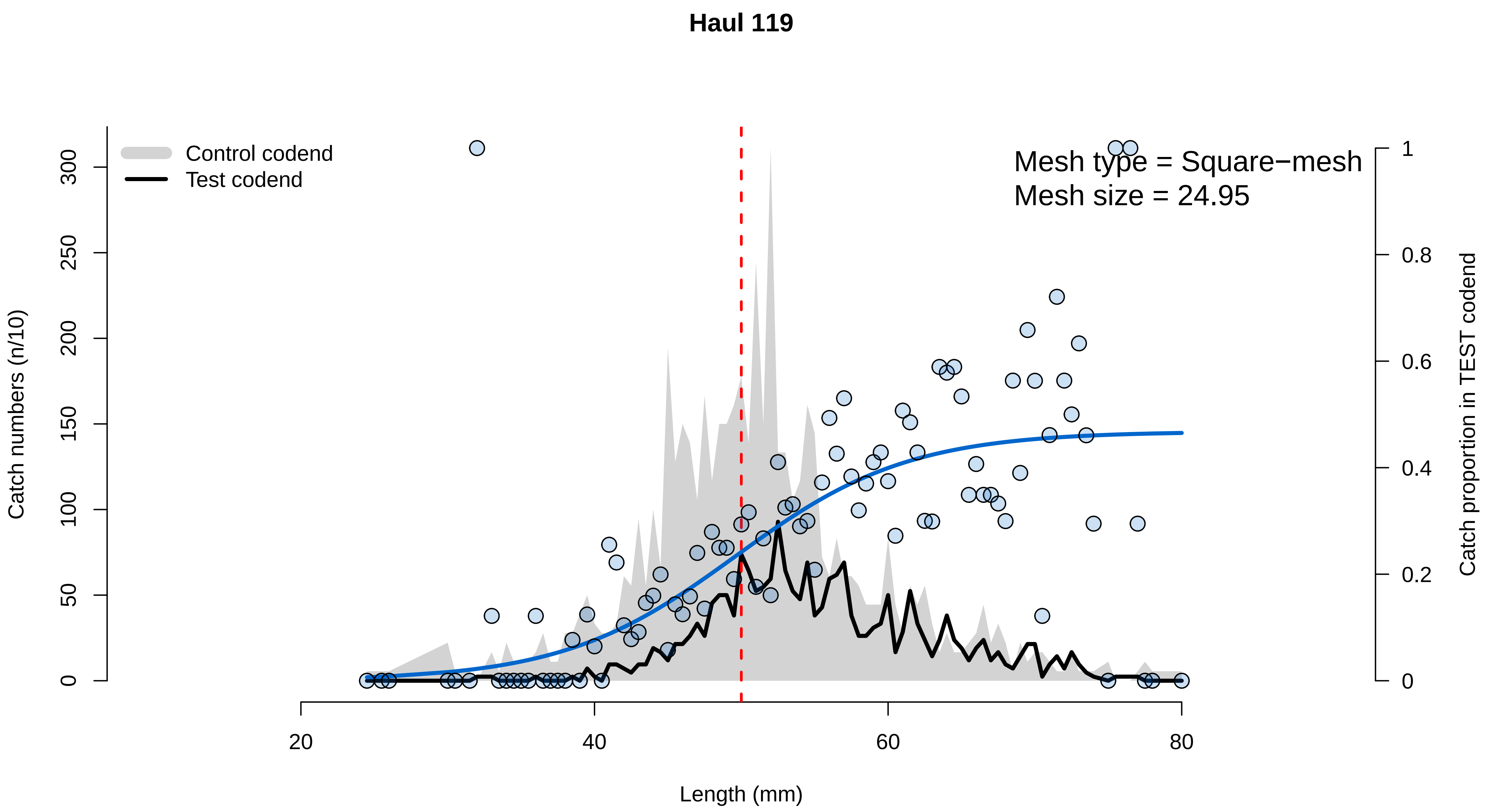

Haul 119
Control codend
Test codend
Mesh type = Square−mesh
Mesh size = 24.95
Catch numbers (n/10)
0
50
100
150
200
250
300
Catch proportion in TEST codend
0
0.2
0.4
0.6
0.8
1
Length (mm)
20
40
60
80

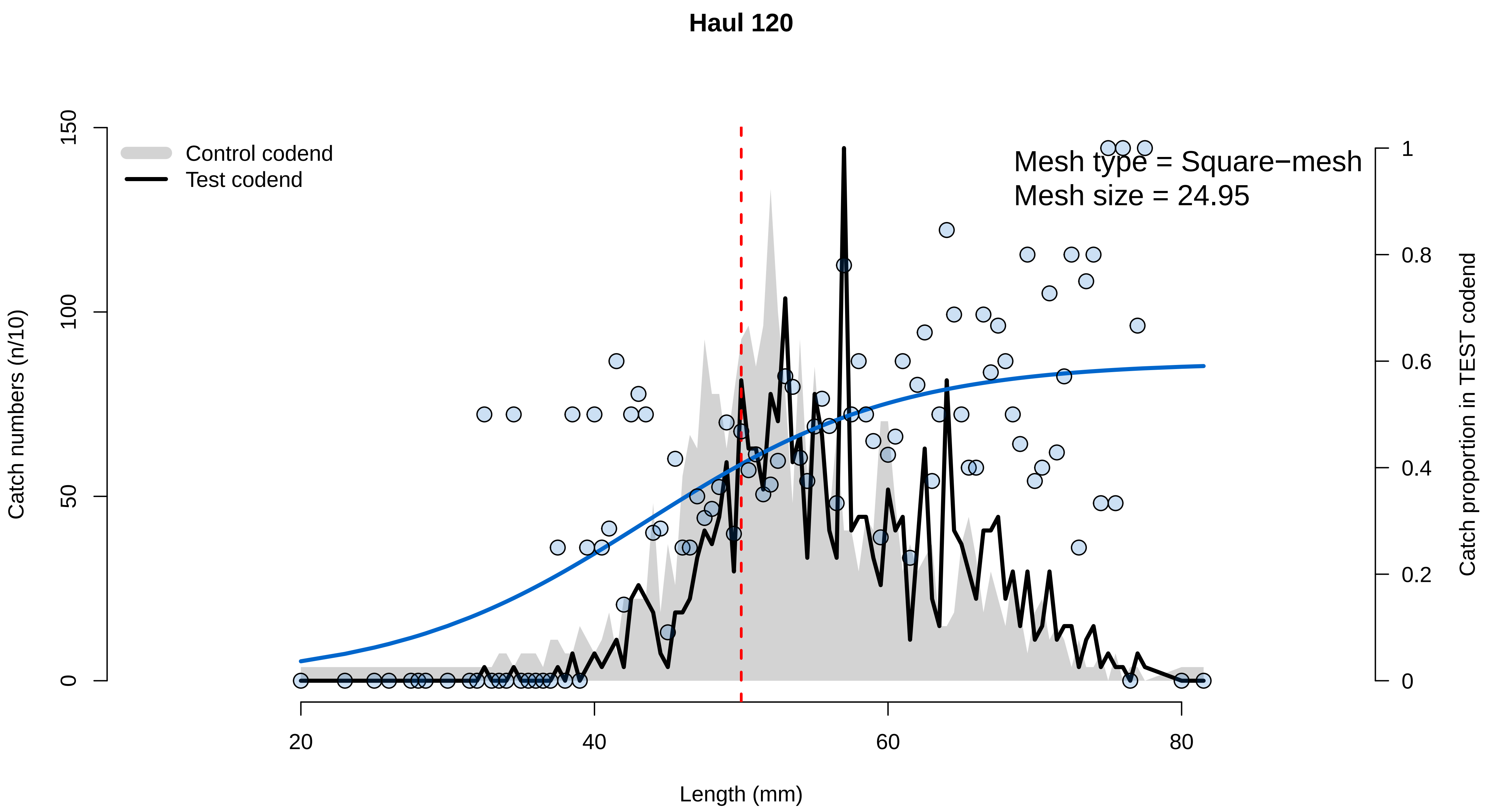

Haul 120
Control codend
Test codend
Mesh type = Square−mesh
Mesh size = 24.95
Catch numbers (n/10)
0
50
100
150
Catch proportion in TEST codend
0
0.2
0.4
0.6
0.8
1
Length (mm)
20
40
60
80

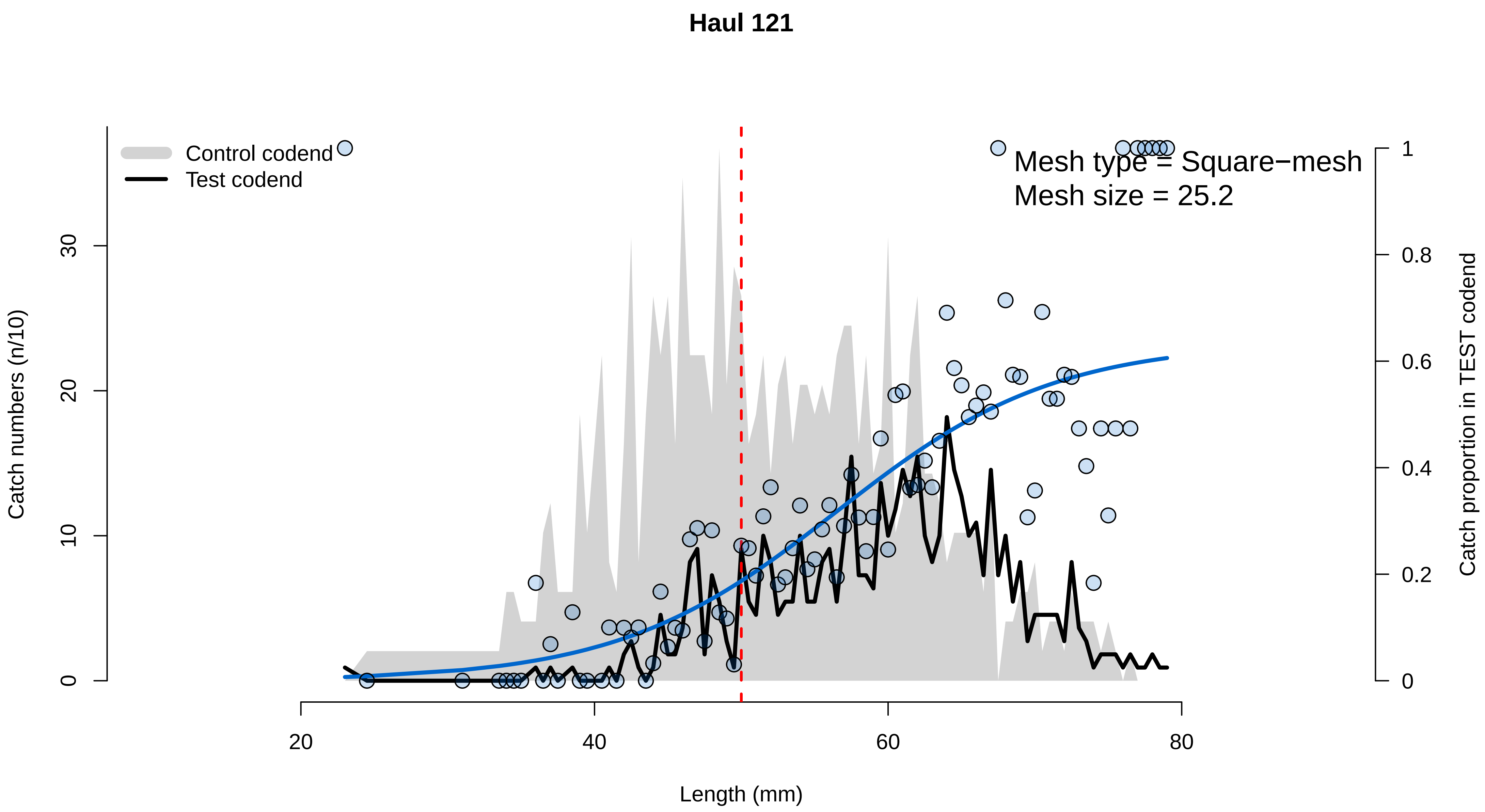

Haul 121
Control codend
Test codend
Mesh type = Square−mesh
Mesh size = 25.2
Catch numbers (n/10)
0
10
20
30
Catch proportion in TEST codend
0
0.2
0.4
0.6
0.8
1
20
40
60
80
Length (mm)

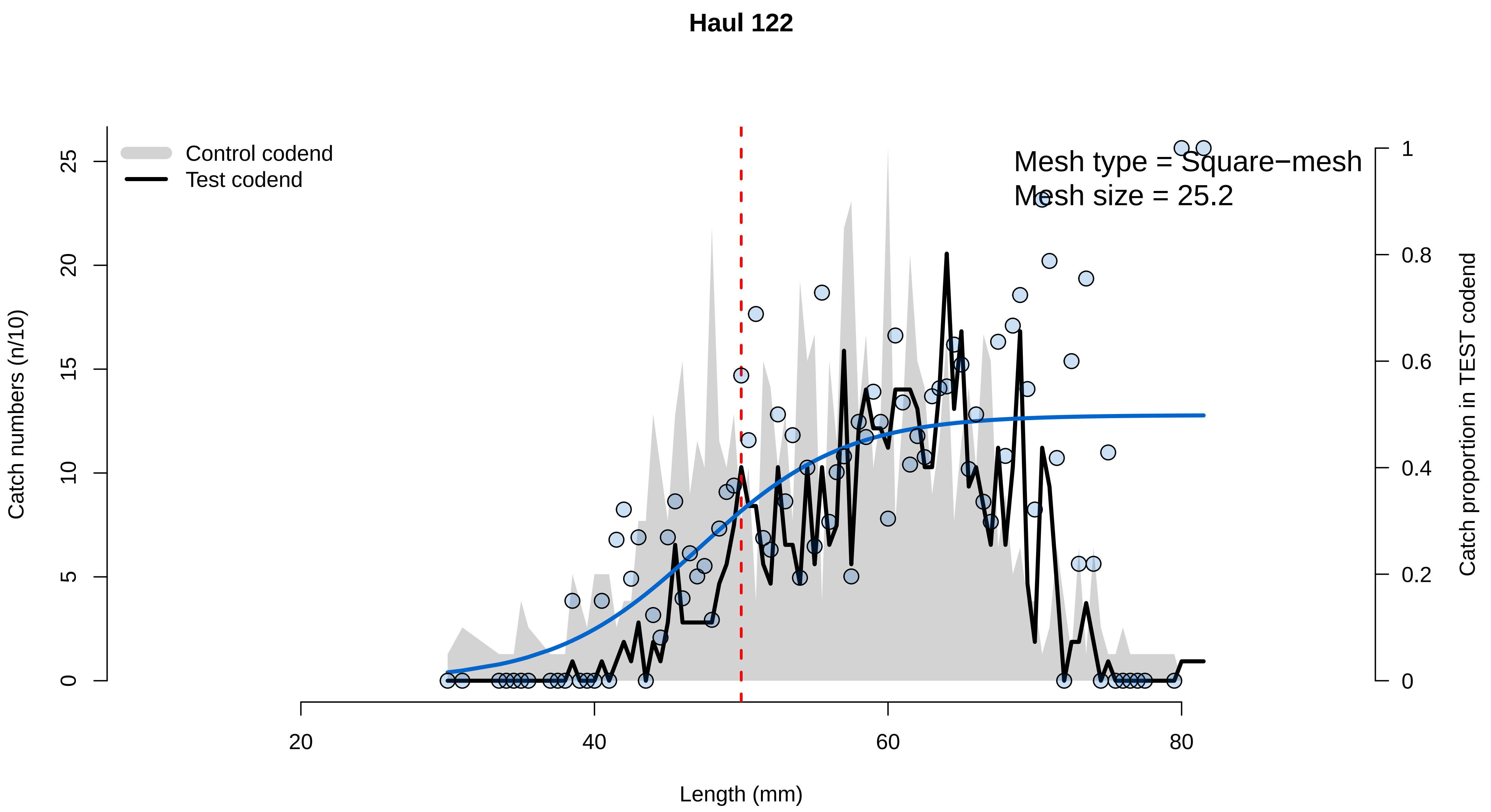

Haul 122
Control codend
Test codend
Mesh type = Square−mesh
Mesh size = 25.2
Catch numbers (n/10)
Catch proportion in TEST codend
Length (mm)
0
5
10
15
20
25
0
0.2
0.4
0.6
0.8
1
20
40
60
80

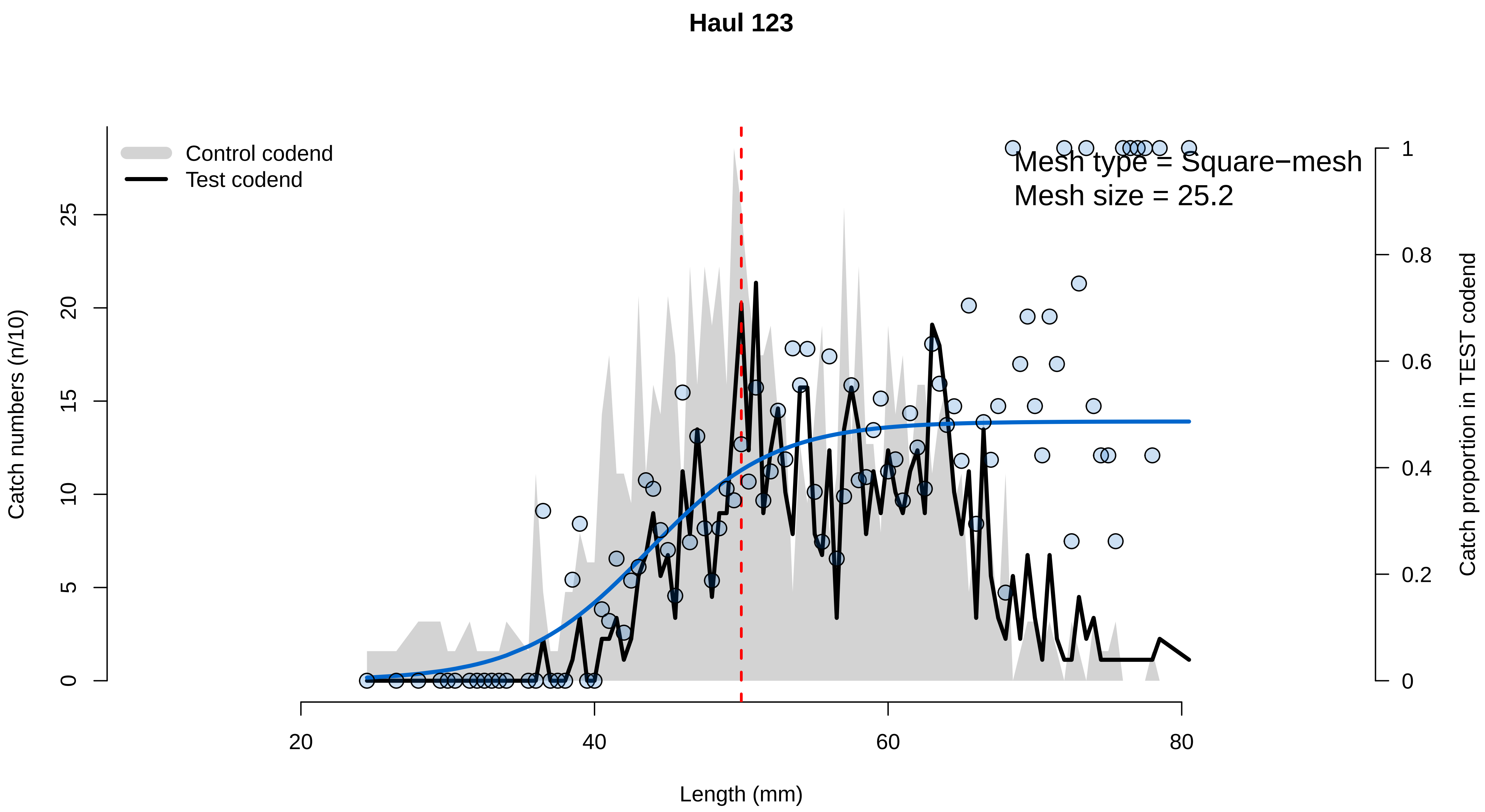

Haul 123
Control codend
Test codend
Mesh type = Square−mesh
Mesh size = 25.2
Catch numbers (n/10)
Catch proportion in TEST codend
Length (mm)
0
5
10
15
20
25
0
0.2
0.4
0.6
0.8
1
20
40
60
80

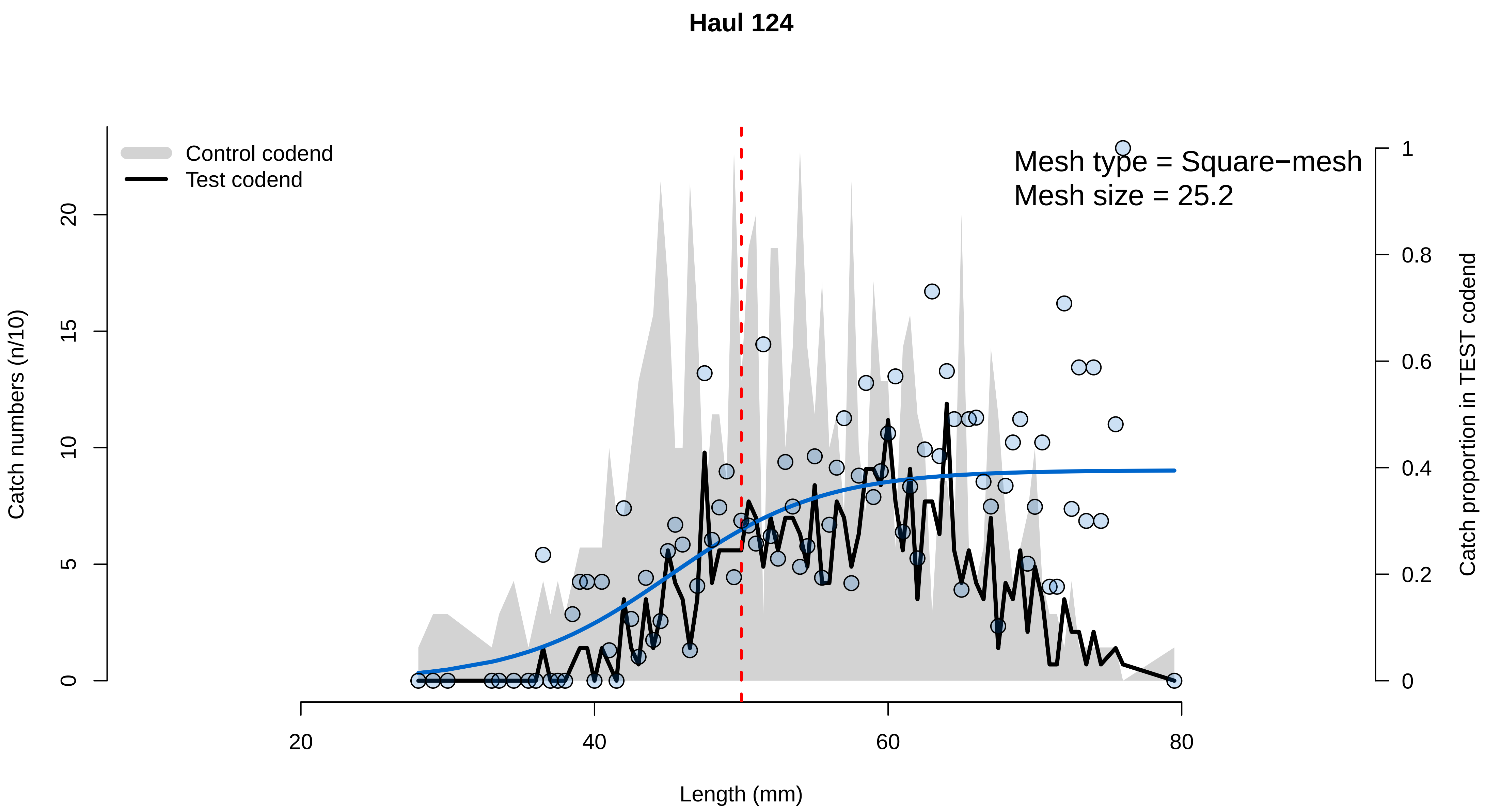

Haul 124
Control codend
Test codend
Mesh type = Square−mesh
Mesh size = 25.2
Catch numbers (n/10)
0
5
10
15
20
20
40
60
80
Length (mm)
Catch proportion in TEST codend
0
0.2
0.4
0.6
0.8
1

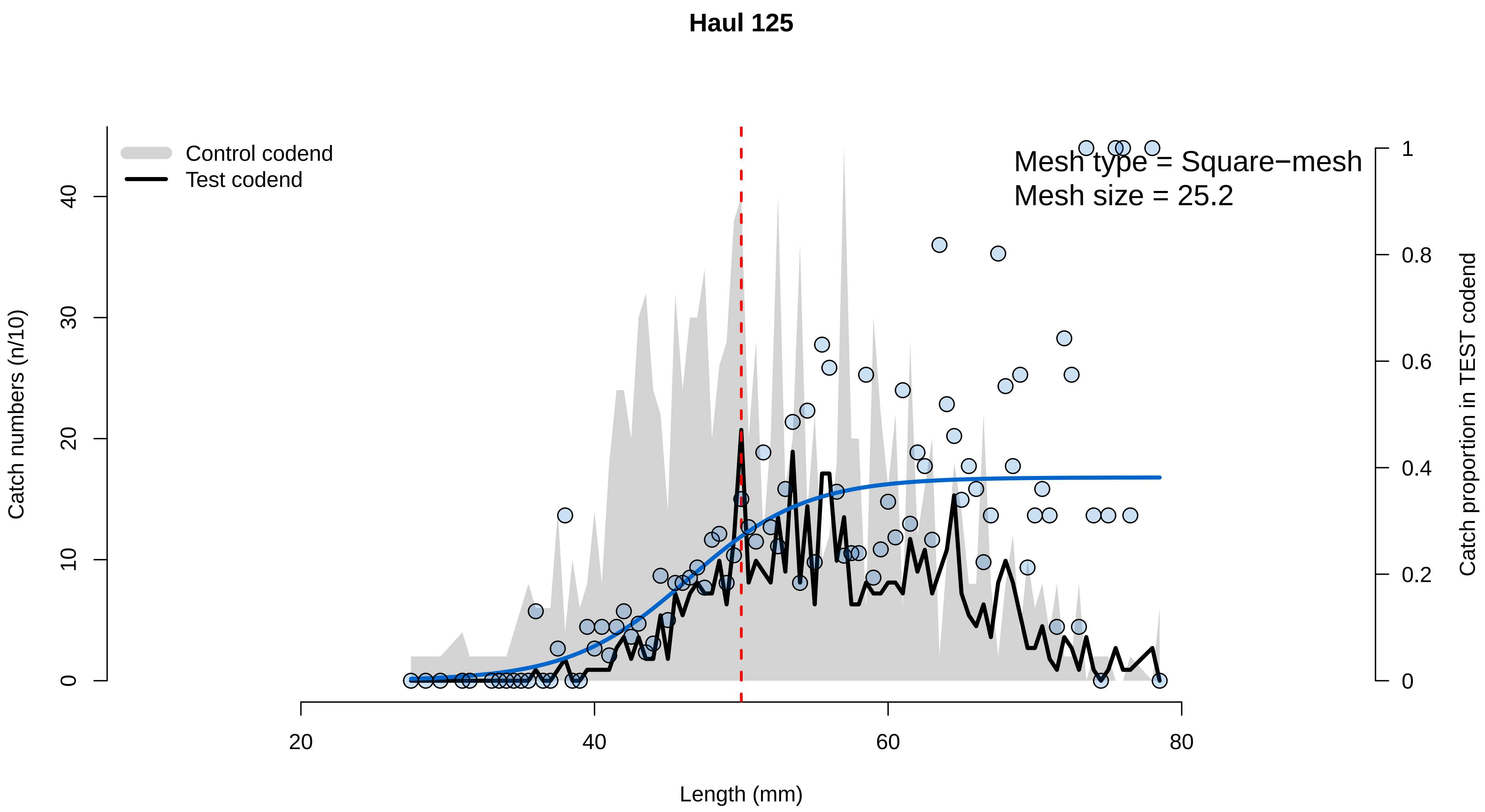

Haul 125
Control codend
Test codend
Mesh type = Square−mesh
Mesh size = 25.2
Catch numbers (n/10)
0
10
20
30
40
Catch proportion in TEST codend
0
0.2
0.4
0.6
0.8
1
20
40
60
80
Length (mm)

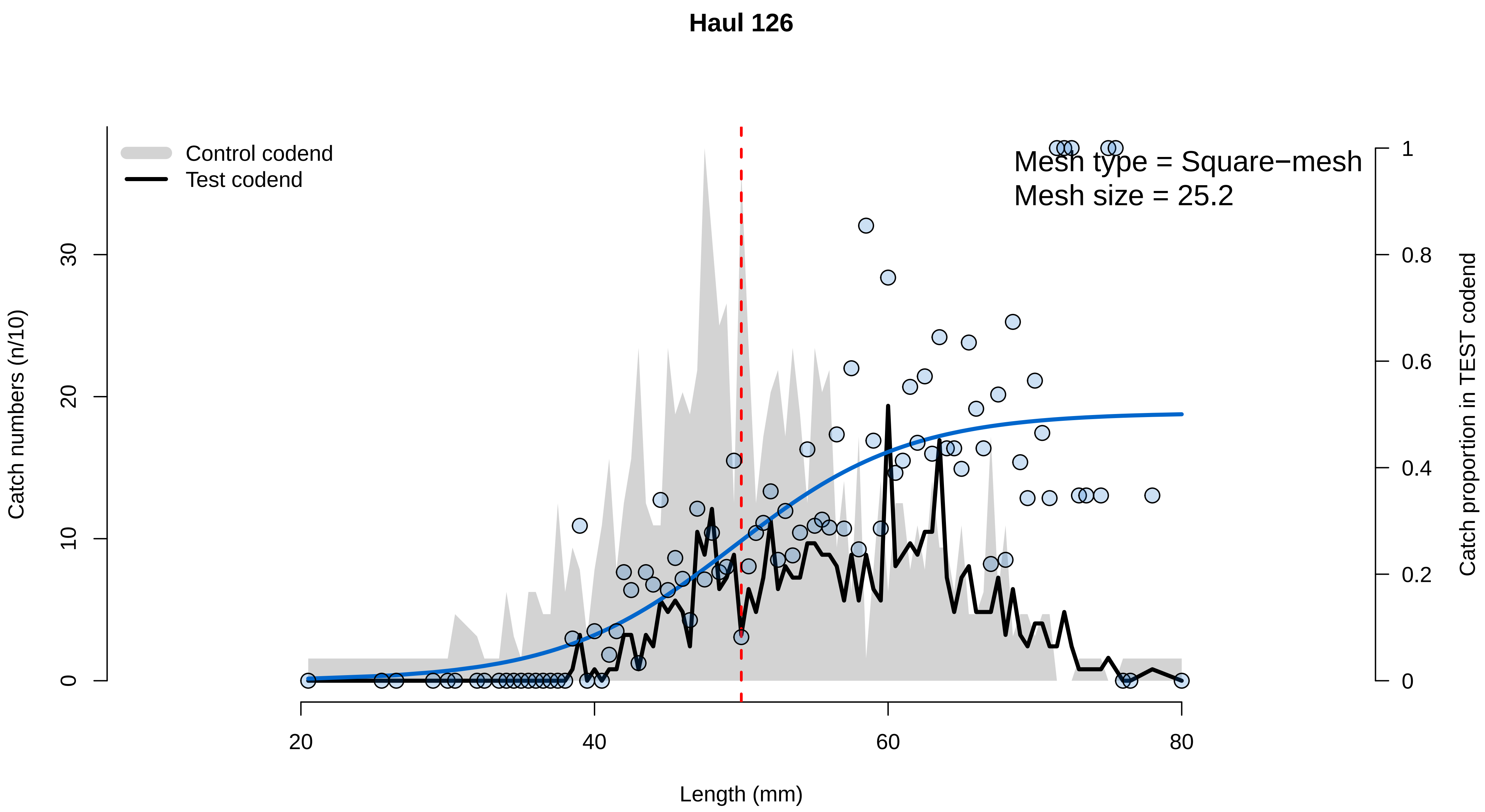

Haul 126
Control codend
Test codend
Mesh type = Square−mesh
Mesh size = 25.2
Catch numbers (n/10)
Catch proportion in TEST codend
Length (mm)
0
10
20
30
20
40
60
80
0
0.2
0.4
0.6
0.8
1

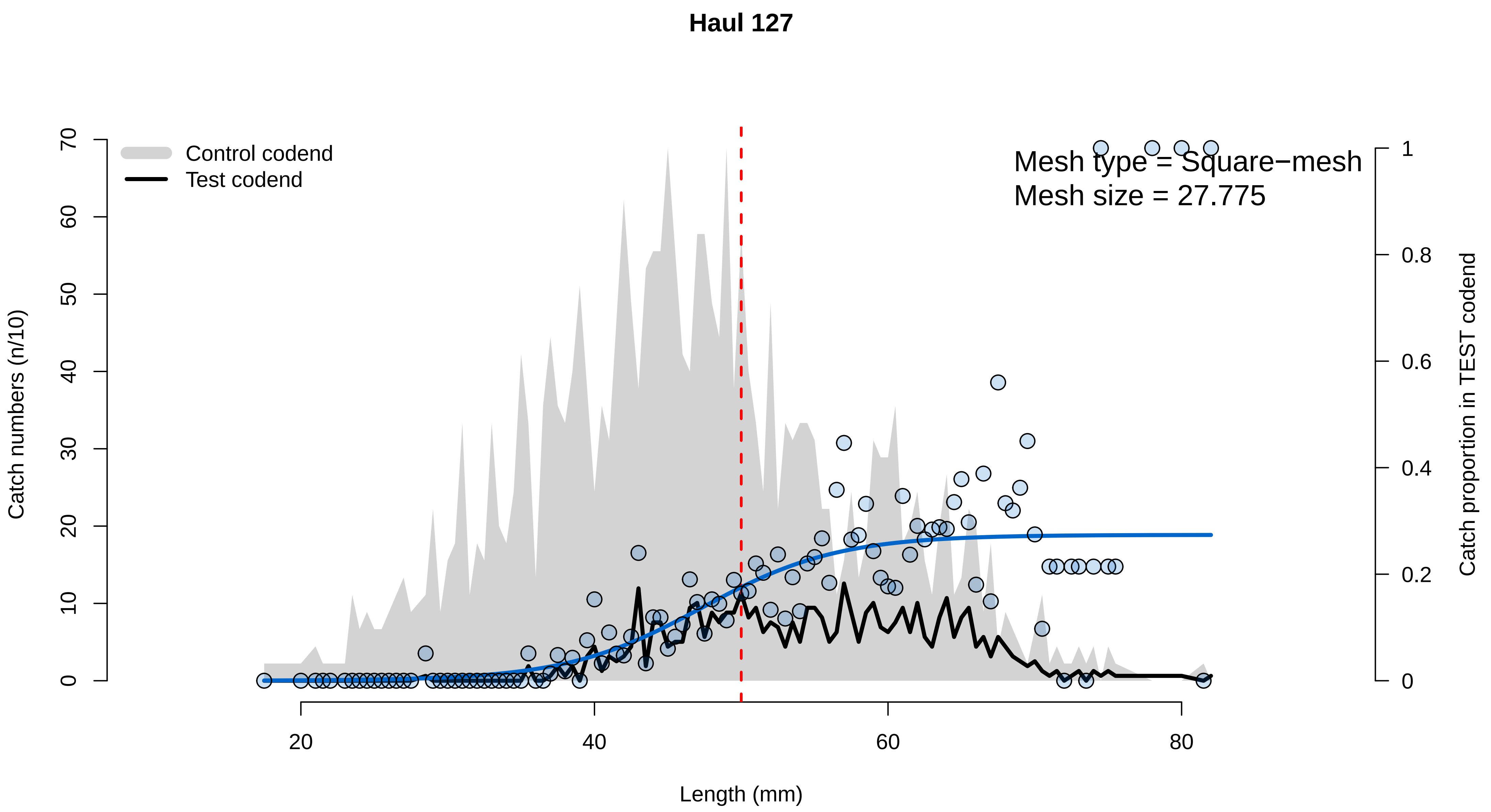

Haul 127
Control codend
Test codend
Mesh type = Square−mesh
Mesh size = 27.775
Catch numbers (n/10)
0
10
20
30
40
50
60
70
Catch proportion in TEST codend
0
0.2
0.4
0.6
0.8
1
20
40
60
80
Length (mm)

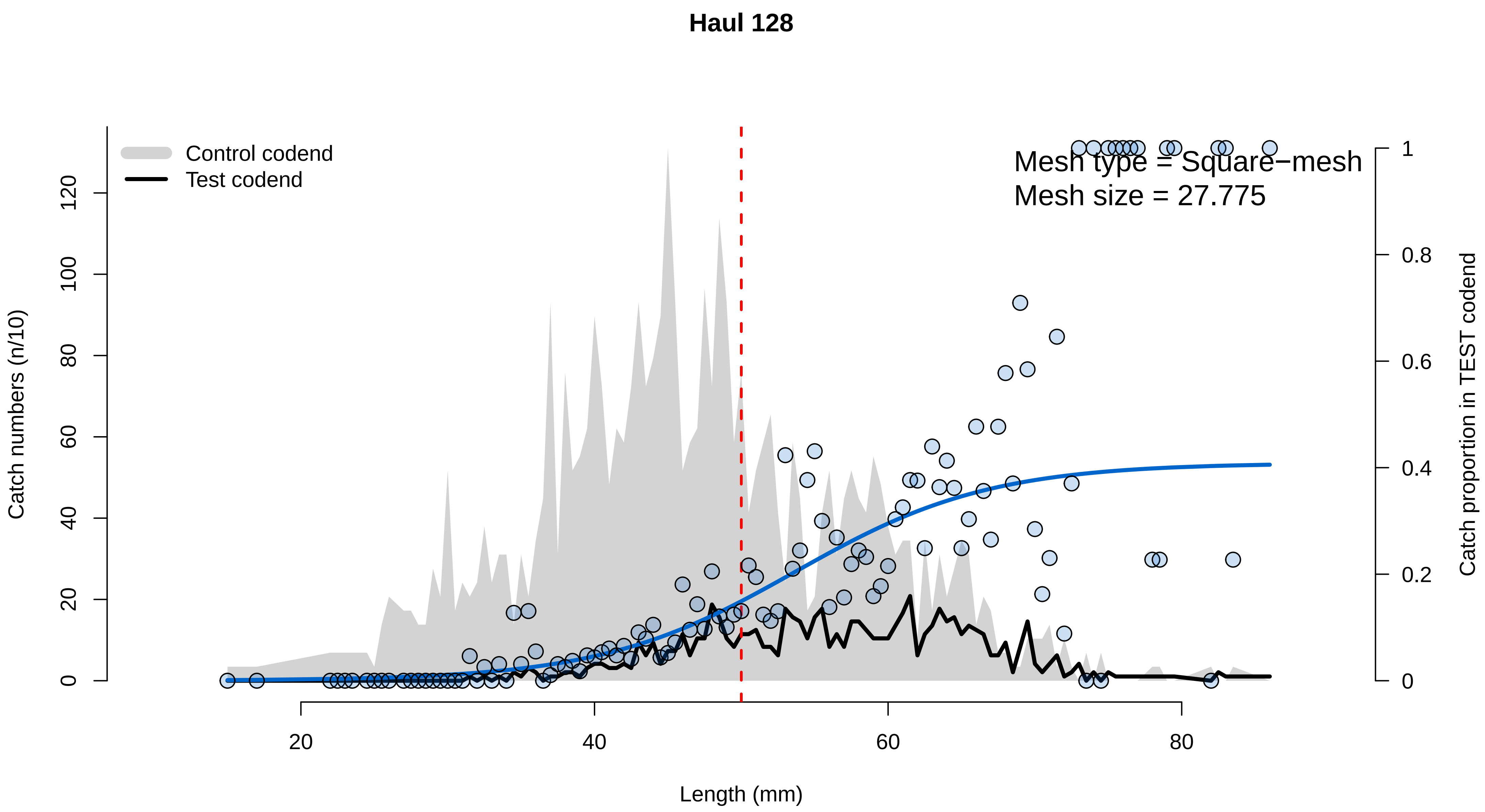
Haul 128
Control codend
Test codend
Mesh type = Square−mesh
Mesh size = 27.775
Catch numbers (n/10)
0
20
40
60
80
100
120
20
40
60
80
Length (mm)
Catch proportion in TEST codend
0
0.2
0.4
0.6
0.8
1

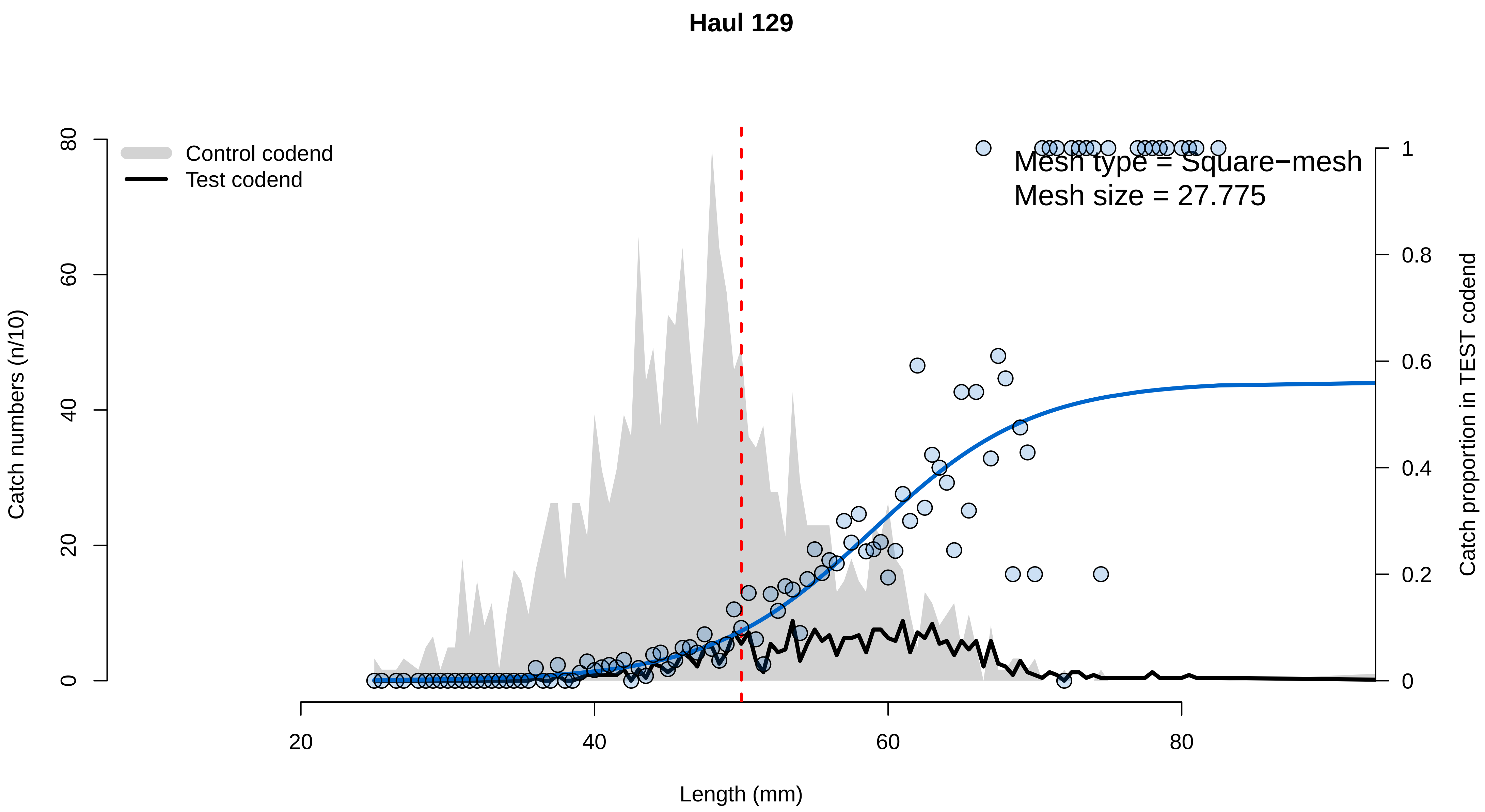

Haul 129
Control codend
Test codend
Mesh type = Square−mesh
Mesh size = 27.775
Catch numbers (n/10)
0
20
40
60
80
Catch proportion in TEST codend
0
0.2
0.4
0.6
0.8
1
20
40
60
80
Length (mm)

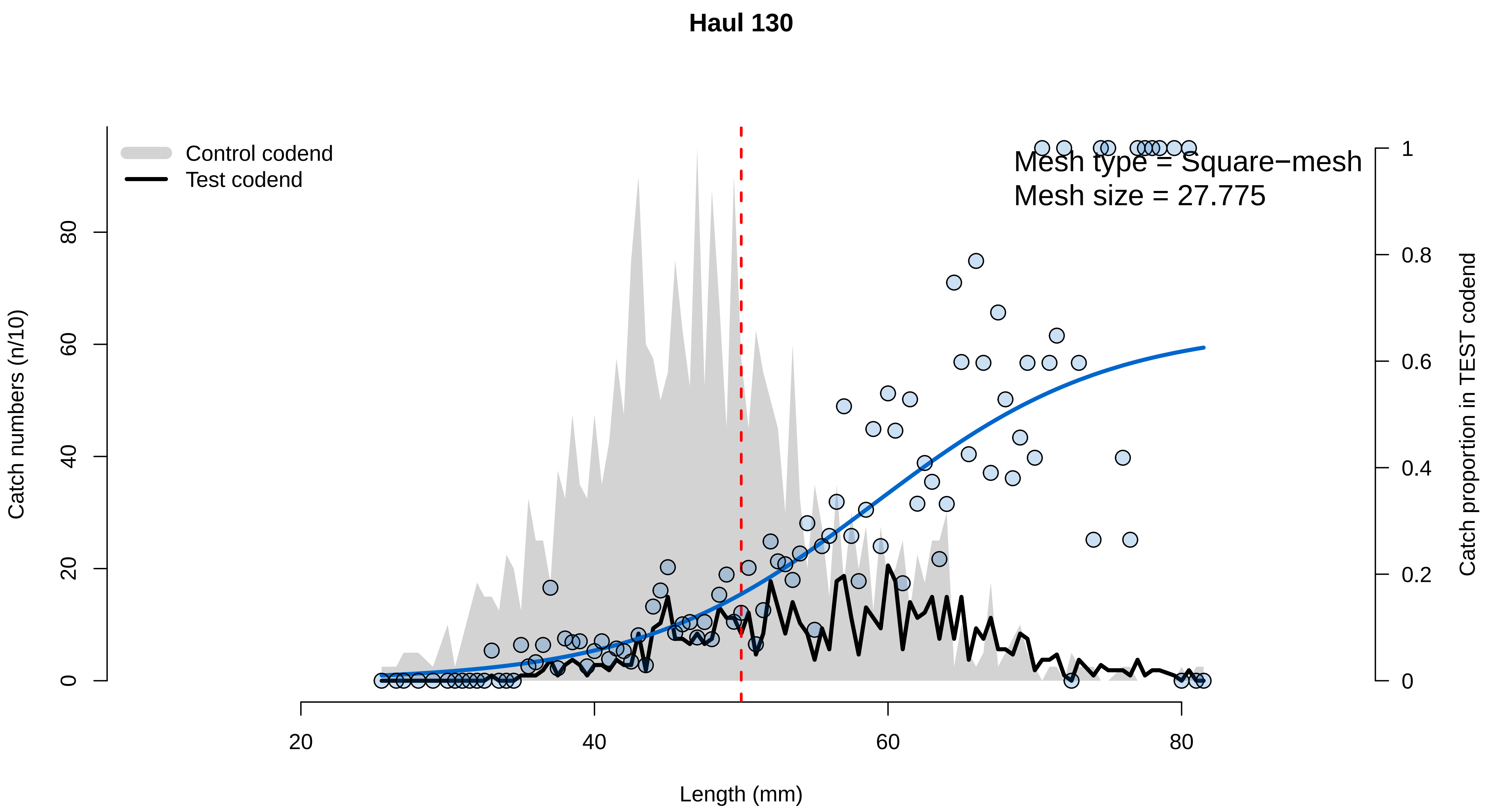

Haul 130
Control codend
Test codend
Mesh type = Square−mesh
Mesh size = 27.775
Catch numbers (n/10)
0
20
40
60
80
Catch proportion in TEST codend
0
0.2
0.4
0.6
0.8
1
20
40
60
80
Length (mm)

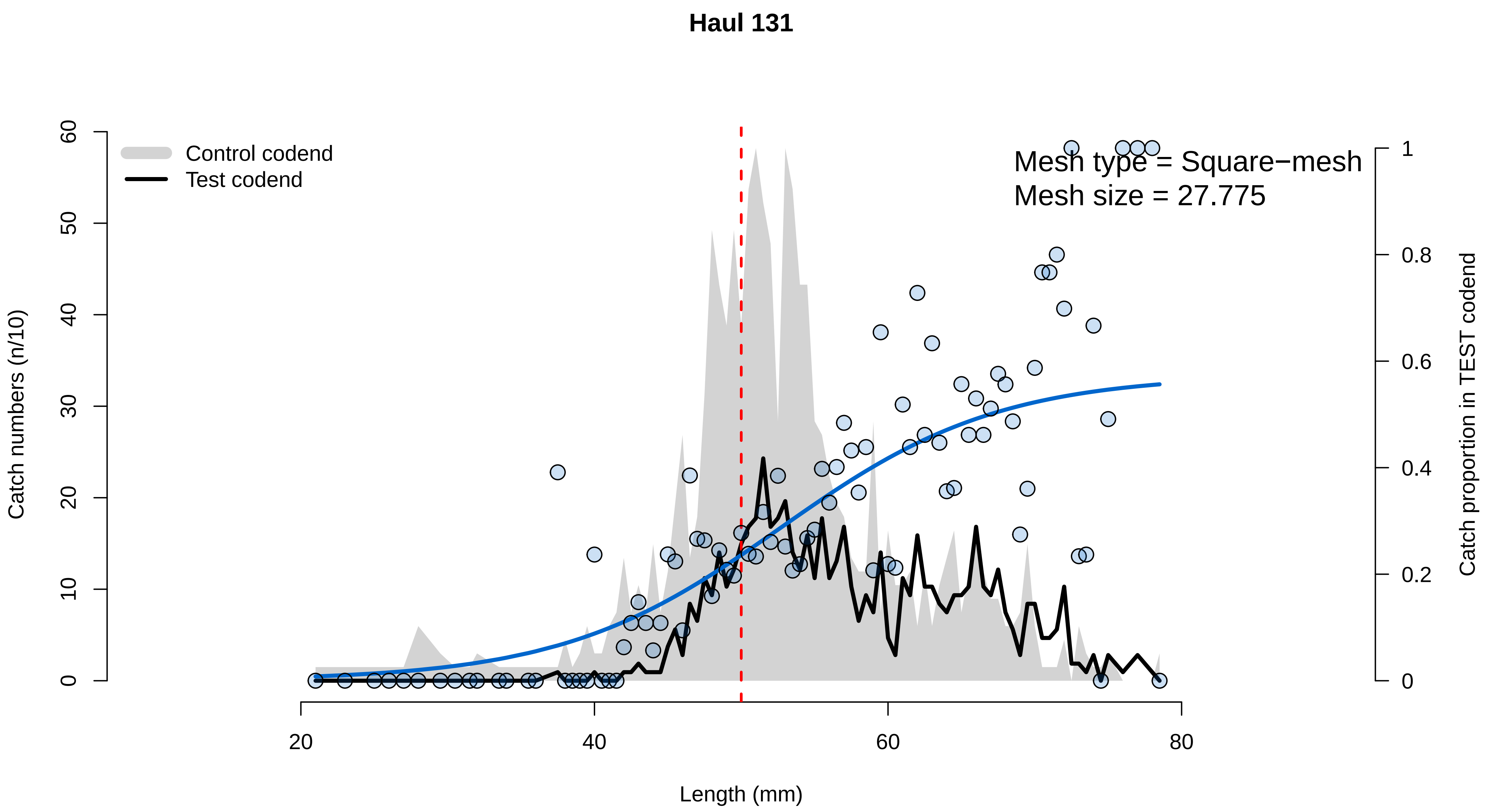
Haul 131
Control codend
Test codend
Mesh type = Square−mesh
Mesh size = 27.775
Catch numbers (n/10)
Catch proportion in TEST codend
Length (mm)
0
10
20
30
40
50
60
0.2
0.4
0.6
0.8
1
20
40
60
80

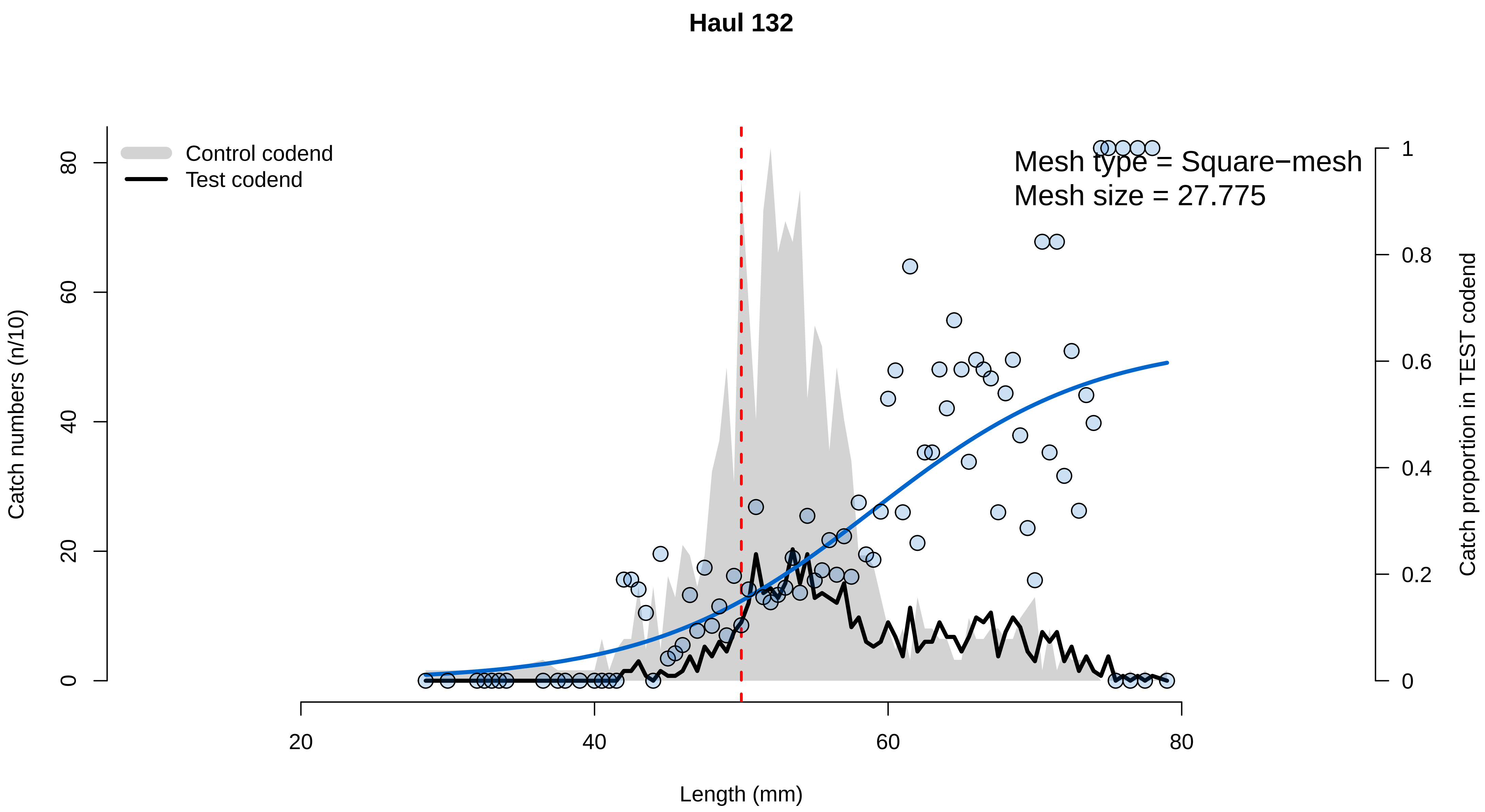

Haul 132
Control codend
Test codend
Mesh type = Square−mesh
Mesh size = 27.775
Catch numbers (n/10)
Catch proportion in TEST codend
Length (mm)
0
20
40
60
80
0
0.2
0.4
0.6
0.8
1
20
40
60
80

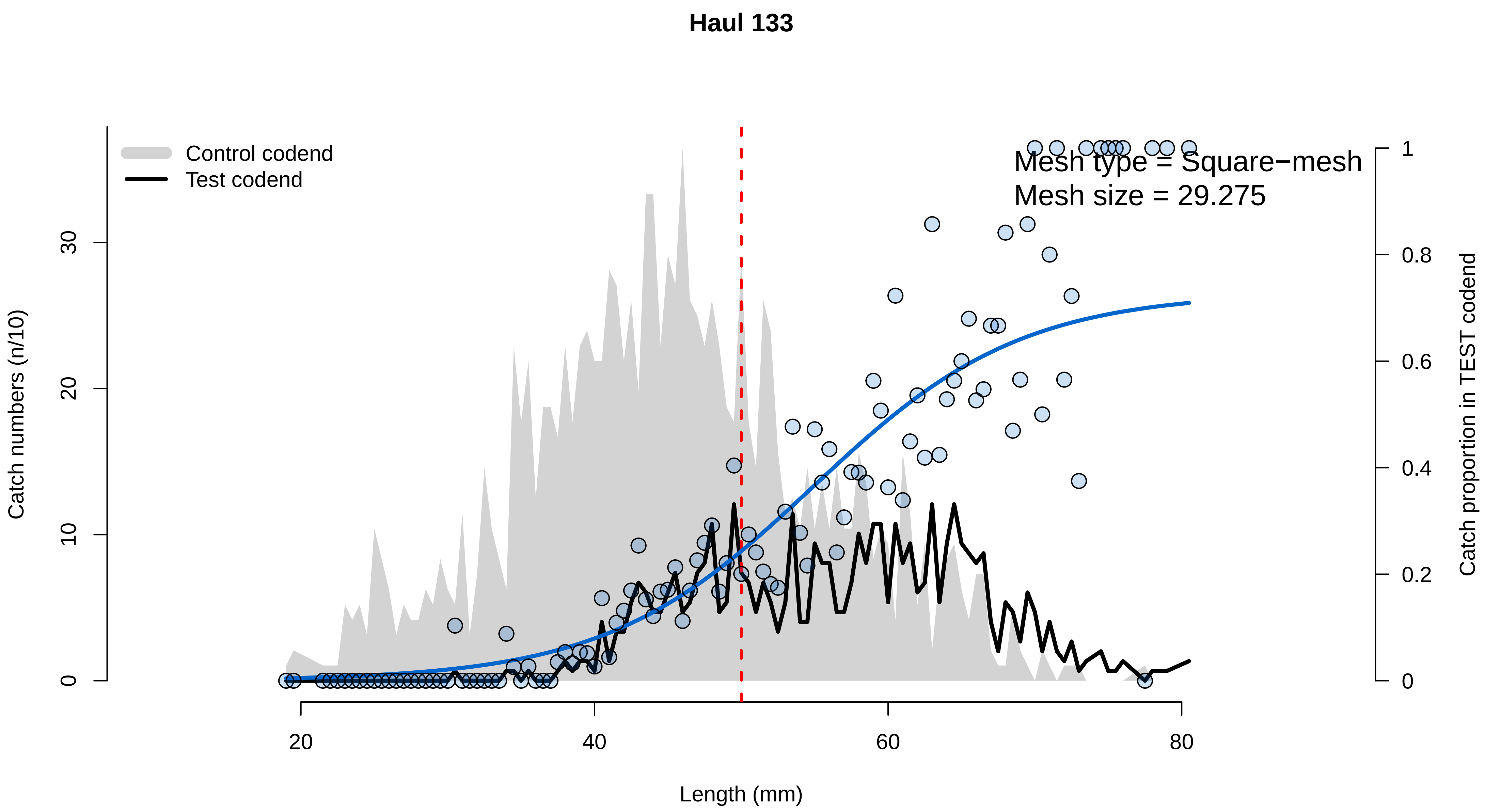

Haul 133
Control codend
Test codend
Mesh type = Square−mesh
Mesh size = 29.275
Catch numbers (n/10)
Catch proportion in TEST codend
Length (mm)
0
10
20
30
20
40
60
80
0
0.2
0.4
0.6
0.8
1

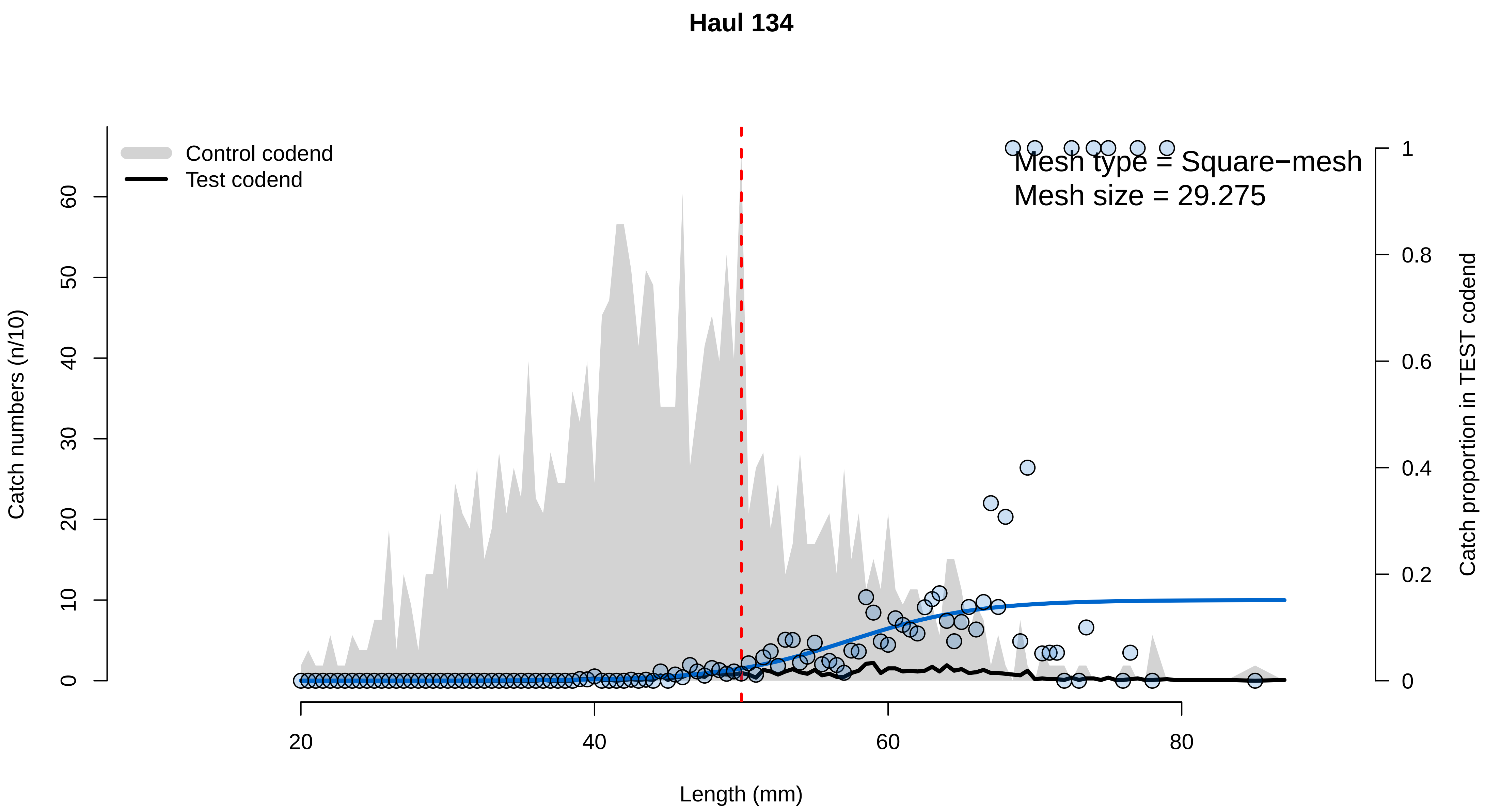

Haul 134
Control codend
Test codend
Mesh type = Square−mesh
Mesh size = 29.275
Catch numbers (n/10)
0
10
20
30
40
50
60
Catch proportion in TEST codend
0
0.2
0.4
0.6
0.8
1
20
40
60
80
Length (mm)

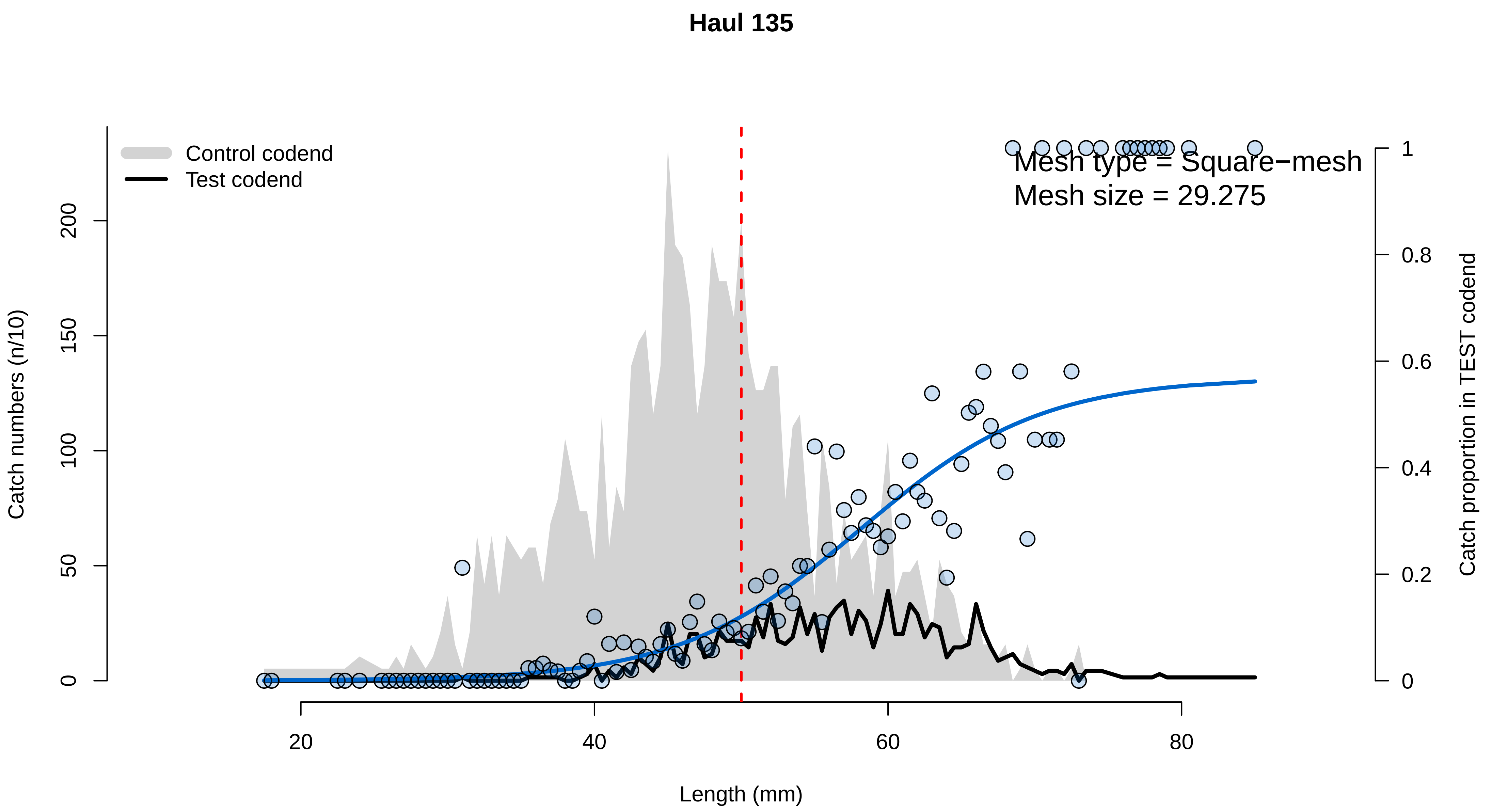
Haul 135
Control codend
Test codend
Mesh type = Square−mesh
Mesh size = 29.275
Catch numbers (n/10)
0
50
100
150
200
Catch proportion in TEST codend
0
0.2
0.4
0.6
0.8
1
20
40
60
80
Length (mm)

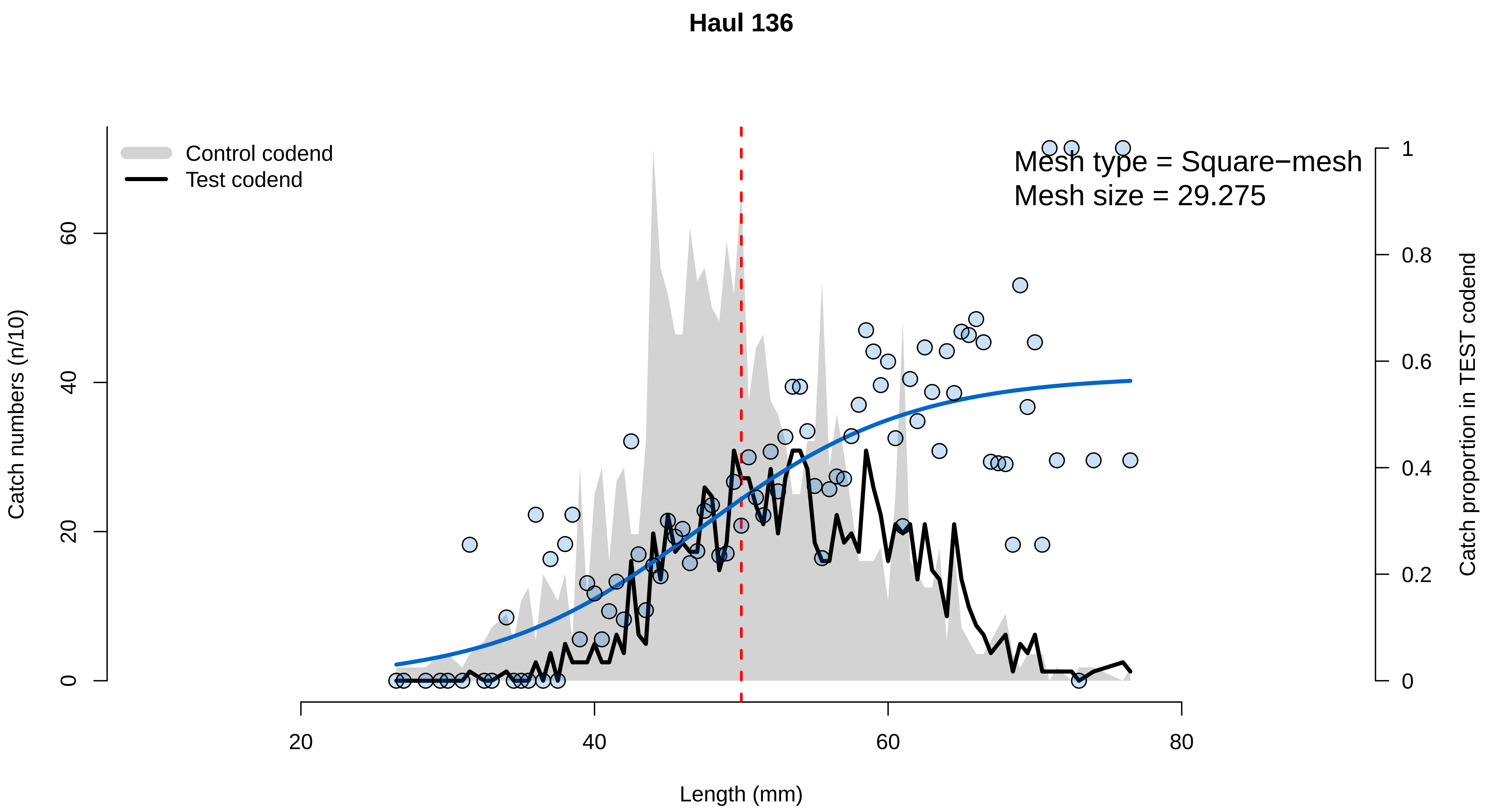

Haul 136
Control codend
Test codend
Mesh type = Square−mesh
Mesh size = 29.275
Catch numbers (n/10)
0
20
40
60
Catch proportion in TEST codend
0
0.2
0.4
0.6
0.8
1
20
40
60
80
Length (mm)

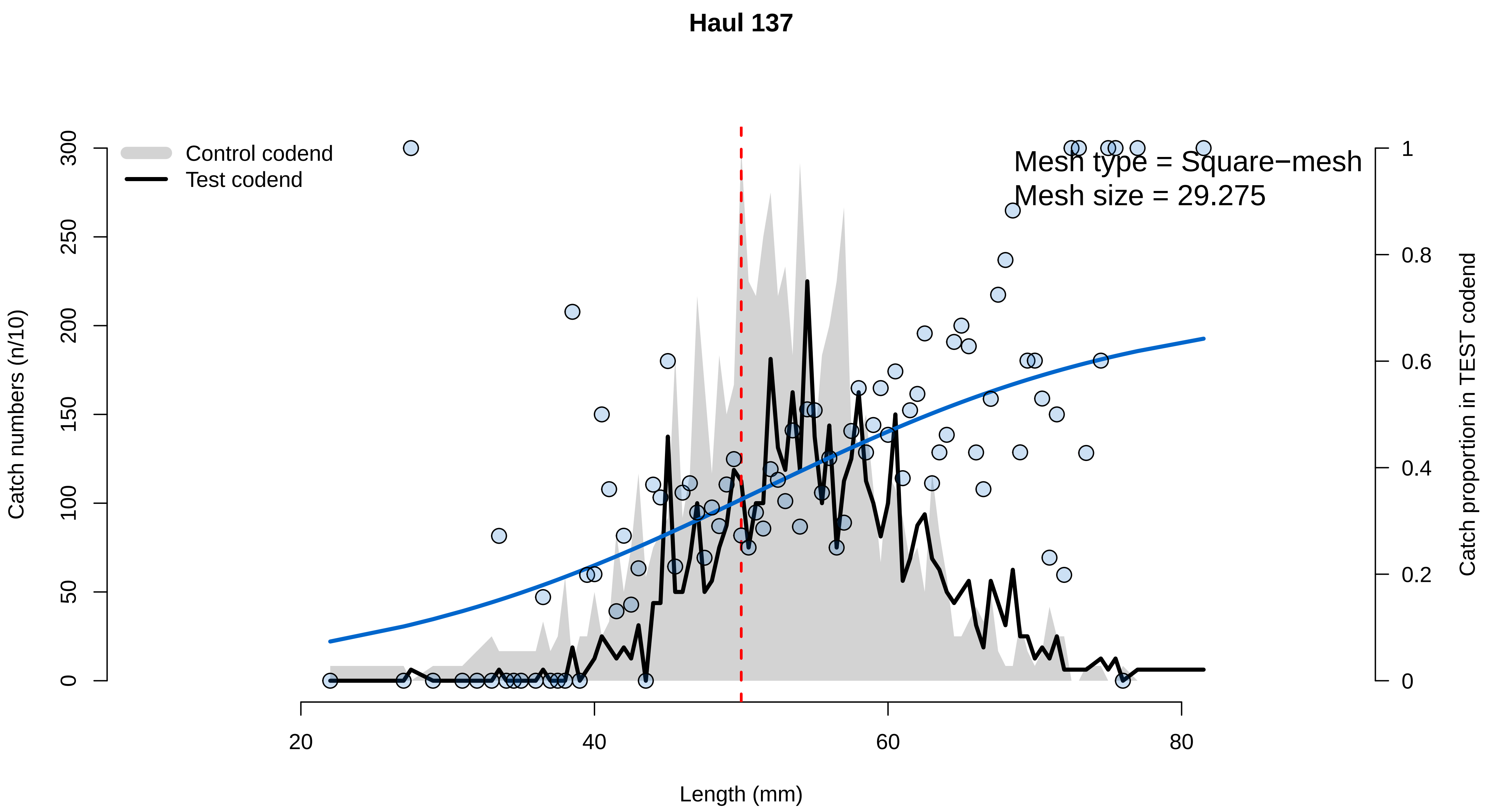

Haul 137
Control codend
Test codend
Mesh type = Square−mesh
Mesh size = 29.275
Catch numbers (n/10)
0
50
100
150
200
250
300
Length (mm)
20
40
60
80
Catch proportion in TEST codend
0
0.2
0.4
0.6
0.8
1

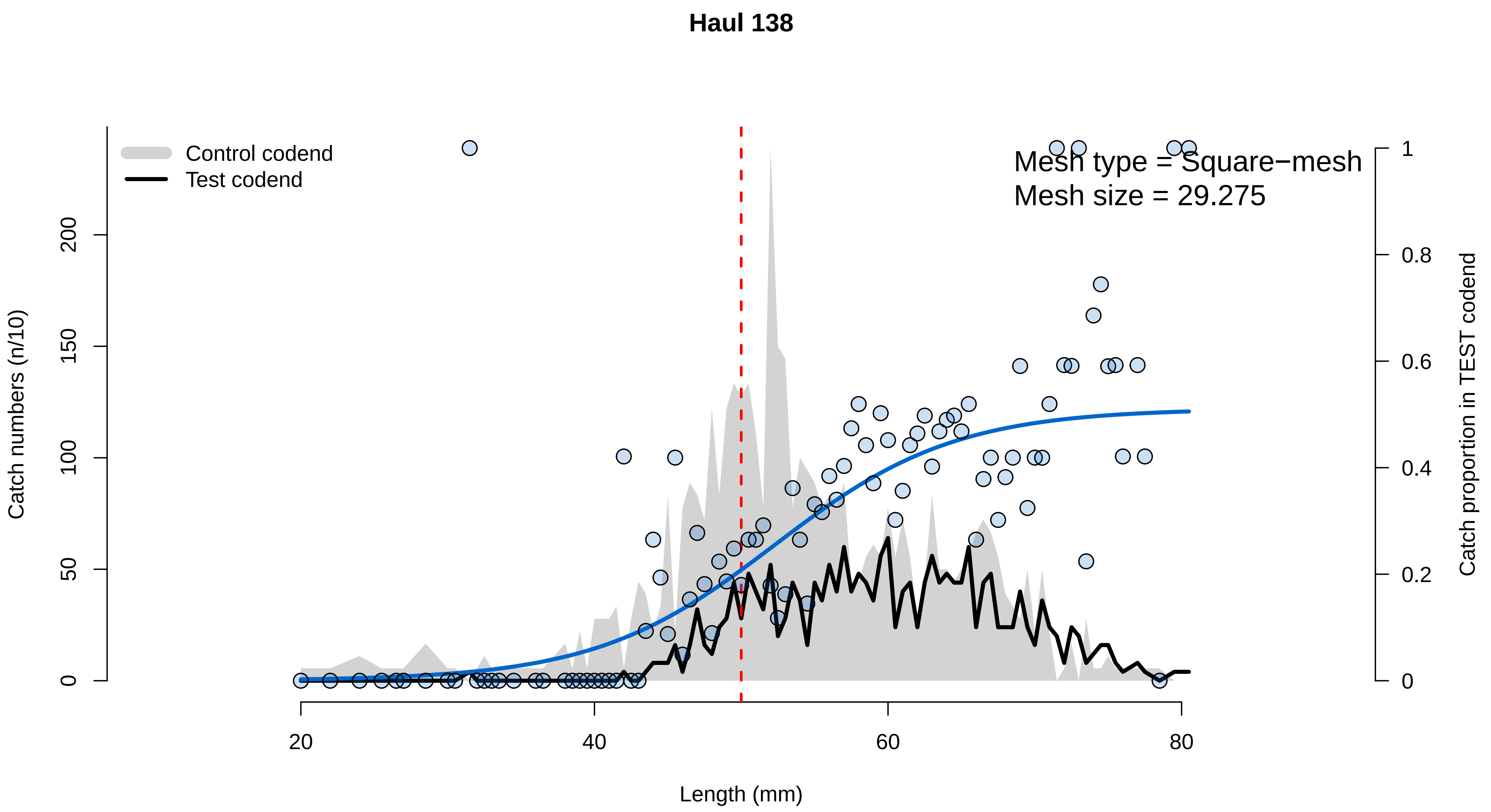
Haul 138
Control codend
Test codend
Mesh type = Square−mesh
Mesh size = 29.275
Catch numbers (n/10)
0
50
100
150
200
Catch proportion in TEST codend
0
0.2
0.4
0.6
0.8
1
20
40
60
80
Length (mm)

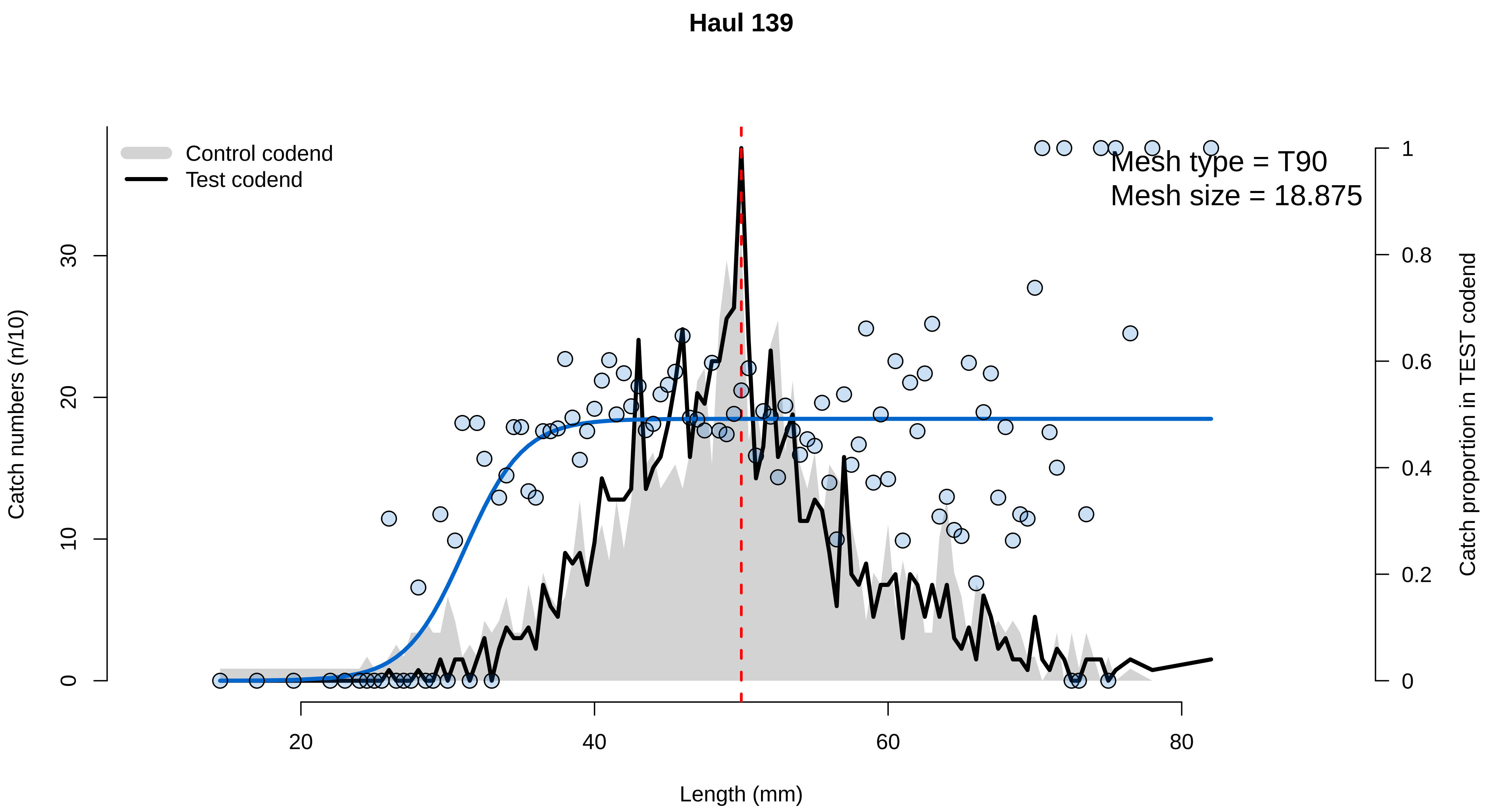

Haul 139
Control codend
Test codend
Mesh type = T90
Mesh size = 18.875
Catch numbers (n/10)
0
10
20
30
20
40
60
80
Length (mm)
0
0.2
0.4
0.6
0.8
1
Catch proportion in TEST codend

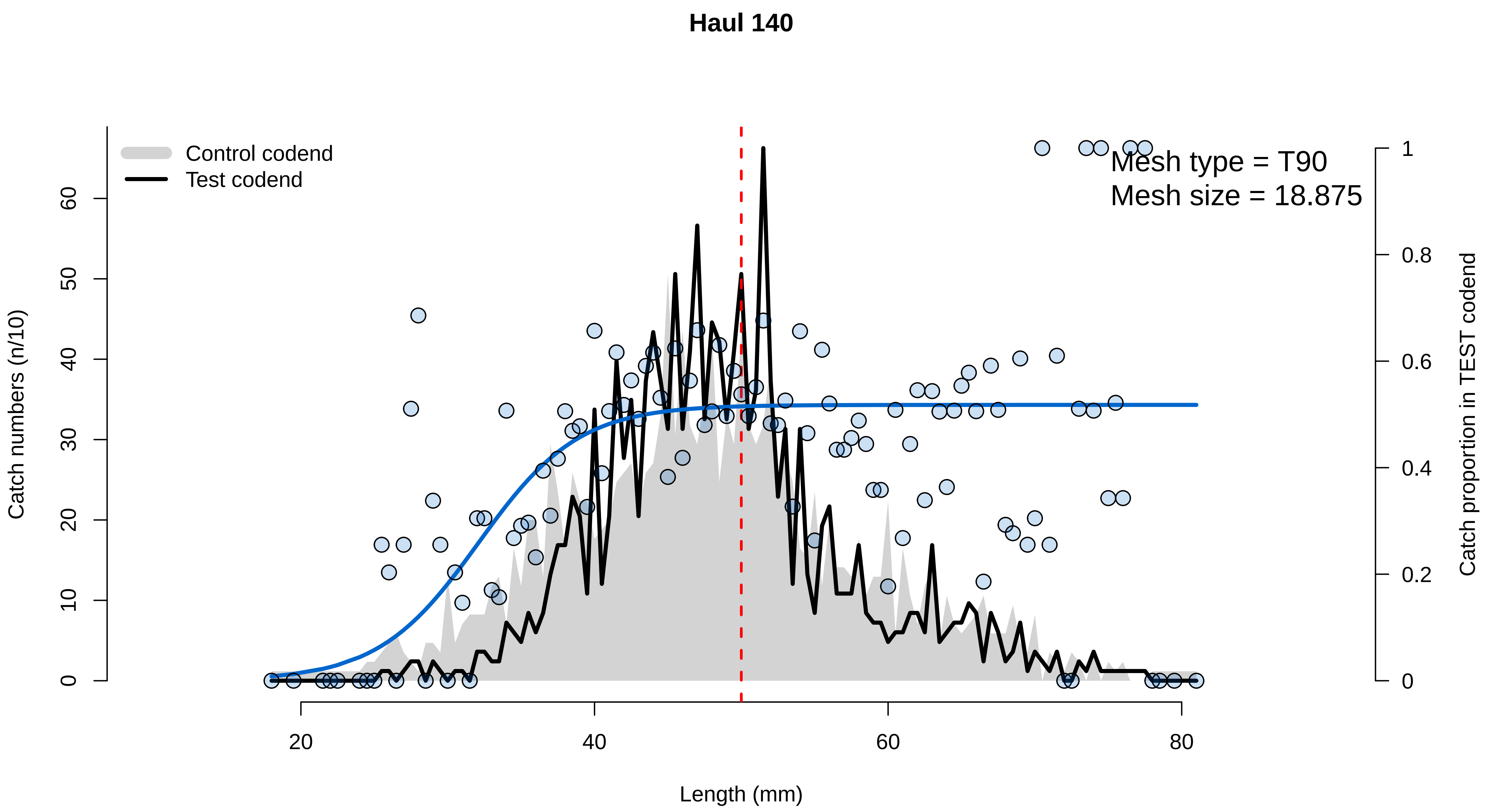

Haul 140
Control codend
Test codend
Mesh type = T90
Mesh size = 18.875
Catch numbers (n/10)
0
10
20
30
40
50
60
Catch proportion in TEST codend
0
0.2
0.4
0.6
0.8
1
20
40
60
80
Length (mm)

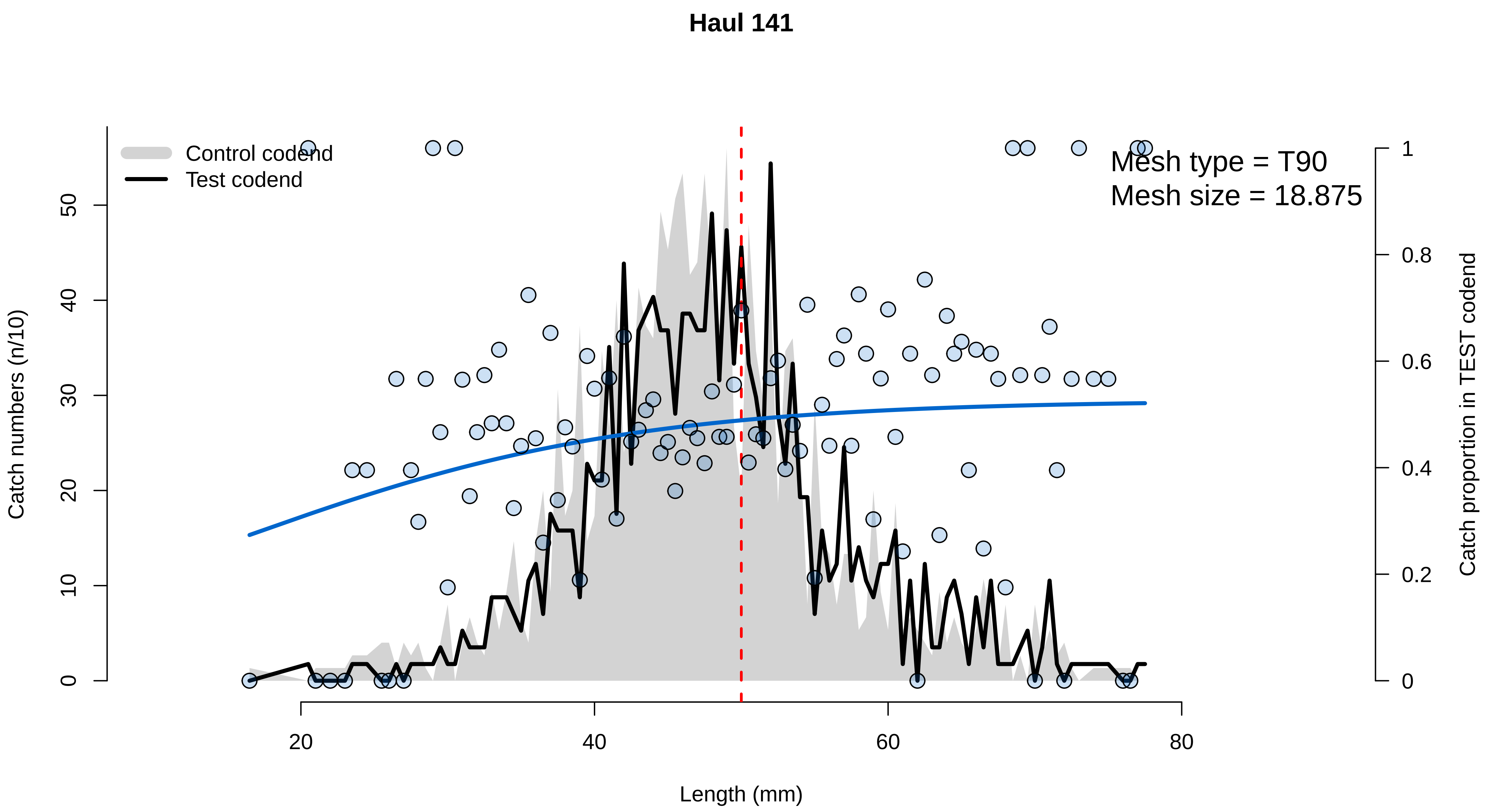
Haul 141
Control codend
Test codend
Mesh type = T90
Mesh size = 18.875
Catch numbers (n/10)
0
10
20
30
40
50
Length (mm)
20
40
60
80
Catch proportion in TEST codend
0
0.2
0.4
0.6
0.8
1

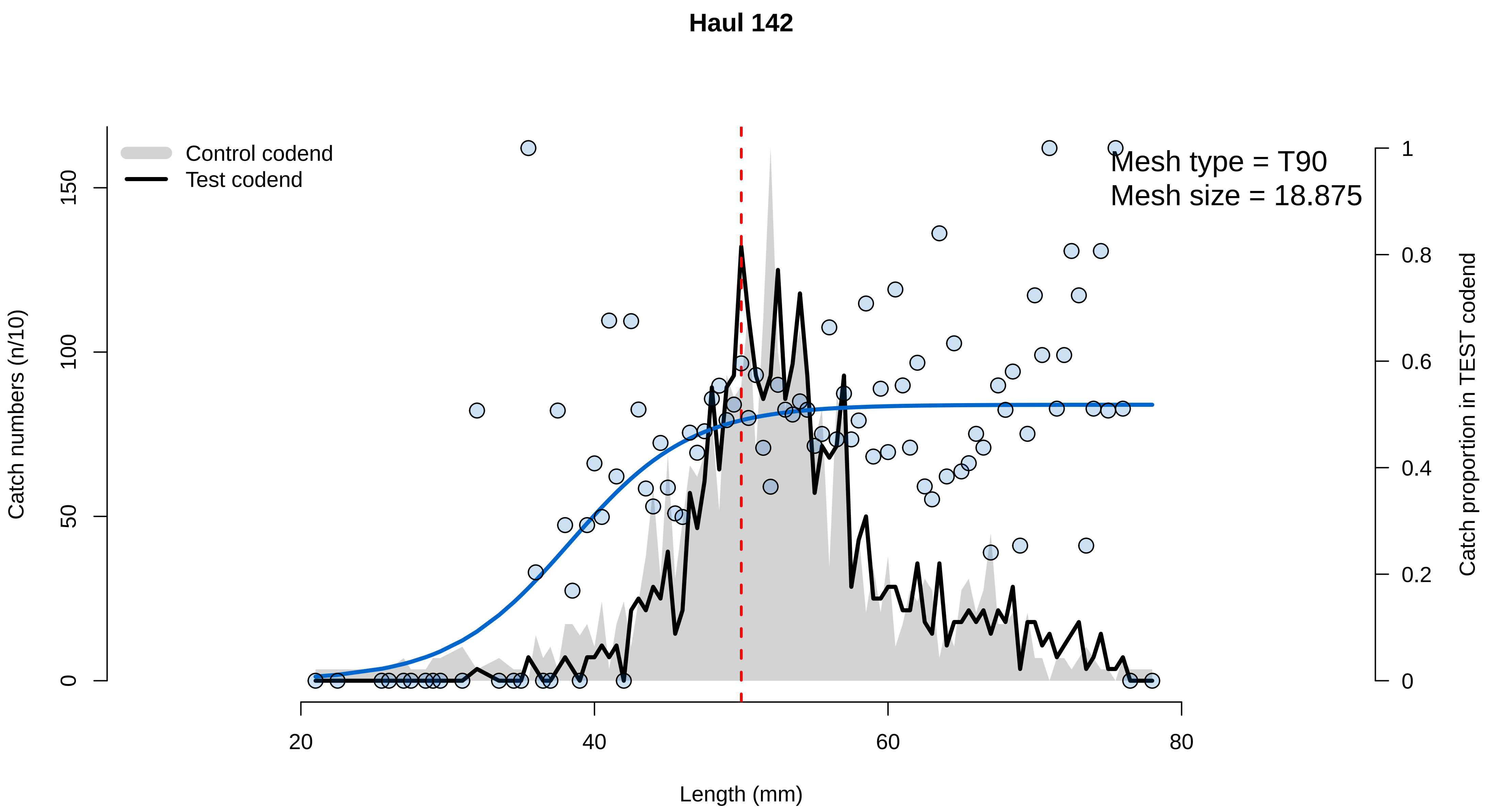

Haul 142
Control codend
Test codend
Mesh type = T90
Mesh size = 18.875
Catch numbers (n/10)
0
50
100
150
Length (mm)
20
40
60
80
Catch proportion in TEST codend
0
0.2
0.4
0.6
0.8
1

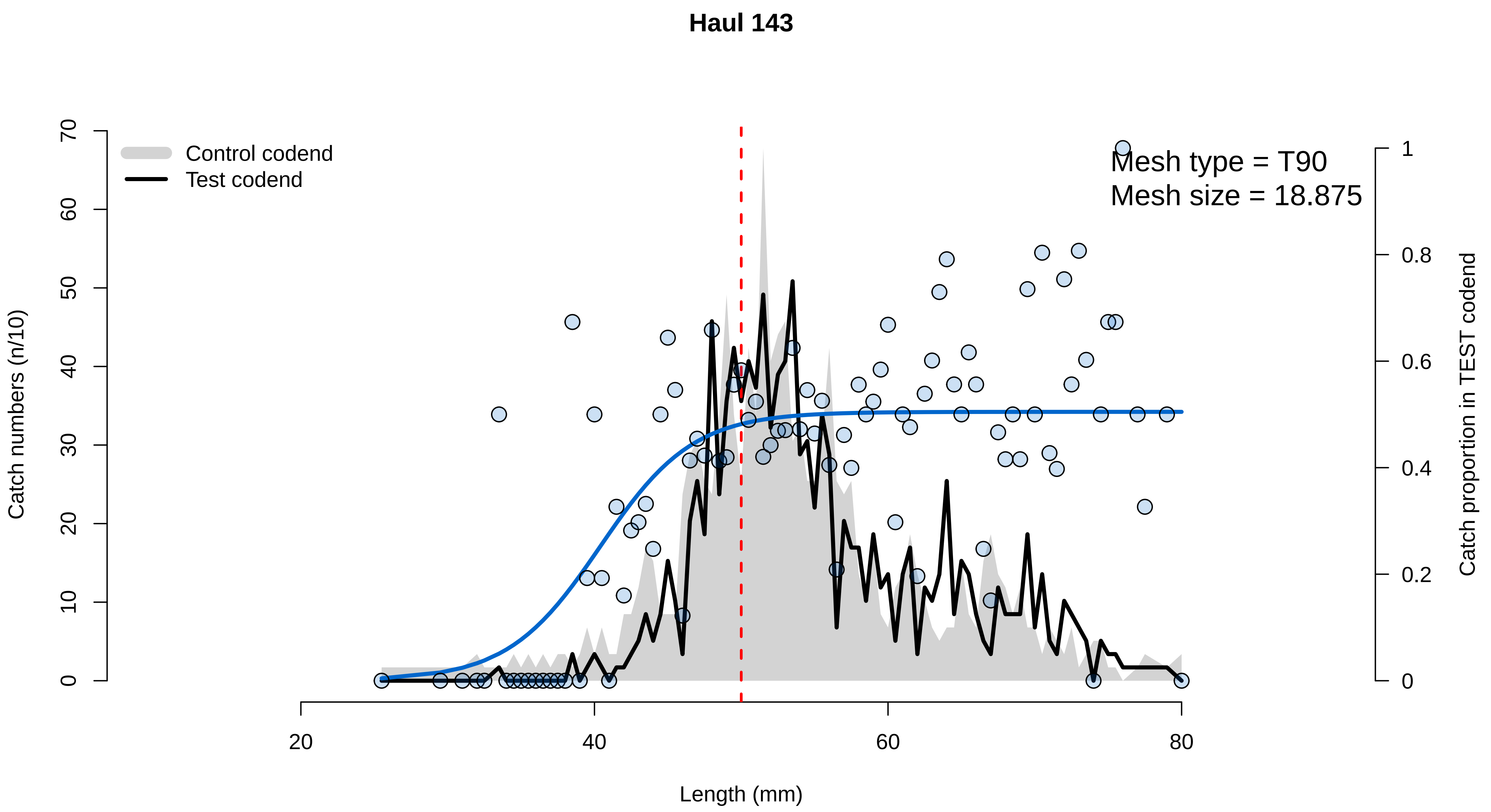
Haul 143
Control codend
Test codend
Mesh type = T90
Mesh size = 18.875
Catch numbers (n/10)
0
10
20
30
40
50
60
70
Catch proportion in TEST codend
0
0.2
0.4
0.6
0.8
1
20
40
60
80
Length (mm)

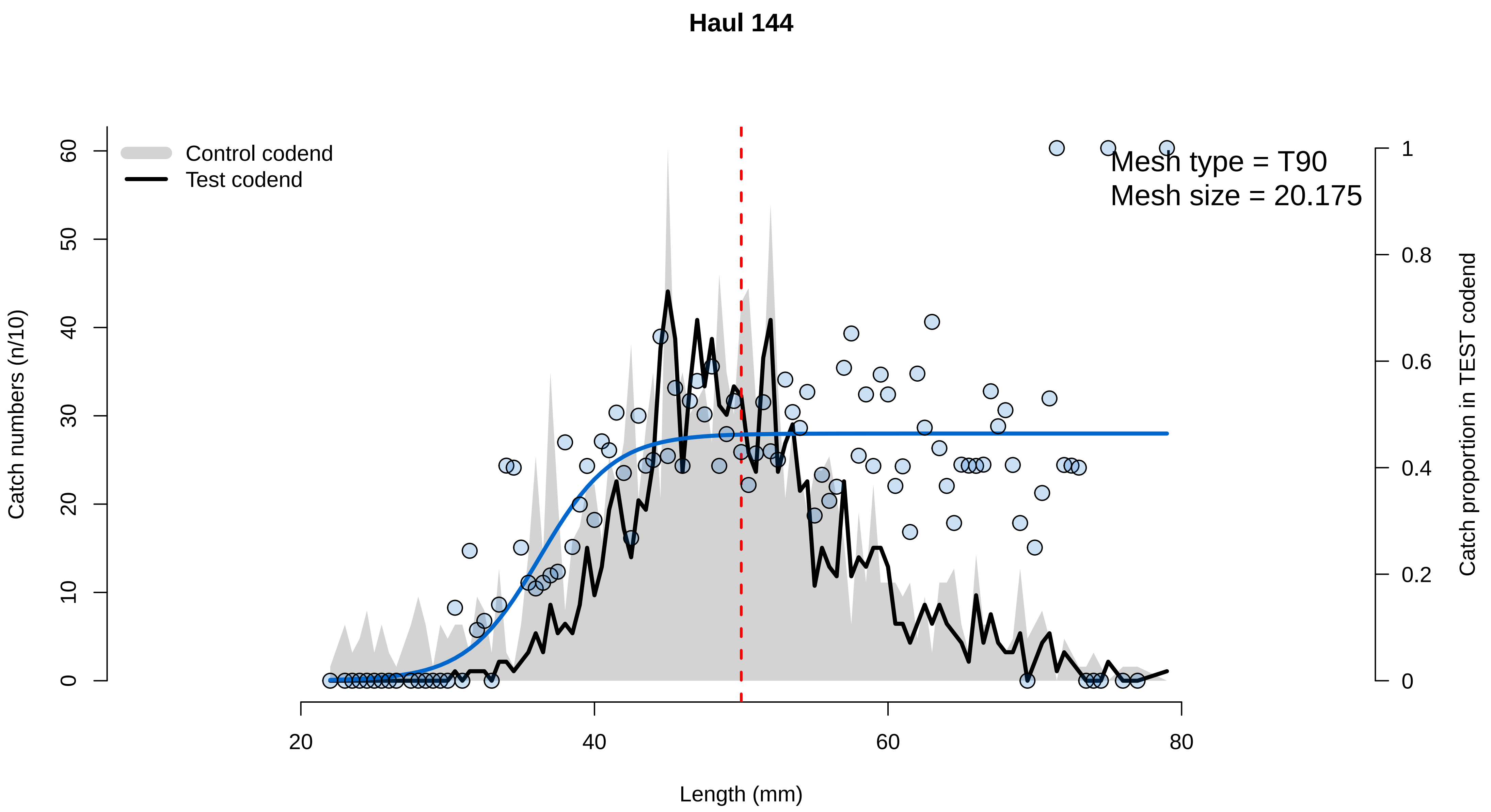

Haul 144
Control codend
Test codend
Mesh type = T90
Mesh size = 20.175
Catch numbers (n/10)
0
10
20
30
40
50
60
Catch proportion in TEST codend
0
0.2
0.4
0.6
0.8
1
20
40
60
80
Length (mm)

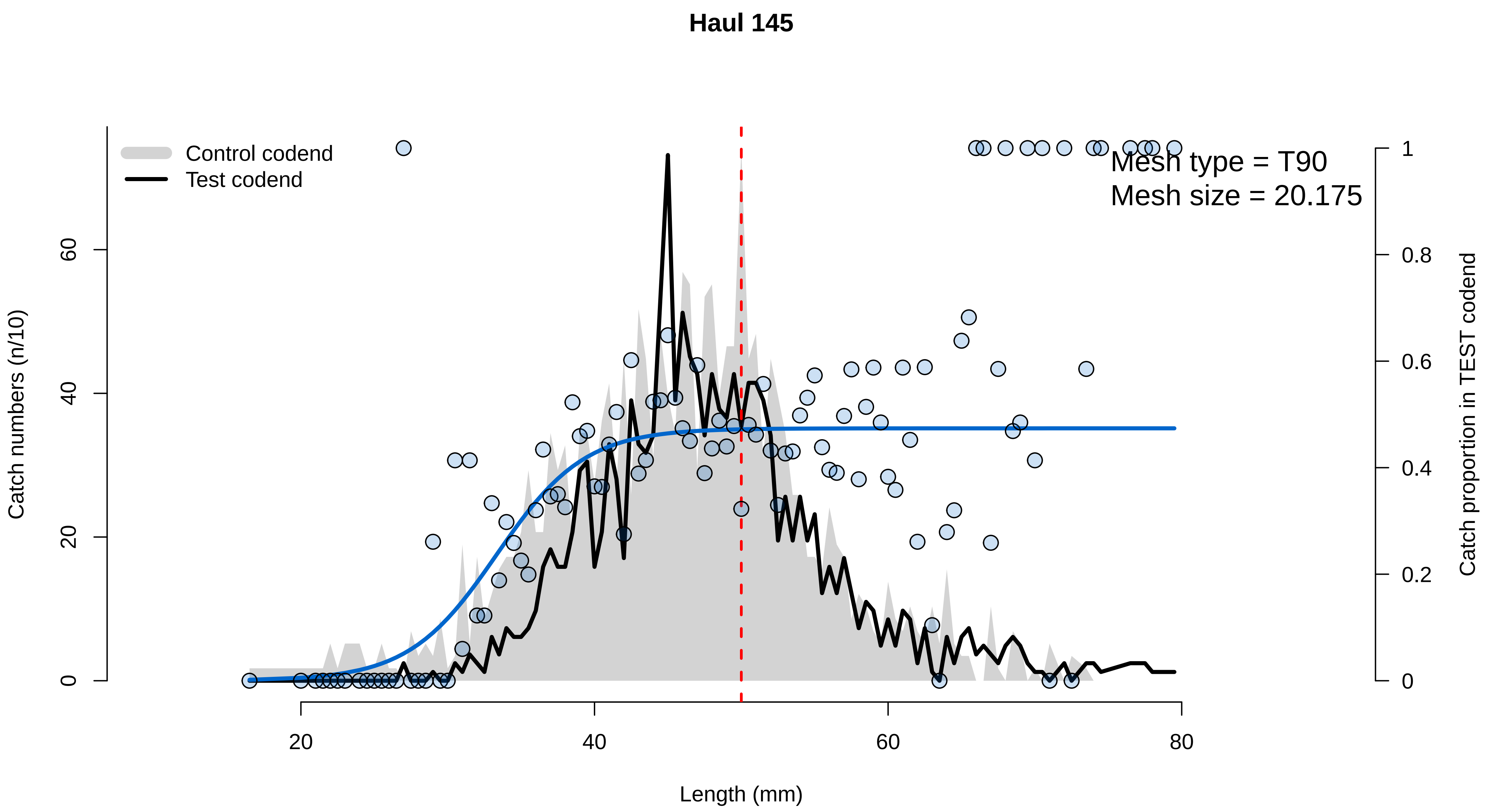

Haul 145
Control codend
Test codend
Mesh type = T90
Mesh size = 20.175
Catch numbers (n/10)
0
20
40
60
Length (mm)
20
40
60
80
Catch proportion in TEST codend
0
0.2
0.4
0.6
0.8
1

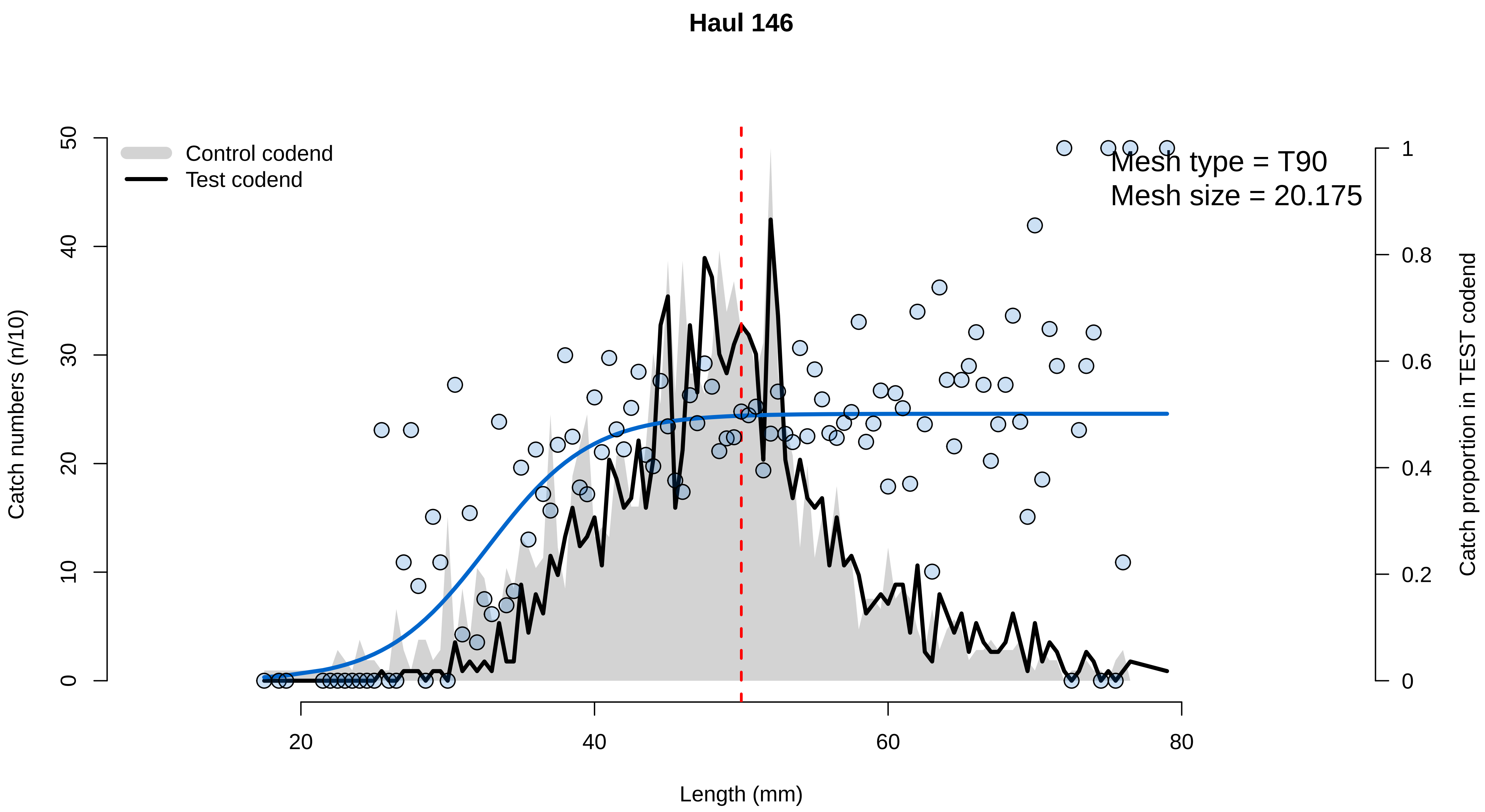

Haul 146
Control codend
Test codend
Mesh type = T90
Mesh size = 20.175
Catch numbers (n/10)
0
10
20
30
40
50
Catch proportion in TEST codend
0
0.2
0.4
0.6
0.8
1
20
40
60
80
Length (mm)

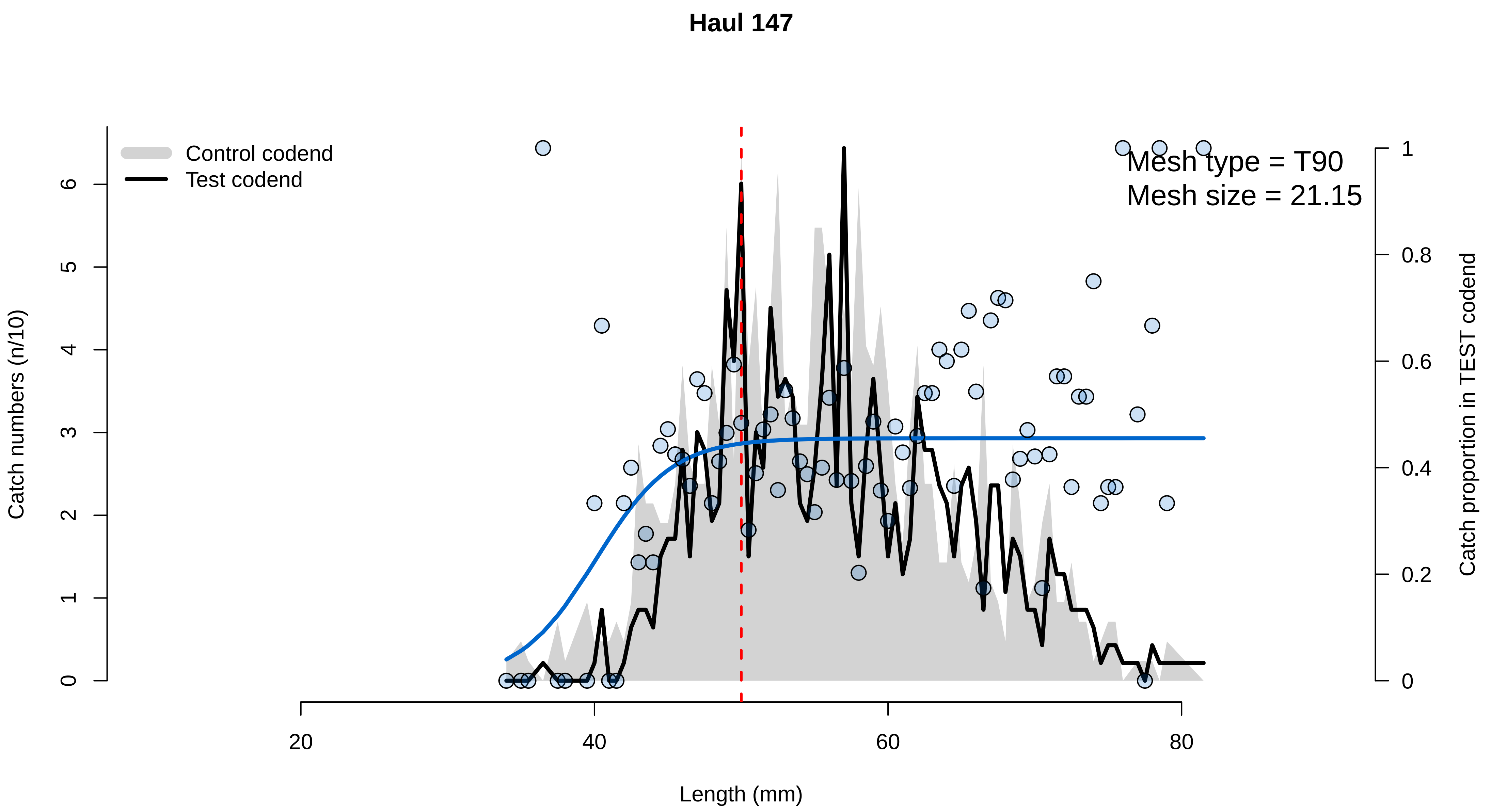
Haul 147
Control codend
Test codend
Mesh type = T90
Mesh size = 21.15
Catch numbers (n/10)
Catch proportion in TEST codend
Length (mm)
0
1
2
3
4
5
6
0
0.2
0.4
0.6
0.8
1
20
40
60
80

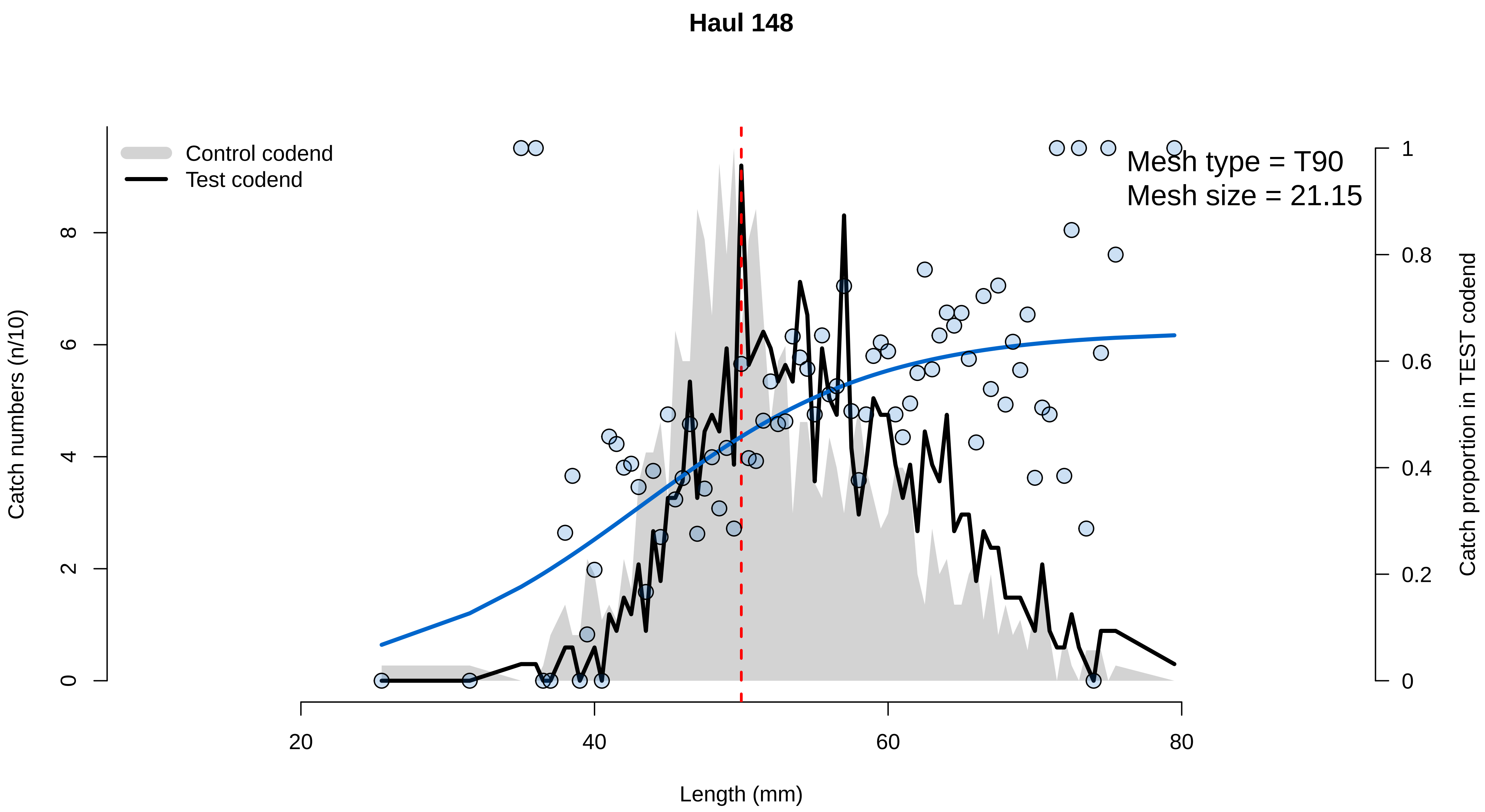

Haul 148
Control codend
Test codend
Mesh type = T90
Mesh size = 21.15
Catch numbers (n/10)
0
2
4
6
8
Catch proportion in TEST codend
0
0.2
0.4
0.6
0.8
1
20
40
60
80
Length (mm)

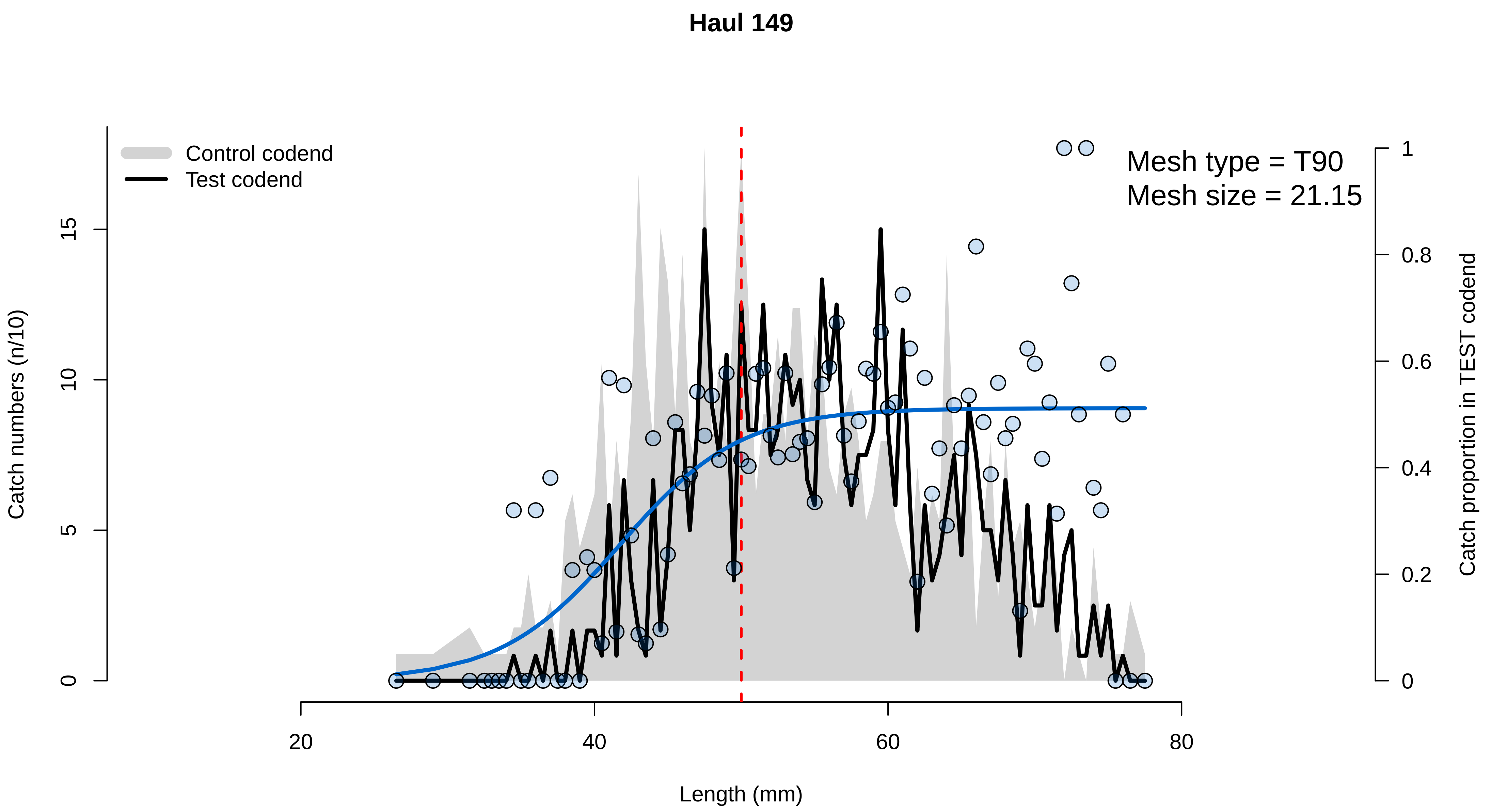
Haul 149
Control codend
Test codend
Mesh type = T90
Mesh size = 21.15
Catch numbers (n/10)
0
5
10
15
Length (mm)
20
40
60
80
Catch proportion in TEST codend
0
0.2
0.4
0.6
0.8
1

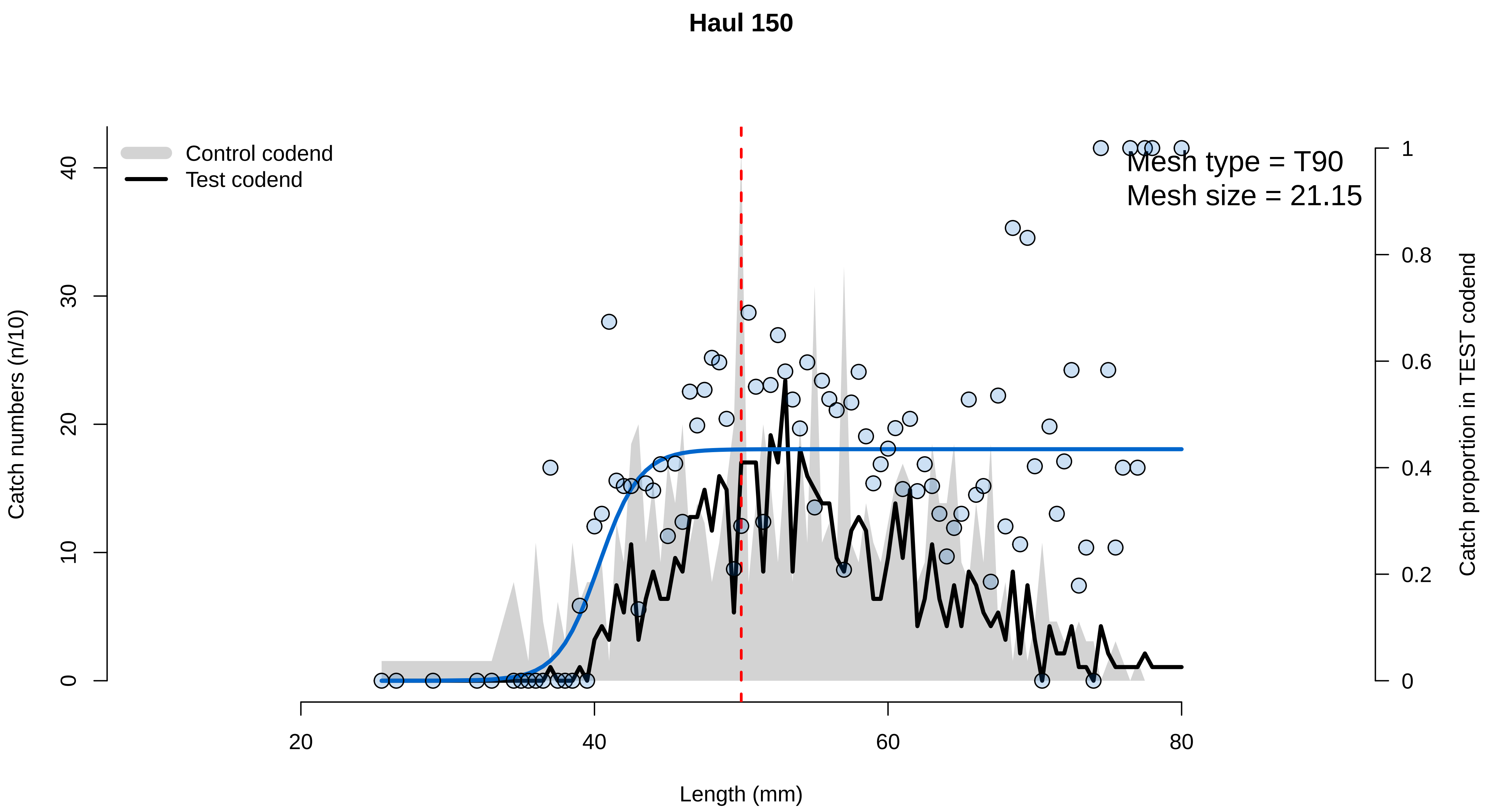

Haul 150
Control codend
Test codend
Mesh type = T90
Mesh size = 21.15
Catch numbers (n/10)
0
10
20
30
40
Catch proportion in TEST codend
0
0.2
0.4
0.6
0.8
1
20
40
60
80
Length (mm)

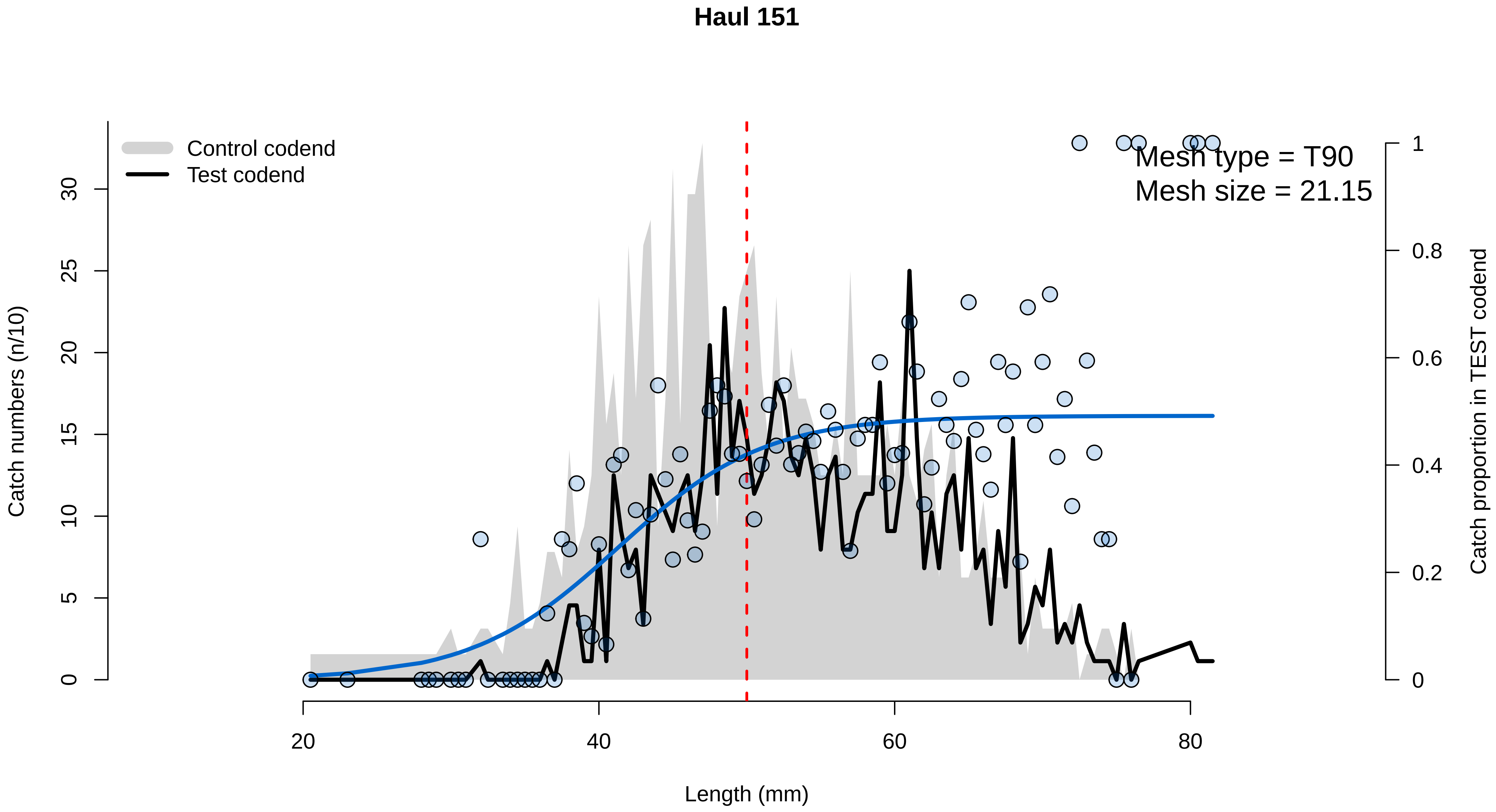
Haul 151
Control codend
Test codend
Mesh type = T90
Mesh size = 21.15
Catch numbers (n/10)
Catch proportion in TEST codend
Length (mm)
0
5
10
15
20
25
30
0
0.2
0.4
0.6
0.8
1
20
40
60
80

Haul 152

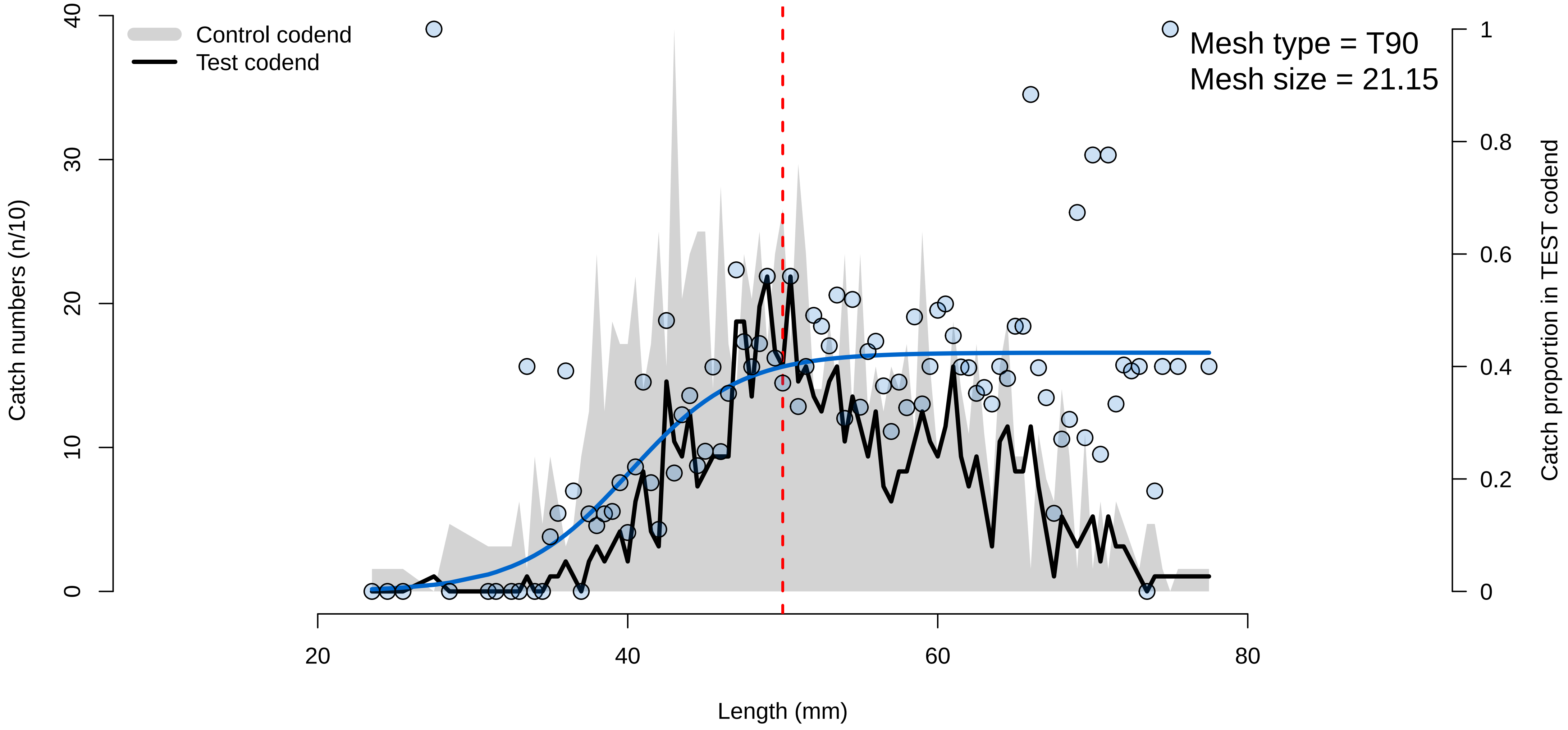

Control codend
Test codend
Mesh type = T90
Mesh size = 21.15
Catch numbers (n/10)
Catch proportion in TEST codend
Length (mm)
0
10
20
30
40
0
0.2
0.4
0.6
0.8
1
20
40
60
80

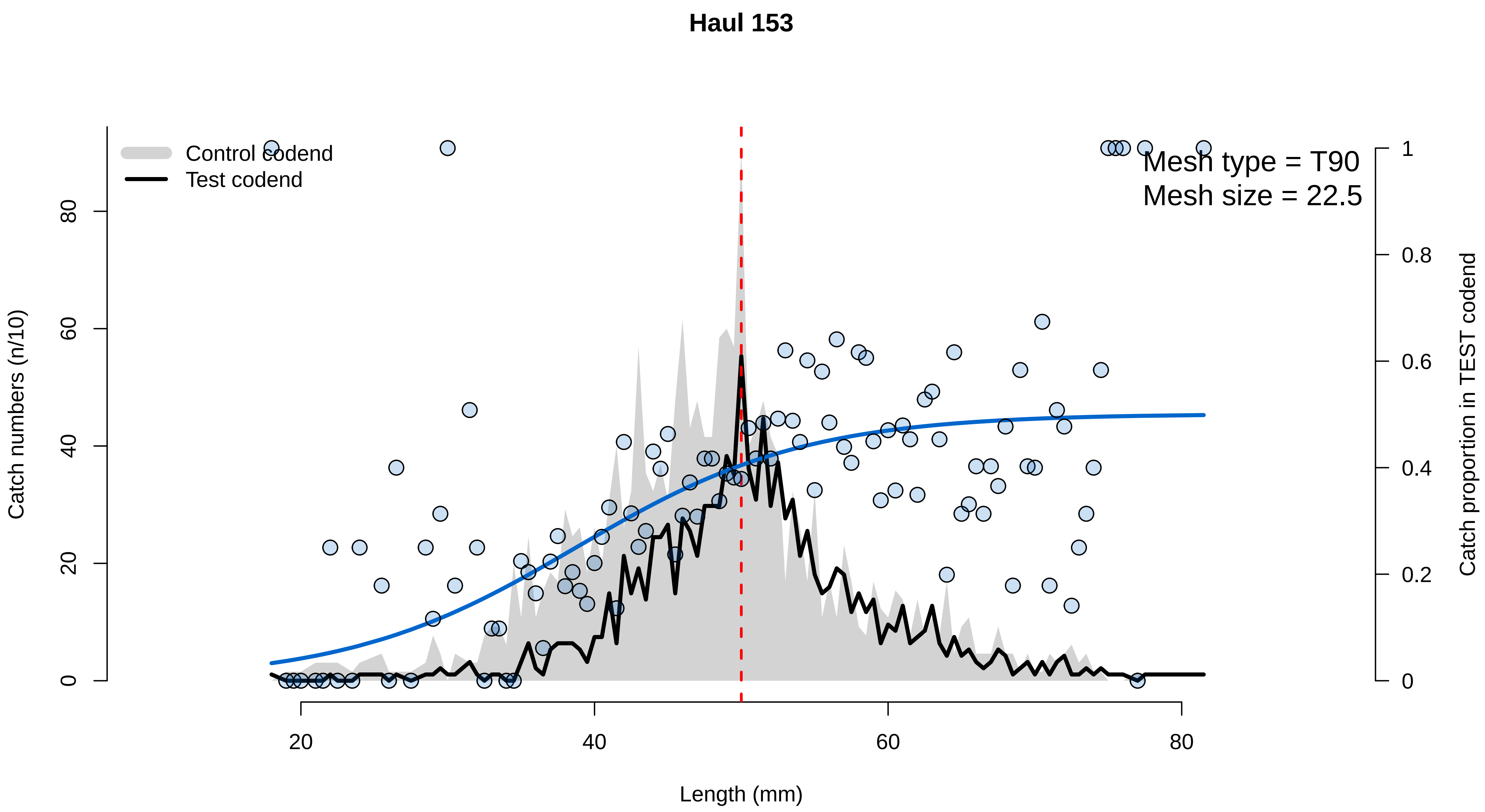
Haul 153
Control codend
Test codend
Mesh type = T90
Mesh size = 22.5
Catch numbers (n/10)
Catch proportion in TEST codend
Length (mm)
0
20
40
60
80
0
0.2
0.4
0.6
0.8
1
20
40
60
80

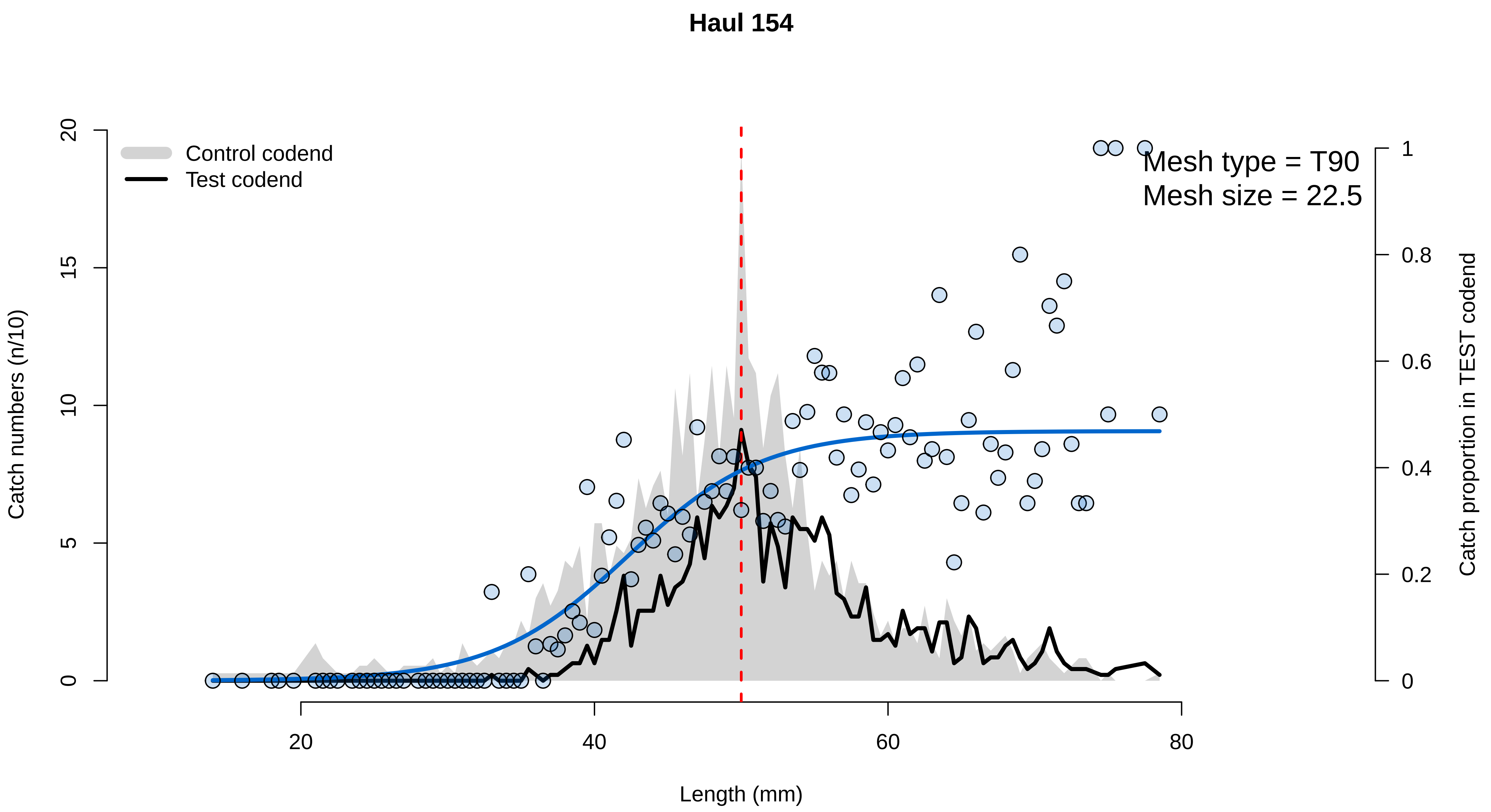

Haul 154
Control codend
Test codend
Mesh type = T90
Mesh size = 22.5
Catch numbers (n/10)
Catch proportion in TEST codend
Length (mm)
0
5
10
15
20
20
40
60
80
0
0.2
0.4
0.6
0.8
1

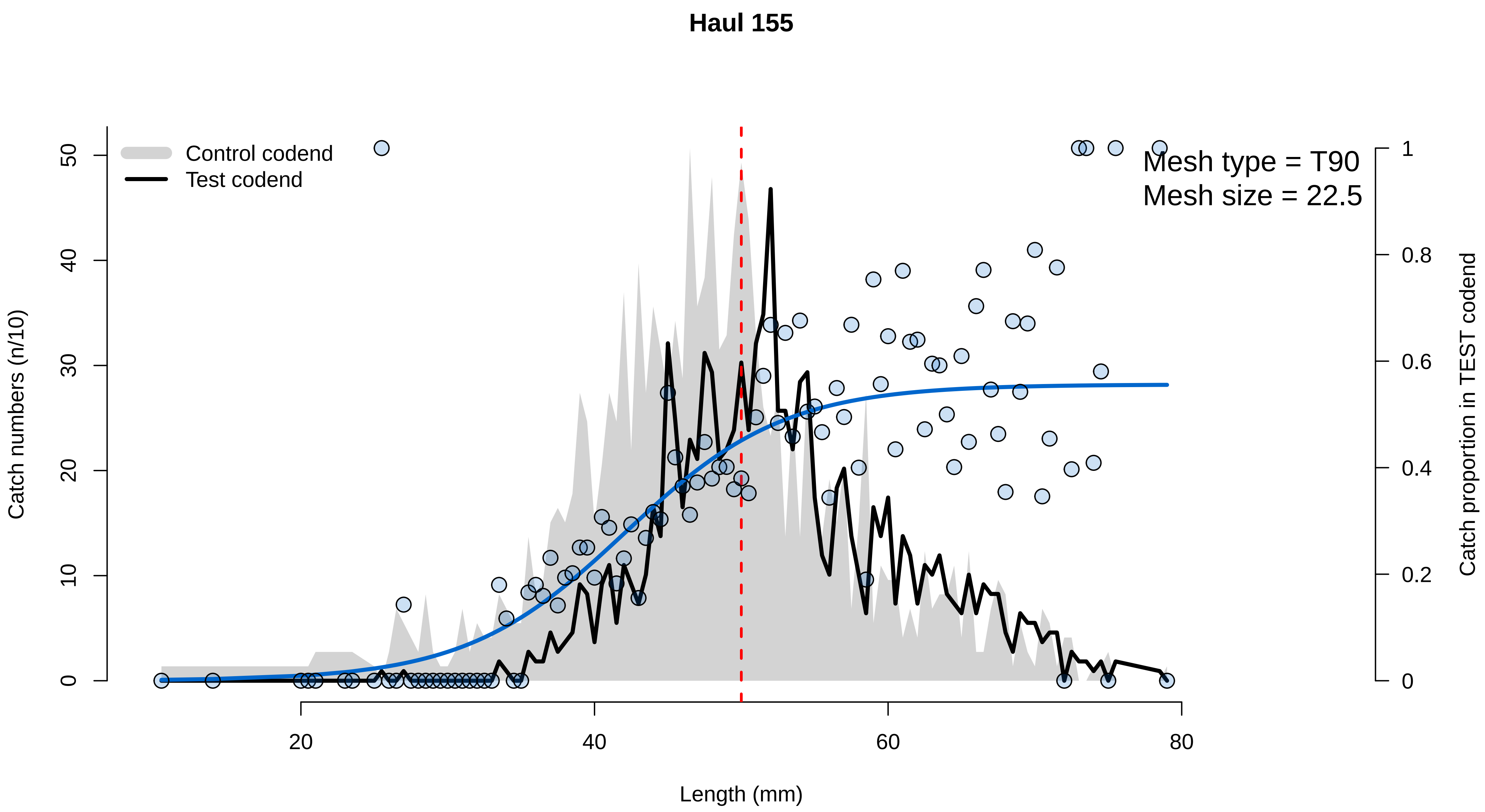
Haul 155
Control codend
Test codend
Mesh type = T90
Mesh size = 22.5
Catch numbers (n/10)
Catch proportion in TEST codend
Length (mm)
0
10
20
30
40
50
20
40
60
80
0
0.2
0.4
0.6
0.8
1

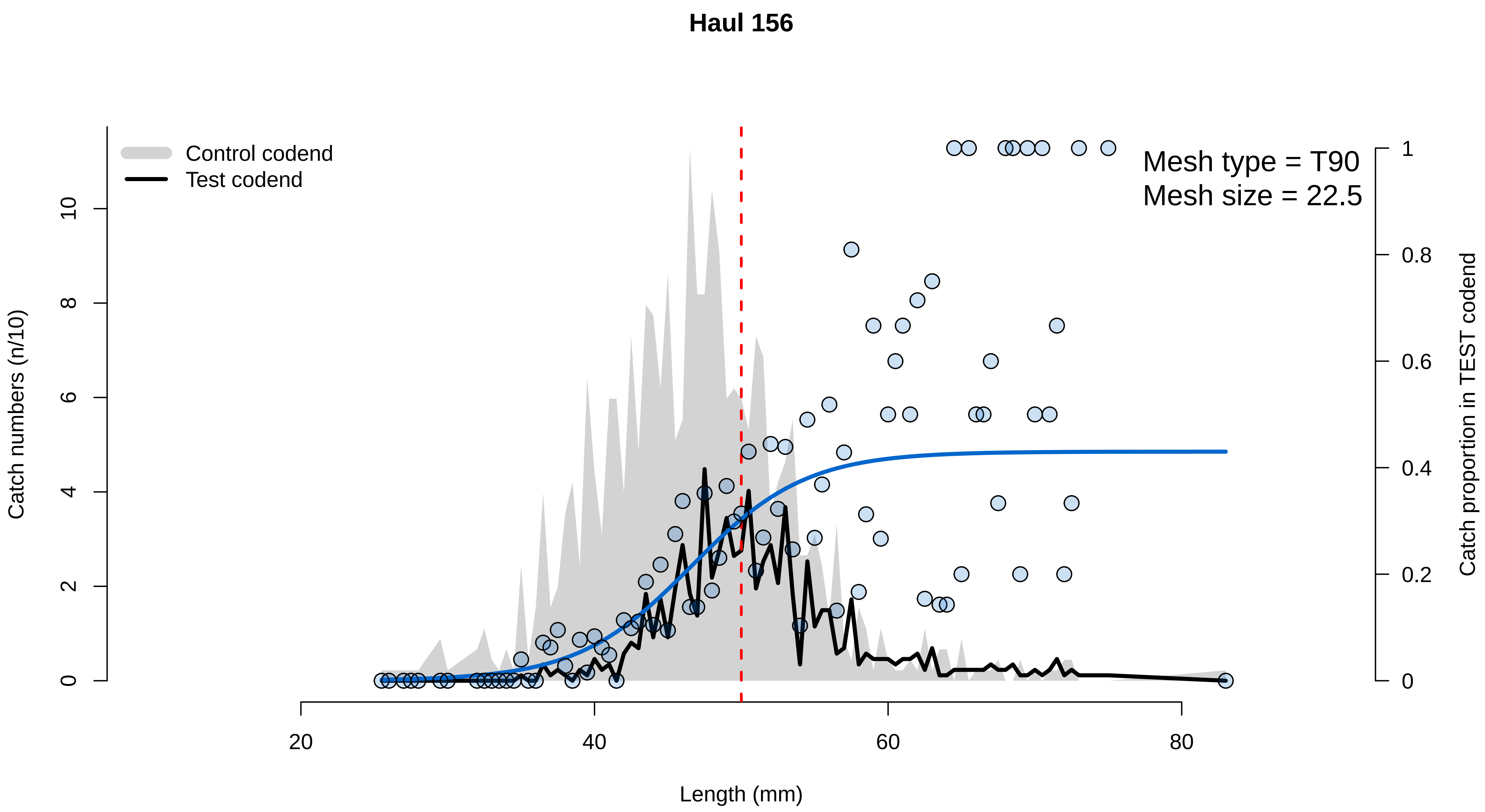
Haul 156
Control codend
Test codend
Mesh type = T90
Mesh size = 22.5
Catch numbers (n/10)
Catch proportion in TEST codend
Length (mm)
0
2
4
6
8
10
20
40
60
80
0
0.2
0.4
0.6
0.8
1

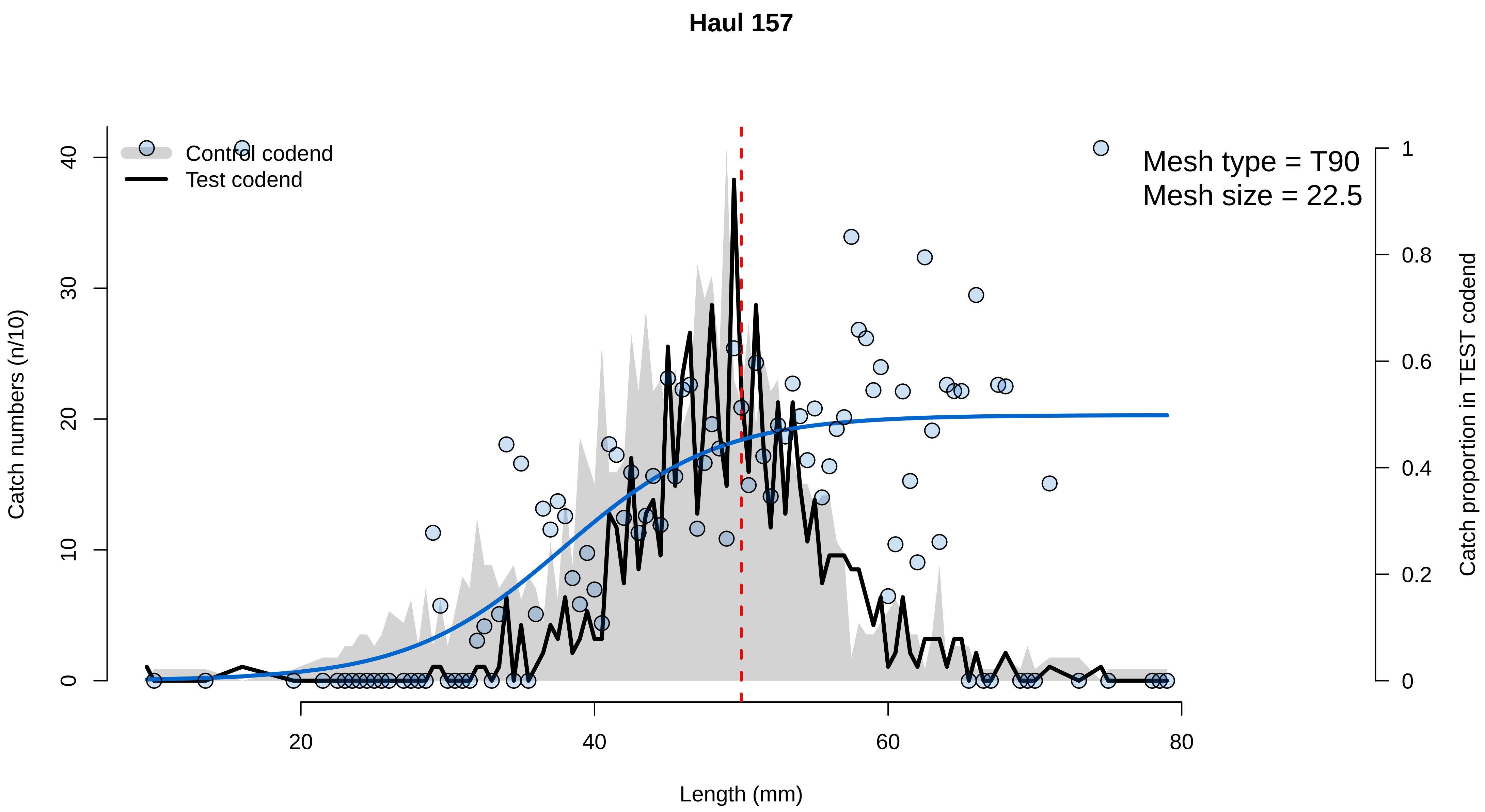
Haul 157
Control codend
Test codend
Mesh type = T90
Mesh size = 22.5
Catch numbers (n/10)
Catch proportion in TEST codend
Length (mm)
0
10
20
30
40
20
40
60
80
0
0.2
0.4
0.6
0.8
1

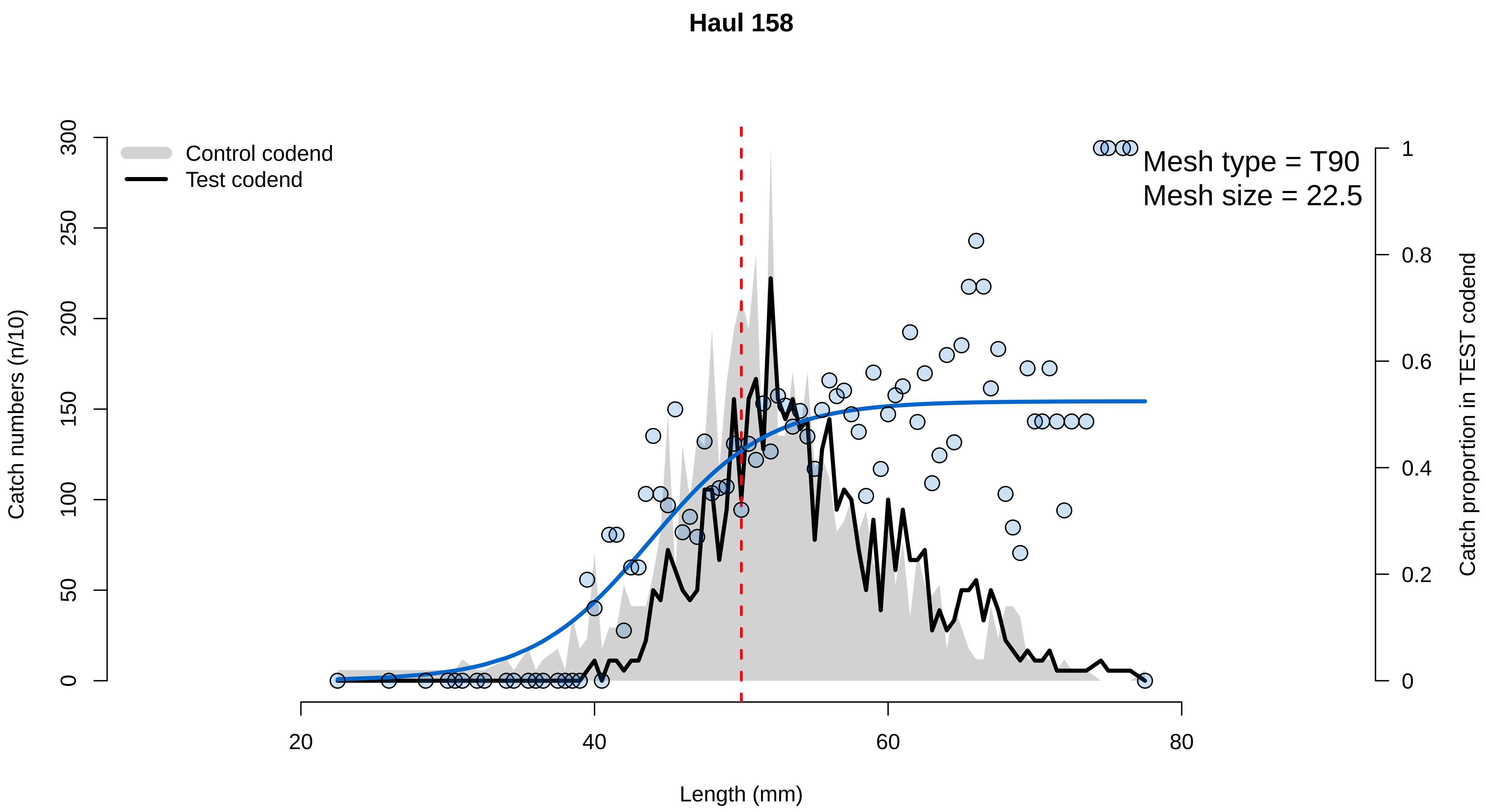
Haul 158
Control codend
Test codend
Mesh type = T90
Mesh size = 22.5
Catch numbers (n/10)
0
50
100
150
200
250
300
Catch proportion in TEST codend
0
0.2
0.4
0.6
0.8
1
20
40
60
80
Length (mm)

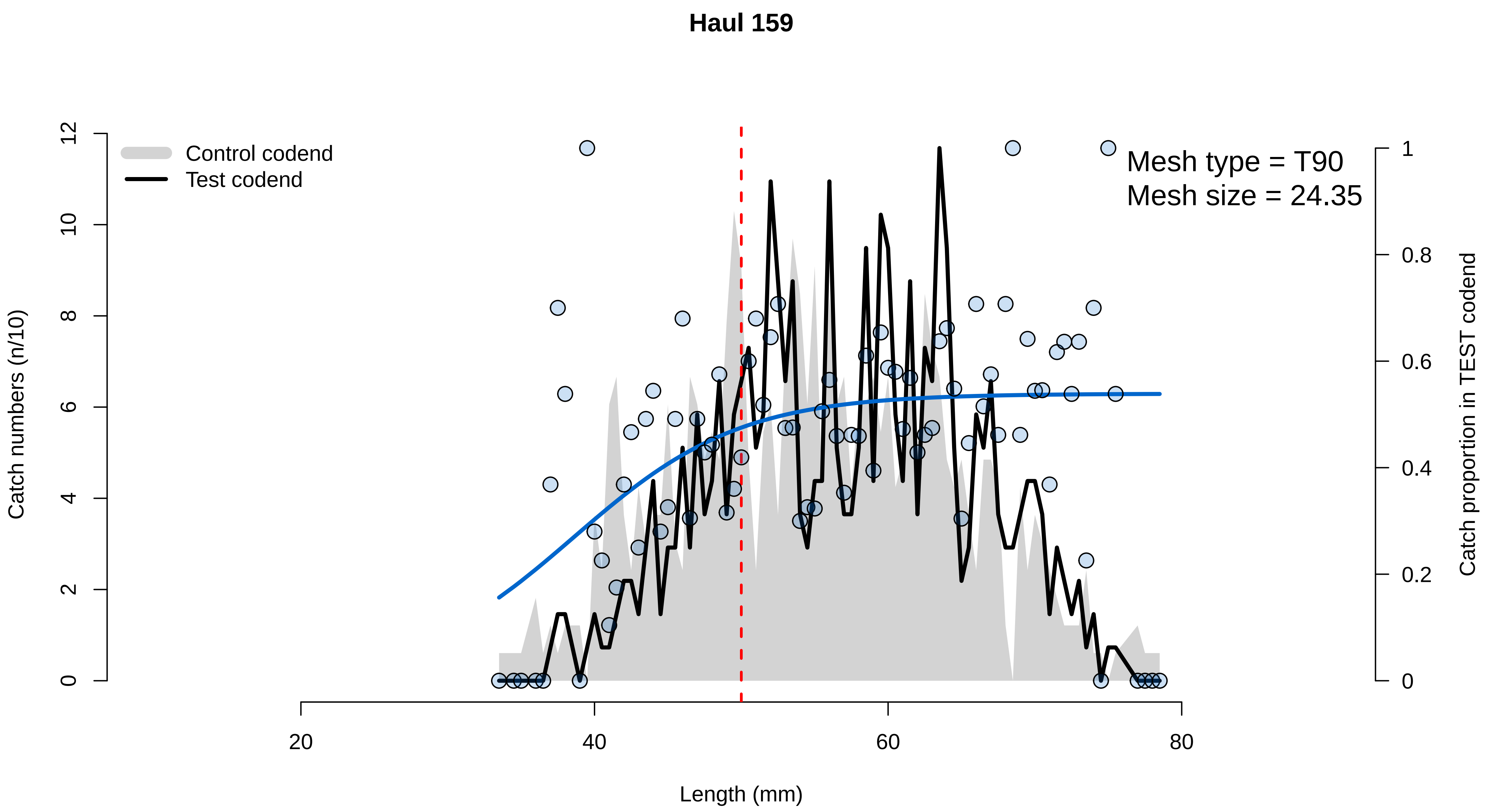
Haul 159
Control codend
Test codend
Mesh type = T90
Mesh size = 24.35
Catch numbers (n/10)
0
2
4
6
8
10
12
Catch proportion in TEST codend
0
0.2
0.4
0.6
0.8
1
20
40
60
80
Length (mm)

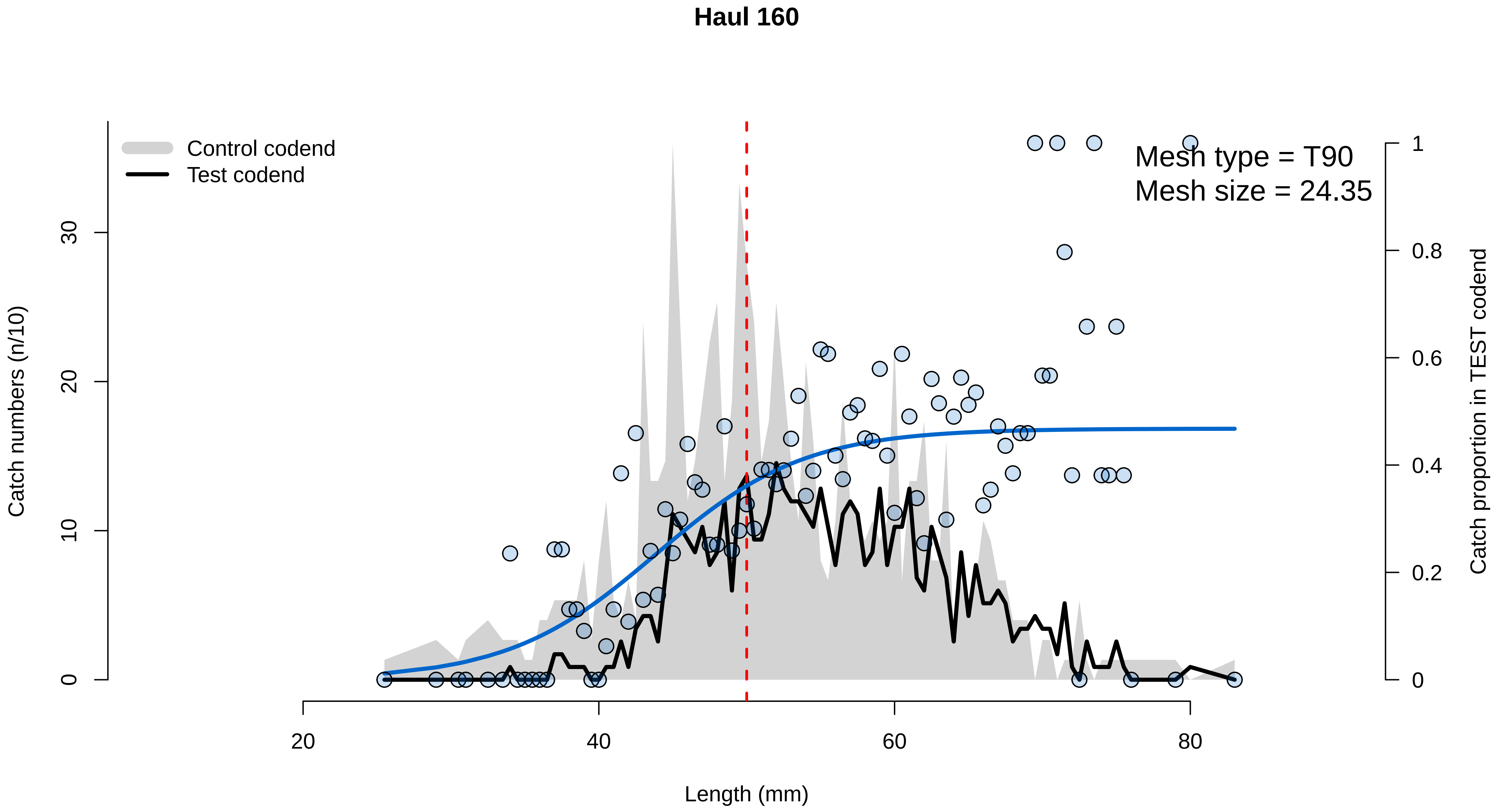

Haul 160
Control codend
Test codend
Mesh type = T90
Mesh size = 24.35
Catch numbers (n/10)
0
10
20
30
1
0.8
0.6
0.4
0.2
0
Catch proportion in TEST codend
20
40
60
80
Length (mm)

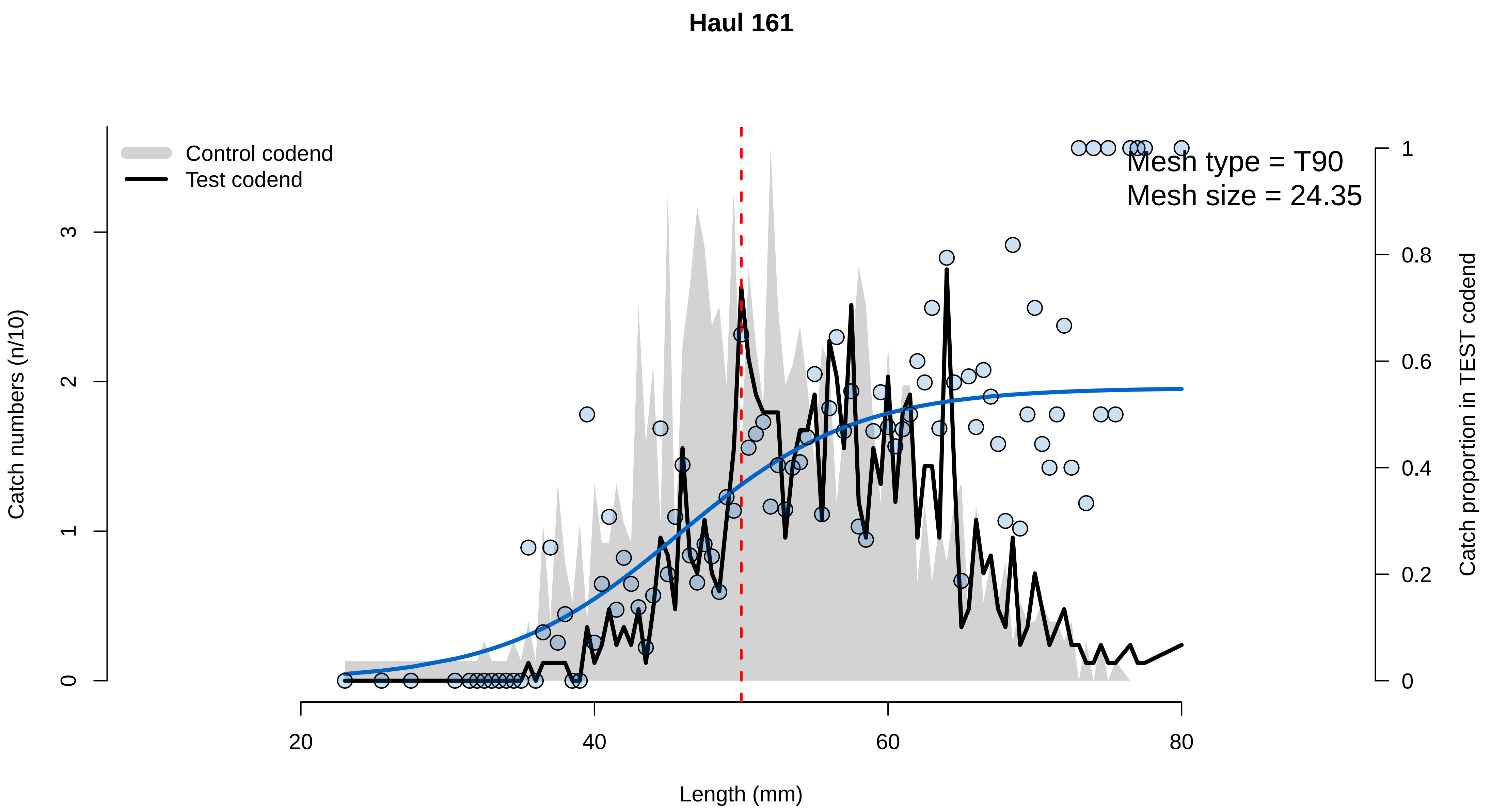

Haul 161
Control codend
Test codend
Mesh type = T90
Mesh size = 24.35
Catch numbers (n/10)
0
1
2
3
Catch proportion in TEST codend
0
0.2
0.4
0.6
0.8
1
20
40
60
80
Length (mm)

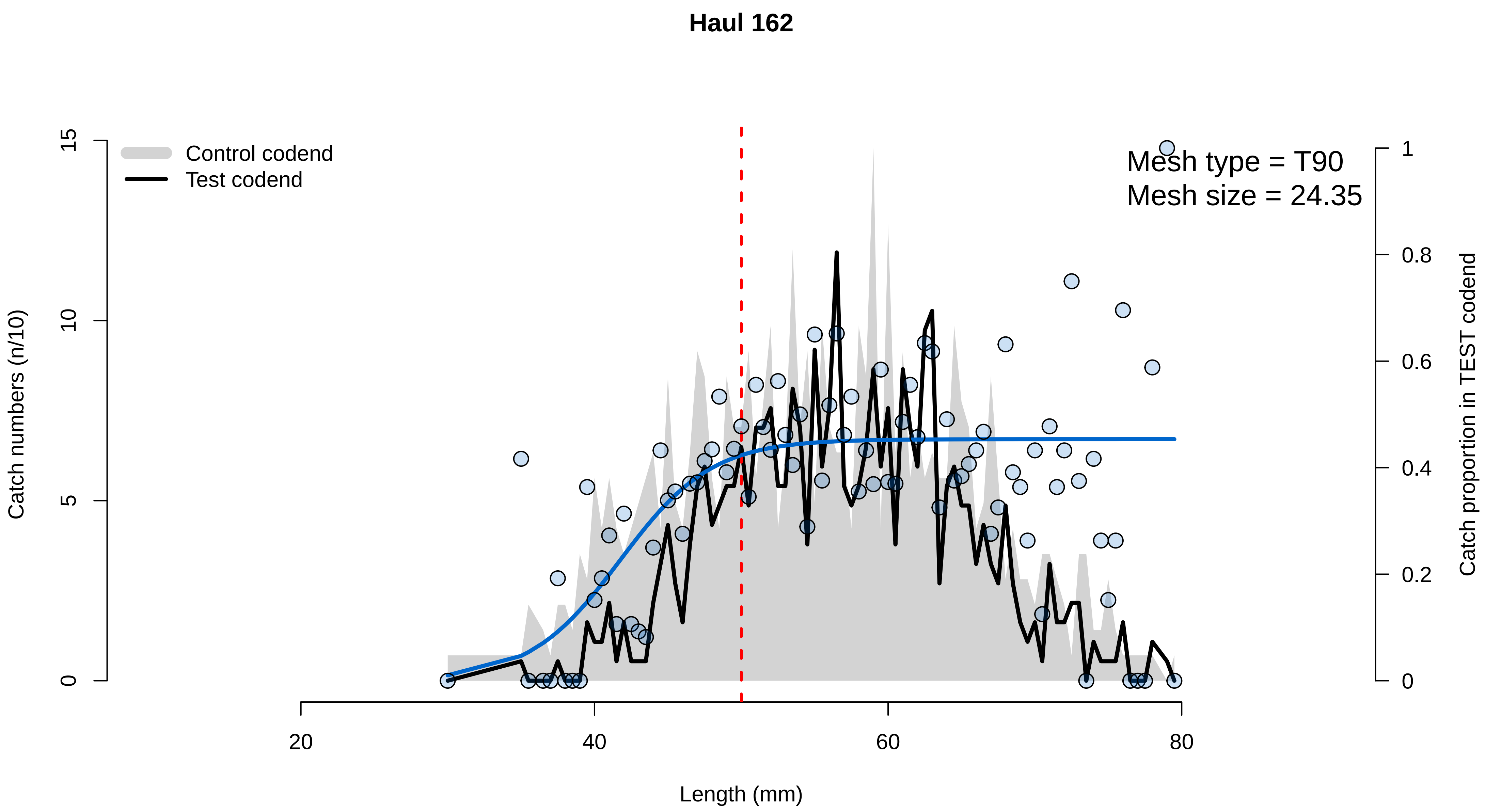

Haul 162
Control codend
Test codend
Mesh type = T90
Mesh size = 24.35
Catch numbers (n/10)
0
5
10
15
20
40
60
80
Length (mm)
Catch proportion in TEST codend
0
0.2
0.4
0.6
0.8
1

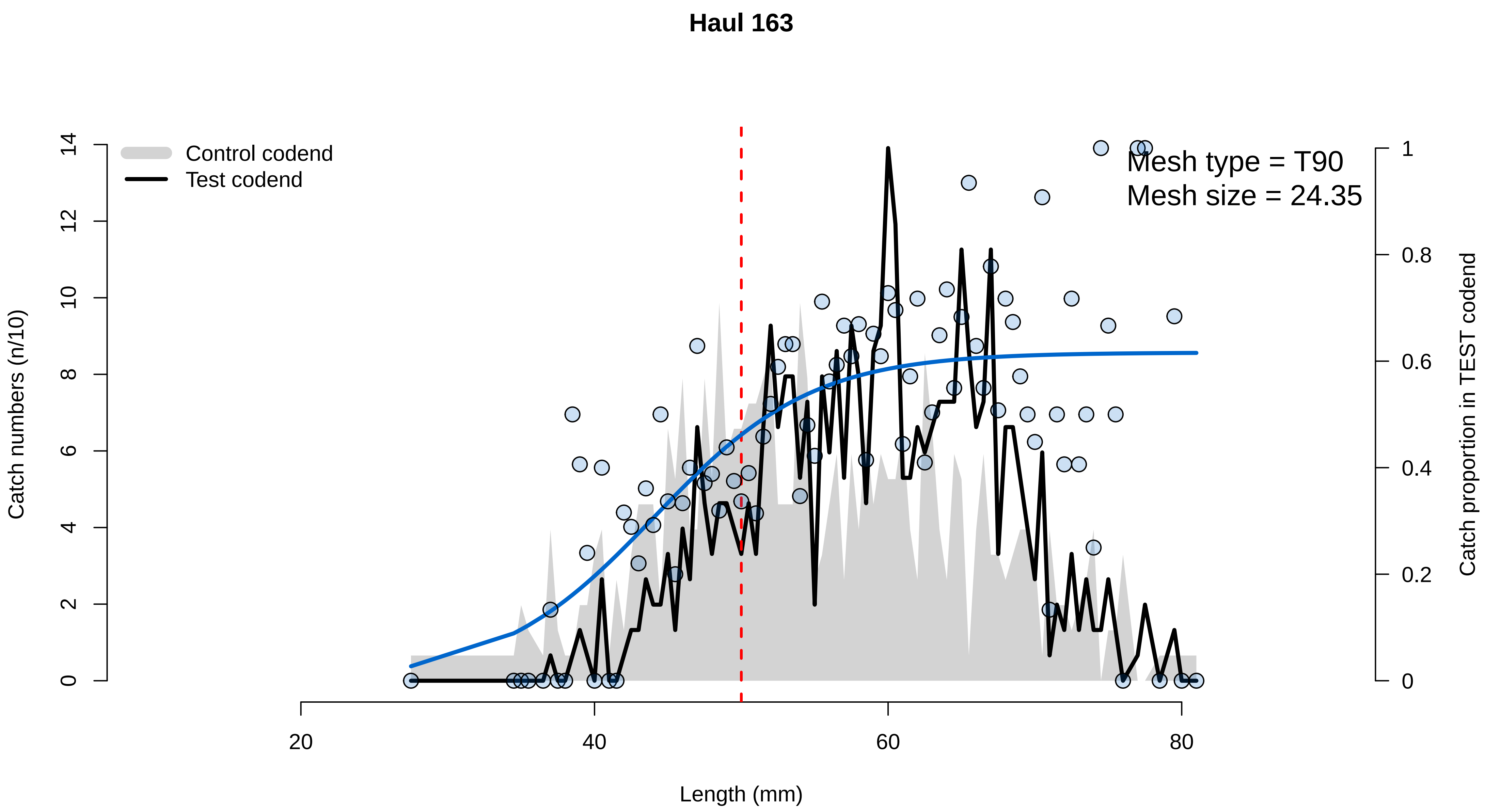
Haul 163
Control codend
Test codend
Mesh type = T90
Mesh size = 24.35
Catch numbers (n/10)
Catch proportion in TEST codend
Length (mm)
0
2
4
6
8
10
12
14
0
0.2
0.4
0.6
0.8
1
20
40
60
80

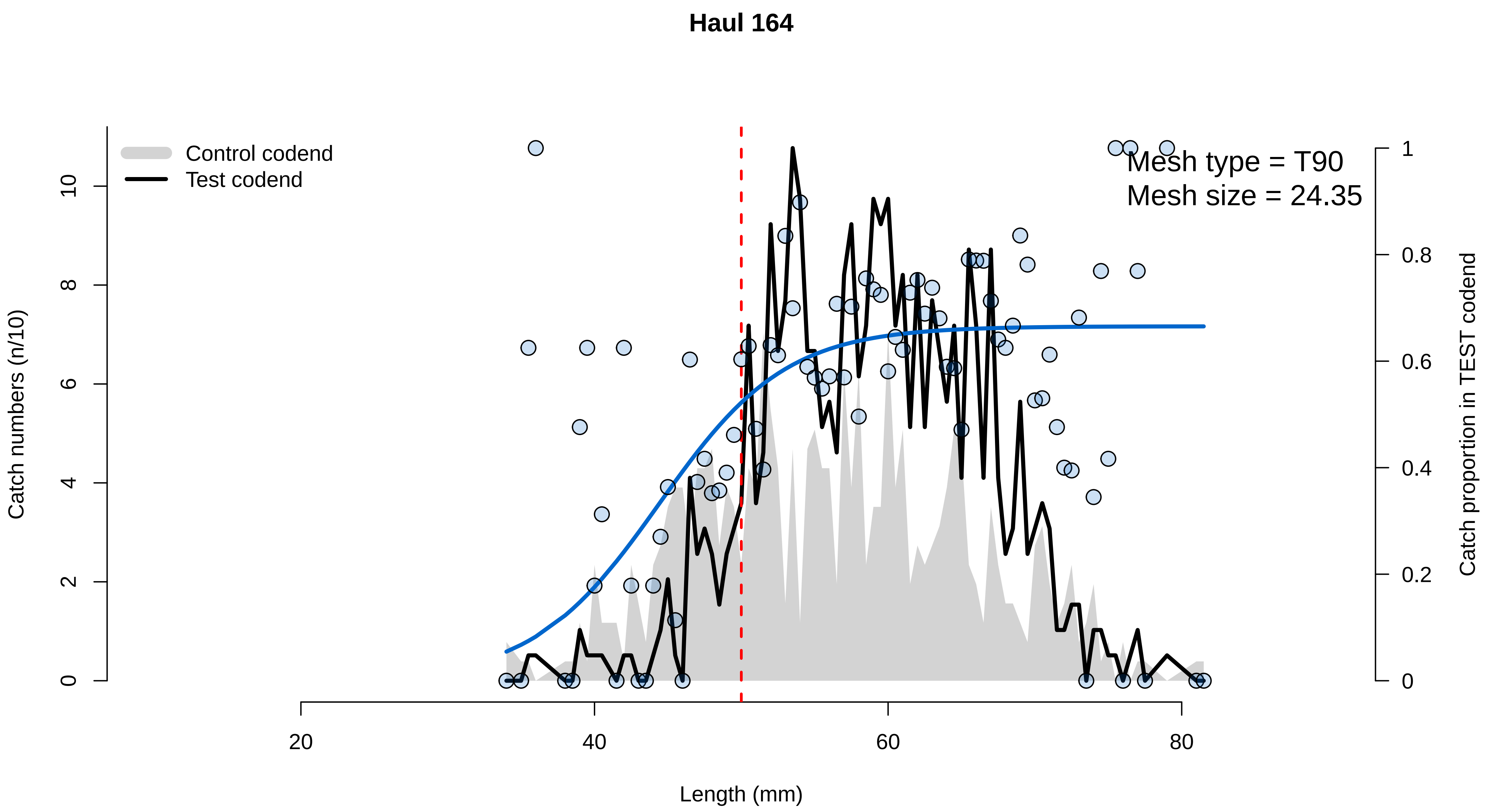
Haul 164
Control codend
Test codend
Mesh type = T90
Mesh size = 24.35
Catch numbers (n/10)
Catch proportion in TEST codend
Length (mm)
0
2
4
6
8
10
20
40
60
80
0
0.2
0.4
0.6
0.8
1

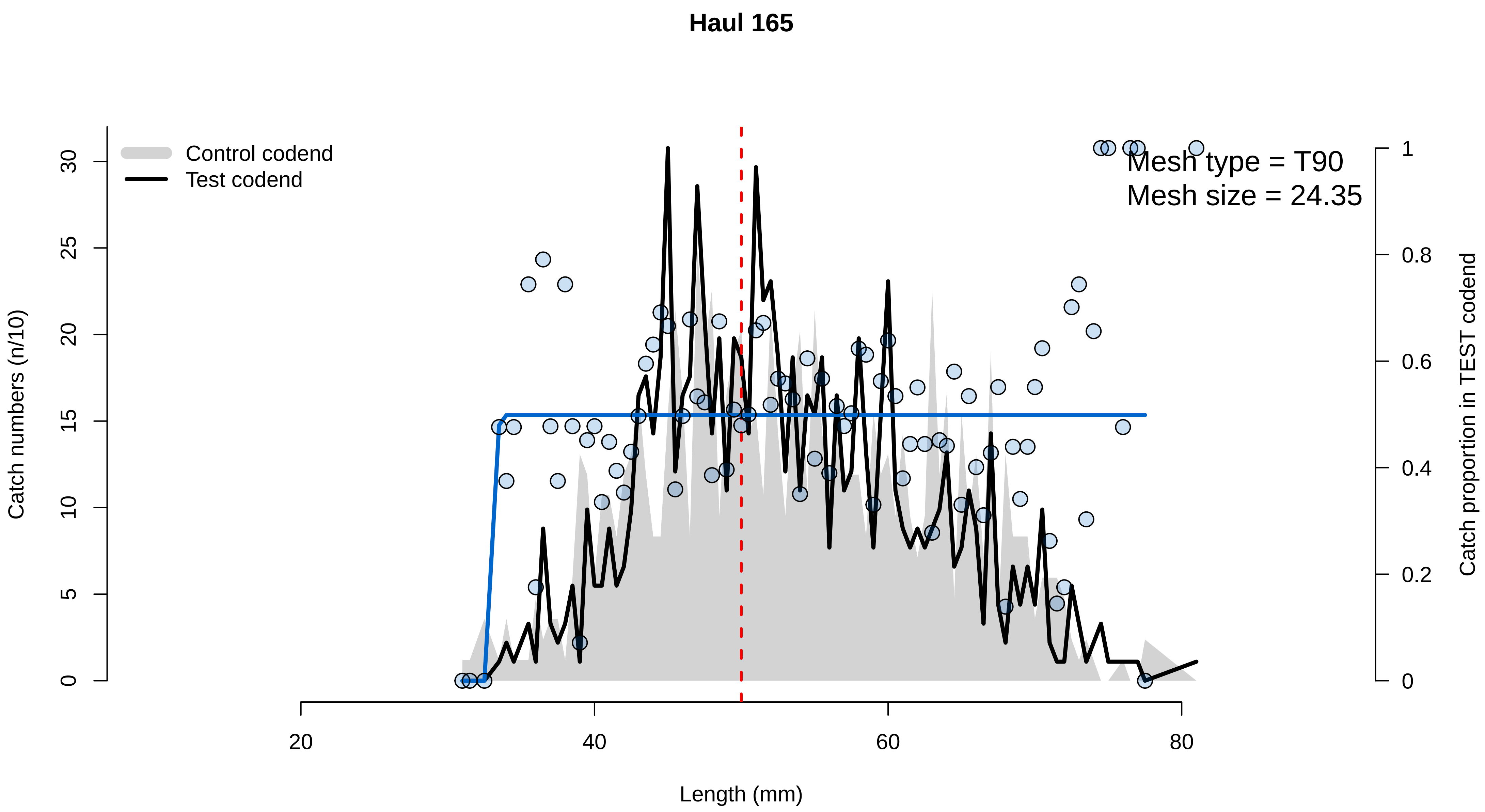

Haul 165
Control codend
Test codend
Mesh type = T90
Mesh size = 24.35
Catch numbers (n/10)
0
5
10
15
20
25
30
Catch proportion in TEST codend
0
0.2
0.4
0.6
0.8
1
20
40
60
80
Length (mm)

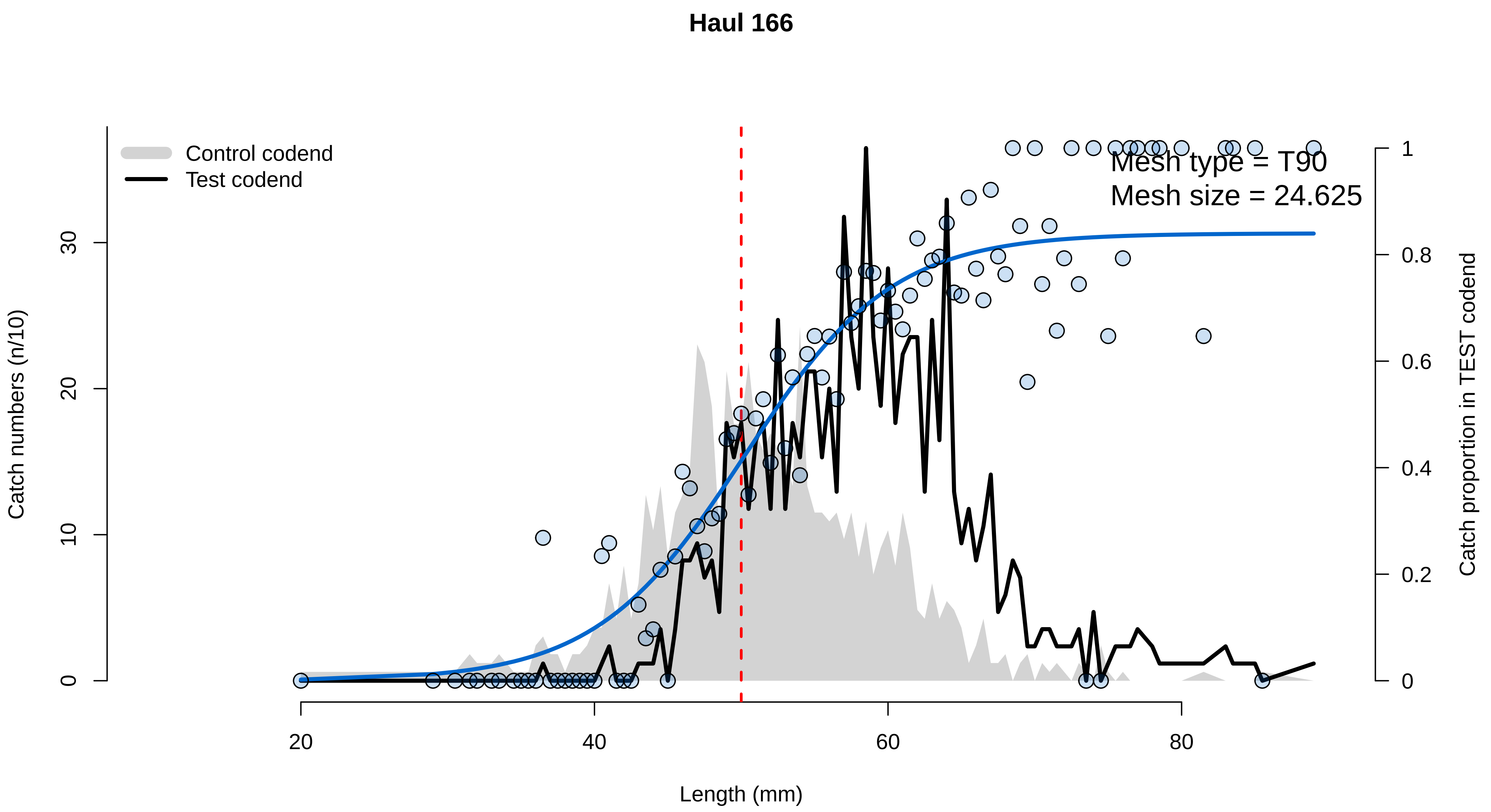
Haul 166
Control codend
Test codend
Mesh type = T90
Mesh size = 24.625
Catch numbers (n/10)
0
10
20
30
Catch proportion in TEST codend
0
0.2
0.4
0.6
0.8
1
20
40
60
80
Length (mm)

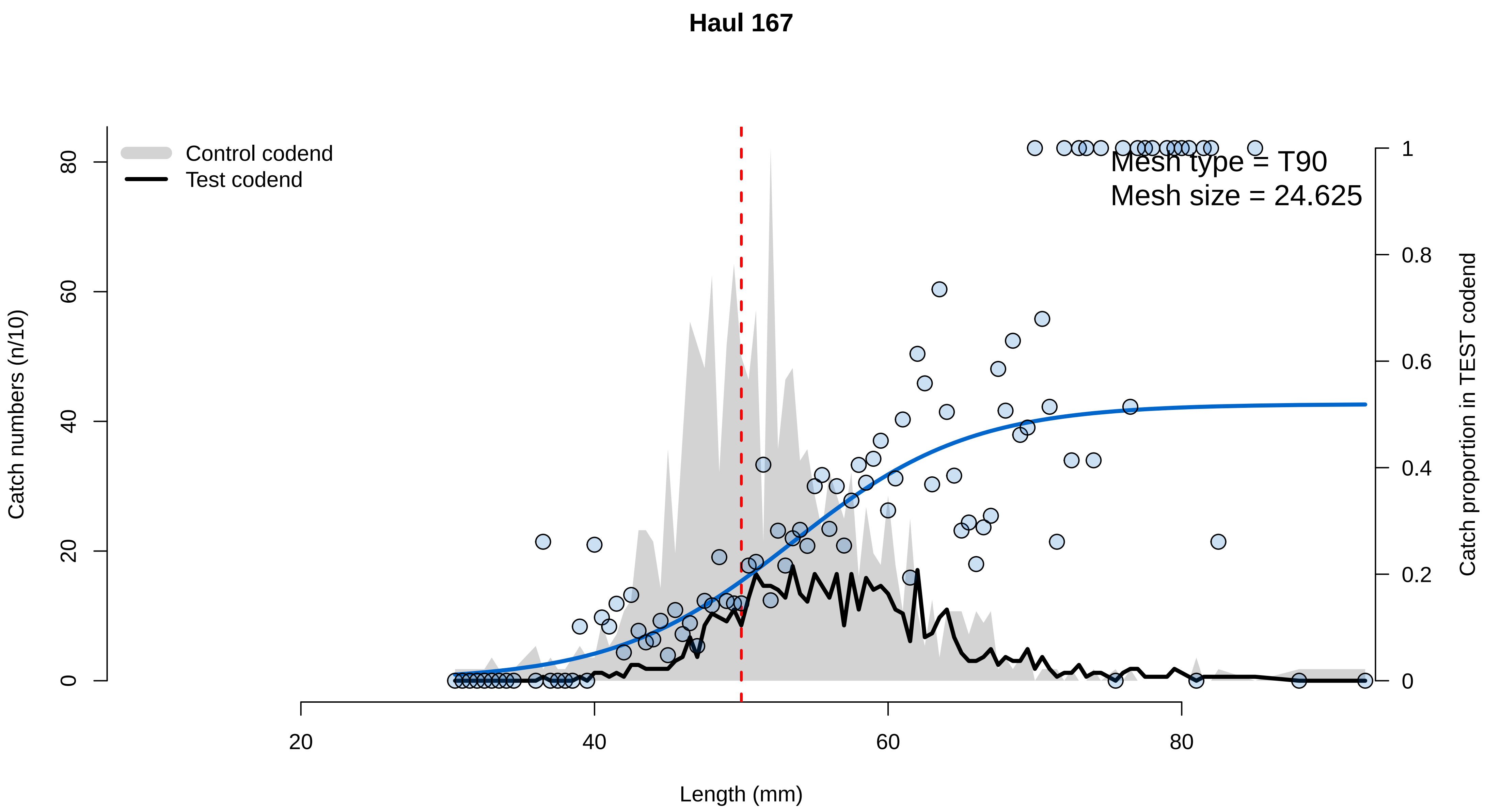
Haul 167
Control codend
Test codend
Mesh type = T90
Mesh size = 24.625
Catch numbers (n/10)
0
20
40
60
80
Catch proportion in TEST codend
0
0.2
0.4
0.6
0.8
1
20
40
60
80
Length (mm)

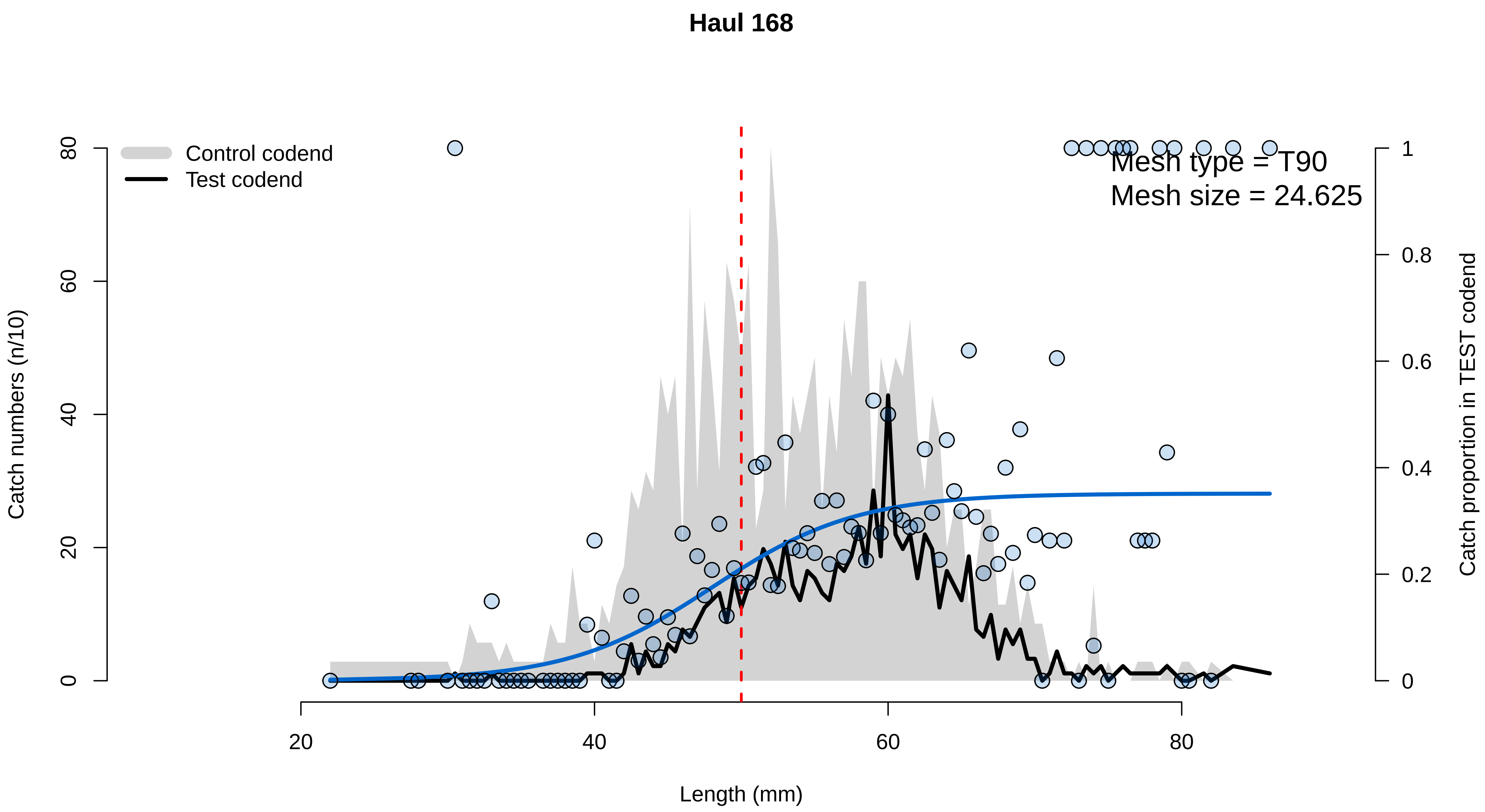

Haul 168
Control codend
Test codend
Mesh type = T90
Mesh size = 24.625
Catch numbers (n/10)
0
20
40
60
80
Catch proportion in TEST codend
0
0.2
0.4
0.6
0.8
1
20
40
60
80
Length (mm)

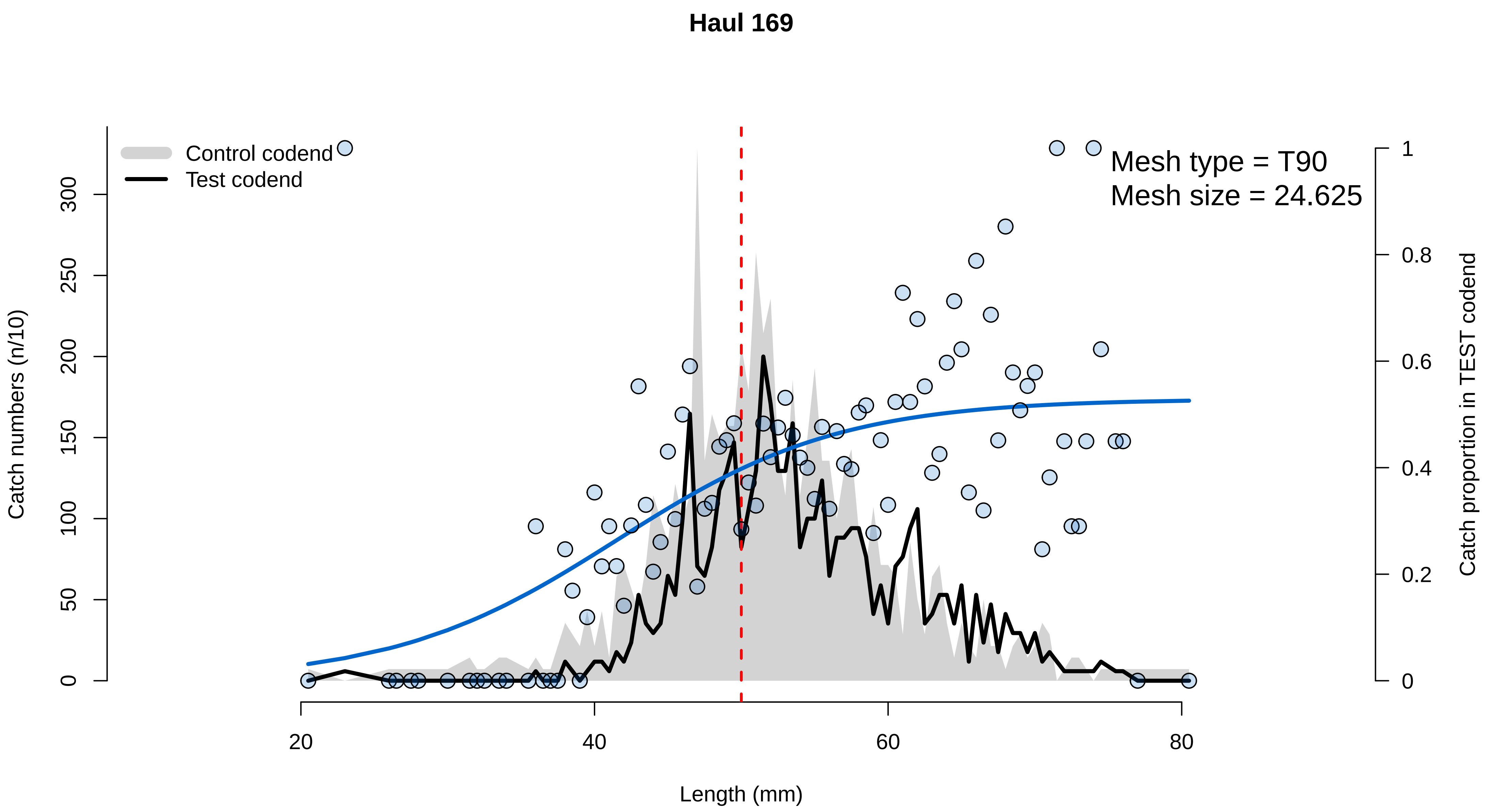

Haul 169
Control codend
Test codend
Mesh type = T90
Mesh size = 24.625
Catch numbers (n/10)
0
50
100
150
200
250
300
Catch proportion in TEST codend
0
0.2
0.4
0.6
0.8
1
20
40
60
80
Length (mm)

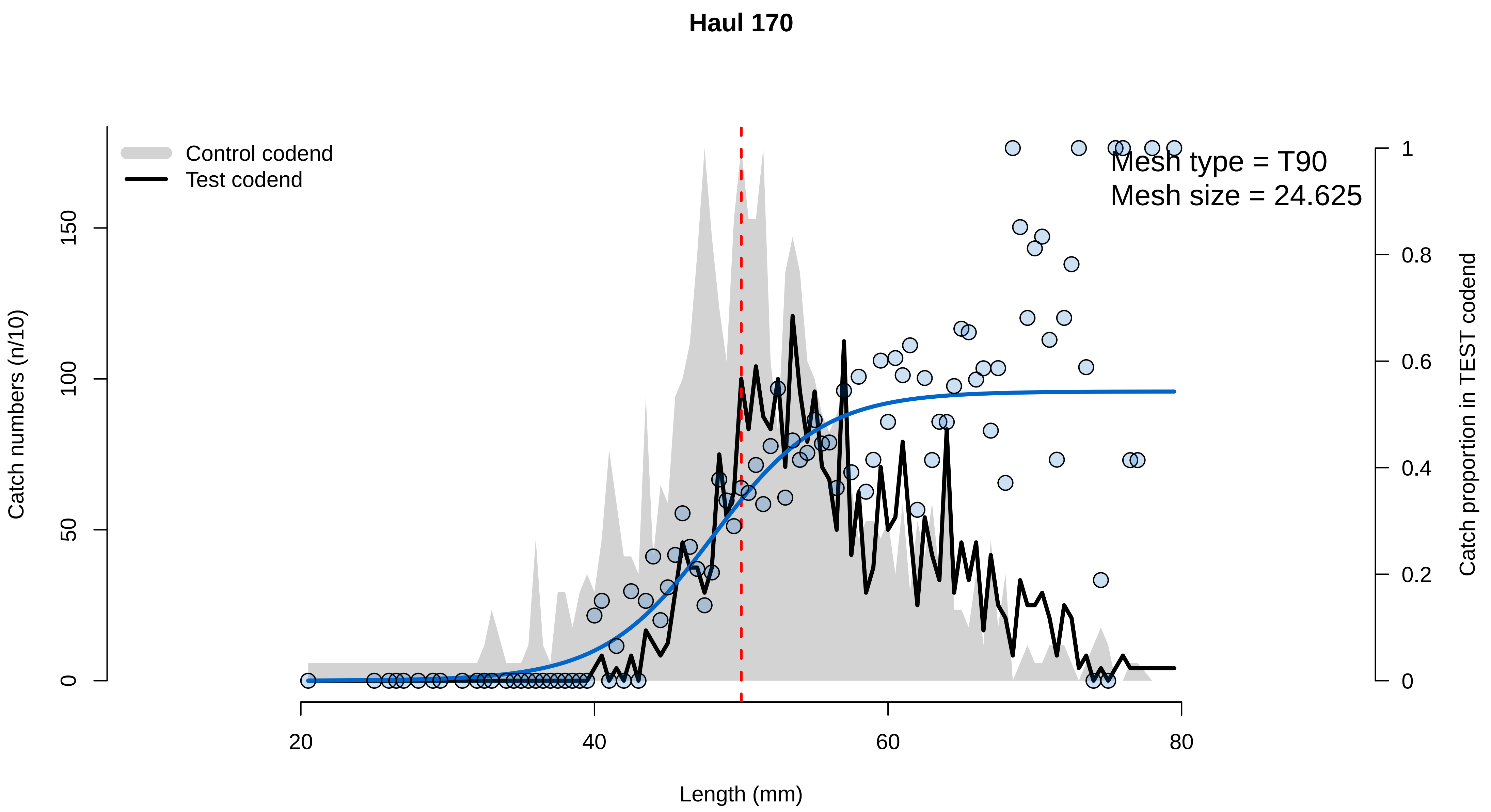
Haul 170
Control codend
Test codend
Mesh type = T90
Mesh size = 24.625
Catch numbers (n/10)
0
50
100
150
Catch proportion in TEST codend
0
0.2
0.4
0.6
0.8
1
20
40
60
80
Length (mm)

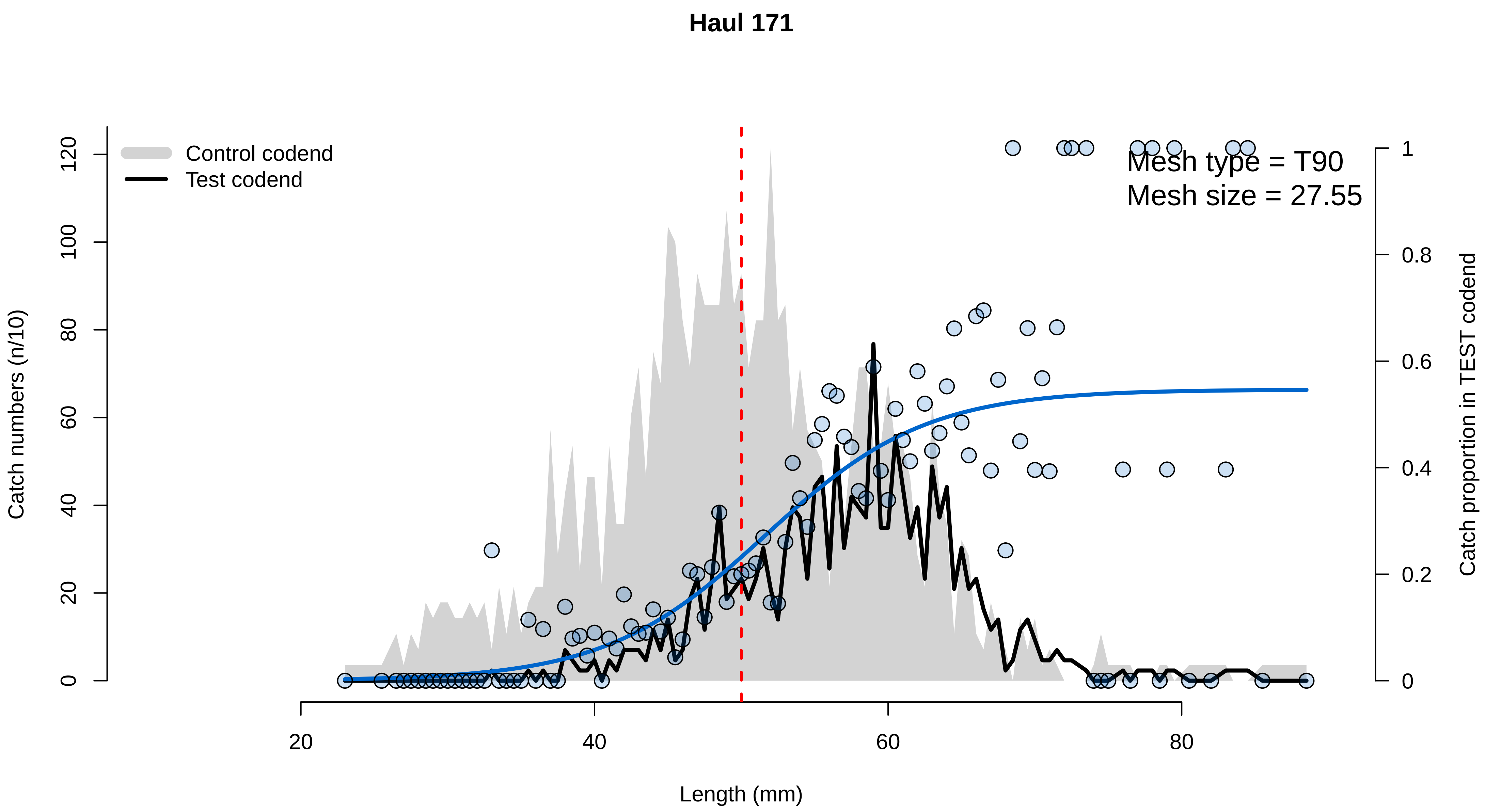
Haul 171
Control codend
Test codend
Mesh type = T90
Mesh size = 27.55
Catch numbers (n/10)
0
20
40
60
80
100
120
Catch proportion in TEST codend
0
0.2
0.4
0.6
0.8
1
20
40
60
80
Length (mm)

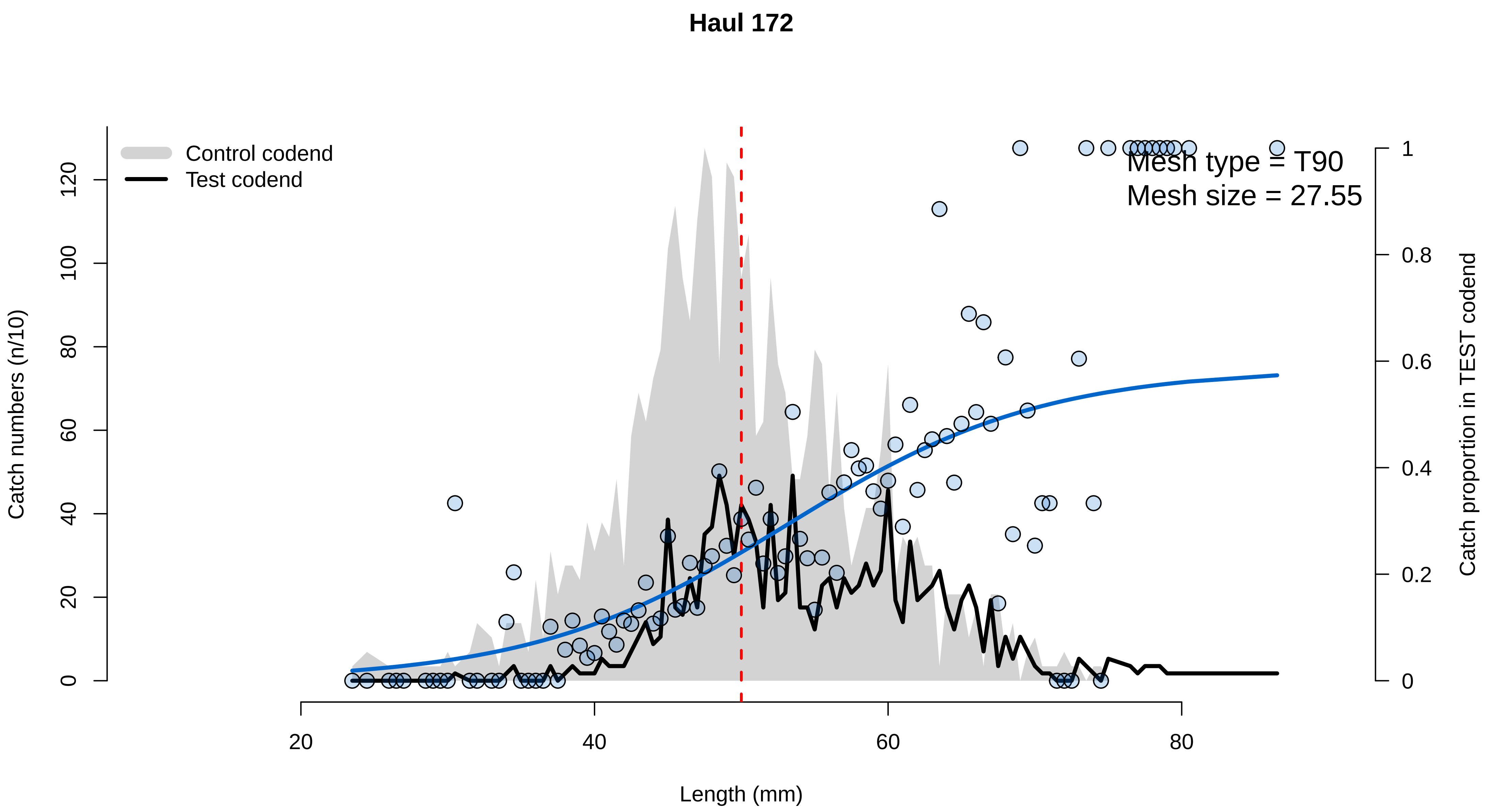
Haul 172
Control codend
Test codend
Mesh type = T90
Mesh size = 27.55
Catch numbers (n/10)
0
20
40
60
80
100
120
Catch proportion in TEST codend
0
0.2
0.4
0.6
0.8
1
Length (mm)
20
40
60
80

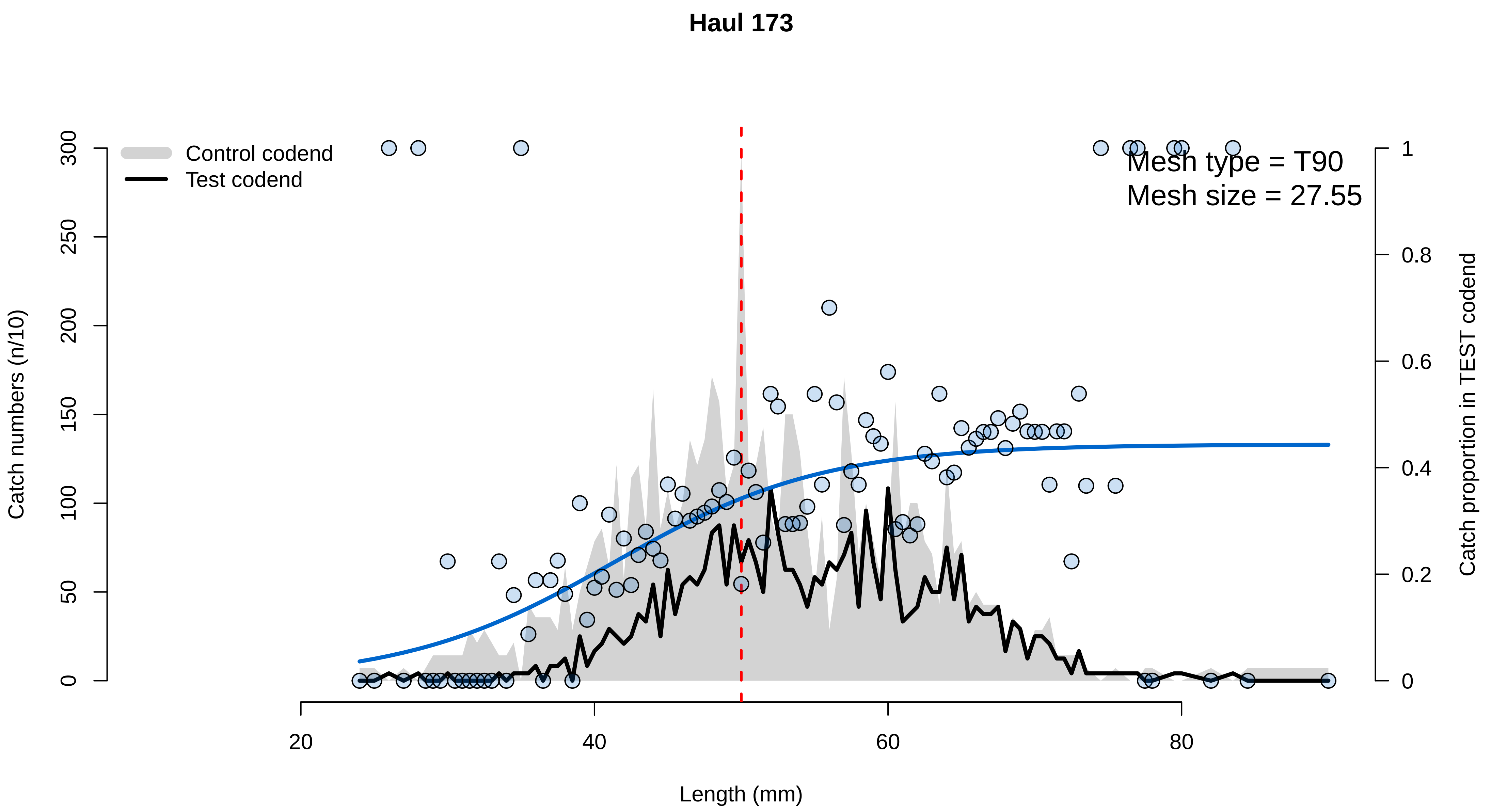

Haul 173
Control codend
Test codend
Mesh type = T90
Mesh size = 27.55
Catch numbers (n/10)
Catch proportion in TEST codend
Length (mm)
0
50
100
150
200
250
300
0
0.2
0.4
0.6
0.8
1
20
40
60
80

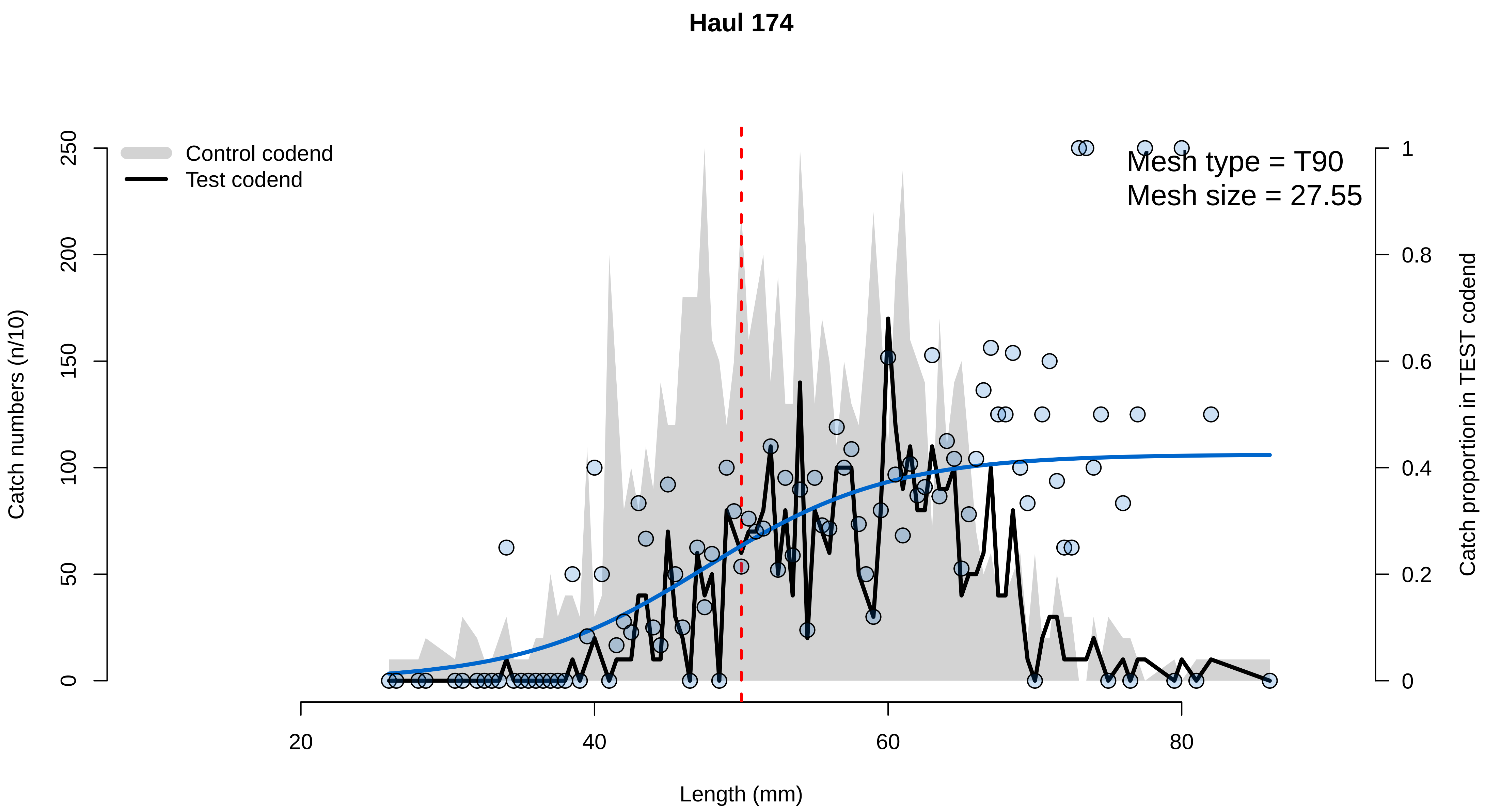
Haul 174
Control codend
Test codend
Mesh type = T90
Mesh size = 27.55
Catch numbers (n/10)
0
50
100
150
200
250
Catch proportion in TEST codend
0
0.2
0.4
0.6
0.8
1
20
40
60
80
Length (mm)

Haul 175

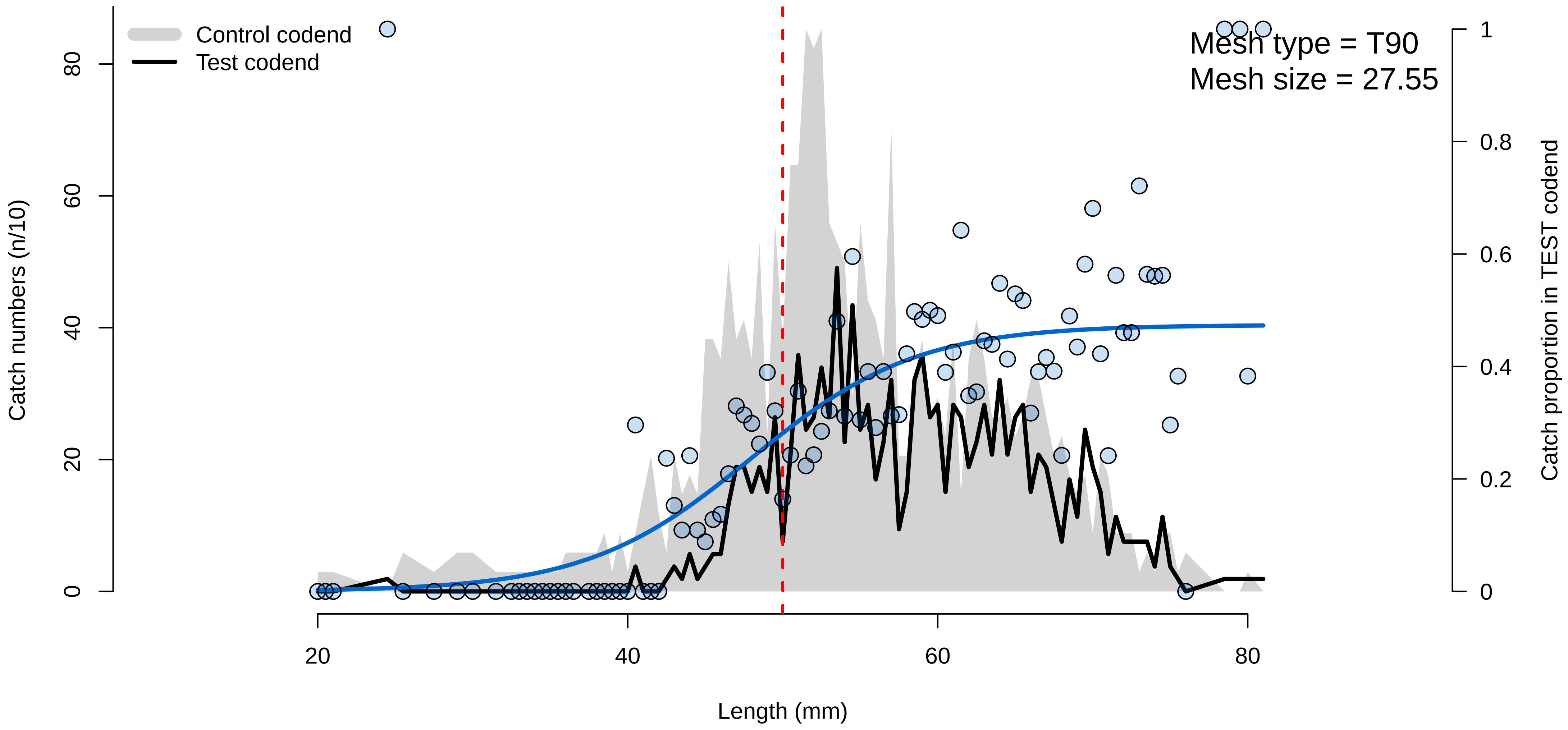

Control codend
Test codend
Mesh type = T90
Mesh size = 27.55
Catch numbers (n/10)
Catch proportion in TEST codend
Length (mm)
0
20
40
60
80
0
0.2
0.4
0.6
0.8
1
20
40
60
80

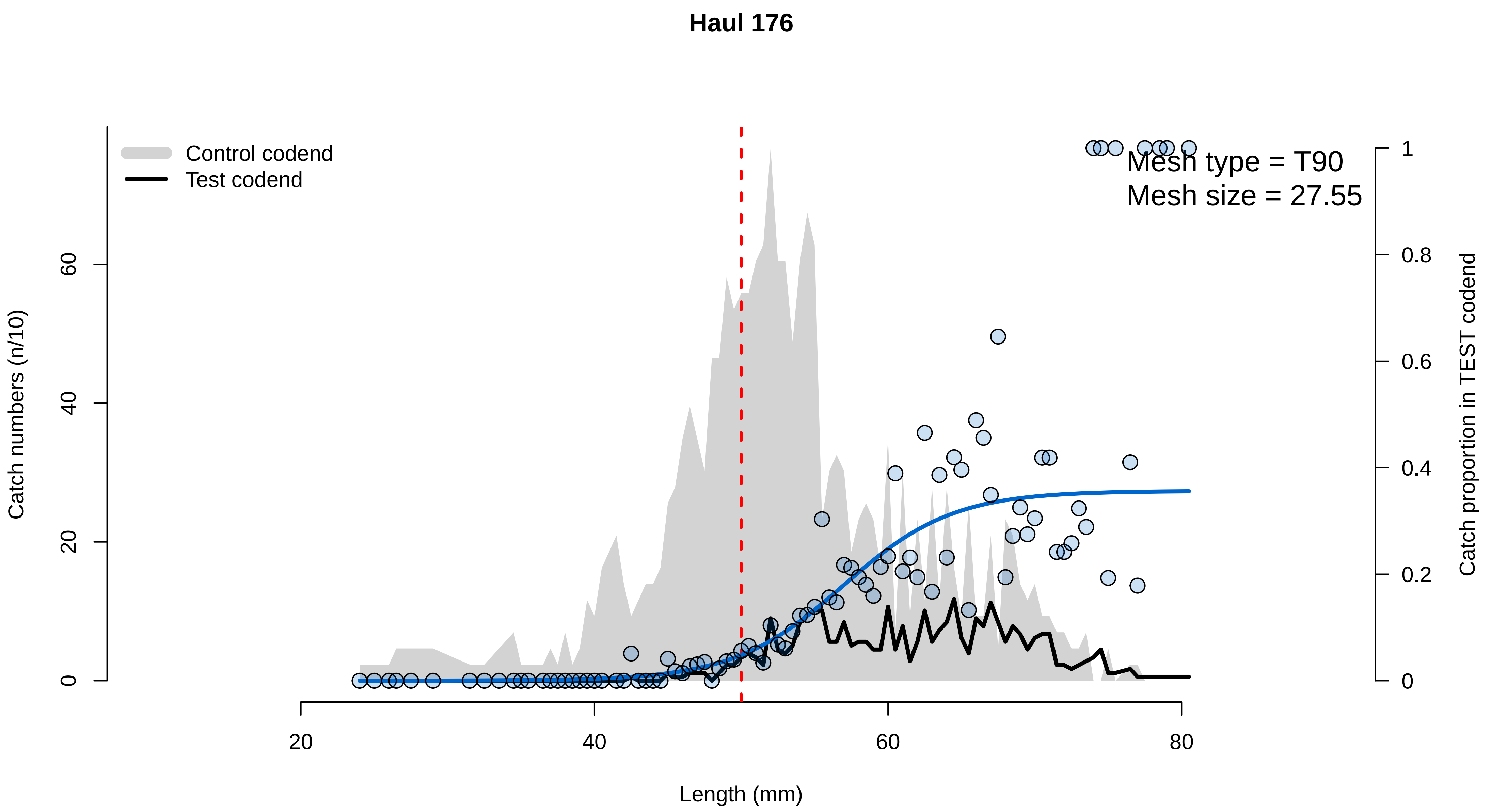

Haul 176
Control codend
Test codend
Mesh type = T90
Mesh size = 27.55
Catch numbers (n/10)
Catch proportion in TEST codend
Length (mm)
0
20
40
60
20
40
60
80
0
0.2
0.4
0.6
0.8
1

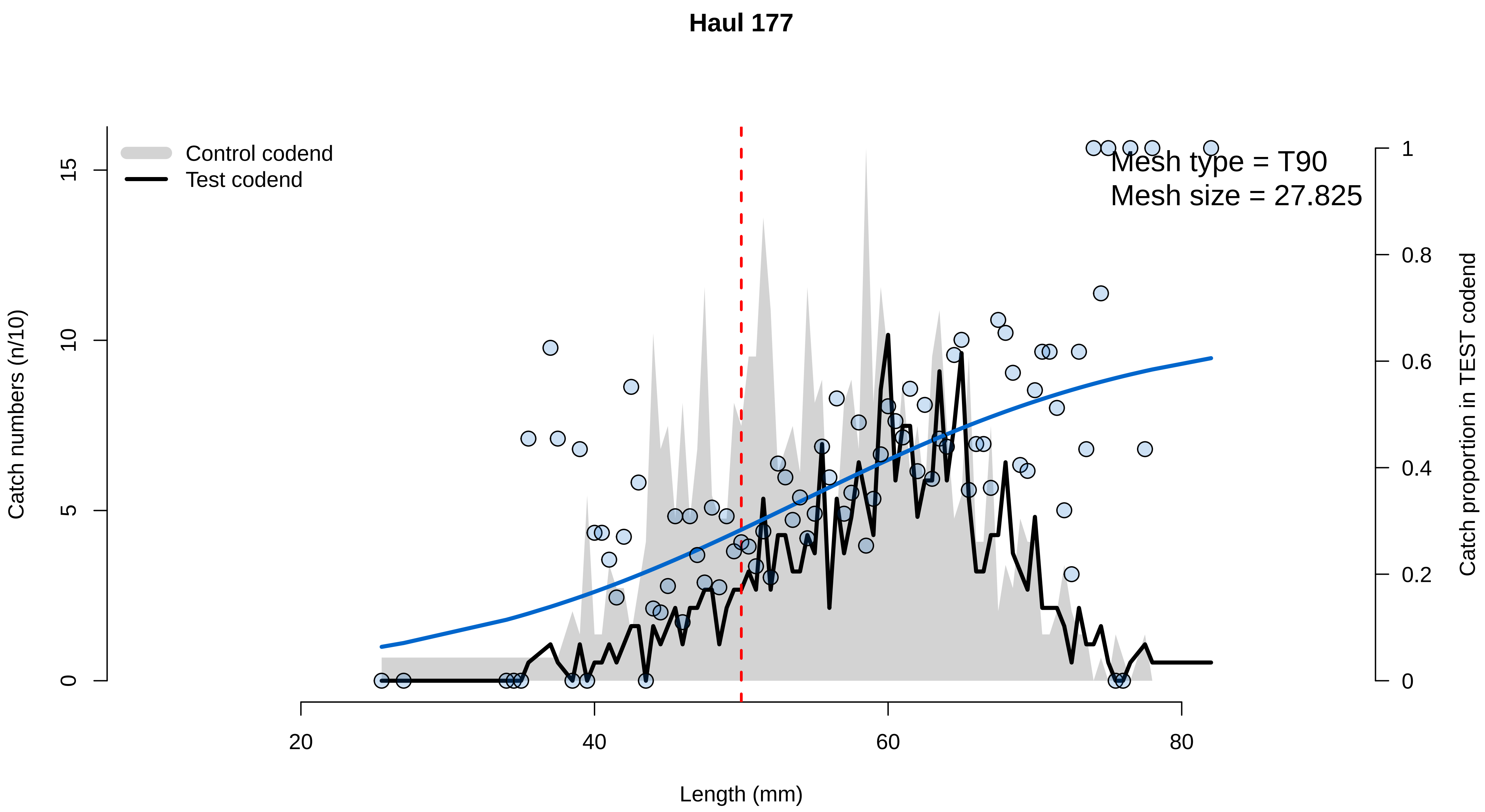

Haul 177
Control codend
Test codend
Mesh type = T90
Mesh size = 27.825
Catch numbers (n/10)
0
5
10
15
Catch proportion in TEST codend
0
0.2
0.4
0.6
0.8
1
20
40
60
80
Length (mm)

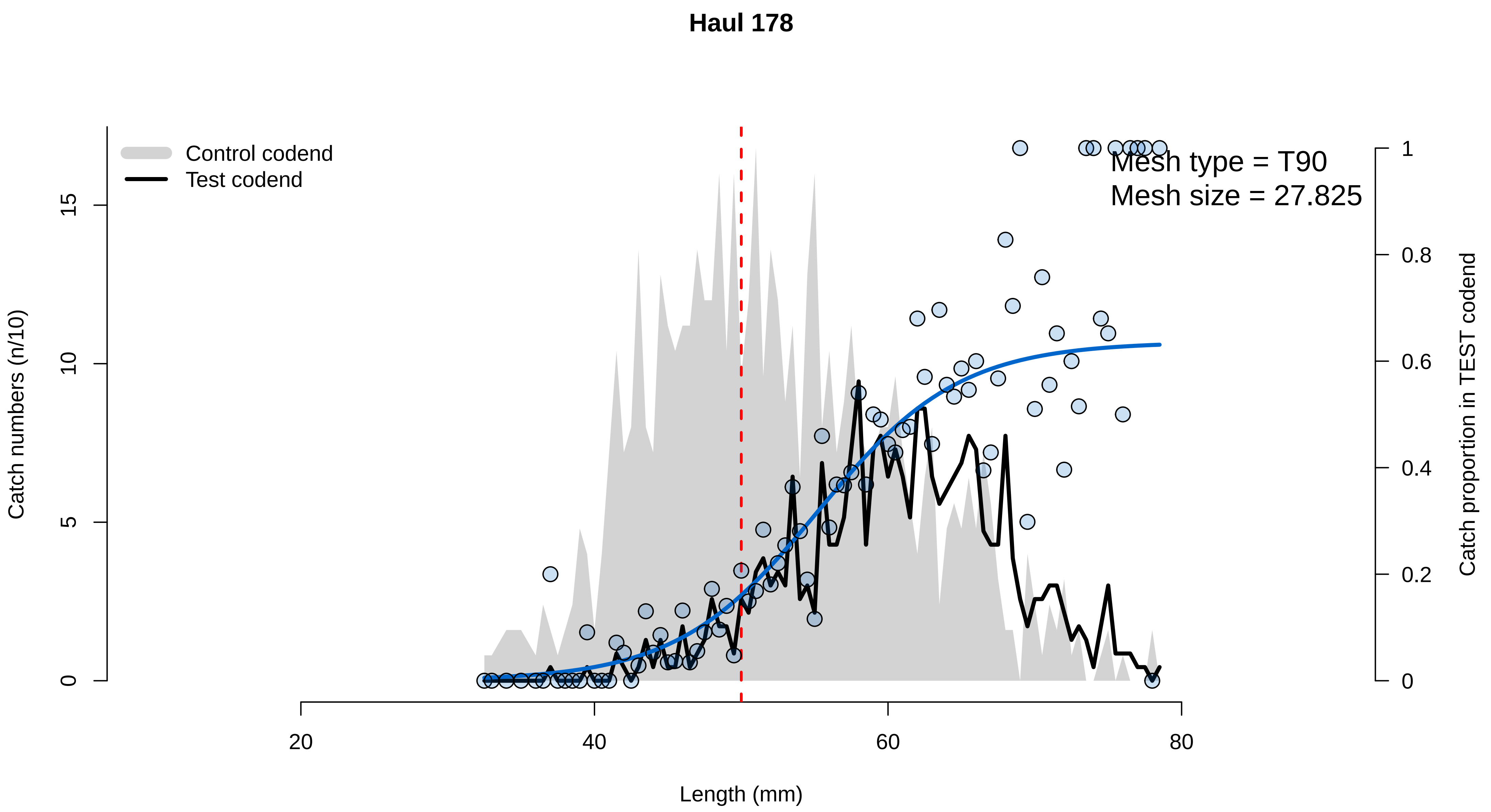

Haul 178
Control codend
Test codend
Mesh type = T90
Mesh size = 27.825
Catch numbers (n/10)
0
5
10
15
Catch proportion in TEST codend
0
0.2
0.4
0.6
0.8
1
20
40
60
80
Length (mm)

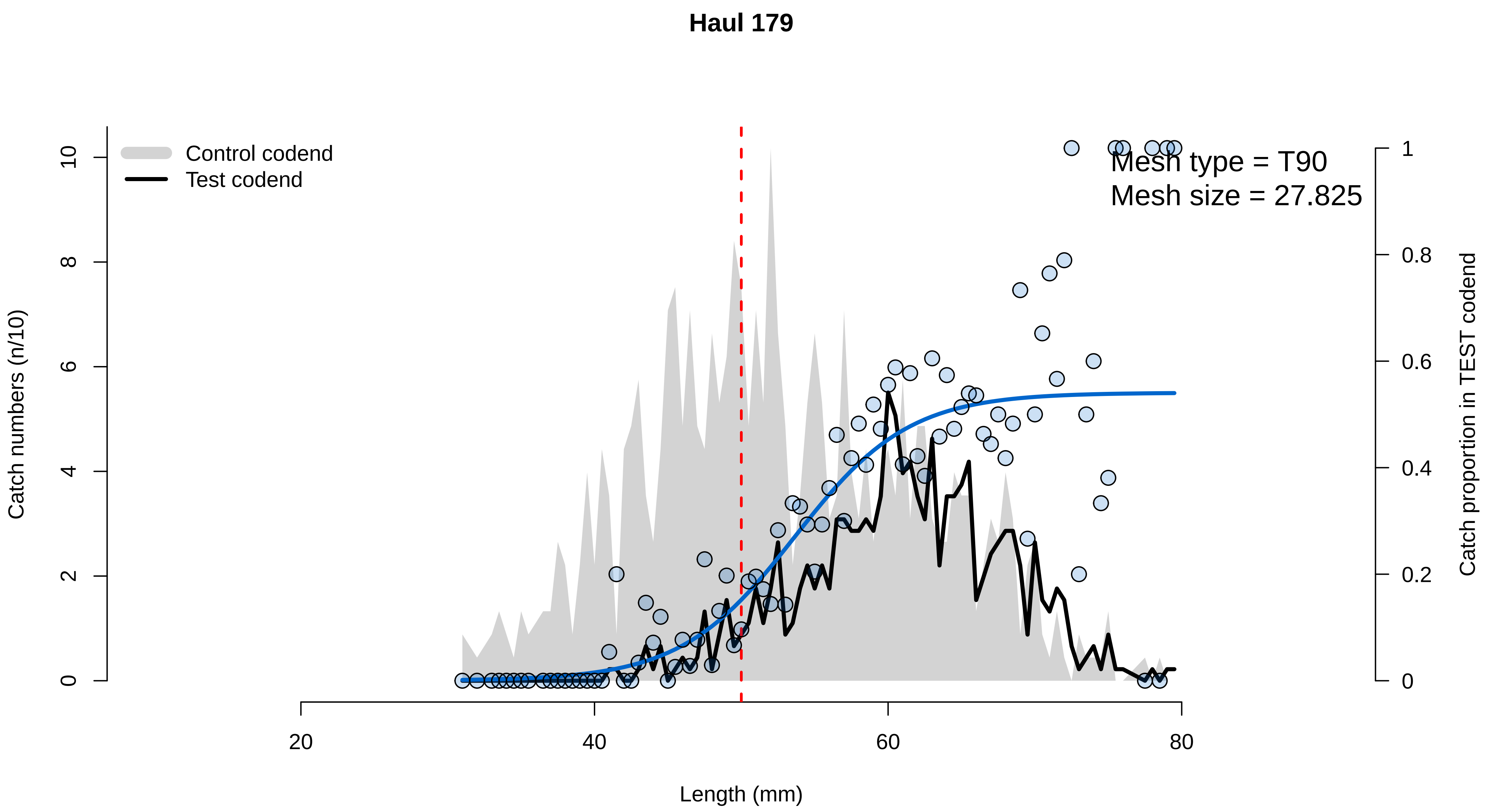

Haul 179
Control codend
Test codend
Mesh type = T90
Mesh size = 27.825
Catch numbers (n/10)
Catch proportion in TEST codend
Length (mm)
0
2
4
6
8
10
0
0.2
0.4
0.6
0.8
1
20
40
60
80

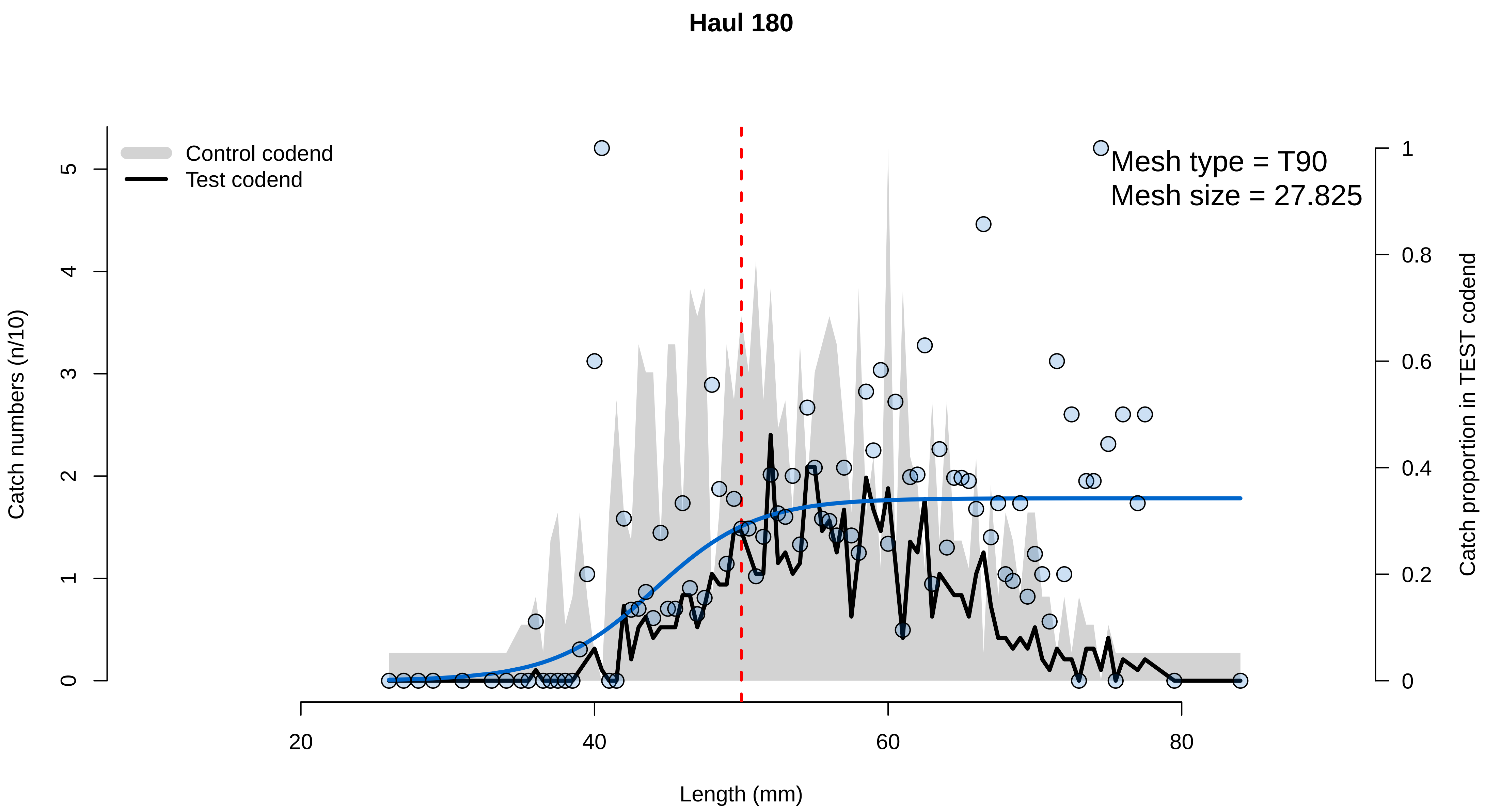

Haul 180
Control codend
Test codend
Mesh type = T90
Mesh size = 27.825
Catch numbers (n/10)
0
1
2
3
4
5
Catch proportion in TEST codend
0
0.2
0.4
0.6
0.8
1
20
40
60
80
Length (mm)

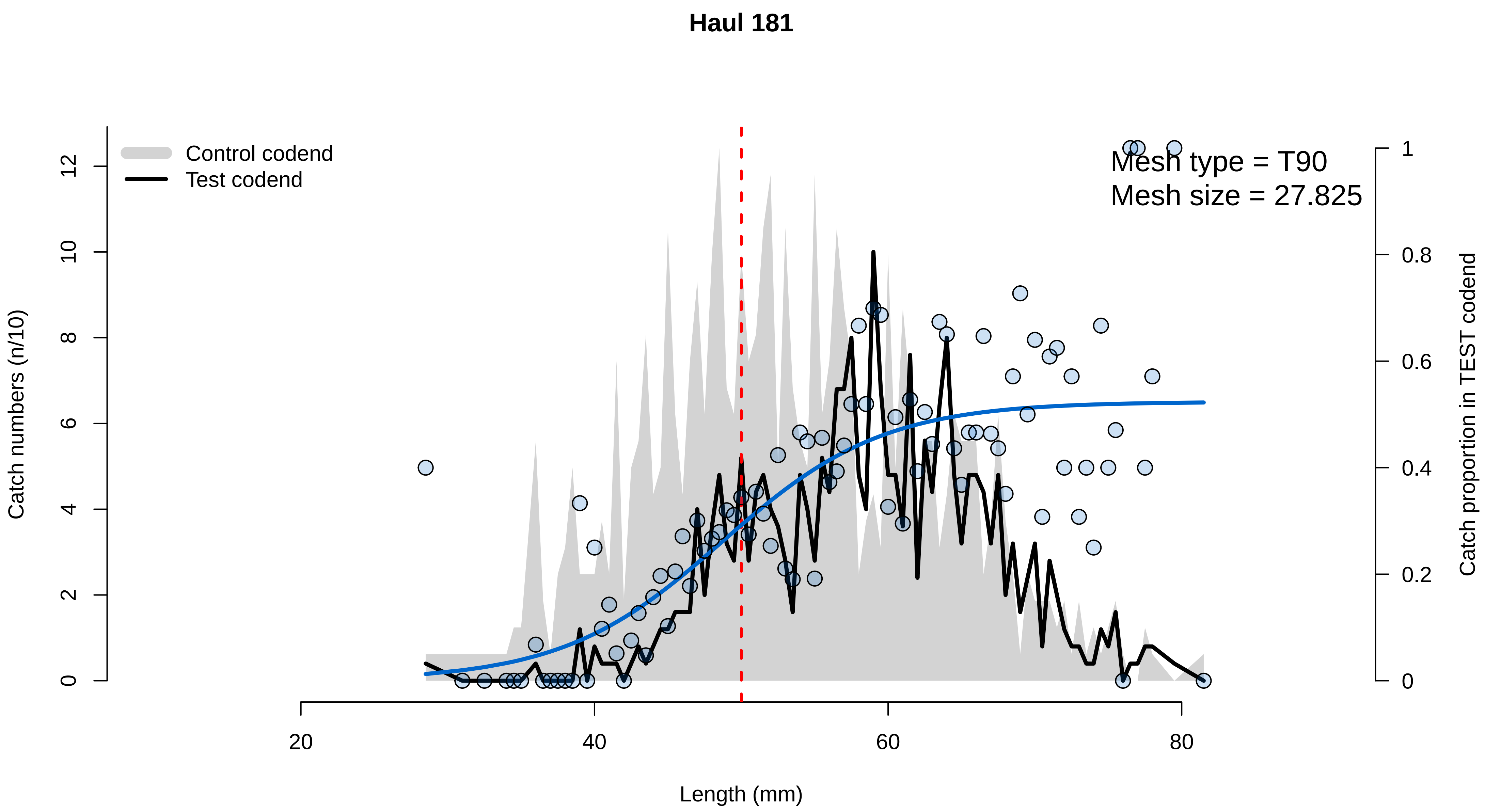
Haul 181
Control codend
Test codend
Mesh type = T90
Mesh size = 27.825
Catch numbers (n/10)
0
2
4
6
8
10
12
Catch proportion in TEST codend
0
0.2
0.4
0.6
0.8
1
20
40
60
80
Length (mm)

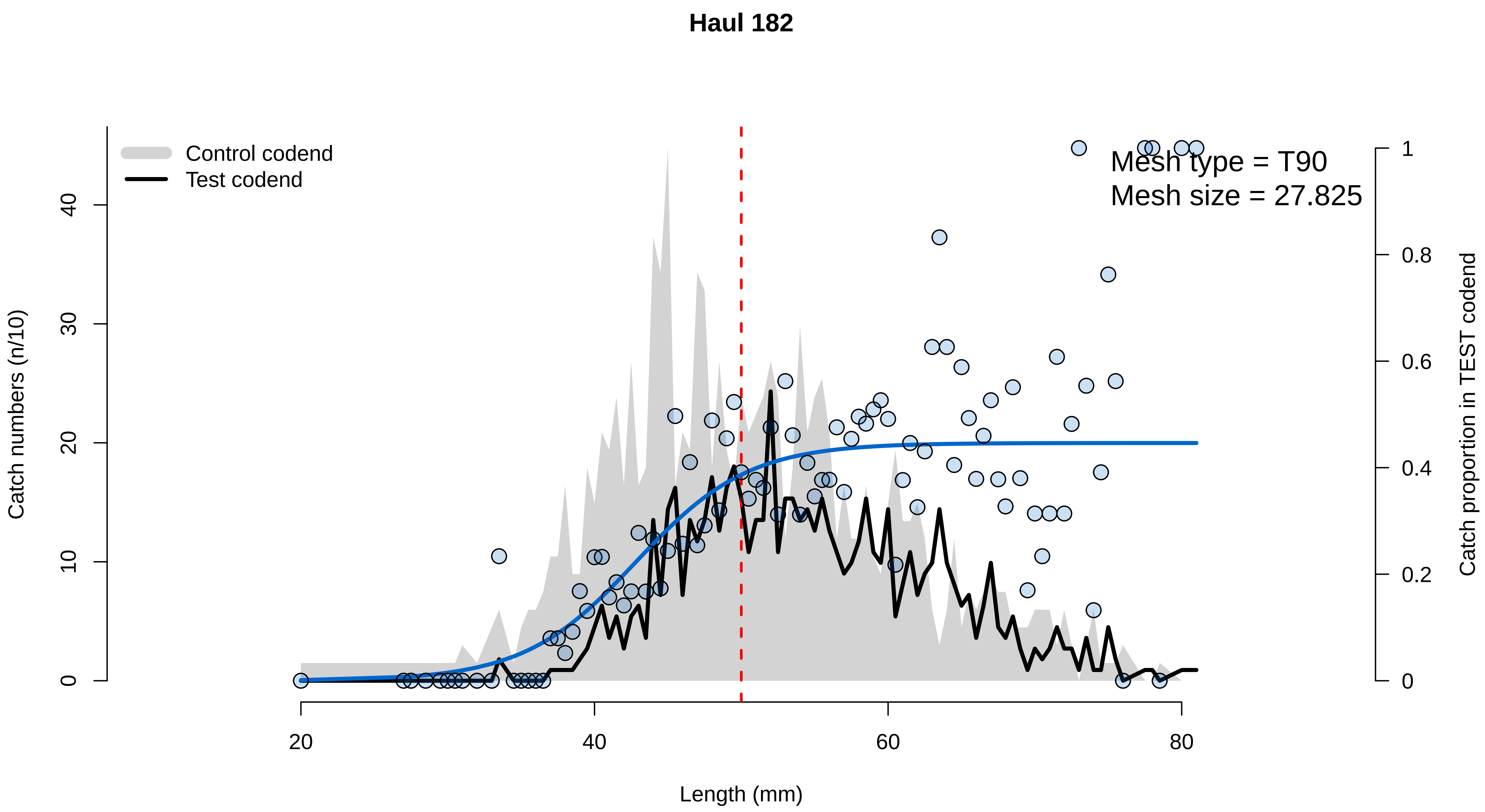

Haul 182
Control codend
Test codend
Mesh type = T90
Mesh size = 27.825
Catch numbers (n/10)
Catch proportion in TEST codend
Length (mm)
0
10
20
30
40
20
40
60
80
0
0.2
0.4
0.6
0.8
1

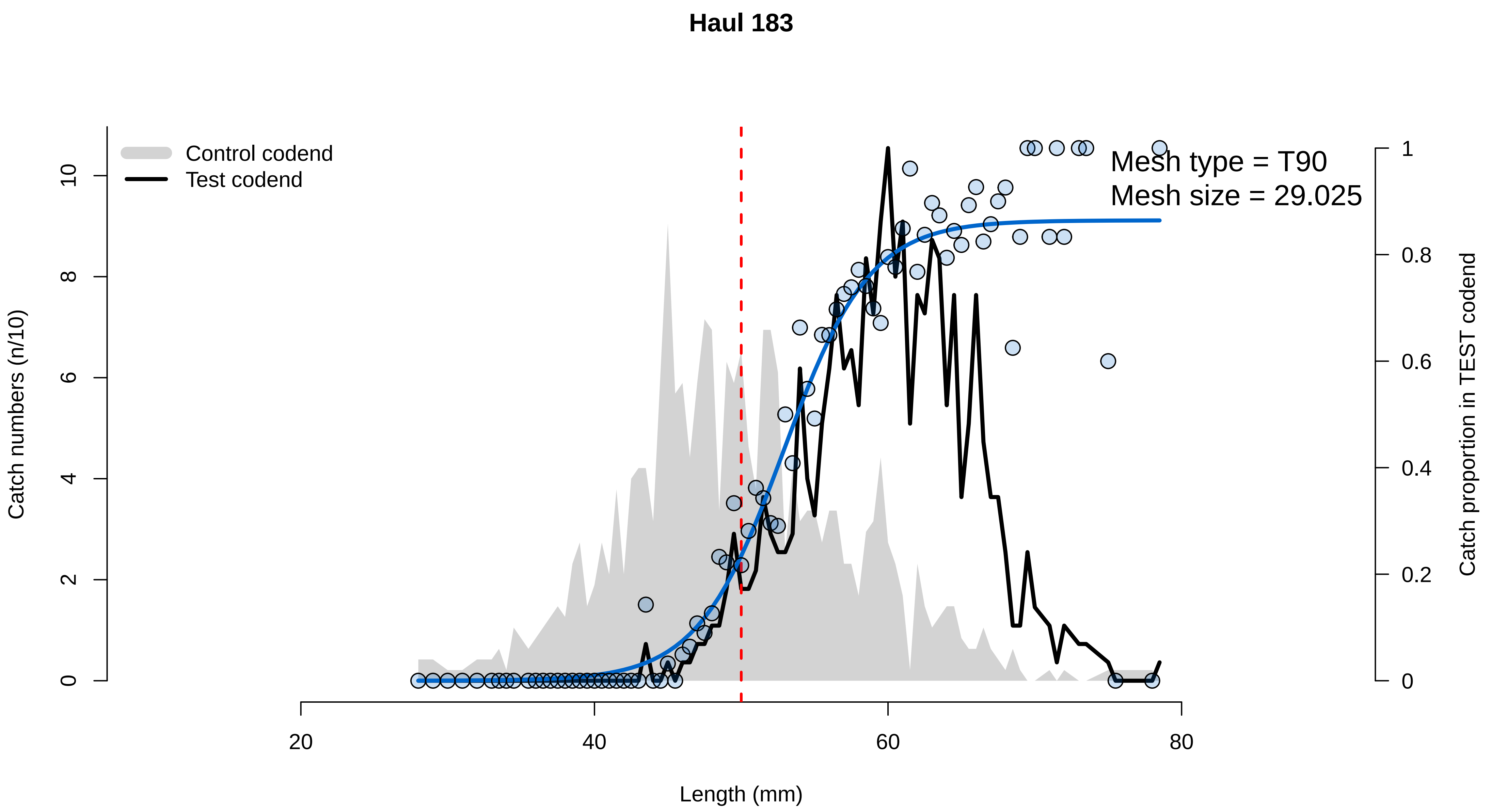
Haul 183
Control codend
Test codend
Mesh type = T90
Mesh size = 29.025
Catch numbers (n/10)
0
2
4
6
8
10
Catch proportion in TEST codend
0
0.2
0.4
0.6
0.8
1
20
40
60
80
Length (mm)

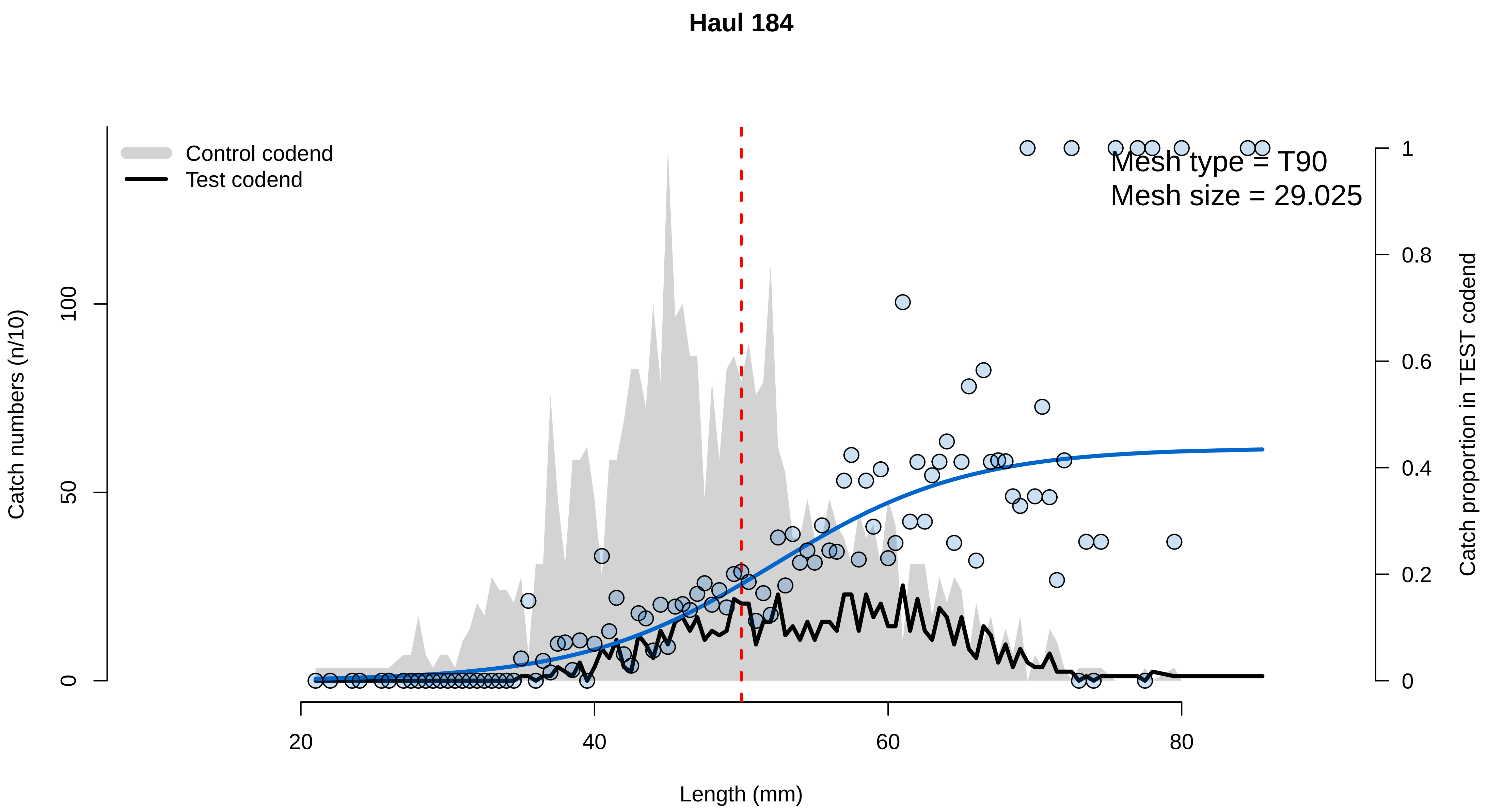
Haul 184
Control codend
Test codend
Mesh type = T90
Mesh size = 29.025
Catch numbers (n/10)
0
50
100
Catch proportion in TEST codend
0
0.2
0.4
0.6
0.8
1
20
40
60
80
Length (mm)

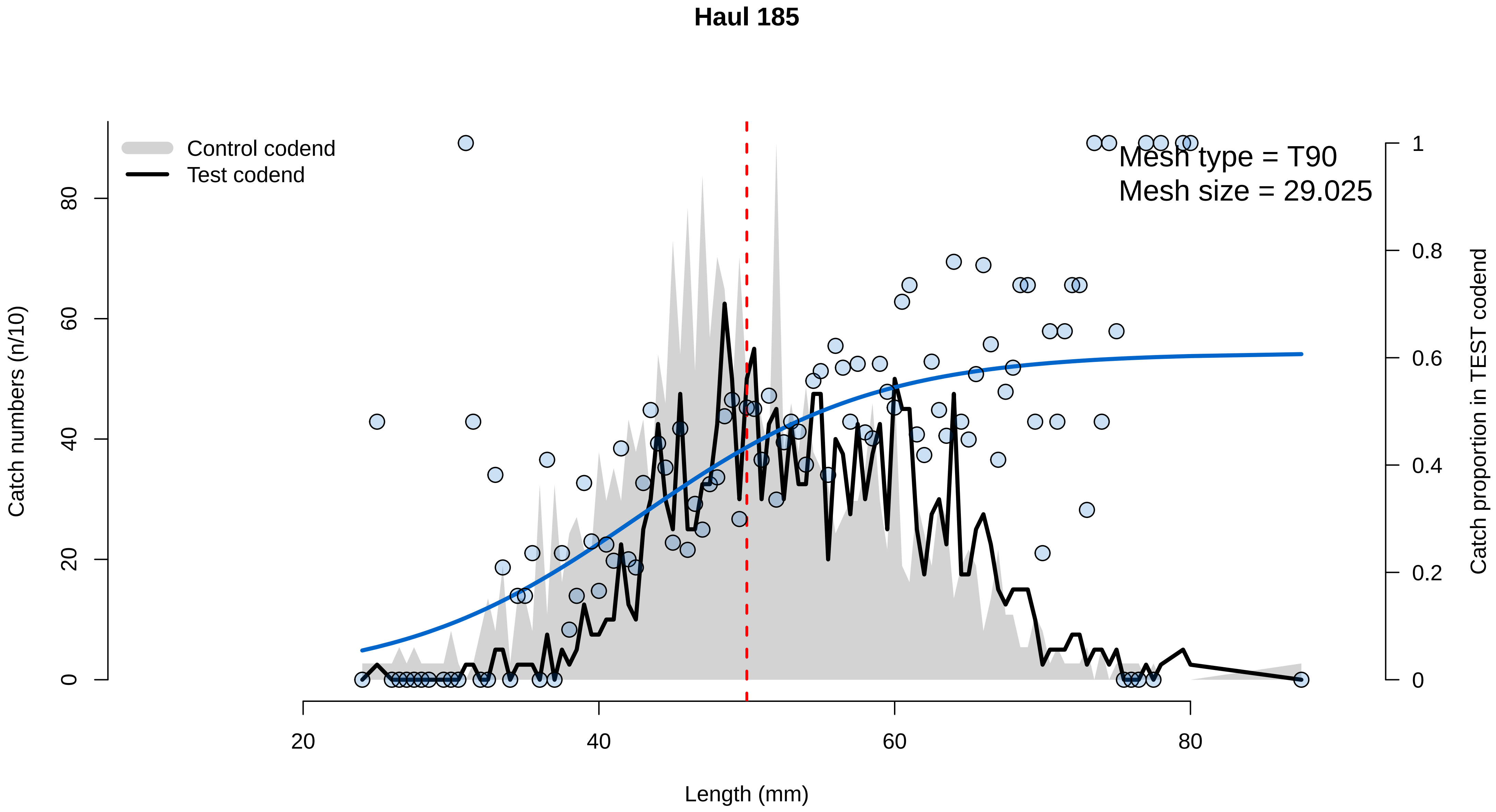
Haul 185
Control codend
Test codend
Mesh type = T90
Mesh size = 29.025
Catch numbers (n/10)
Catch proportion in TEST codend
Length (mm)
0
20
40
60
80
0
0.2
0.4
0.6
0.8
1
20
40
60
80

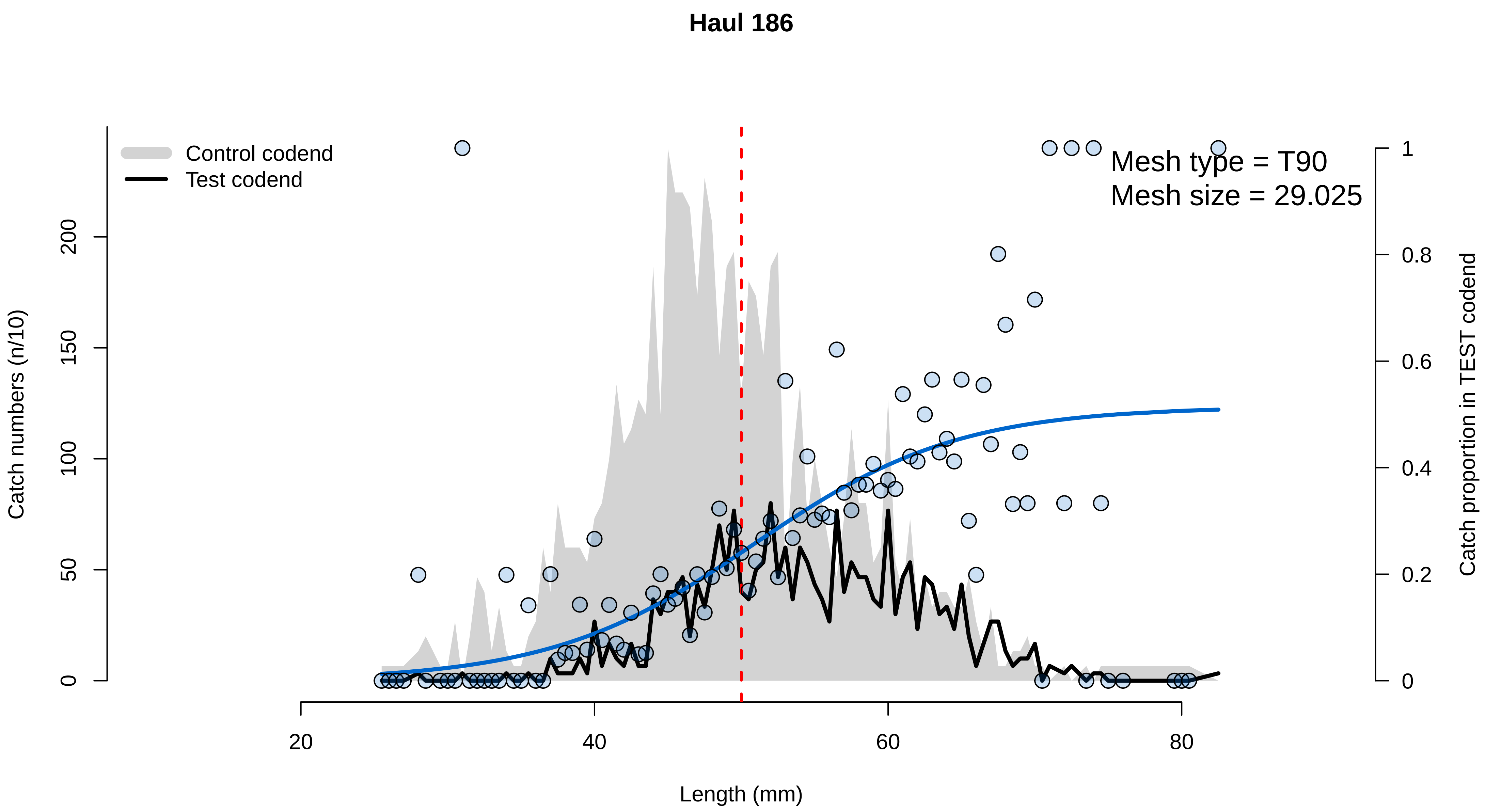

Haul 186
Control codend
Test codend
Mesh type = T90
Mesh size = 29.025
Catch numbers (n/10)
0
50
100
150
200
Catch proportion in TEST codend
0
0.2
0.4
0.6
0.8
1
20
40
60
80
Length (mm)

Haul 187

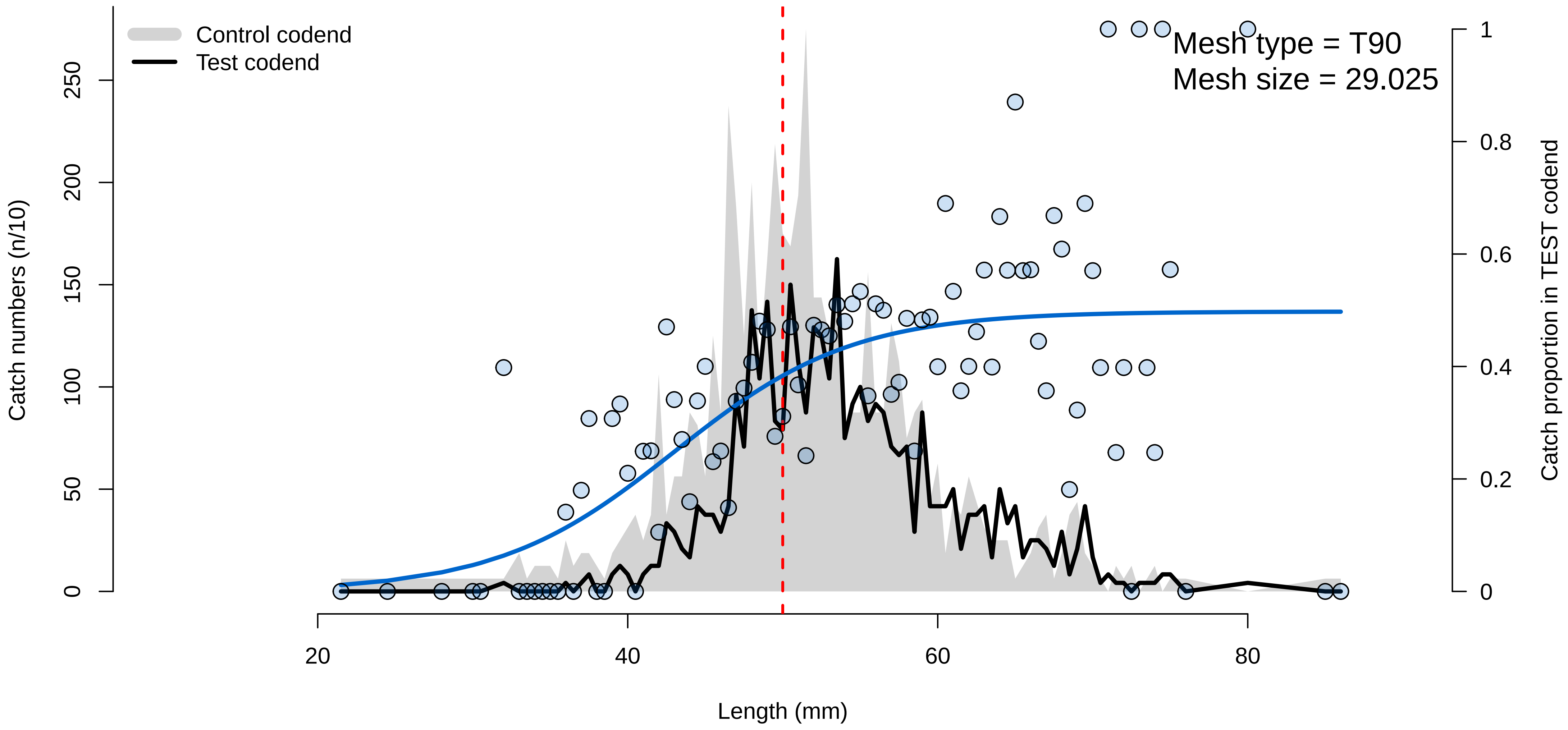
Control codend
Test codend
Mesh type = T90
Mesh size = 29.025
Catch numbers (n/10)
0
50
100
150
200
250
Catch proportion in TEST codend
0
0.2
0.4
0.6
0.8
1
Length (mm)
20
40
60
80

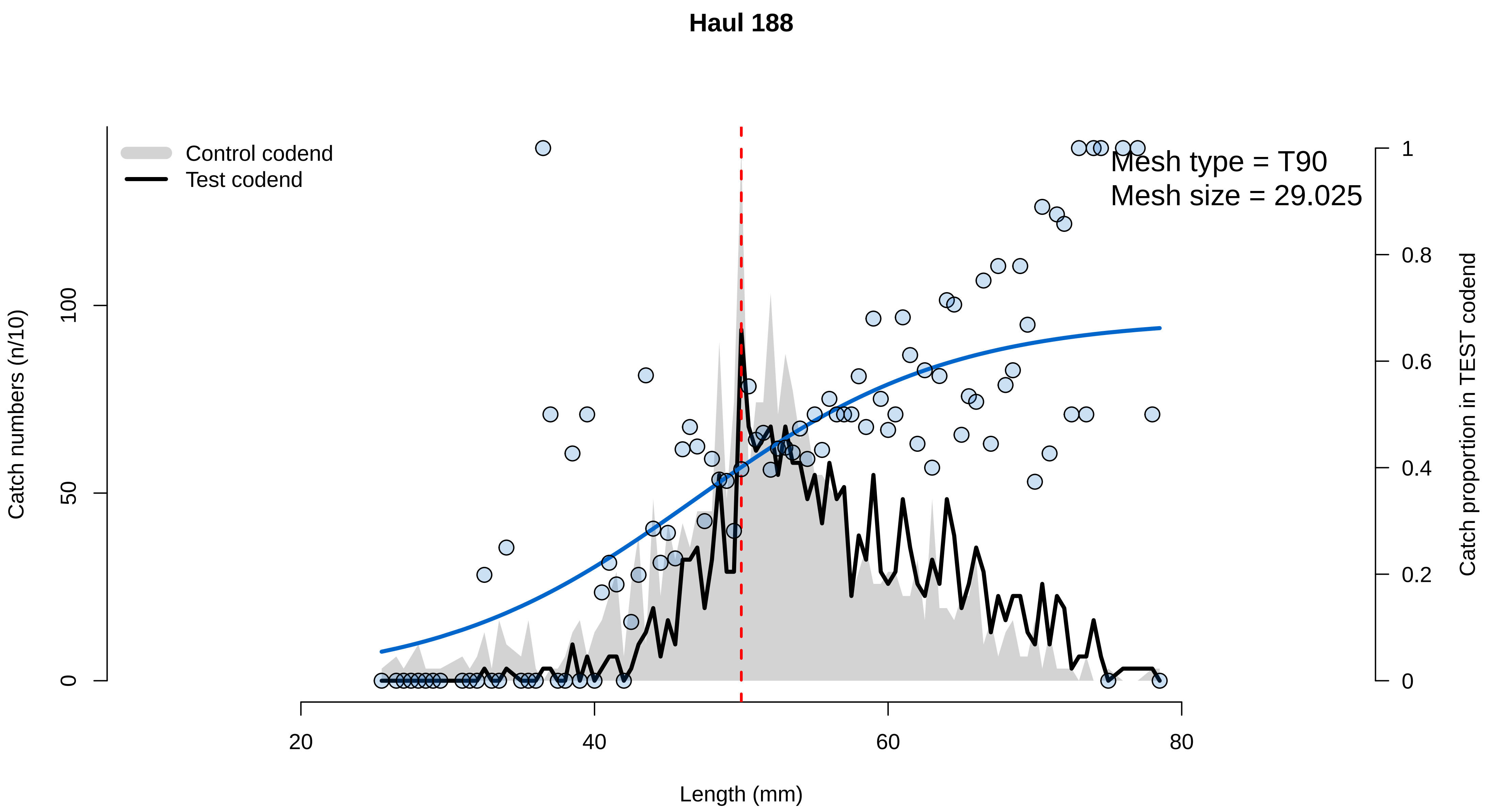

Haul 188
Control codend
Test codend
Mesh type = T90
Mesh size = 29.025
Catch numbers (n/10)
0
50
100
Catch proportion in TEST codend
0
0.2
0.4
0.6
0.8
1
20
40
60
80
Length (mm)

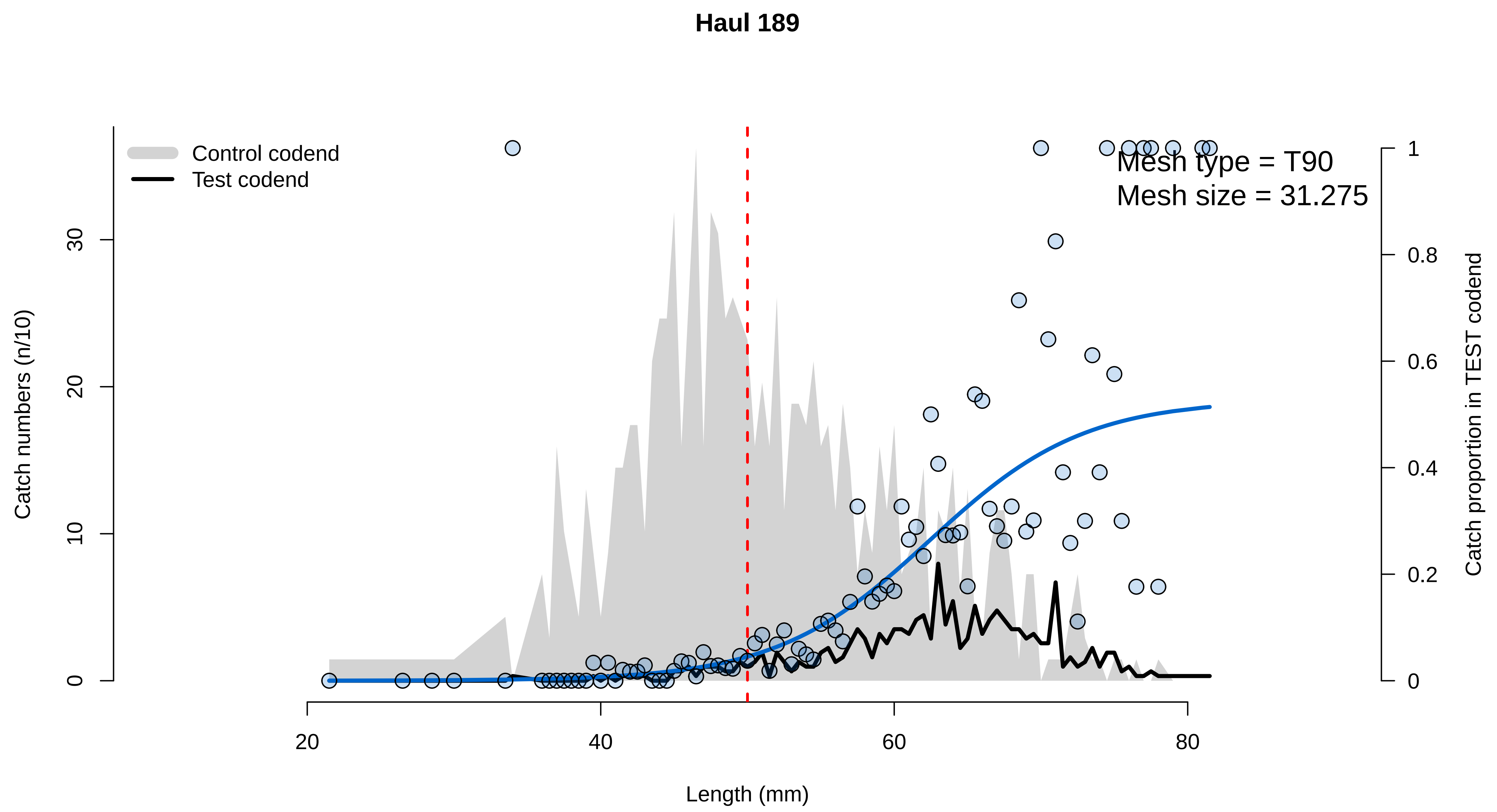
Haul 189
Control codend
Test codend
Mesh type = T90
Mesh size = 31.275
Catch numbers (n/10)
0
10
20
30
Catch proportion in TEST codend
0
0.2
0.4
0.6
0.8
1
20
40
60
80
Length (mm)

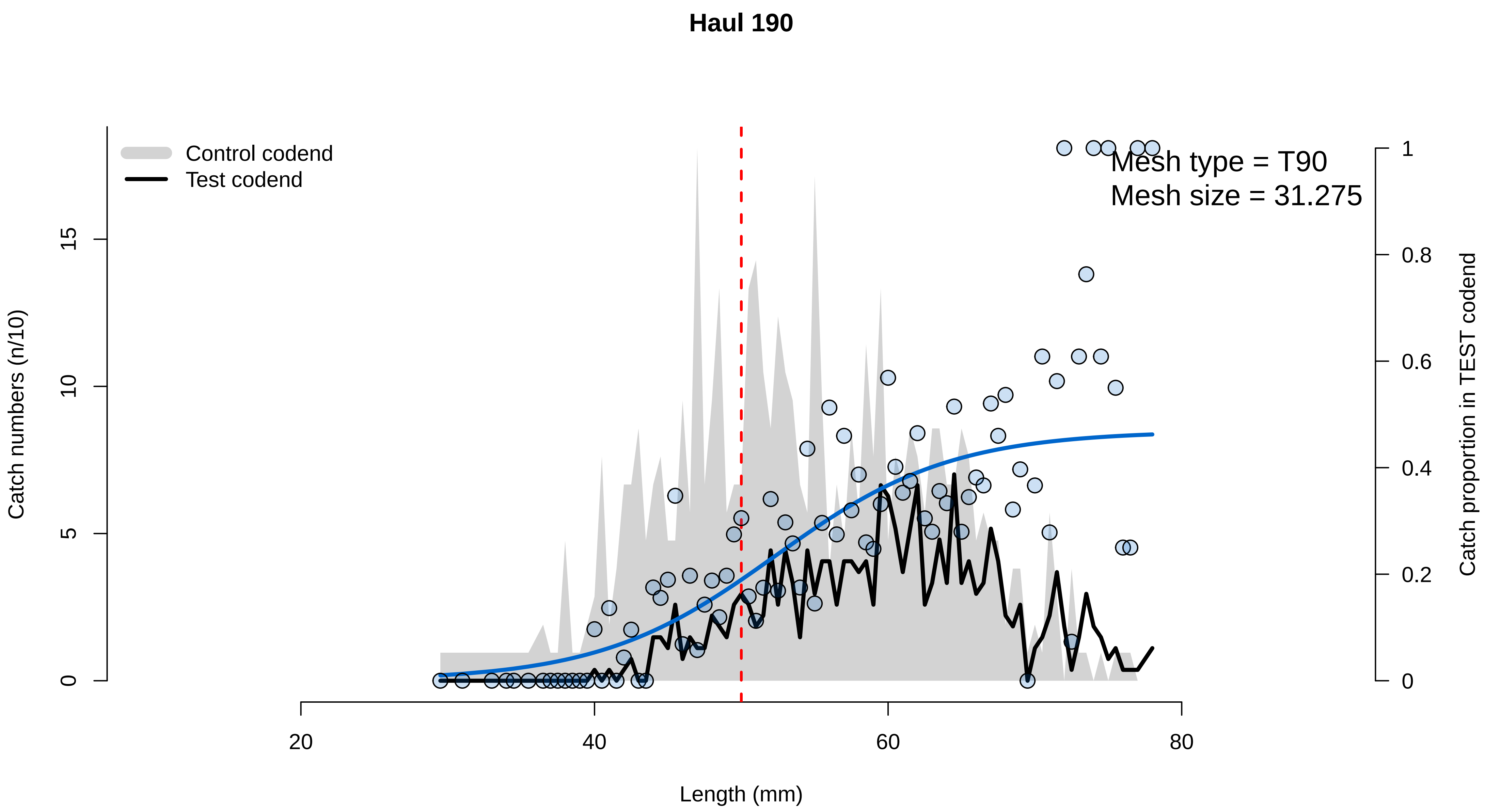

Haul 190
Control codend
Test codend
Mesh type = T90
Mesh size = 31.275
Catch numbers (n/10)
0
5
10
15
Catch proportion in TEST codend
0
0.2
0.4
0.6
0.8
1
Length (mm)
20
40
60
80

Haul 191

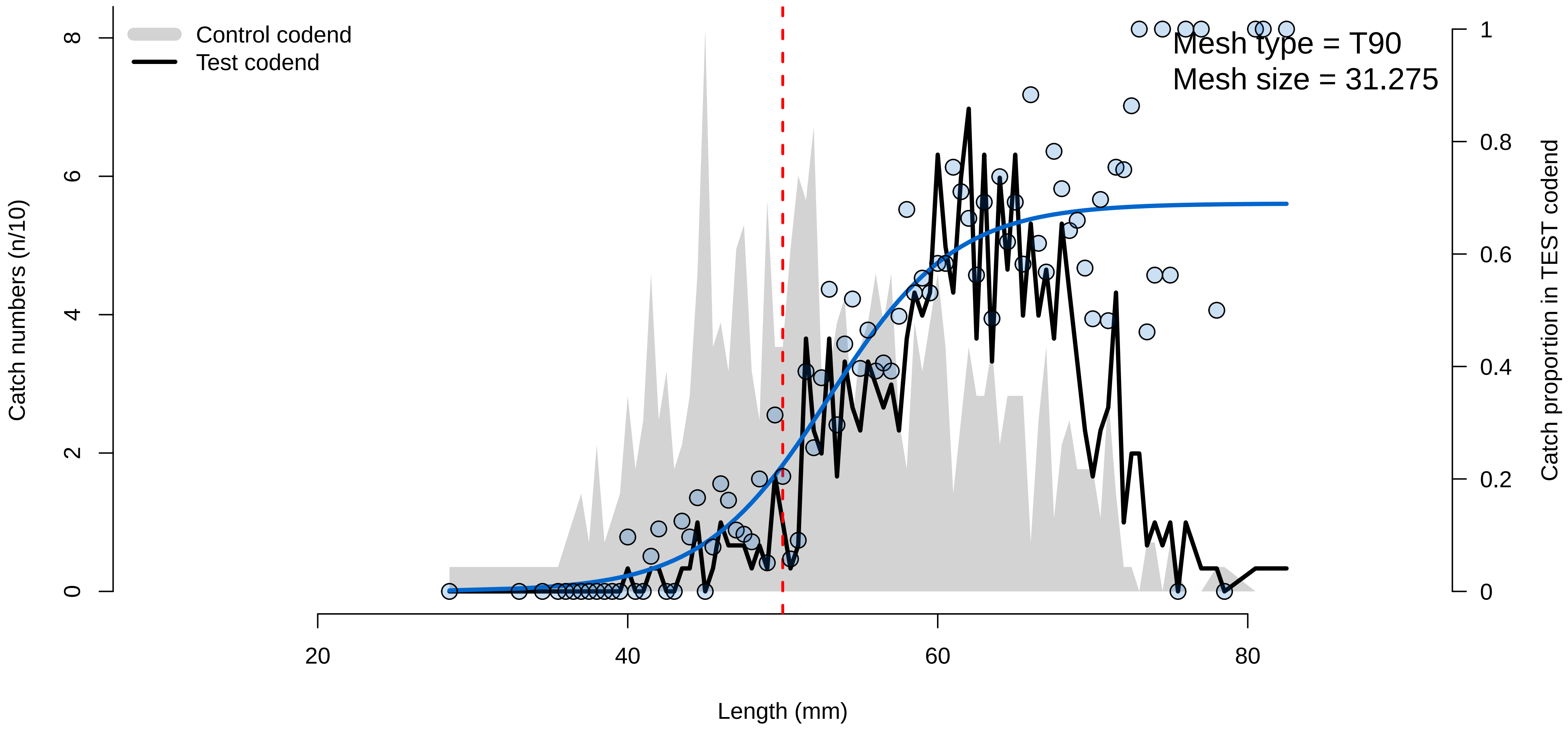

Control codend
Test codend
Mesh type = T90
Mesh size = 31.275
Catch numbers (n/10)
0
2
4
6
8
Catch proportion in TEST codend
0
0.2
0.4
0.6
0.8
1
20
40
60
80
Length (mm)

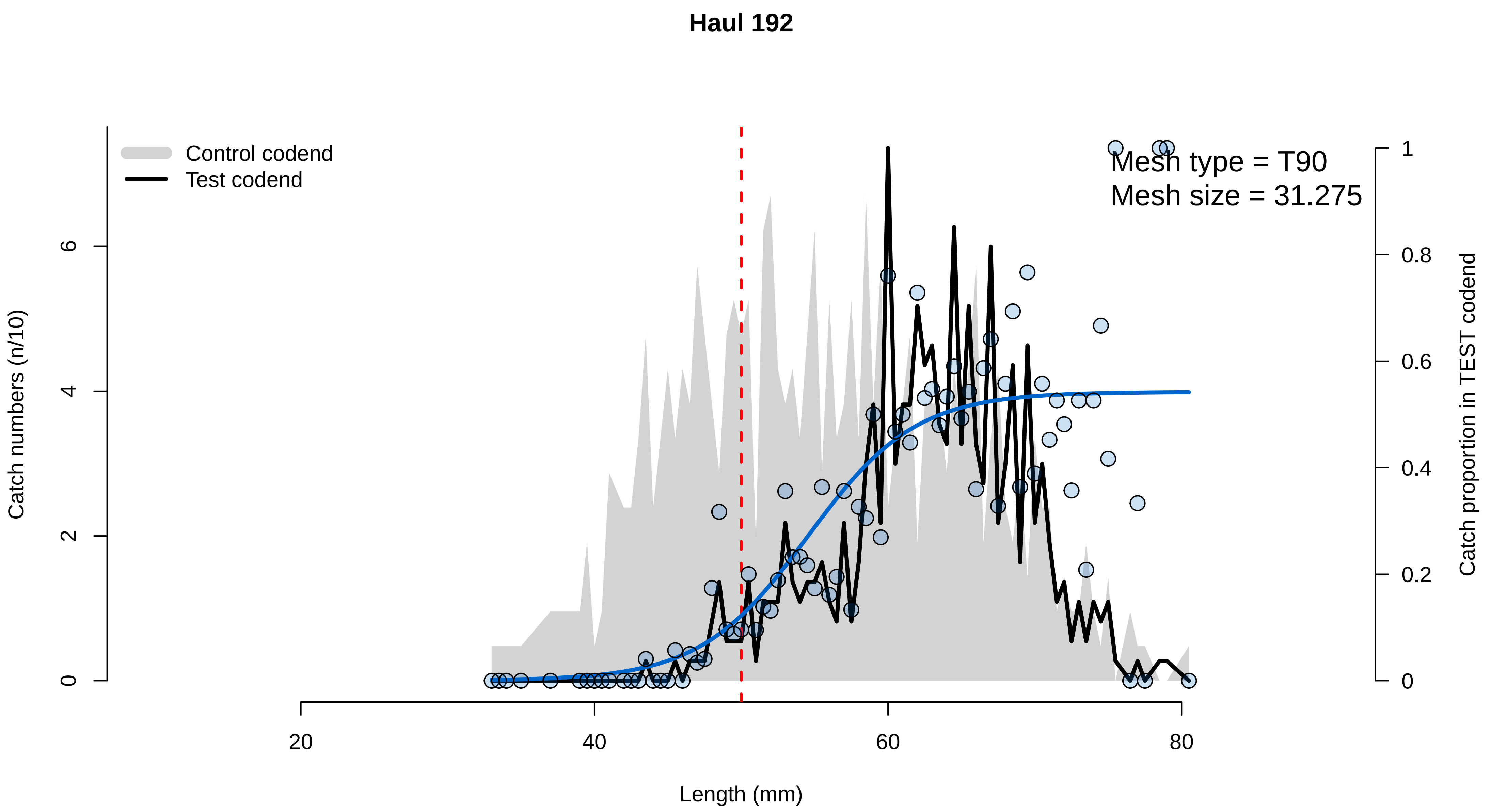

Haul 192
Control codend
Test codend
Mesh type = T90
Mesh size = 31.275
Catch numbers (n/10)
0
2
4
6
Catch proportion in TEST codend
0
0.2
0.4
0.6
0.8
1
20
40
60
80
Length (mm)

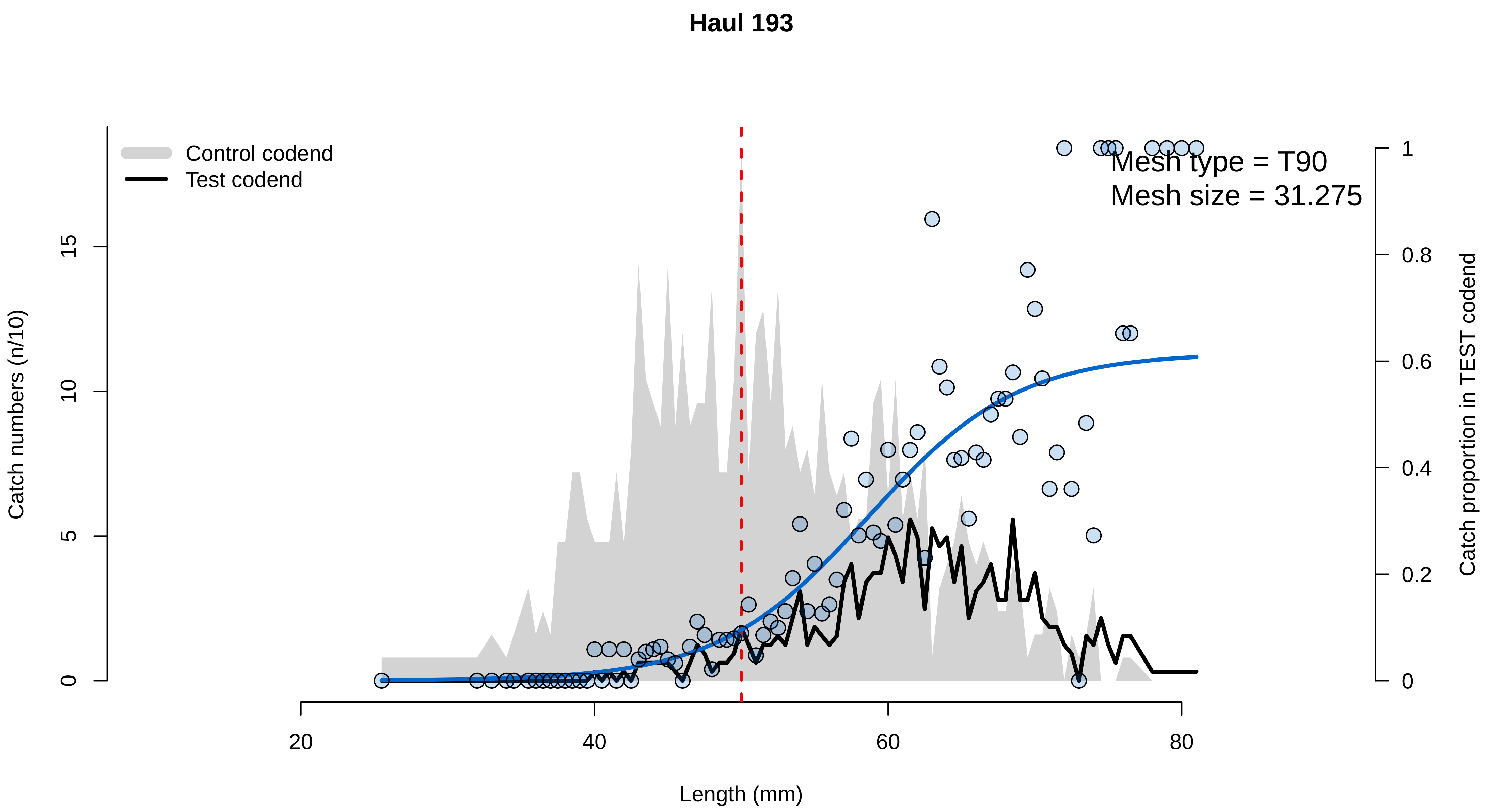
Haul 193
Control codend
Test codend
Mesh type = T90
Mesh size = 31.275
Catch numbers (n/10)
0
5
10
15
Catch proportion in TEST codend
0
0.2
0.4
0.6
0.8
1
20
40
60
80
Length (mm)

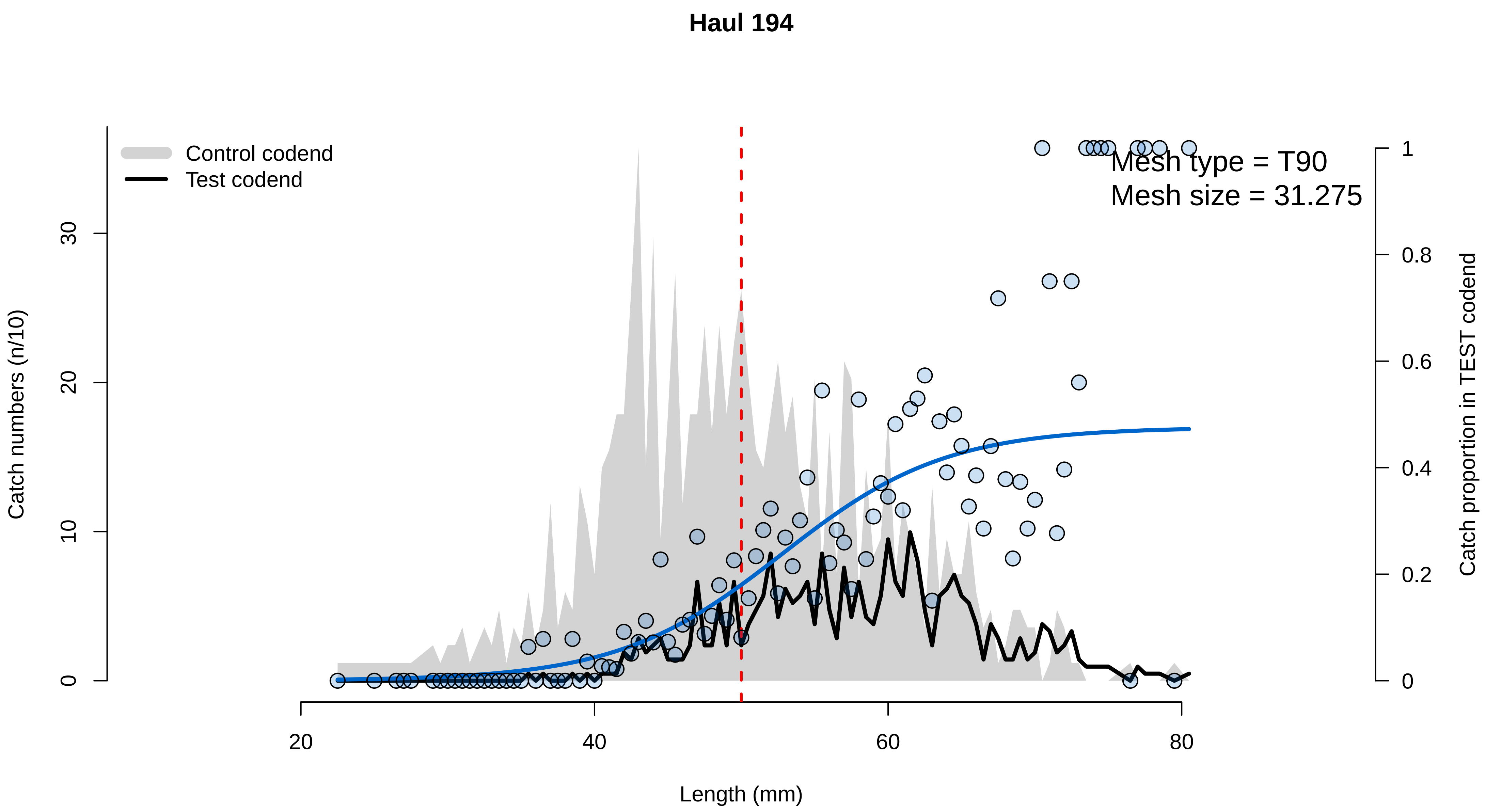
Haul 194
Control codend
Test codend
Mesh type = T90
Mesh size = 31.275
Catch numbers (n/10)
0
10
20
30
Catch proportion in TEST codend
0
0.2
0.4
0.6
0.8
1
20
40
60
80
Length (mm)

Haul 195

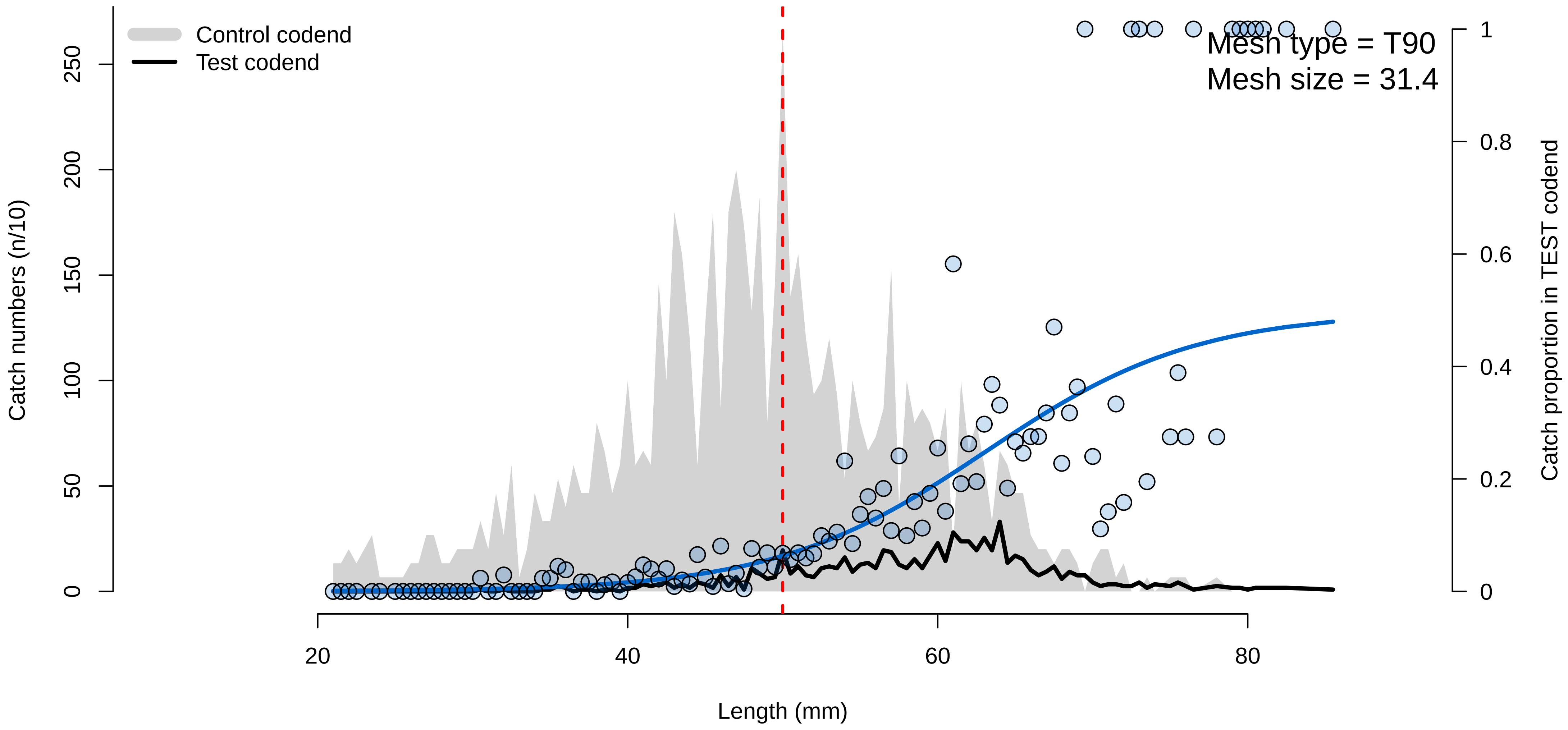
Control codend
Test codend
Mesh type = T90
Mesh size = 31.4
Catch numbers (n/10)
0
50
100
150
200
250
Catch proportion in TEST codend
0
0.2
0.4
0.6
0.8
1
20
40
60
80
Length (mm)

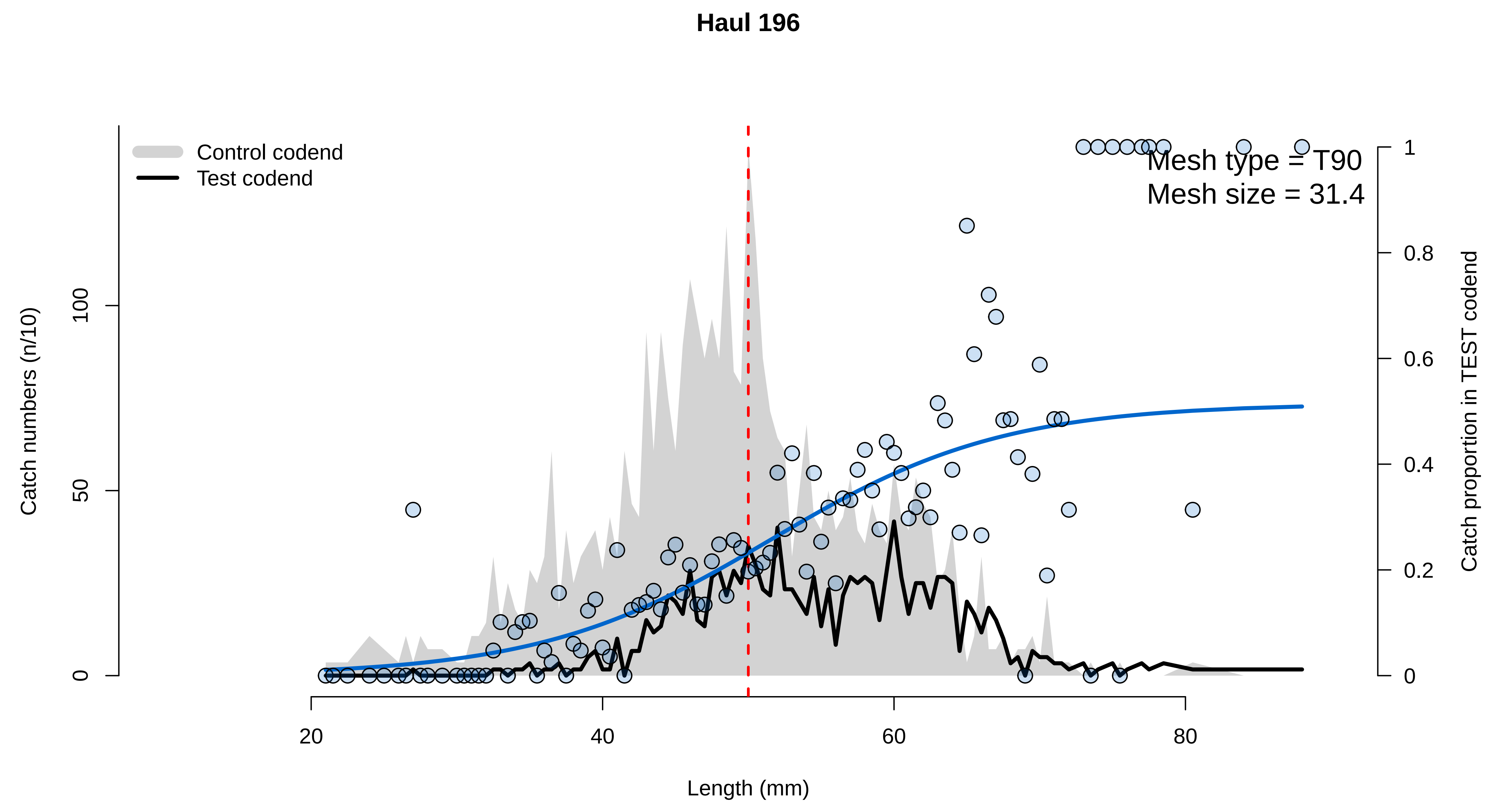
Haul 196
Control codend
Test codend
Mesh type = T90
Mesh size = 31.4
Catch numbers (n/10)
0
50
100
20
40
60
80
Length (mm)
Catch proportion in TEST codend
0
0.2
0.4
0.6
0.8
1

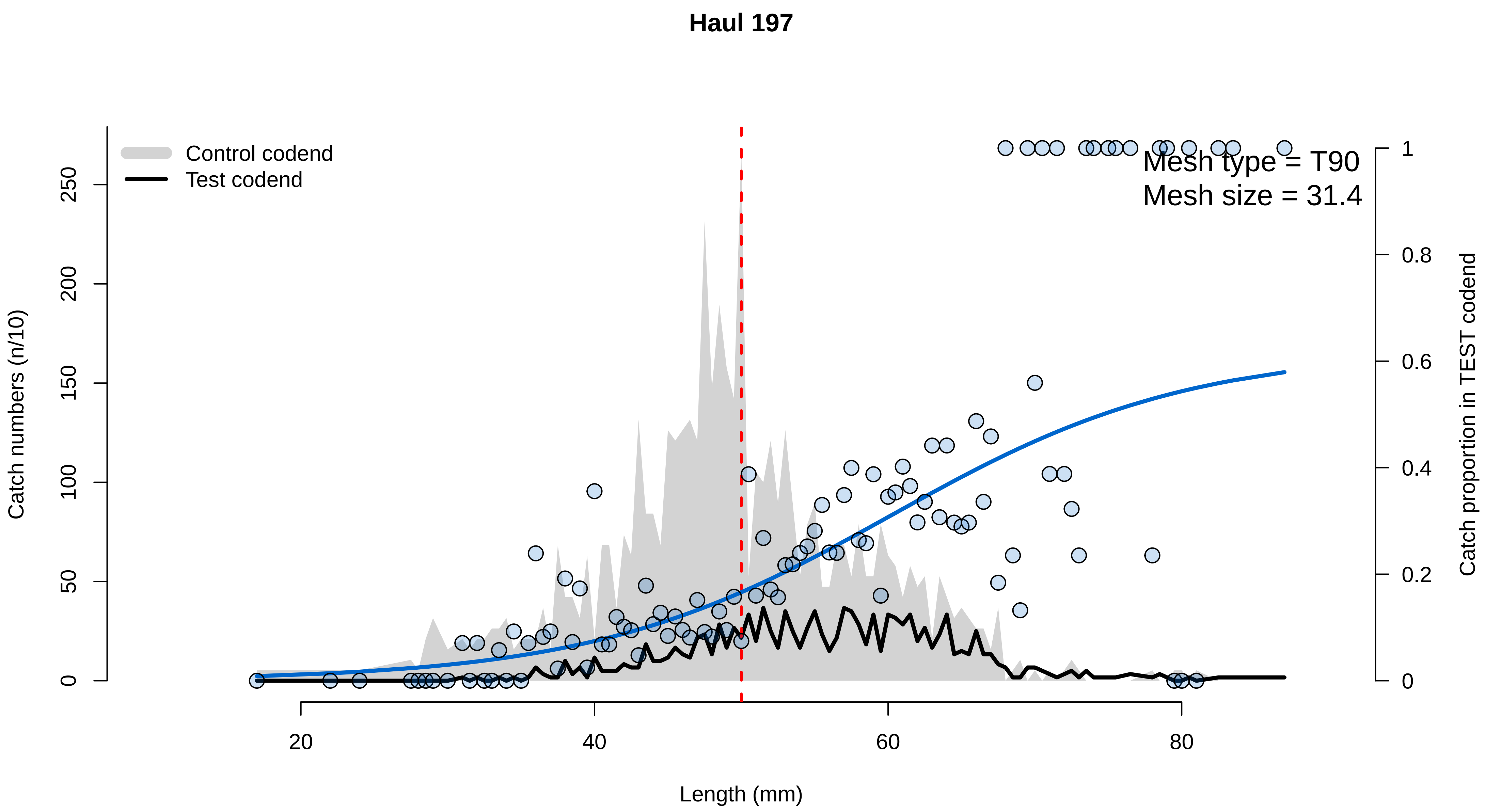

Haul 197
Control codend
Test codend
Mesh type = T90
Mesh size = 31.4
Catch numbers (n/10)
0
50
100
150
200
250
Catch proportion in TEST codend
0
0.2
0.4
0.6
0.8
1
20
40
60
80
Length (mm)

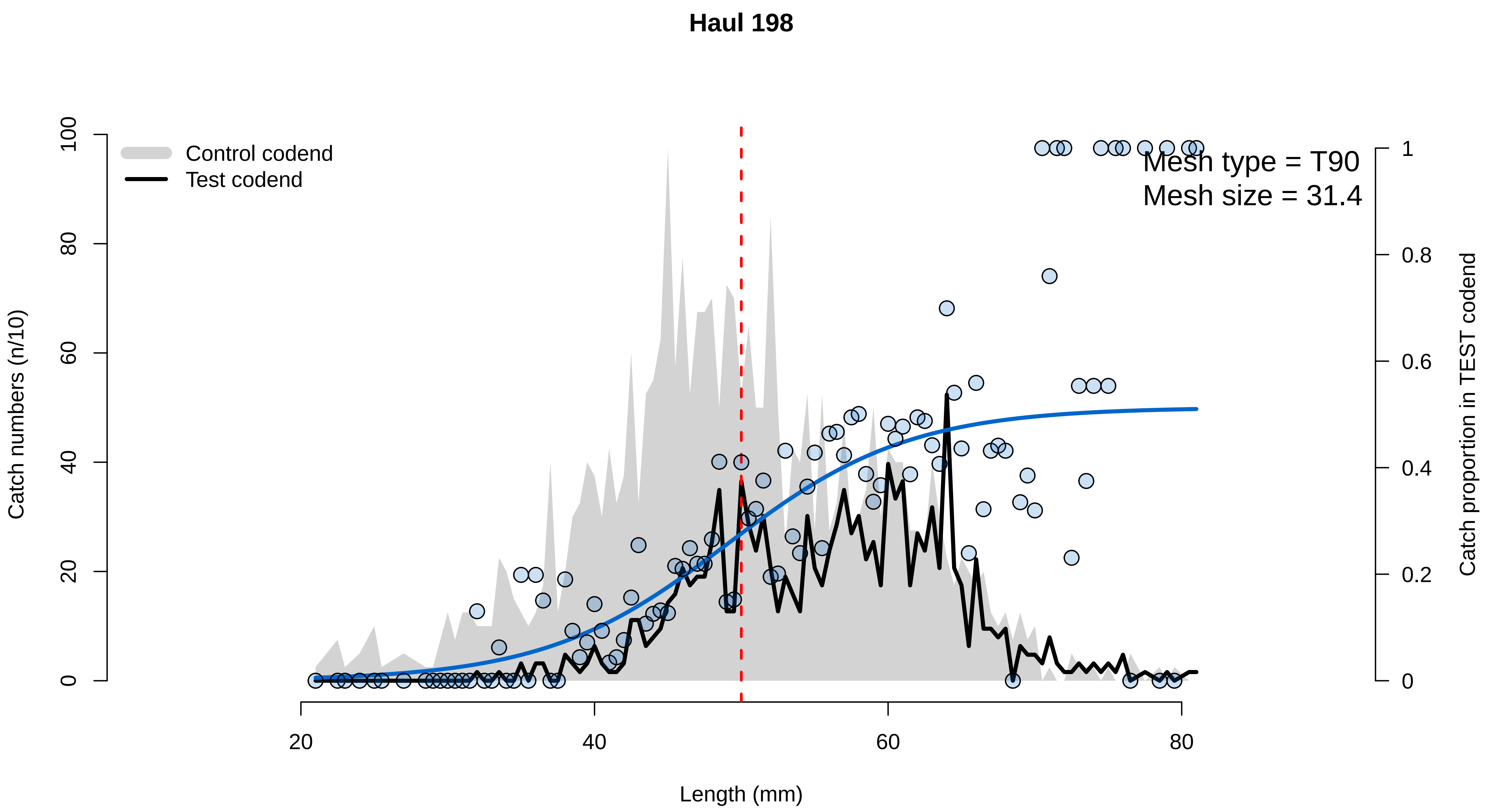

Haul 198
Control codend
Test codend
Mesh type = T90
Mesh size = 31.4
Catch numbers (n/10)
0
20
40
60
80
100
Catch proportion in TEST codend
0
0.2
0.4
0.6
0.8
1
20
40
60
80
Length (mm)

Haul 199

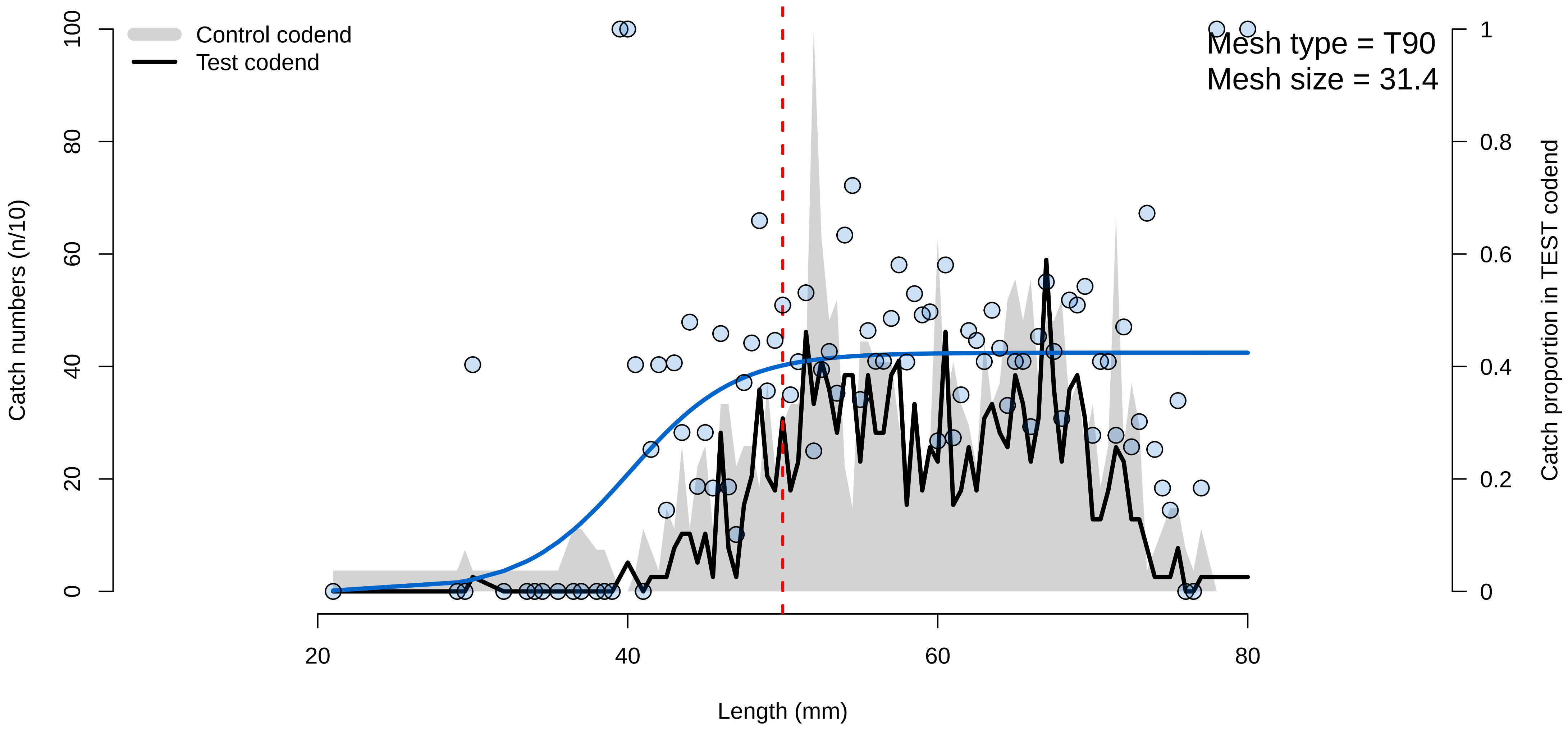

Control codend
Test codend
Mesh type = T90
Mesh size = 31.4
Catch numbers (n/10)
Catch proportion in TEST codend
Length (mm)
0
20
40
60
80
100
0.2
0.4
0.6
0.8
1
20
40
60
80

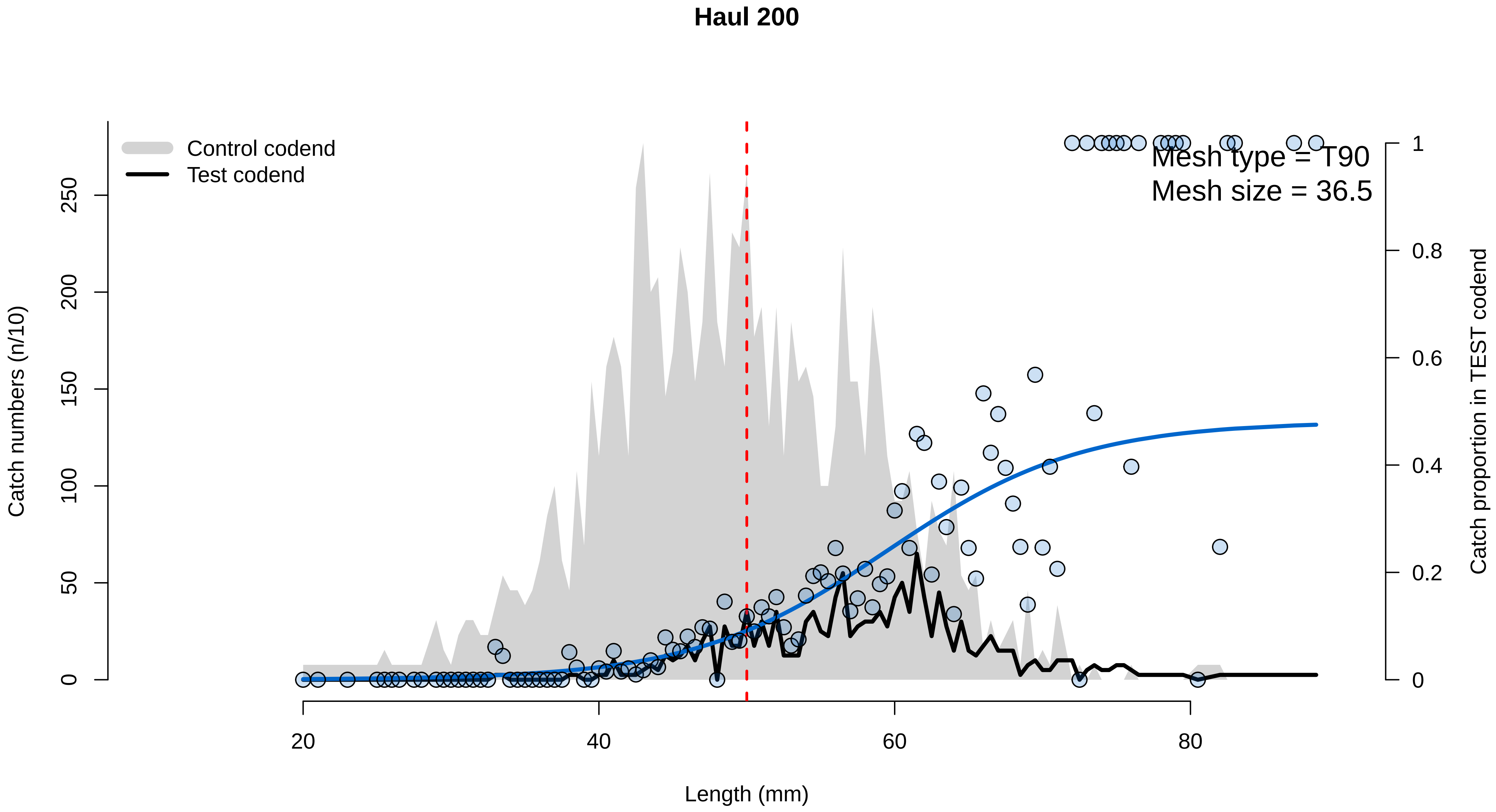

Haul 200
Control codend
Test codend
Mesh type = T90
Mesh size = 36.5
Catch numbers (n/10)
0
50
100
150
200
250
Catch proportion in TEST codend
0
0.2
0.4
0.6
0.8
1
20
40
60
80
Length (mm)

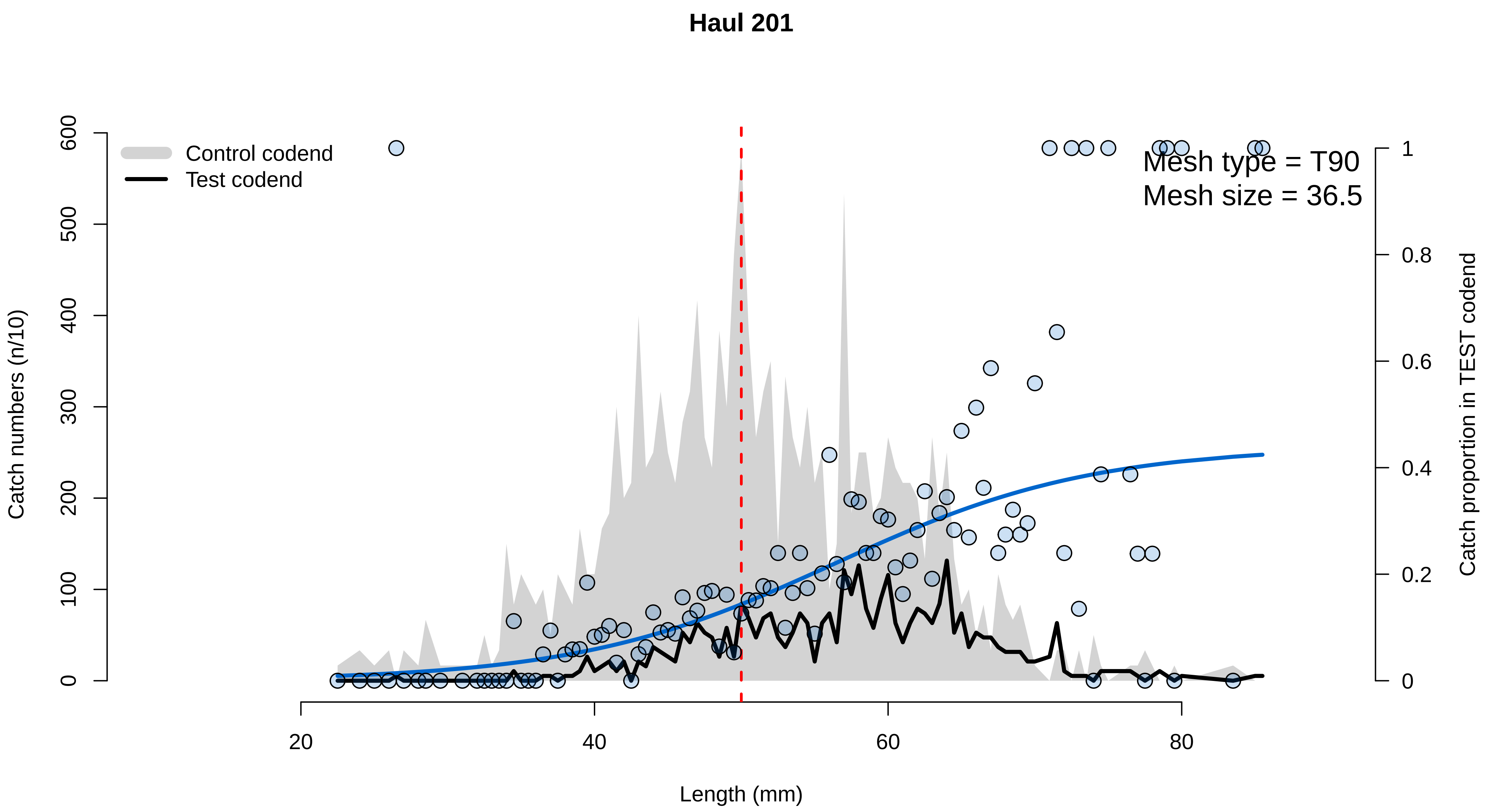
Haul 201
Control codend
Test codend
Mesh type = T90
Mesh size = 36.5
Catch numbers (n/10)
0
100
200
300
400
500
600
Catch proportion in TEST codend
0
0.2
0.4
0.6
0.8
1
20
40
60
80
Length (mm)

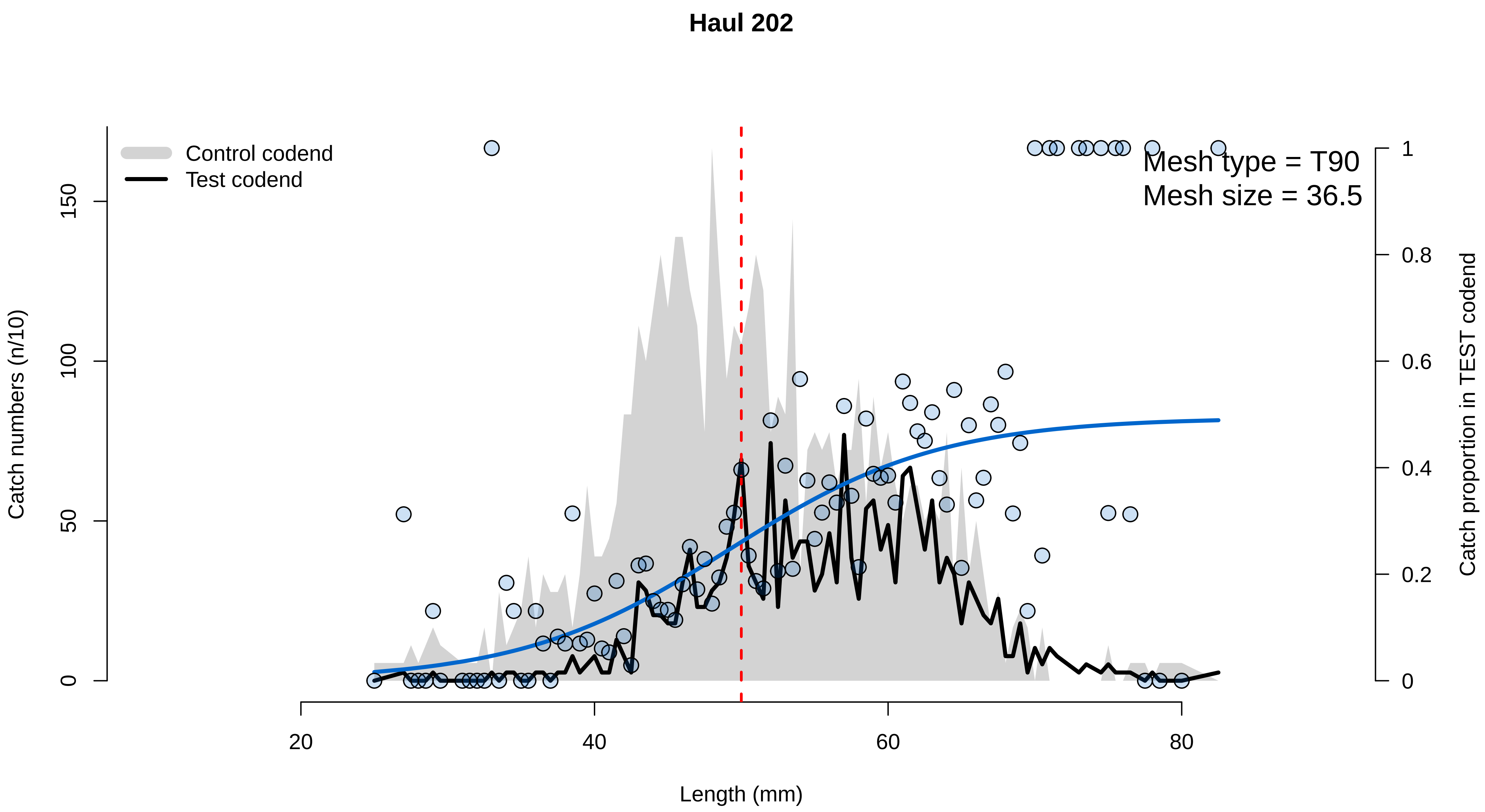

Haul 202
Control codend
Test codend
Mesh type = T90
Mesh size = 36.5
Catch numbers (n/10)
0
50
100
150
Catch proportion in TEST codend
0
0.2
0.4
0.6
0.8
1
20
40
60
80
Length (mm)

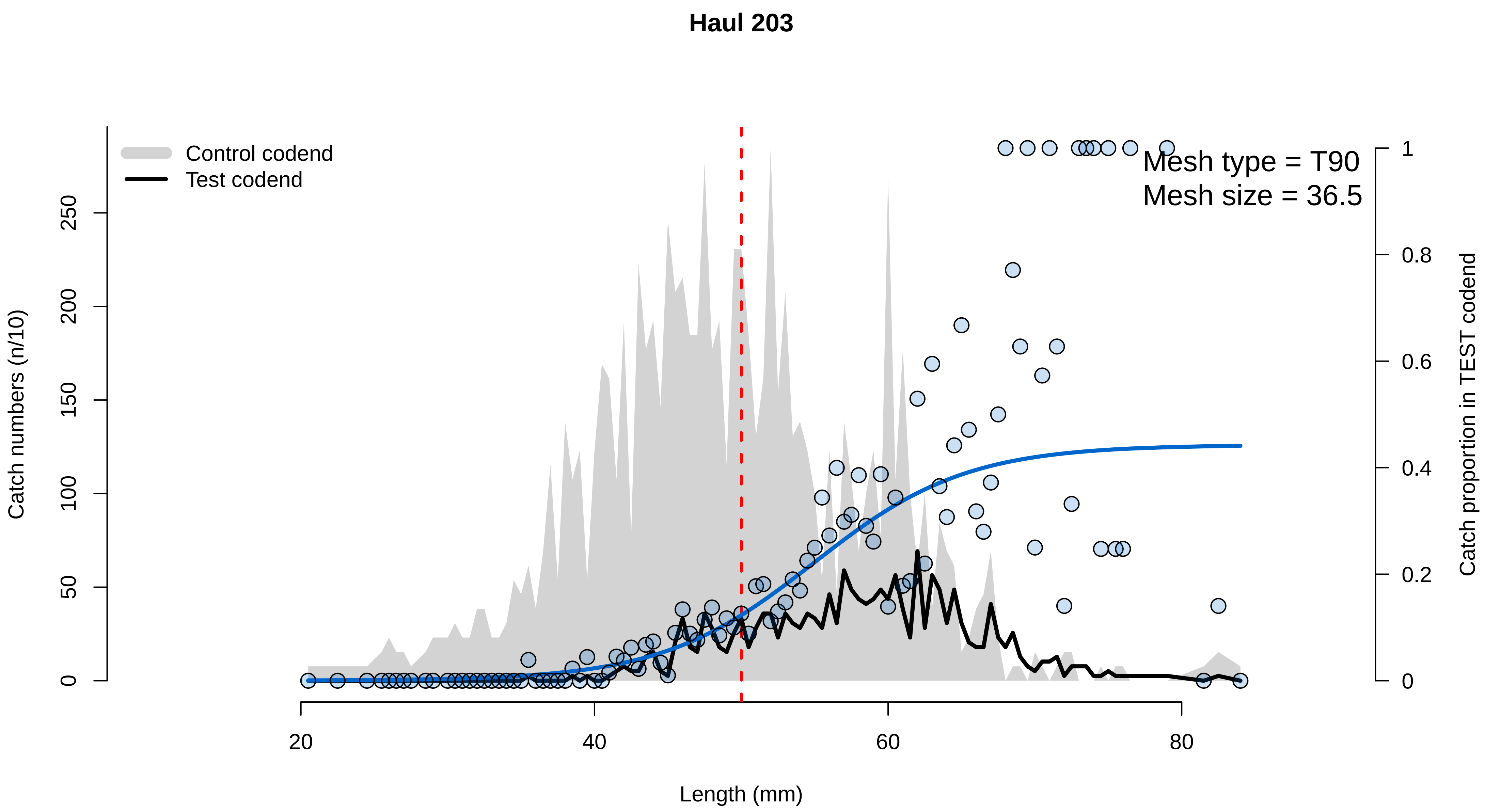

Haul 203
Control codend
Test codend
Mesh type = T90
Mesh size = 36.5
Catch numbers (n/10)
0
50
100
150
200
250
Catch proportion in TEST codend
0
0.2
0.4
0.6
0.8
1
Length (mm)
20
40
60
80

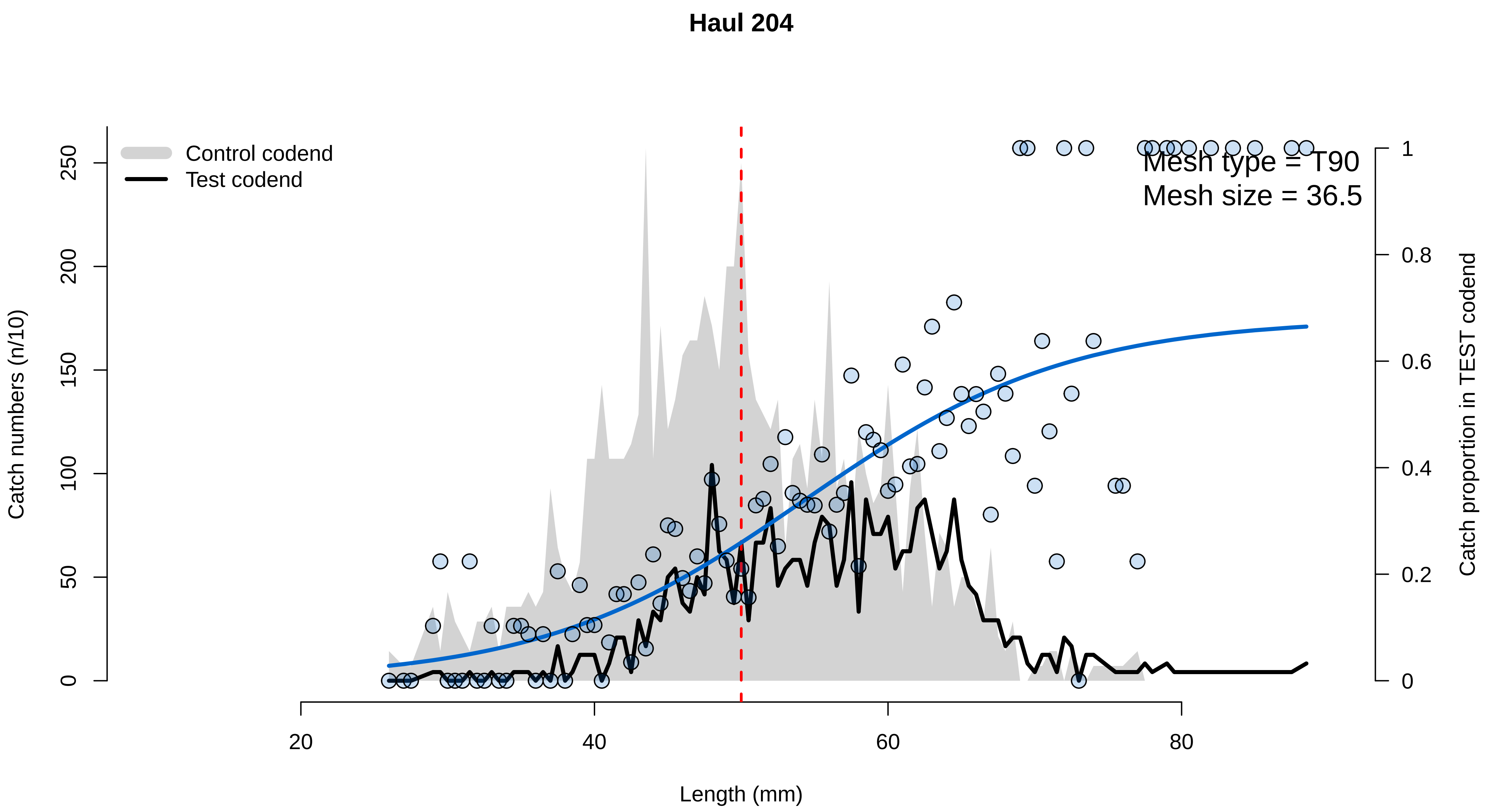
Haul 204
Control codend
Test codend
Mesh type = T90
Mesh size = 36.5
Catch numbers (n/10)
Catch proportion in TEST codend
Length (mm)
0
50
100
150
200
250
20
40
60
80
0
0.2
0.4
0.6
0.8
1

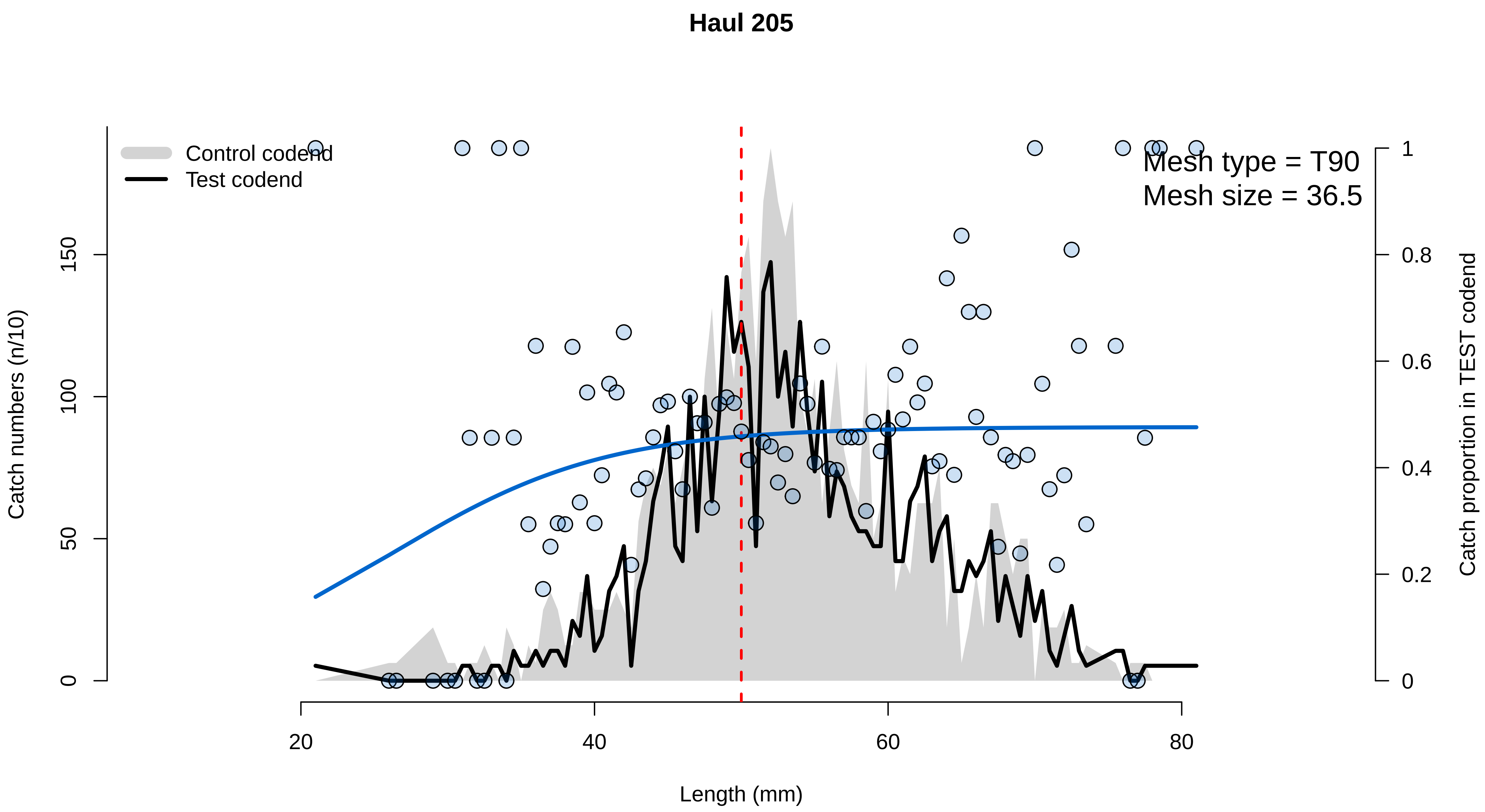

Haul 205
Control codend
Test codend
Mesh type = T90
Mesh size = 36.5
Catch numbers (n/10)
0
50
100
150
Catch proportion in TEST codend
0
0.2
0.4
0.6
0.8
1
20
40
60
80
Length (mm)
